# A regulatory mutant on *TRIM26* conferring the risk of nasopharyngeal carcinoma by inducing low immune response

**DOI:** 10.1002/cam4.1537

**Published:** 2018-06-28

**Authors:** Xiao‐Ming Lyu, Xiao‐Wei Zhu, Manli Zhao, Xian‐Bo Zuo, Zhong‐Xi Huang, Xiao Liu, Tao Jiang, Xue‐Xi Yang, Xin Li, Xiao‐Bing Long, Jian‐Guo Wang, Jin‐Bang Li, Ming‐Yu Han, Shuang Wang, Teng‐Fei Liu, Bo Zhang, Tao Sun, Zhi Cheng, Mo‐Chang Qiu, Lei Dong, Lu Zheng, Long‐Cheng Zhang, Jia‐Hong Wang, Gan‐Guan Wei, Kaitai Yao, Qian Wang, Hou‐Feng Zheng, Xin Li

**Affiliations:** ^1^ Department of laboratory medicine The Third Affiliated Hospital Southern Medical University Guangzhou China; ^2^ Shenzhen Key Laboratory of Viral Oncology the Clinical Innovation & Research Center (CIRC) Shenzhen Hospital Southern Medical University Shenzhen China; ^3^ Zhujiang Hospital Southern Medical University Guangzhou China; ^4^ Institute of Basic Medical Sciences Westlake Institute for Advanced Study Westlake University Hangzhou Zhejiang China; ^5^ Institute of Aging Research and the Affiliated Hospital School of Medicine Hangzhou Normal University Hangzhou Zhejiang China; ^6^ School of Life Sciences Fudan University Shanghai China; ^7^ Department of Pathology School of Medicine The Children Hospital of Zhejiang University Hangzhou China; ^8^ Institute of Dermatology and Department of Dermatology No. 1 Hospital Anhui Medical University Hefei Anhui China; ^9^ Cancer Research Institute and the Provincial Key Laboratory of Functional Proteomics Southern Medical University Guangzhou China; ^10^ Beijing Genome Institute (BGI) Shenzhen China; ^11^ School of Biotechnology of Southern Medical University Guangzhou China; ^12^ Clinical Laboratory Nanfang Hospital of Southern Medical University Guangzhou China; ^13^ Department of Otolaryngology‐Head and Neck Surgery Zhujiang Hospital Southern Medical University Guangzhou China; ^14^ Department of Pathology Qingyuan People's Hospital Qingyuan China; ^15^ School of Biomolecular and Biomedical Science University College Dublin Dublin Ireland; ^16^ Department of Pathology Nanfang Hospital of Southern Medical University Guangzhou China; ^17^ The Second Affiliated Hospital of Soochow University Suzhou Jiangsu China; ^18^ Suzhou Science& Technology Town Hospital Suzhou Hospital Affiliated to Nanjing Medical University Suzhou Jiangsu China; ^19^ Jiangxi Medical College Shangrao Jiangxi China; ^20^ School of Life Science Beijing Institute of Technology Beijing China; ^21^ Tongji Hospital Affiliated to Tongji Medical College of Huazhong University of Science and Technology Wuhan Hubei China; ^22^ Department of Otolaryngology‐Head and Neck Surgery 303 Hospital of People's Liberation Army of China Nanning China

**Keywords:** immune response, nasopharyngeal carcinoma, single‐nucleotide polymorphisms, targeted MHC sequencing, *TRIM26*

## Abstract

The major histocompatibility complex (MHC) is most closely associated with nasopharyngeal carcinoma (NPC), but the complexity of its genome structure has proven challenging for the discovery of causal MHC loci or genes. We conducted a targeted MHC sequencing in 40 Cantonese NPC patients followed by a two‐stage replication in 1065 NPC cases and 2137 controls of Southern Chinese descendent. Quantitative RT‐PCR analysis (qRT‐PCR) was used to detect gene expression status in 108 NPC and 43 noncancerous nasopharyngeal (NP) samples. Luciferase reporter assay and chromatin immunoprecipitation (ChIP) were used to assess the transcription factor binding site. We discovered that a novel SNP rs117565607_A at *TRIM26* displayed the strongest association (OR = 1.909, Pcombined = 2.750 × 10^−19^). We also observed that *TRIM26* was significantly downregulated in NPC tissue samples with genotype AA/AT than TT. Immunohistochemistry (IHC) test also found the *TRIM26* protein expression in NPC tissue samples with the genotype AA/AT was lower than TT. According to computational prediction, rs117565607 locus was a binding site for the transcription factor Yin Yang 1 (YY1). We observed that the luciferase activity of YY1 which is binding to the A allele of rs117565607 was suppressed. ChIP data showed that YY1 was binding with T not A allele. Significance analysis of microarray suggested that *TRIM26* downregulation was related to low immune response in NPC. We have identified a novel gene *TRIM26* and a novel SNP rs117565607_A associated with NPC risk by regulating transcriptional process and established a new functional link between *TRIM26* downregulation and low immune response in NPC.

## 1 BACKGROUND

Nasopharyngeal carcinoma (NPC) is the most common cancer in South China and Southeast Asia; more than 80% of new NPC cases per year worldwide were reported from these areas,^1^, ^2^ and it has a remarkably distinctive ethnic and geographic distribution, prevalent among Southern Chinese,^3^ suggesting the existence of a genetic predisposition to NPC in these areas or populations in addition to other risk factors including Epstein‐Barr virus (EBV) infection.

The human major histocompatibility complex (MHC) is located on the short arm of chromosome 6. It comprises more than 200 classical and nonclassical MHC genes that have long been believed to be involved in infection, inflammation, autoimmunity, and various cancers.^4^ Since the 1970s, numerous efforts have been made to reveal the association of the MHC with NPC, hypothesizing that some MHC loci or genes may play major roles in the genetic predisposition to NPC, probably either by modulating innate and adaptive immune responses or as genetic markers of predisposition loci in close linkage.^2^, ^5^ This was also strongly supported by two genomewide association studies (GWAS)^6^, ^7^ and our recent association study.^5^ Despite the success of GWAS in identifying genetic variations, the variations in the genome have small effect sizes and only explain a modest fraction of the predicted genetic variance.^8^ And the single‐nucleotide polymorphisms (SNPs) that contribute to the bulk of the heritability tend to be spread across the genome and are not near genes with disease‐specific functions.^9^

The MHC sequencing was first conducted in 1999,^10^ and its extended sequencing was next completed in 2004.^11^ The MHC region is one of the most complex regions in the human genome,^12^, ^13^ owing to its extreme levels of polymorphism, interspersed repeats, and linkage disequilibrium, and it may complicate the genomewide association studies of causal loci or genes contributing to disease phenotypes in this region. It is still unclear, however, whether these findings reflect direct associations with human leukocyte antigen (HLA) genes and/or to other genes in this gene‐rich region.

Target‐enrichment strategy is the method, in which genomic regions of particular interest are selectively captured before sequencing.^14^, ^15^ This approach has proved to be a cost‐effective solution for studying MHC region,^16^ particularly facilitating the population‐based research. Consequently, in this study, we applied this approach to conduct a targeted MHC sequencing in 40 Cantonese NPC patients followed by a two‐stage population‐based replication to identify the causal MHC loci or genes contributing to NPC. Furthermore, we explored the molecular and functional implications of the most significantly associated SNP and its corresponding gene.

## 2 MATERIALS AND METHODS

### 2.1 Study populations and Subjects

All NPC cases used for the targeted sequencing (40 cases), the stage I replication (297 cases and 611 controls), and the stage II replication (768 cases and 1526 controls) were recruited from 4 hospitals (Jiangmen Hospital, Zhongshan Hospital, and Nanfang Hospital of Southern Medical University in Guangdong, China, as well as 303 Hospital of People's Liberation Army of China in Nanning, China) in Guangdong and Guangxi provinces in South China from January 2004 to July 2008 (Table S12). All cases were newly diagnosed and histologically confirmed and had not received any therapy prior to sample collection. Healthy control subjects were recruited from the physical examination centers at the same hospitals. They had neither a family/personal history of cancer nor other major illnesses (such as diabetes, SLE, and rheumatoid arthritis). The control subjects were also frequency‐matched to cases by age, gender, geographic region, and ethnicity. Peripheral blood samples were collected from all participants, and genomic DNA (gDNA) was extracted from peripheral blood mononuclear cells (PBMCs).

Additionally, 108 NPC specimens, 43 noncancerous nasopharyngeal (NP) samples, and their corresponding peripheral blood samples were collected from Zhongshan hospital (June 2009‐December 2012). NPC patients were pathologically diagnosed and had received no therapy prior to biopsy. Noncancerous subjects, suffering from noncancerous disorders such as nasal obstruction and ear blockness, were enrolled from the same hospital and histologically confirmed. Total RNA and gDNA were extracted from both tissue and PBMC samples.

The study was approved by the Human Ethical Committee at Southern Medical University. Written informed consent was obtained from all participants.

### 2.2 Target enrichment, sequencing, and data analysis

Before we get started, we also carefully designed a targeted MHC massive paralleled sequencing approach, using more than 30 000 baits, based on the SureSelect platform (Agilent).^17^ This approach displayed a superior sequencing coverage (96.2%) of the MHC region than that (<30%) of a whole exome sequencing (SureSelect)^18^(Table S13), proving its higher robustness.

A total of 30 816 baits (probes, 120 mer) were generated from 4.42 Mb of MHC (Chr6:29.07‐33.49 Mb, hg18) via Agilent's eArray design and then synthesized by Agilent Technologies. Repeat masker was used, and 2× tiling was selected for probe coverage (Figure S1). Totally, 2.31 Mb of nonrepetitive MHC regions was targeted.

Targeted capture was carried out using SureSelect Reagent Kit (Agilent), according to the manufacturer's protocols. Briefly, gDNA samples were randomly fragmented by Covaris S2 (Covaris Inc.) to approximately 200 bp, and adapters were ligated to both ends of the fragments. The adaptor‐ligated templates were purified using Agencourt AMPure XP beads (Beckman Coulter). Product was amplified by ligation‐mediated PCR, purified, and hybridized to the SureSelect baits for enrichment. After 24 hours, the hybridized fragments were washed, eluted, and further amplified by PCR with 10 cycles. The captured library was sequenced in Illumina GA IIx, according to the manufacturer's protocols.

In read mapping and variant detection, the reference human genome used was UCSC assembly hg18 build (NCBI build 36.3), excluding random placed contigs and alternative haplotypes. The quality‐filtered sequence reads were aligned to the human reference by SOAP2.20^19^ with a maximum of 4 mismatches. The parameters were set as “‐a ‐b ‐D ‐o ‐t ‐r1 ‐n 4 ‐v 2”. The reads in the alignment that have identical start sites were considered as PCR duplicates and then removed.

The targeted sequencing data were compared with the 40 Han Chinese in Beijing, China (CHB), sequences from the 1000 Genomes Project. In fact, the CHB data we analyzed in our paper were from 1000 Genomes Pilot; therefore, only 45 CHB subjects were available at that time. After quality control (excluding CHB samples with very low proportion of overlapping SNPs with our target sequencing data), we only included 40 CHB subjects in our discovery stage. We next used maximum‐likelihood method to discover candidate NPC‐associated SNPs. This method is based on integrating over uncertainty in the data for each individual rather than first calling genotype. The likelihood ratio test (LRT) was utilized in the algorithm to qualify the significance of association between cases and controls.^20^ Fisher's exact test was also performed for each variant based on the minor and major allele count in the aggregate data of cases and controls.

### 2.3 The selection of candidate SNPs for replication

Single‐nucleotide polymorphisms were annotated using an in‐house pipeline and categorized into missense, splice site, untranslated region (UTR) and promoter SNPs that are likely to be more deleterious than synonymous and other noncoding SNPs. Totally, 155 SNPs with potential functions were selected for further replication for showing evidence of association; 81 SNPs with LRT ≥5 were firstly chosen, and additional 74 SNPs with Fisher's exact test (*P*‐value < .001) were then collected (Tables S4 and S5). Quality control cutoffs were set at minor allele frequency (MAF) >1% in the population and call rate >95% in cases or controls. The significant SNPs found at the stage I were further replicated in an additional population of Southern Chinese at the stage II.

### 2.4 Genotyping and association analysis in the two‐stage replication study

The selected SNPs were genotyped on Sequenom MassARRAY system.^21^ Briefly, gDNA samples were diluted to approximately 5‐10 ng/μL and amplified by multiplex PCR. PCR products were applied for locus‐specific single‐base extension reactions. Resulting products were desalted and transferred to a 384‐element SpectroCHIP array for the allele detection on MALDI‐TOF mass spectrometry (Sequenom).

The associations were analyzed by comparing MAF in cases with those of controls using PLINK 1.07 software,^22^ using logistic regressions with an additive model to obtain the *P* values, odds ratios, and 95% confidence interval. Age and gender were included as covariates. All SNPs should pass the quality control (call rate > 95%, Hardy‐Weinberg equilibrium *P* > .001, in the controls). The genetic statistical power was estimated for all genotyped SNPs using CaTS‐Power Calculator software.^23^

### 2.5 SNP genotyping of NPC tissues and patient‐derived PBMCs

High‐resolution DNA melting analysis (HRM)^24^ was performed for SNP genotyping of NPC tissues and patient‐derived PBMCs, according to the manufacturer's instruction. Primer set (forward: GTTGAGTATGAGAGATGTGAGCAG; reverse: ATAAA AGGGCAGAACCATAGCAG) and probe (GACAAATTGTGTATGGGGAAGG GGAG) were designed for rs117565607 (chr6_30280350). Primer asymmetry ratio of 10:1 was used to gain sufficient double‐stranded products for amplicon melting and enough single‐stranded products for probe annealing. PCR was conducted in 96‐well format with 20 μL volumes including 20 ng of gDNA using THUNDERBIRDTM Probe qPCR Mix (TOYOBO QPS‐101) on Mx3005P Stratagene. Melting acquisition was performed on the LightScannerTM (Idaho Technology, Salt Lake City, Utah) modified for high‐resolution melting of LC Green Plus (Idaho). Melting curves were analyzed by the custom software written in LightScanner Call‐IT (Idaho). Normalization and background subtraction were also performed by fitting an exponential to the background surrounding the melting transitions of interest.

### 2.6 Quantitative RT‐PCR analysis (qRT‐PCR)

Total RNA from each sample was reverse‐transcribed to cDNA using a GoScript™ Reverse Transcription System (Promega). Real‐time PCR was performed using Promega GoTaq^®^ qPCR Master Mix (Promega) on Mx3005P Stratagene (Agilent). All data were normalized to ARF5 expression and further normalized to the control sample unless otherwise indicated. Primer sets were listed in Table S14.

### 2.7 Chromatin immunoprecipitation (ChIP)

Chromatin immunoprecipitation was performed using EpiQuik tissue ChIP Kit (Epigentek, Farmingdale, NY) and YY1 antibody (Santa Cruz; Protein A/G PLUS‐Agarose was used), according to the manufacturer's instruction, using NPC tissue samples with different SNP genotypes and genders. After precipitation, primer set for rs117565607 (chr6_30280350) (forward: 5′‐GTTGAGTATGAGAGATGTGAGCAG‐3′, reverse: 5′‐ATAAAAGGGCAGAA CCATAGCAG‐3′) was used for enrichment by conventional PCR.

### 2.8 Luciferase reporter assay

The PCR‐amplified promoter fragment and the synthesized SNP‐T‐ or SNP‐A‐containing fragment (401 bp) of TRIM26 were cloned into luciferase reporter plasmid PGL3‐basic vector using TOPO cloning kit (Invitrogen). The Yin Yang 1 (YY1) gene fragment was also amplified by PCR and cloned into PCDNA3.1 (+) vector. The cotransfections were performed using DharmaFECT Duo Transfection Reagent (Thermo Scientific) in 293T cells, according to the manufacturer's instruction. 293T cells were harvested 48 hours after transfection, followed by the luciferase assays using the Dual‐Luciferase^®^ Reporter Assay System (Promega). Firefly and Renilla luciferase activities were measured as relative light units on luminometer (STRATEC Biomedical Systems, Birkenfeld, Germany). Transfection efficiency was standardized by the cotransfection of Renilla control vector. All assays were independently performed in triplicate.

### 2.9 Immunohistochemistry (IHC)

Immunohistochemistry was conducted using standard techniques as previously described,^25^ using a tripartite motif containing 26 (TRIM26) rabbit anti‐human polyclonal antibody (Novus Biologicals). Semiquantitative assessment of staining was carried out independently by two histopathologists.

### 2.10 Microarray data analysis

Based on our previous gene expression profiling data of 23 NPC and 8 NP tissues (GEO dataset GSE40290), we reordered and selectively rearranged these samples into 3 subgroups (8 high‐TRIM26‐NPs, 7 high‐TRIM26‐NPCs, and 7 low‐TRIM26‐NPCs), according to their TRIM26 expression levels (their mean sample/reference ratios ± SEM were 2.01 ± 0.1058, 2.064 ± 0.0466, and 1.263 ± 0.0654, respectively (*P*‐values < .0001), when comparing high‐TRIM26‐NPCs or high‐TRIM26‐NPs with low‐TRIM26‐NPCs). Significance analysis of microarray (SAM)^26^ was used to analyze gene expression difference by between high‐TRIM26‐NPCs and low‐TRIM26‐NPCs, and high‐TRIM26‐NPs and low‐TRIM26‐NPCs. The delta was set to 0.94 so that false discovery rate (FDR) was 5% for each group comparison. In addition, the gene expression signatures were analyzed by the knowledge‐based annotation software, GenCLiP 2.0.^27^ In GenCLiP 2.0, *P* values were calculated by chi‐square test, and the enrichment of gene functions was further clustered based on related genes, which is similar to DAVID software.^28^ The heat map symbol provided a graphical view of gene‐term relationships. The gene ontology (GO) analysis was also provided by GenCLiP 2.0.

### 2.11 Cell isolation, culture, transfection, and interferon (IFN) treatment

Three NPC cell lines (5‐8F, CNE2, and Sune1) were obtained from the Cancer Research Institute, Southern Medical University, Guangzhou, China, and cultured in RPMI‐1640 (Corning) with 10% calf serum (Gibco) at 37°C in 5% CO_2_.

PBMCs from healthy donors were provided by Guangzhou blood bank center. PBMCs were purified from the buffy coat of heparinized whole‐blood preparations by density centrifugation on low‐endotoxin Ficoll‐Hypaque (Pharmacia Biotech, Piscataway, NJ) and then cultured in RPMI‐1640 (Corning) with 10% calf serum (Gibco) at 37°C in 5% CO_2_.

Natural killer (NK) cells were isolated from PBMCs using human CD56 microbeads MACS kit (Miltenyi Biotec), following the manufacturer's protocol, and cultured in RPMI‐1640 (Corning) with 10% calf serum (Gibco) at 37°C in 5% CO_2_.

SiRNA against TRIM26 (TRIM26‐siRNA) was synthesized by Genepharma (Shanghai, China). Cells (1 × 105) were seeded on 6‐well plates in DMEM (Gibco) with 10% FBS (Gibco) 24 hours prior to transfection and then transfected with siRNA at final concentrations of 0, 20, 50, and 100 nmol/L, respectively, using Lipofectamine TM 2000 (Invitrogen) in serum‐free conditions. Five hours later, the medium was replaced with fresh DMEM with 10% FBS or 10% FBS containing IFN‐α2b.

The cells were treated with IFN‐α2b at final concentrations of 0, 125, 250, and 500 IU/mL, respectively.

### 2.12 Western blotting

Cell lysate was prepared using RIPA buffer with protease inhibitors and then quantified using the BCA protein assay (BioTek, Beijing, China). Protein (20 μg) was loaded onto a 10% SDS‐PAGE gel that was then transferred onto PVDF membrane and incubated with anti‐TRIM26 (Novus), anti‐NFKB2 (Proteintech), and anti‐IRF7 (Proteintech), respectively, at 4°C overnight in a blocker (3% nonfat dry milk/BSA in TTBS), followed by incubation with HRP‐conjugated secondary anti‐rabbit (Proteintech). Protein was normalized with GAPDH (Abmart).

### 2.13 Natural Killer (NK) cell cytotoxicity assay

NK cell cytotoxicity was evaluated by 3‐(4,5‐dimethylthiazol‐2‐yl)‐2,5‐diphenyltetrazolium bromide (MTT) assay using CNE2 as target cells. TRIM26‐siRNA and control siRNA were used to treat these two types of cells.

Briefly, target cells were washed with PBS, resuspended with fresh 1640 culture medium, and seeded into a 96‐well plate at a density of 5000 cells per well. Primary human NK cells were added at an effector‐to‐target (E:T) ratio of 25:1 and incubated at 37°C in a humidified atmosphere of 5% CO_2_. Two hundred μL of the same concentration of NK cells (E) and 200 μL of the same concentration of CNE2 cells (T) were also incubated in different wells as blank and controls, respectively. After 24 hours, 20 μL of MTT solution (5 mg/mL) was added to each well and incubated for 4 hours. The supernatant was removed, and 200 μL of dimethyl sulfoxide (DMSO) was added to each well and agitated for 10 minutes to fully liquefy the crystals. Absorbance was detected at 490 nm (630 nm as a reference wavelength) with an automatic ELISA reader. The resulting data are reported as the average optical density (OD) value. The percentage of NK cell cytotoxicity was calculated as follows: NKA% = (1‐[ODexperimental – ODE]/ODT) × 100%.

### 2.14 Statistical analysis

Statistical analyses were performed by the SPSS 13.0 statistical software package (SPSS Inc., Chicago, IL). Student's *t* test was used to determine the differences between groups for gene expression analyses and in vitro analyses. *P*‐value of <.05 was used as the criterion of statistical significance if not particularly mentioned. All statistical tests were two‐sided.

## 3 RESULTS

### 3.1 Targeted MHC sequencing and the two‐stage replication

**Figure 1:**
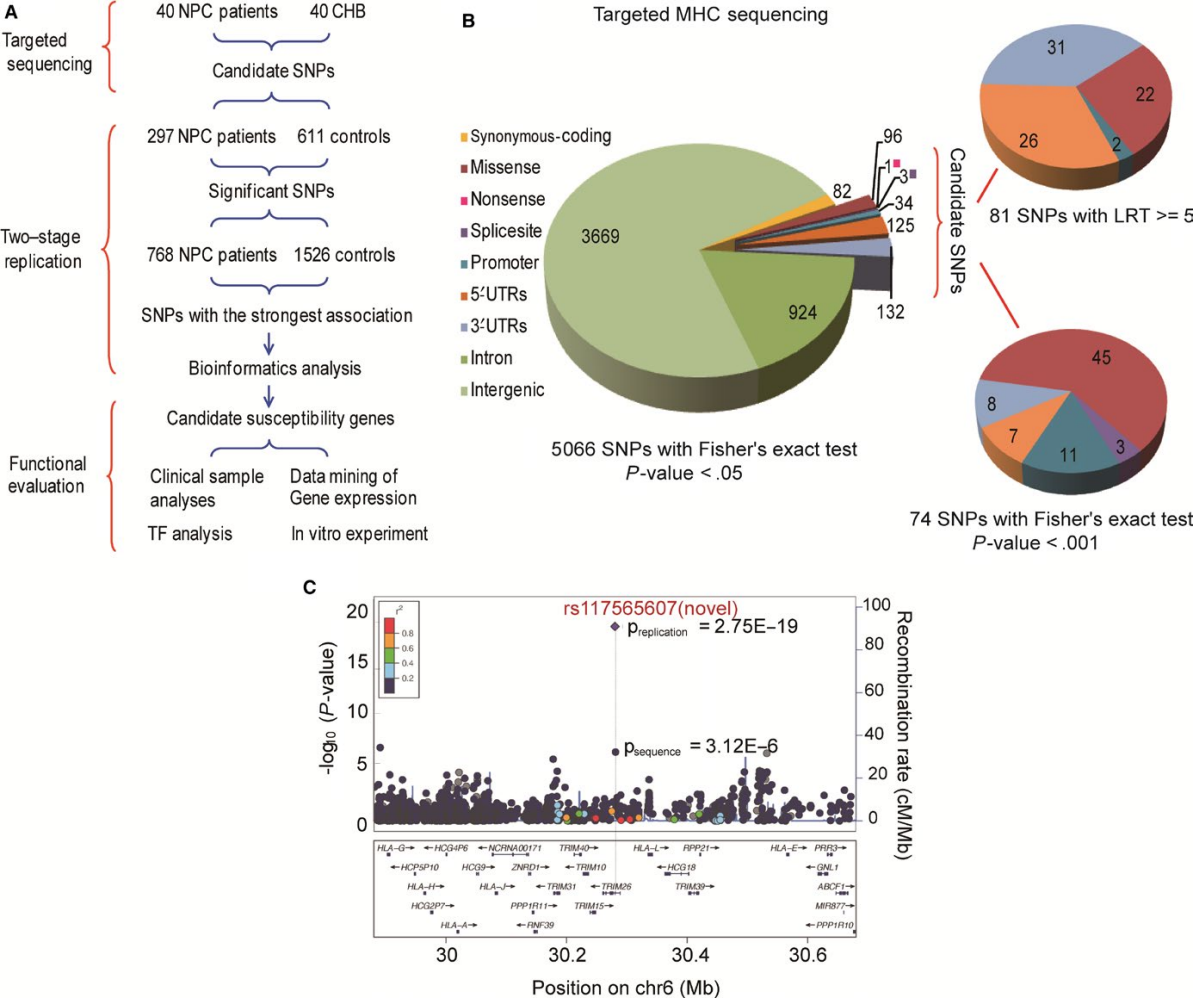
The targeted MHC sequencing and replication study. A, The study design outline. B, As pie charts indicated, 155 SNPs (81 SNPs with likelihood ratio test (LRT) >5 and 74 SNPs with Fisher's exact test *P*‐value < .001) with potential functions and suggestive associations were selected as candidates from 5066 significant SNPs identified by targeted MHC sequencing. C, Top‐ranking SNPs with association evidence were replicated from 155 candidates in the two‐stage replication study. The regional plot of association result was displayed, respectively, for the SNP with the most significant associations. Shown are *P*‐values (−log10 scale) of the association tests for genotyped (diamonds) and sequenced or imputed (circles) SNPs. Genetic recombination rates, based on CHB (Han Chinese in Beijing, China) and JPT (Japanese in Tokyo, Japan) samples from the HapMap Project, are represented by light blue lines, and genes within the regions are depicted by dark blue arrows

The whole MHC region was firstly sequenced using genomic DNA extracted from peripheral blood mononuclear cells (PBMCs) of 40 Cantonese NPC patients (Figure S1). Mapping to the human reference genome, we achieved an average of 92.3 Mb of mappable data per sample with a mean sequencing depth of 40.33‐fold. The average sequence coverage was more than 95%, and 86.3% of coding and noncoding regions were covered at least tenfold (Table S1), subsequently using 40 CHB (Han Chinese in Beijing, China) genome sequences from the 1000 Genomes Project as a control data. In order to exclude population heterogeneity, we had the principal component analysis (PCA) between 40 NPC samples and 40 CHB samples in the MHC region. And the result of PCA indicated that there was no significant difference between them (Figure S2). And we conducted a sequencing‐based association analysis through LRT and the Fisher's exact test and identified 5066 SNPs with significant evidence (*P* < .05, Figure 1A,B, Table S2). Notably, these variants include most of signals previously reported by two GWAS efforts^6^, ^7^ and our previous study (Table S3),^5^ indicating the reliability of our results.

We next focused on missense, splice site, and regulatory SNPs that were more likely to have functional impacts than others. Totally, 155 SNPs including 81 SNPs with LRT ≥5 and 74 SNPs with Fisher's exact test *P* < .001 were chosen as candidates for subsequent replications (Figure 1B, Tables S4 and S5). After quality control, 111 candidate SNPs were successfully genotyped in 297 NPC cases and 611 healthy controls of Southern Chinese descent at the stage I replication (of the 44 excluded SNPs, 9 were removed due to the repetitive sequence involved after extracting their flanking sequences and the other 35 SNPs failed to pass the quality criterion: call rate >95% and Hardy‐Weinberg equilibrium *P* > .001 in the control). For this part, we identified that 9 SNPs were significantly associated with NPC (Table S6) and they were further genotyped in another independent cohort of 768 NPC cases and 1526 healthy controls of Southern Chinese descent at the stage II replication. It was discovered that 5 SNPs displayed consistent association (Table 1). Then, we made a joint analysis of the combined stage I and stage II replication samples; these 5 SNPs showed more significant association, and two SNPs had the strongest association with NPC, surpassing the genomewide significance threshold after Bonferroni's correction (Table 1). They are rs117565607 (novel‐1: chr6_30280350) at the 5′UTR of tripartite motif containing 26 genes (*TRIM26*; OR = 1.91, *P*_combined_ = 2.75 × 10^−19^) and rs1265053 at chromosome 6 open reading frame 15 (C6orf15; OR = 0.60, *P*_combined_ = 8.62 × 10^−20^; Table 1, Figure 1C).

**Table 1:** Association evidence for top‐ranking SNPs in the two‐stage replication study of Southern Chinese population

SNP	Position	Gene	Function	Allele^a^	Stage I^b^		Stage II^c^		Combined^d^		
					OR (L95‐U95)	*P*	OR (L95‐U95)	*P*	MAF (case/ctrl)	OR (L95‐U95)	*P*
rs1265053	31187868	*C6ORF15*	Missense	C/G	0.6812 (0.5599‐0.8292)	1.218E‐04	0.5757 (0.5058‐0.6549)	4.036E‐17	0.4458/0.5717	0.6024 (0.5402‐0.6720)	**8.620E‐20**
rs117565607	30280350	*TRIM26*	utr‐5	A/T	1.9310 (1.4670‐2.5410)	2.018E‐06	1.8870 ((1.6000‐2.2260)	2.650E‐14	0.2009/0.1164	1.9090 (1.6550‐2.2010)	**2.750E‐19**
6:31190425	31190425	*PSORS1C1*	Promoter	G/C	0.4419 (0.2653‐0.7360)	1.300E‐03	0.8222 (0.5423‐1.2470)	3.560E‐02	0.0225/0.0360	0.6812 (0.4923‐0.9427)	1.985E‐02
rs17604492	32286548	*NOTCH42*	Missense	T/C	0.6619 (0.4406‐0.9943)	4.554E‐02	0.7730 (0.6087‐0.9817)	3.430E‐02	0.0623/0.0808	0.7644 (0.6202‐0.9421)	1.155E‐02
rs1610696	29906782	*HLA‐G*	utr‐3	G/C	0.6442 (0.4081‐1.0170)	4.954E‐02	0.6833 (0.4954‐0.9426)	1.966E‐02	0.0362/0.0534	0.6655 (0.5088‐0.8705)	2.777E‐03

SNP, single‐nucleotide polymorphism; ORs, odds ratio.

Genomewide significance was in bold.

a Minor/major allele. ORs were calculated according to the minor allele.

b Stage I, n = 297 cases and 611 controls.

c Stage II, n = 768 cases and 1526 controls.

d Results from the joint analysis of the combined stage I and stage II replication samples. n = 1065 cases and 2137 controls.

PolyPhen shows that the impact of rs1265053 may be benign although it is probably related to the exonic splicing of C6orf15. Both TFSEARCH and FastSNP predict that rs117565607 is probably involved in the transcriptional regulation of *TRIM26*. *TRIM26* is a member of the tripartite motif (TRIM) protein family composed of more than 70 members in human.^29^ In recent years, the TRIM protein family, such as *TRIM3*,* TRIM16*,* TRIM28*, and *TRIM40*, have been reported for their roles in cancers.^30^, ^31^, ^32^, ^33^ However, to date, its role in tumor remains unclear. Therefore, in this study, to investigate into the relationship between *TRIM26* and NPC, further functional studies were carried out.

### 3.2 The downexpression of *TRIM26* in both NPC tissues and patient‐derived PBMCs

**Figure 2:**
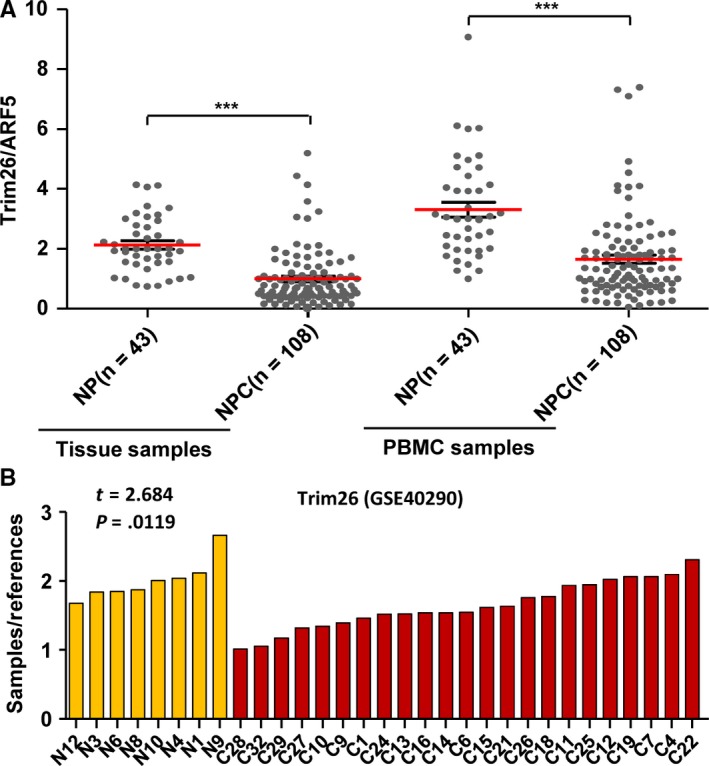
The downexpression of *TRIM26* in both NPC tissues and patient‐derived PBMCs. A, The expression of *TRIM26* was measured by qRT‐PCR in NPC tissue samples, patient‐derived PBMCs, and their control samples (***P < .001). B, *TRIM26* expression in NPC relative to normal controls based on the data mining of gene expression profiling of NPC derived from GEO (GSE40290) (ratio of sample to reference and two‐sided nonparametric *t*‐statistic test were used. *t* = 2.684, *P* = .0119). (N: NP sample; C: NPC sample)

To determine whether *TRIM26* downregulation plays roles in NPC, we firstly test whether or not *TRIM26* was downregulated in NPC. QRT‐PCR was used to detect *TRIM26* expression status in 108 NPC and 43 NP samples. As shown in Figure 2A, *TRIM26* was significantly downexpressed in NPC specimens relative to NP samples, consistent with a set of gene expression data we previously submitted to GEO (GSE40290, Figure 2B). Moreover, we observed a reduced *TRIM26* expression in patient‐derived PBMCs that contain various immune cells (Figure 2A). These data suggested that *TRIM26* was indeed downregulated in NPC, probably being involved in influencing immune response.

### 3.3 The A allele of rs117565607 responsible for *TRIM26* downregulation

**Figure 3:**
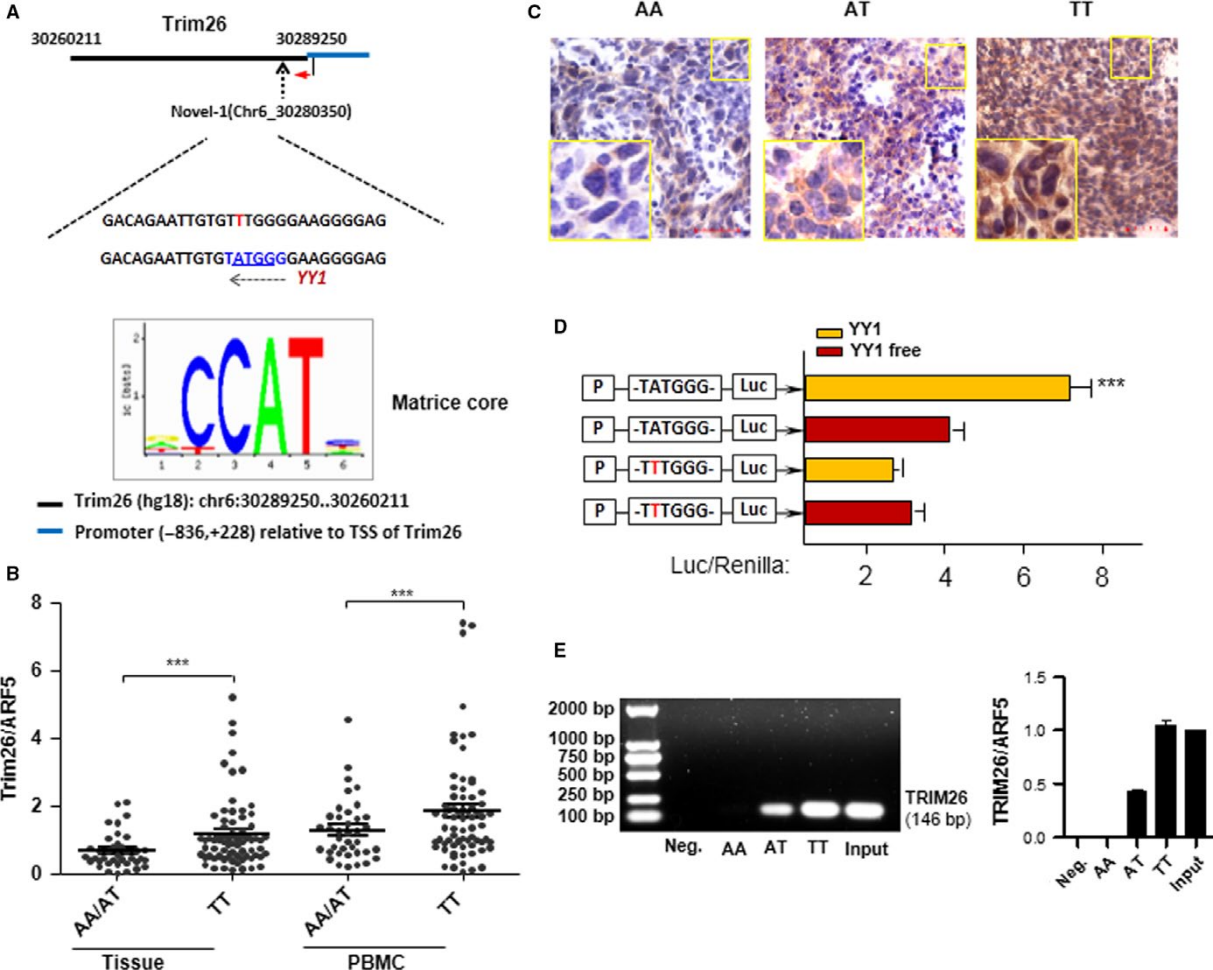
The effect of SNP rs117565607 on *TRIM26* downexpression. (A) As indicated, a transcription factor binding site for YY1 is predicted to be produced at the 5′URT of *TRIM26*. (B) *TRIM26* mRNA expression was assayed by qRT‐PCR in clinical NPC tissues or corresponding PBMC samples with genotype AA/AT or TT of rs117565607 on the sense strand of the sequence. The mean and standard deviation (SD) are marked with black lines. Two‐sided *P*‐values were calculated by Student's *t* test (***P < .001). (C) The *TRIM26* protein expression was evaluated by IHC in representative NPC samples with AA, AT, or TT (left to right) on the sense strand of the sequence. Scale bar = 50 μm. (D) We designed antisense strand with luciferase reporter to introduce exogenous YY1 and observed that the luciferase activity with TTTGGG which is binding to the A allele of rs117565607 was suppressed. (A 401‐bp fragment around rs117565607 in the 5′UTR of *TRIM26* and promoter sequence were inserted into pGL3‐basic vector. Luciferase assay was performed using 293T cells cotransfected with YY1‐vector. Two independent‐sample *t* test, ***P < .001.) (E) ChIP, using NPC tissue samples. DNA was immunoprecipitated with anti‐YY1 or IgG (negative). PCR for genomic fragment (146 bp) around SNP rs117565607 was performed

According to the computational prediction, we noted that rs117565607 locus was a binding site for the transcription factor YY1, which was encoded by *YY1* gene located on the chromosome 14q32.2 (Figure 3A, Figure S4). As a member of the GLI‐Kruppel family of transcriptional factors, YY1 functions as an oncogene in various types of cancers.^34^, ^35^ However, the role of YY1 in nasopharyngeal carcinoma remains unknown.

To determine whether the SNP rs117565607 (A/T) was involved in the transcriptional regulation of *TRIM26* in NPC, we firstly divided 108 clinical NPC samples into two groups according to the SNP genotype of rs117565607 and observed that *TRIM26* was significantly downregulated in NPC tissue samples with genotype AA/AT (genotype based on sense strand of the sequence) than TT (Figure 3B). Similar results appeared in their corresponding PBMCs (Figure 3B), suggesting that rs117565607_A was probably responsible for *TRIM26* downregulation in NPC. The *TRIM26* protein expression was also evaluated by IHC in representative NPC samples and found the *TRIM26* protein expression in NPC tissue samples with the genotype AA/AT was lower than TT (Figure 3C).

In order to validate whether the different alleles (A or T) could influence the expression of *TRIM26*, we next did luciferase reporter assays using 293T cells of human origin. We designed **antisense strand** with luciferase reporter to introduce exogenous *YY1* and observed that the luciferase activity with TTTGGG which is binding to the A allele of rs117565607 was suppressed (Figure 3D). To further illustrate this result, we did ChIP assay using NPC tissue samples, and indeed, *YY1* was binding with T (on the **sense strand** of the sequence) allele not the A allele (Figure 3E). These data indicated that when transcriptional factor *YY1* was bind to the mutant of *TRIM26* (rs117565607_A), the binding affinity of YY1 was reduced and the transcription activation ability was impaired; therefore, those subjects carrying this A allele were expected to have low expression of *TRIM26*.

### 3.4 The relationship of *TRIM26* downregulation with low immune response in NPC

**Figure 4:**
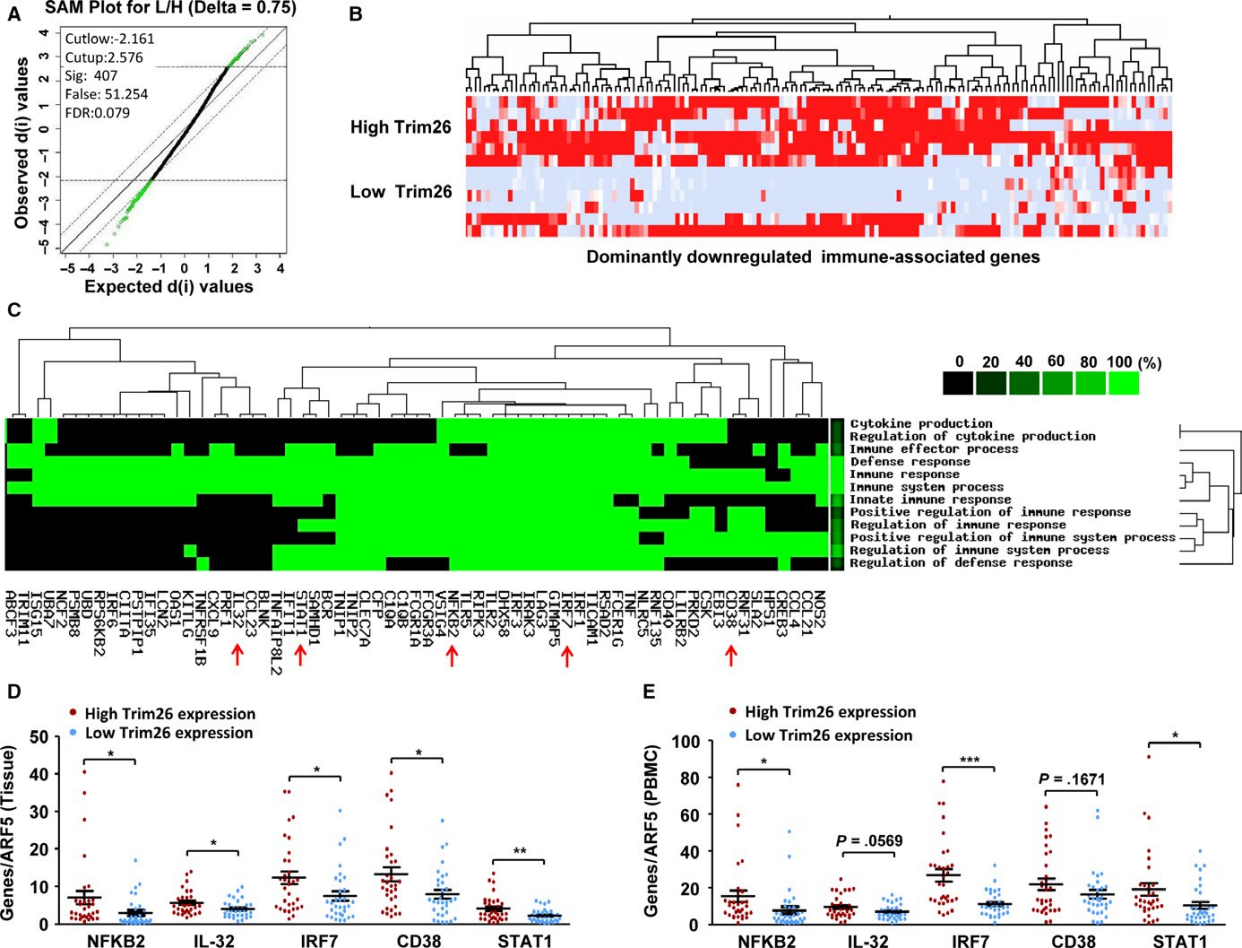
The influence of *TRIM26* downregulation on immune response in NPC. (A) As SAM (significance analysis of microarray) plot displayed, using data mining of gene expression profiling of NPC (GSE40290). (B) The significantly associated GO terms and associated gene heat map. (C) The most significantly associated GO terms (at least *P* < 1e‐05). Green represents the positive relationship between genes and terms. Black represents the unknown relationship. (D) Expression levels of *TRIM26* and some immune genes in NPC tissue samples. E, Expression levels of *TRIM26* and some immune genes in corresponding PBMC samples. *P < .05, **P < .01, ***P < .001

To explore the functional relevance of *TRIM26* to NPC, we next applied the significance analysis of microarray (SAM) to do mining of NPC gene expression data (GSE40290). NPC samples were reclassified according to their relative *TRIM26* expression levels, and gene expression patterns were compared between high‐*TRIM26*‐NPCs and low‐*TRIM26*‐NPCs, and we found 407 significantly differentially expressed genes in Figure 4A, Table S8. Then, we used GenCLiP programs to do data mining analysis of NPC gene expression data (GSE40290). The gene ontology (GO) analysis showed that genes related to the immune response, innate immune response, and antivirus response were significantly overrepresented in these downregulated genes; green represents the positive relationship between genes and terms, and black represents the unknown relationship (Figure 4B,C, Figure S4). These downexpressed genes were majorly involved in the multiple positive regulations (S9). To validate microarray data, we detected the expression of some important immune genes such as *NFKB2*,* IL‐32*,* IRF7*,* CD38*, and *STAT1* in NPC samples and patient‐derived PBMCs with high‐ or low‐*TRIM26* expression level. We observed that most of these immune genes were indeed significantly downregulated upon *TRIM26* downregulation (Figure 4D,E). These data suggested that *TRIM26* downregulation was related to low immune response in NPC.

**Figure 5:**
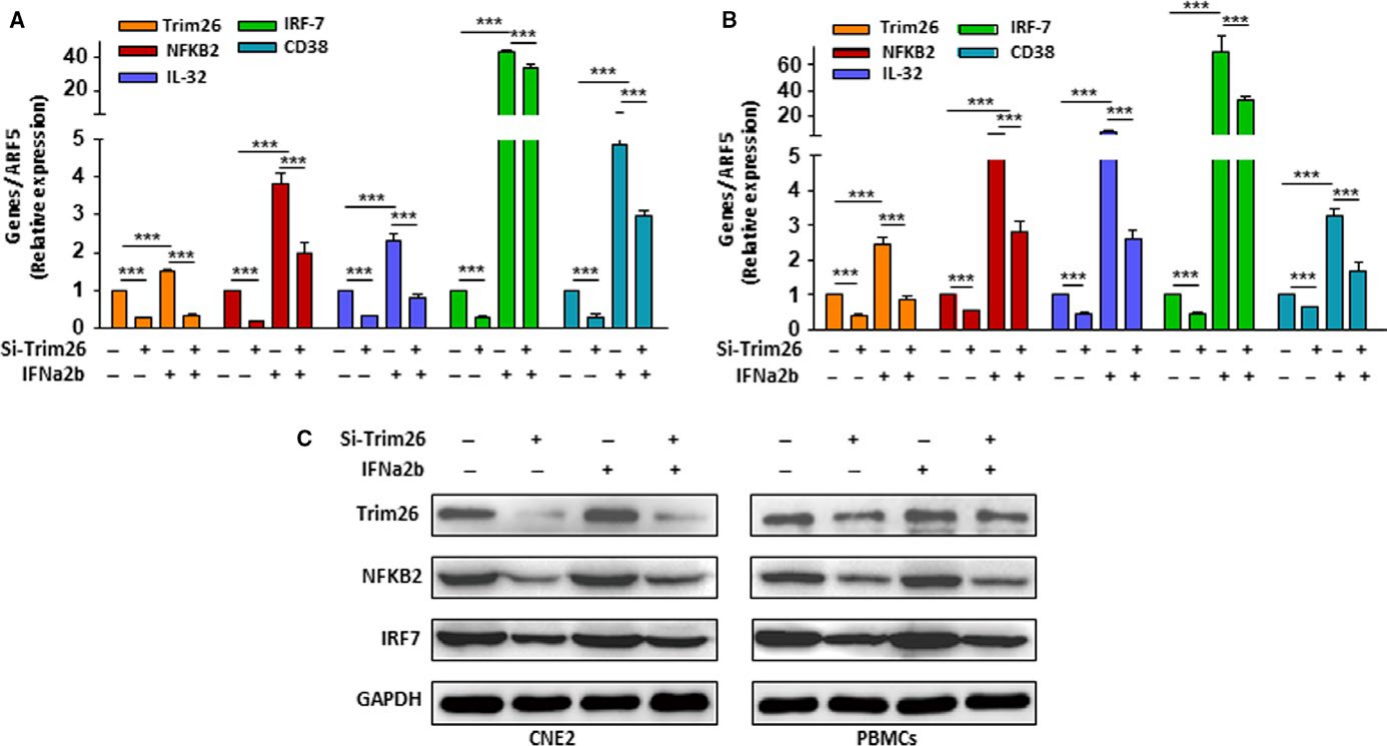
The IFN‐induced immune gene expressions were detected in NPC cell line CNE2 and normal PBMCs that were treated with *TRIM26*‐siRNA. A, NPC cell line CNE2 by RT‐QPCR; B, normal PBMCs by RT‐QPCR. All qRT‐PCR assays were performed in triplicate, and data shown are the mean and SD (*P < .05, **P < .01, ***P < .001). C, The effect of *TRIM26* was confirmed by Western blotting.

Interferons have long been reported to play pleiotropic roles in cancer immunosurveillance and in the broader process of cancer immunoediting.^36^ Many TRIM genes are IFN inducible and involved in IFN‐mediated immune response.^37^, ^38^ To explore whether *TRIM26* downregulation could reduce the effects of IFN‐mediated immune response, we used type I IFN to treat NPC cells and PBMCs in which *TRIM26* was silenced by siRNA. The IFN‐induced immune gene expressions were suppressed in NPC cell line CNE2 (Figure 5A) and normal PBMCs (Figure 5B) when *TRIM26* was silenced. We have also obtained the consistency outcome in Western blotting (Figure 5C). Additionally, we also observed that silencing of *TRIM26* did attenuate immune gene expression in a dose‐dependent manner (Figure S5A,B). Similar results were also observed in normal PBMCs (Figure S5E), suggesting that silencing of *TRIM26* was related to low immune response basally. We found that *TRIM26* could be induced by IFN in a dose‐dependent manner (Figure S5C,D) and similar results were also observed in normal PBMCs (Figure S5F). Collectively, these in vitro data further supported that *TRIM26* downregulation was indeed related to low immune response in NPC.

### 3.5 Downregulation of *TRIM26* and attenuated cytotoxicity of NK

*TRIM26* may influence immune response in both NPC cells and PBMCs, but we did not know whether downregulation of *TRIM26* would weaken immune response of immune or cancer cells. So we used normal NK cell as a representative effector model cocultured with target cells (NPC CNE2 cells) to further verify it. We evaluated the cell‐mediated killing of NK cells cocultured with CNE2 cells with the different treatments of *TRIM26* by siRNA. We initially detected the cytotoxic activity of NK cells lacking *TRIM26* expression by siRNA and observed that NK cell‐mediated killing was relatively attenuated (Figure S6A). Next, we evaluated the cytotoxic activity of normal NK cells cocultured with CNE2 cells lacking *TRIM26* expression. NK cell‐mediated killing was relatively inhibited as well (Figure S6A). Furthermore, we did an in vitro experiment using coculturing CNE2 cells and NK cells, both of which lacked *TRIM26* expression, and a substantial suppression of NK cell‐mediated killing appeared (Figure S6B). These data demonstrated that *TRIM26* downregulation could influence immune response by attenuating either immune cell function or the sensitization of cancer cells to immune defenses.

### 3.6 Downregulation of *TRIM26* and extensive gene expression dysregulations

We also extended our mining effort using GSE40290 data to compare gene expression pattern between high‐ or low‐*TRIM26*‐NPCs with NP samples. We found that 3711 genes were differentially expressed in low‐*TRIM26*‐NPCs (Figure S7B) and only 573 genes in high‐*TRIM26*‐NPCs compared with NP samples (Figure S7A). These genes were involved in multiple biologic processes in addition to immunity (Tables S10 and S11). It is tempting to speculate that lower *TRIM26* expression may be associated with a more extensive dysregulation of gene expressions involved in multiple signaling pathways, thus leading to high NPC susceptibility, although this still remains to be further elucidated.

## 4 DISCUSSION

Although GWAS has successfully identified numerous loci influencing disease risk so far, the mechanisms by which many significantly associated loci, particularly MHC loci, influence diseases are often unclear.^39^ Recently, the identification of genes responsible for diseases has been facilitated with newly developed technologies, particularly the targeted capturing the genome of interest, followed by sequencing. Exome sequencing, emerging as a popular approach, has been widely applied to assess the associations of coding variations, both common and rare, with complex phenotypes.^15^, ^40^, ^41^ The present study first applied a targeted MHC sequencing to human cancer research followed by a two‐stage population‐based replication study, successfully identifying 5 SNPs with consistent associations and eventually pointing to a novel SNP rs117565607 at *TRIM26*, with the strongest association (OR = 1.9090, *P*_combined_ = 2.750 × 10^−19^) and potential function.

Interestingly, rs117565607 is monomorphic in other population except East Asian, and the frequency of A allele is higher in South China than in the north. We acknowledge the fact that the CHB and CHS (Southern Han Chinese) are separated as two subpopulations in Chinese Han in general and the allele frequency for the A allele of rs117565607 is 0.152 in CHS and 0.073 in CHB from 1000 Genomes Project Phase 3 (103 genotypes in CHB and 105 genotypes in CHS) and 0.165 in CHS and 0.062 in CHB from 1000 Genomes Project Phase 1 (97 genotypes in CHB and 100 genotypes in CHS). With respect to the difference between CHS and CHB, we replicated our results in two independent stages with 297 cases/611 controls and 768 cases/1526 controls, and the combined allele frequency in cases and controls was 0.2009/0.1164 with a *P* value reaching the genomewide significance (Table 1).

Furthermore, we calculated the linkage disequilibrium (LD) between the top SNP rs117565607 with the previously reported SNPs^5^, ^6^, ^7^ associated with NPC in MHC region in the 1000 Genomes Project Phase 3 data. As shown in Table S7, very low correlations were identified between rs117560607 with those SNPs, with the largest R^2^ of 0.0339 between rs117565607 and rs3129055 (near *HLA‐F* gene), and SNP rs3129055 was found to have a significant association with NPC in the Taiwanese population.^6^ The lowest correlation was found between rs117565607 and rs28421666 with *R* ^2^ of 0.0002, which was near *HLA‐DQ/DR* gene.^7^ These results suggested that the genetic effect of rs117565607 was independent of the previously reported signals.

Therefore, we decided to do further investigation of this SNP rs117565607. Comprehensive functional and in vitro experiments revealed that this particular SNP was responsible for *TRIM26* downregulation, which was related to abundant immune gene suppressions and extensive gene expression dysregulations, leading to high susceptibility to NPC.

*YY1* is a member of the GLI‐Kruppel family of transcriptional factors, and in recent years, many studies have found that *YY1* was abnormal expression in different human tumor tissues and participated in multiple signaling pathways which were associated with tumorigenesis.^42^, ^43^ Our clinical data and functional analyses supported that genomic DNA fragment containing the susceptible allele (A) of this particular SNP had a lower binding affinity to transcription factor *YY1* and a higher silencing efficacy against *TRIM26* than that of the T allele, thereby downregulating *TRIM26* expression (Figure 3). To our knowledge, rs117565607 has never been reported in NPC before and is not genetically linked to other NPC‐associated variant sites previously reported.

Besides rs117565607, we observed additional 4 SNPs with consistent associations with NPC (Table 1). Rs1265053 at *C6orf15* is less damaging based on the computational prediction. However, the function of *C6orf15* in humans is not clear. For other 3 SNPs, the *P* values are not small enough to provide convincing statistical support for the associations of these 3 SNPs with NPC in the present study. This may be attributed to their relatively low MAFs in study population, so a larger‐scale population is required to strengthen their associations.

*TRIM26* is a member of the TRIM gene family and localizes to the MHC class I region on chromosome 6, and the protein mainly localizes to cytoplasmic bodies.^44^ However, the detailed function of this protein is not well defined, and to date, its role in tumor remains unclear. In the past, Xiong et al^45^ found that the expression of *TRIM26* mRNA in nontumor NP epithelial tissues was significantly higher than that in NPC, suggesting that *TRIM26* gene plays an important role in the development of nasopharyngeal carcinoma, which may be a tumor suppressor gene of nasopharyngeal carcinoma. In 2015, Wang et al^46^ found that *TRIM26* was a novel tumor suppressor gene of hepatocellular carcinoma and its downregulation contributes to worse prognosis. In the present study, we observed that *TRIM26* was significantly downregulated in NPC and further discovered the suppression of abundant immune genes in NPC upon *TRIM26* downregulation, suggesting that *TRIM26* was involved in influencing immune response in NPC.

Nasopharyngeal carcinoma is an Epstein‐Barr virus (EBV)‐associated tumor, and most NPC patients have evidence of infection by this virus.^47^ We used type I IFN to treat NPC cells and PBMCs to support it because IFNs have been best known for initiating an antiviral program of gene expression,^48^ although we did not directly discover the antiviral effects of *TRIM26* in this study. Impressively, our results showed that IFN could induce *TRIM26* expression along with highly expressed immune genes (Figure 5, Figure S6C,D), and in turn, the silencing of *TRIM26* could attenuate IFN‐mediated immune gene expression (Figure 5). These suggest a potential antiviral role of *TRIM26* in NPC. It is worth noting that *TRIM26* was downregulated in NPC cells in addition to PBMCs (Figure 3B). This may be a way in which cancer cells escape immune‐mediated destruction under selection pressure. It is well known that TRIMs have diverse expression patterns and have been found to be expressed in most tissues including cancer tissues, involving multiple processes. For example, *TRIM28* expression in the normal adjacent tissues *TRIM28* mRNA is significantly higher in breast cancer tissues.^49^ And in this study, we found that the *TRIM26* expression was higher with T allele at the SNP rs117565607 than with A allele (Figure 3B,C) and A allele was the risk for NPC in our study. To our knowledge, TRIM members have been rarely reported in cancer immune escape that is important for cancer development and progression, but some other immune genes are now reported to be involving it. For example, silencing of IRF7 pathways can promote immune escape of breast cancer cells, facilitating bone metastasis.^50^ Interestingly, silencing of *TRIM26* was found in our study to be able to attenuate the expression of IFN‐mediated immune genes including *IRF7* and *NFKB2* (Figure 5, Figure S6). This suggests that the *TRIM26* may be involved in cancer immune escape.

Natural killer cells are a main component of PBMCs^51^ and participate in innate immune responses against viruses and tumors.^52^, ^53^, ^54^ In the NK‐mediated killing assays cocultured with NPC cells, we did observe that silencing of *TRIM26* not only directly attenuated NK cell cytotoxicity but also reduced the sensitization of NPC cells to NK‐mediated killing (Figure S7). Particularly, an enhanced attenuation appeared as both target cells and effector cells lacked *TRIM26* expression. This virtually mimics a low immune response in a cancer patient with low *TRIM26* expression and further supports the presence of a potential *TRIM26*‐associated mechanism underlying immune escape of cancer cells.

In addition to low immune response, a more extensive dysregulation of gene expressions was observed in NPC patients with reduced *TRIM26*. Despite no support available from further clinical and experimental data, these results are heuristic for understanding the association of *TRIM26* with NPC susceptibility; low immune response and extensively dysregulated gene expression owing to *TRIM26* downregulation probably confer a high susceptibility to NPC.

Our study was not without its limitations. Even we investigated a new functional link between *TRIM26* downregulation and low immune response in NPC. The mechanisms by which *TRIM26* directly or indirectly regulates immune genes were not fully explored. The direct effect of *TRIM26* on restricting EBV infection was also not investigated. Future studies are being planned. Moreover, the targeted MHC sequencing and replication studies should be planned in a larger study population and different populations.

## 5 CONCLUSIONS

In conclusion, through a targeted MHC sequencing to cancer research and a population‐based replication study as well as functional and molecular investigations, we have identified a novel SNP rs117565607 of *TRIM26* gene and the A allele associated with NPC risk that functions to decrease *TRIM26* expression through YY1‐mediated transcription and the establishment of a new functional link between *TRIM26* downregulation and low immune response in NPC. This may help improve understanding of NPC susceptibility, develop new preventive strategies in NPC endemic areas, and extend the functional spectrum of *TRIM26* on other diseases in addition to NPC.

## CONFLICT OF INTEREST

The authors declare that they have no competing interests.
