# Supplementary material for: A regulatory mutant on TRIM26 conferring the risk of nasopharyngeal carcinoma by inducing low immune response
Source: Cancer Med. 2018 Jun 28;7(8):3848–61. doi: 10.1002/cam4.1537 (PMC6089173; doi:10.1002/cam4.1537)
Supplement: Supplementary file 1 [file CAM4-7-3848-s001.pdf]

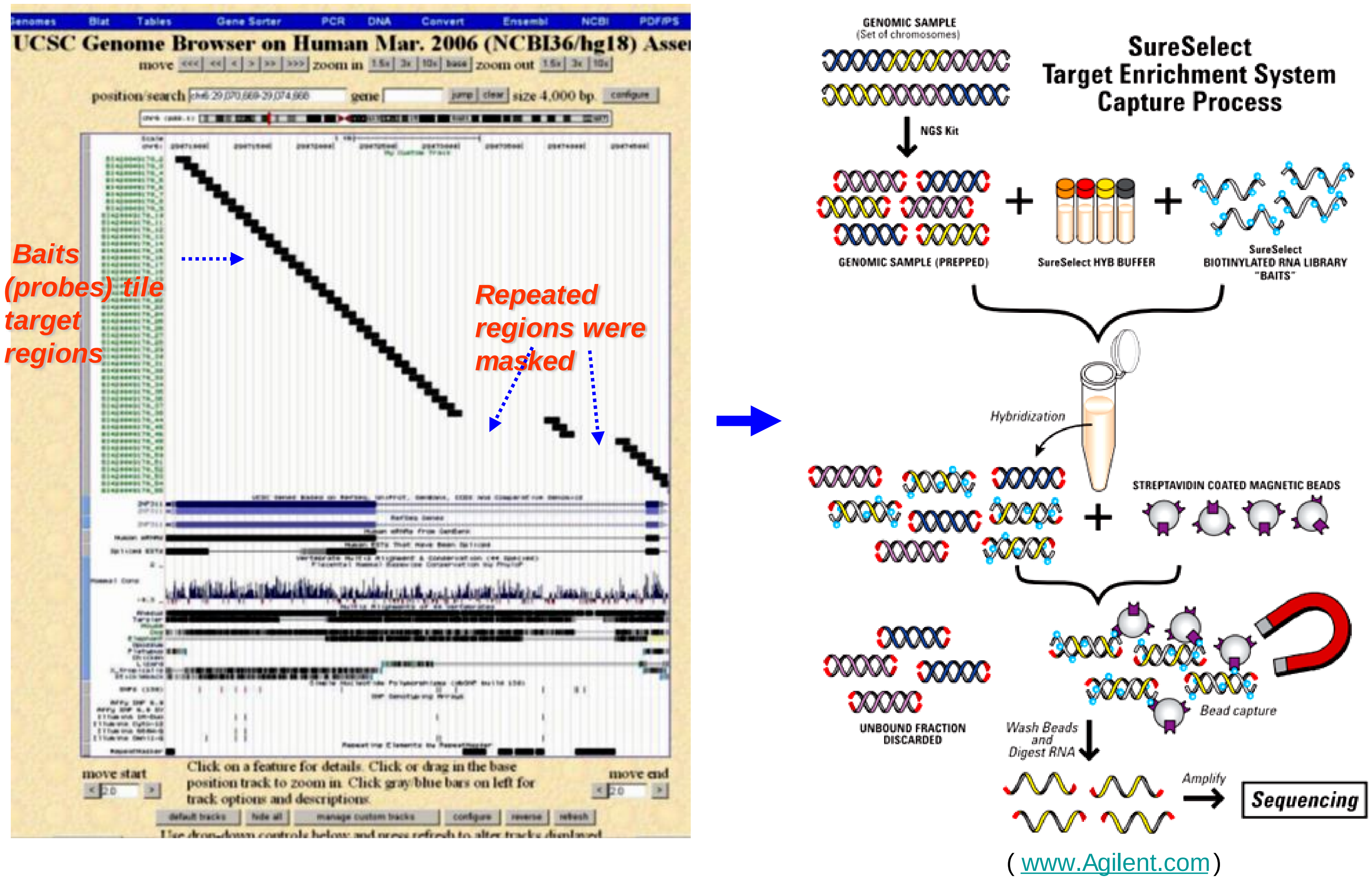

Supplemental Figure 1. A representative target region tiled by baits (probes) and Sureselect capture/sequencing (Agilent SureSelect system).

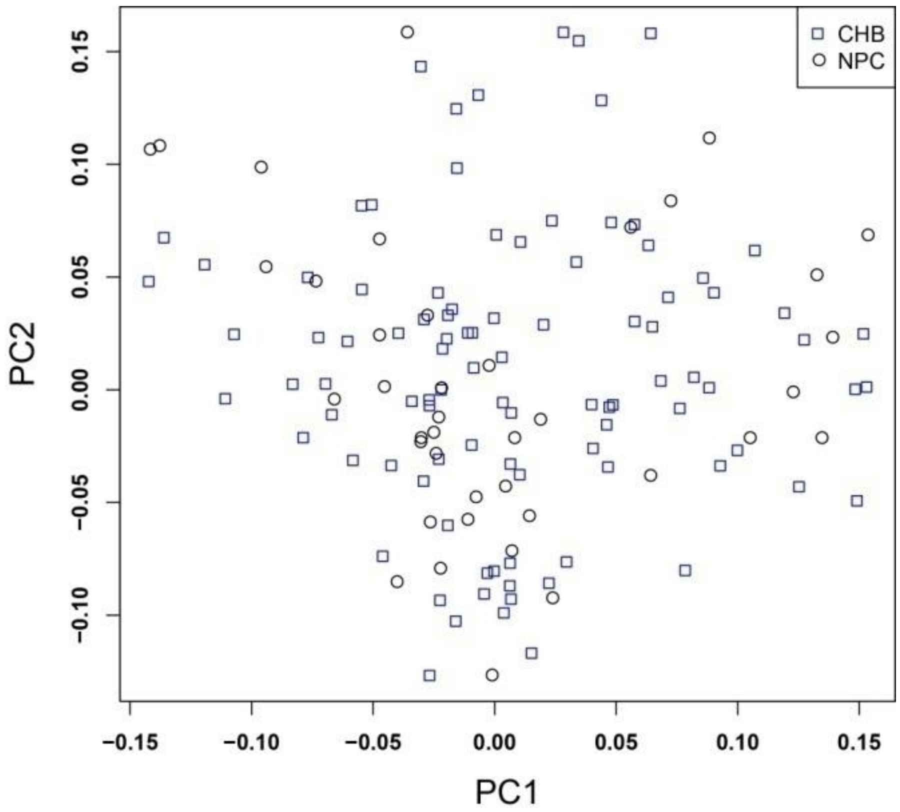

Supplemental Figure 2. Principal component analysis of the population genetic structure between NPC and CHB group in the MHC region.

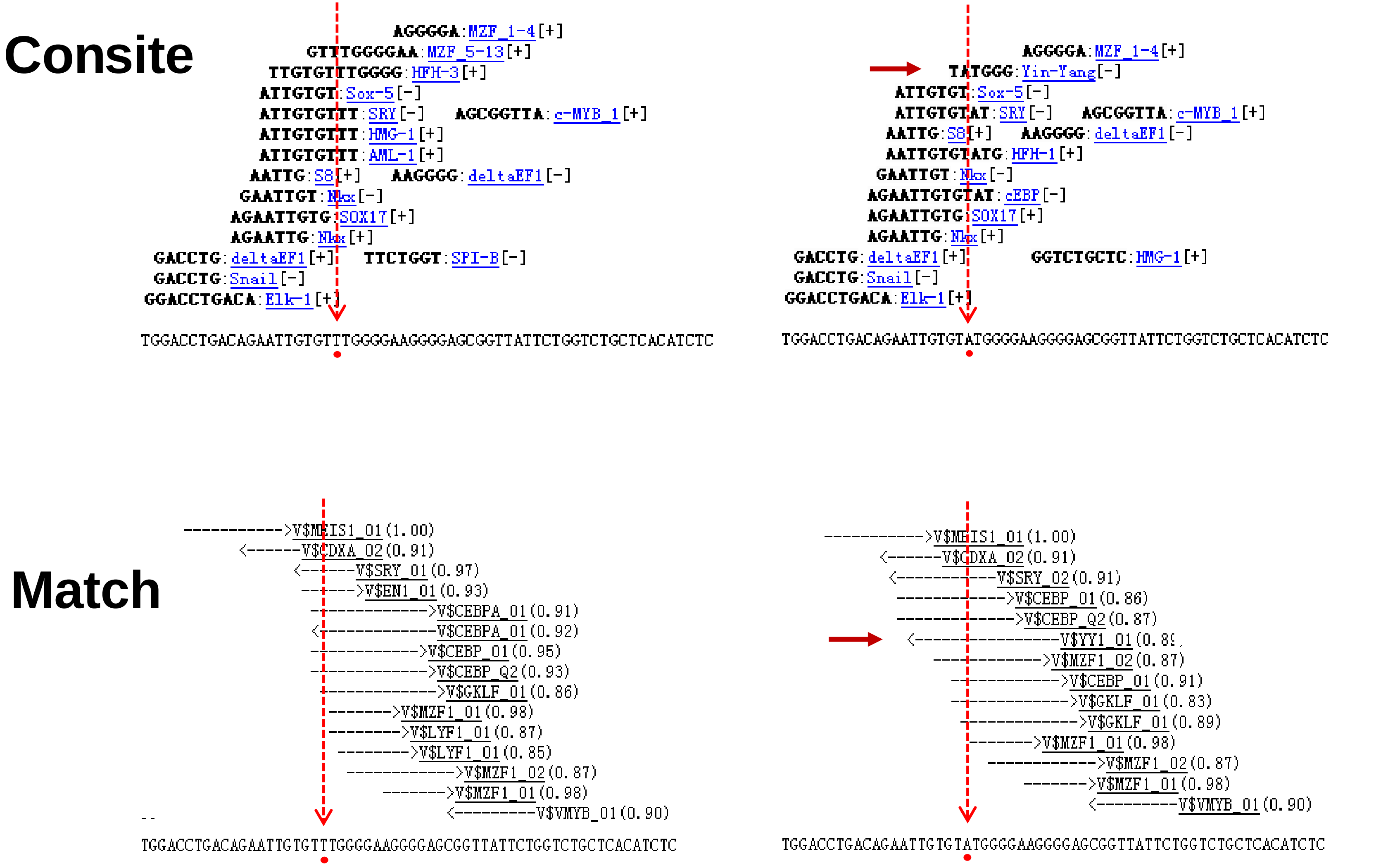

Supplemental Figure 3. Predicted result of transcription Factor Binding Sites for rs117565607 (novel-1:chr6_30280350) by TFSEARCH and FastSNP.

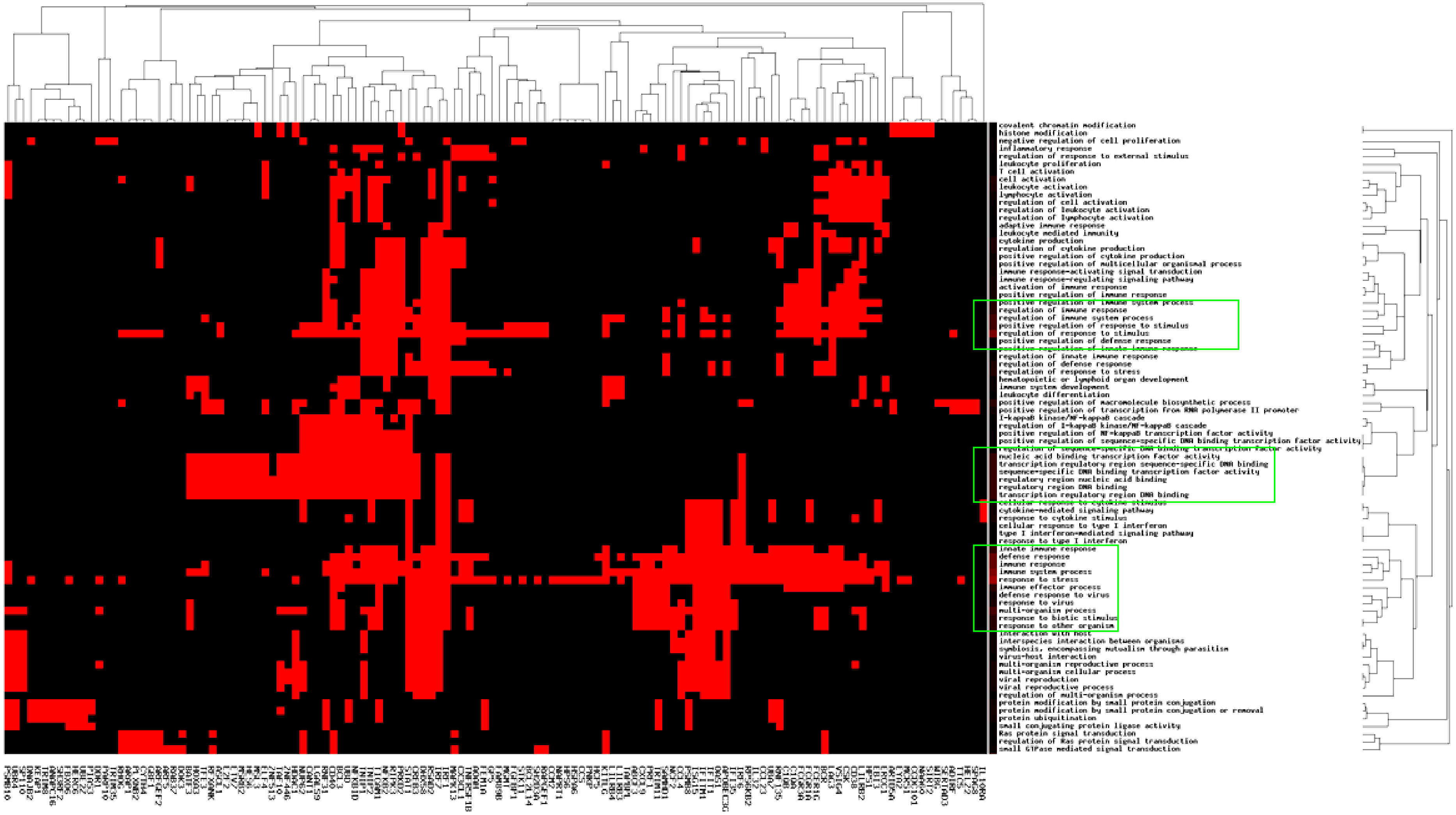

Supplemental Figure 4. The significant associated GO terms and associated gene heat map. The significant associated GO terms and associated genes were displayed in an unfiltered heat map. Three most significant GO term groups (Immune response, regulation of immune response, and DNA-binding) were highlighted by green frames.

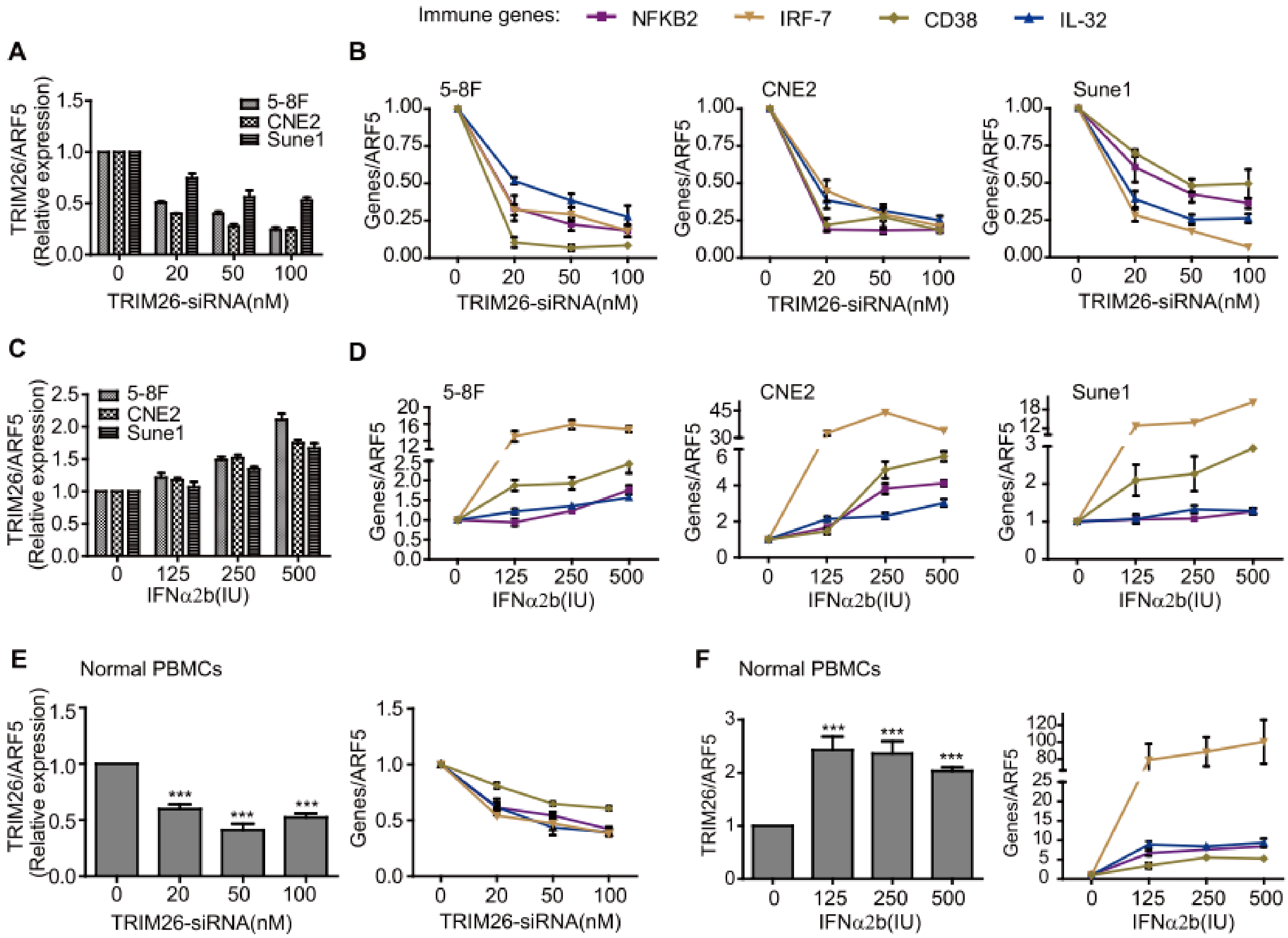

Supplemental Figure 5. In vitro evaluation of the effect of TRIM26 down-regulation on immune response. (A) As indicated, TRIM26 mRNA expression was silenced in NPC cell lines (5-8F, CNE2, Sune1) by TRIM26-siRNA in a dose-dependent manner. (B) The expression of immune genes was next detected in indicated NPC cell lines. (C) Exogenously introduced IFN induced the TRIM2 expression of three NPC cell lines in a dose-dependent manner. (D) The expression of immune genes was next assayed in indicated NPC cell lines. (E) TRIM26 expression was silenced in normal PBMCs by TRIM26-siRNA in a dose-dependent manner (left) followed by the expression detection of immune genes (right). (F) TRIM26 expression was induced by treatment of IFN in a dose-dependent manner (left) and the expression of immune genes was measured (right).

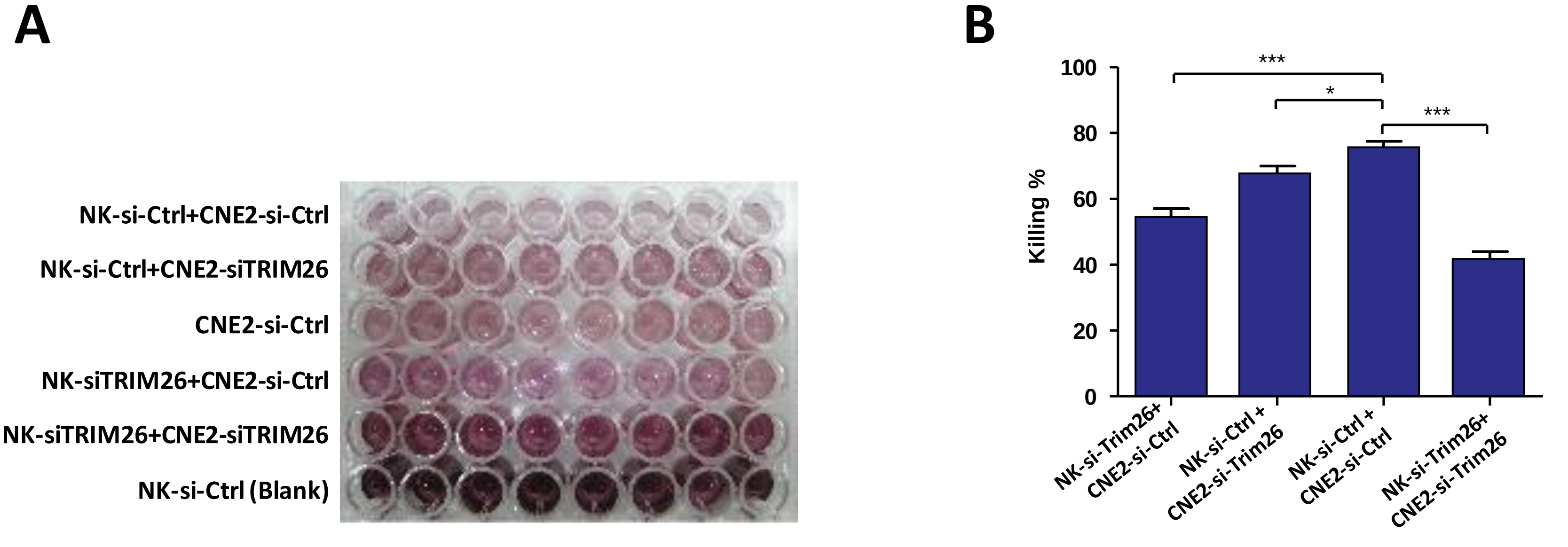

Supplemental Figure 6. NK Cytotoxicity assay. (A) Cytotoxicity induced by NK cells (treated with TRIM26-siRNA or Si-Ctrl) was evaluated by MTT assay. (B) Absorbance was detected at 490 nm (630 nm as reference wavelength) with an automatic ELISA reader. Data represent the mean and SEM from three independent experiments using NK cells from different donors. Two-tailed Student's t-test was used. *P < 0.05, ***P < 0.001.

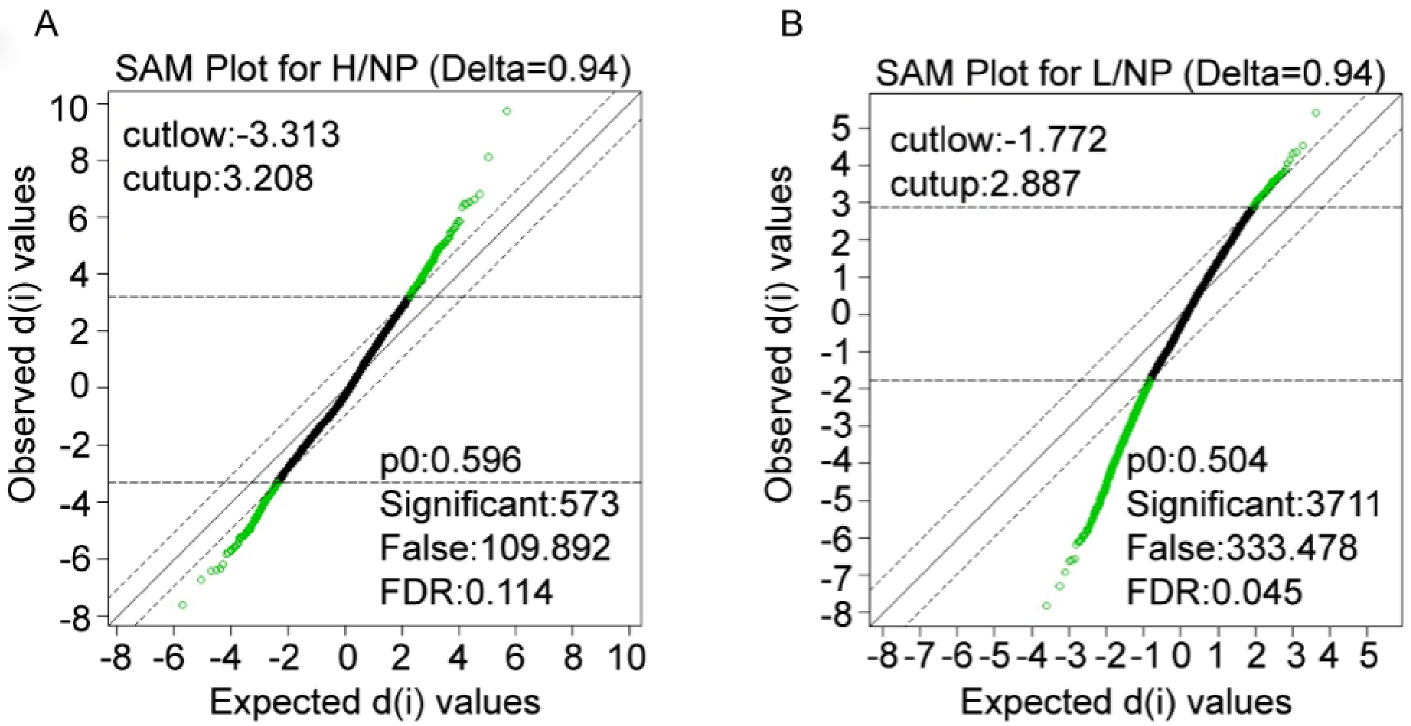

Supplemental Figure 7 (A, B). As SAM plot displayed, using data mining of gene expression profiling of NPC (GSE40290), 573 genes and 3,711 genes (**green**) were differentially expressed in high-TRIM26 NPC (H) (Δ=0.94, FDR=0.114) and low-TRIM26 NPC (L) (Δ=0.94, FDR=0.045) respectively, compared with NP samples (NP).
