# Supplementary material for: A regulatory mutant on TRIM26 conferring the risk of nasopharyngeal carcinoma by inducing low immune response
Source: Cancer Med. 2018 Jun 28;7(8):3848–61. doi: 10.1002/cam4.1537 (PMC6089173; doi:10.1002/cam4.1537)
Supplement: Supplementary file 2 [file CAM4-7-3848-s002.docx]

| **Supplemental Table 1. Data production summary** | |
| --- | --- |
| Data | Targeted sequencing (Mean±SD) |
| Mapped data amount (Mb) | 92.3±44.2 |
| Sequencing depth （fold） | 40.33±12.87 |
| Coverage (%) | 96.2±1.2 |
| Fraction of target covered >=5x | 92.2±2.2 |
| Fraction of target covered >=10x | 86.3±4.1 |
| Fraction of target covered >=15x | 79.3±6.7 |
| Fraction of target covered >=20x | 71.5±9.6 |
| Fraction of target covered >=25x | 63.3±12.4 |
| Fraction of target covered >=30x | 55.1±14.8 |
| Rate of nucleotide difference (%) | 0.39±0.07 |
| Mapped data amount: the sum of read bases that were aligned to the 2.31Mb HLA region. | |
| Coverage: the proportion of target region that was covered by at least one uniquely aligned read. | |
| Rate of nucleotide difference: the percentage of mismatches in unique alignments. | |

| **Supplemental Table 2. Summary of detected variantsa** |  |
| --- | --- |
| SNPs | Target sequencing |
| Synonymous-coding | 82 |
| Missense | 96 |
| Nonsense | 1 |
| Splicesite | 3 |
| Promotor | 34 |
| 5' UTRs **^b^** | 125 |
| 3' UTRs **^b^** | 132 |
| Intron **^c^** | 924 |
| Intergenic | 3,669 |
| Total number | 5,066 |
| a Fisher’s exact test p-value <0.05 |  |
| b SNPs in untranslated regions with 200bp 5' of initiation codon or 3' of termination codon. | |
| C Intronic SNPs within 4bp of exon/ intron boundary. | |

| **Supplemental Table 3. Published loci associated with NPC in MHC region** | | | | | |
| --- | --- | --- | --- | --- | --- |
| SNPs | Paired reads | Significance | Chr. | Population | Ref. |
|  | covered | (*p-value*) |  |  |  |
| rs2517716 | yes | 7.77E-06 | chr6 | Cantonese | 5 |
| rs16896923 | yes | 9.31E-07 | chr6 | Taiwanese | 6 |
| rs2076483 | yes | 1.01E-02 | chr6 | Taiwanese | 6 |
| rs2267633 | yes | 2.42E-02 | chr6 | Taiwanese | 6 |
| rs2517713 | yes | - | chr6 | Taiwanese | 6 |
| rs29232 | yes | 4.95E-01 | chr6 | Taiwanese | 6 |
| rs3129055 | yes | 5.00E-02 | chr6 | Taiwanese | 6 |
| rs9258122 | yes | 1.78E-02 | chr6 | Taiwanese | 6 |
| rs2975042 | not | - | chr6 | Taiwanese | 6 |
| rs3869062 | not | - | chr6 | Taiwanese | 6 |
| rs9260734 | not | - | chr6 | Taiwanese | 6 |
| rs28421666 | yes | - | chr6 | Cantonese | 7 |
| rs2860580 | not | - | chr6 | Cantonese | 7 |
| rs2894207 | not | - | chr6 | Cantonese | 7 |

| **Supplemental Table 4. The candidate SNPs with with LRT >=5 and potential functions for replication study.** | | | | | | | | | | | | | | |
| --- | --- | --- | --- | --- | --- | --- | --- | --- | --- | --- | --- | --- | --- | --- |
| No | chrID | ID | Start_pos | End_pos | Allele | Function | Gene | LRT | *p*_value | Fisher's exact test *p*-value | MAF_control | MAF_case | MAF_total | DAF in genome 1000 |
| 1 | chr6 | snp725 | 32248546 | 32248546 | A/G | utr-5 | AGPAT1 | 12.59319 | 0.0003872 | 2.65E-11 | 0.029576 | 0.2375 | 0.146381 | 0.0083 |
| 2 | chr6 | rs3130059 | 31617263 | 31617263 | C/G | utr-5 | BAT1 | 5.46454 | 0.0194062 | 0.023108688 | 0.599965 | 0.399921 | 0.487819 | 0.5508 |
| 3 | chr6 | rs2239527 | 31617758 | 31617758 | G/C | utr-5 | BAT1 | 9.383875 | 0.002189 | 3.39E-10 | 0.714835 | 0.434588 | 0.546595 | 0.5339 |
| 4 | chr6 | snp688 | 31606141 | 31606141 | T/G | utr-3 | BAT1 | 28.92618 | 7.52E-08 | 9.94E-28 | 0.295922 | 0 | 0.127438 | 0.3644 |
| 5 | chr6 | rs2076523 | 32478813 | 32478813 | T/C | missense | BTNL2 | 5.018303 | 0.0250807 | 0.000463242 | 0.452939 | 0.258585 | 0.344643 | 0.4667 |
| 6 | chr6 | rs28362680 | 32478794 | 32478794 | G/A | missense | BTNL2 | 7.663588 | 0.0056346 | 8.63E-22 | 0.301475 | 0.090058 | 0.180214 | 0.3333 |
| 7 | chr6 | rs1265053 | 31187868 | 31187868 | C/G | missense | C6orf15 | 5.587273 | 0.0180914 | 0.001548047 | 0.349675 | 0.572076 | 0.477729 | 0.5932 |
| 8 | chr6 | rs2233984 | 31187243 | 31187243 | C/T | missense | C6orf15 | 6.234003 | 0.012532 | 0.000186408 | 0.09112 | 0.306461 | 0.220012 | 0.1833 |
| 9 | chr6 | rs17201248 | 31911109 | 31911109 | C/T | utr-5 | C6orf48 | 5.678761 | 0.0171715 | 0.03136709 | 0.048276 | 0.175137 | 0.120437 | 0.0833 |
| 10 | chr6 | rs6921489 | 31912625 | 31912625 | C/T | utr-5 | C6orf48 | 5.824233 | 0.0158069 | 1.62E-07 | 0.218064 | 0.072827 | 0.137171 | 0.2333 |
| 11 | chr6 | rs733539 | 31915468 | 31915468 | C/T | utr-3 | C6orf48 | 7.37285 | 0.0066216 | 3.25E-08 | 0.237346 | 0.066015 | 0.139833 | 0.25 |
| 12 | chr6 | rs707913 | 31192766 | 31192766 | A/G | missense | CDSN | 5.055878 | 0.0245425 | 0.000248838 | 0.220234 | 0.412418 | 0.328326 | 0.3983 |
| 13 | chr6 | rs17180416 | 30052080 | 30052080 | C/A | utr-3 | HCG9 | 9.958154 | 0.0016014 | 1.53E-06 | 0.344753 | 0.124603 | 0.208332 | . |
| 14 | chr6 | rs2975053 | 30021609 | 30021609 | A/C | utr-3 | HLA-A | 12.09042 | 0.0005068 | 1.23E-05 | 0.081595 | 0.361938 | 0.253223 | . |
| 15 | chr6 | rs1056429 | 31429862 | 31429862 | G/A | utr-3 | HLA-B | 5.757328 | 0.01642 | 3.70E-06 | 0.30354 | 0.525 | 0.429861 | 0.2167 |
| 16 | chr6 | rs52797228 | 31430890 | 31430890 | T/C | missense | HLA-B | 7.167471 | 0.0074237 | 8.87E-05 | 0.563625 | 0.362501 | 0.44986 | 0.6017 |
| 17 | chr6 | rs1055348 | 31429927 | 31429927 | T/G | utr-3 | HLA-B | 8.40523 | 0.0037414 | 7.36E-11 | 0.174367 | 0.0375 | 0.097992 | 0.4661 |
| 18 | chr6 | rs3180225 | 31429782 | 31429782 | G/A | utr-3 | HLA-B | 11.81663 | 0.000587 | 4.11E-23 | 0.273037 | 0.071014 | 0.163605 | 0.2373 |
| 19 | chr6 | snp590 | 31430016 | 31430016 | C/A | utr-3 | HLA-B | 27.89332 | 1.28E-07 | 1.33E-25 | 0.260354 | 0 | 0.115053 | 0.1 |
| 20 | chr6 | snp522 | 31345812 | 31345812 | T/C | missense | HLA-C | 6.163381 | 0.0130422 | 2.67E-25 | 0.699187 | 0.365651 | 0.566734 | 0.7583 |
| 21 | chr6 | rs1065711 | 31344832 | 31344832 | A/G | utr-3 | HLA-C | 6.912631 | 0.0085589 | 2.61E-06 | 0.271673 | 0.103045 | 0.17465 | . |
| 22 | chr6 | rs697743 | 31346909 | 31346909 | A/C | missense | HLA-C | 9.5861 | 0.0019606 | 0.001422755 | 0.174144 | 0.511082 | 0.381471 | 0.325 |
| 23 | chr6 | rs41555916 | 31347357 | 31347357 | G/T | missense | HLA-C | 10.31519 | 0.0013194 | 9.28E-08 | 0.60631 | 0.267579 | 0.380985 | 0.6083 |
| 24 | chr6 | rs41543814 | 31347409 | 31347409 | T/C | missense | HLA-C | 11.23516 | 0.0008026 | 1.98E-12 | 0.638734 | 0.290798 | 0.40874 | 0.5917 |
| 25 | chr6 | rs1042688 | 33141033 | 33141033 | A/T | utr-3 | HLA-DPA1 | 5.264551 | 0.021764 | 0.0479297 | 0.458845 | 0.659805 | 0.572077 | 0.7712 |
| 26 | chr6 | rs1062658 | 33143903 | 33143903 | A/T | utr-3 | HLA-DPA1 | 7.100447 | 0.0077065 | 5.46E-24 | 0.48263 | 0.212276 | 0.343168 | 0.7712 |
| 27 | chr6 | snp1149 | 33141265 | 33141265 | G/C | utr-3 | HLA-DPA1 | 19.28106 | 1.13E-05 | 2.96E-43 | 0.444349 | 0.040254 | 0.249991 | 0.7627 |
| 28 | chr6 | rs36013091 | 33145557 | 33145557 | A/T | missense | HLA-DPA1 | 20.9879 | 4.62E-06 | 2.53E-32 | 0.485566 | 0.084692 | 0.271234 | 0.7712 |
| 29 | chr6 | rs1042901 | 33140843 | 33140843 | A/C | utr-3 | HLA-DPA1 | 31.4924 | 2.00E-08 | 6.53E-41 | 0.510317 | 0 | 0.290995 | 0.6949 |
| 30 | chr6 | rs1042920 | 33140809 | 33140809 | G/A | utr-3 | HLA-DPA1 | 33.08144 | 8.84E-09 | 2.36E-51 | 0.56341 | 0 | 0.304738 | 0.7542 |
| 31 | chr6 | rs9277534 | 33162785 | 33162785 | G/A | utr-3 | HLA-DPB1 | 6.583735 | 0.0102915 | 1.78E-06 | 0.464059 | 0.262499 | 0.352536 | 0.7458 |
| 32 | chr6 | rs9277535 | 33162839 | 33162839 | G/A | utr-3 | HLA-DPB1 | 6.999745 | 0.0081521 | 1.67E-05 | 0.496896 | 0.275006 | 0.374159 | 0.55 |
| 33 | chr6 | rs928 | 33162530 | 33162530 | C/G | utr-3 | HLA-DPB1 | 9.581682 | 0.0019653 | 0.003879283 | 0.43284 | 0.711653 | 0.595539 | 0.6949 |
| 34 | chr6 | rs9277532 | 33162689 | 33162689 | G/T | utr-3 | HLA-DPB1 | 14.66415 | 0.0001285 | 0.035594349 | 0.365344 | 0.716185 | 0.56896 | 0.7458 |
| 35 | chr6 | rs9277493 | 33162024 | 33162024 | T/G | utr-3 | HLA-DPB1 | 21.58893 | 3.38E-06 | 5.05E-41 | 0.399225 | 0 | 0.224032 | 0.678 |
| 36 | chr6 | rs929 | 33162597 | 33162597 | G/A | utr-3 | HLA-DPB1 | 24.45676 | 7.60E-07 | 1.18E-37 | 0.478814 | 0 | 0.284802 | 0.7458 |
| 37 | chr6 | rs9272847 | 32718673 | 32718673 | C/T | utr-3 | HLA-DQA1 | 6.483261 | 0.0108895 | 2.39E-14 | 0.421187 | 0.172639 | 0.260723 | 0.4333 |
| 38 | chr6 | snp1068 | 32821022 | 32821022 | C/G | missense | HLA-DQA2 | 18.58347 | 1.63E-05 | 8.46E-36 | 0.190921 | 0 | 0.086094 | 0.1417 |
| 39 | chr6 | rs34240976 | 32742404 | 32742404 | G/A | utr-5 | HLA-DQB1 | 6.602936 | 0.0101811 | 7.15E-07 | 0.157404 | 0.406338 | 0.287455 | 0.2288 |
| 40 | chr6 | snp1048 | 32737742 | 32737742 | C/T | missense | HLA-DQB1 | 6.626287 | 0.0100485 | 3.91E-16 | 0.404676 | 0.180142 | 0.279346 | 0.4917 |
| 41 | chr6 | rs6689 | 32735678 | 32735678 | A/G | utr-3 | HLA-DQB1 | 7.195179 | 0.00731 | 3.59E-12 | 0.131655 | 0.324953 | 0.237115 | 0.2627 |
| 42 | chr6 | rs9273651 | 32737177 | 32737177 | T/C | missense | HLA-DQB1 | 36.69367 | 1.38E-09 | 1.06E-54 | 0.515566 | 0 | 0.278285 | 0.4917 |
| 43 | chr6 | rs1041885 | 32520787 | 32520787 | T/A | utr-3 | HLA-DRA | 5.809804 | 0.0159371 | 1.87E-11 | 0.172114 | 0.05 | 0.10332 | 0.1583 |
| 44 | chr6 | snp1030 | 32665613 | 32665613 | G/C | promotor | HLA-DRB1 | 6.305152 | 0.0120388 | 0.00555043 | 0.16446 | 0.363876 | 0.271962 | . |
| 45 | chr6 | rs34817315 | 32665461 | 32665461 | T/C | missense | HLA-DRB1 | 8.070374 | 0.0044995 | 3.49E-24 | 0.849341 | 0.590722 | 0.702364 | . |
| 46 | chr6 | rs2308765 | 32657518 | 32657518 | C/T | missense | HLA-DRB1 | 8.357017 | 0.003842 | 8.60E-08 | 0.105926 | 0.274999 | 0.200125 | 0.0667 |
| 47 | chr6 | rs1064707 | 32654721 | 32654721 | C/T | utr-3 | HLA-DRB1 | 8.793501 | 0.0030231 | 8.46E-28 | 0.857186 | 0.534897 | 0.668948 | 0.8167 |
| 48 | chr6 | rs33936261 | 32657318 | 32657318 | C/G | missense | HLA-DRB1 | 26.38862 | 2.79E-07 | 2.96E-28 | 0.306044 | 0 | 0.11828 | 0.3917 |
| 49 | chr6 | snp1014 | 32656610 | 32656610 | T/A | missense | HLA-DRB1;HLA-DRB1 | 8.073135 | 0.0044926 | 3.12E-05 | 0.347842 | 0.180364 | 0.26916 | . |
| 50 | chr6 | snp877 | 32595287 | 32595287 | T/C | missense | HLA-DRB5 | 6.01185 | 0.0142101 | 2.69E-13 | 0.837937 | 0.55756 | 0.667772 | . |
| 51 | chr6 | snp923 | 32606045 | 32606045 | C/T | promotor | HLA-DRB5 | 6.886358 | 0.0086856 | 1.37E-16 | 0.118843 | 0.307302 | 0.227253 | 0.8729 |
| 52 | chr6 | rs1610696 | 29906782 | 29906782 | C/G | utr-3 | HLA-G | 11.38806 | 0.0007392 | 2.03E-19 | 0.166315 | 0.012501 | 0.081936 | 0.275 |
| 53 | chr6 | rs9368696 | 31662361 | 31662361 | A/G | utr-5 | LST1 | 6.22171 | 0.0126193 | 3.99E-07 | 0.720898 | 0.512479 | 0.597491 | 0.225 |
| 54 | chr6 | rs1041981 | 31648763 | 31648763 | A/C | missense | LTA | 10.57603 | 0.0011456 | 1.57E-15 | 0.67719 | 0.387347 | 0.511035 | 0.5254 |
| 55 | chr6 | rs3749952 | 31791136 | 31791136 | T/G | missense | LY6G6D | 5.29071 | 0.0214395 | 0.008611552 | 0.077372 | 0.204664 | 0.147796 | 0.161 |
| 56 | chr6 | rs2242653 | 31783744 | 31783744 | G/A | missense | LY6G6F | 5.202918 | 0.022549 | 3.48E-05 | 0.190984 | 0.063313 | 0.120007 | 0.2333 |
| 57 | chr6 | rs2516498 | 31573878 | 31573878 | G/C | utr-5 | MICB | 6.393328 | 0.011455 | 4.05E-11 | 0.23361 | 0.074932 | 0.136666 | 0.2333 |
| 58 | chr6 | snp687 | 31585753 | 31585753 | A/T | utr-3 | MICB | 13.86336 | 0.0001966 | 0.048919439 | 0.059421 | 0.305423 | 0.204229 | . |
| 59 | chr6 | rs986475 | 31664688 | 31664688 | A/G | utr-3 | NCR3 | 5.280146 | 0.0215699 | 1.83E-09 | 0.186707 | 0.062524 | 0.114644 | 0.1917 |
| 60 | chr6 | rs2071592 | 31623319 | 31623319 | A/T | utr-5 | NFKBIL1 | 6.711589 | 0.0095788 | 0.000103795 | 0.60556 | 0.387246 | 0.480656 | 0.5254 |
| 61 | chr6 | rs3095302 | 31201045 | 31201045 | A/G | utr-5 | PSORS1C1 | 5.11581 | 0.0237088 | 2.03E-07 | 0.197632 | 0.387665 | 0.309054 | 0.7119 |
| 62 | chr6 | rs9263717 | 31204505 | 31204505 | A/G | utr-5 | PSORS1C1 | 5.308706 | 0.0212191 | 1.28E-09 | 0.470214 | 0.274901 | 0.35615 | 0.4407 |
| 63 | chr6 | rs3131003 | 31201461 | 31201461 | A/G | utr-5 | PSORS1C1 | 5.713619 | 0.0168338 | 0.000491913 | 0.213822 | 0.412501 | 0.330408 | 0.7119 |
| 64 | chr6 | rs3778639 | 31201755 | 31201755 | A/G | utr-5 | PSORS1C1 | 5.737465 | 0.0166067 | 1.28E-09 | 0.298961 | 0.119227 | 0.191501 | 0.2333 |
| 65 | chr6 | rs17196961 | 31202782 | 31202782 | G/T | utr-5 | PSORS1C1 | 6.94537 | 0.0084036 | 1.16E-12 | 0.282756 | 0.1125 | 0.192582 | 0.2083 |
| 66 | chr6 | rs3778640 | 31202099 | 31202099 | G/C | utr-5 | PSORS1C1 | 7.675822 | 0.0055966 | 5.56E-08 | 0.312676 | 0.11256 | 0.196566 | 0.2333 |
| 67 | chr6 | rs3130997 | 31198837 | 31198837 | G/A | utr-5 | PSORS1C1 | 7.753167 | 0.0053618 | 4.57E-11 | 0.618103 | 0.30182 | 0.417238 | 0.6949 |
| 68 | chr6 | rs6931464 | 31202986 | 31202986 | A/G | utr-5 | PSORS1C1 | 7.970478 | 0.0047546 | 6.86E-13 | 0.353042 | 0.140235 | 0.227827 | 0.3583 |
| 69 | chr6 | rs9263701 | 31202133 | 31202133 | G/T | utr-5 | PSORS1C1 | 8.353143 | 0.0038502 | 1.64E-06 | 0.40802 | 0.175002 | 0.272944 | 0.3583 |
| 70 | chr6 | rs12199460 | 31202707 | 31202707 | A/G | utr-5 | PSORS1C1 | 9.430494 | 0.0021341 | 2.83E-11 | 0.349981 | 0.1125 | 0.206903 | 0.2333 |
| 71 | chr6 | rs3909109 | 31204346 | 31204346 | A/G | utr-5 | PSORS1C1 | 11.27502 | 0.0007856 | 1.40E-13 | 0.445627 | 0.176397 | 0.293395 | 0.3167 |
| 72 | chr6 | rs9263699 | 31201678 | 31201678 | G/A | utr-5 | PSORS1C1 | 11.42117 | 0.0007261 | 9.44E-18 | 0.450613 | 0.175 | 0.29746 | 0.3583 |
| 73 | chr6 | rs9263703 | 31202461 | 31202461 | T/G | utr-5 | PSORS1C1 | 12.8122 | 0.0003444 | 2.54E-11 | 0.462867 | 0.175633 | 0.295696 | 0.3583 |
| 74 | chr6 | rs3815087 | 31201566 | 31201566 | G/A | utr-5 | PSORS1C1 | 12.99858 | 0.0003117 | 8.47E-18 | 0.491249 | 0.175011 | 0.306326 | 0.3583 |
| 75 | chr6 | rs6931633 | 31203030 | 31203030 | C/T | utr-5 | PSORS1C1 | 22.34798 | 2.27E-06 | 5.61E-33 | 0.419474 | 0.046458 | 0.188464 | 0.3583 |
| 76 | chr6 | rs2071544 | 32914099 | 32914099 | T/C | utr-5 | TAP2 | 8.002829 | 0.0046704 | 0.004354215 | 0.343049 | 0.626619 | 0.520173 | 0.45 |
| 77 | chr6 | rs3132528 | 31239548 | 31239548 | T/C | utr-3 | TCF19 | 6.043502 | 0.0139576 | 0.000261815 | 0.468158 | 0.245598 | 0.336454 | 0.8814 |
| 78 | chr6 | rs2269426 | 32184477 | 32184477 | G/A | utr-5 | TNXB | 6.299326 | 0.0120784 | 1.09E-06 | 0.360768 | 0.177108 | 0.25594 | 0.4167 |
| 79 | chr6 | snp418 | 30801014 | 30801014 | T/C | utr-3 | TUBB | 27.33439 | 1.71E-07 | 3.24E-06 | 0.072652 | 0.405005 | 0.240994 | . |
| 80 | chr6 | rs7781 | 30801100 | 30801100 | A/G | utr-3 | TUBB | 33.42218 | 7.42E-09 | 2.59E-06 | 0.155409 | 0.486556 | 0.332773 | 0.5847 |
| 81 | chr6 | snp704 | 31975605 | 31975605 | C/G | utr-3 | ZBTB12 | 6.216645 | 0.0126555 | 8.79E-05 | 0.051969 | 0.200002 | 0.139037 | 0.05 |

| **Supplemental Table 5. The candidate SNPs with with Fisher's exact test *p*-value<0.001 and potential functions for replication study.** | | | | | | | | | | | |
| --- | --- | --- | --- | --- | --- | --- | --- | --- | --- | --- | --- |
| No. | chrID | ID | start_pos | end_pos | status | Allele | Gene | Function | Fisher's exact test *p*-value | MAF_in_samples | DAF in genome 1000 |
| 1 | chr6 | snp3315 | 31615034 | 31615034 | novel | G/A | BAT1 | spliceSite | 2.13E-06 | 0.1125 | . |
| 2 | chr6 | snp3382 | 31712492 | 31712492 | novel | A/C | BAT2 | missense | 7.40E-05 | 0.0875 | . |
| 3 | chr6 | snp3363 | 31697494 | 31697494 | novel | T/C | BAT2 | utr-5 | 6.70E-05 | 0.0125 | 0.025 |
| 4 | chr6 | snp3425 | 31763660 | 31763660 | novel | T/C | BAT5 | spliceSite | 9.37E-05 | 0.1 | 0.025 |
| 5 | chr6 | snp5256 | 33045881 | 33045881 | novel | G/C | BRD2 | utr-5 | 0.000440686 | 0.0625 | . |
| 6 | chr6 | rs28362678 | 32470723 | 32470723 | dbSNP | A/G | BTNL2 | missense | 2.58E-13 | 0.0128 | 0.2 |
| 7 | chr6 | snp3491 | 31851818 | 31851818 | novel | A/G | C6orf27 | missense | 0.000602816 | 0.0641 | . |
| 8 | chr6 | rs130079 | 31220716 | 31220716 | dbSNP | A/C | CCHCR1 | missense | 0.000333625 | 0.0128 | 0.225 |
| 9 | chr6 | rs130065 | 31230479 | 31230479 | dbSNP | A/G | CCHCR1 | missense | 8.94E-07 | 0.0128 | 0.225 |
| 10 | chr6 | snp3671 | 32115843 | 32115843 | novel | A/C | CYP21A2 | missense | 0.000377862 | 0.0125 | . |
| 11 | chr6 | snp3664 | 32113983 | 32113983 | novel | C/T | CYP21A2 | promotor | 1.90E-05 | 0.0125 | 0.0333 |
| 12 | chr6 | rs41554316 | 30020294 | 30020294 | dbSNP | C/A | HLA-A | missense | 0.000711677 | 0.0286 | . |
| 13 | chr6 | rs1059509 | 30019133 | 30019133 | dbSNP | A/C | HLA-A | missense | 1.04E-08 | 0.0952 | 0.4472 |
| 14 | chr6 | rs1136692 | 30018780 | 30018780 | dbSNP | C/A | HLA-A | missense | 8.80E-17 | 0.0333 | . |
| 15 | chr6 | rs1050628 | 31432130 | 31432130 | dbSNP | T/C | HLA-B | missense | 0.000203672 | 0.16 | 0.6441 |
| 16 | chr6 | rs34414019 | 31431982 | 31431982 | dbSNP | G/T | HLA-B | missense | 5.36E-06 | 0.4 | 0.675 |
| 17 | chr6 | snp2935 | 31429636 | 31429636 | novel | A/G | HLA-B | utr-3 | 1.78E-07 | 0.0375 | 0.0917 |
| 18 | chr6 | rs41562313 | 31347036 | 31347036 | dbSNP | T/C | HLA-C | missense | 1.76E-07 | 0.0132 | 0.3305 |
| 19 | chr6 | rs45613631 | 32892654 | 32892654 | dbSNP | T/C | HLA-DOB | missense | 0.000544988 | 0.025 | 0.1525 |
| 20 | chr6 | snp5470 | 33149553 | 33149553 | novel | A/G | HLA-DPA1 | promotor | 0.000188827 | 0.1 | . |
| 21 | chr6 | snp5410 | 33145058 | 33145058 | novel | A/G | HLA-DPA1 | spliceSite | 5.24E-05 | 0.0217 | 0.7458 |
| 22 | chr6 | snp5399 | 33143823 | 33143823 | novel | A/G | HLA-DPA1 | utr-3 | 1.50E-05 | 0.0652 | 0.7712 |
| 23 | chr6 | snp5495 | 33151612 | 33151612 | novel | C/T | HLA-DPB1 | promotor | 4.20E-05 | 0.1 | . |
| 24 | chr6 | snp5494 | 33151545 | 33151545 | novel | C/T | HLA-DPB1 | promotor | 1.68E-05 | 0.1 | . |
| 25 | chr6 | snp5497 | 33151664 | 33151664 | novel | T/C | HLA-DPB1 | promotor | 6.13E-06 | 0.05 | . |
| 26 | chr6 | snp5496 | 33151645 | 33151645 | novel | T/C | HLA-DPB1 | promotor | 1.72E-06 | 0.1 | . |
| 27 | chr6 | rs41552513 | 32717173 | 32717173 | dbSNP | A/G | HLA-DQA1 | missense | 1.37E-05 | 0.0735 | 0.1667 |
| 28 | chr6 | rs1142323 | 32717170 | 32717170 | dbSNP | G/A | HLA-DQA1 | missense | 1.09E-05 | 0.0833 | 0.322 |
| 29 | chr6 | snp4799 | 32718674 | 32718674 | novel | A/G | HLA-DQA1 | utr-3 | 4.28E-06 | 0.0417 | 0.1333 |
| 30 | chr6 | rs9272793 | 32718473 | 32718473 | dbSNP | G/A | HLA-DQA1 | missense | 2.61E-06 | 0.0909 | . |
| 31 | chr6 | rs9260 | 32718439 | 32718439 | dbSNP | G/A | HLA-DQA1 | missense | 8.14E-07 | 0.0446 | . |
| 32 | chr6 | rs1071630 | 32717104 | 32717104 | dbSNP | C/T | HLA-DQA1 | missense | 5.14E-09 | 0.1364 | 0.4915 |
| 33 | chr6 | snp4801 | 32719100 | 32719100 | novel | C/T | HLA-DQA1 | utr-3 | 4.93E-09 | 0.0806 | 0.1583 |
| 34 | chr6 | rs12722051 | 32717125 | 32717125 | dbSNP | T/A | HLA-DQA1 | missense | 1.05E-12 | 0.0882 | 0.1917 |
| 35 | chr6 | rs9272689 | 32717083 | 32717083 | dbSNP | A/G | HLA-DQA1 | missense | 2.31E-14 | 0.0312 | 0.5083 |
| 36 | chr6 | snp4972 | 32822069 | 32822069 | novel | G/A | HLA-DQA2 | missense | 5.98E-06 | 0.0128 | . |
| 37 | chr6 | snp4850 | 32737898 | 32737898 | novel | T/C | HLA-DQB1 | missense | 0.000833078 | 0.0517 | 0.0417 |
| 38 | chr6 | rs12722107 | 32740810 | 32740810 | dbSNP | A/T | HLA-DQB1 | missense | 0.000150112 | 0.1176 | 0.839 |
| 39 | chr6 | snp4833 | 32735863 | 32735863 | novel | T/C | HLA-DQB1 | utr-3 | 1.05E-06 | 0.18 | 0.0424 |
| 40 | chr6 | snp4845 | 32737733 | 32737733 | novel | A/G | HLA-DQB1 | missense | 1.64E-11 | 0.0139 | 0.3833 |
| 41 | chr6 | snp4840 | 32737107 | 32737107 | novel | T/C | HLA-DQB1 | missense | 2.14E-25 | 0.1522 | 0.2288 |
| 42 | chr6 | snp4695 | 32665413 | 32665413 | novel | C/A | HLA-DRB1 | missense | 1.46E-06 | 0.0312 | 0.6167 |
| 43 | chr6 | snp4679 | 32659890 | 32659890 | novel | C/A | HLA-DRB1 | missense | 4.37E-08 | 0.0307 | 0.0833 |
| 44 | chr6 | rs16822820 | 32660037 | 32660037 | dbSNP | T/G | HLA-DRB1 | missense | 7.28E-13 | 0.0513 | 0.6949 |
| 45 | chr6 | rs1136796 | 32595376 | 32595376 | dbSNP | T/C | HLA-DRB5 | missense | 0.000127236 | 0.0789 | 0.6417 |
| 46 | chr6 | snp4365 | 32595368 | 32595368 | novel | G/T | HLA-DRB5 | missense | 9.47E-07 | 0.119 | 0.8644 |
| 47 | chr6 | snp4455 | 32606112 | 32606112 | novel | A/C | HLA-DRB5 | promotor | 1.01E-07 | 0.05 | 0.6667 |
| 48 | chr6 | snp4451 | 32605940 | 32605940 | novel | C/T | HLA-DRB5 | missense | 3.55E-10 | 0.0714 | 0.822 |
| 49 | chr6 | snp4454 | 32606072 | 32606072 | novel | A/G | HLA-DRB5 | promotor | 2.16E-23 | 0.2174 | . |
| 50 | chr6 | rs1736924 | 29800990 | 29800990 | dbSNP | T/T | HLA-F | missense | 1.31E-07 | 0.12 | . |
| 51 | chr6 | snp1982 | 30779185 | 30779185 | novel | C/T | MDC1 | missense | 0.000331343 | 0.0875 | . |
| 52 | chr6 | snp2269 | 31062314 | 31062314 | novel | T/C | MUC21 | missense | 3.46E-05 | 0.0256 | . |
| 53 | chr6 | rs520692 | 32296618 | 32296618 | dbSNP | C/T | NOTCH4 | missense | 0.000508277 | 0.0694 | 0.2542 |
| 54 | chr6 | rs17604492 | 32286548 | 32286548 | dbSNP | T/C | NOTCH4 | missense | 5.15E-06 | 0.025 | 0.1102 |
| 55 | chr6 | rs2071282 | 32296921 | 32296921 | dbSNP | A/G | NOTCH4 | missense | 1.83E-14 | 0.0128 | 0.1 |
| 56 | chr6 | rs2074468 | 29516249 | 29516249 | dbSNP | T/C | OR10C1 | missense | 0.000384283 | 0.075 | 0.339 |
| 57 | chr6 | snp389 | 29503159 | 29503159 | novel | G/A | OR11A1 | missense | 0.000420949 | 0.0125 | 0.1271 |
| 58 | chr6 | snp391 | 29503546 | 29503546 | novel | T/C | OR11A1 | promotor | 0.000241666 | 0.075 | . |
| 59 | chr6 | snp483 | 29535681 | 29535681 | novel | T/C | OR2H1 | utr-5 | 9.39E-06 | 0.0125 | 0.1271 |
| 60 | chr6 | snp173 | 29188365 | 29188365 | novel | C/T | OR2J3 | missense | 5.51E-06 | 0.0125 | 0.0678 |
| 61 | chr6 | snp2463 | 31203921 | 31203921 | novel | A/G | PSORS1C1 | utr-5 | 7.58E-05 | 0.0875 | 0.0667 |
| 62 | chr6 | snp2441 | 31190425 | 31190425 | novel | G/C | PSORS1C1 | promotor | 5.53E-05 | 0.025 | 0.0917 |
| 63 | chr6 | snp2478 | 31214215 | 31214215 | novel | A/G | PSORS1C1 | missense | 2.34E-05 | 0.0125 | 0.1083 |
| 64 | chr6 | snp2453 | 31198170 | 31198170 | novel | C/G | PSORS1C1 | utr-5 | 5.11E-07 | 0.025 | 0.0917 |
| 65 | chr6 | snp1366 | 30146699 | 30146699 | novel | T/C | RNF39 | utr-3 | 2.79E-08 | 0.0125 | 0.0333 |
| 66 | chr6 | rs3131787 | 31007503 | 31007503 | dbSNP | C/T | SFTA2 | missense | 1.40E-06 | 0.0125 | 0.2167 |
| 67 | chr6 | rs438999 | 32036285 | 32036285 | dbSNP | G/A | SKIV2L | missense | 1.31E-05 | 0.075 | 0.1441 |
| 68 | chr6 | snp5089 | 32897933 | 32897933 | novel | T/C | TAP2 | utr-3 | 1.53E-06 | 0.0125 | 0.2542 |
| 69 | chr6 | snp1539 | 30280350 | 30280350 | novel | A/T | TRIM26 | utr-5 | 3.12E-06 | 0.1625 | 0.0583 |
| 70 | chr6 | snp1541 | 30283297 | 30283297 | novel | T/C | TRIM26 | utr-5 | 1.71E-07 | 0.025 | 0.2712 |
| 71 | chr6 | rs1116221 | 30179309 | 30179309 | dbSNP | T/C | TRIM31 | missense | 1.08E-18 | 0.025 | 0.4576 |
| 72 | chr6 | rs2249464 | 30996140 | 30996140 | dbSNP | T/C | VARS2 | missense | 6.93E-11 | 0.025 | 0.8167 |
| 73 | chr6 | snp5709 | 33326253 | 33326253 | novel | C/G | VPS52 | utr-3 | 0.000112033 | 0.075 | . |
| 74 | chr6 | snp827 | 29757028 | 29757028 | novel | C/A | ZFP57 | promotor | 0.000311217 | 0.0125 | 0.2034 |

| **Supplemental Table 6. Association evidence for 9 SNPs in the stage1 replication of Cantonese population** | | | | | | | |
| --- | --- | --- | --- | --- | --- | --- | --- |
| SNVs | Position | Allelea | MAF | OR(L95-U95) ^b^ | *P-value* | Gene | Function |
|  |  |  | (case/ctrl) |  |  |  |  |
| novel-1 | 30280350 | A/T | 0.1818/0.1032 | 1.9310 (1.4670-2.5410) | 2.02E-06 | *TRIM26* | utr-5 |
| rs28362678 | 32470723 | A/G | 0.1193/0.0645 | 1.9640(1.4070--2.7410) | 5.65E-05 | *BTNL2* | Missense (codonChange='CCG=>CTG') |
| novel-2 | 31190425 | G/C | 0.0313/0.0680 | 0.4419 (0.2653-0.7360) | 1.30E-03 | *PSORS1C1* | promotor |
| rs1265053 | 31187868 | C/G | 0.4559/0.5515 | 0.6812 (0.5599-0.8292) | 1.22E-04 | *C6orf15* | Missense (codonChange='GCA=>CCA') |
| rs2249464 | 30996140 | T/C | 0.0795/0.1327 | 0.5643 (0.4026-0.7909) | 7.87E-04 | *VARS2* | Missense (codonChange='TGG=>CGG') |
| rs1071630 | 32717104 | C/T | 0.4519/0.5289 | 0.7342 (0.5383-0.8638) | 2.31E-03 | *HLA-DQA1* | Missense (codonChange='TTT=>TCT') |
| rs6689 | 32735678 | G/A | 0.3177/0.2419 | 1.4600 (1.1760-1.8110) | 5.79E-04 | *HLA-DQB1* | utr-3 |
| rs17604492 | 32286548 | T/C | 0.0538/0.0790 | 0.6619 (0.4406-0.9943) | 4.55E-02 | *NOTCH4* | Missense (codonChange='GGG=>AGG') |
| rs1610696 | 29906782 | G/C | 0.0428/0.0649 | 0.6442 (0.4081-1.0170) | 4.95E-02 | *HLA-G* | utr-3 |
| a Minor / major allele. b ORs were calculated according to the minor allele. | | | | |  |  |  |

| **Supplemental Table 7. According to 1000 Genomes Project Phase3 data， linkage disequilibrium (LD) between rs117560607 and other previously reported SNPs associated with NPC of MHC region.** | | | | | | |  |
| --- | --- | --- | --- | --- | --- | --- | --- |
| CHR_A | BP_A | SNP_A | CHR_B | BP_B | SNP_B | R^2^ | |
| 6 | 30172371 | rs117565607 | 6 | 29570841 | rs2267633 | 0.000324 | |
| 6 | 30172371 | rs117565607 | 6 | 29571545 | rs2076483 | 0.000324 | |
| 6 | 30172371 | rs117565607 | 6 | 29611431 | rs29232 | 0.010094 | |
| 6 | 30172371 | rs117565607 | 6 | 29670261 | rs3129055 | 0.033915 | |
| 6 | 30172371 | rs117565607 | 6 | 29906691 | rs2860580 | 0.012292 | |
| 6 | 30172371 | rs117565607 | 6 | 29917260 | rs2517716 | 0.012796 | |
| 6 | 30172371 | rs117565607 | 6 | 29918099 | rs2517713 | 0.00545 | |
| 6 | 30172371 | rs117565607 | 6 | 29920536 | rs2975042 | 0.005346 | |
| 6 | 30172371 | rs117565607 | 6 | 29932666 | rs9260734 | 0.003277 | |
| 6 | 30172371 | rs117565607 | 6 | 29934891 | rs3869062 | 0.003269 | |
| 6 | 30172371 | rs117565607 | 6 | 30000687 | rs16896923 | 0.002022 | |
| 6 | 30172371 | rs117565607 | 6 | 30172371 | rs117565607 | 1 | |
| 6 | 30172371 | rs117565607 | 6 | 31079889 | rs1265053 | 0.007194 | |
| 6 | 30172371 | rs117565607 | 6 | 31263751 | rs2894207 | 0.008266 | |
| 6 | 30172371 | rs117565607 | 6 | 32592737 | rs28421666 | 0.000223 | |

| **Supplemental Table 8. Differenttially expressed genes between low-Trim26-NPC and high-Trim26-NPC samples.** | | | | | | | | |
| --- | --- | --- | --- | --- | --- | --- | --- | --- |
| Row | d.value | stdev | rawp | q.value | R.fold | ENTREZID | SYMBOL | GENENAME |
| 1156 | -4.863748 | 0.3696078 | 6.5E-06 | 0.0635482 | 0.16342 | 11326 | VSIG4 | V-set and immunoglobulin domain containing 4 |
| 4777 | -4.398748 | 0.2349406 | 1.4E-05 | 0.0674815 | 0.29298 | 27128 | CYTH4 | cytohesin 4 |
| 12116 | -4.097886 | 0.2029646 | 2.9E-05 | 0.0674815 | 0.348939 | 79132 | DHX58 | DEXH (Asp-Glu-X-His) box polypeptide 58 |
| 14775 | -3.931843 | 0.4187329 | 4.2E-05 | 0.0674815 | 0.202252 | 9235 | IL32 | interleukin 32 |
| 4521 | -3.841709 | 0.6348275 | 5.2E-05 | 0.0674815 | 0.118004 | 26228 | STAP1 | signal transducing adaptor family member 1 |
| 5480 | -3.787649 | 0.7505565 | 5.9E-05 | 0.0674815 | 0.089744 | 3002 | GZMB | granzyme B (granzyme 2, cytotoxic T-lymphocyte-associated serine esterase 1) |
| 4534 | -3.748098 | 0.3725874 | 6.4E-05 | 0.0674815 | 0.245695 | 26240 | FAM50B | family with sequence similarity 50, member B |
| 11944 | -3.742837 | 0.1131992 | 6.5E-05 | 0.0674815 | 0.482509 | 7726 | TRIM26 | tripartite motif containing 26 |
| 1629 | -3.711504 | 0.6294764 | 7.1E-05 | 0.0674815 | 0.128627 | 126859 | AXDND1 | axonemal dynein light chain domain containing 1 |
| 13469 | -3.503883 | 0.5976388 | 0.00012 | 0.0719689 | 0.155861 | 84419 | C15orf48 | chromosome 15 open reading frame 48 |
| 8075 | -3.414916 | 0.6040019 | 0.00015 | 0.0719689 | 0.160951 | 53836 | GPR87 | G protein-coupled receptor 87 |
| 3177 | -3.395736 | 0.3545966 | 0.00016 | 0.0719689 | 0.29248 | 2209 | FCGR1A | Fc fragment of IgG, high affinity Ia, receptor (CD64) |
| 9561 | -3.385821 | 0.4660158 | 0.00016 | 0.0719689 | 0.225992 | 5699 | PSMB10 | proteasome (prosome, macropain) subunit, beta type, 10 |
| 7108 | -3.378195 | 0.7821446 | 0.00016 | 0.0719689 | 0.108159 | 4915 | NTRK2 | neurotrophic tyrosine kinase, receptor, type 2 |
| 6297 | -3.349847 | 0.4135254 | 0.00018 | 0.0719689 | 0.25935 | 3902 | LAG3 | lymphocyte-activation gene 3 |
| 5782 | -3.275945 | 0.3650346 | 0.00021 | 0.0719689 | 0.298289 | 338440 | ANO9 | anoctamin 9 |
| 5581 | -3.275814 | 0.4682819 | 0.00021 | 0.0719689 | 0.235963 | 3108 | HLA-DMA | major histocompatibility complex, class II, DM alpha |
| 4552 | -3.270242 | 0.2421985 | 0.00022 | 0.0719689 | 0.39489 | 26270 | FBXO6 | F-box protein 6 |
| 1108 | -3.241476 | 0.2137077 | 0.00023 | 0.0719689 | 0.42445 | 11284 | PNKP | polynucleotide kinase 3'-phosphatase |
| 2135 | -3.234504 | 0.614332 | 0.00024 | 0.0719689 | 0.173199 | 1475 | CSTA | cystatin A (stefin A) |
| 7502 | -3.189372 | 0.2925667 | 0.00026 | 0.0719689 | 0.36149 | 51224 | TCEB3B | transcription elongation factor B polypeptide 3B (elongin A2) |
| 5699 | -3.136517 | 0.2494835 | 0.0003 | 0.0719689 | 0.403737 | 326624 | RAB37 | RAB37, member RAS oncogene family |
| 3173 | -3.110349 | 0.5519792 | 0.00033 | 0.0719689 | 0.211912 | 2207 | FCER1G | Fc fragment of IgE, high affinity I, receptor for; gamma polypeptide |
| 8615 | -3.10028 | 0.3535249 | 0.00034 | 0.0719689 | 0.326249 | 55080 | TAPBPL | TAP binding protein-like |
| 13320 | -3.051399 | 0.4854643 | 0.00038 | 0.0719689 | 0.251203 | 84174 | SLA2 | Src-like-adaptor 2 |
| 14857 | -3.040937 | 0.1770215 | 0.00039 | 0.0719689 | 0.483542 | 92922 | CCDC102A | coiled-coil domain containing 102A |
| 4520 | -3.034897 | 0.1730018 | 0.0004 | 0.0719689 | 0.488353 | 26226 | FBXW4P1 | F-box and WD repeat domain containing 4 pseudogene 1 |
| 6015 | -3.014243 | 0.4483861 | 0.00043 | 0.0719689 | 0.276042 | 3659 | IRF1 | interferon regulatory factor 1 |
| 5952 | -3.009745 | 0.3047099 | 0.00043 | 0.0719689 | 0.373235 | 3587 | IL10RA | interleukin 10 receptor, alpha |
| 1244 | -3.009458 | 0.1812754 | 0.00043 | 0.0719689 | 0.482889 | 114785 | MBD6 | methyl-CpG binding domain protein 6 |
| 733 | -3.005771 | 0.6602242 | 0.00044 | 0.0719689 | 0.178184 | 10866 | HCP5 | HLA complex P5 (non-protein coding) |
| 10715 | -3.00279 | 0.2173124 | 0.00044 | 0.0719689 | 0.448718 | 64114 | TMBIM1 | transmembrane BAX inhibitor motif containing 1 |
| 9610 | -3.000839 | 0.3784744 | 0.00044 | 0.0719689 | 0.321081 | 57091 | CASS4 | Cas scaffolding protein family member 4 |
| 10600 | -2.994146 | 0.4037219 | 0.00045 | 0.0719689 | 0.305463 | 6376 | CX3CL1 | chemokine (C-X3-C motif) ligand 1 |
| 11586 | -2.993863 | 0.611187 | 0.00045 | 0.0719689 | 0.198624 | 713 | C1QB | complement component 1, q subcomponent, B chain |
| 5582 | -2.992499 | 0.4429535 | 0.00045 | 0.0719689 | 0.281774 | 3109 | HLA-DMB | major histocompatibility complex, class II, DM beta |
| 6672 | -2.985883 | 0.7464799 | 0.00046 | 0.0719689 | 0.150763 | 4283 | CXCL9 | chemokine (C-X-C motif) ligand 9 |
| 9462 | -2.983146 | 0.1018208 | 0.00046 | 0.0719689 | 0.572747 | 56848 | SPHK2 | sphingosine kinase 2 |
| 8921 | -2.969553 | 0.1452291 | 0.00048 | 0.0719689 | 0.525124 | 5551 | PRF1 | perforin 1 (pore forming protein) |
| 12182 | -2.966191 | 0.236795 | 0.00048 | 0.0719689 | 0.43533 | 79370 | BCL2L14 | BCL2-like 14 (apoptosis facilitator) |
| 4955 | -2.965515 | 0.2867153 | 0.00049 | 0.0719689 | 0.392949 | 2793 | GNGT2 | guanine nucleotide binding protein (G protein), gamma transducing activity polypeptide 2 |
| 732 | -2.957842 | 0.273102 | 0.0005 | 0.0719689 | 0.405049 | 10865 | ARID5A | AT rich interactive domain 5A (MRF1-like) |
| 6022 | -2.951514 | 0.2982232 | 0.00051 | 0.0719689 | 0.385503 | 3665 | IRF7 | interferon regulatory factor 7 |
| 13722 | -2.938681 | 0.1756304 | 0.00052 | 0.0719689 | 0.496909 | 84878 | ZBTB45 | zinc finger and BTB domain containing 45 |
| 3536 | -2.936356 | 0.1975296 | 0.00053 | 0.0719689 | 0.47551 | 23087 | TRIM35 | tripartite motif containing 35 |
| 6521 | -2.935328 | 0.2357504 | 0.00053 | 0.0719689 | 0.440048 | 4082 | MARCKS | myristoylated alanine-rich protein kinase C substrate |
| 3584 | -2.931506 | 0.1943322 | 0.00054 | 0.0719689 | 0.479197 | 23144 | ZC3H3 | zinc finger CCCH-type containing 3 |
| 6691 | -2.918385 | 0.4196836 | 0.00056 | 0.0719689 | 0.304767 | 4301 | MLLT4 | myeloid/lymphoid or mixed-lineage leukemia (trithorax homolog, Drosophila); translocated to, 4 |
| 12956 | -2.891966 | 0.3094954 | 0.0006 | 0.0719689 | 0.384208 | 81894 | SLC25A28 | solute carrier family 25 (mitochondrial iron transporter), member 28 |
| 15229 | -2.867258 | 0.2469664 | 0.00065 | 0.0719689 | 0.438618 | 9636 | ISG15 | ISG15 ubiquitin-like modifier |
| 13929 | -2.863994 | 0.1865619 | 0.00065 | 0.0719689 | 0.494961 | 85441 | HELZ2 | helicase with zinc finger 2, transcriptional coactivator |
| 1394 | -2.86313 | 0.2280991 | 0.00065 | 0.0719689 | 0.455893 | 116412 | ZNF837 | zinc finger protein 837 |
| 8790 | -2.852939 | 0.2825622 | 0.00067 | 0.0719689 | 0.41049 | 55268 | ECHDC2 | enoyl CoA hydratase domain containing 2 |
| 5586 | -2.849592 | 0.7280608 | 0.00068 | 0.0719689 | 0.170452 | 3112 | HLA-DOB | major histocompatibility complex, class II, DO beta |
| 8361 | -2.842076 | 0.177058 | 0.00069 | 0.0719689 | 0.507037 | 54788 | DNAJB12 | DnaJ (Hsp40) homolog, subfamily B, member 12 |
| 12685 | -2.839409 | 0.3700304 | 0.00069 | 0.0719689 | 0.347035 | 80301 | PLEKHO2 | pleckstrin homology domain containing, family O member 2 |
| 13807 | -2.811773 | 0.1696933 | 0.00075 | 0.0719689 | 0.518106 | 84975 | MFSD5 | major facilitator superfamily domain containing 5 |
| 8001 | -2.800583 | 0.2476594 | 0.00077 | 0.0719689 | 0.446503 | 53336 | CPXCR1 | CPX chromosome region, candidate 1 |
| 11469 | -2.791609 | 0.2301649 | 0.00079 | 0.0719689 | 0.463072 | 6992 | PPP1R11 | protein phosphatase 1, regulatory (inhibitor) subunit 11 |
| 12209 | -2.780758 | 0.1375724 | 0.00082 | 0.0719689 | 0.555209 | 79575 | ABHD8 | abhydrolase domain containing 8 |
| 2025 | -2.779409 | 0.3147578 | 0.00082 | 0.0719689 | 0.394759 | 1445 | CSK | c-src tyrosine kinase |
| 12443 | -2.778857 | 0.3545479 | 0.00082 | 0.0719689 | 0.365702 | 79873 | NUDT18 | nudix (nucleoside diphosphate linked moiety X)-type motif 18 |
| 7022 | -2.772631 | 0.3553442 | 0.00083 | 0.0719689 | 0.365966 | 4794 | NFKBIE | nuclear factor of kappa light polypeptide gene enhancer in B-cells inhibitor, epsilon |
| 12012 | -2.7681 | 0.6865602 | 0.00084 | 0.0719689 | 0.194159 | 7850 | IL1R2 | interleukin 1 receptor, type II |
| 10710 | -2.765483 | 0.3112121 | 0.00085 | 0.0719689 | 0.399306 | 64108 | RTP4 | receptor (chemosensory) transporter protein 4 |
| 3212 | -2.760486 | 0.5841673 | 0.00086 | 0.0719689 | 0.237249 | 2214 | FCGR3A | Fc fragment of IgG, low affinity IIIa, receptor (CD16a) |
| 12051 | -2.759176 | 0.2548455 | 0.00087 | 0.0719689 | 0.445691 | 79008 | SLX1B | SLX1 structure-specific endonuclease subunit homolog B (S. cerevisiae) |
| 14561 | -2.758428 | 0.6520069 | 0.00087 | 0.0719689 | 0.208611 | 9113 | LATS1 | large tumor suppressor kinase 1 |
| 13872 | -2.755683 | 0.8158352 | 0.00087 | 0.0719689 | 0.152796 | 8530 | CST7 | cystatin F (leukocystatin) |
| 2673 | -2.753784 | 0.3605024 | 0.00088 | 0.0719689 | 0.364865 | 1749 | DLX5 | distal-less homeobox 5 |
| 15529 | -2.753569 | 0.6388373 | 0.00088 | 0.0719689 | 0.214513 | 9957 | HS3ST1 | heparan sulfate (glucosamine) 3-O-sulfotransferase 1 |
| 14717 | -2.751302 | 0.1801403 | 0.00089 | 0.0719689 | 0.515119 | 92002 | FAM58A | family with sequence similarity 58, member A |
| 2877 | -2.750629 | 0.1650636 | 0.00089 | 0.0719689 | 0.530227 | 199221 | DZIP1L | DAZ interacting zinc finger protein 1-like |
| 14412 | -2.747739 | 0.227058 | 0.0009 | 0.0719689 | 0.47149 | 90324 | CCDC97 | coiled-coil domain containing 97 |
| 5695 | -2.740141 | 0.3043754 | 0.00091 | 0.0719689 | 0.407943 | 3257 | HPS1 | Hermansky-Pudlak syndrome 1 |
| 12770 | -2.737831 | 0.2787364 | 0.00092 | 0.0719689 | 0.428606 | 80776 | B9D2 | B9 protein domain 2 |
| 13847 | -2.735889 | 0.226301 | 0.00092 | 0.0719689 | 0.473701 | 8509 | NDST2 | N-deacetylase/N-sulfotransferase (heparan glucosaminyl) 2 |
| 505 | -2.735578 | 0.2908457 | 0.00092 | 0.0719689 | 0.419169 | 10564 | ARFGEF2 | ADP-ribosylation factor guanine nucleotide-exchange factor 2 (brefeldin A-inhibited) |
| 1340 | -2.732063 | 0.3577529 | 0.00093 | 0.0719689 | 0.369698 | 115704 | EVI5L | ecotropic viral integration site 5-like |
| 8488 | -2.732014 | 0.1419835 | 0.00093 | 0.0719689 | 0.556299 | 54936 | ADPRHL2 | ADP-ribosylhydrolase like 2 |
| 13015 | -2.726309 | 0.1304137 | 0.00095 | 0.0719689 | 0.569292 | 8310 | ACOX3 | acyl-CoA oxidase 3, pristanoyl |
| 7128 | -2.722595 | 0.5315521 | 0.00096 | 0.0719689 | 0.267241 | 4938 | OAS1 | 2'-5'-oligoadenylate synthetase 1, 40/46kDa |
| 13720 | -2.710714 | 0.2985598 | 0.00099 | 0.0719689 | 0.416415 | 84875 | PARP10 | poly (ADP-ribose) polymerase family, member 10 |
| 6021 | -2.706298 | 0.3363803 | 0.001 | 0.0719689 | 0.38845 | 3664 | IRF6 | interferon regulatory factor 6 |
| 11051 | -2.702052 | 0.5851165 | 0.00102 | 0.0719689 | 0.24415 | 6518 | SLC2A5 | solute carrier family 2 (facilitated glucose/fructose transporter), member 5 |
| 6104 | -2.69866 | 0.3988298 | 0.00103 | 0.0719689 | 0.346546 | 3747 | KCNC2 | potassium voltage-gated channel, Shaw-related subfamily, member 2 |
| 4710 | -2.691668 | 0.7506735 | 0.00105 | 0.0719689 | 0.180245 | 2701 | GJA4 | gap junction protein, alpha 4, 37kDa |
| 11578 | -2.68976 | 0.6987941 | 0.00105 | 0.0719689 | 0.198792 | 712 | C1QA | complement component 1, q subcomponent, A chain |
| 1721 | -2.669642 | 0.1641191 | 0.00112 | 0.0719689 | 0.54117 | 130557 | ZNF513 | zinc finger protein 513 |
| 10114 | -2.66828 | 0.7086709 | 0.00112 | 0.0719689 | 0.197728 | 58475 | MS4A7 | membrane-spanning 4-domains, subfamily A, member 7 |
| 7823 | -2.664578 | 0.5366733 | 0.00113 | 0.0719689 | 0.272275 | 51700 | CYB5R2 | cytochrome b5 reductase 2 |
| 9440 | -2.663483 | 0.9316796 | 0.00114 | 0.0719689 | 0.13138 | 56673 | C11orf16 | chromosome 11 open reading frame 16 |
| 438 | -2.659891 | 0.2304568 | 0.00115 | 0.0719689 | 0.479944 | 10488 | CREB3 | cAMP responsive element binding protein 3 |
| 9392 | -2.657549 | 0.4720602 | 0.00116 | 0.0719689 | 0.307743 | 5639 | PRRG2 | proline rich Gla (G-carboxyglutamic acid) 2 |
| 14061 | -2.654317 | 0.6071276 | 0.00117 | 0.0719689 | 0.240374 | 8676 | STX11 | syntaxin 11 |
| 4276 | -2.649362 | 0.3088027 | 0.00118 | 0.0719689 | 0.416839 | 25865 | PRKD2 | protein kinase D2 |
| 11317 | -2.644264 | 0.2364985 | 0.0012 | 0.0719689 | 0.47671 | 6772 | STAT1 | signal transducer and activator of transcription 1, 91kDa |
| 11552 | -2.643551 | 0.248838 | 0.0012 | 0.0719689 | 0.466146 | 7090 | TLE3 | transducin-like enhancer of split 3 (E(sp1) homolog, Drosophila) |
| 9591 | -2.643511 | 0.5495639 | 0.0012 | 0.0719689 | 0.268668 | 57047 | PLSCR2 | phospholipid scramblase 2 |
| 481 | -2.643313 | 0.573 | 0.00121 | 0.0719689 | 0.257401 | 10537 | UBD | ubiquitin D |
| 4750 | -2.632797 | 1.1144348 | 0.00125 | 0.0719689 | 0.096347 | 2709 | GJB5 | gap junction protein, beta 5, 31.1kDa |
| 6798 | -2.631813 | 0.3091918 | 0.00125 | 0.0719689 | 0.418965 | 441072 | FLJ31104 | uncharacterized LOC441072 |
| 5994 | -2.630468 | 0.5419997 | 0.00125 | 0.0719689 | 0.274171 | 3628 | INPP1 | inositol polyphosphate-1-phosphatase |
| 15154 | -2.618604 | 0.2733863 | 0.0013 | 0.0719689 | 0.449054 | 9546 | APBA3 | amyloid beta (A4) precursor protein-binding, family A, member 3 |
| 4979 | -2.616884 | 0.1309149 | 0.0013 | 0.0719689 | 0.581782 | 28232 | SLCO3A1 | solute carrier organic anion transporter family, member 3A1 |
| 5430 | -2.613469 | 0.2184896 | 0.00132 | 0.0719689 | 0.496786 | 29946 | SERTAD3 | SERTA domain containing 3 |
| 7236 | -2.608716 | 0.3183982 | 0.00134 | 0.0719689 | 0.415206 | 50619 | DEF6 | differentially expressed in FDCP 6 homolog (mouse) |
| 8756 | -2.606211 | 0.2770549 | 0.00135 | 0.0719689 | 0.447781 | 55231 | CCDC87 | coiled-coil domain containing 87 |
| 847 | -2.601064 | 0.4737997 | 0.00137 | 0.0719689 | 0.314561 | 11006 | LILRB4 | leukocyte immunoglobulin-like receptor, subfamily B (with TM and ITIM domains), member 4 |
| 7299 | -2.59948 | 0.3721845 | 0.00138 | 0.0719689 | 0.37803 | 5087 | PBX1 | pre-B-cell leukemia homeobox 1 |
| 825 | -2.599052 | 0.5929798 | 0.00138 | 0.0719689 | 0.254008 | 10974 | ADIRF | adipogenesis regulatory factor |
| 874 | -2.594977 | 0.3830753 | 0.0014 | 0.0719689 | 0.371322 | 11035 | RIPK3 | receptor-interacting serine-threonine kinase 3 |
| 274 | -2.594518 | 0.3580748 | 0.0014 | 0.0719689 | 0.388466 | 10288 | LILRB2 | leukocyte immunoglobulin-like receptor, subfamily B (with TM and ITIM domains), member 2 |
| 6440 | -2.593759 | 0.530314 | 0.0014 | 0.0719689 | 0.285094 | 401265 | KLHL31 | kelch-like family member 31 |
| 4971 | -2.593528 | 0.285944 | 0.0014 | 0.0719689 | 0.442409 | 2814 | GP5 | glycoprotein V (platelet) |
| 12069 | -2.587626 | 0.1441466 | 0.00143 | 0.0719689 | 0.571589 | 79034 | C7orf26 | chromosome 7 open reading frame 26 |
| 11706 | -2.58483 | 0.3716051 | 0.00144 | 0.0722461 | 0.380503 | 7318 | UBA7 | ubiquitin-like modifier activating enzyme 7 |
| 5837 | -2.575354 | 0.3111073 | 0.00148 | 0.0730195 | 0.425401 | 3430 | IFI35 | interferon-induced protein 35 |
| 15182 | -2.574878 | 0.3558678 | 0.00149 | 0.0730195 | 0.3928 | 958 | CD40 | CD40 molecule, TNF receptor superfamily member 5 |
| 1871 | -2.573875 | 0.2990836 | 0.00149 | 0.0730195 | 0.434838 | 138948 | DBH-AS1 | DBH antisense RNA 1 |
| 12384 | -2.571531 | 0.3431962 | 0.0015 | 0.0730195 | 0.402262 | 79803 | HPS6 | Hermansky-Pudlak syndrome 6 |
| 8072 | -2.57121 | 0.5636263 | 0.0015 | 0.0730195 | 0.271608 | 53831 | GPR84 | G protein-coupled receptor 84 |
| 7037 | -2.567921 | 0.734162 | 0.00151 | 0.0730931 | 0.200835 | 4818 | NKG7 | natural killer cell granule protein 7 |
| 8844 | -2.567221 | 0.238368 | 0.00152 | 0.0730931 | 0.485494 | 55324 | ABCF3 | ATP-binding cassette, sub-family F (GCN20), member 3 |
| 3624 | -2.556983 | 0.3516111 | 0.00157 | 0.0737871 | 0.398354 | 2319 | FLOT2 | flotillin 2 |
| 4510 | -2.556007 | 0.5423254 | 0.00157 | 0.0737871 | 0.284236 | 26206 | SPAG8 | sperm associated antigen 8 |
| 8103 | -2.555269 | 0.1454858 | 0.00158 | 0.0737871 | 0.574237 | 5406 | PNLIP | pancreatic lipase |
| 10692 | -2.552258 | 0.5737212 | 0.00159 | 0.0737871 | 0.269376 | 64078 | SLC28A3 | solute carrier family 28 (concentrative nucleoside transporter), member 3 |
| 8913 | -2.549471 | 0.3240285 | 0.0016 | 0.0737871 | 0.419384 | 55502 | HES6 | hes family bHLH transcription factor 6 |
| 13717 | -2.545564 | 0.1740558 | 0.00162 | 0.0737871 | 0.547158 | 84872 | ZC3H10 | zinc finger CCCH-type containing 10 |
| 10578 | -2.54418 | 0.6128289 | 0.00163 | 0.0737871 | 0.25247 | 6351 | CCL4 | chemokine (C-C motif) ligand 4 |
| 4694 | -2.534912 | 0.2361719 | 0.00168 | 0.0737871 | 0.491823 | 26984 | SEC22A | SEC22 vesicle trafficking protein homolog A (S. cerevisiae) |
| 9527 | -2.532185 | 0.27682 | 0.00169 | 0.0737871 | 0.458307 | 56948 | SDR39U1 | short chain dehydrogenase/reductase family 39U, member 1 |
| 15550 | -2.528702 | 0.2884461 | 0.0017 | 0.0737871 | 0.449544 | 9982 | FGFBP1 | fibroblast growth factor binding protein 1 |
| 12865 | -2.527123 | 0.1267995 | 0.00171 | 0.0737871 | 0.59698 | 81559 | TRIM11 | tripartite motif containing 11 |
| 6896 | -2.527066 | 0.276817 | 0.00171 | 0.0737871 | 0.459033 | 4636 | MYL5 | myosin, light chain 5, regulatory |
| 12469 | -2.52327 | 0.0965753 | 0.00173 | 0.0739498 | 0.629882 | 79903 | NAA60 | N(alpha)-acetyltransferase 60, NatF catalytic subunit |
| 2109 | -2.519259 | 1.36428 | 0.00176 | 0.0743414 | 0.068895 | 146723 | C17orf77 | chromosome 17 open reading frame 77 |
| 8556 | -2.512973 | 0.3022491 | 0.00179 | 0.0747143 | 0.441053 | 55008 | HERC6 | HECT and RLD domain containing E3 ubiquitin protein ligase family member 6 |
| 10640 | -2.511194 | 0.1573275 | 0.0018 | 0.0747143 | 0.56793 | 63925 | ZNF335 | zinc finger protein 335 |
| 8962 | -2.509351 | 0.1378332 | 0.00181 | 0.0747143 | 0.587761 | 55588 | MED29 | mediator complex subunit 29 |
| 2398 | -2.502067 | 0.1359321 | 0.00185 | 0.0752188 | 0.590613 | 153769 | SH3RF2 | SH3 domain containing ring finger 2 |
| 8920 | -2.501462 | 0.4016955 | 0.00185 | 0.0752188 | 0.372593 | 55509 | BATF3 | basic leucine zipper transcription factor, ATF-like 3 |
| 12950 | -2.498321 | 0.3033136 | 0.00187 | 0.0752188 | 0.442347 | 81888 | HYI | hydroxypyruvate isomerase (putative) |
| 12071 | -2.495894 | 0.2499439 | 0.00189 | 0.0752188 | 0.485518 | 79036 | KXD1 | KxDL motif containing 1 |
| 11313 | -2.494871 | 0.3977642 | 0.00189 | 0.0752188 | 0.376111 | 6768 | ST14 | suppression of tumorigenicity 14 (colon carcinoma) |
| 8610 | -2.491728 | 0.1049433 | 0.00191 | 0.0752188 | 0.624442 | 55072 | RNF31 | ring finger protein 31 |
| 5424 | -2.488352 | 0.2723622 | 0.00193 | 0.0755323 | 0.468124 | 29940 | DSE | dermatan sulfate epimerase |
| 8365 | -2.487361 | 0.3737664 | 0.00193 | 0.0755323 | 0.393155 | 54793 | KCTD9 | potassium channel tetramerization domain containing 9 |
| 4863 | -2.479714 | 0.880716 | 0.00198 | 0.0761071 | 0.164964 | 27299 | ADAMDEC1 | ADAM-like, decysin 1 |
| 7706 | -2.479139 | 0.5687559 | 0.00198 | 0.0761071 | 0.282088 | 51513 | ETV7 | ets variant 7 |
| 15228 | -2.476513 | 0.8003499 | 0.00199 | 0.0763451 | 0.189807 | 9635 | CLCA2 | chloride channel accessory 2 |
| 5785 | -2.473006 | 0.6061847 | 0.00202 | 0.076761 | 0.265387 | 338645 | LUZP2 | leucine zipper protein 2 |
| 1727 | -2.469622 | 0.7135117 | 0.00204 | 0.076761 | 0.221247 | 1308 | COL17A1 | collagen, type XVII, alpha 1 |
| 6431 | -2.468016 | 0.3084346 | 0.00205 | 0.076761 | 0.442848 | 401061 | NA | NA |
| 11031 | -2.456512 | 0.1537636 | 0.00211 | 0.0774259 | 0.57847 | 65083 | NOL6 | nucleolar protein 6 (RNA-associated) |
| 5881 | -2.454869 | 0.1956955 | 0.00212 | 0.0774259 | 0.538831 | 348262 | FAM195B | family with sequence similarity 195, member B |
| 8098 | -2.454346 | 0.4358748 | 0.00213 | 0.0774259 | 0.358144 | 54020 | SLC37A1 | solute carrier family 37 (glucose-6-phosphate transporter), member 1 |
| 2171 | -2.453811 | 0.2358609 | 0.00213 | 0.0774259 | 0.503384 | 148223 | C19orf25 | chromosome 19 open reading frame 25 |
| 5590 | -2.452368 | 0.9663121 | 0.00214 | 0.0774259 | 0.145488 | 3118 | HLA-DQA2 | major histocompatibility complex, class II, DQ alpha 2 |
| 14636 | -2.452019 | 0.5496337 | 0.00214 | 0.0774259 | 0.295469 | 91543 | RSAD2 | radical S-adenosyl methionine domain containing 2 |
| 8861 | -2.447999 | 0.9835905 | 0.00217 | 0.0779521 | 0.141771 | 55340 | GIMAP5 | GTPase, IMAP family member 5 |
| 3031 | -2.443659 | 0.4520986 | 0.0022 | 0.0782212 | 0.349997 | 2072 | ERCC4 | excision repair cross-complementation group 4 |
| 12883 | -2.441888 | 0.1851563 | 0.00221 | 0.0782212 | 0.550325 | 81603 | TRIM8 | tripartite motif containing 8 |
| 2475 | -2.435441 | 0.5906515 | 0.00225 | 0.0782212 | 0.277984 | 1580 | CYP4B1 | cytochrome P450, family 4, subfamily B, polypeptide 1 |
| 1998 | -2.433494 | 0.24238 | 0.00226 | 0.0782212 | 0.500716 | 143686 | SESN3 | sestrin 3 |
| 5838 | -2.432885 | 0.2821625 | 0.00227 | 0.0782212 | 0.468308 | 3431 | SP110 | SP110 nuclear body protein |
| 1030 | -2.431598 | 0.6342808 | 0.00228 | 0.0782212 | 0.258798 | 11213 | IRAK3 | interleukin-1 receptor-associated kinase 3 |
| 10651 | -2.431066 | 0.5899698 | 0.00228 | 0.0782212 | 0.278944 | 63940 | GPSM3 | G-protein signaling modulator 3 |
| 3355 | -2.431057 | 0.4187506 | 0.00228 | 0.0782212 | 0.372238 | 22865 | SLITRK3 | SLIT and NTRK-like family, member 3 |
| 11487 | -2.423084 | 0.1622396 | 0.00233 | 0.0784165 | 0.574557 | 7016 | TESK1 | testis-specific kinase 1 |
| 836 | -2.422963 | 0.4731264 | 0.00233 | 0.0784165 | 0.34087 | 10990 | LILRB5 | leukocyte immunoglobulin-like receptor, subfamily B (with TM and ITIM domains), member 5 |
| 108 | -2.421981 | 0.2505432 | 0.00234 | 0.0784165 | 0.49552 | 10114 | HIPK3 | homeodomain interacting protein kinase 3 |
| 800 | -2.421817 | 0.2900039 | 0.00234 | 0.0784165 | 0.463782 | 10943 | MSL3 | male-specific lethal 3 homolog (Drosophila) |
| 3141 | -2.41867 | 0.1437198 | 0.00236 | 0.0786527 | 0.593274 | 219770 | GJD4 | gap junction protein, delta 4, 40.1kDa |
| 6018 | -2.418205 | 0.2001315 | 0.00236 | 0.0786527 | 0.539801 | 3661 | IRF3 | interferon regulatory factor 3 |
| 6327 | -2.416745 | 1.1936588 | 0.00237 | 0.0787442 | 0.102233 | 3934 | LCN2 | lipocalin 2 |
| 10426 | -2.411095 | 0.3240789 | 0.00241 | 0.0793347 | 0.439601 | 613 | BCR | breakpoint cluster region |
| 6784 | -2.410705 | 0.6809968 | 0.00242 | 0.0793347 | 0.24216 | 440786 | LOC440786 | uncharacterized LOC440786 |
| 9233 | -2.406152 | 0.2115809 | 0.00245 | 0.0794914 | 0.531221 | 55909 | BIN3 | bridging integrator 3 |
| 3576 | -2.404816 | 0.8998909 | 0.00246 | 0.0794914 | 0.168712 | 23137 | SMC5 | structural maintenance of chromosomes 5 |
| 3978 | -2.403976 | 0.1865004 | 0.00246 | 0.0794914 | 0.554209 | 23625 | FAM89B | family with sequence similarity 89, member B |
| 1583 | -2.403628 | 0.4094126 | 0.00247 | 0.0794914 | 0.382314 | 124790 | HEXIM2 | hexamethylene bis-acetamide inducible 2 |
| 2713 | -2.400171 | 0.249886 | 0.00249 | 0.0794914 | 0.499209 | 1801 | DPH1 | diphthamide biosynthesis 1 |
| 5458 | -2.400163 | 0.2979877 | 0.00249 | 0.0794914 | 0.460817 | 29985 | SLC39A3 | solute carrier family 39 (zinc transporter), member 3 |
| 4340 | -2.39923 | 0.4165485 | 0.0025 | 0.0794914 | 0.378469 | 25939 | SAMHD1 | SAM domain and HD domain 1 |
| 15128 | -2.398283 | 0.2417139 | 0.0025 | 0.0794914 | 0.506313 | 952 | CD38 | CD38 molecule |
| 6350 | -2.397471 | 0.5352803 | 0.00251 | 0.0794914 | 0.31092 | 3965 | LGALS9 | lectin, galactoside-binding, soluble, 9 |
| 9696 | -2.391406 | 0.1450371 | 0.00255 | 0.0797297 | 0.595474 | 57192 | MCOLN1 | mucolipin 1 |
| 11422 | -2.389714 | 0.2476825 | 0.00257 | 0.0799336 | 0.502553 | 6903 | TBCC | tubulin folding cofactor C |
| 7057 | -2.380847 | 1.3701365 | 0.00264 | 0.080968 | 0.079035 | 4843 | NOS2 | nitric oxide synthase 2, inducible |
| 7038 | -2.380222 | 0.6139686 | 0.00264 | 0.080968 | 0.275371 | 482 | ATP1B2 | ATPase, Na+/K+ transporting, beta 2 polypeptide |
| 7555 | -2.37037 | 0.4047323 | 0.00271 | 0.0818967 | 0.390425 | 51291 | GMIP | GEM interacting protein |
| 5525 | -2.369497 | 0.2467673 | 0.00272 | 0.0818967 | 0.506247 | 3065 | HDAC1 | histone deacetylase 1 |
| 7506 | -2.368413 | 0.2865058 | 0.00273 | 0.0818967 | 0.474423 | 51228 | GLTP | glycolipid transfer protein |
| 14188 | -2.367051 | 0.6480626 | 0.00274 | 0.0818967 | 0.262255 | 8827 | NA | NA |
| 5839 | -2.366339 | 0.2839322 | 0.00275 | 0.0818967 | 0.476741 | 3434 | IFIT1 | interferon-induced protein with tetratricopeptide repeats 1 |
| 4137 | -2.363584 | 0.2502353 | 0.00277 | 0.0821188 | 0.504235 | 254359 | ZDHHC24 | zinc finger, DHHC-type containing 24 |
| 11589 | -2.361814 | 0.3460809 | 0.00279 | 0.0823007 | 0.431232 | 7133 | TNFRSF1B | tumor necrosis factor receptor superfamily, member 1B |
| 2665 | -2.35849 | 0.6458213 | 0.00281 | 0.0827787 | 0.264495 | 174 | AFP | alpha-fetoprotein |
| 6936 | -2.35477 | 0.5162914 | 0.00284 | 0.0827787 | 0.327451 | 4688 | NCF2 | neutrophil cytosolic factor 2 |
| 2889 | -2.354686 | 0.1803498 | 0.00285 | 0.0827787 | 0.566617 | 199745 | THAP8 | THAP domain containing 8 |
| 9276 | -2.35428 | 0.2612375 | 0.00285 | 0.0827787 | 0.496599 | 5603 | MAPK13 | mitogen-activated protein kinase 13 |
| 4335 | -2.352956 | 0.577173 | 0.00286 | 0.0827787 | 0.296754 | 259307 | IL4I1 | interleukin 4 induced 1 |
| 14094 | -2.350566 | 0.3766521 | 0.00289 | 0.0827787 | 0.411926 | 8717 | TRADD | TNFRSF1A-associated via death domain |
| 13209 | -2.347677 | 0.1870334 | 0.00291 | 0.0827787 | 0.561436 | 83931 | STK40 | serine/threonine kinase 40 |
| 7019 | -2.346014 | 0.356804 | 0.00292 | 0.0827787 | 0.426169 | 4791 | NFKB2 | nuclear factor of kappa light polypeptide gene enhancer in B-cells 2 (p49/p100) |
| 15415 | -2.345712 | 0.2206113 | 0.00293 | 0.0827787 | 0.531863 | 9830 | TRIM14 | tripartite motif containing 14 |
| 10673 | -2.345384 | 0.2391759 | 0.00293 | 0.0827787 | 0.516096 | 64005 | MYO1G | myosin IG |
| 117 | -2.345234 | 0.1685806 | 0.00293 | 0.0827787 | 0.57888 | 10126 | DNAL4 | dynein, axonemal, light chain 4 |
| 8930 | -2.343781 | 0.1937859 | 0.00294 | 0.0827787 | 0.555843 | 55527 | FEM1A | fem-1 homolog a (C. elegans) |
| 12461 | -2.343334 | 0.3192465 | 0.00295 | 0.0827787 | 0.453418 | 79894 | ZNF672 | zinc finger protein 672 |
| 11684 | -2.339029 | 0.3438513 | 0.00298 | 0.0827787 | 0.43632 | 7287 | TULP1 | tubby like protein 1 |
| 10468 | -2.338826 | 0.4185928 | 0.00298 | 0.0827787 | 0.386558 | 6199 | RPS6KB2 | ribosomal protein S6 kinase, 70kDa, polypeptide 2 |
| 12413 | -2.337852 | 0.3574939 | 0.00299 | 0.0827787 | 0.426958 | 7984 | ARHGEF5 | Rho guanine nucleotide exchange factor (GEF) 5 |
| 814 | -2.337772 | 0.1491379 | 0.00299 | 0.0827787 | 0.598448 | 10960 | LMAN2 | lectin, mannose-binding 2 |
| 3416 | -2.336381 | 0.2386 | 0.00301 | 0.0827787 | 0.517891 | 22933 | SIRT2 | sirtuin 2 |
| 13101 | -2.331137 | 0.0644513 | 0.00305 | 0.0827787 | 0.687207 | 83606 | GUCD1 | guanylyl cyclase domain containing 1 |
| 10590 | -2.331053 | 0.847048 | 0.00305 | 0.0827787 | 0.194058 | 6366 | CCL21 | chemokine (C-C motif) ligand 21 |
| 68 | -2.330856 | 0.2520293 | 0.00305 | 0.0827787 | 0.507565 | 10072 | DPP3 | dipeptidyl-peptidase 3 |
| 12994 | -2.328378 | 0.2040788 | 0.00308 | 0.0828738 | 0.5488 | 8263 | F8A1 | coagulation factor VIII-associated 1 |
| 4222 | -2.327145 | 0.1664014 | 0.00309 | 0.0828738 | 0.583373 | 2580 | GAK | cyclin G associated kinase |
| 13672 | -2.326384 | 0.1449691 | 0.00309 | 0.0828738 | 0.603993 | 84807 | NFKBID | nuclear factor of kappa light polypeptide gene enhancer in B-cells inhibitor, delta |
| 5178 | -2.325481 | 0.2234759 | 0.0031 | 0.0828738 | 0.532303 | 2889 | RAPGEF1 | Rap guanine nucleotide exchange factor (GEF) 1 |
| 14998 | -2.323085 | 0.4050261 | 0.00312 | 0.0829011 | 0.397631 | 94 | ACVRL1 | activin A receptor type II-like 1 |
| 5481 | -2.3203 | 0.7843802 | 0.00315 | 0.0830269 | 0.216266 | 3003 | GZMK | granzyme K (granzyme 3; tryptase II) |
| 8912 | -2.318716 | 0.2026376 | 0.00316 | 0.0830269 | 0.551444 | 55501 | CHST12 | carbohydrate (chondroitin 4) sulfotransferase 12 |
| 11337 | -2.317379 | 0.1716625 | 0.00318 | 0.0830269 | 0.579774 | 6794 | STK11 | serine/threonine kinase 11 |
| 13321 | -2.317167 | 0.3138939 | 0.00318 | 0.0830269 | 0.46139 | 84179 | MFSD7 | major facilitator superfamily domain containing 7 |
| 7821 | -2.315464 | 0.3112557 | 0.00319 | 0.0830269 | 0.463612 | 517 | ATP5G2 | ATP synthase, H+ transporting, mitochondrial Fo complex, subunit C2 (subunit 9) |
| 1143 | -2.312057 | 0.2874564 | 0.00322 | 0.0830569 | 0.482181 | 113179 | ADAT3 | adenosine deaminase, tRNA-specific 3 |
| 12136 | -2.309993 | 0.2908715 | 0.00325 | 0.0831285 | 0.479864 | 79155 | TNIP2 | TNFAIP3 interacting protein 2 |
| 12998 | -2.308028 | 0.2723613 | 0.00327 | 0.0832309 | 0.494595 | 8270 | LAGE3 | L antigen family, member 3 |
| 1134 | -2.307829 | 0.271798 | 0.00327 | 0.0832309 | 0.495071 | 11314 | CD300A | CD300a molecule |
| 7971 | -2.306977 | 0.2189256 | 0.00328 | 0.0832309 | 0.538888 | 5301 | PIN1P1 | peptidylprolyl cis/trans isomerase, NIMA-interacting 1 pseudogene 1 |
| 10324 | -2.30642 | 0.5051371 | 0.00329 | 0.0832309 | 0.341072 | 6013 | RLN1 | relaxin 1 |
| 9235 | -2.304882 | 0.4311966 | 0.0033 | 0.0832737 | 0.384114 | 55911 | APOBR | apolipoprotein B receptor |
| 12243 | -2.303818 | 0.6629471 | 0.00331 | 0.0832737 | 0.265417 | 79626 | TNFAIP8L2 | tumor necrosis factor, alpha-induced protein 8-like 2 |
| 1114 | -2.302317 | 0.2900492 | 0.00333 | 0.0832737 | 0.481667 | 112869 | CCDC101 | coiled-coil domain containing 101 |
| 8794 | -2.300629 | 0.155345 | 0.00334 | 0.0832737 | 0.597407 | 55272 | IMP3 | IMP3, U3 small nucleolar ribonucleoprotein |
| 13712 | -2.298247 | 0.4400557 | 0.00337 | 0.0832737 | 0.379776 | 84868 | HAVCR2 | hepatitis A virus cellular receptor 2 |
| 14106 | -2.297025 | 0.2280249 | 0.00338 | 0.0832737 | 0.532555 | 8729 | GBF1 | golgi brefeldin A resistant guanine nucleotide exchange factor 1 |
| 12266 | -2.295444 | 0.1705564 | 0.0034 | 0.0832737 | 0.583799 | 79651 | RHBDF2 | rhomboid 5 homolog 2 (Drosophila) |
| 9218 | -2.295398 | 0.7796295 | 0.0034 | 0.0832737 | 0.221518 | 55891 | LENEP | lens epithelial protein |
| 12980 | -2.295158 | 0.2015276 | 0.0034 | 0.0832737 | 0.555769 | 8225 | GTPBP6 | GTP binding protein 6 (putative) |
| 6707 | -2.29399 | 0.8026905 | 0.00341 | 0.0832737 | 0.21374 | 4321 | MMP12 | matrix metallopeptidase 12 (macrophage elastase) |
| 2161 | -2.2937 | 0.1975347 | 0.00342 | 0.0832737 | 0.559517 | 148022 | TICAM1 | toll-like receptor adaptor molecule 1 |
| 3495 | -2.290748 | 0.5987971 | 0.00345 | 0.0835638 | 0.296097 | 2304 | FOXE1 | forkhead box E1 (thyroid transcription factor 2) |
| 12552 | -2.289975 | 0.2048102 | 0.00346 | 0.0835638 | 0.553615 | 80019 | UBTD1 | ubiquitin domain containing 1 |
| 10592 | -2.285903 | 0.3397212 | 0.0035 | 0.0836102 | 0.447536 | 6368 | CCL23 | chemokine (C-C motif) ligand 23 |
| 4396 | -2.285383 | 0.448923 | 0.0035 | 0.0836102 | 0.376513 | 26018 | LRIG1 | leucine-rich repeats and immunoglobulin-like domains 1 |
| 14015 | -2.282328 | 0.1788922 | 0.00353 | 0.0837176 | 0.577926 | 8625 | RFXANK | regulatory factor X-associated ankyrin-containing protein |
| 3515 | -2.281683 | 0.176097 | 0.00354 | 0.0837176 | 0.580576 | 23061 | TBC1D9B | TBC1 domain family, member 9B (with GRAM domain) |
| 14455 | -2.278086 | 0.5219978 | 0.00358 | 0.0837176 | 0.336529 | 9051 | PSTPIP1 | proline-serine-threonine phosphatase interacting protein 1 |
| 614 | -2.277381 | 0.3958922 | 0.00359 | 0.0837176 | 0.410792 | 10686 | CLDN16 | claudin 16 |
| 3074 | -2.275894 | 0.2907838 | 0.0036 | 0.0837176 | 0.48516 | 2139 | EYA2 | eyes absent homolog 2 (Drosophila) |
| 386 | -2.275321 | 0.4023633 | 0.00361 | 0.0837176 | 0.406948 | 10435 | CDC42EP2 | CDC42 effector protein (Rho GTPase binding) 2 |
| 10860 | -2.274598 | 0.6646512 | 0.00362 | 0.0837176 | 0.269196 | 64581 | CLEC7A | C-type lectin domain family 7, member A |
| 1669 | -2.27258 | 0.5320353 | 0.00364 | 0.0837176 | 0.332123 | 128434 | VSTM2L | V-set and transmembrane domain containing 2 like |
| 11558 | -2.272109 | 0.4770781 | 0.00364 | 0.0837176 | 0.362232 | 7097 | TLR2 | toll-like receptor 2 |
| 13100 | -2.27186 | 0.1691303 | 0.00364 | 0.0837176 | 0.588356 | 83605 | CCM2 | cerebral cavernous malformation 2 |
| 1040 | -2.267741 | 0.2939675 | 0.00369 | 0.0844562 | 0.48399 | 11224 | RPL35 | ribosomal protein L35 |
| 3155 | -2.265159 | 0.401619 | 0.00372 | 0.0845498 | 0.409063 | 220002 | CYB561A3 | cytochrome b561 family, member A3 |
| 3943 | -2.263668 | 0.317599 | 0.00374 | 0.0847214 | 0.466981 | 23581 | CASP14 | caspase 14, apoptosis-related cysteine peptidase |
| 137 | -2.259912 | 0.3345175 | 0.00378 | 0.0847422 | 0.455343 | 10148 | EBI3 | Epstein-Barr virus induced 3 |
| 6313 | -2.259473 | 0.3352142 | 0.00379 | 0.0847422 | 0.454916 | 3914 | LAMB3 | laminin, beta 3 |
| 6299 | -2.258366 | 0.6442966 | 0.0038 | 0.0847422 | 0.280526 | 3904 | LAIR2 | leukocyte-associated immunoglobulin-like receptor 2 |
| 7444 | -2.258235 | 0.4867737 | 0.0038 | 0.0847422 | 0.358996 | 51147 | ING4 | inhibitor of growth family, member 4 |
| 8015 | -2.258018 | 0.2305138 | 0.00381 | 0.0847422 | 0.536191 | 5337 | PLD1 | phospholipase D1, phosphatidylcholine-specific |
| 4023 | -2.25776 | 0.1742568 | 0.00381 | 0.0847422 | 0.585579 | 23761 | PISD | phosphatidylserine decarboxylase |
| 7893 | -2.254663 | 0.5860627 | 0.00384 | 0.085032 | 0.307895 | 5199 | CFP | complement factor properdin |
| 2294 | -2.253624 | 0.9600166 | 0.00386 | 0.085032 | 0.171769 | 151174 | LOC151174 | uncharacterized LOC151174 |
| 13338 | -2.253152 | 0.3841224 | 0.00386 | 0.085032 | 0.422392 | 84217 | ZMYND12 | zinc finger, MYND-type containing 12 |
| 14696 | -2.252213 | 0.3673112 | 0.00387 | 0.085032 | 0.43378 | 91875 | TTC5 | tetratricopeptide repeat domain 5 |
| 14629 | -2.251919 | 0.4051489 | 0.00387 | 0.085032 | 0.408946 | 9152 | SLC6A5 | solute carrier family 6 (neurotransmitter transporter), member 5 |
| 14028 | -2.248648 | 1.0380787 | 0.00391 | 0.0855067 | 0.152684 | 8639 | AOC3 | amine oxidase, copper containing 3 |
| 9538 | -2.243752 | 0.3468188 | 0.00397 | 0.0857182 | 0.449234 | 5696 | PSMB8 | proteasome (prosome, macropain) subunit, beta type, 8 |
| 1794 | -2.240582 | 0.5438404 | 0.00401 | 0.0861718 | 0.331189 | 134510 | UBLCP1 | ubiquitin-like domain containing CTD phosphatase 1 |
| 3658 | -2.238111 | 0.3627894 | 0.00404 | 0.0861718 | 0.439124 | 23227 | NA | NA |
| 8882 | -2.233523 | 0.3968419 | 0.0041 | 0.0861718 | 0.417277 | 55365 | TMEM176A | transmembrane protein 176A |
| 11457 | -2.232919 | 0.3978059 | 0.00411 | 0.0861718 | 0.416753 | 6948 | TCN2 | transcobalamin II |
| 1929 | -2.231859 | 0.2565323 | 0.00412 | 0.0861718 | 0.51877 | 140706 | CCM2L | cerebral cavernous malformation 2-like |
| 6660 | -2.231835 | 0.1992584 | 0.00412 | 0.0861718 | 0.566836 | 4254 | KITLG | KIT ligand |
| 5653 | -2.230656 | 0.1388439 | 0.00414 | 0.0861718 | 0.622523 | 3200 | HOXA3 | homeobox A3 |
| 9641 | -2.229568 | 0.6143346 | 0.00415 | 0.0861718 | 0.298622 | 57125 | PLXDC1 | plexin domain containing 1 |
| 7420 | -2.229456 | 0.2721285 | 0.00415 | 0.0861718 | 0.506774 | 51117 | COQ4 | coenzyme Q4 |
| 14203 | -2.228906 | 0.624548 | 0.00416 | 0.0861718 | 0.294053 | 8843 | HCAR3 | hydroxycarboxylic acid receptor 3 |
| 6084 | -2.227368 | 0.2062294 | 0.00417 | 0.0861718 | 0.561405 | 3737 | KCNA2 | potassium voltage-gated channel, shaker-related subfamily, member 2 |
| 6655 | -2.22373 | 0.7083353 | 0.00423 | 0.0861718 | 0.259162 | 4246 | SCGB2A1 | secretoglobin, family 2A, member 1 |
| 13071 | -2.223358 | 0.2441605 | 0.00423 | 0.0861718 | 0.530079 | 83483 | PLVAP | plasmalemma vesicle associated protein |
| 7844 | -2.223245 | 0.4862348 | 0.00423 | 0.0861718 | 0.365042 | 51734 | MSRB1 | methionine sulfoxide reductase B1 |
| 5343 | -2.222877 | 0.6228249 | 0.00424 | 0.0861718 | 0.295812 | 29760 | BLNK | B-cell linker |
| 6161 | -2.222603 | 0.1459728 | 0.00424 | 0.0861718 | 0.616778 | 3786 | KCNQ3 | potassium voltage-gated channel, KQT-like subfamily, member 3 |
| 1097 | -2.221685 | 0.336275 | 0.00425 | 0.0861718 | 0.460196 | 11277 | TREX1 | three prime repair exonuclease 1 |
| 299 | -2.221007 | 0.3188801 | 0.00426 | 0.0861718 | 0.472798 | 10318 | TNIP1 | TNFAIP3 interacting protein 1 |
| 4741 | -2.220437 | 0.8421915 | 0.00427 | 0.0861718 | 0.211333 | 27074 | LAMP3 | lysosomal-associated membrane protein 3 |
| 15512 | -2.220237 | 0.643016 | 0.00427 | 0.0861718 | 0.287177 | 9938 | ARHGAP25 | Rho GTPase activating protein 25 |
| 11563 | -2.220191 | 0.6461036 | 0.00427 | 0.0861718 | 0.285823 | 7100 | TLR5 | toll-like receptor 5 |
| 13523 | -2.216348 | 0.8428267 | 0.00432 | 0.086478 | 0.211732 | 84525 | HOPX | HOP homeobox |
| 5585 | -2.214037 | 0.9138842 | 0.00435 | 0.0869224 | 0.190165 | 3111 | HLA-DOA | major histocompatibility complex, class II, DO alpha |
| 12738 | -2.211777 | 0.5245921 | 0.00438 | 0.0873288 | 0.345989 | 80709 | AKNA | AT-hook transcription factor |
| 12529 | -2.21096 | 0.5746875 | 0.00439 | 0.0873576 | 0.320546 | 79983 | POF1B | premature ovarian failure, 1B |
| 7478 | -2.209988 | 0.278316 | 0.00441 | 0.0873718 | 0.504982 | 5119 | CHMP1A | charged multivesicular body protein 1A |
| 6558 | -2.206107 | 0.262504 | 0.00445 | 0.0874659 | 0.517962 | 4125 | MAN2B1 | mannosidase, alpha, class 2B, member 1 |
| 3541 | -2.205783 | 0.3666689 | 0.00446 | 0.0874659 | 0.441747 | 23092 | ARHGAP26 | Rho GTPase activating protein 26 |
| 13397 | -2.205619 | 0.1546282 | 0.00446 | 0.0874659 | 0.610922 | 84282 | RNF135 | ring finger protein 135 |
| 5726 | -2.20037 | 0.3070383 | 0.00453 | 0.0880774 | 0.484777 | 3300 | DNAJB2 | DnaJ (Hsp40) homolog, subfamily B, member 2 |
| 7196 | -2.199527 | 0.2766156 | 0.00455 | 0.0880774 | 0.507933 | 5031 | P2RY6 | pyrimidinergic receptor P2Y, G-protein coupled, 6 |
| 12368 | -2.196383 | 0.3085282 | 0.00459 | 0.0880774 | 0.484314 | 79784 | MYH14 | myosin, heavy chain 14, non-muscle |
| 6986 | -2.194659 | 0.4412972 | 0.00461 | 0.0880774 | 0.395967 | 4741 | NEFM | neurofilament, medium polypeptide |
| 11138 | -2.192878 | 0.4422031 | 0.00463 | 0.0880774 | 0.395719 | 6595 | SMARCA2 | SWI/SNF related, matrix associated, actin dependent regulator of chromatin, subfamily a, member 2 |
| 12688 | -2.191868 | 0.4965258 | 0.00465 | 0.0880774 | 0.364527 | 80305 | TRABD | TraB domain containing |
| 6661 | -2.191762 | 0.3096375 | 0.00465 | 0.0880774 | 0.484236 | 4255 | MGMT | O-6-methylguanine-DNA methyltransferase |
| 11291 | -2.191452 | 0.4446633 | 0.00465 | 0.0880774 | 0.394481 | 6737 | TRIM21 | tripartite motif containing 21 |
| 11403 | -2.191167 | 0.1683873 | 0.00466 | 0.0880774 | 0.600223 | 6881 | TAF10 | TAF10 RNA polymerase II, TATA box binding protein (TBP)-associated factor, 30kDa |
| 3024 | -2.190551 | 0.1247932 | 0.00467 | 0.0880774 | 0.641389 | 2067 | ERCC1 | excision repair cross-complementation group 1 |
| 6179 | -2.189178 | 0.1369595 | 0.00469 | 0.0881525 | 0.629832 | 381 | ARF5 | ADP-ribosylation factor 5 |
| 3407 | -2.18734 | 0.2143538 | 0.00472 | 0.0885044 | 0.560316 | 22921 | MSRB2 | methionine sulfoxide reductase B2 |
| 11581 | -2.186503 | 0.6850233 | 0.00473 | 0.0885709 | 0.274621 | 7124 | TNF | tumor necrosis factor |
| 10793 | -2.184886 | 0.5317569 | 0.00475 | 0.0887363 | 0.3467 | 64333 | ARHGAP9 | Rho GTPase activating protein 9 |
| 9341 | -2.184409 | 0.5751651 | 0.00476 | 0.0887363 | 0.324721 | 56169 | GSDMC | gasdermin C |
| 7936 | -2.183649 | 0.1738448 | 0.00477 | 0.0887363 | 0.596328 | 5261 | PHKG2 | phosphorylase kinase, gamma 2 (testis) |
| 1488 | -2.18344 | 0.363839 | 0.00477 | 0.0887363 | 0.447329 | 119504 | ANAPC16 | anaphase promoting complex subunit 16 |
| 6665 | -2.173407 | 0.5978238 | 0.00492 | 0.0903042 | 0.315606 | 4261 | CIITA | class II, major histocompatibility complex, transactivator |
| 14812 | -2.168237 | 0.071346 | 0.005 | 0.091109 | 0.698187 | 92595 | ZNF764 | zinc finger protein 764 |
| 6077 | -2.167666 | 0.3842173 | 0.005 | 0.091109 | 0.436369 | 3728 | JUP | junction plakoglobin |
| 13313 | -2.162159 | 0.4429024 | 0.00509 | 0.09207 | 0.400472 | 84166 | NLRC5 | NLR family, CARD domain containing 5 |
| 2008 | -2.16115 | 0.2218943 | 0.0051 | 0.09207 | 0.557878 | 144097 | C11orf84 | chromosome 11 open reading frame 84 |
| 8474 | -2.161035 | 0.2943786 | 0.0051 | 0.09207 | 0.500494 | 54921 | CHTF8 | CTF8, chromosome transmission fidelity factor 8 homolog (S. cerevisiae) |
| 10604 | 2.576056 | 0.5401558 | 0.00148 | 0.0730195 | 3.53935 | 6383 | SDC2 | syndecan 2 |
| 7224 | 2.5880138 | 0.5376887 | 0.00143 | 0.0719689 | 3.544455 | 5053 | PAH | phenylalanine hydroxylase |
| 4549 | 2.5886107 | 0.2852704 | 0.00142 | 0.0719689 | 2.254132 | 26268 | FBXO9 | F-box protein 9 |
| 12408 | 2.5899597 | 0.2206686 | 0.00142 | 0.0719689 | 2.00815 | 79834 | PEAK1 | pseudopodium-enriched atypical kinase 1 |
| 13280 | 2.5937852 | 0.4550035 | 0.0014 | 0.0719689 | 3.063471 | 84085 | FBXO30 | F-box protein 30 |
| 7467 | 2.5950168 | 0.3474219 | 0.0014 | 0.0719689 | 2.525831 | 51175 | TUBE1 | tubulin, epsilon 1 |
| 12256 | 2.5959649 | 0.2569927 | 0.00139 | 0.0719689 | 2.147258 | 79642 | ARSJ | arylsulfatase family, member J |
| 10080 | 2.5975328 | 0.4302326 | 0.00139 | 0.0719689 | 2.934588 | 5806 | PTX3 | pentraxin 3, long |
| 740 | 2.6004693 | 0.5668023 | 0.00137 | 0.0719689 | 3.758244 | 10874 | NMU | neuromedin U |
| 8320 | 2.6023626 | 0.3109115 | 0.00136 | 0.0719689 | 2.371054 | 5471 | PPAT | phosphoribosyl pyrophosphate amidotransferase |
| 13803 | 2.6025613 | 0.4111128 | 0.00136 | 0.0719689 | 2.84101 | 8497 | PPFIA4 | protein tyrosine phosphatase, receptor type, f polypeptide (PTPRF), interacting protein (liprin), alpha 4 |
| 4329 | 2.6110864 | 0.1782723 | 0.00133 | 0.0719689 | 1.870432 | 25927 | CNRIP1 | cannabinoid receptor interacting protein 1 |
| 2265 | 2.6258146 | 0.285861 | 0.00127 | 0.0719689 | 2.28307 | 150465 | TTL | tubulin tyrosine ligase |
| 343 | 2.6280393 | 0.4853565 | 0.00126 | 0.0719689 | 3.285849 | 10388 | SYCP2 | synaptonemal complex protein 2 |
| 11390 | 2.6354946 | 0.4024286 | 0.00124 | 0.0719689 | 2.833488 | 687 | KLF9 | Kruppel-like factor 9 |
| 13829 | 2.6369307 | 0.1761017 | 0.00123 | 0.0719689 | 1.874609 | 8501 | SLC43A1 | solute carrier family 43 (amino acid system L transporter), member 1 |
| 10769 | 2.6416741 | 0.2684928 | 0.00121 | 0.0719689 | 2.22266 | 64240 | ABCG5 | ATP-binding cassette, sub-family G (WHITE), member 5 |
| 10757 | 2.6469442 | 0.2819414 | 0.00119 | 0.0719689 | 2.281819 | 6422 | SFRP1 | secreted frizzled-related protein 1 |
| 10973 | 2.6472864 | 0.5972035 | 0.00119 | 0.0719689 | 4.069713 | 6490 | PMEL | premelanosome protein |
| 641 | 2.6484702 | 0.3158934 | 0.00119 | 0.0719689 | 2.429721 | 10732 | TCFL5 | transcription factor-like 5 (basic helix-loop-helix) |
| 14108 | 2.6512033 | 0.148601 | 0.00118 | 0.0719689 | 1.788297 | 8731 | RNMT | RNA (guanine-7-) methyltransferase |
| 1406 | 2.6610757 | 0.2060236 | 0.00115 | 0.0719689 | 1.992411 | 116519 | APOA5 | apolipoprotein A-V |
| 4459 | 2.6689989 | 0.2788294 | 0.00112 | 0.0719689 | 2.284368 | 26108 | PYGO1 | pygopus family PHD finger 1 |
| 8034 | 2.6772922 | 0.5624019 | 0.00109 | 0.0719689 | 3.876365 | 5354 | PLP1 | proteolipid protein 1 |
| 4624 | 2.6776507 | 0.1899405 | 0.00109 | 0.0719689 | 1.942137 | 2653 | GCSH | glycine cleavage system protein H (aminomethyl carrier) |
| 6485 | 2.6844189 | 0.3117765 | 0.00107 | 0.0719689 | 2.440411 | 4047 | LSS | lanosterol synthase (2,3-oxidosqualene-lanosterol cyclase) |
| 801 | 2.685938 | 0.2127796 | 0.00107 | 0.0719689 | 2.030666 | 10944 | C11orf58 | chromosome 11 open reading frame 58 |
| 8550 | 2.7034818 | 0.2879895 | 0.00101 | 0.0719689 | 2.348853 | 55002 | TMCO3 | transmembrane and coiled-coil domains 3 |
| 4663 | 2.7068894 | 0.3194541 | 0.001 | 0.0719689 | 2.494379 | 2673 | GFPT1 | glutamine--fructose-6-phosphate transaminase 1 |
| 11161 | 2.7088489 | 0.1939327 | 0.001 | 0.0719689 | 1.971942 | 660 | BMX | BMX non-receptor tyrosine kinase |
| 7500 | 2.7151416 | 0.3999907 | 0.00098 | 0.0719689 | 2.910706 | 5122 | PCSK1 | proprotein convertase subtilisin/kexin type 1 |
| 11250 | 2.7227887 | 0.1973418 | 0.00096 | 0.0719689 | 1.991617 | 6690 | SPINK1 | serine peptidase inhibitor, Kazal type 1 |
| 8827 | 2.7232435 | 0.357262 | 0.00096 | 0.0719689 | 2.693735 | 55304 | SPTLC3 | serine palmitoyltransferase, long chain base subunit 3 |
| 940 | 2.7422817 | 0.2924835 | 0.00091 | 0.0719689 | 2.398215 | 11112 | HIBADH | 3-hydroxyisobutyrate dehydrogenase |
| 9403 | 2.7430877 | 0.3428416 | 0.00091 | 0.0719689 | 2.639874 | 5651 | TMPRSS15 | transmembrane protease, serine 15 |
| 10625 | 2.7443578 | 0.4953715 | 0.0009 | 0.0719689 | 3.530112 | 63904 | DUSP21 | dual specificity phosphatase 21 |
| 1997 | 2.7655501 | 0.3624781 | 0.00085 | 0.0719689 | 2.763014 | 143684 | FAM76B | family with sequence similarity 76, member B |
| 9405 | 2.7802586 | 0.357553 | 0.00082 | 0.0719689 | 2.751747 | 56521 | DNAJC12 | DnaJ (Hsp40) homolog, subfamily C, member 12 |
| 1693 | 2.7840792 | 0.2714537 | 0.00081 | 0.0719689 | 2.333746 | 1295 | COL8A1 | collagen, type VIII, alpha 1 |
| 2623 | 2.7916752 | 0.4435455 | 0.00079 | 0.0719689 | 3.263472 | 1690 | COCH | cochlin |
| 4878 | 2.8116832 | 0.5168356 | 0.00075 | 0.0719689 | 3.796595 | 27324 | TOX3 | TOX high mobility group box family member 3 |
| 5516 | 2.8205547 | 0.5086761 | 0.00073 | 0.0719689 | 3.752273 | 3053 | SERPIND1 | serpin peptidase inhibitor, clade D (heparin cofactor), member 1 |
| 12796 | 2.8269955 | 0.2686446 | 0.00072 | 0.0719689 | 2.351454 | 80854 | SETD7 | SET domain containing (lysine methyltransferase) 7 |
| 6825 | 2.8312433 | 0.2818595 | 0.00071 | 0.0719689 | 2.416336 | 444 | ASPH | aspartate beta-hydroxylase |
| 3229 | 2.8362517 | 0.1933091 | 0.0007 | 0.0719689 | 2.033439 | 221718 | LINC00518 | long intergenic non-protein coding RNA 518 |
| 12367 | 2.8511009 | 0.3992874 | 0.00067 | 0.0719689 | 3.066403 | 79783 | SUGCT | succinylCoA:glutarate-CoA transferase |
| 14295 | 2.8714257 | 0.2473117 | 0.00064 | 0.0719689 | 2.284193 | 8973 | CHRNA6 | cholinergic receptor, nicotinic, alpha 6 (neuronal) |
| 4301 | 2.8741101 | 0.4209647 | 0.00063 | 0.0719689 | 3.23081 | 259 | AMBP | alpha-1-microglobulin/bikunin precursor |
| 9759 | 2.891154 | 0.3778139 | 0.00061 | 0.0719689 | 2.983847 | 574 | BAGE | B melanoma antigen |
| 14733 | 2.8930922 | 0.145439 | 0.0006 | 0.0719689 | 1.873777 | 92106 | OXNAD1 | oxidoreductase NAD-binding domain containing 1 |
| 15119 | 2.8971706 | 0.4038941 | 0.00059 | 0.0719689 | 3.151448 | 9510 | ADAMTS1 | ADAM metallopeptidase with thrombospondin type 1 motif, 1 |
| 669 | 2.8995147 | 0.2450886 | 0.00059 | 0.0719689 | 2.292459 | 10769 | PLK2 | polo-like kinase 2 |
| 13981 | 2.9110395 | 0.1565346 | 0.00057 | 0.0719689 | 1.92368 | 857 | CAV1 | caveolin 1, caveolae protein, 22kDa |
| 8795 | 2.9190029 | 0.2473397 | 0.00056 | 0.0719689 | 2.315801 | 55273 | TMEM100 | transmembrane protein 100 |
| 3609 | 2.9289801 | 0.3267197 | 0.00054 | 0.0719689 | 2.728588 | 23171 | GPD1L | glycerol-3-phosphate dehydrogenase 1-like |
| 9391 | 2.930565 | 0.4261952 | 0.00054 | 0.0719689 | 3.341412 | 5638 | PRRG1 | proline rich Gla (G-carboxyglutamic acid) 1 |
| 6620 | 2.9363865 | 0.2923524 | 0.00053 | 0.0719689 | 2.550713 | 4199 | ME1 | malic enzyme 1, NADP(+)-dependent, cytosolic |
| 10378 | 2.936864 | 0.2505109 | 0.00053 | 0.0719689 | 2.342807 | 60529 | ALX4 | ALX homeobox 4 |
| 10091 | 2.9375224 | 0.3836514 | 0.00053 | 0.0719689 | 3.072933 | 58189 | WFDC1 | WAP four-disulfide core domain 1 |
| 7115 | 2.9413632 | 0.4250887 | 0.00052 | 0.0719689 | 3.348734 | 4922 | NTS | neurotensin |
| 5036 | 2.9858139 | 0.1990035 | 0.00046 | 0.0719689 | 2.136009 | 283859 | NA | NA |
| 3287 | 2.993731 | 0.3579815 | 0.00045 | 0.0719689 | 2.976792 | 2258 | FGF13 | fibroblast growth factor 13 |
| 1874 | 3.0413488 | 0.3421861 | 0.00039 | 0.0719689 | 2.929696 | 139081 | MAGEC3 | melanoma antigen family C, 3 |
| 9132 | 3.0524263 | 0.1359209 | 0.00038 | 0.0719689 | 1.901041 | 5578 | PRKCA | protein kinase C, alpha |
| 3392 | 3.0625102 | 0.2540605 | 0.00038 | 0.0719689 | 2.448088 | 22903 | BTBD3 | BTB (POZ) domain containing 3 |
| 1042 | 3.1335329 | 0.326279 | 0.00031 | 0.0719689 | 2.923931 | 11227 | GALNT5 | UDP-N-acetyl-alpha-D-galactosamine:polypeptide N-acetylgalactosaminyltransferase 5 (GalNAc-T5) |
| 7182 | 3.1501647 | 0.6632924 | 0.00029 | 0.0719689 | 6.138018 | 5015 | OTX2 | orthodenticle homeobox 2 |
| 5015 | 3.1562409 | 0.2848092 | 0.00029 | 0.0719689 | 2.691178 | 283417 | DPY19L2 | dpy-19-like 2 (C. elegans) |
| 5866 | 3.1981905 | 0.339249 | 0.00026 | 0.0719689 | 3.076584 | 3475 | IFRD1 | interferon-related developmental regulator 1 |
| 6749 | 3.2368563 | 0.2145276 | 0.00024 | 0.0719689 | 2.357448 | 439948 | NA | NA |
| 14474 | 3.2596787 | 0.1164448 | 0.00022 | 0.0719689 | 1.900305 | 90625 | ERVH48-1 | endogenous retrovirus group 48, member 1 |
| 6553 | 3.3284221 | 0.4692716 | 0.00018 | 0.0719689 | 4.347304 | 412 | STS | steroid sulfatase (microsomal), isozyme S |
| 12991 | 3.3297146 | 0.4510243 | 0.00018 | 0.0719689 | 4.1704 | 825 | CAPN3 | calpain 3, (p94) |
| 6272 | 3.3574737 | 0.1680103 | 0.00017 | 0.0719689 | 2.184267 | 389002 | NA | NA |
| 10700 | 3.4661162 | 0.462758 | 0.00013 | 0.0719689 | 4.548061 | 64093 | SMOC1 | SPARC related modular calcium binding 1 |
| 576 | 3.6223664 | 0.4226391 | 8.8E-05 | 0.0713227 | 4.402845 | 10642 | IGF2BP1 | insulin-like growth factor 2 mRNA binding protein 1 |
| 6924 | 3.6860079 | 0.316052 | 7.6E-05 | 0.0674815 | 3.441709 | 4675 | NAP1L3 | nucleosome assembly protein 1-like 3 |
| 10857 | 3.9118821 | 0.250124 | 4.4E-05 | 0.0674815 | 3.104779 | 6457 | SH3GL3 | SH3-domain GRB2-like 3 |

| **Supplemental Table 9. GO analysis of down-regulated genes in in low-TRIM26-NPC samples relative to high-TRIM26-NPC samples #** | | | | | |
| --- | --- | --- | --- | --- | --- |
|   \|  \| \| --- \| | **GO Term** | **Hit** | **Total** | ***P*-Value** | **Q-Value** |
|  | cluster1       Enrichment Score : 7.21 |  |  |  |  |
|  | [defense response to virus](http://amigo.geneontology.org/cgi-bin/amigo/term_details?term=GO:0051607) | [18](http://ci.smu.edu.cn/GenCLip/hit_GO_genes.php?user=guest&random=2013-11-14_04-39-16_4596&keyword=defense%20response%20to%20virus&genes=10488;148022;25939;3434;3659;3665;4283;4938;55324;5551;60489;6772;79132;81559;8519;91543;958;9636) | 192 | 2.31E-16 | 1.92E-14 |
|  | [response to virus](http://amigo.geneontology.org/cgi-bin/amigo/term_details?term=GO:0009615) | [22](http://ci.smu.edu.cn/GenCLip/hit_GO_genes.php?user=guest&random=2013-11-14_04-39-16_4596&keyword=response%20to%20virus&genes=10488;148022;25939;3430;3434;3659;3665;4283;4938;55324;55509;5551;602;60489;6351;6772;79132;81559;8519;91543;958;9636) | 272 | 2.32E-16 | 9.64E-15 |
|  | [cellular response to type I interferon](http://amigo.geneontology.org/cgi-bin/amigo/term_details?term=GO:0071357) | [10](http://ci.smu.edu.cn/GenCLip/hit_GO_genes.php?user=guest&random=2013-11-14_04-39-16_4596&keyword=cellular%20response%20to%20type%20I%20interferon&genes=3430;3434;3659;3664;3665;4938;5696;6772;8519;9636) | 67 | 5.49E-16 | 1.14E-14 |
|  | [type I interferon-mediated signaling pathway](http://amigo.geneontology.org/cgi-bin/amigo/term_details?term=GO:0060337) | [10](http://ci.smu.edu.cn/GenCLip/hit_GO_genes.php?user=guest&random=2013-11-14_04-39-16_4596&keyword=type%20I%20interferon-mediated%20signaling%20pathway&genes=3430;3434;3659;3664;3665;4938;5696;6772;8519;9636) | 67 | 5.49E-16 | 1.52E-14 |
|  | [response to type I interferon](http://amigo.geneontology.org/cgi-bin/amigo/term_details?term=GO:0034340) | [10](http://ci.smu.edu.cn/GenCLip/hit_GO_genes.php?user=guest&random=2013-11-14_04-39-16_4596&keyword=response%20to%20type%20I%20interferon&genes=3430;3434;3659;3664;3665;4938;5696;6772;8519;9636) | 68 | 1.02E-15 | 1.69E-14 |
|  | [response to other organism](http://amigo.geneontology.org/cgi-bin/amigo/term_details?term=GO:0051707) | [28](http://ci.smu.edu.cn/GenCLip/hit_GO_genes.php?user=guest&random=2013-11-14_04-39-16_4596&keyword=response%20to%20other%20organism&genes=10288;10488;148022;2207;25939;3430;3434;3659;3665;4283;4688;4938;55324;55509;5551;602;60489;613;6351;6772;7133;79132;79155;81559;8519;91543;958;9636) | 593 | 1.76E-09 | 1.22E-08 |
|  | [innate immune response](http://amigo.geneontology.org/cgi-bin/amigo/term_details?term=GO:0045087) | [31](http://ci.smu.edu.cn/GenCLip/hit_GO_genes.php?user=guest&random=2013-11-14_04-39-16_4596&keyword=innate%20immune%20response&genes=10318;10537;11035;11326;148022;2209;2214;25939;3430;3434;3659;3664;3665;3902;4254;4688;4791;4938;5696;60489;6199;6772;712;713;7318;79132;79155;84282;8519;91543;9636) | 722 | 9.40E-09 | 5.57E-08 |
|  | [response to biotic stimulus](http://amigo.geneontology.org/cgi-bin/amigo/term_details?term=GO:0009607) | [28](http://ci.smu.edu.cn/GenCLip/hit_GO_genes.php?user=guest&random=2013-11-14_04-39-16_4596&keyword=response%20to%20biotic%20stimulus&genes=10288;10488;148022;2207;25939;3430;3434;3659;3665;4283;4688;4938;55324;55509;5551;602;60489;613;6351;6772;7133;79132;79155;81559;8519;91543;958;9636) | 622 | 1.01E-08 | 5.25E-08 |
|  | [symbiosis, encompassing mutualism through parasitism](http://amigo.geneontology.org/cgi-bin/amigo/term_details?term=GO:0044403) | [17](http://ci.smu.edu.cn/GenCLip/hit_GO_genes.php?user=guest&random=2013-11-14_04-39-16_4596&keyword=symbiosis,%20encompassing%20mutualism%20through%20parasitism&genes=10318;10488;23352;3065;3431;3434;3665;4688;5696;5699;60489;6351;6772;79132;8519;91543;9636) | 449 | 0.0003 | 0.0006 |
|  | [interspecies interaction between organisms](http://amigo.geneontology.org/cgi-bin/amigo/term_details?term=GO:0044419) | [17](http://ci.smu.edu.cn/GenCLip/hit_GO_genes.php?user=guest&random=2013-11-14_04-39-16_4596&keyword=interspecies%20interaction%20between%20organisms&genes=10318;10488;23352;3065;3431;3434;3665;4688;5696;5699;60489;6351;6772;79132;8519;91543;9636) | 449 | 0.0003 | 0.0006 |
|  | [interaction with host](http://amigo.geneontology.org/cgi-bin/amigo/term_details?term=GO:0051701) | [16](http://ci.smu.edu.cn/GenCLip/hit_GO_genes.php?user=guest&random=2013-11-14_04-39-16_4596&keyword=interaction%20with%20host&genes=10318;10488;23352;3065;3431;3434;3665;4688;5696;5699;60489;6772;79132;8519;91543;9636) | 414 | 0.0003 | 0.0006 |
|  | [multi-organism process](http://amigo.geneontology.org/cgi-bin/amigo/term_details?term=GO:0051704) | [39](http://ci.smu.edu.cn/GenCLip/hit_GO_genes.php?user=guest&random=2013-11-14_04-39-16_4596&keyword=multi-organism%20process&genes=10288;10318;10488;148022;2207;23352;23636;25939;3065;3430;3431;3434;3659;3665;4283;4688;4938;55324;55509;5551;55663;5696;5699;602;60489;613;6351;6772;6881;7133;780;79132;79155;81559;8519;91543;952;958;9636) | 1405 | 0.0005 | 0.0008 |
|  | [virus-host interaction](http://amigo.geneontology.org/cgi-bin/amigo/term_details?term=GO:0019048) | [14](http://ci.smu.edu.cn/GenCLip/hit_GO_genes.php?user=guest&random=2013-11-14_04-39-16_4596&keyword=virus-host%20interaction&genes=10488;23352;3065;3431;3434;3665;5696;5699;60489;6772;79132;8519;91543;9636) | 373 | 0.0011 | 0.0019 |
|  | [regulation of multi-organism process](http://amigo.geneontology.org/cgi-bin/amigo/term_details?term=GO:0043900) | [10](http://ci.smu.edu.cn/GenCLip/hit_GO_genes.php?user=guest&random=2013-11-14_04-39-16_4596&keyword=regulation%20of%20multi-organism%20process&genes=10318;10488;3065;3434;4938;60489;6351;79132;8519;91543) | 216 | 0.0013 | 0.002 |
|  | [viral reproductive process](http://amigo.geneontology.org/cgi-bin/amigo/term_details?term=GO:0022415) | [19](http://ci.smu.edu.cn/GenCLip/hit_GO_genes.php?user=guest&random=2013-11-14_04-39-16_4596&keyword=viral%20reproductive%20process&genes=10318;10488;23352;23636;3065;3431;3434;3665;4938;5696;5699;60489;6351;6772;6881;79132;8519;91543;9636) | 588 | 0.002 | 0.003 |
|  | [viral reproduction](http://amigo.geneontology.org/cgi-bin/amigo/term_details?term=GO:0016032) | [20](http://ci.smu.edu.cn/GenCLip/hit_GO_genes.php?user=guest&random=2013-11-14_04-39-16_4596&keyword=viral%20reproduction&genes=10318;10488;23352;23636;3065;3431;3434;3665;4938;55663;5696;5699;60489;6351;6772;6881;79132;8519;91543;9636) | 699 | 0.0094 | 0.0129 |
|  | [multi-organism cellular process](http://amigo.geneontology.org/cgi-bin/amigo/term_details?term=GO:0044764) | [20](http://ci.smu.edu.cn/GenCLip/hit_GO_genes.php?user=guest&random=2013-11-14_04-39-16_4596&keyword=multi-organism%20cellular%20process&genes=10318;10488;23352;23636;3065;3431;3434;3665;4938;55663;5696;5699;60489;6351;6772;6881;79132;8519;91543;9636) | 701 | 0.0097 | 0.013 |
|  | [multi-organism reproductive process](http://amigo.geneontology.org/cgi-bin/amigo/term_details?term=GO:0044703) | [21](http://ci.smu.edu.cn/GenCLip/hit_GO_genes.php?user=guest&random=2013-11-14_04-39-16_4596&keyword=multi-organism%20reproductive%20process&genes=10318;10488;23352;23636;3065;3431;3434;3665;4938;5696;5699;60489;6351;6772;6881;780;79132;8519;91543;952;9636) | 779 | 0.0174 | 0.0219 |
|  | cluster2       Enrichment Score : 6.91 |  |  |  |  |
|  | [immune response](http://amigo.geneontology.org/cgi-bin/amigo/term_details?term=GO:0006955) | [51](http://ci.smu.edu.cn/GenCLip/hit_GO_genes.php?user=guest&random=2013-11-14_04-39-16_4596&keyword=immune%20response&genes=10148;10288;10318;10537;11035;11326;1445;148022;2067;2207;2209;2214;25865;25939;3430;3434;3659;3664;3665;3902;4254;4283;4688;4791;4938;55072;5551;5696;5699;602;60489;613;6199;6351;6368;6376;6772;7030;712;713;7133;7318;79132;79155;84282;8519;91543;9235;952;958;9636) | 1224 | 4.82E-13 | 6.67E-12 |
|  | [defense response](http://amigo.geneontology.org/cgi-bin/amigo/term_details?term=GO:0006952) | [52](http://ci.smu.edu.cn/GenCLip/hit_GO_genes.php?user=guest&random=2013-11-14_04-39-16_4596&keyword=defense%20response&genes=10288;10318;10488;10537;10866;11025;11035;11326;148022;2207;2209;2214;25939;313;3430;3434;3659;3664;3665;3902;4254;4283;4688;4791;4938;55324;5551;55527;5603;5696;602;60489;613;6199;6351;6368;6376;6772;712;713;7133;7318;79132;79155;81559;84282;84807;8519;91543;9235;958;9636) | 1297 | 2.64E-12 | 3.13E-11 |
|  | [positive regulation of cytokine production](http://amigo.geneontology.org/cgi-bin/amigo/term_details?term=GO:0001819) | [16](http://ci.smu.edu.cn/GenCLip/hit_GO_genes.php?user=guest&random=2013-11-14_04-39-16_4596&keyword=positive%20regulation%20of%20cytokine%20production&genes=10288;10564;11035;148022;2207;25865;3659;3665;4791;5603;602;6376;79132;84282;91543;958) | 208 | 3.06E-11 | 2.83E-10 |
|  | [regulation of cytokine production](http://amigo.geneontology.org/cgi-bin/amigo/term_details?term=GO:0001817) | [22](http://ci.smu.edu.cn/GenCLip/hit_GO_genes.php?user=guest&random=2013-11-14_04-39-16_4596&keyword=regulation%20of%20cytokine%20production&genes=10148;10288;10564;11035;11326;1445;148022;2207;25865;3659;3665;3902;4791;5603;602;6376;7318;79132;84282;91543;958;9636) | 385 | 1.89E-10 | 1.57E-09 |
|  | [regulation of immune system process](http://amigo.geneontology.org/cgi-bin/amigo/term_details?term=GO:0002682) | [38](http://ci.smu.edu.cn/GenCLip/hit_GO_genes.php?user=guest&random=2013-11-14_04-39-16_4596&keyword=regulation%20of%20immune%20system%20process&genes=10148;10288;10318;10488;11006;11025;11035;11326;1445;148022;2207;2209;2214;25865;25939;3257;3434;3659;3665;3902;4254;4791;55072;60489;613;6351;6772;7030;712;713;79132;79155;84282;84807;8519;91543;952;958) | 929 | 1.43E-09 | 1.08E-08 |
|  | [regulation of defense response](http://amigo.geneontology.org/cgi-bin/amigo/term_details?term=GO:0031347) | [23](http://ci.smu.edu.cn/GenCLip/hit_GO_genes.php?user=guest&random=2013-11-14_04-39-16_4596&keyword=regulation%20of%20defense%20response&genes=10318;10488;11035;148022;2207;25939;313;3434;3659;3665;3902;4791;55527;5603;60489;613;6376;6772;7133;79132;79155;84282;91543) | 457 | 6.94E-09 | 4.43E-08 |
|  | [immune system process](http://amigo.geneontology.org/cgi-bin/amigo/term_details?term=GO:0002376) | [62](http://ci.smu.edu.cn/GenCLip/hit_GO_genes.php?user=guest&random=2013-11-14_04-39-16_4596&keyword=immune%20system%20process&genes=10148;10288;10318;10488;10537;11006;11025;11035;11326;1445;148022;2000;2067;2207;2209;2214;25865;25939;3200;3257;3430;3434;3659;3664;3665;3902;4254;4283;4688;4791;4938;55072;55080;55324;55509;5551;5696;5699;602;60489;613;6199;6351;6368;6376;6772;7030;712;713;7133;7318;79132;79155;81559;84282;84807;8519;91543;9235;952;958;9636) | 1940 | 9.55E-09 | 5.28E-08 |
|  | [cytokine production](http://amigo.geneontology.org/cgi-bin/amigo/term_details?term=GO:0001816) | [22](http://ci.smu.edu.cn/GenCLip/hit_GO_genes.php?user=guest&random=2013-11-14_04-39-16_4596&keyword=cytokine%20production&genes=10148;10288;10564;11035;11326;1445;148022;2207;25865;3659;3665;3902;4791;5603;602;6376;7318;79132;84282;91543;958;9636) | 434 | 1.19E-08 | 5.81E-08 |
|  | [regulation of immune response](http://amigo.geneontology.org/cgi-bin/amigo/term_details?term=GO:0050776) | [27](http://ci.smu.edu.cn/GenCLip/hit_GO_genes.php?user=guest&random=2013-11-14_04-39-16_4596&keyword=regulation%20of%20immune%20response&genes=10288;10318;11035;11326;1445;148022;2207;2209;2214;25865;25939;3659;3665;3902;4791;55072;613;6772;712;713;79132;79155;84282;8519;91543;952;958) | 606 | 2.69E-08 | 1.24E-07 |
|  | [immune response-regulating signaling pathway](http://amigo.geneontology.org/cgi-bin/amigo/term_details?term=GO:0002764) | [18](http://ci.smu.edu.cn/GenCLip/hit_GO_genes.php?user=guest&random=2013-11-14_04-39-16_4596&keyword=immune%20response-regulating%20signaling%20pathway&genes=10288;10318;11035;1445;148022;2207;2209;2214;25865;3659;3665;4791;55072;79132;79155;91543;952;958) | 336 | 5.91E-08 | 2.58E-07 |
|  | [activation of immune response](http://amigo.geneontology.org/cgi-bin/amigo/term_details?term=GO:0002253) | [19](http://ci.smu.edu.cn/GenCLip/hit_GO_genes.php?user=guest&random=2013-11-14_04-39-16_4596&keyword=activation%20of%20immune%20response&genes=10318;11035;11326;1445;148022;2207;2209;2214;25865;3659;3665;4791;55072;712;713;79132;79155;91543;952) | 374 | 1.16E-07 | 4.81E-07 |
|  | [positive regulation of immune system process](http://amigo.geneontology.org/cgi-bin/amigo/term_details?term=GO:0002684) | [26](http://ci.smu.edu.cn/GenCLip/hit_GO_genes.php?user=guest&random=2013-11-14_04-39-16_4596&keyword=positive%20regulation%20of%20immune%20system%20process&genes=10148;10288;10318;10488;11035;11326;1445;148022;2207;2209;2214;25865;3257;3659;3665;3902;4791;55072;6351;712;713;79132;79155;91543;952;958) | 607 | 1.74E-07 | 6.87E-07 |
|  | [positive regulation of immune response](http://amigo.geneontology.org/cgi-bin/amigo/term_details?term=GO:0050778) | [20](http://ci.smu.edu.cn/GenCLip/hit_GO_genes.php?user=guest&random=2013-11-14_04-39-16_4596&keyword=positive%20regulation%20of%20immune%20response&genes=10318;11035;11326;1445;148022;2207;2209;2214;25865;3659;3665;3902;4791;55072;712;713;79132;79155;91543;952) | 439 | 1.08E-06 | 3.90E-06 |
|  | [immune response-activating signal transduction](http://amigo.geneontology.org/cgi-bin/amigo/term_details?term=GO:0002757) | [16](http://ci.smu.edu.cn/GenCLip/hit_GO_genes.php?user=guest&random=2013-11-14_04-39-16_4596&keyword=immune%20response-activating%20signal%20transduction&genes=10318;11035;1445;148022;2207;2209;2214;25865;3659;3665;4791;55072;79132;79155;91543;952) | 324 | 2.33E-06 | 7.17E-06 |
|  | [regulation of innate immune response](http://amigo.geneontology.org/cgi-bin/amigo/term_details?term=GO:0045088) | [13](http://ci.smu.edu.cn/GenCLip/hit_GO_genes.php?user=guest&random=2013-11-14_04-39-16_4596&keyword=regulation%20of%20innate%20immune%20response&genes=10318;11035;148022;25939;3659;3665;3902;4791;6772;79132;79155;84282;91543) | 254 | 3.31E-05 | 9.16E-05 |
|  | [positive regulation of defense response](http://amigo.geneontology.org/cgi-bin/amigo/term_details?term=GO:0031349) | [13](http://ci.smu.edu.cn/GenCLip/hit_GO_genes.php?user=guest&random=2013-11-14_04-39-16_4596&keyword=positive%20regulation%20of%20defense%20response&genes=10318;11035;148022;2207;3659;3665;3902;4791;5603;6376;79132;79155;91543) | 258 | 4.37E-05 | 0.0001 |
|  | [regulation of response to stress](http://amigo.geneontology.org/cgi-bin/amigo/term_details?term=GO:0080134) | [25](http://ci.smu.edu.cn/GenCLip/hit_GO_genes.php?user=guest&random=2013-11-14_04-39-16_4596&keyword=regulation%20of%20response%20to%20stress&genes=10318;10488;11035;148022;2207;25939;2814;313;3434;3659;3665;3902;4255;4791;55527;5603;60489;613;6376;6772;7133;79132;79155;84282;91543) | 769 | 0.0003 | 0.0006 |
|  | [positive regulation of response to stimulus](http://amigo.geneontology.org/cgi-bin/amigo/term_details?term=GO:0048584) | [37](http://ci.smu.edu.cn/GenCLip/hit_GO_genes.php?user=guest&random=2013-11-14_04-39-16_4596&keyword=positive%20regulation%20of%20response%20to%20stimulus&genes=10045;10318;10488;10537;11035;11326;124583;1445;148022;2207;2209;2214;23636;25865;2889;3659;3665;3902;3965;4254;4255;429;4791;55072;5603;6351;6376;6794;712;713;79132;79155;79370;91543;952;958;9982) | 1296 | 0.0003 | 0.0006 |
|  | [regulation of response to stimulus](http://amigo.geneontology.org/cgi-bin/amigo/term_details?term=GO:0048583) | [61](http://ci.smu.edu.cn/GenCLip/hit_GO_genes.php?user=guest&random=2013-11-14_04-39-16_4596&keyword=regulation%20of%20response%20to%20stimulus&genes=10045;10288;10318;10488;10537;10564;10974;11035;11326;116985;124583;1445;148022;2207;2209;2214;23061;23625;23636;23654;25865;25939;27128;2814;2889;3065;313;3434;3659;3665;3902;391;3965;4254;4255;429;4791;55072;55527;5603;602;60489;613;6351;6376;6772;6794;712;713;7133;79132;79155;79370;84282;84807;8519;8729;91543;952;958;9982) | 2526 | 0.0009 | 0.0015 |
|  | [positive regulation of multicellular organismal process](http://amigo.geneontology.org/cgi-bin/amigo/term_details?term=GO:0051240) | [17](http://ci.smu.edu.cn/GenCLip/hit_GO_genes.php?user=guest&random=2013-11-14_04-39-16_4596&keyword=positive%20regulation%20of%20multicellular%20organismal%20process&genes=10288;10564;11035;148022;2207;25865;3659;3665;4791;5603;602;6376;79132;84282;91543;952;958) | 546 | 0.0058 | 0.0085 |
|  | cluster3       Enrichment Score : 5.47 |  |  |  |  |
|  | [immune effector process](http://amigo.geneontology.org/cgi-bin/amigo/term_details?term=GO:0002252) | [28](http://ci.smu.edu.cn/GenCLip/hit_GO_genes.php?user=guest&random=2013-11-14_04-39-16_4596&keyword=immune%20effector%20process&genes=10488;11326;148022;2067;2207;2209;2214;25939;3434;3659;3665;3902;4283;4938;55324;5551;602;60489;613;6772;712;713;79132;81559;8519;91543;958;9636) | 529 | 1.76E-11 | 1.83E-10 |
|  | [adaptive immune response](http://amigo.geneontology.org/cgi-bin/amigo/term_details?term=GO:0002250) | [11](http://ci.smu.edu.cn/GenCLip/hit_GO_genes.php?user=guest&random=2013-11-14_04-39-16_4596&keyword=adaptive%20immune%20response&genes=10148;2067;2207;3659;3665;4791;602;712;713;91543;958) | 199 | 4.80E-05 | 0.0001 |
|  | [adaptive immune response based on somatic recombination of immune receptors built from immunoglobulin superfamily domains](http://amigo.geneontology.org/cgi-bin/amigo/term_details?term=GO:0002460) | [10](http://ci.smu.edu.cn/GenCLip/hit_GO_genes.php?user=guest&random=2013-11-14_04-39-16_4596&keyword=adaptive%20immune%20response%20based%20on%20somatic%20recombination%20of%20immune%20receptors%20built%20from%20immunoglobulin%20superfamily%20domains&genes=10148;2067;2207;3665;4791;602;712;713;91543;958) | 181 | 0.0001 | 0.0003 |
|   \|  \| \| --- \| | [leukocyte mediated immunity](http://amigo.geneontology.org/cgi-bin/amigo/term_details?term=GO:0002443) | [10](http://ci.smu.edu.cn/GenCLip/hit_GO_genes.php?user=guest&random=2013-11-14_04-39-16_4596&keyword=leukocyte%20mediated%20immunity&genes=2067;2207;3665;3902;602;613;712;713;91543;958) | 215 | 0.0012 | 0.002 |
|  | cluster4       Enrichment Score : 5.15 |  |  |  |  |
|  | [cytokine-mediated signaling pathway](http://amigo.geneontology.org/cgi-bin/amigo/term_details?term=GO:0019221) | [18](http://ci.smu.edu.cn/GenCLip/hit_GO_genes.php?user=guest&random=2013-11-14_04-39-16_4596&keyword=cytokine-mediated%20signaling%20pathway&genes=10148;2209;23636;3430;3434;3587;3659;3664;3665;4938;5696;6376;6772;7133;7318;79155;8519;9636) | 355 | 2.66E-07 | 1.00E-06 |
|  | [cellular response to cytokine stimulus](http://amigo.geneontology.org/cgi-bin/amigo/term_details?term=GO:0071345) | [18](http://ci.smu.edu.cn/GenCLip/hit_GO_genes.php?user=guest&random=2013-11-14_04-39-16_4596&keyword=cellular%20response%20to%20cytokine%20stimulus&genes=10148;2209;23636;3430;3434;3587;3659;3664;3665;4938;5696;6376;6772;7133;7318;79155;8519;9636) | 431 | 2.64E-05 | 7.56E-05 |
|  | [response to cytokine stimulus](http://amigo.geneontology.org/cgi-bin/amigo/term_details?term=GO:0034097) | [20](http://ci.smu.edu.cn/GenCLip/hit_GO_genes.php?user=guest&random=2013-11-14_04-39-16_4596&keyword=response%20to%20cytokine%20stimulus&genes=10148;10537;2209;23636;3430;3434;3587;3659;3664;3665;4938;5696;6376;6772;7133;7318;79155;8519;952;9636) | 517 | 5.02E-05 | 0.0001 |
|  | cluster5       Enrichment Score : 3.55 |  |  |  |  |
|  | [leukocyte activation](http://amigo.geneontology.org/cgi-bin/amigo/term_details?term=GO:0045321) | [22](http://ci.smu.edu.cn/GenCLip/hit_GO_genes.php?user=guest&random=2013-11-14_04-39-16_4596&keyword=leukocyte%20activation&genes=10148;10288;10537;11326;1445;148022;2000;2067;2207;3257;3659;3902;55509;5699;602;613;6376;79155;84807;91543;952;958) | 548 | 8.50E-06 | 2.52E-05 |
|  | [regulation of cell activation](http://amigo.geneontology.org/cgi-bin/amigo/term_details?term=GO:0050865) | [15](http://ci.smu.edu.cn/GenCLip/hit_GO_genes.php?user=guest&random=2013-11-14_04-39-16_4596&keyword=regulation%20of%20cell%20activation&genes=10148;10288;11326;1445;148022;2207;2814;3257;3659;3902;613;79155;84807;952;958) | 350 | 8.13E-05 | 0.0002 |
|  | [regulation of leukocyte activation](http://amigo.geneontology.org/cgi-bin/amigo/term_details?term=GO:0002694) | [14](http://ci.smu.edu.cn/GenCLip/hit_GO_genes.php?user=guest&random=2013-11-14_04-39-16_4596&keyword=regulation%20of%20leukocyte%20activation&genes=10148;10288;11326;1445;148022;2207;3257;3659;3902;613;79155;84807;952;958) | 325 | 0.0001 | 0.0003 |
|  | [cell activation](http://amigo.geneontology.org/cgi-bin/amigo/term_details?term=GO:0001775) | [25](http://ci.smu.edu.cn/GenCLip/hit_GO_genes.php?user=guest&random=2013-11-14_04-39-16_4596&keyword=cell%20activation&genes=10148;10288;10537;11326;1445;148022;2000;2067;2207;2814;3257;3659;3902;391;4791;55509;5699;602;613;6376;79155;84807;91543;952;958) | 755 | 0.0002 | 0.0005 |
|  | [leukocyte proliferation](http://amigo.geneontology.org/cgi-bin/amigo/term_details?term=GO:0070661) | [10](http://ci.smu.edu.cn/GenCLip/hit_GO_genes.php?user=guest&random=2013-11-14_04-39-16_4596&keyword=leukocyte%20proliferation&genes=10148;10288;11326;148022;2000;3659;4254;5699;952;958) | 194 | 0.0003 | 0.0006 |
|  | [lymphocyte activation](http://amigo.geneontology.org/cgi-bin/amigo/term_details?term=GO:0046649) | [17](http://ci.smu.edu.cn/GenCLip/hit_GO_genes.php?user=guest&random=2013-11-14_04-39-16_4596&keyword=lymphocyte%20activation&genes=10148;10288;11326;1445;148022;2000;2067;3257;3659;3902;5699;602;79155;84807;91543;952;958) | 460 | 0.0004 | 0.0008 |
|  | [regulation of lymphocyte activation](http://amigo.geneontology.org/cgi-bin/amigo/term_details?term=GO:0051249) | [12](http://ci.smu.edu.cn/GenCLip/hit_GO_genes.php?user=guest&random=2013-11-14_04-39-16_4596&keyword=regulation%20of%20lymphocyte%20activation&genes=10148;10288;11326;1445;148022;3257;3659;3902;79155;84807;952;958) | 284 | 0.0013 | 0.002 |
|  | [T cell activation](http://amigo.geneontology.org/cgi-bin/amigo/term_details?term=GO:0042110) | [11](http://ci.smu.edu.cn/GenCLip/hit_GO_genes.php?user=guest&random=2013-11-14_04-39-16_4596&keyword=T%20cell%20activation&genes=10148;10288;11326;1445;2000;3659;3902;5699;602;84807;91543) | 325 | 0.0123 | 0.016 |
|  | cluster6       Enrichment Score : 2.95 |  |  |  |  |
|  | [I-kappaB kinase/NF-kappaB cascade](http://amigo.geneontology.org/cgi-bin/amigo/term_details?term=GO:0007249) | [13](http://ci.smu.edu.cn/GenCLip/hit_GO_genes.php?user=guest&random=2013-11-14_04-39-16_4596&keyword=I-kappaB%20kinase/NF-kappaB%20cascade&genes=10318;10537;11035;124583;148022;23636;3965;55072;602;6772;79155;84807;958) | 217 | 1.49E-06 | 5.16E-06 |
|  | [positive regulation of NF-kappaB transcription factor activity](http://amigo.geneontology.org/cgi-bin/amigo/term_details?term=GO:0051092) | [15](http://ci.smu.edu.cn/GenCLip/hit_GO_genes.php?user=guest&random=2013-11-14_04-39-16_4596&keyword=positive%20regulation%20of%20NF-kappaB%20transcription%20factor%20activity&genes=10318;10537;11035;124583;148022;23636;25865;3965;4791;55072;602;6772;79155;84807;958) | 274 | 1.58E-06 | 5.24E-06 |
|  | [regulation of I-kappaB kinase/NF-kappaB cascade](http://amigo.geneontology.org/cgi-bin/amigo/term_details?term=GO:0043122) | [12](http://ci.smu.edu.cn/GenCLip/hit_GO_genes.php?user=guest&random=2013-11-14_04-39-16_4596&keyword=regulation%20of%20I-kappaB%20kinase/NF-kappaB%20cascade&genes=10318;10537;124583;148022;23636;3965;55072;602;6772;79155;84807;958) | 194 | 2.23E-06 | 7.11E-06 |
|  | [positive regulation of sequence-specific DNA binding transcription factor activity](http://amigo.geneontology.org/cgi-bin/amigo/term_details?term=GO:0051091) | [15](http://ci.smu.edu.cn/GenCLip/hit_GO_genes.php?user=guest&random=2013-11-14_04-39-16_4596&keyword=positive%20regulation%20of%20sequence-specific%20DNA%20binding%20transcription%20factor%20activity&genes=10318;10537;11035;124583;148022;23636;25865;3965;4791;55072;602;6772;79155;84807;958) | 347 | 6.95E-05 | 0.0002 |
|  | [regulation of sequence-specific DNA binding transcription factor activity](http://amigo.geneontology.org/cgi-bin/amigo/term_details?term=GO:0051090) | [15](http://ci.smu.edu.cn/GenCLip/hit_GO_genes.php?user=guest&random=2013-11-14_04-39-16_4596&keyword=regulation%20of%20sequence-specific%20DNA%20binding%20transcription%20factor%20activity&genes=10318;10537;11035;124583;148022;23636;25865;3965;4791;55072;602;6772;79155;84807;958) | 489 | 0.0116 | 0.0153 |
|  | [sequence-specific DNA binding transcription factor activity](http://amigo.geneontology.org/cgi-bin/amigo/term_details?term=GO:0003700) | [33](http://ci.smu.edu.cn/GenCLip/hit_GO_genes.php?user=guest&random=2013-11-14_04-39-16_4596&keyword=sequence-specific%20DNA%20binding%20transcription%20factor%20activity&genes=10318;10488;10537;10943;11035;124583;148022;1870;2000;22921;23636;25865;3065;3200;3659;3664;3665;3965;429;4791;51513;55072;55502;55509;55663;602;6772;6881;7030;79155;84807;8625;958) | 1402 | 0.0278 | 0.0329 |
|  | [nucleic acid binding transcription factor activity](http://amigo.geneontology.org/cgi-bin/amigo/term_details?term=GO:0001071) | [33](http://ci.smu.edu.cn/GenCLip/hit_GO_genes.php?user=guest&random=2013-11-14_04-39-16_4596&keyword=nucleic%20acid%20binding%20transcription%20factor%20activity&genes=10318;10488;10537;10943;11035;124583;148022;1870;2000;22921;23636;25865;3065;3200;3659;3664;3665;3965;429;4791;51513;55072;55502;55509;55663;602;6772;6881;7030;79155;84807;8625;958) | 1404 | 0.0284 | 0.0332 |
|  | [transcription regulatory region sequence-specific DNA binding](http://amigo.geneontology.org/cgi-bin/amigo/term_details?term=GO:0000976) | [33](http://ci.smu.edu.cn/GenCLip/hit_GO_genes.php?user=guest&random=2013-11-14_04-39-16_4596&keyword=transcription%20regulatory%20region%20sequence-specific%20DNA%20binding&genes=10318;10488;10537;10943;11035;124583;148022;1870;2000;22921;23636;25865;3065;3200;3659;3664;3665;3965;429;4791;51513;55072;55502;55509;55663;602;6772;6881;7030;79155;84807;8625;958) | 1411 | 0.0306 | 0.0348 |
|  | [regulatory region nucleic acid binding](http://amigo.geneontology.org/cgi-bin/amigo/term_details?term=GO:0001067) | [34](http://ci.smu.edu.cn/GenCLip/hit_GO_genes.php?user=guest&random=2013-11-14_04-39-16_4596&keyword=regulatory%20region%20nucleic%20acid%20binding&genes=10318;10488;10537;10943;11035;124583;130557;148022;1870;2000;22921;23636;25865;3065;3200;3659;3664;3665;3965;429;4791;51513;55072;55502;55509;55663;602;6772;6881;7030;79155;84807;8625;958) | 1465 | 0.0315 | 0.0349 |
|  | [regulatory region DNA binding](http://amigo.geneontology.org/cgi-bin/amigo/term_details?term=GO:0000975) | [34](http://ci.smu.edu.cn/GenCLip/hit_GO_genes.php?user=guest&random=2013-11-14_04-39-16_4596&keyword=regulatory%20region%20DNA%20binding&genes=10318;10488;10537;10943;11035;124583;130557;148022;1870;2000;22921;23636;25865;3065;3200;3659;3664;3665;3965;429;4791;51513;55072;55502;55509;55663;602;6772;6881;7030;79155;84807;8625;958) | 1465 | 0.0315 | 0.0345 |
|  | [transcription regulatory region DNA binding](http://amigo.geneontology.org/cgi-bin/amigo/term_details?term=GO:0044212) | [34](http://ci.smu.edu.cn/GenCLip/hit_GO_genes.php?user=guest&random=2013-11-14_04-39-16_4596&keyword=transcription%20regulatory%20region%20DNA%20binding&genes=10318;10488;10537;10943;11035;124583;130557;148022;1870;2000;22921;23636;25865;3065;3200;3659;3664;3665;3965;429;4791;51513;55072;55502;55509;55663;602;6772;6881;7030;79155;84807;8625;958) | 1465 | 0.0315 | 0.034 |
|  | cluster7       Enrichment Score : 2.01 |  |  |  |  |
|  | [protein modification by small protein conjugation](http://amigo.geneontology.org/cgi-bin/amigo/term_details?term=GO:0032446) | [19](http://ci.smu.edu.cn/GenCLip/hit_GO_genes.php?user=guest&random=2013-11-14_04-39-16_4596&keyword=protein%20modification%20by%20small%20protein%20conjugation&genes=10401;10537;119504;148022;153769;23352;26270;3300;55008;55072;55527;5696;5699;65264;7318;81559;81603;84282;9817) | 630 | 0.0055 | 0.0082 |
|  | [protein ubiquitination](http://amigo.geneontology.org/cgi-bin/amigo/term_details?term=GO:0016567) | [18](http://ci.smu.edu.cn/GenCLip/hit_GO_genes.php?user=guest&random=2013-11-14_04-39-16_4596&keyword=protein%20ubiquitination&genes=10537;119504;148022;153769;23352;26270;3300;55008;55072;55527;5696;5699;65264;7318;81559;81603;84282;9817) | 595 | 0.0067 | 0.0096 |
|  | [protein modification by small protein conjugation or removal](http://amigo.geneontology.org/cgi-bin/amigo/term_details?term=GO:0070647) | [20](http://ci.smu.edu.cn/GenCLip/hit_GO_genes.php?user=guest&random=2013-11-14_04-39-16_4596&keyword=protein%20modification%20by%20small%20protein%20conjugation%20or%20removal&genes=10401;10537;119504;148022;153769;23352;26270;3300;55008;55072;55527;5696;5699;65264;6881;7318;81559;81603;84282;9817) | 697 | 0.009 | 0.0126 |
|  | [small conjugating protein ligase activity](http://amigo.geneontology.org/cgi-bin/amigo/term_details?term=GO:0019787) | [11](http://ci.smu.edu.cn/GenCLip/hit_GO_genes.php?user=guest&random=2013-11-14_04-39-16_4596&keyword=small%20conjugating%20protein%20ligase%20activity&genes=10401;23352;26270;55008;55072;55527;5696;5699;65264;81559;84282) | 352 | 0.0265 | 0.0319 |
|  | cluster8       Enrichment Score : 1.75 |  |  |  |  |
|  | [Ras protein signal transduction](http://amigo.geneontology.org/cgi-bin/amigo/term_details?term=GO:0007265) | [14](http://ci.smu.edu.cn/GenCLip/hit_GO_genes.php?user=guest&random=2013-11-14_04-39-16_4596&keyword=Ras%20protein%20signal%20transduction&genes=10564;116985;23061;23636;23654;27128;391;4254;5603;613;79930;8625;8729;958) | 439 | 0.0096 | 0.013 |
|  | [regulation of Ras protein signal transduction](http://amigo.geneontology.org/cgi-bin/amigo/term_details?term=GO:0046578) | [11](http://ci.smu.edu.cn/GenCLip/hit_GO_genes.php?user=guest&random=2013-11-14_04-39-16_4596&keyword=regulation%20of%20Ras%20protein%20signal%20transduction&genes=10564;116985;23061;23636;23654;27128;391;4254;613;8729;958) | 339 | 0.0187 | 0.0231 |
|  | [small GTPase mediated signal transduction](http://amigo.geneontology.org/cgi-bin/amigo/term_details?term=GO:0007264) | [18](http://ci.smu.edu.cn/GenCLip/hit_GO_genes.php?user=guest&random=2013-11-14_04-39-16_4596&keyword=small%20GTPase%20mediated%20signal%20transduction&genes=10045;10564;116985;23061;23636;23654;27128;2889;326624;381;391;4254;5603;613;79930;8625;8729;958) | 676 | 0.0324 | 0.0341 |
|  | cluster9       Enrichment Score : 1.74 |  |  |  |  |
|  | [leukocyte differentiation](http://amigo.geneontology.org/cgi-bin/amigo/term_details?term=GO:0002521) | [12](http://ci.smu.edu.cn/GenCLip/hit_GO_genes.php?user=guest&random=2013-11-14_04-39-16_4596&keyword=leukocyte%20differentiation&genes=10288;10537;11006;11025;3659;3665;4254;55509;602;7030;84807;91543) | 337 | 0.005 | 0.0076 |
|  | [immune system development](http://amigo.geneontology.org/cgi-bin/amigo/term_details?term=GO:0002520) | [16](http://ci.smu.edu.cn/GenCLip/hit_GO_genes.php?user=guest&random=2013-11-14_04-39-16_4596&keyword=immune%20system%20development&genes=10288;10537;11006;11025;2067;3200;3659;3665;4254;4791;55509;602;7030;84807;91543;958) | 580 | 0.0307 | 0.0344 |
|  | [hematopoietic or lymphoid organ development](http://amigo.geneontology.org/cgi-bin/amigo/term_details?term=GO:0048534) | [15](http://ci.smu.edu.cn/GenCLip/hit_GO_genes.php?user=guest&random=2013-11-14_04-39-16_4596&keyword=hematopoietic%20or%20lymphoid%20organ%20development&genes=10288;10537;11006;11025;2067;3200;3659;3665;4254;4791;55509;602;7030;84807;91543) | 549 | 0.0403 | 0.0413 |
|  | cluster10       Enrichment Score : 1.70 |  |  |  |  |
|  | [inflammatory response](http://amigo.geneontology.org/cgi-bin/amigo/term_details?term=GO:0006954) | [15](http://ci.smu.edu.cn/GenCLip/hit_GO_genes.php?user=guest&random=2013-11-14_04-39-16_4596&keyword=inflammatory%20response&genes=10318;148022;2207;313;4283;55527;5603;613;6351;6368;6376;7133;79155;84807;958) | 501 | 0.0153 | 0.0196 |
|  | [regulation of response to external stimulus](http://amigo.geneontology.org/cgi-bin/amigo/term_details?term=GO:0032101) | [12](http://ci.smu.edu.cn/GenCLip/hit_GO_genes.php?user=guest&random=2013-11-14_04-39-16_4596&keyword=regulation%20of%20response%20to%20external%20stimulus&genes=10318;10488;2207;25865;2814;313;55527;5603;613;6351;6376;7133) | 393 | 0.0255 | 0.0311 |
|  | cluster11       Enrichment Score : 1.46 |  |  |  |  |
|  | [histone modification](http://amigo.geneontology.org/cgi-bin/amigo/term_details?term=GO:0016570) | [10](http://ci.smu.edu.cn/GenCLip/hit_GO_genes.php?user=guest&random=2013-11-14_04-39-16_4596&keyword=histone%20modification&genes=10445;10865;10943;112869;2139;22933;25865;3065;6881;79903) | 317 | 0.0319 | 0.034 |
|  | [covalent chromatin modification](http://amigo.geneontology.org/cgi-bin/amigo/term_details?term=GO:0016569) | [10](http://ci.smu.edu.cn/GenCLip/hit_GO_genes.php?user=guest&random=2013-11-14_04-39-16_4596&keyword=covalent%20chromatin%20modification&genes=10445;10865;10943;112869;2139;22933;25865;3065;6881;79903) | 323 | 0.0373 | 0.0387 |
|  | cluster12       Enrichment Score : 1.33 |  |  |  |  |
|  | [positive regulation of macromolecule biosynthetic process](http://amigo.geneontology.org/cgi-bin/amigo/term_details?term=GO:0010557) | [29](http://ci.smu.edu.cn/GenCLip/hit_GO_genes.php?user=guest&random=2013-11-14_04-39-16_4596&keyword=positive%20regulation%20of%20macromolecule%20biosynthetic%20process&genes=10148;10318;10488;10865;10974;148022;2000;23636;25865;26206;29946;3065;3659;3664;3665;391;4254;429;602;6199;6772;7030;79155;84305;85441;8625;91875;952;958) | 1244 | 0.0458 | 0.0464 |
|  | [positive regulation of transcription from RNA polymerase II promoter](http://amigo.geneontology.org/cgi-bin/amigo/term_details?term=GO:0045944) | [19](http://ci.smu.edu.cn/GenCLip/hit_GO_genes.php?user=guest&random=2013-11-14_04-39-16_4596&keyword=positive%20regulation%20of%20transcription%20from%20RNA%20polymerase%20II%20promoter&genes=10318;10488;10865;10974;2000;25865;26206;3065;3659;3665;429;602;6772;7030;79155;85441;8625;91875;958) | 749 | 0.0478 | 0.0478 |
|  | nocluster |  |  |  |  |
|  | [response to stress](http://amigo.geneontology.org/cgi-bin/amigo/term_details?term=GO:0006950) | [75](http://ci.smu.edu.cn/GenCLip/hit_GO_genes.php?user=guest&random=2013-11-14_04-39-16_4596&keyword=response%20to%20stress&genes=10045;10288;10318;10445;10488;10537;10866;11025;11035;11284;11326;1445;148022;2067;2139;2207;2209;2214;25939;26270;2814;3065;313;3257;3300;3310;3430;3434;3659;3664;3665;3902;391;4254;4255;4283;4688;4791;4938;55324;5551;55527;5603;5696;5699;602;60489;613;6199;6351;6368;6376;6772;6794;712;713;7133;7318;780;79132;79155;79803;81559;83605;84282;84807;8519;91543;91875;9235;93100;952;958;9636;9973) | 3066 | 0.0001 | 0.0002 |
|  | [positive regulation of innate immune response](http://amigo.geneontology.org/cgi-bin/amigo/term_details?term=GO:0045089) | [10](http://ci.smu.edu.cn/GenCLip/hit_GO_genes.php?user=guest&random=2013-11-14_04-39-16_4596&keyword=positive%20regulation%20of%20innate%20immune%20response&genes=10318;11035;148022;3659;3665;3902;4791;79132;79155;91543) | 197 | 0.0004 | 0.0007 |
|  | [negative regulation of cell proliferation](http://amigo.geneontology.org/cgi-bin/amigo/term_details?term=GO:0008285) | [15](http://ci.smu.edu.cn/GenCLip/hit_GO_genes.php?user=guest&random=2013-11-14_04-39-16_4596&keyword=negative%20regulation%20of%20cell%20proliferation&genes=10288;11326;1445;23087;23636;3300;3659;3664;6368;6772;6794;780;84875;8519;9982) | 532 | 0.0293 | 0.0338 |
|  | | | | | |
|  | # Of 329 genes, 278 genes have GO annotations. |  |  |  |  |

| **Supplemental Table 12. The summary of samples analyzed in targeted sequencing and replication study** | | | | | | | | |  |  |  |
| --- | --- | --- | --- | --- | --- | --- | --- | --- | --- | --- | --- |
|  |  | Targeted-seq |  | Replication study | | | | | | | |
| Characteristics |  | Cases |  | Stage 1* | |  | Stage 2 | |  | Combined | |
|  |  |  |  | Case | Control |  | Case | Control |  | Case | Control |
| Sample size (n) |  | 40 |  | 297 | 611 |  | 768 | 1526 |  | 1065 | 2137 |
| Mean age (s.d) |  | 44.79(2.172) |  | 48.20(11.53) | 39.69(8.01) |  | 45.28(11.0) | 48.05(17.44) |  | 46.38(11.27) | 43.53(18.19) |
| Male |  | 30 (75%) |  | 232(78.1%) | 446(73.0%) |  | 538(70%) | 1,028(67.35%) |  | 771(72.4%) | 1,477(69.1%) |
| Female |  | 10 (25%) |  | 65(21.9%) | 165(27.0%) |  | 230(30%) | 498(32.6%) |  | 294(27.6%) | 660(30.9%) |
| * 310 cases and 618 controls were firstly collected at this stage, but 297 cases and 611 controls were successfully genotyped for association analysis. | | | | | | | | | | | |

| **Supplemental Table 13. The comparison of data production between targeted sequencing and exome-Seq of the MHC region** | | | | | |
| --- | --- | --- | --- | --- | --- |
|  | Targeted MHC Sequencing |  | Exome sequencing^a^ | | |
|  | Mean±SD (40 NPC samples) |  | Sample A ^c^ | Sample B ^c^ | Sample C ^c^ |
| Mean depth (X)^b^ | 40.33±12.87 |  | 5.89164591 | 5.73096803 | 4.57723871 |
| Coverage (%) ^b^ | 96.2±1.2 |  | 29.3305222 | 29.6232001 | 28.7560219 |
| a Capture method: Agilent Sureselect all exons 50M . Mean Depth on exome: 70~80X. Coverage on exome: ~98%. | | | | | |
| b Mean depth and Coverage are evaluated by the Non-repetitive 2.31M MHC region. | | | | |  |
| c 3 NPC tissue samples. | |  |  |  |  |

| **Supplemental Table 14. Primers used for RT-PCR** | |  |
| --- | --- | --- |
| Gene symbol | Forward primer | Reverse primer |
| *TRIM26* | 5'-AGGGTGCGGTTGCTTCATTC-3' | 5'-TCCTTGGAGACGCGACATAG-3' |
| NFKB2 | 5'-AGAGGCTTCCGATTTCGATATGG-3' | 5'-GGATAGGTCTTTCGGCCCTTC-3' |
| IL-32 | 5'-TGGCGGCTTATTATGAGGAGC-3' | 5'-CTCGGCACCGTAATCCATCTC-3' |
| IRF7 | 5'-GCTGGACGTGACCATCATGTA-3' | 5'-GGGCCGTATAGGAACGTGC-3' |
| CD38 | 5'-GGCCCATCAGTTCACACAGG-3' | 5'-GGTCATCAGCAAGGTAGCCTA-3' |
| STAT1 | 5'-CAGCTTGACTCAAAATTCCTGGA-3' | 5'-TGAAGATTACGCTTGCTTTTCCT-3' |
