# Supplementary material for: A regulatory mutant on TRIM26 conferring the risk of nasopharyngeal carcinoma by inducing low immune response
Source: Cancer Med. 2018 Jun 28;7(8):3848–61. doi: 10.1002/cam4.1537 (PMC6089173; doi:10.1002/cam4.1537)
Supplement: Supplementary file 3 [file CAM4-7-3848-s003.docx]

Supplemental Table 10. Differentially expressed genes between low-Trim26-NPC and NP samples.

| Row | d.value | stdev | rawp | q.value | R.fold | oligo_id | SYMBOL | GENENAME | LocusLink |
| --- | --- | --- | --- | --- | --- | --- | --- | --- | --- |
| 9409 | -2.1653 | 40.72247 | 9.56E-03 | 0.034474 | 0.016377 | H200012923 | LRRC46 | leucine rich repeat containing 46 | 90506 |
| 12454 | -3.0567 | 91.9583 | 8.65E-04 | 0.010492 | 0.043391 | H200005578 | BPIFB1 | BPI fold containing family B, member 1 | 92747 |
| 14745 | -1.9256 | 17.02894 | 1.89E-02 | 0.049965 | 0.043396 | H200005796 | SPEF1 | sperm flagellar 1 | 25876 |
| 4818 | -2.4604 | 39.43199 | 4.14E-03 | 0.022069 | 0.04354 | H200011897 | CXCR5 | chemokine (C-X-C motif) receptor 5 | 643 |
| 11896 | -4.0288 | 8.559382 | 1.10E-04 | 0.005439 | 0.046356 | H200000567 | BLK | B lymphoid tyrosine kinase | 640 |
| 7130 | -3.6183 | 16.2094 | 2.38E-04 | 0.006669 | 0.048876 | H200013227 | PLD4 | phospholipase D family, member 4 | 122618 |
| 12809 | -2.5006 | 11.55203 | 3.71E-03 | 0.020795 | 0.051638 | H200000762 | KRT4 | keratin 4 | 3851 |
| 3503 | -2.6889 | 58.89999 | 2.23E-03 | 0.016186 | 0.05382 | H200014434 | PLA2G2D | phospholipase A2, group IID | 26279 |
| 5258 | -2.0606 | 71.19904 | 1.29E-02 | 0.040197 | 0.0571 | H200011232 | SMC5 | structural maintenance of chromosomes 5 | 23137 |
| 3473 | -3.5912 | 8.727879 | 2.52E-04 | 0.006809 | 0.058575 | H200013222 | VPREB3 | pre-B lymphocyte 3 | 29802 |
| 11288 | -1.786 | 43.38553 | 2.82E-02 | 0.062912 | 0.05886 | H200014995 | PIGR | polymeric immunoglobulin receptor | 5284 |
| 11438 | -3.2568 | 17.3806 | 5.40E-04 | 0.008913 | 0.060488 | H200000626 | TCL1A | T-cell leukemia/lymphoma 1A | 8115 |
| 20562 | -2.3894 | 18.88194 | 5.04E-03 | 0.024137 | 0.060849 | H200009073 | TUBA4B | tubulin, alpha 4b (pseudogene) | 80086 |
| 20492 | -2.5813 | 13.175 | 2.98E-03 | 0.018531 | 0.061759 | H200005273 | LRRK1 | leucine-rich repeat kinase 1 | 79705 |
| 11163 | -4.4372 | 9.898902 | 5.40E-05 | 0.00473 | 0.061807 | H200009241 | GPSM3 | G-protein signaling modulator 3 | 63940 |
| 4885 | -2.6523 | 52.36905 | 2.46E-03 | 0.017004 | 0.062691 | H200014979 | LCN2 | lipocalin 2 | 3934 |
| 6738 | -3.3938 | 60.55532 | 3.94E-04 | 0.007814 | 0.064082 | H200016030 | NA | NA | - |
| 570 | -4.4046 | 13.63496 | 5.80E-05 | 0.00473 | 0.064132 | H200005521 | PTPN6 | protein tyrosine phosphatase, non-receptor type 6 | 5777 |
| 14748 | -1.8662 | 12.19914 | 2.24E-02 | 0.054934 | 0.065323 | H200005826 | CNR2 | cannabinoid receptor 2 (macrophage) | 1269 |
| 3623 | -2.2772 | 56.03247 | 6.94E-03 | 0.028627 | 0.066187 | H200020134 | NA | NA | - |
| 3396 | -2.6783 | 9.187873 | 2.29E-03 | 0.0164 | 0.0673 | H200009452 | NA | NA | - |
| 20593 | -2.1086 | 9.041257 | 1.12E-02 | 0.037592 | 0.069071 | H200010623 | FOXJ1 | forkhead box J1 | 2302 |
| 13995 | -3.4138 | 46.17223 | 3.76E-04 | 0.007716 | 0.069608 | H200013386 | CST7 | cystatin F (leukocystatin) | 8530 |
| 7846 | -2.2554 | 5.261807 | 7.40E-03 | 0.029872 | 0.072 | H200003667 | MS4A8B | membrane-spanning 4-domains, subfamily A, member 8B | 83661 |
| 11382 | -3.0238 | 9.637546 | 9.39E-04 | 0.010845 | 0.072014 | H200019531 | SLA2 | Src-like-adaptor 2 | 84174 |
| 3376 | -2.0989 | 36.79358 | 1.15E-02 | 0.038151 | 0.073467 | H200008360 | DHRS9 | dehydrogenase/reductase (SDR family) member 9 | 10170 |
| 2056 | -3.769 | 11.66149 | 1.77E-04 | 0.00608 | 0.074116 | H200010723 | CD19 | CD19 molecule | 930 |
| 19097 | -3.4877 | 9.925846 | 3.21E-04 | 0.007429 | 0.074685 | H200000565 | SCGB1A1 | secretoglobin, family 1A, member 1 (uteroglobin) | 7356 |
| 20494 | -2.1474 | 13.13928 | 1.01E-02 | 0.035331 | 0.074982 | H200005297 | ADCY4 | adenylate cyclase 4 | 196883 |
| 13723 | -3.494 | 15.7105 | 3.16E-04 | 0.007429 | 0.077963 | H200000466 | HLA-DOB | major histocompatibility complex, class II, DO beta | 3112 |
| 20485 | -1.7795 | 9.194468 | 2.88E-02 | 0.063552 | 0.078561 | H200004893 | DYDC2 | DPY30 domain containing 2 | 84332 |
| 10740 | -4.0862 | 8.845801 | 9.89E-05 | 0.005431 | 0.079159 | H200010970 | RASGRP2 | RAS guanyl releasing protein 2 (calcium and DAG-regulated) | 10235 |
| 3300 | -1.8145 | 14.54512 | 2.60E-02 | 0.060005 | 0.079633 | H200004892 | LILRA4 | leukocyte immunoglobulin-like receptor, subfamily A (with TM domain), member 4 | 23547 |
| 21366 | -2.5172 | 19.71739 | 3.55E-03 | 0.020355 | 0.079829 | H200005285 | CCL21 | chemokine (C-C motif) ligand 21 | 6366 |
| 49 | -2.107 | 10.01811 | 1.13E-02 | 0.037686 | 0.081419 | H200002380 | IRF8 | interferon regulatory factor 8 | 3394 |
| 10637 | -2.303 | 37.86089 | 6.45E-03 | 0.027385 | 0.083045 | H200006048 | NA | NA | - |
| 485 | -3.6877 | 2.996314 | 2.06E-04 | 0.006332 | 0.083307 | H200001383 | CYTH4 | cytohesin 4 | 27128 |
| 5686 | -2.8553 | 41.59451 | 1.45E-03 | 0.013122 | 0.085126 | H200009558 | IGHM | immunoglobulin heavy constant mu | 3507 |
| 10353 | -5.9359 | 7.555964 | 4.78E-06 | 0.003715 | 0.085174 | H200014123 | PTPRCAP | protein tyrosine phosphatase, receptor type, C-associated protein | 5790 |
| 8581 | -2.7025 | 107.8474 | 2.15E-03 | 0.015982 | 0.087713 | H200016878 | NA | NA | - |
| 18449 | -2.6193 | 12.99664 | 2.69E-03 | 0.017693 | 0.088256 | H200008913 | WDFY4 | WDFY family member 4 | 57705 |
| 20173 | -1.9298 | 16.03044 | 1.87E-02 | 0.049657 | 0.088444 | H200009554 | SLAMF6 | SLAM family member 6 | 114836 |
| 8490 | -1.8527 | 13.75767 | 2.33E-02 | 0.056262 | 0.091078 | H200012656 | FAM81B | family with sequence similarity 81, member B | 153643 |
| 7835 | -2.1162 | 7.222688 | 1.10E-02 | 0.037198 | 0.092183 | H200003257 | EFCAB1 | EF-hand calcium binding domain 1 | 79645 |
| 4626 | -2.5662 | 4.894649 | 3.10E-03 | 0.018915 | 0.093 | H200002777 | CCDC17 | coiled-coil domain containing 17 | 149483 |
| 8600 | -1.8099 | 0.628173 | 2.64E-02 | 0.060372 | 0.093153 | H200017668 | NA | NA | - |
| 13466 | -3.0834 | 4.571928 | 8.12E-04 | 0.010284 | 0.094149 | H200010221 | CD180 | CD180 molecule | 4064 |
| 18913 | -2.5981 | 5.643365 | 2.85E-03 | 0.018251 | 0.095809 | H200012736 | NA | NA | - |
| 14090 | -3.8901 | 8.1294 | 1.40E-04 | 0.005831 | 0.096706 | H200017928 | NA | NA | - |
| 6200 | -2.1335 | 5.964536 | 1.05E-02 | 0.036173 | 0.096875 | H200012479 | CCDC151 | coiled-coil domain containing 151 | 115948 |
| 9564 | -3.3046 | 14.14762 | 4.83E-04 | 0.008593 | 0.09946 | H200020173 | NA | NA | - |
| 729 | -2.8246 | 6.545538 | 1.56E-03 | 0.013671 | 0.099763 | H200013115 | NA | NA | - |
| 18669 | -1.8917 | 8.177295 | 2.09E-02 | 0.052668 | 0.100322 | H200021025 | NCCRP1 | non-specific cytotoxic cell receptor protein 1 homolog (zebrafish) | 342897 |
| 16572 | -2.852 | 3.644345 | 1.46E-03 | 0.013131 | 0.100845 | H200005731 | ODF2L | outer dense fiber of sperm tails 2-like | 57489 |
| 8304 | -3.6608 | 18.44044 | 2.18E-04 | 0.006496 | 0.101878 | H200003608 | GIMAP5 | GTPase, IMAP family member 5 | 55340 |
| 13724 | -2.0309 | 25.46337 | 1.40E-02 | 0.042356 | 0.102215 | H200000472 | HCP5 | HLA complex P5 (non-protein coding) | 10866 |
| 598 | -2.3061 | 17.75686 | 6.40E-03 | 0.027263 | 0.102511 | H200006709 | CD79A | CD79a molecule, immunoglobulin-associated alpha | 973 |
| 1930 | -2.0206 | 48.51393 | 1.44E-02 | 0.043072 | 0.102602 | H200004951 | SLC27A6 | solute carrier family 27 (fatty acid transporter), member 6 | 28965 |
| 1836 | -3.3154 | 40.58604 | 4.72E-04 | 0.008585 | 0.103359 | H200000415 | NA | NA | 4687 |
| 1973 | -3.0366 | 9.123038 | 9.08E-04 | 0.010685 | 0.103911 | H200006893 | IL16 | interleukin 16 | 3603 |
| 11188 | -2.5904 | 25.29116 | 2.90E-03 | 0.018382 | 0.107426 | H200010387 | MS4A1 | membrane-spanning 4-domains, subfamily A, member 1 | 931 |
| 9478 | -3.252 | 5.164907 | 5.48E-04 | 0.008952 | 0.107727 | H200016017 | NA | NA | - |
| 9306 | -4.6434 | 3.921122 | 3.93E-05 | 0.004216 | 0.108521 | H200007989 | CD22 | CD22 molecule | 933 |
| 10363 | -2.6489 | 39.33099 | 2.48E-03 | 0.017043 | 0.108713 | H200014527 | SPIB | Spi-B transcription factor (Spi-1/PU.1 related) | 6689 |
| 18443 | -3.8286 | 4.777881 | 1.57E-04 | 0.005935 | 0.112448 | H200008841 | CPNE5 | copine V | 57699 |
| 20921 | -5.5355 | 3.517297 | 1.02E-05 | 0.003715 | 0.113738 | H200005742 | DCP1A | DCP1 decapping enzyme homolog A (S. cerevisiae) | 55802 |
| 4745 | -4.1843 | 18.32361 | 8.33E-05 | 0.005263 | 0.114025 | H200008471 | CD27 | CD27 molecule | 939 |
| 3240 | -2.8364 | 12.49051 | 1.52E-03 | 0.013482 | 0.114378 | H200001900 | NKG7 | natural killer cell group 7 sequence | 4818 |
| 3951 | -3.1082 | 9.690191 | 7.65E-04 | 0.009987 | 0.114434 | H200013851 | CD37 | CD37 molecule | 951 |
| 12123 | -5.7005 | 5.541119 | 7.78E-06 | 0.003715 | 0.115173 | H200011533 | KLF2 | Kruppel-like factor 2 (lung) | 10365 |
| 19790 | -2.4994 | 8.120932 | 3.72E-03 | 0.020804 | 0.116644 | H200012030 | SIGLEC5 | sialic acid binding Ig-like lectin 5 | 8778 |
| 11520 | -4.128 | 16.30379 | 9.19E-05 | 0.0054 | 0.116655 | H200004450 | TSPAN1 | tetraspanin 1 | 10103 |
| 2428 | -2.7242 | 4.674897 | 2.03E-03 | 0.015612 | 0.117342 | H200006828 | CD6 | CD6 molecule | 923 |
| 4601 | -5.3131 | 10.31503 | 1.46E-05 | 0.003715 | 0.11744 | H200001631 | PTGDS | prostaglandin D2 synthase 21kDa (brain) | 5730 |
| 15085 | -4.5372 | 7.180853 | 4.58E-05 | 0.004512 | 0.118545 | H200000239 | CD48 | CD48 molecule | 962 |
| 12368 | -2.9266 | 6.837345 | 1.20E-03 | 0.012106 | 0.119501 | H200001422 | RSPH1 | radial spoke head 1 homolog (Chlamydomonas) | 89765 |
| 516 | -2.3557 | 5.255706 | 5.56E-03 | 0.025423 | 0.120501 | H200002885 | PSCA | prostate stem cell antigen | 8000 |
| 19935 | -6.5778 | 4.168909 | 2.39E-06 | 0.003715 | 0.120678 | H200018876 | IL24 | interleukin 24 | 11009 |
| 3509 | -4.2595 | 86.79804 | 7.40E-05 | 0.005013 | 0.121528 | H200014790 | HLA-DQA1 | major histocompatibility complex, class II, DQ alpha 1 | 3117 |
| 9251 | -5.5779 | 2.175131 | 9.07E-06 | 0.003715 | 0.122014 | H200005347 | FAIM3 | Fas apoptotic inhibitory molecule 3 | 9214 |
| 16569 | -3.3918 | 4.453407 | 3.96E-04 | 0.007814 | 0.123015 | H200005701 | SUPT5H | suppressor of Ty 5 homolog (S. cerevisiae) | 6829 |
| 13718 | -3.6448 | 6.929831 | 2.27E-04 | 0.00661 | 0.123782 | H200000400 | ARHGAP25 | Rho GTPase activating protein 25 | 9938 |
| 1610 | -2.5875 | 4.197297 | 2.93E-03 | 0.018427 | 0.124207 | H200011613 | ACAP1 | ArfGAP with coiled-coil, ankyrin repeat and PH domains 1 | 9744 |
| 21361 | -4.3843 | 3.611184 | 5.96E-05 | 0.00473 | 0.125624 | H200004935 | GMIP | GEM interacting protein | 51291 |
| 4322 | -2.8258 | 21.34711 | 1.56E-03 | 0.013645 | 0.12575 | H200010080 | MYO19 | myosin XIX | 80179 |
| 14776 | -2.0908 | 6.109728 | 1.18E-02 | 0.038543 | 0.125758 | H200007014 | FABP4 | fatty acid binding protein 4, adipocyte | 2167 |
| 4167 | -2.873 | 27.35567 | 1.38E-03 | 0.012844 | 0.126545 | H200002546 | TNFAIP8L2 | tumor necrosis factor, alpha-induced protein 8-like 2 | 79626 |
| 16069 | -5.405 | 1.913277 | 1.26E-05 | 0.003715 | 0.128202 | H200003671 | CAPS | calcyphosine | 828 |
| 1030 | -3.8572 | 3.338079 | 1.48E-04 | 0.005879 | 0.128349 | H200005486 | IGFLR1 | IGF-like family receptor 1 | 79713 |
| 2088 | -3.9848 | 4.122144 | 1.18E-04 | 0.005541 | 0.128568 | H200012243 | PARVG | parvin, gamma | 64098 |
| 13646 | -1.8743 | 4.064738 | 2.19E-02 | 0.054174 | 0.129655 | H200018913 | CTSH | cathepsin H | 1512 |
| 2441 | -3.7584 | 9.800724 | 1.80E-04 | 0.006118 | 0.129843 | H200007558 | NA | NA | - |
| 20513 | -3.5871 | 14.796 | 2.55E-04 | 0.006843 | 0.130351 | H200006413 | HLA-DMA | major histocompatibility complex, class II, DM alpha | 3108 |
| 6084 | -3.8274 | 8.297525 | 1.57E-04 | 0.005935 | 0.130793 | H200006827 | NA | NA | 90925 |
| 11955 | -2.6328 | 6.553898 | 2.59E-03 | 0.017393 | 0.131622 | H200003553 | RAMP3 | receptor (G protein-coupled) activity modifying protein 3 | 10268 |
| 9183 | -3.358 | 15.22598 | 4.28E-04 | 0.008167 | 0.131751 | H200001975 | NA | NA | - |
| 19697 | -3.0514 | 3.257153 | 8.77E-04 | 0.010542 | 0.134104 | H200007500 | TMEM190 | transmembrane protein 190 | 147744 |
| 10711 | -2.5484 | 1.976713 | 3.26E-03 | 0.019458 | 0.134815 | H200009492 | NA | NA | - |
| 15074 | -2.0721 | 3.212035 | 1.25E-02 | 0.039539 | 0.136069 | H200008489 | RPL5 | ribosomal protein L5 | 6125 |
| 19205 | -5.1229 | 3.340474 | 1.88E-05 | 0.003715 | 0.136682 | H200005837 | NA | NA | - |
| 8372 | -3.5999 | 15.25217 | 2.49E-04 | 0.00677 | 0.137062 | H200006980 | SELL | selectin L | 6402 |
| 1018 | -3.0457 | 3.197162 | 8.90E-04 | 0.010642 | 0.138617 | H200005058 | CFP | complement factor properdin | 5199 |
| 2435 | -3.06 | 3.19558 | 8.59E-04 | 0.010432 | 0.138944 | H200007202 | MAP4K1 | mitogen-activated protein kinase kinase kinase kinase 1 | 11184 |
| 9190 | -2.8541 | 6.044896 | 1.45E-03 | 0.013129 | 0.139723 | H200002337 | NAALADL1 | N-acetylated alpha-linked acidic dipeptidase-like 1 | 10004 |
| 15893 | -3.3022 | 10.32863 | 4.86E-04 | 0.008593 | 0.141489 | H200016876 | NA | NA | - |
| 19871 | -2.8784 | 11.21927 | 1.36E-03 | 0.012738 | 0.141498 | H200015836 | RBP5 | retinol binding protein 5, cellular | 83758 |
| 13743 | -3.1915 | 1.97053 | 6.28E-04 | 0.009386 | 0.142971 | H200001558 | VSTM2L | V-set and transmembrane domain containing 2 like | 128434 |
| 12110 | -2.0308 | 6.611417 | 1.40E-02 | 0.042356 | 0.14345 | H200010803 | NA | NA | - |
| 6747 | -2.1967 | 2.837286 | 8.73E-03 | 0.032584 | 0.144568 | H200016428 | EFHB | EF-hand domain family, member B | 151651 |
| 3351 | -2.4673 | 5.57363 | 4.06E-03 | 0.021871 | 0.144883 | H200007214 | EPHX3 | epoxide hydrolase 3 | 79852 |
| 19439 | -3.6609 | 13.14378 | 2.18E-04 | 0.006496 | 0.147748 | H200016881 | NA | NA | - |
| 11521 | -5.1871 | 3.43116 | 1.73E-05 | 0.003715 | 0.147987 | H200004752 | PPP1R16B | protein phosphatase 1, regulatory subunit 16B | 26051 |
| 21673 | -1.852 | 16.98193 | 2.34E-02 | 0.056341 | 0.148764 | H200019755 | SIGLEC10 | sialic acid binding Ig-like lectin 10 | 89790 |
| 7081 | -2.2094 | 3.375479 | 8.43E-03 | 0.032001 | 0.148947 | H200010941 | CCDC65 | coiled-coil domain containing 65 | 85478 |
| 16803 | -5.3026 | 1.881065 | 1.49E-05 | 0.003715 | 0.149361 | H200016745 | UPK1B | uroplakin 1B | 7348 |
| 7973 | -2.6235 | 7.833663 | 2.66E-03 | 0.017585 | 0.15067 | H200009741 | NA | NA | - |
| 14971 | -1.9593 | 13.25325 | 1.72E-02 | 0.047462 | 0.150725 | H200016460 | IKZF3 | IKAROS family zinc finger 3 (Aiolos) | 22806 |
| 1631 | -1.8666 | 10.65283 | 2.24E-02 | 0.054907 | 0.150788 | H200012439 | NA | NA | - |
| 4796 | -2.0408 | 6.337901 | 1.36E-02 | 0.041653 | 0.152256 | H200010781 | CCL22 | chemokine (C-C motif) ligand 22 | 6367 |
| 8928 | -3.0881 | 3.777184 | 8.03E-04 | 0.010245 | 0.152715 | H200011683 | PSD4 | pleckstrin and Sec7 domain containing 4 | 23550 |
| 21680 | -2.9481 | 5.524893 | 1.13E-03 | 0.011762 | 0.152884 | H200020129 | LOC440786 | uncharacterized LOC440786 | 440786 |
| 14986 | -2.3953 | 2.996463 | 4.96E-03 | 0.023935 | 0.15435 | H200017202 | NA | NA | - |
| 16060 | -4.6091 | 2.845797 | 4.16E-05 | 0.004304 | 0.154468 | H200003273 | TFEB | transcription factor EB | 7942 |
| 15137 | -4.3581 | 3.508396 | 6.24E-05 | 0.00476 | 0.154581 | H200002851 | ARHGAP9 | Rho GTPase activating protein 9 | 64333 |
| 10472 | -4.3873 | 7.669463 | 5.93E-05 | 0.00473 | 0.154974 | H200019805 | IGLJ3 | immunoglobulin lambda joining 3 | 28831 |
| 12902 | -2.9287 | 2.979595 | 1.19E-03 | 0.01207 | 0.155835 | H200004996 | SIRPG | signal-regulatory protein gamma | 55423 |
| 7545 | -2.3027 | 8.650343 | 6.46E-03 | 0.027394 | 0.156519 | H200011118 | FAM65B | family with sequence similarity 65, member B | 9750 |
| 18819 | -2.8048 | 5.184146 | 1.64E-03 | 0.014047 | 0.156881 | H200007464 | S1PR4 | sphingosine-1-phosphate receptor 4 | 8698 |
| 11837 | -3.1025 | 6.404535 | 7.76E-04 | 0.010041 | 0.157015 | H200019620 | HVCN1 | hydrogen voltage-gated channel 1 | 84329 |
| 15947 | -3.8661 | 33.26175 | 1.46E-04 | 0.005857 | 0.160427 | H200019512 | NA | NA | - |
| 20805 | -4.8162 | 2.068404 | 2.99E-05 | 0.004028 | 0.160796 | H200000090 | IL10RA | interleukin 10 receptor, alpha | 3587 |
| 21099 | -3.0338 | 1.851718 | 9.15E-04 | 0.010731 | 0.160992 | H200014126 | MSLN | mesothelin | 10232 |
| 20464 | -3.9506 | 4.073519 | 1.25E-04 | 0.005665 | 0.1619 | H200003753 | TSPAN33 | tetraspanin 33 | 340348 |
| 12282 | -2.3264 | 2.231502 | 6.04E-03 | 0.026561 | 0.162633 | H200019115 | NA | NA | - |
| 7945 | -2.9922 | 7.218954 | 1.01E-03 | 0.011215 | 0.164204 | H200008553 | GNGT2 | guanine nucleotide binding protein (G protein), gamma transducing activity polypeptide 2 | 2793 |
| 1231 | -2.0765 | 2.719453 | 1.23E-02 | 0.039392 | 0.164378 | H200015004 | NA | NA | - |
| 5872 | -2.2127 | 9.001411 | 8.36E-03 | 0.031783 | 0.164869 | H200018322 | ATP6V1D | ATPase, H+ transporting, lysosomal 34kDa, V1 subunit D | 51382 |
| 15204 | -7.8337 | 3.308272 | 3.99E-07 | 0.003715 | 0.165036 | H200005921 | ADIRF | adipogenesis regulatory factor | 10974 |
| 1215 | -4.6526 | 1.185556 | 3.89E-05 | 0.004216 | 0.166662 | H200014244 | LY86 | lymphocyte antigen 86 | 9450 |
| 21271 | -3.1095 | 13.68475 | 7.63E-04 | 0.00998 | 0.167356 | H200000731 | GZMK | granzyme K (granzyme 3; tryptase II) | 3003 |
| 14618 | -2.4494 | 1.7436 | 4.27E-03 | 0.02224 | 0.167933 | H2NC000009 | NA | NA | - |
| 11232 | -3.298 | 5.074807 | 4.91E-04 | 0.008622 | 0.169552 | H200012335 | MYO1F | myosin IF | 4542 |
| 19438 | -3.5206 | 20.66205 | 2.97E-04 | 0.007417 | 0.169663 | H200016875 | NA | NA | - |
| 18323 | -3.3778 | 2.631094 | 4.08E-04 | 0.007963 | 0.172224 | H200002073 | TMEM176A | transmembrane protein 176A | 55365 |
| 5315 | -2.5692 | 6.648312 | 3.08E-03 | 0.018851 | 0.172756 | H200013910 | MNDA | myeloid cell nuclear differentiation antigen | 4332 |
| 21114 | -2.5055 | 4.580053 | 3.66E-03 | 0.020647 | 0.173209 | H200014880 | APOBR | apolipoprotein B receptor | 55911 |
| 8729 | -4.0431 | 1.529916 | 1.08E-04 | 0.005439 | 0.173224 | H200002485 | DEF6 | differentially expressed in FDCP 6 homolog (mouse) | 50619 |
| 14289 | -3.2039 | 2.71125 | 6.09E-04 | 0.009361 | 0.17427 | H200005999 | TRIM9 | tripartite motif containing 9 | 114088 |
| 2093 | -2.0628 | 2.430328 | 1.28E-02 | 0.040063 | 0.174944 | H200012593 | GRID1 | glutamate receptor, ionotropic, delta 1 | 2894 |
| 12457 | -2.5168 | 3.023041 | 3.55E-03 | 0.020357 | 0.175723 | H200005904 | NA | NA | 400786 |
| 14631 | -5.1525 | 1.973356 | 1.81E-05 | 0.003715 | 0.176299 | H200000168 | CYP4B1 | cytochrome P450, family 4, subfamily B, polypeptide 1 | 1580 |
| 15544 | -2.966 | 15.38481 | 1.08E-03 | 0.011546 | 0.176547 | H200000186 | FCER1G | Fc fragment of IgE, high affinity I, receptor for; gamma polypeptide | 2207 |
| 21694 | -2.1345 | 2.314514 | 1.04E-02 | 0.0361 | 0.176714 | H200020865 | NA | NA | - |
| 21364 | -2.2476 | 2.531401 | 7.57E-03 | 0.030316 | 0.17694 | H200005261 | HAS1 | hyaluronan synthase 1 | 3036 |
| 9320 | -5.9061 | 1.64108 | 5.18E-06 | 0.003715 | 0.177209 | H200008441 | FERMT3 | fermitin family member 3 | 83706 |
| 7827 | -2.0104 | 2.622078 | 1.49E-02 | 0.043702 | 0.177538 | H200002877 | STAG3 | stromal antigen 3 | 10734 |
| 17014 | -5.5412 | 2.193496 | 1.01E-05 | 0.003715 | 0.178215 | H200005226 | LAT2 | linker for activation of T cells family, member 2 | 7462 |
| 1382 | -2.5718 | 3.075869 | 3.06E-03 | 0.018794 | 0.178649 | H200000641 | TNFRSF17 | tumor necrosis factor receptor superfamily, member 17 | 608 |
| 16878 | -2.7006 | 10.70449 | 2.16E-03 | 0.016009 | 0.179788 | H200020195 | NA | NA | - |
| 1489 | -3.1891 | 2.746363 | 6.31E-04 | 0.009397 | 0.180175 | H200005907 | LAG3 | lymphocyte-activation gene 3 | 3902 |
| 5485 | -3.3148 | 5.011425 | 4.72E-04 | 0.008585 | 0.180312 | H200000052 | DARC | Duffy blood group, chemokine receptor | 2532 |
| 8268 | -1.9906 | 2.033926 | 1.57E-02 | 0.045068 | 0.180715 | H200002040 | TM4SF4 | transmembrane 4 L six family member 4 | 7104 |
| 9735 | -4.1196 | 4.888159 | 9.33E-05 | 0.0054 | 0.181613 | H200006772 | DPT | dermatopontin | 1805 |
| 1479 | -2.7972 | 2.13293 | 1.68E-03 | 0.014184 | 0.181847 | H200005219 | RAB33A | RAB33A, member RAS oncogene family | 9363 |
| 14875 | -3.3215 | 4.401753 | 4.64E-04 | 0.00858 | 0.18201 | H200011900 | LGALS2 | lectin, galactoside-binding, soluble, 2 | 3957 |
| 9349 | -4.0074 | 1.879081 | 1.14E-04 | 0.005439 | 0.182285 | H200009931 | L3MBTL1 | l(3)mbt-like 1 (Drosophila) | 26013 |
| 14397 | -4.0086 | 1.302577 | 1.14E-04 | 0.005439 | 0.182757 | H200010987 | 1-Sep | septin 1 | 1731 |
| 4360 | -3.6291 | 1.534751 | 2.34E-04 | 0.006651 | 0.183851 | H200011672 | RHOH | ras homolog family member H | 399 |
| 920 | -1.8078 | 4.412133 | 2.65E-02 | 0.060627 | 0.183993 | H200000190 | NA | NA | - |
| 20104 | -2.9752 | 2.086428 | 1.06E-03 | 0.011443 | 0.184581 | H200005772 | PRSS21 | protease, serine, 21 (testisin) | 10942 |
| 9440 | -2.0496 | 2.518055 | 1.33E-02 | 0.040923 | 0.184858 | H200014141 | NA | NA | - |
| 11254 | -2.6231 | 2.189047 | 2.66E-03 | 0.017597 | 0.185337 | H200013451 | PLA2G10 | phospholipase A2, group X | 8399 |
| 16917 | -1.871 | 2.470212 | 2.21E-02 | 0.054414 | 0.186147 | H200000364 | EGR2 | early growth response 2 | 1959 |
| 2456 | -2.0713 | 2.2539 | 1.25E-02 | 0.039555 | 0.187488 | H200008300 | TTC16 | tetratricopeptide repeat domain 16 | 158248 |
| 12165 | -2.2235 | 2.85453 | 8.10E-03 | 0.031288 | 0.187518 | H200013457 | TCL6 | T-cell leukemia/lymphoma 6 (non-protein coding) | 27004 |
| 7908 | -1.8494 | 3.141292 | 2.35E-02 | 0.056608 | 0.187777 | H200006683 | CCNA1 | cyclin A1 | 8900 |
| 9456 | -2.3194 | 3.829386 | 6.16E-03 | 0.026737 | 0.188995 | H200014901 | CCDC78 | coiled-coil domain containing 78 | 124093 |
| 6027 | -1.7836 | 2.57232 | 2.84E-02 | 0.063182 | 0.189056 | H200004161 | NA | NA | - |
| 15644 | -2.4072 | 10.86522 | 4.80E-03 | 0.023472 | 0.189231 | H200005078 | CLEC10A | C-type lectin domain family 10, member A | 10462 |
| 8002 | -2.5024 | 3.90295 | 3.69E-03 | 0.020741 | 0.189763 | H200011219 | RPS6KB2 | ribosomal protein S6 kinase, 70kDa, polypeptide 2 | 6199 |
| 21438 | -1.8814 | 5.06144 | 2.15E-02 | 0.053552 | 0.190188 | H200008705 | NA | NA | - |
| 9477 | -3.7601 | 9.061869 | 1.79E-04 | 0.006117 | 0.190336 | H200016011 | NA | NA | - |
| 4127 | -2.1416 | 1.918064 | 1.02E-02 | 0.035729 | 0.190462 | H200000646 | DEFA4 | defensin, alpha 4, corticostatin | 1669 |
| 556 | -3.6382 | 5.43919 | 2.30E-04 | 0.00665 | 0.190683 | H200004785 | NA | NA | - |
| 16699 | -3.6261 | 7.972134 | 2.35E-04 | 0.006659 | 0.192047 | H200011805 | C15orf48 | chromosome 15 open reading frame 48 | 84419 |
| 1379 | -5.9551 | 1.200994 | 4.49E-06 | 0.003715 | 0.192461 | H200000611 | POU2AF1 | POU class 2 associating factor 1 | 5450 |
| 18199 | -1.8919 | 2.900725 | 2.08E-02 | 0.052648 | 0.192493 | H200017832 | C11orf16 | chromosome 11 open reading frame 16 | 56673 |
| 11206 | -1.8919 | 3.8133 | 2.08E-02 | 0.052648 | 0.19275 | H200011171 | MXD4 | MAX dimerization protein 4 | 10608 |
| 15735 | -1.9262 | 9.632234 | 1.89E-02 | 0.049925 | 0.193292 | H200009300 | MPEG1 | macrophage expressed 1 | 219972 |
| 13271 | -5.7178 | 1.329444 | 7.68E-06 | 0.003715 | 0.194132 | H200001071 | ATP2A3 | ATPase, Ca++ transporting, ubiquitous | 489 |
| 10527 | -3.0997 | 2.243531 | 7.81E-04 | 0.010095 | 0.195018 | H200000752 | NA | NA | - |
| 4226 | -2.8052 | 2.101984 | 1.64E-03 | 0.014047 | 0.196107 | H200005520 | C11orf84 | chromosome 11 open reading frame 84 | 144097 |
| 5049 | -5.3259 | 1.097231 | 1.44E-05 | 0.003715 | 0.196228 | H200001346 | SIPA1 | signal-induced proliferation-associated 1 | 6494 |
| 15331 | -3.3926 | 2.20761 | 3.95E-04 | 0.007814 | 0.196933 | H200011995 | SLC25A34 | solute carrier family 25, member 34 | 284723 |
| 9605 | -3.24 | 6.345231 | 5.63E-04 | 0.009073 | 0.197353 | H200000668 | WFDC2 | WAP four-disulfide core domain 2 | 10406 |
| 9451 | -3.4015 | 1.343064 | 3.86E-04 | 0.00776 | 0.198013 | H200014847 | INE1 | inactivation escape 1 (non-protein coding) | 8552 |
| 3546 | -3.529 | 2.758422 | 2.91E-04 | 0.007373 | 0.198179 | H200016648 | RIPK3 | receptor-interacting serine-threonine kinase 3 | 11035 |
| 15088 | -3.4658 | 2.209495 | 3.37E-04 | 0.007534 | 0.198803 | H200000269 | RRAD | Ras-related associated with diabetes | 6236 |
| 2846 | -3.3367 | 22.94782 | 4.48E-04 | 0.008358 | 0.198901 | H200004963 | CCL19 | chemokine (C-C motif) ligand 19 | 6363 |
| 9407 | -2.951 | 2.899765 | 1.12E-03 | 0.011725 | 0.199117 | H200012615 | C12orf42 | chromosome 12 open reading frame 42 | 374470 |
| 10617 | -2.8522 | 2.669088 | 1.46E-03 | 0.013131 | 0.199455 | H200005240 | LBH | limb bud and heart development | 81606 |
| 5482 | -2.0341 | 2.082783 | 1.39E-02 | 0.042134 | 0.199858 | H200000010 | LTA | lymphotoxin alpha (TNF superfamily, member 1) | 4049 |
| 18746 | -2.8676 | 2.593819 | 1.40E-03 | 0.012925 | 0.199911 | H200003634 | TNFSF12 | tumor necrosis factor (ligand) superfamily, member 12 | 8742 |
| 16768 | -2.0117 | 2.918744 | 1.48E-02 | 0.043665 | 0.200066 | H200014899 | TTC25 | tetratricopeptide repeat domain 25 | 83538 |
| 17041 | -1.9722 | 36.0179 | 1.66E-02 | 0.046455 | 0.200082 | H200006396 | PLEK | pleckstrin | 5341 |
| 17706 | -2.2184 | 2.346934 | 8.22E-03 | 0.031526 | 0.200849 | H200016021 | MC3R | melanocortin 3 receptor | 4159 |
| 18189 | -3.7137 | 1.052364 | 1.96E-04 | 0.006274 | 0.200852 | H200017428 | NA | NA | - |
| 17243 | -2.3242 | 34.32274 | 6.08E-03 | 0.02664 | 0.201214 | H200015920 | NA | NA | - |
| 13799 | -3.5012 | 4.219541 | 3.11E-04 | 0.007429 | 0.201302 | H200004218 | SLC2A5 | solute carrier family 2 (facilitated glucose/fructose transporter), member 5 | 6518 |
| 2170 | -1.8139 | 3.903678 | 2.61E-02 | 0.060043 | 0.202118 | H200016351 | CCDC11 | coiled-coil domain containing 11 | 220136 |
| 2497 | -1.9972 | 2.246799 | 1.54E-02 | 0.044765 | 0.202436 | H200010218 | NA | NA | - |
| 9091 | -1.8237 | 4.029935 | 2.54E-02 | 0.059009 | 0.202469 | H200019609 | MUC20 | mucin 20, cell surface associated | 200958 |
| 4071 | -2.5433 | 3.371793 | 3.30E-03 | 0.019584 | 0.202595 | H200019551 | SLC44A4 | solute carrier family 44, member 4 | 80736 |
| 21566 | -2.4075 | 0.636634 | 4.80E-03 | 0.023472 | 0.203627 | H200014785 | AOC3 | amine oxidase, copper containing 3 (vascular adhesion protein 1) | 8639 |
| 6575 | -2.702 | 2.564114 | 2.15E-03 | 0.015982 | 0.203816 | H200008400 | RIN3 | Ras and Rab interactor 3 | 79890 |
| 11545 | -2.6529 | 66.48988 | 2.46E-03 | 0.016997 | 0.20416 | H200005892 | HLA-DQB1 | major histocompatibility complex, class II, DQ beta 1 | 3119 |
| 2948 | -2.8878 | 2.457257 | 1.32E-03 | 0.012529 | 0.204223 | H200009595 | CARD11 | caspase recruitment domain family, member 11 | 84433 |
| 20115 | -2.138 | 8.996259 | 1.03E-02 | 0.035899 | 0.204453 | H200006490 | PECAM1 | platelet/endothelial cell adhesion molecule 1 | 5175 |
| 20426 | -4.0849 | 1.999747 | 9.94E-05 | 0.005431 | 0.205446 | H200001811 | PSMB10 | proteasome (prosome, macropain) subunit, beta type, 10 | 5699 |
| 16859 | -3.9411 | 1.023187 | 1.27E-04 | 0.00567 | 0.205473 | H200019405 | FXYD5 | FXYD domain containing ion transport regulator 5 | 53827 |
| 13937 | -2.65 | 4.609161 | 2.47E-03 | 0.017018 | 0.205696 | H200010702 | SOX15 | SRY (sex determining region Y)-box 15 | 6665 |
| 258 | -2.3228 | 2.139963 | 6.10E-03 | 0.02668 | 0.205949 | H200012266 | C9orf116 | chromosome 9 open reading frame 116 | 138162 |
| 18977 | -2.8277 | 3.461318 | 1.55E-03 | 0.01361 | 0.206985 | H200016174 | GFI1B | growth factor independent 1B transcription repressor | 8328 |
| 5525 | -3.8709 | 1.724347 | 1.45E-04 | 0.005857 | 0.20776 | H200001952 | NLRC5 | NLR family, CARD domain containing 5 | 84166 |
| 15172 | -3.8272 | 0.973479 | 1.57E-04 | 0.005935 | 0.208722 | H200004401 | MYO1G | myosin IG | 64005 |
| 4398 | -2.2637 | 1.727772 | 7.22E-03 | 0.029479 | 0.208752 | H200013548 | GBA3 | glucosidase, beta, acid 3 (cytosolic) | 57733 |
| 16339 | -3.6189 | 1.389432 | 2.38E-04 | 0.006669 | 0.208782 | H200016567 | FCRLA | Fc receptor-like A | 84824 |
| 1213 | -1.996 | 7.953674 | 1.55E-02 | 0.044836 | 0.20891 | H200014220 | MSMB | microseminoprotein, beta- | 4477 |
| 1575 | -1.936 | 2.112658 | 1.84E-02 | 0.049255 | 0.208924 | H200009779 | NA | NA | - |
| 17606 | -2.409 | 28.4142 | 4.78E-03 | 0.023403 | 0.208979 | H200011413 | LTF | lactotransferrin | 4057 |
| 1932 | -3.0683 | 4.785051 | 8.42E-04 | 0.010389 | 0.209567 | H200004975 | IFT27 | intraflagellar transport 27 homolog (Chlamydomonas) | 11020 |
| 16464 | -2.9172 | 1.814134 | 1.22E-03 | 0.012195 | 0.209747 | H200000459 | LCK | lymphocyte-specific protein tyrosine kinase | 3932 |
| 11138 | -3.9406 | 1.565528 | 1.28E-04 | 0.00567 | 0.212557 | H200008083 | IL16 | interleukin 16 | 3603 |
| 4114 | -3.5528 | 5.451518 | 2.74E-04 | 0.007084 | 0.212629 | H200000200 | GPR183 | G protein-coupled receptor 183 | 1880 |
| 20093 | -2.0105 | 2.005957 | 1.49E-02 | 0.043702 | 0.212977 | H200005344 | CD5 | CD5 molecule | 921 |
| 10457 | -5.2691 | 1.504941 | 1.56E-05 | 0.003715 | 0.213748 | H200019063 | NA | NA | - |
| 2955 | -2.1125 | 3.841236 | 1.11E-02 | 0.037401 | 0.214077 | H200009969 | EFHC2 | EF-hand domain (C-terminal) containing 2 | 80258 |
| 4131 | -5.6664 | 1.115144 | 8.27E-06 | 0.003715 | 0.214302 | H200000978 | FGD3 | FYVE, RhoGEF and PH domain containing 3 | 89846 |
| 708 | -2.5425 | 3.962711 | 3.31E-03 | 0.019595 | 0.214785 | H200012005 | NA | NA | - |
| 3928 | -4.7536 | 1.52639 | 3.37E-05 | 0.004054 | 0.215168 | H200012717 | GNG7 | guanine nucleotide binding protein (G protein), gamma 7 | 2788 |
| 8919 | -3.1095 | 2.517238 | 7.63E-04 | 0.00998 | 0.216218 | H200011297 | NOP14-AS1 | NOP14 antisense RNA 1 | 317648 |
| 2020 | -2.5778 | 2.047637 | 3.01E-03 | 0.018597 | 0.217202 | H200009155 | CRLF2 | cytokine receptor-like factor 2 | 64109 |
| 17486 | -2.1224 | 1.641439 | 1.08E-02 | 0.036888 | 0.217714 | H200005713 | HINT2 | histidine triad nucleotide binding protein 2 | 84681 |
| 628 | -2.4598 | 17.28048 | 4.15E-03 | 0.022075 | 0.217776 | H200008205 | CSF1R | colony stimulating factor 1 receptor | 1436 |
| 1254 | -1.9108 | 3.7326 | 1.98E-02 | 0.051224 | 0.219603 | H200016126 | HHCM | Mahlavu hepatocellular carcinoma | 10639 |
| 16149 | -5.1232 | 1.05688 | 1.87E-05 | 0.003715 | 0.220182 | H200007471 | AKNA | AT-hook transcription factor | 80709 |
| 3542 | -1.9802 | 3.769874 | 1.62E-02 | 0.045841 | 0.221003 | H200016316 | FCN1 | ficolin (collagen/fibrinogen domain containing) 1 | 2219 |
| 1557 | -2.5623 | 1.468826 | 3.14E-03 | 0.019068 | 0.221468 | H200008995 | NA | NA | - |
| 15855 | -1.8267 | 8.813592 | 2.51E-02 | 0.058663 | 0.22183 | H200015000 | ENTPD1 | ectonucleoside triphosphate diphosphohydrolase 1 | 953 |
| 17826 | -4.8862 | 2.787482 | 2.67E-05 | 0.004028 | 0.221839 | H200000298 | HLA-DMB | major histocompatibility complex, class II, DM beta | 3109 |
| 10629 | -2.1094 | 2.343127 | 1.12E-02 | 0.037569 | 0.222122 | H200005668 | NA | NA | - |
| 12467 | -3.231 | 5.245504 | 5.74E-04 | 0.009191 | 0.222594 | H200006308 | RGCC | regulator of cell cycle | 28984 |
| 20 | -2.0227 | 1.799896 | 1.44E-02 | 0.042936 | 0.223023 | H200000890 | SVOP | SV2 related protein homolog (rat) | 55530 |
| 18392 | -3.0181 | 1.773542 | 9.53E-04 | 0.010914 | 0.223778 | H200005867 | SELP | selectin P (granule membrane protein 140kDa, antigen CD62) | 6403 |
| 18218 | -2.2391 | 3.952373 | 7.75E-03 | 0.030653 | 0.223951 | H200018918 | NA | NA | - |
| 548 | -3.9583 | 1.432185 | 1.23E-04 | 0.005658 | 0.224062 | H200004405 | RARRES2 | retinoic acid receptor responder (tazarotene induced) 2 | 5919 |
| 5770 | -2.4396 | 1.638674 | 4.39E-03 | 0.02241 | 0.224221 | H200013690 | HIST1H1A | histone cluster 1, H1a | 3024 |
| 2605 | -1.9496 | 3.879702 | 1.77E-02 | 0.048266 | 0.224675 | H200015490 | LINC00158 | long intergenic non-protein coding RNA 158 | 54072 |
| 19117 | -1.94 | 1.325834 | 1.82E-02 | 0.048978 | 0.225044 | H200001657 | DUS2L | dihydrouridine synthase 2-like, SMM1 homolog (S. cerevisiae) | 54920 |
| 20733 | -2.2818 | 1.514696 | 6.85E-03 | 0.028388 | 0.225176 | H200018223 | SLITRK6 | SLIT and NTRK-like family, member 6 | 84189 |
| 15076 | -2.1682 | 2.123271 | 9.48E-03 | 0.034246 | 0.225755 | H200008489 | RPL5 | ribosomal protein L5 | 6125 |
| 2353 | -4.7296 | 1.251734 | 3.47E-05 | 0.004076 | 0.225976 | H200003378 | FOLR2 | folate receptor 2 (fetal) | 2350 |
| 21098 | -2.4297 | 4.211549 | 4.51E-03 | 0.022738 | 0.226171 | H200014120 | NA | NA | - |
| 1745 | -3.0684 | 1.94116 | 8.42E-04 | 0.010389 | 0.226443 | H200018067 | KLHL31 | kelch-like family member 31 | 401265 |
| 2124 | -1.8925 | 1.50762 | 2.08E-02 | 0.052624 | 0.226506 | H200014095 | LRRC23 | leucine rich repeat containing 23 | 10233 |
| 15538 | -4.0053 | 1.050211 | 1.14E-04 | 0.005439 | 0.227023 | H200000114 | FLT3LG | fms-related tyrosine kinase 3 ligand | 2323 |
| 15792 | -4.3111 | 1.445714 | 6.77E-05 | 0.004858 | 0.227286 | H200011966 | DPEP2 | dipeptidase 2 | 64174 |
| 7575 | -2.5219 | 9.733409 | 3.50E-03 | 0.020189 | 0.227411 | H200012330 | WNT10A | wingless-type MMTV integration site family, member 10A | 80326 |
| 20188 | -2.1477 | 2.11806 | 1.00E-02 | 0.035308 | 0.227525 | H200010332 | CD2 | CD2 molecule | 914 |
| 2476 | -2.0667 | 1.509713 | 1.27E-02 | 0.039784 | 0.227986 | H200009108 | NA | NA | - |
| 15473 | -3.2097 | 1.487012 | 6.01E-04 | 0.009326 | 0.22846 | H200018811 | SP140 | SP140 nuclear body protein | 11262 |
| 9445 | -3.7473 | 2.887564 | 1.84E-04 | 0.006172 | 0.228573 | H200014491 | FCRL5 | Fc receptor-like 5 | 83416 |
| 9434 | -2.8023 | 3.567244 | 1.65E-03 | 0.014054 | 0.228702 | H200014069 | NA | NA | - |
| 9241 | -1.9479 | 4.690923 | 1.78E-02 | 0.048329 | 0.228812 | H200004943 | RAI2 | retinoic acid induced 2 | 10742 |
| 8286 | -1.9582 | 3.333709 | 1.73E-02 | 0.04755 | 0.229107 | H200002824 | S100A12 | S100 calcium binding protein A12 | 6283 |
| 14906 | -2.6654 | 1.735187 | 2.38E-03 | 0.016707 | 0.229152 | H200013402 | TEKT5 | tektin 5 | 146279 |
| 13858 | -5.1719 | 1.851995 | 1.75E-05 | 0.003715 | 0.229199 | H200006908 | CD53 | CD53 molecule | 963 |
| 19375 | -1.8369 | 3.830614 | 2.44E-02 | 0.057671 | 0.229227 | H200013841 | VWA5A | von Willebrand factor A domain containing 5A | 4013 |
| 14282 | -1.845 | 6.1228 | 2.38E-02 | 0.056985 | 0.229461 | H200005625 | CCL17 | chemokine (C-C motif) ligand 17 | 6361 |
| 12343 | -3.0788 | 6.727148 | 8.20E-04 | 0.010325 | 0.229529 | H200000276 | GZMB | granzyme B (granzyme 2, cytotoxic T-lymphocyte-associated serine esterase 1) | 3002 |
| 4481 | -2.2088 | 3.387131 | 8.44E-03 | 0.032024 | 0.229844 | H200017674 | NA | NA | - |
| 9457 | -2.2081 | 1.777762 | 8.46E-03 | 0.032052 | 0.229996 | H200015203 | GPR123 | G protein-coupled receptor 123 | 84435 |
| 20768 | -2.9863 | 2.911205 | 1.03E-03 | 0.011308 | 0.230229 | H200020123 | C17orf72 | chromosome 17 open reading frame 72 | 92340 |
| 7773 | -3.0363 | 18.68412 | 9.09E-04 | 0.010685 | 0.230263 | H200000241 | HLA-DPA1 | major histocompatibility complex, class II, DP alpha 1 | 3113 |
| 11319 | -4.0543 | 4.517129 | 1.06E-04 | 0.005439 | 0.230938 | H200016509 | TRAF3IP3 | TRAF3 interacting protein 3 | 80342 |
| 3285 | -2.5049 | 2.546364 | 3.67E-03 | 0.020675 | 0.231543 | H200004150 | SLC25A23 | solute carrier family 25 (mitochondrial carrier; phosphate carrier), member 23 | 79085 |
| 14169 | -4.1692 | 1.448379 | 8.49E-05 | 0.005263 | 0.232223 | H200000299 | ATP12A | ATPase, H+/K+ transporting, nongastric, alpha polypeptide | 479 |
| 18835 | -1.9198 | 1.991363 | 1.92E-02 | 0.050442 | 0.232959 | H200008248 | AQP8 | aquaporin 8 | 343 |
| 5318 | -2.8392 | 1.845442 | 1.50E-03 | 0.013422 | 0.233168 | H200013940 | CHI3L2 | chitinase 3-like 2 | 1117 |
| 18729 | -4.8659 | 1.168436 | 2.80E-05 | 0.004028 | 0.233292 | H200002542 | STK10 | serine/threonine kinase 10 | 6793 |
| 6011 | -1.8555 | 16.9921 | 2.31E-02 | 0.055959 | 0.233493 | H200003401 | CXCL14 | chemokine (C-X-C motif) ligand 14 | 9547 |
| 6221 | -2.8934 | 4.135726 | 1.30E-03 | 0.012515 | 0.233887 | H200013589 | RAB37 | RAB37, member RAS oncogene family | 326624 |
| 12905 | -4.5093 | 1.355388 | 4.81E-05 | 0.004564 | 0.234317 | H200005322 | ABI3BP | ABI family, member 3 (NESH) binding protein | 25890 |
| 3540 | -2.1597 | 1.998254 | 9.72E-03 | 0.034686 | 0.234676 | H200016292 | HRH3 | histamine receptor H3 | 11255 |
| 926 | -1.9974 | 1.574619 | 1.54E-02 | 0.044762 | 0.23503 | H200000546 | WAS | Wiskott-Aldrich syndrome | 7454 |
| 7162 | -4.4101 | 1.33779 | 5.74E-05 | 0.00473 | 0.235311 | H200014747 | HMHA1 | histocompatibility (minor) HA-1 | 23526 |
| 6490 | -2.3316 | 1.649213 | 5.95E-03 | 0.026313 | 0.236171 | H200004250 | NA | NA | - |
| 8914 | -4.8848 | 0.890242 | 2.68E-05 | 0.004028 | 0.236392 | H200011231 | RAB3IP | RAB3A interacting protein | 117177 |
| 156 | -2.5286 | 1.366076 | 3.43E-03 | 0.019964 | 0.23645 | H200007350 | TNFRSF13B | tumor necrosis factor receptor superfamily, member 13B | 23495 |
| 15657 | -2.5209 | 1.184949 | 3.51E-03 | 0.020229 | 0.237009 | H200005808 | CYP2F1 | cytochrome P450, family 2, subfamily F, polypeptide 1 | 1572 |
| 14751 | -2.9504 | 1.108803 | 1.12E-03 | 0.011725 | 0.237188 | H200005868 | ALOX15 | arachidonate 15-lipoxygenase | 246 |
| 10674 | -3.2718 | 1.290413 | 5.21E-04 | 0.008787 | 0.238234 | H200007906 | NUDT18 | nudix (nucleoside diphosphate linked moiety X)-type motif 18 | 79873 |
| 10132 | -2.3054 | 6.25971 | 6.41E-03 | 0.027284 | 0.238257 | H200003513 | IL1R2 | interleukin 1 receptor, type II | 7850 |
| 3032 | -2.5398 | 3.887316 | 3.33E-03 | 0.019619 | 0.23886 | H200013727 | NDUFS7 | NADH dehydrogenase (ubiquinone) Fe-S protein 7, 20kDa (NADH-coenzyme Q reductase) | 374291 |
| 238 | -4.4156 | 1.770454 | 5.67E-05 | 0.00473 | 0.238928 | H200011174 | TSC22D4 | TSC22 domain family, member 4 | 81628 |
| 9271 | -2.1991 | 21.79814 | 8.68E-03 | 0.032474 | 0.23899 | H200006155 | CD14 | CD14 molecule | 929 |
| 3722 | -2.3719 | 7.620669 | 5.30E-03 | 0.02471 | 0.238996 | H200003145 | LILRB2 | leukocyte immunoglobulin-like receptor, subfamily B (with TM and ITIM domains), member 2 | 10288 |
| 10668 | -4.5083 | 1.125895 | 4.83E-05 | 0.004564 | 0.239049 | H200007550 | PIK3CD | phosphatidylinositol-4,5-bisphosphate 3-kinase, catalytic subunit delta | 5293 |
| 17592 | -2.7602 | 8.097504 | 1.84E-03 | 0.014844 | 0.23918 | H200010677 | C1QC | complement component 1, q subcomponent, C chain | 714 |
| 13337 | -3.7865 | 1.077126 | 1.71E-04 | 0.006053 | 0.239332 | H200004135 | MFNG | MFNG O-fucosylpeptide 3-beta-N-acetylglucosaminyltransferase | 4242 |
| 21048 | -1.7848 | 5.402291 | 2.83E-02 | 0.063046 | 0.239424 | H200011816 | VSIG2 | V-set and immunoglobulin domain containing 2 | 23584 |
| 2340 | -4.5108 | 0.883497 | 4.80E-05 | 0.004564 | 0.240234 | H200002648 | ULK4 | unc-51-like kinase 4 (C. elegans) | 54986 |
| 18097 | -3.2005 | 4.358736 | 6.15E-04 | 0.009361 | 0.240255 | H200012916 | ABI3 | ABI family, member 3 | 51225 |
| 5725 | -3.4244 | 0.975204 | 3.69E-04 | 0.007696 | 0.240551 | H200011452 | NA | NA | - |
| 19631 | -2.4127 | 1.458055 | 4.73E-03 | 0.023286 | 0.240869 | H200004436 | KIAA0125 | KIAA0125 | 9834 |
| 7505 | -1.856 | 3.043394 | 2.31E-02 | 0.055922 | 0.241239 | H200009218 | LRRC61 | leucine rich repeat containing 61 | 65999 |
| 8465 | -1.9757 | 1.658434 | 1.64E-02 | 0.046187 | 0.241362 | H200011510 | KMO | kynurenine 3-monooxygenase (kynurenine 3-hydroxylase) | 8564 |
| 14462 | -3.8679 | 0.91056 | 1.46E-04 | 0.005857 | 0.241593 | H200014033 | NA | NA | - |
| 17646 | -2.8947 | 1.454985 | 1.30E-03 | 0.012515 | 0.241959 | H200013313 | SAMD1 | sterile alpha motif domain containing 1 | 90378 |
| 5978 | -2.5496 | 20.00975 | 3.25E-03 | 0.019456 | 0.24225 | H200001863 | TYROBP | TYRO protein tyrosine kinase binding protein | 7305 |
| 19464 | -1.8444 | 1.794422 | 2.39E-02 | 0.057006 | 0.242468 | H200018039 | NA | NA | - |
| 20112 | -2.0695 | 2.431039 | 1.26E-02 | 0.039634 | 0.24258 | H200006158 | NPPA | natriuretic peptide A | 4878 |
| 17146 | -2.5224 | 1.958934 | 3.49E-03 | 0.02018 | 0.242686 | H200011354 | KIF9 | kinesin family member 9 | 64147 |
| 1068 | -2.5313 | 2.888457 | 3.41E-03 | 0.019867 | 0.243284 | H200007362 | CCDC19 | coiled-coil domain containing 19 | 25790 |
| 534 | -3.4977 | 2.344741 | 3.13E-04 | 0.007429 | 0.243415 | H200003669 | PIK3IP1 | phosphoinositide-3-kinase interacting protein 1 | 113791 |
| 9857 | -2.0631 | 1.788277 | 1.28E-02 | 0.040059 | 0.24423 | H200012496 | DEFB126 | defensin, beta 126 | 81623 |
| 1513 | -1.9695 | 0.434674 | 1.67E-02 | 0.046701 | 0.244556 | H200007047 | XIST | X inactive specific transcript (non-protein coding) | 7503 |
| 12706 | -2.6486 | 2.834255 | 2.48E-03 | 0.017043 | 0.244575 | H200017690 | C5AR2 | complement component 5a receptor 2 | 27202 |
| 14789 | -3.5983 | 2.276183 | 2.49E-04 | 0.00677 | 0.244799 | H200007744 | BLNK | B-cell linker | 29760 |
| 20103 | -1.8924 | 1.85442 | 2.08E-02 | 0.052624 | 0.244807 | H200005754 | C19orf21 | chromosome 19 open reading frame 21 | 126353 |
| 6447 | -2.3593 | 9.852839 | 5.50E-03 | 0.02527 | 0.245728 | H200002320 | HOPX | HOP homeobox | 84525 |
| 9602 | -6.9036 | 0.693999 | 1.30E-06 | 0.003715 | 0.245822 | H200000342 | CD1C | CD1c molecule | 911 |
| 11823 | -1.9736 | 2.54567 | 1.65E-02 | 0.046351 | 0.246156 | H200018884 | FANK1 | fibronectin type III and ankyrin repeat domains 1 | 92565 |
| 8067 | -2.337 | 1.796158 | 5.85E-03 | 0.026174 | 0.246307 | H200014277 | OSCP1 | organic solute carrier partner 1 | 127700 |
| 19258 | -5.4517 | 1.039489 | 1.14E-05 | 0.003715 | 0.246656 | H200008467 | NA | NA | - |
| 3184 | -2.5961 | 2.016979 | 2.86E-03 | 0.018254 | 0.246808 | H200020947 | NA | NA | - |
| 13186 | -2.4128 | 1.98132 | 4.73E-03 | 0.023286 | 0.247019 | H200018628 | NA | NA | - |
| 2629 | -2.5576 | 1.730217 | 3.18E-03 | 0.019203 | 0.247086 | H200016630 | NA | NA | - |
| 20687 | -2.3296 | 1.747181 | 5.99E-03 | 0.026392 | 0.247425 | H200015895 | PCDHA10 | protocadherin alpha 10 | 56139 |
| 8123 | -1.9803 | 1.481905 | 1.62E-02 | 0.045841 | 0.248475 | H200016937 | FBXO40 | F-box protein 40 | 51725 |
| 18793 | -2.8512 | 1.735897 | 1.46E-03 | 0.013131 | 0.248561 | H200005968 | SPOCK2 | sparc/osteonectin, cwcv and kazal-like domains proteoglycan (testican) 2 | 9806 |
| 6449 | -2.4492 | 2.035044 | 4.28E-03 | 0.02224 | 0.24869 | H200002344 | RCSD1 | RCSD domain containing 1 | 92241 |
| 9610 | -2.6051 | 1.276529 | 2.79E-03 | 0.018002 | 0.248716 | H200000722 | CD3E | CD3e molecule, epsilon (CD3-TCR complex) | 916 |
| 7261 | -3.7484 | 0.553041 | 1.84E-04 | 0.006172 | 0.249117 | H200019349 | FTHL17 | ferritin, heavy polypeptide-like 17 | 53940 |
| 1821 | -2.7686 | 0.76125 | 1.80E-03 | 0.014707 | 0.249125 | H2NC000006 | NA | NA | - |
| 11335 | -5.0266 | 0.792807 | 2.16E-05 | 0.003794 | 0.250398 | H200017269 | TREX1 | three prime repair exonuclease 1 | 11277 |
| 21399 | -2.791 | 1.683079 | 1.70E-03 | 0.014256 | 0.250684 | H200006811 | FBXO25 | F-box protein 25 | 26260 |
| 12901 | -4.2452 | 0.877885 | 7.62E-05 | 0.005085 | 0.250923 | H200004990 | LIMD2 | LIM domain containing 2 | 80774 |
| 7914 | -2.9313 | 1.309544 | 1.18E-03 | 0.012028 | 0.251324 | H200007039 | LAT | linker for activation of T cells | 27040 |
| 11390 | -4.6156 | 0.651211 | 4.13E-05 | 0.004304 | 0.252355 | H200019911 | CMTM7 | CKLF-like MARVEL transmembrane domain containing 7 | 112616 |
| 20784 | -2.1104 | 2.262978 | 1.12E-02 | 0.037522 | 0.252898 | H200021209 | NA | NA | - |
| 16148 | -2.074 | 2.643636 | 1.24E-02 | 0.03943 | 0.254002 | H200007453 | BTK | Bruton agammaglobulinemia tyrosine kinase | 695 |
| 19480 | -2.8804 | 34.57052 | 1.35E-03 | 0.012665 | 0.254064 | H200018799 | NA | NA | - |
| 16509 | -4.0022 | 0.994338 | 1.15E-04 | 0.005439 | 0.254154 | H200002709 | ARRB2 | arrestin, beta 2 | 409 |
| 155 | -2.2199 | 1.503234 | 8.18E-03 | 0.031449 | 0.254929 | H200007344 | RENBP | renin binding protein | 5973 |
| 923 | -2.7203 | 1.30607 | 2.05E-03 | 0.015644 | 0.254987 | H200000516 | NA | NA | - |
| 20244 | -1.9609 | 1.906939 | 1.71E-02 | 0.047374 | 0.255456 | H200013372 | NA | NA | - |
| 13134 | -1.936 | 1.425297 | 1.84E-02 | 0.049255 | 0.255999 | H200016016 | NA | NA | - |
| 8986 | -1.8606 | 5.201037 | 2.28E-02 | 0.055447 | 0.256061 | H200014651 | CH25H | cholesterol 25-hydroxylase | 9023 |
| 3352 | -1.8632 | 4.650071 | 2.26E-02 | 0.055218 | 0.256064 | H200007220 | NA | NA | - |
| 18264 | -2.161 | 2.025628 | 9.68E-03 | 0.034648 | 0.256172 | H200020890 | NA | NA | - |
| 6387 | -2.9332 | 1.212909 | 1.18E-03 | 0.012 | 0.256312 | H200021261 | CLEC4D | C-type lectin domain family 4, member D | 338339 |
| 18528 | -2.6293 | 1.028605 | 2.62E-03 | 0.017435 | 0.25643 | H200013419 | NA | NA | - |
| 15842 | -3.1185 | 0.82977 | 7.46E-04 | 0.009958 | 0.256567 | H200014554 | AGXT2 | alanine--glyoxylate aminotransferase 2 | 64902 |
| 19593 | -3.007 | 7.747065 | 9.81E-04 | 0.011076 | 0.256643 | H200002560 | AMICA1 | adhesion molecule, interacts with CXADR antigen 1 | 120425 |
| 20903 | -3.0832 | 1.216875 | 8.12E-04 | 0.010284 | 0.256816 | H200004958 | CDADC1 | cytidine and dCMP deaminase domain containing 1 | 81602 |
| 21184 | -2.4434 | 1.80151 | 4.35E-03 | 0.022348 | 0.257582 | H200018276 | NA | NA | - |
| 8308 | -2.9566 | 0.892483 | 1.11E-03 | 0.011684 | 0.257923 | H200003940 | CDKN2D | cyclin-dependent kinase inhibitor 2D (p19, inhibits CDK4) | 1032 |
| 14013 | -2.9303 | 0.977968 | 1.19E-03 | 0.012039 | 0.258109 | H200014454 | NPHS1 | nephrosis 1, congenital, Finnish type (nephrin) | 4868 |
| 6648 | -2.5097 | 6.202518 | 3.62E-03 | 0.020512 | 0.258455 | H200011826 | GIMAP6 | GTPase, IMAP family member 6 | 474344 |
| 11344 | -1.8494 | 1.213958 | 2.35E-02 | 0.056608 | 0.258649 | H200017655 | NA | NA | - |
| 21595 | -1.9306 | 2.692092 | 1.87E-02 | 0.049597 | 0.258695 | H200016263 | NA | NA | - |
| 2033 | -1.8934 | 1.854856 | 2.08E-02 | 0.052586 | 0.259477 | H200009885 | GGACT | gamma-glutamylamine cyclotransferase | 87769 |
| 18290 | -1.8954 | 1.141085 | 2.06E-02 | 0.052372 | 0.259595 | H200000499 | SAA4 | serum amyloid A4, constitutive | 6291 |
| 8107 | -2.8277 | 6.064999 | 1.55E-03 | 0.01361 | 0.26008 | H200016177 | NA | NA | - |
| 3650 | -2.7465 | 0.897926 | 1.91E-03 | 0.015139 | 0.260251 | H2NC000012 | NA | NA | - |
| 16578 | -4.4564 | 1.953081 | 5.25E-05 | 0.004712 | 0.260837 | H200006087 | SLA | Src-like-adaptor | 6503 |
| 6056 | -4.667 | 2.310644 | 3.79E-05 | 0.004216 | 0.261036 | H200005639 | LILRB4 | leukocyte immunoglobulin-like receptor, subfamily B (with TM and ITIM domains), member 4 | 11006 |
| 5448 | -4.7948 | 0.800768 | 3.14E-05 | 0.004036 | 0.261335 | H200020044 | TNFRSF13C | tumor necrosis factor receptor superfamily, member 13C | 115650 |
| 13828 | -5.1143 | 0.704066 | 1.93E-05 | 0.003715 | 0.261349 | H200005412 | SH3TC1 | SH3 domain and tetratricopeptide repeats 1 | 54436 |
| 7165 | -2.3784 | 3.920823 | 5.20E-03 | 0.02448 | 0.262297 | H200014789 | CXCR3 | chemokine (C-X-C motif) receptor 3 | 2833 |
| 13990 | -2.2575 | 2.90121 | 7.35E-03 | 0.029793 | 0.262344 | H200013320 | FAM50B | family with sequence similarity 50, member B | 26240 |
| 12242 | -3.1118 | 1.421046 | 7.58E-04 | 0.00998 | 0.262451 | H200017215 | NA | NA | - |
| 18693 | -3.8822 | 1.419995 | 1.41E-04 | 0.005857 | 0.2625 | H200000624 | NA | NA | - |
| 2750 | -1.8436 | 2.077483 | 2.39E-02 | 0.057107 | 0.262758 | H200000403 | CDX1 | caudal type homeobox 1 | 1044 |
| 17597 | -3.4881 | 0.947365 | 3.21E-04 | 0.007429 | 0.262795 | H200010731 | NFATC1 | nuclear factor of activated T-cells, cytoplasmic, calcineurin-dependent 1 | 4772 |
| 819 | -4.5913 | 0.898444 | 4.23E-05 | 0.004325 | 0.262799 | H200017319 | PTPN18 | protein tyrosine phosphatase, non-receptor type 18 (brain-derived) | 26469 |
| 11557 | -3.3451 | 3.074046 | 4.41E-04 | 0.008313 | 0.262816 | H200006320 | SDS | serine dehydratase | 10993 |
| 10526 | -4.2074 | 5.603556 | 8.04E-05 | 0.005233 | 0.263032 | H200000734 | CIITA | class II, major histocompatibility complex, transactivator | 4261 |
| 823 | -2.4716 | 1.263132 | 4.02E-03 | 0.02173 | 0.263295 | H200017367 | NA | NA | - |
| 4186 | -2.9831 | 0.695078 | 1.04E-03 | 0.011407 | 0.263733 | H200003620 | BEND5 | BEN domain containing 5 | 79656 |
| 2062 | -3.0698 | 1.399225 | 8.39E-04 | 0.010389 | 0.264098 | H200011079 | CASP9 | caspase 9, apoptosis-related cysteine peptidase | 842 |
| 12782 | -2.0689 | 11.30329 | 1.26E-02 | 0.039652 | 0.264372 | H200021158 | NA | NA | - |
| 8936 | -3.873 | 1.994559 | 1.44E-04 | 0.005857 | 0.264842 | H200012063 | KRT14 | keratin 14 | 3861 |
| 3017 | -4.0498 | 0.213041 | 1.07E-04 | 0.005439 | 0.264873 | H200012985 | CLCF1 | cardiotrophin-like cytokine factor 1 | 23529 |
| 1663 | -3.723 | 0.821892 | 1.93E-04 | 0.006231 | 0.265595 | H200013959 | FGF20 | fibroblast growth factor 20 | 26281 |
| 19877 | -1.9582 | 1.82736 | 1.73E-02 | 0.04755 | 0.265786 | H200016192 | RHOJ | ras homolog family member J | 57381 |
| 21189 | -2.4704 | 1.9129 | 4.03E-03 | 0.021768 | 0.266061 | H200018330 | NA | NA | - |
| 14831 | -1.8549 | 2.654917 | 2.32E-02 | 0.05601 | 0.267537 | H200009668 | GJA4 | gap junction protein, alpha 4, 37kDa | 2701 |
| 3435 | -1.7828 | 2.683019 | 2.85E-02 | 0.06323 | 0.267689 | H200011346 | SLC34A2 | solute carrier family 34 (sodium phosphate), member 2 | 10568 |
| 1605 | -2.3456 | 2.090176 | 5.71E-03 | 0.025809 | 0.268094 | H200011275 | PRB4 | proline-rich protein BstNI subfamily 4 | 5545 |
| 4151 | -2.4029 | 1.203466 | 4.86E-03 | 0.023602 | 0.268597 | H200001786 | ALDH1L1 | aldehyde dehydrogenase 1 family, member L1 | 10840 |
| 19893 | -3.8363 | 1.107996 | 1.55E-04 | 0.005935 | 0.268619 | H200016952 | LAX1 | lymphocyte transmembrane adaptor 1 | 54900 |
| 7876 | -3.18 | 1.37905 | 6.46E-04 | 0.009443 | 0.268833 | H200005163 | SMPD2 | sphingomyelin phosphodiesterase 2, neutral membrane (neutral sphingomyelinase) | 6610 |
| 17960 | -4.989 | 0.458539 | 2.32E-05 | 0.003926 | 0.268897 | H200006450 | P2RX5 | purinergic receptor P2X, ligand-gated ion channel, 5 | 5026 |
| 4092 | -3.052 | 1.471251 | 8.75E-04 | 0.010542 | 0.268924 | H200020649 | NA | NA | - |
| 6486 | -2.1174 | 3.988498 | 1.10E-02 | 0.037142 | 0.269047 | H200004202 | SLAMF1 | signaling lymphocytic activation molecule family member 1 | 6504 |
| 14442 | -2.8315 | 1.053706 | 1.54E-03 | 0.013549 | 0.269133 | H200013225 | HIST1H1D | histone cluster 1, H1d | 3007 |
| 21346 | -2.2889 | 1.759614 | 6.71E-03 | 0.028043 | 0.269418 | H200004477 | LRMP | lymphoid-restricted membrane protein | 4033 |
| 16575 | -2.6962 | 1.227239 | 2.19E-03 | 0.016152 | 0.269575 | H200005773 | NA | NA | - |
| 16751 | -4.1927 | 0.782735 | 8.24E-05 | 0.005263 | 0.2701 | H200014133 | NA | NA | - |
| 4326 | -2.8902 | 2.099949 | 1.31E-03 | 0.012515 | 0.270883 | H200010128 | NA | NA | - |
| 21461 | -2.2264 | 1.520298 | 8.04E-03 | 0.031179 | 0.27116 | H200009827 | CLDN9 | claudin 9 | 9080 |
| 19325 | -5.5434 | 0.631338 | 9.97E-06 | 0.003715 | 0.271695 | H200011537 | ABTB1 | ankyrin repeat and BTB (POZ) domain containing 1 | 80325 |
| 6919 | -3.5318 | 0.944527 | 2.90E-04 | 0.007373 | 0.271803 | H200003033 | B3GALT4 | UDP-Gal:betaGlcNAc beta 1,3-galactosyltransferase, polypeptide 4 | 8705 |
| 15293 | -2.6657 | 3.051226 | 2.37E-03 | 0.016706 | 0.271807 | H200010119 | PPP1CB | protein phosphatase 1, catalytic subunit, beta isozyme | 5500 |
| 7537 | -3.3073 | 0.946589 | 4.80E-04 | 0.008593 | 0.272029 | H200010738 | OGG1 | 8-oxoguanine DNA glycosylase | 4968 |
| 2425 | -2.1348 | 2.052582 | 1.04E-02 | 0.036082 | 0.272871 | H200006798 | PLCD1 | phospholipase C, delta 1 | 5333 |
| 9794 | -4.9648 | 0.542392 | 2.39E-05 | 0.003926 | 0.272919 | H200009462 | ORAI2 | ORAI calcium release-activated calcium modulator 2 | 80228 |
| 4235 | -2.1432 | 9.125141 | 1.02E-02 | 0.035607 | 0.273933 | H200005918 | SYK | spleen tyrosine kinase | 6850 |
| 10870 | -2.3721 | 1.042231 | 5.30E-03 | 0.02471 | 0.27412 | H200017074 | OSBPL7 | oxysterol binding protein-like 7 | 114881 |
| 20918 | -2.3218 | 0.983771 | 6.12E-03 | 0.02668 | 0.274382 | H200005712 | MOCS2 | molybdenum cofactor synthesis 2 | 4338 |
| 6114 | -3.2733 | 1.398928 | 5.19E-04 | 0.008769 | 0.274743 | H200008323 | ANAPC16 | anaphase promoting complex subunit 16 | 119504 |
| 6564 | -2.5122 | 1.446732 | 3.59E-03 | 0.020489 | 0.275262 | H200007694 | GPR68 | G protein-coupled receptor 68 | 8111 |
| 4941 | -2.1601 | 2.324038 | 9.70E-03 | 0.034686 | 0.27528 | H200017639 | CLIC5 | chloride intracellular channel 5 | 53405 |
| 20010 | -2.9659 | 1.127915 | 1.08E-03 | 0.011546 | 0.275573 | H200000790 | RAB11FIP4 | RAB11 family interacting protein 4 (class II) | 84440 |
| 14924 | -2.3456 | 1.070178 | 5.71E-03 | 0.025809 | 0.275784 | H200014186 | PSG1 | pregnancy specific beta-1-glycoprotein 1 | 5669 |
| 11338 | -2.704 | 1.486665 | 2.14E-03 | 0.015956 | 0.27587 | H200017583 | FAM13C | family with sequence similarity 13, member C | 220965 |
| 6438 | -3.3025 | 2.626322 | 4.85E-04 | 0.008593 | 0.275998 | H200001922 | THEMIS2 | thymocyte selection associated family member 2 | 9473 |
| 16103 | -3.2975 | 1.241879 | 4.91E-04 | 0.008622 | 0.276314 | H200005215 | BCAS4 | breast carcinoma amplified sequence 4 | 55653 |
| 20288 | -2.5799 | 1.308835 | 2.99E-03 | 0.018549 | 0.277637 | H200015966 | HRH2 | histamine receptor H2 | 3274 |
| 13224 | -2.4117 | 1.152006 | 4.74E-03 | 0.023319 | 0.277699 | H200020220 | NA | NA | - |
| 17670 | -1.8956 | 2.759992 | 2.06E-02 | 0.052372 | 0.277877 | H200014453 | NA | NA | - |
| 4634 | -4.4977 | 1.137975 | 4.95E-05 | 0.004597 | 0.278172 | H200003157 | CYBASC3 | cytochrome b, ascorbate dependent 3 | 220002 |
| 4845 | -1.8619 | 2.28273 | 2.27E-02 | 0.055275 | 0.278186 | H200013079 | GSDMC | gasdermin C | 56169 |
| 4346 | -1.903 | 1.754125 | 2.02E-02 | 0.051882 | 0.278364 | H200011220 | LSM2 | LSM2 homolog, U6 small nuclear RNA associated (S. cerevisiae) | 57819 |
| 3818 | -1.8258 | 2.536634 | 2.52E-02 | 0.058791 | 0.278669 | H200007705 | NA | NA | - |
| 16553 | -3.2954 | 0.964026 | 4.94E-04 | 0.008627 | 0.278839 | H200004941 | LPXN | leupaxin | 9404 |
| 12219 | -1.98 | 1.600187 | 1.62E-02 | 0.045841 | 0.280895 | H200016093 | NKX6-1 | NK6 homeobox 1 | 4825 |
| 1597 | -5.1175 | 0.847367 | 1.92E-05 | 0.003715 | 0.281317 | H200010895 | CCDC88B | coiled-coil domain containing 88B | 283234 |
| 4341 | -2.0643 | 2.407764 | 1.27E-02 | 0.039974 | 0.281373 | H200010882 | DNAH9 | dynein, axonemal, heavy chain 9 | 1770 |
| 4344 | -2.2569 | 1.18725 | 7.36E-03 | 0.02983 | 0.281453 | H200010912 | NA | NA | 89231 |
| 11224 | -5.3473 | 0.696352 | 1.38E-05 | 0.003715 | 0.281641 | H200011955 | MYH14 | myosin, heavy chain 14, non-muscle | 79784 |
| 15332 | -4.6834 | 0.536252 | 3.73E-05 | 0.004216 | 0.282521 | H200012001 | VAV1 | vav 1 guanine nucleotide exchange factor | 7409 |
| 4369 | -2.1397 | 8.308101 | 1.03E-02 | 0.035828 | 0.282722 | H200012354 | TEKT1 | tektin 1 | 83659 |
| 24 | -3.2443 | 1.308821 | 5.57E-04 | 0.009016 | 0.283365 | H200000938 | FAM49A | family with sequence similarity 49, member A | 81553 |
| 20917 | -1.9522 | 1.493284 | 1.76E-02 | 0.048038 | 0.283503 | H200005410 | KCNA1 | potassium voltage-gated channel, shaker-related subfamily, member 1 (episodic ataxia with myokymia) | 3736 |
| 14734 | -3.7826 | 0.843043 | 1.72E-04 | 0.006053 | 0.283609 | H200005090 | IKZF1 | IKAROS family zinc finger 1 (Ikaros) | 10320 |
| 6436 | -2.0892 | 2.755041 | 1.19E-02 | 0.038613 | 0.284594 | H200001614 | SYNE1 | spectrin repeat containing, nuclear envelope 1 | 23345 |
| 13941 | -2.0173 | 1.375727 | 1.46E-02 | 0.043372 | 0.284994 | H200011034 | PRG2 | proteoglycan 2, bone marrow (natural killer cell activator, eosinophil granule major basic protein) | 5553 |
| 6076 | -4.4682 | 0.66695 | 5.15E-05 | 0.004703 | 0.285102 | H200006447 | CSK | c-src tyrosine kinase | 1445 |
| 14386 | -4.2508 | 0.864343 | 7.48E-05 | 0.005022 | 0.285123 | H200010565 | VASP | vasodilator-stimulated phosphoprotein | 7408 |
| 2008 | -3.2606 | 1.172184 | 5.35E-04 | 0.008903 | 0.285254 | H200008443 | MOB3A | MOB kinase activator 3A | 126308 |
| 14681 | -2.7048 | 0.935278 | 2.14E-03 | 0.015941 | 0.285263 | H200002756 | ADAMTSL3 | ADAMTS-like 3 | 57188 |
| 5084 | -5.3722 | 0.409993 | 1.34E-05 | 0.003715 | 0.285447 | H200002896 | C20orf196 | chromosome 20 open reading frame 196 | 149840 |
| 5194 | -1.986 | 5.053348 | 1.59E-02 | 0.04541 | 0.285638 | H200008192 | ADAM28 | ADAM metallopeptidase domain 28 | 10863 |
| 18827 | -1.9524 | 2.255753 | 1.76E-02 | 0.048019 | 0.285662 | H200007862 | KLRB1 | killer cell lectin-like receptor subfamily B, member 1 | 3820 |
| 20810 | -2.7534 | 1.013077 | 1.88E-03 | 0.015014 | 0.285787 | H200000440 | FGFBP1 | fibroblast growth factor binding protein 1 | 9982 |
| 3255 | -2.4147 | 5.349816 | 4.70E-03 | 0.023271 | 0.286034 | H200002654 | RARRES3 | retinoic acid receptor responder (tazarotene induced) 3 | 5920 |
| 14400 | -1.9346 | 1.616748 | 1.84E-02 | 0.049352 | 0.286159 | H200011017 | ADRB1 | adrenoceptor beta 1 | 153 |
| 12153 | -5.7828 | 0.145644 | 6.68E-06 | 0.003715 | 0.286346 | H200013029 | FBXO2 | F-box protein 2 | 26232 |
| 4281 | -6.0694 | 0.38552 | 3.99E-06 | 0.003715 | 0.286371 | H200008174 | MAST3 | microtubule associated serine/threonine kinase 3 | 23031 |
| 15744 | -3.8947 | 1.030664 | 1.38E-04 | 0.005806 | 0.286412 | H200009686 | P2RY10 | purinergic receptor P2Y, G-protein coupled, 10 | 27334 |
| 13653 | -3.3038 | 1.178046 | 4.84E-04 | 0.008593 | 0.286839 | H200019287 | PTCD1 | pentatricopeptide repeat domain 1 | 26024 |
| 16428 | -3.1179 | 1.257646 | 7.47E-04 | 0.009958 | 0.286863 | H200020753 | NA | NA | - |
| 9087 | -2.5623 | 1.363464 | 3.14E-03 | 0.019068 | 0.286888 | H200019277 | OR7E91P | olfactory receptor, family 7, subfamily E, member 91 pseudogene | 79315 |
| 1844 | -3.6542 | 0.864285 | 2.21E-04 | 0.006534 | 0.287131 | H200000795 | LSM10 | LSM10, U7 small nuclear RNA associated | 84967 |
| 8013 | -3.1301 | 1.480515 | 7.23E-04 | 0.009887 | 0.287141 | H200011641 | PREX1 | phosphatidylinositol-3,4,5-trisphosphate-dependent Rac exchange factor 1 | 57580 |
| 13213 | -1.8439 | 1.079679 | 2.39E-02 | 0.057071 | 0.287753 | H200019810 | AGFG2 | ArfGAP with FG repeats 2 | 3268 |
| 19141 | -3.064 | 3.07496 | 8.50E-04 | 0.010429 | 0.288439 | H200002797 | SKAP1 | src kinase associated phosphoprotein 1 | 8631 |
| 7934 | -3.7376 | 1.128185 | 1.89E-04 | 0.006183 | 0.288458 | H200007847 | CNN2 | calponin 2 | 1265 |
| 11118 | -2.5279 | 3.773075 | 3.44E-03 | 0.019984 | 0.288539 | H200006991 | NA | NA | - |
| 5011 | -1.9558 | 1.697454 | 1.74E-02 | 0.047803 | 0.288652 | H200021035 | NA | NA | - |
| 21691 | -1.8329 | 1.324215 | 2.47E-02 | 0.05798 | 0.289318 | H200020823 | LOC153910 | uncharacterized LOC153910 | 153910 |
| 5185 | -2.9457 | 0.43843 | 1.14E-03 | 0.011769 | 0.290123 | H200007806 | TCF7 | transcription factor 7 (T-cell specific, HMG-box) | 6932 |
| 5028 | -2.58 | 11.21366 | 2.99E-03 | 0.018549 | 0.290338 | H200000236 | LTB | lymphotoxin beta (TNF superfamily, member 3) | 4050 |
| 15709 | -5.0237 | 1.065754 | 2.18E-05 | 0.003794 | 0.290473 | H200008136 | PLEKHO1 | pleckstrin homology domain containing, family O member 1 | 51177 |
| 11046 | -3.8594 | 0.724749 | 1.48E-04 | 0.005878 | 0.291586 | H200003571 | SYTL1 | synaptotagmin-like 1 | 84958 |
| 153 | -2.3825 | 1.170769 | 5.14E-03 | 0.024326 | 0.292292 | H200007320 | ERC2-IT1 | ERC2 intronic transcript 1 (non-protein coding) | 711 |
| 9827 | -2.0938 | 1.799989 | 1.17E-02 | 0.038372 | 0.292449 | H200011000 | FPR2 | formyl peptide receptor 2 | 2358 |
| 14682 | -3.6352 | 0.964776 | 2.31E-04 | 0.006651 | 0.292851 | H200002762 | HSD17B14 | hydroxysteroid (17-beta) dehydrogenase 14 | 51171 |
| 11573 | -5.6326 | 1.425117 | 8.77E-06 | 0.003715 | 0.293013 | H200007080 | ITGB2 | integrin, beta 2 (complement component 3 receptor 3 and 4 subunit) | 3689 |
| 11607 | -2.4429 | 1.033637 | 4.35E-03 | 0.022348 | 0.293201 | H200008624 | RPL35 | ribosomal protein L35 | 11224 |
| 228 | -3.2948 | 1.9847 | 4.95E-04 | 0.008628 | 0.293441 | H200010770 | CD247 | CD247 molecule | 919 |
| 11540 | -2.5285 | 1.311071 | 3.44E-03 | 0.019964 | 0.293585 | H200005542 | SDCBP2 | syndecan binding protein (syntenin) 2 | 27111 |
| 12152 | -2.6681 | 3.353046 | 2.36E-03 | 0.016639 | 0.29391 | H200012727 | IL4I1 | interleukin 4 induced 1 | 259307 |
| 19809 | -4.257 | 0.847374 | 7.42E-05 | 0.005013 | 0.293926 | H200012820 | C17orf59 | chromosome 17 open reading frame 59 | 54785 |
| 18022 | -2.0211 | 1.921506 | 1.44E-02 | 0.043048 | 0.29398 | H200009466 | FLJ12334 | uncharacterized LOC400946 | 400946 |
| 19999 | -2.6304 | 0.938434 | 2.61E-03 | 0.017425 | 0.294474 | H200000072 | CYTIP | cytohesin 1 interacting protein | 9595 |
| 10432 | -2.1569 | 34.28034 | 9.79E-03 | 0.034786 | 0.294986 | H200017905 | HLA-DQA2 | major histocompatibility complex, class II, DQ alpha 2 | 3118 |
| 7094 | -2.121 | 9.43261 | 1.08E-02 | 0.036931 | 0.2951 | H200011375 | GIMAP6 | GTPase, IMAP family member 6 | 474344 |
| 11302 | -3.0787 | 4.209091 | 8.20E-04 | 0.010325 | 0.295223 | H200015731 | CCL5 | chemokine (C-C motif) ligand 5 | 6352 |
| 17541 | -2.6674 | 1.354762 | 2.36E-03 | 0.016666 | 0.295719 | H200008071 | DGKA | diacylglycerol kinase, alpha 80kDa | 1606 |
| 2376 | -1.8864 | 4.518473 | 2.12E-02 | 0.053131 | 0.295725 | H200004500 | HS3ST1 | heparan sulfate (glucosamine) 3-O-sulfotransferase 1 | 9957 |
| 20715 | -1.9124 | 1.457172 | 1.97E-02 | 0.05113 | 0.295759 | H200017415 | NA | NA | - |
| 3912 | -1.9472 | 1.579227 | 1.78E-02 | 0.048351 | 0.296073 | H200011957 | RIMBP3 | RIMS binding protein 3 | 85376 |
| 11492 | -5.1956 | 0.500243 | 1.69E-05 | 0.003715 | 0.296096 | H200003262 | RNASE6 | ribonuclease, RNase A family, k6 | 6039 |
| 15950 | -3.1269 | 0.812496 | 7.29E-04 | 0.009909 | 0.296222 | H200019542 | NA | NA | - |
| 17342 | -1.9021 | 2.325139 | 2.02E-02 | 0.051949 | 0.296329 | H200020806 | NA | NA | - |
| 17178 | -2.9587 | 0.973952 | 1.10E-03 | 0.011664 | 0.296333 | H200012874 | POLL | polymerase (DNA directed), lambda | 27343 |
| 8953 | -2.7478 | 0.964609 | 1.91E-03 | 0.015124 | 0.296519 | H200013125 | FXYD7 | FXYD domain containing ion transport regulator 7 | 53822 |
| 9767 | -3.0742 | 1.172433 | 8.28E-04 | 0.010354 | 0.296867 | H200008292 | NA | NA | - |
| 12035 | -2.9474 | 1.258332 | 1.13E-03 | 0.011769 | 0.297048 | H200007353 | TTTY1 | testis-specific transcript, Y-linked 1 (non-protein coding) | 50858 |
| 8915 | -2.6825 | 19.28372 | 2.27E-03 | 0.01629 | 0.297141 | H200011249 | SCAF1 | SR-related CTD-associated factor 1 | 58506 |
| 18203 | -3.7112 | 6.443138 | 1.97E-04 | 0.006274 | 0.297716 | H200018164 | NA | NA | - |
| 18154 | -1.8474 | 1.441854 | 2.37E-02 | 0.056748 | 0.297851 | H200015878 | OR10H2 | olfactory receptor, family 10, subfamily H, member 2 | 26538 |
| 6133 | -3.6984 | 0.878906 | 2.02E-04 | 0.006332 | 0.298189 | H200009409 | WDR96 | WD repeat domain 96 | 80217 |
| 9272 | -2.6867 | 3.034541 | 2.24E-03 | 0.01621 | 0.298254 | H200006161 | PLCG2 | phospholipase C, gamma 2 (phosphatidylinositol-specific) | 5336 |
| 9707 | -1.9556 | 1.694449 | 1.74E-02 | 0.047803 | 0.298343 | H200005300 | HOXA2 | homeobox A2 | 3199 |
| 18852 | -3.129 | 1.069407 | 7.26E-04 | 0.009887 | 0.298657 | H200009340 | NA | NA | - |
| 10234 | -2.25 | 0.844922 | 7.51E-03 | 0.030151 | 0.299563 | H200008429 | ZNF671 | zinc finger protein 671 | 79891 |
| 11820 | -3.0895 | 0.819242 | 8.00E-04 | 0.010245 | 0.299876 | H200018842 | NA | NA | - |
| 8477 | -1.8647 | 1.345011 | 2.25E-02 | 0.055037 | 0.300389 | H200011938 | SCEL | sciellin | 8796 |
| 19348 | -2.0509 | 1.375602 | 1.32E-02 | 0.040846 | 0.300595 | H200012671 | NIPAL2 | NIPA-like domain containing 2 | 79815 |
| 2712 | -3.1218 | 1.235132 | 7.40E-04 | 0.009945 | 0.300673 | H200020460 | PPM1M | protein phosphatase, Mg2+/Mn2+ dependent, 1M | 132160 |
| 2330 | -2.0708 | 1.136068 | 1.25E-02 | 0.039589 | 0.300728 | H200002244 | NA | NA | - |
| 3786 | -2.0071 | 2.297953 | 1.50E-02 | 0.043914 | 0.300885 | H200006185 | ABP1 | amiloride binding protein 1 (amine oxidase (copper-containing)) | 26 |
| 7641 | -3.8476 | 0.461308 | 1.51E-04 | 0.005916 | 0.3011 | H200015678 | SCRN2 | secernin 2 | 90507 |
| 557 | -2.5267 | 1.127986 | 3.45E-03 | 0.020025 | 0.301576 | H200004803 | NA | NA | - |
| 4117 | -3.4813 | 0.809087 | 3.26E-04 | 0.007449 | 0.301813 | H200000242 | ARID5A | AT rich interactive domain 5A (MRF1-like) | 10865 |
| 11640 | -2.5928 | 1.015206 | 2.89E-03 | 0.018355 | 0.302023 | H200010150 | KLF1 | Kruppel-like factor 1 (erythroid) | 10661 |
| 10401 | -5.1894 | 0.754296 | 1.72E-05 | 0.003715 | 0.302204 | H200016403 | TNFRSF1B | tumor necrosis factor receptor superfamily, member 1B | 7133 |
| 9878 | -1.9377 | 1.455054 | 1.83E-02 | 0.049149 | 0.302302 | H200013594 | UBR1 | ubiquitin protein ligase E3 component n-recognin 1 | 197131 |
| 20265 | -2.1048 | 1.248494 | 1.14E-02 | 0.037836 | 0.302411 | H200014512 | UTS2R | urotensin 2 receptor | 2837 |
| 5190 | -2.7766 | 1.185727 | 1.77E-03 | 0.01453 | 0.302493 | H200007860 | TRIM69 | tripartite motif containing 69 | 140691 |
| 18631 | -2.723 | 1.026015 | 2.04E-03 | 0.015612 | 0.302575 | H200018793 | PACRG | PARK2 co-regulated | 135138 |
| 19514 | -1.7982 | 1.239499 | 2.72E-02 | 0.061667 | 0.302657 | H200020627 | NA | NA | - |
| 2483 | -2.1571 | 1.691637 | 9.79E-03 | 0.034784 | 0.303152 | H200009482 | COTL1 | coactosin-like 1 (Dictyostelium) | 23406 |
| 15096 | -2.9958 | 5.839161 | 1.00E-03 | 0.011182 | 0.303708 | H200000649 | CSTA | cystatin A (stefin A) | 1475 |
| 8267 | -5.7579 | 0.377534 | 6.88E-06 | 0.003715 | 0.303772 | H200002034 | SIGIRR | single immunoglobulin and toll-interleukin 1 receptor (TIR) domain | 59307 |
| 17118 | -1.8031 | 1.678515 | 2.69E-02 | 0.061217 | 0.303983 | H200010166 | HIST2H4A | histone cluster 2, H4a | 8370 |
| 7240 | -3.8674 | 1.111377 | 1.46E-04 | 0.005857 | 0.304368 | H200018239 | NA | NA | 79164 |
| 19358 | -2.2435 | 1.479715 | 7.65E-03 | 0.030476 | 0.304625 | H200013075 | NA | NA | - |
| 6680 | -2.1689 | 2.420784 | 9.46E-03 | 0.034201 | 0.304811 | H200013346 | CD96 | CD96 molecule | 10225 |
| 733 | -2.0162 | 0.685227 | 1.46E-02 | 0.043443 | 0.305441 | H200013163 | CCDC71 | coiled-coil domain containing 71 | 64925 |
| 4631 | -2.9785 | 1.041174 | 1.05E-03 | 0.011443 | 0.305535 | H200002843 | PNKD | paroxysmal nonkinesigenic dyskinesia | 25953 |
| 6267 | -2.5044 | 1.420377 | 3.67E-03 | 0.020681 | 0.306726 | H200015561 | RBM38 | RNA binding motif protein 38 | 55544 |
| 9829 | -2.3767 | 1.443959 | 5.23E-03 | 0.024556 | 0.307495 | H200011308 | PLD2 | phospholipase D2 | 5338 |
| 11575 | -2.7122 | 11.05766 | 2.10E-03 | 0.015819 | 0.308093 | H200007104 | CD74 | CD74 molecule, major histocompatibility complex, class II invariant chain | 972 |
| 21638 | -4.8173 | 0.614577 | 2.98E-05 | 0.004028 | 0.308129 | H200018205 | C22orf32 | chromosome 22 open reading frame 32 | 91689 |
| 16302 | -3.12 | 0.897333 | 7.43E-04 | 0.009958 | 0.308643 | H200014697 | FCRL2 | Fc receptor-like 2 | 79368 |
| 17265 | -1.9424 | 0.827795 | 1.80E-02 | 0.048772 | 0.309125 | H200017036 | NA | NA | - |
| 1541 | -2.3636 | 0.807806 | 5.43E-03 | 0.025063 | 0.30918 | H200008235 | NA | NA | 158014 |
| 7862 | -3.4375 | 1.01628 | 3.59E-04 | 0.007636 | 0.309258 | H200004427 | SP110 | SP110 nuclear body protein | 3431 |
| 8708 | -5.2185 | 0.825799 | 1.65E-05 | 0.003715 | 0.309403 | H200001375 | PLEKHO2 | pleckstrin homology domain containing, family O member 2 | 80301 |
| 801 | -2.1371 | 1.172686 | 1.04E-02 | 0.035925 | 0.309659 | H200016535 | TMIGD2 | transmembrane and immunoglobulin domain containing 2 | 126259 |
| 18872 | -5.4427 | 0.481705 | 1.17E-05 | 0.003715 | 0.309751 | H200010474 | PRKD2 | protein kinase D2 | 25865 |
| 5272 | -3.7383 | 0.650301 | 1.88E-04 | 0.006183 | 0.309769 | H200011684 | SLC52A1 | solute carrier family 52, riboflavin transporter, member 1 | 55065 |
| 17727 | -2.6461 | 4.068866 | 2.50E-03 | 0.017063 | 0.309791 | H200017119 | TARP | TCR gamma alternate reading frame protein | 445347 |
| 13285 | -2.9562 | 4.587111 | 1.11E-03 | 0.011684 | 0.309951 | H200001807 | C1QA | complement component 1, q subcomponent, A chain | 712 |
| 21100 | -2.0388 | 1.382759 | 1.37E-02 | 0.041794 | 0.310483 | H200014144 | SOX3 | SRY (sex determining region Y)-box 3 | 6658 |
| 20922 | -1.8113 | 1.707735 | 2.63E-02 | 0.060266 | 0.310757 | H200005760 | NA | NA | - |
| 20006 | -2.2785 | 2.579786 | 6.91E-03 | 0.028579 | 0.310896 | H200000452 | IL2RA | interleukin 2 receptor, alpha | 3559 |
| 15252 | -3.9441 | 0.969571 | 1.27E-04 | 0.005667 | 0.311037 | H200008201 | ITGAL | integrin, alpha L (antigen CD11A (p180), lymphocyte function-associated antigen 1; alpha polypeptide) | 3683 |
| 11864 | -2.0974 | 0.937941 | 1.16E-02 | 0.038197 | 0.311242 | H200020790 | FLJ31713 | uncharacterized protein FLJ31713 | 158263 |
| 5948 | -2.7584 | 1.070052 | 1.85E-03 | 0.014863 | 0.311425 | H200000367 | FCER2 | Fc fragment of IgE, low affinity II, receptor for (CD23) | 2208 |
| 15908 | -2.8164 | 1.413689 | 1.60E-03 | 0.013809 | 0.311437 | H200017618 | FOXP2 | forkhead box P2 | 93986 |
| 20541 | -2.395 | 2.088495 | 4.96E-03 | 0.023937 | 0.311455 | H200007933 | CECR1 | cat eye syndrome chromosome region, candidate 1 | 51816 |
| 17827 | -3.4582 | 0.973859 | 3.43E-04 | 0.007546 | 0.311498 | H200000304 | DUSP2 | dual specificity phosphatase 2 | 1844 |
| 17540 | -3.7393 | 1.172182 | 1.88E-04 | 0.006183 | 0.311589 | H200008065 | ACVRL1 | activin A receptor type II-like 1 | 94 |
| 20284 | -3.7191 | 1.009287 | 1.94E-04 | 0.006235 | 0.312043 | H200015628 | RASSF5 | Ras association (RalGDS/AF-6) domain family member 5 | 83593 |
| 2742 | -3.6624 | 0.65189 | 2.18E-04 | 0.006496 | 0.3122 | H200000023 | ABHD14B | abhydrolase domain containing 14B | 84836 |
| 2529 | -2.8878 | 0.710727 | 1.32E-03 | 0.012529 | 0.31223 | H200011738 | PLEKHG2 | pleckstrin homology domain containing, family G (with RhoGef domain) member 2 | 64857 |
| 9727 | -2.0987 | 1.131333 | 1.15E-02 | 0.038159 | 0.312383 | H200006392 | CDX2 | caudal type homeobox 2 | 1045 |
| 17071 | -4.1306 | 1.517612 | 9.16E-05 | 0.0054 | 0.312504 | H200007892 | RUNX3 | runt-related transcription factor 3 | 864 |
| 18008 | -3.9477 | 0.826876 | 1.26E-04 | 0.005667 | 0.312515 | H200008730 | WIF1 | WNT inhibitory factor 1 | 11197 |
| 11531 | -3.1662 | 0.879579 | 6.67E-04 | 0.009587 | 0.312562 | H200005156 | CAPSL | calcyphosine-like | 133690 |
| 9400 | -2.2153 | 3.632781 | 8.29E-03 | 0.031626 | 0.312667 | H200012241 | TMEM154 | transmembrane protein 154 | 201799 |
| 19958 | -6.1777 | 0.406587 | 3.29E-06 | 0.003715 | 0.312817 | H200020010 | FLJ22447 | uncharacterized LOC400221 | 400221 |
| 18561 | -3.3142 | 0.992461 | 4.73E-04 | 0.008585 | 0.312959 | H200014993 | HSH2D | hematopoietic SH2 domain containing | 84941 |
| 7283 | -3.9163 | 0.56826 | 1.33E-04 | 0.005801 | 0.31298 | H200020465 | FAM102A | family with sequence similarity 102, member A | 399665 |
| 12330 | -2.7014 | 0.895953 | 2.16E-03 | 0.015982 | 0.313026 | H2NC000009 | NA | NA | - |
| 14964 | -1.8575 | 1.898243 | 2.30E-02 | 0.055802 | 0.313029 | H200016086 | NA | NA | - |
| 20045 | -2.812 | 0.957591 | 1.61E-03 | 0.013872 | 0.313058 | H200002690 | LYVE1 | lymphatic vessel endothelial hyaluronan receptor 1 | 10894 |
| 13309 | -2.7652 | 0.922488 | 1.82E-03 | 0.014755 | 0.313334 | H200002947 | LHPP | phospholysine phosphohistidine inorganic pyrophosphate phosphatase | 64077 |
| 10563 | -3.2809 | 1.302855 | 5.11E-04 | 0.00873 | 0.314185 | H200002604 | TMEM40 | transmembrane protein 40 | 55287 |
| 9403 | -2.0606 | 1.513854 | 1.29E-02 | 0.040197 | 0.314409 | H200012567 | PRSS22 | protease, serine, 22 | 64063 |
| 3231 | -3.1111 | 2.776206 | 7.59E-04 | 0.00998 | 0.314415 | H200001514 | SLCO2B1 | solute carrier organic anion transporter family, member 2B1 | 11309 |
| 1169 | -2.0371 | 2.156626 | 1.38E-02 | 0.041886 | 0.314673 | H200012272 | STK17B | serine/threonine kinase 17b | 9262 |
| 19203 | -1.8657 | 2.710709 | 2.25E-02 | 0.054973 | 0.314845 | H200005813 | IL7 | interleukin 7 | 3574 |
| 4678 | -1.9226 | 2.018044 | 1.91E-02 | 0.050194 | 0.31524 | H200005105 | NA | NA | - |
| 3851 | -3.3057 | 2.162094 | 4.81E-04 | 0.008593 | 0.315726 | H200009243 | NA | NA | - |
| 1627 | -1.9603 | 1.117826 | 1.72E-02 | 0.047387 | 0.315771 | H200012391 | NA | NA | - |
| 17061 | -3.8127 | 0.667172 | 1.62E-04 | 0.005936 | 0.3164 | H200007204 | FAM212A | family with sequence similarity 212, member A | 389119 |
| 20679 | -2.3883 | 0.874576 | 5.06E-03 | 0.024149 | 0.316555 | H200015509 | ZAP70 | zeta-chain (TCR) associated protein kinase 70kDa | 7535 |
| 14987 | -1.9219 | 1.49041 | 1.91E-02 | 0.050264 | 0.31685 | H200017220 | COX4I2 | cytochrome c oxidase subunit IV isoform 2 (lung) | 84701 |
| 15524 | -1.9658 | 0.683029 | 1.69E-02 | 0.047024 | 0.316895 | H200021121 | NA | NA | 219848 |
| 10185 | -2.3732 | 2.097603 | 5.28E-03 | 0.024655 | 0.316898 | H200006143 | IL2RB | interleukin 2 receptor, beta | 3560 |
| 10306 | -3.4312 | 0.527113 | 3.64E-04 | 0.007655 | 0.317181 | H200011849 | PARD6A | par-6 partitioning defective 6 homolog alpha (C. elegans) | 50855 |
| 2336 | -4.4831 | 0.8967 | 5.05E-05 | 0.00465 | 0.317455 | H200002600 | UBA7 | ubiquitin-like modifier activating enzyme 7 | 7318 |
| 18577 | -2.0517 | 1.013012 | 1.32E-02 | 0.040821 | 0.317659 | H200016079 | HIST1H1E | histone cluster 1, H1e | 3008 |
| 9552 | -3.1667 | 1.327721 | 6.66E-04 | 0.009584 | 0.317686 | H200019461 | NA | NA | - |
| 9267 | -2.8658 | 0.642919 | 1.40E-03 | 0.012969 | 0.318141 | H200006107 | TSC22D3 | TSC22 domain family, member 3 | 1831 |
| 18801 | -2.9317 | 1.025724 | 1.18E-03 | 0.012028 | 0.3186 | H200006656 | SELPLG | selectin P ligand | 6404 |
| 8326 | -1.9624 | 1.032902 | 1.71E-02 | 0.047295 | 0.318819 | H200004724 | ATP10A | ATPase, class V, type 10A | 57194 |
| 19333 | -3.5163 | 4.433788 | 3.00E-04 | 0.007429 | 0.31884 | H200011917 | COX7A1 | cytochrome c oxidase subunit VIIa polypeptide 1 (muscle) | 1346 |
| 19110 | -3.4166 | 0.566204 | 3.74E-04 | 0.007706 | 0.318911 | H200001295 | GNG7 | guanine nucleotide binding protein (G protein), gamma 7 | 2788 |
| 8369 | -3.2795 | 1.015689 | 5.13E-04 | 0.00873 | 0.319489 | H200006950 | POLD4 | polymerase (DNA-directed), delta 4, accessory subunit | 57804 |
| 16417 | -2.4506 | 0.808824 | 4.26E-03 | 0.022206 | 0.319521 | H200020343 | A1BG | alpha-1-B glycoprotein | 1 |
| 431 | -2.096 | 1.48421 | 1.16E-02 | 0.038245 | 0.319771 | H200020312 | TRIML1 | tripartite motif family-like 1 | 339976 |
| 20342 | -3.7959 | 0.735943 | 1.67E-04 | 0.006005 | 0.319805 | H200018692 | ANGPTL6 | angiopoietin-like 6 | 83854 |
| 11180 | -4.1584 | 0.589588 | 8.65E-05 | 0.005263 | 0.320178 | H200010007 | EFHD2 | EF-hand domain family, member D2 | 79180 |
| 8032 | -1.953 | 1.398607 | 1.75E-02 | 0.047981 | 0.320213 | H200012431 | RAB3B | RAB3B, member RAS oncogene family | 5865 |
| 5328 | -3.937 | 1.062939 | 1.29E-04 | 0.005699 | 0.320635 | H200014344 | EBI3 | Epstein-Barr virus induced 3 | 10148 |
| 19006 | -3.1153 | 1.009189 | 7.52E-04 | 0.009958 | 0.320846 | H200017700 | RETN | resistin | 56729 |
| 20965 | -2.286 | 0.838242 | 6.77E-03 | 0.028175 | 0.320865 | H200007690 | NA | NA | - |
| 2308 | -2.1385 | 1.260869 | 1.03E-02 | 0.03587 | 0.320943 | H200001128 | MUC13 | mucin 13, cell surface associated | 56667 |
| 3173 | -2.0683 | 1.102685 | 1.26E-02 | 0.03968 | 0.321299 | H200020537 | NA | NA | - |
| 4979 | -2.8904 | 1.553602 | 1.31E-03 | 0.012515 | 0.321338 | H200019515 | IRF5 | interferon regulatory factor 5 | 3663 |
| 6898 | -1.9006 | 1.788208 | 2.03E-02 | 0.052104 | 0.321458 | H200002207 | 1-Mar | membrane-associated ring finger (C3HC4) 1, E3 ubiquitin protein ligase | 55016 |
| 20634 | -2.2684 | 1.189054 | 7.13E-03 | 0.029163 | 0.321653 | H200012897 | RASGRP4 | RAS guanyl releasing protein 4 | 115727 |
| 342 | -2.7195 | 0.922427 | 2.05E-03 | 0.015644 | 0.321717 | H200016114 | KRT36 | keratin 36 | 8689 |
| 11404 | -2.2447 | 1.208917 | 7.63E-03 | 0.030457 | 0.321853 | H200020647 | HPS4 | Hermansky-Pudlak syndrome 4 | 89781 |
| 12357 | -4.8273 | 0.94209 | 2.93E-05 | 0.004028 | 0.32197 | H200001012 | GMFG | glia maturation factor, gamma | 9535 |
| 6258 | -4.6276 | 0.558333 | 4.03E-05 | 0.004281 | 0.322218 | H200015163 | NA | NA | 441881 |
| 20211 | -3.1281 | 0.891835 | 7.27E-04 | 0.009889 | 0.322358 | H200011786 | SBF1 | SET binding factor 1 | 6305 |
| 2392 | -2.1433 | 1.14567 | 1.02E-02 | 0.035607 | 0.322382 | H200005260 | LRRC6 | leucine rich repeat containing 6 | 23639 |
| 13264 | -2.9347 | 0.917929 | 1.17E-03 | 0.011986 | 0.32264 | H200000697 | PRKCG | protein kinase C, gamma | 5582 |
| 11585 | -3.0845 | 1.081048 | 8.10E-04 | 0.010284 | 0.323113 | H200007792 | CCL23 | chemokine (C-C motif) ligand 23 | 6368 |
| 14290 | -3.1541 | 0.491523 | 6.85E-04 | 0.00973 | 0.323565 | H200006005 | CLU | clusterin | 1191 |
| 1740 | -3.4939 | 0.160651 | 3.17E-04 | 0.007429 | 0.323816 | H200017717 | SDR39U1 | short chain dehydrogenase/reductase family 39U, member 1 | 56948 |
| 17777 | -1.8471 | 1.699327 | 2.37E-02 | 0.056781 | 0.323882 | H200019423 | NA | NA | - |
| 19100 | -2.5957 | 0.779124 | 2.86E-03 | 0.018254 | 0.323993 | H200000891 | TBC1D10A | TBC1 domain family, member 10A | 83874 |
| 8059 | -3.7692 | 1.632184 | 1.77E-04 | 0.00608 | 0.32424 | H200013897 | FRZB | frizzled-related protein | 2487 |
| 20582 | -3.0277 | 0.894401 | 9.30E-04 | 0.01078 | 0.324409 | H200010195 | MADD | MAP-kinase activating death domain | 8567 |
| 6219 | -2.207 | 1.022944 | 8.49E-03 | 0.032081 | 0.32465 | H200013281 | EXPH5 | exophilin 5 | 23086 |
| 11037 | -6.0996 | 0.458708 | 3.69E-06 | 0.003715 | 0.324939 | H200003185 | RGS19 | regulator of G-protein signaling 19 | 10287 |
| 108 | -2.9881 | 0.672045 | 1.02E-03 | 0.011287 | 0.325094 | H200005070 | VIP | vasoactive intestinal peptide | 7432 |
| 12635 | -3.4677 | 1.02607 | 3.36E-04 | 0.007529 | 0.32531 | H200014288 | FLOT2 | flotillin 2 | 2319 |
| 16796 | -1.9302 | 1.606073 | 1.87E-02 | 0.049621 | 0.325634 | H200016371 | NA | NA | - |
| 12570 | -2.0017 | 1.254266 | 1.52E-02 | 0.044412 | 0.325821 | H200011230 | PLIN1 | perilipin 1 | 5346 |
| 13497 | -2.9071 | 1.094621 | 1.26E-03 | 0.012286 | 0.326191 | H200011735 | ALKBH7 | alkB, alkylation repair homolog 7 (E. coli) | 84266 |
| 924 | -2.0842 | 0.89495 | 1.20E-02 | 0.038927 | 0.326471 | H200000522 | TPO | thyroid peroxidase | 7173 |
| 11122 | -1.8375 | 1.498937 | 2.43E-02 | 0.057629 | 0.326915 | H200007323 | KCNH1 | potassium voltage-gated channel, subfamily H (eag-related), member 1 | 3756 |
| 183 | -2.3667 | 1.046296 | 5.38E-03 | 0.02492 | 0.327372 | H200008532 | KLK2 | kallikrein-related peptidase 2 | 3817 |
| 1329 | -2.3557 | 1.068303 | 5.56E-03 | 0.025423 | 0.327575 | H200019872 | R3HDML | R3H domain containing-like | 140902 |
| 7864 | -2.9791 | 5.077878 | 1.05E-03 | 0.011443 | 0.328022 | H200004451 | S100A2 | S100 calcium binding protein A2 | 6273 |
| 2721 | -1.7773 | 1.234738 | 2.89E-02 | 0.06371 | 0.328352 | H200020858 | NA | NA | - |
| 20377 | -2.2253 | 0.854105 | 8.06E-03 | 0.031187 | 0.328781 | H200020592 | OR4D2 | olfactory receptor, family 4, subfamily D, member 2 | 124538 |
| 13050 | -2.0981 | 1.706786 | 1.16E-02 | 0.038171 | 0.329217 | H200012168 | STX11 | syntaxin 11 | 8676 |
| 13331 | -2.1214 | 1.117331 | 1.08E-02 | 0.036916 | 0.329261 | H200003779 | HHATL | hedgehog acyltransferase-like | 57467 |
| 16905 | -2.3301 | 0.960297 | 5.98E-03 | 0.026365 | 0.329276 | H200008489 | RPL5 | ribosomal protein L5 | 6125 |
| 1031 | -2.5473 | 1.471169 | 3.27E-03 | 0.019483 | 0.329569 | H200005504 | BATF3 | basic leucine zipper transcription factor, ATF-like 3 | 55509 |
| 10651 | -2.1866 | 1.221278 | 8.99E-03 | 0.033168 | 0.329645 | H200006784 | REPS2 | RALBP1 associated Eps domain containing 2 | 9185 |
| 16805 | -4.1596 | 0.77815 | 8.63E-05 | 0.005263 | 0.329667 | H200016769 | TSPAN32 | tetraspanin 32 | 10077 |
| 19221 | -4.0739 | 0.486831 | 1.01E-04 | 0.005431 | 0.32999 | H200006597 | MEF2C | myocyte enhancer factor 2C | 4208 |
| 7717 | -3.5088 | 0.700703 | 3.06E-04 | 0.007429 | 0.33029 | H200019146 | MIF4GD | MIF4G domain containing | 57409 |
| 4487 | -1.9414 | 2.789361 | 1.81E-02 | 0.048858 | 0.330388 | H200017746 | TMEM156 | transmembrane protein 156 | 80008 |
| 685 | -1.838 | 1.391437 | 2.43E-02 | 0.05761 | 0.330487 | H200010883 | NA | NA | - |
| 7978 | -2.8386 | 0.67443 | 1.51E-03 | 0.013422 | 0.330576 | H200010079 | NA | NA | - |
| 13688 | -1.8192 | 2.518829 | 2.57E-02 | 0.059561 | 0.330857 | H200020837 | NA | NA | - |
| 11861 | -2.017 | 1.146477 | 1.46E-02 | 0.043394 | 0.330992 | H200020760 | NA | NA | - |
| 15186 | -2.1219 | 1.554992 | 1.08E-02 | 0.036901 | 0.331515 | H200005137 | CDH26 | cadherin 26 | 60437 |
| 20184 | -4.7742 | 0.532288 | 3.28E-05 | 0.004051 | 0.331945 | H200010284 | HDAC1 | histone deacetylase 1 | 3065 |
| 160 | -4.7059 | 0.631158 | 3.60E-05 | 0.004148 | 0.332471 | H200007398 | TMSB4Y | thymosin beta 4, Y-linked | 9087 |
| 13575 | -2.4427 | 1.196827 | 4.35E-03 | 0.022348 | 0.332474 | H200015511 | AQP3 | aquaporin 3 (Gill blood group) | 360 |
| 13774 | -3.7479 | 0.652853 | 1.84E-04 | 0.006172 | 0.332663 | H200003060 | FAM5B | family with sequence similarity 5, member B | 57795 |
| 10723 | -2.8359 | 0.796384 | 1.52E-03 | 0.013487 | 0.332779 | H200010204 | NA | NA | 93964 |
| 14307 | -3.2273 | 0.817205 | 5.79E-04 | 0.009223 | 0.33293 | H200006783 | UCP2 | uncoupling protein 2 (mitochondrial, proton carrier) | 7351 |
| 9106 | -2.7957 | 0.656212 | 1.68E-03 | 0.014216 | 0.33305 | H200020351 | FLJ35390 | uncharacterized LOC255031 | 255031 |
| 8746 | -3.4965 | 0.524342 | 3.14E-04 | 0.007429 | 0.333221 | H200003251 | SYT5 | synaptotagmin V | 6861 |
| 2625 | -2.0528 | 2.180992 | 1.32E-02 | 0.040744 | 0.333252 | H200016298 | FLG | filaggrin | 2312 |
| 17880 | -5.0578 | 0.647444 | 2.09E-05 | 0.003794 | 0.333346 | H200002650 | CRIP1 | cysteine-rich protein 1 (intestinal) | 1396 |
| 17747 | -1.9377 | 1.288218 | 1.83E-02 | 0.049149 | 0.333473 | H200017927 | NMRK1 | nicotinamide riboside kinase 1 | 54981 |
| 21264 | -3.4229 | 0.74428 | 3.70E-04 | 0.007696 | 0.33352 | H200000369 | FGR | Gardner-Rasheed feline sarcoma viral (v-fgr) oncogene homolog | 2268 |
| 11230 | -1.8487 | 1.413186 | 2.36E-02 | 0.056625 | 0.333618 | H200012311 | GLRA1 | glycine receptor, alpha 1 | 2741 |
| 20359 | -1.8343 | 1.46252 | 2.46E-02 | 0.057829 | 0.333695 | H200019784 | ATP6V1E2 | ATPase, H+ transporting, lysosomal 31kDa, V1 subunit E2 | 90423 |
| 15365 | -1.8679 | 3.151741 | 2.23E-02 | 0.054752 | 0.333857 | H200013539 | TAGAP | T-cell activation RhoGTPase activating protein | 117289 |
| 1425 | -2.1834 | 1.23088 | 9.07E-03 | 0.03337 | 0.334037 | H200002867 | ACRBP | acrosin binding protein | 84519 |
| 9146 | -4.1751 | 1.012905 | 8.42E-05 | 0.005263 | 0.334163 | H200000389 | HRG | histidine-rich glycoprotein | 3273 |
| 6499 | -3.0702 | 3.00652 | 8.38E-04 | 0.010389 | 0.334264 | H200004648 | LAIR2 | leukocyte-associated immunoglobulin-like receptor 2 | 3904 |
| 4842 | -2.0204 | 1.892975 | 1.44E-02 | 0.04308 | 0.33465 | H200013037 | HCG26 | HLA complex group 26 (non-protein coding) | 352961 |
| 5277 | -4.7934 | 0.369253 | 3.17E-05 | 0.004036 | 0.334733 | H200012034 | NA | NA | - |
| 14293 | -2.4931 | 0.809392 | 3.78E-03 | 0.020998 | 0.334735 | H200006047 | IRF3 | interferon regulatory factor 3 | 3661 |
| 5146 | -2.2785 | 19.70204 | 6.91E-03 | 0.028579 | 0.334736 | H200005912 | KRT13 | keratin 13 | 3860 |
| 4057 | -4.4259 | 0.468783 | 5.56E-05 | 0.00473 | 0.334781 | H200019099 | EGLN2 | egl nine homolog 2 (C. elegans) | 112398 |
| 20746 | -1.9304 | 0.441089 | 1.87E-02 | 0.049613 | 0.334831 | H200018977 | OSBPL10 | oxysterol binding protein-like 10 | 114884 |
| 5140 | -3.6175 | 0.627775 | 2.39E-04 | 0.006669 | 0.334887 | H200005556 | CLIC3 | chloride intracellular channel 3 | 9022 |
| 20662 | -1.784 | 1.224015 | 2.84E-02 | 0.063128 | 0.334945 | H200014417 | NA | NA | - |
| 1347 | -2.2483 | 0.910881 | 7.55E-03 | 0.030281 | 0.334947 | H200020656 | LOC151657 | uncharacterized LOC151657 | 151657 |
| 9425 | -5.8663 | 0.595317 | 5.68E-06 | 0.003715 | 0.335661 | H200013683 | RPS6KA1 | ribosomal protein S6 kinase, 90kDa, polypeptide 1 | 6195 |
| 6485 | -2.7912 | 0.894442 | 1.70E-03 | 0.014256 | 0.335841 | H200004196 | GRK4 | G protein-coupled receptor kinase 4 | 2868 |
| 8995 | -4.0966 | 0.719511 | 9.69E-05 | 0.005431 | 0.336041 | H200015049 | CCDC69 | coiled-coil domain containing 69 | 26112 |
| 3312 | -2.6512 | 2.687789 | 2.47E-03 | 0.017015 | 0.336527 | H200005320 | PLEKHF1 | pleckstrin homology domain containing, family F (with FYVE domain) member 1 | 79156 |
| 17778 | -1.9595 | 1.016025 | 1.72E-02 | 0.047447 | 0.33654 | H200019441 | NA | NA | - |
| 10480 | -2.363 | 1.175218 | 5.44E-03 | 0.025082 | 0.336699 | H200020185 | NA | NA | - |
| 16415 | -3.3191 | 0.792771 | 4.66E-04 | 0.00858 | 0.336835 | H200020035 | WFDC6 | WAP four-disulfide core domain 6 | 140870 |
| 21432 | -1.7845 | 1.206093 | 2.83E-02 | 0.063066 | 0.337389 | H200008349 | LOC441052 | uncharacterized LOC441052 | 441052 |
| 18973 | -3.2819 | 0.887661 | 5.09E-04 | 0.00873 | 0.337463 | H200015824 | ITIH5 | inter-alpha-trypsin inhibitor heavy chain family, member 5 | 80760 |
| 19735 | -2.2775 | 0.909487 | 6.93E-03 | 0.028627 | 0.337567 | H200009376 | CIDEB | cell death-inducing DFFA-like effector b | 27141 |
| 20629 | -3.0234 | 0.874967 | 9.40E-04 | 0.010845 | 0.337678 | H200012831 | TNFSF14 | tumor necrosis factor (ligand) superfamily, member 14 | 8740 |
| 7549 | -1.9907 | 1.802759 | 1.57E-02 | 0.045068 | 0.337682 | H200011166 | SLC13A2 | solute carrier family 13 (sodium-dependent dicarboxylate transporter), member 2 | 9058 |
| 7735 | -4.8698 | 0.327069 | 2.77E-05 | 0.004028 | 0.337687 | H200019930 | DNAJC4 | DnaJ (Hsp40) homolog, subfamily C, member 4 | 3338 |
| 261 | -2.1735 | 0.976174 | 9.33E-03 | 0.033945 | 0.337832 | H200012308 | NA | NA | - |
| 19111 | -4.8024 | 0.448882 | 3.08E-05 | 0.004028 | 0.337889 | H200001301 | TWF2 | twinfilin, actin-binding protein, homolog 2 (Drosophila) | 11344 |
| 10067 | -3.197 | 0.657563 | 6.20E-04 | 0.009361 | 0.338107 | H200000467 | SLC18A2 | solute carrier family 18 (vesicular monoamine), member 2 | 6571 |
| 21290 | -2.6182 | 1.223256 | 2.70E-03 | 0.017697 | 0.338316 | H200001817 | CD300A | CD300a molecule | 11314 |
| 5029 | -2.3657 | 3.18669 | 5.40E-03 | 0.024946 | 0.338483 | H200000254 | NCF2 | neutrophil cytosolic factor 2 | 4688 |
| 20371 | -2.6693 | 0.886597 | 2.35E-03 | 0.01662 | 0.338618 | H200020218 | NA | NA | - |
| 8407 | -3.2621 | 0.528439 | 5.34E-04 | 0.008903 | 0.33951 | H200008542 | ZNF672 | zinc finger protein 672 | 79894 |
| 10007 | -1.9465 | 1.05267 | 1.78E-02 | 0.04839 | 0.339815 | H200019692 | SYVN1 | synovial apoptosis inhibitor 1, synoviolin | 84447 |
| 5268 | -4.2119 | 0.809795 | 7.99E-05 | 0.005233 | 0.339883 | H200011636 | TNIP1 | TNFAIP3 interacting protein 1 | 10318 |
| 10471 | -2.642 | 0.855814 | 2.53E-03 | 0.017125 | 0.340024 | H200019799 | NA | NA | - |
| 13552 | -1.8061 | 1.262094 | 2.66E-02 | 0.060799 | 0.340039 | H200014377 | NA | NA | - |
| 21391 | -1.8297 | 0.869476 | 2.49E-02 | 0.05837 | 0.340183 | H200006431 | NA | NA | - |
| 20276 | -3.1584 | 0.718218 | 6.77E-04 | 0.009673 | 0.340558 | H200015230 | LRRC56 | leucine rich repeat containing 56 | 115399 |
| 5943 | -4.0707 | 1.195315 | 1.02E-04 | 0.005431 | 0.340713 | H200000313 | ACP5 | acid phosphatase 5, tartrate resistant | 54 |
| 5954 | -2.0502 | 1.04459 | 1.33E-02 | 0.040882 | 0.341155 | H200000723 | TFAP4 | transcription factor AP-4 (activating enhancer binding protein 4) | 7023 |
| 1393 | -2.6202 | 0.80135 | 2.68E-03 | 0.017671 | 0.341193 | H200001347 | DOCK3 | dedicator of cytokinesis 3 | 1795 |
| 3303 | -3.0023 | 0.576795 | 9.92E-04 | 0.011152 | 0.341337 | H200004934 | WDR78 | WD repeat domain 78 | 79819 |
| 17645 | -2.8555 | 1.06181 | 1.44E-03 | 0.013122 | 0.341384 | H200013011 | PIK3C2B | phosphatidylinositol-4-phosphate 3-kinase, catalytic subunit type 2 beta | 5287 |
| 7521 | -3.9575 | 2.03676 | 1.24E-04 | 0.005658 | 0.341414 | H200009978 | RAC2 | ras-related C3 botulinum toxin substrate 2 (rho family, small GTP binding protein Rac2) | 5880 |
| 13253 | -5.2846 | 0.386814 | 1.52E-05 | 0.003715 | 0.34167 | H200000287 | TGFB1 | transforming growth factor, beta 1 | 7040 |
| 6912 | -2.8551 | 0.892825 | 1.45E-03 | 0.013122 | 0.341739 | H200002659 | APBA3 | amyloid beta (A4) precursor protein-binding, family A, member 3 | 9546 |
| 8050 | -1.9609 | 1.034096 | 1.71E-02 | 0.047374 | 0.342319 | H200013499 | NA | NA | - |
| 2606 | -2.7458 | 0.623665 | 1.92E-03 | 0.015139 | 0.342606 | H200015496 | MAPK8IP1 | mitogen-activated protein kinase 8 interacting protein 1 | 9479 |
| 15092 | -2.1891 | 0.974446 | 8.92E-03 | 0.033031 | 0.342757 | H200000601 | KCNJ12 | potassium inwardly-rectifying channel, subfamily J, member 12 | 3768 |
| 21054 | -4.805 | 0.913029 | 3.06E-05 | 0.004028 | 0.34326 | H200012172 | NA | NA | - |
| 739 | -2.5589 | 0.728068 | 3.17E-03 | 0.019182 | 0.343347 | H200013519 | LINC00208 | long intergenic non-protein coding RNA 208 | 83655 |
| 13194 | -2.4758 | 0.972661 | 3.97E-03 | 0.02157 | 0.343735 | H200019008 | RSG1 | REM2 and RAB-like small GTPase 1 | 79363 |
| 622 | -3.4812 | 0.544284 | 3.27E-04 | 0.007449 | 0.34379 | H200007849 | NA | NA | - |
| 12455 | -2.765 | 0.845353 | 1.82E-03 | 0.014755 | 0.344111 | H200005596 | CD38 | CD38 molecule | 952 |
| 1971 | -4.3254 | 0.203281 | 6.61E-05 | 0.004858 | 0.344301 | H200006869 | GYPC | glycophorin C (Gerbich blood group) | 2995 |
| 11463 | -2.2555 | 0.948523 | 7.39E-03 | 0.029872 | 0.344396 | H200001784 | NA | NA | - |
| 6613 | -2.0956 | 0.564701 | 1.17E-02 | 0.03827 | 0.344617 | H200010276 | TASP1 | taspase, threonine aspartase, 1 | 55617 |
| 6225 | -2.1395 | 1.322541 | 1.03E-02 | 0.035835 | 0.344639 | H200013637 | CNFN | cornifelin | 84518 |
| 11063 | -4.0233 | 0.485646 | 1.11E-04 | 0.005439 | 0.345052 | H200004349 | CD7 | CD7 molecule | 924 |
| 21520 | -2.1993 | 0.324852 | 8.67E-03 | 0.032474 | 0.345072 | H200012529 | NA | NA | - |
| 6885 | -2.763 | 0.31286 | 1.83E-03 | 0.014822 | 0.345146 | H200001489 | CDIP1 | cell death-inducing p53 target 1 | 29965 |
| 9939 | -4.9659 | 0.53022 | 2.38E-05 | 0.003926 | 0.345316 | H200016320 | ARHGEF1 | Rho guanine nucleotide exchange factor (GEF) 1 | 9138 |
| 3021 | -2.6156 | 0.794756 | 2.71E-03 | 0.017735 | 0.345332 | H200013317 | NA | NA | - |
| 1600 | -2.8036 | 0.760512 | 1.65E-03 | 0.014047 | 0.345369 | H200010925 | APBB1IP | amyloid beta (A4) precursor protein-binding, family B, member 1 interacting protein | 54518 |
| 2202 | -3.9097 | 0.630548 | 1.35E-04 | 0.005801 | 0.345372 | H200017871 | NA | NA | - |
| 7587 | -2.7111 | 1.941839 | 2.10E-03 | 0.015839 | 0.345788 | H200013042 | NCKAP1L | NCK-associated protein 1-like | 3071 |
| 14085 | -2.1695 | 0.540726 | 9.44E-03 | 0.034187 | 0.345924 | H200017874 | OSBPL10 | oxysterol binding protein-like 10 | 114884 |
| 4912 | -3.8705 | 0.39387 | 1.45E-04 | 0.005857 | 0.346075 | H200016149 | NA | NA | - |
| 16644 | -3.447 | 0.545664 | 3.52E-04 | 0.007621 | 0.346148 | H200009151 | PASK | PAS domain containing serine/threonine kinase | 23178 |
| 20595 | -2.1613 | 1.213989 | 9.67E-03 | 0.034648 | 0.346325 | H200010949 | SPATA17 | spermatogenesis associated 17 | 128153 |
| 13121 | -2.2354 | 1.251214 | 7.84E-03 | 0.030755 | 0.346807 | H200015582 | HMGB4 | high mobility group box 4 | 127540 |
| 18772 | -2.0718 | 1.088814 | 1.25E-02 | 0.039551 | 0.346863 | H200004828 | HYDIN | HYDIN, axonemal central pair apparatus protein | 54768 |
| 5827 | -2.2293 | 1.128085 | 7.97E-03 | 0.031081 | 0.347149 | H200016368 | IQCD | IQ motif containing D | 115811 |
| 17704 | -2.4703 | 0.885583 | 4.03E-03 | 0.021768 | 0.347252 | H200015997 | NA | NA | - |
| 9701 | -4.7167 | 0.415339 | 3.50E-05 | 0.004076 | 0.347375 | H200005228 | LSP1 | lymphocyte-specific protein 1 | 4046 |
| 150 | -4.168 | 0.304379 | 8.51E-05 | 0.005263 | 0.347532 | H200006994 | MAP4K2 | mitogen-activated protein kinase kinase kinase kinase 2 | 5871 |
| 19814 | -2.3175 | 1.32273 | 6.20E-03 | 0.026798 | 0.347564 | H200013170 | SPATS1 | spermatogenesis associated, serine-rich 1 | 221409 |
| 14877 | -2.6033 | 0.976364 | 2.81E-03 | 0.01805 | 0.347586 | H200011924 | SIDT1 | SID1 transmembrane family, member 1 | 54847 |
| 13229 | -2.4202 | 0.68168 | 4.63E-03 | 0.023035 | 0.348483 | H200020570 | SESN3 | sestrin 3 | 143686 |
| 11579 | -1.9963 | 0.651572 | 1.55E-02 | 0.044828 | 0.34851 | H200007436 | MOCS3 | molybdenum cofactor synthesis 3 | 27304 |
| 11458 | -2.5478 | 3.738927 | 3.27E-03 | 0.019477 | 0.34862 | H200001718 | C1QB | complement component 1, q subcomponent, B chain | 713 |
| 18602 | -3.3186 | 1.159294 | 4.67E-04 | 0.00858 | 0.349043 | H200017267 | NA | NA | - |
| 10974 | -2.389 | 0.741628 | 5.05E-03 | 0.024148 | 0.3492 | H200000151 | NA | NA | - |
| 15929 | -2.1914 | 1.295115 | 8.87E-03 | 0.032916 | 0.349713 | H200018728 | KRTAP3-1 | keratin associated protein 3-1 | 83896 |
| 8694 | -3.3705 | 0.539835 | 4.15E-04 | 0.008002 | 0.349803 | H200000639 | ADRB2 | adrenoceptor beta 2, surface | 154 |
| 7592 | -2.6662 | 0.608862 | 2.37E-03 | 0.016702 | 0.350339 | H200013096 | LMX1B | LIM homeobox transcription factor 1, beta | 4010 |
| 4088 | -1.9676 | 1.041822 | 1.68E-02 | 0.046882 | 0.350633 | H200020317 | NA | NA | 200383 |
| 13625 | -1.8824 | 1.319364 | 2.14E-02 | 0.053423 | 0.350634 | H200017815 | EPB41L4B | erythrocyte membrane protein band 4.1 like 4B | 54566 |
| 8825 | -2.0668 | 1.437274 | 1.27E-02 | 0.039784 | 0.350824 | H200007045 | CSRP3 | cysteine and glycine-rich protein 3 (cardiac LIM protein) | 8048 |
| 7755 | -1.9812 | 0.767282 | 1.62E-02 | 0.045818 | 0.35094 | H200021022 | NA | NA | - |
| 3730 | -4.0711 | 0.25983 | 1.02E-04 | 0.005431 | 0.351101 | H200003525 | SLC29A1 | solute carrier family 29 (nucleoside transporters), member 1 | 2030 |
| 7372 | -2.3912 | 0.95447 | 5.02E-03 | 0.02407 | 0.351567 | H200002788 | NA | NA | - |
| 785 | -2.2233 | 2.262724 | 8.11E-03 | 0.031291 | 0.351601 | H200015775 | TNF | tumor necrosis factor | 7124 |
| 9122 | -3.4836 | 0.361586 | 3.25E-04 | 0.007449 | 0.35214 | H200021111 | RAB37 | RAB37, member RAS oncogene family | 326624 |
| 17724 | -2.0376 | 0.967291 | 1.38E-02 | 0.041854 | 0.352425 | H200016805 | NA | NA | 54792 |
| 7431 | -2.9727 | 0.844569 | 1.07E-03 | 0.011452 | 0.352638 | H200005490 | C9orf9 | chromosome 9 open reading frame 9 | 11092 |
| 1948 | -2.7053 | 0.771532 | 2.14E-03 | 0.015941 | 0.352921 | H200005735 | SLC38A6 | solute carrier family 38, member 6 | 145389 |
| 9041 | -2.0996 | 1.29218 | 1.15E-02 | 0.038116 | 0.35296 | H200017305 | CLDN7 | claudin 7 | 1366 |
| 19833 | -3.5828 | 0.600626 | 2.57E-04 | 0.00686 | 0.353 | H200013960 | NA | NA | 388554 |
| 17120 | -2.1252 | 1.228862 | 1.07E-02 | 0.036751 | 0.353314 | H200010190 | PIEZO1 | piezo-type mechanosensitive ion channel component 1 | 9780 |
| 15686 | -2.0869 | 0.820975 | 1.20E-02 | 0.038746 | 0.353874 | H200007002 | GCHFR | GTP cyclohydrolase I feedback regulator | 2644 |
| 6228 | -4.4533 | 0.373318 | 5.29E-05 | 0.004712 | 0.353929 | H200013667 | CYB561D2 | cytochrome b-561 domain containing 2 | 11068 |
| 9934 | -1.8745 | 1.533852 | 2.19E-02 | 0.054174 | 0.354157 | H200016254 | ALPL | alkaline phosphatase, liver/bone/kidney | 249 |
| 1238 | -2.3954 | 0.948341 | 4.96E-03 | 0.023935 | 0.354539 | H200015366 | PPP1R17 | protein phosphatase 1, regulatory subunit 17 | 10842 |
| 15995 | -5.1191 | 0.407895 | 1.91E-05 | 0.003715 | 0.354624 | H200000227 | IMPDH1 | IMP (inosine 5'-monophosphate) dehydrogenase 1 | 3614 |
| 2526 | -2.5415 | 0.576212 | 3.32E-03 | 0.019619 | 0.354942 | H200011696 | DPM3 | dolichyl-phosphate mannosyltransferase polypeptide 3 | 54344 |
| 3340 | -3.6326 | 0.36281 | 2.33E-04 | 0.006651 | 0.354954 | H200006792 | AMFR | autocrine motility factor receptor, E3 ubiquitin protein ligase | 267 |
| 14601 | -6.6065 | 0.903533 | 2.09E-06 | 0.003715 | 0.355196 | H200020819 | NA | NA | - |
| 12546 | -2.1611 | 1.12857 | 9.68E-03 | 0.034648 | 0.355228 | H200010090 | ARPP19 | cAMP-regulated phosphoprotein, 19kDa | 10776 |
| 5165 | -3.0817 | 0.634602 | 8.14E-04 | 0.010284 | 0.355291 | H200006714 | RELT | RELT tumor necrosis factor receptor | 84957 |
| 11119 | -2.5596 | 0.444146 | 3.16E-03 | 0.019157 | 0.355449 | H200007009 | ALDH3B1 | aldehyde dehydrogenase 3 family, member B1 | 221 |
| 76 | -3.7224 | 0.642859 | 1.93E-04 | 0.006231 | 0.355605 | H200003550 | CDK2AP2 | cyclin-dependent kinase 2 associated protein 2 | 10263 |
| 917 | -2.1899 | 1.210814 | 8.90E-03 | 0.033006 | 0.355839 | H200000160 | CPA3 | carboxypeptidase A3 (mast cell) | 1359 |
| 10011 | -2.606 | 0.708191 | 2.79E-03 | 0.017996 | 0.356288 | H200019740 | NA | NA | - |
| 5858 | -2.2171 | 1.550764 | 8.26E-03 | 0.031577 | 0.356331 | H200017870 | EIF4EBP3 | eukaryotic translation initiation factor 4E binding protein 3 | 8637 |
| 19052 | -4.3106 | 0.420778 | 6.78E-05 | 0.004858 | 0.356564 | H200020028 | FAM83C | family with sequence similarity 83, member C | 128876 |
| 19505 | -4.2213 | 0.596658 | 7.84E-05 | 0.005168 | 0.356656 | H200019945 | ZFP36 | ZFP36 ring finger protein | 7538 |
| 20714 | -2.6289 | 0.8768 | 2.62E-03 | 0.017438 | 0.356686 | H200017409 | CABP2 | calcium binding protein 2 | 51475 |
| 6892 | -1.8172 | 1.050466 | 2.58E-02 | 0.059713 | 0.357009 | H200001851 | RMDN2 | regulator of microtubule dynamics 2 | 151393 |
| 3112 | -1.8367 | 1.394641 | 2.44E-02 | 0.057671 | 0.357074 | H200017527 | DNAJC15 | DnaJ (Hsp40) homolog, subfamily C, member 15 | 29103 |
| 14557 | -2.2748 | 0.989899 | 6.99E-03 | 0.02876 | 0.357256 | H200018587 | NA | NA | - |
| 16988 | -2.8596 | 0.622717 | 1.43E-03 | 0.013052 | 0.357449 | H200003778 | MTMR14 | myotubularin related protein 14 | 64419 |
| 396 | -1.811 | 1.171237 | 2.63E-02 | 0.06028 | 0.357562 | H200018750 | DKFZp547J222 | uncharacterized LOC84237 | 84237 |
| 1651 | -2.8386 | 0.579551 | 1.51E-03 | 0.013422 | 0.357563 | H200013531 | NA | NA | - |
| 5806 | -2.7252 | 0.613105 | 2.03E-03 | 0.0156 | 0.357675 | H200015258 | DBH-AS1 | DBH antisense RNA 1 | 138948 |
| 9346 | -2.1632 | 1.422268 | 9.62E-03 | 0.0346 | 0.35777 | H200009889 | B3GNT7 | UDP-GlcNAc:betaGal beta-1,3-N-acetylglucosaminyltransferase 7 | 93010 |
| 692 | -2.5381 | 0.815567 | 3.35E-03 | 0.019638 | 0.357843 | H200011245 | GPT | glutamic-pyruvate transaminase (alanine aminotransferase) | 2875 |
| 9508 | -5.209 | 0.697998 | 1.67E-05 | 0.003715 | 0.358026 | H200017513 | MAN2B1 | mannosidase, alpha, class 2B, member 1 | 4125 |
| 18957 | -2.0743 | 0.967083 | 1.24E-02 | 0.03943 | 0.358582 | H200015040 | NA | NA | 196515 |
| 3734 | -3.4606 | 0.550446 | 3.40E-04 | 0.007537 | 0.358597 | H200003573 | NA | NA | - |
| 1256 | -1.9106 | 0.961625 | 1.98E-02 | 0.051224 | 0.358611 | H200016150 | NA | NA | - |
| 3717 | -3.3387 | 0.629487 | 4.46E-04 | 0.008358 | 0.358845 | H200002807 | MOB2 | MOB kinase activator 2 | 81532 |
| 9443 | -2.9117 | 0.501147 | 1.24E-03 | 0.01225 | 0.359503 | H200014467 | C9orf66 | chromosome 9 open reading frame 66 | 157983 |
| 12193 | -2.449 | 0.944498 | 4.28E-03 | 0.02224 | 0.359602 | H200014929 | ARMC4 | armadillo repeat containing 4 | 55130 |
| 10064 | -2.8875 | 1.478948 | 1.32E-03 | 0.012529 | 0.359865 | H200000425 | ADORA2A | adenosine A2a receptor | 135 |
| 20253 | -3.287 | 0.6176 | 5.03E-04 | 0.008682 | 0.360004 | H200014066 | DBP | D site of albumin promoter (albumin D-box) binding protein | 1628 |
| 17987 | -2.8696 | 4.005024 | 1.39E-03 | 0.012916 | 0.360038 | H200007904 | PTPRC | protein tyrosine phosphatase, receptor type, C | 5788 |
| 14071 | -2.4758 | 0.85933 | 3.97E-03 | 0.02157 | 0.360218 | H200017138 | NA | NA | - |
| 12199 | -3.6772 | 0.445097 | 2.11E-04 | 0.006401 | 0.360385 | H200015001 | PRX | periaxin | 57716 |
| 6009 | -3.0299 | 1.074706 | 9.25E-04 | 0.01078 | 0.360407 | H200003377 | NA | NA | - |
| 18877 | -1.9116 | 2.079547 | 1.97E-02 | 0.051161 | 0.360818 | H200010528 | MYCL1 | v-myc myelocytomatosis viral oncogene homolog 1, lung carcinoma derived (avian) | 4610 |
| 6046 | -3.0186 | 1.302715 | 9.52E-04 | 0.010914 | 0.36082 | H200005235 | ST14 | suppression of tumorigenicity 14 (colon carcinoma) | 6768 |
| 19227 | -3.1457 | 5.50612 | 6.99E-04 | 0.009835 | 0.360822 | H200006953 | AOAH | acyloxyacyl hydrolase (neutrophil) | 313 |
| 14595 | -2.9083 | 0.758923 | 1.25E-03 | 0.012277 | 0.360887 | H200020463 | TUBA3C | tubulin, alpha 3c | 7278 |
| 6339 | -3.0616 | 0.425523 | 8.56E-04 | 0.010432 | 0.360959 | H200018981 | HYI | hydroxypyruvate isomerase (putative) | 81888 |
| 9853 | -2.409 | 0.361146 | 4.78E-03 | 0.023403 | 0.361226 | H200012448 | SPAG4 | sperm associated antigen 4 | 6676 |
| 13897 | -2.6691 | 0.498042 | 2.35E-03 | 0.01662 | 0.361435 | H200008802 | PCDHB17 | protocadherin beta 17 pseudogene | 54661 |
| 1524 | -2.5397 | 0.722249 | 3.33E-03 | 0.019619 | 0.361449 | H200007457 | GRAP | GRB2-related adaptor protein | 10750 |
| 21211 | -1.8391 | 0.32975 | 2.42E-02 | 0.057474 | 0.361667 | H200019446 | NA | NA | - |
| 15078 | -2.3426 | 0.817504 | 5.77E-03 | 0.025935 | 0.361894 | H200008489 | RPL5 | ribosomal protein L5 | 6125 |
| 16291 | -2.6868 | 0.509099 | 2.24E-03 | 0.01621 | 0.361966 | H200014287 | LDLRAP1 | low density lipoprotein receptor adaptor protein 1 | 26119 |
| 19995 | -2.2738 | 0.676892 | 7.01E-03 | 0.028833 | 0.362024 | H200000024 | IL2RG | interleukin 2 receptor, gamma | 3561 |
| 17352 | -3.037 | 0.674142 | 9.07E-04 | 0.010685 | 0.362245 | H200021210 | TRDMT1 | tRNA aspartic acid methyltransferase 1 | 1787 |
| 9762 | -2.6044 | 0.792905 | 2.80E-03 | 0.018028 | 0.362518 | H200007942 | MYL5 | myosin, light chain 5, regulatory | 4636 |
| 19771 | -1.8915 | 0.853407 | 2.09E-02 | 0.052674 | 0.362572 | H200011228 | NA | NA | - |
| 15043 | -1.8375 | 1.135775 | 2.43E-02 | 0.057629 | 0.362579 | H200019880 | LINC00317 | long intergenic non-protein coding RNA 317 | 378828 |
| 17410 | -3.3473 | 0.569182 | 4.40E-04 | 0.008301 | 0.362758 | H200001961 | NA | NA | 114782 |
| 13879 | -3.1051 | 1.32212 | 7.70E-04 | 0.010006 | 0.362893 | H200008018 | NUAK2 | NUAK family, SNF1-like kinase, 2 | 81788 |
| 5928 | -3.1171 | 0.737152 | 7.49E-04 | 0.009958 | 0.362941 | H200020982 | NSMCE4A | non-SMC element 4 homolog A (S. cerevisiae) | 54780 |
| 2593 | -2.0384 | 0.326098 | 1.37E-02 | 0.041802 | 0.362974 | H200014778 | SARDH | sarcosine dehydrogenase | 1757 |
| 6940 | -2.3681 | 1.158824 | 5.36E-03 | 0.024864 | 0.363286 | H200004131 | B9D2 | B9 protein domain 2 | 80776 |
| 17207 | -2.0714 | 0.833552 | 1.25E-02 | 0.039555 | 0.363472 | H200014352 | KATNAL1 | katanin p60 subunit A-like 1 | 84056 |
| 1739 | -2.8902 | 1.15335 | 1.31E-03 | 0.012515 | 0.363626 | H200017711 | CASS4 | Cas scaffolding protein family member 4 | 57091 |
| 17622 | -2.6298 | 0.848971 | 2.61E-03 | 0.017425 | 0.363954 | H200012173 | CCM2L | cerebral cavernous malformation 2-like | 140706 |
| 10360 | -4.3991 | 0.480927 | 5.86E-05 | 0.00473 | 0.364001 | H200014485 | JSRP1 | junctional sarcoplasmic reticulum protein 1 | 126306 |
| 13219 | -3.2554 | 0.670406 | 5.42E-04 | 0.008915 | 0.364245 | H200020166 | SLC9A2 | solute carrier family 9, subfamily A (NHE2, cation proton antiporter 2), member 2 | 6549 |
| 17287 | -2.1174 | 1.016643 | 1.10E-02 | 0.037142 | 0.364577 | H200018152 | NA | NA | - |
| 17031 | -3.5212 | 0.643994 | 2.96E-04 | 0.007417 | 0.364703 | H200005992 | TBCC | tubulin folding cofactor C | 6903 |
| 21362 | -2.3131 | 0.453408 | 6.28E-03 | 0.027011 | 0.364773 | H200005237 | NA | NA | - |
| 14536 | -2.1128 | 0.874431 | 1.11E-02 | 0.037397 | 0.364838 | H200017477 | TRPV2 | transient receptor potential cation channel, subfamily V, member 2 | 51393 |
| 1908 | -2.6251 | 0.796434 | 2.64E-03 | 0.017539 | 0.364844 | H200003835 | NEIL1 | nei endonuclease VIII-like 1 (E. coli) | 79661 |
| 17221 | -1.9613 | 2.554671 | 1.71E-02 | 0.04736 | 0.365096 | H200014804 | SMARCA2 | SWI/SNF related, matrix associated, actin dependent regulator of chromatin, subfamily a, member 2 | 6595 |
| 16072 | -3.315 | 0.711525 | 4.72E-04 | 0.008585 | 0.365119 | H200003701 | EAF2 | ELL associated factor 2 | 55840 |
| 2876 | -2.1781 | 4.691326 | 9.21E-03 | 0.033702 | 0.365237 | H200006175 | CCL4 | chemokine (C-C motif) ligand 4 | 6351 |
| 600 | -2.2476 | 0.996361 | 7.57E-03 | 0.030316 | 0.365843 | H200006733 | NA | NA | - |
| 20626 | -2.958 | 0.713888 | 1.10E-03 | 0.011668 | 0.36591 | H200012499 | DAGLA | diacylglycerol lipase, alpha | 747 |
| 1516 | -2.5418 | 1.227665 | 3.32E-03 | 0.019619 | 0.365967 | H200007077 | HPS1 | Hermansky-Pudlak syndrome 1 | 3257 |
| 4633 | -1.9319 | 0.850775 | 1.86E-02 | 0.049545 | 0.366049 | H200003151 | IQSEC3 | IQ motif and Sec7 domain 3 | 440073 |
| 14089 | -1.8452 | 1.374615 | 2.38E-02 | 0.056968 | 0.366066 | H200017922 | NA | NA | 440016 |
| 3084 | -3.0363 | 0.733401 | 9.10E-04 | 0.010685 | 0.366223 | H200016055 | CHRM4 | cholinergic receptor, muscarinic 4 | 1132 |
| 1604 | -1.9737 | 1.035171 | 1.65E-02 | 0.04635 | 0.367017 | H200011257 | RIPK2 | receptor-interacting serine-threonine kinase 2 | 8767 |
| 5820 | -4.1352 | 0.485526 | 9.03E-05 | 0.00538 | 0.367248 | H200015994 | NA | NA | - |
| 7723 | -4.2934 | 0.35797 | 6.98E-05 | 0.004908 | 0.36741 | H200019502 | NUDT22 | nudix (nucleoside diphosphate linked moiety X)-type motif 22 | 84304 |
| 853 | -1.888 | 0.791838 | 2.11E-02 | 0.053027 | 0.367422 | H200018863 | ZBTB37 | zinc finger and BTB domain containing 37 | 84614 |
| 11626 | -2.0613 | 0.480062 | 1.28E-02 | 0.040181 | 0.367433 | H200009698 | NA | NA | - |
| 7364 | -3.7764 | 0.646967 | 1.74E-04 | 0.006053 | 0.367545 | H200002408 | BIN2 | bridging integrator 2 | 51411 |
| 21525 | -2.3057 | 0.458101 | 6.40E-03 | 0.027269 | 0.367762 | H200012867 | CIB2 | calcium and integrin binding family member 2 | 10518 |
| 8852 | -2.1148 | 0.7343 | 1.10E-02 | 0.037262 | 0.367798 | H200008215 | NEK10 | NIMA-related kinase 10 | 152110 |
| 15267 | -2.0797 | 0.818277 | 1.22E-02 | 0.039167 | 0.368447 | H200008955 | NA | NA | - |
| 3215 | -2.0167 | 1.002221 | 1.46E-02 | 0.043402 | 0.368505 | H200000754 | REG1P | regenerating islet-derived 1 pseudogene | 5969 |
| 1880 | -4.7778 | 0.49975 | 3.23E-05 | 0.004051 | 0.36851 | H200002363 | SEPW1 | selenoprotein W, 1 | 6415 |
| 20286 | -2.3826 | 0.93413 | 5.14E-03 | 0.024326 | 0.368561 | H200015652 | PRAM1 | PML-RARA regulated adaptor molecule 1 | 84106 |
| 19479 | -2.1089 | 0.974982 | 1.12E-02 | 0.037576 | 0.368618 | H200018781 | PRSS47 | protease, serine, 47 | 138652 |
| 21378 | -6.5495 | 0.377032 | 2.59E-06 | 0.003715 | 0.368626 | H200005997 | RHOG | ras homolog family member G | 391 |
| 13677 | -4.5471 | 0.462663 | 4.54E-05 | 0.004512 | 0.368741 | H200020427 | TBC1D10A | TBC1 domain family, member 10A | 83874 |
| 318 | -2.4718 | 1.362008 | 4.02E-03 | 0.021726 | 0.368761 | H200014974 | LILRB1 | leukocyte immunoglobulin-like receptor, subfamily B (with TM and ITIM domains), member 1 | 10859 |
| 8093 | -1.7817 | 1.654232 | 2.86E-02 | 0.063323 | 0.368805 | H200015441 | MMP28 | matrix metallopeptidase 28 | 79148 |
| 3191 | -3.8455 | 0.507861 | 1.52E-04 | 0.005916 | 0.368811 | H200021321 | PLCB2 | phospholipase C, beta 2 | 5330 |
| 15946 | -2.0491 | 1.026695 | 1.33E-02 | 0.040956 | 0.368955 | H200019494 | NA | NA | - |
| 922 | -1.7963 | 1.219396 | 2.74E-02 | 0.061772 | 0.369706 | H200000498 | CRYAB | crystallin, alpha B | 1410 |
| 5152 | -3.7268 | 1.722757 | 1.92E-04 | 0.006226 | 0.369721 | H200005984 | VAMP5 | vesicle-associated membrane protein 5 | 10791 |
| 7314 | -3.2382 | 0.570638 | 5.65E-04 | 0.009086 | 0.370516 | H200000104 | ADH7 | alcohol dehydrogenase 7 (class IV), mu or sigma polypeptide | 131 |
| 5769 | -1.8021 | 1.430444 | 2.69E-02 | 0.061281 | 0.370777 | H200013684 | FOXS1 | forkhead box S1 | 2307 |
| 19364 | -5.0956 | 0.363228 | 1.99E-05 | 0.003764 | 0.370889 | H200013431 | FAM78A | family with sequence similarity 78, member A | 286336 |
| 2002 | -2.1016 | 0.299593 | 1.15E-02 | 0.037965 | 0.371216 | H200008371 | MYBL2 | v-myb myeloblastosis viral oncogene homolog (avian)-like 2 | 4605 |
| 2801 | -2.8962 | 1.739788 | 1.30E-03 | 0.012515 | 0.371381 | H200002725 | C1orf54 | chromosome 1 open reading frame 54 | 79630 |
| 15350 | -2.2883 | 0.863175 | 6.72E-03 | 0.028055 | 0.371565 | H200012785 | ODF3 | outer dense fiber of sperm tails 3 | 113746 |
| 8685 | -3.1546 | 1.407624 | 6.84E-04 | 0.00973 | 0.372392 | H200000253 | IL32 | interleukin 32 | 9235 |
| 5955 | -5.0282 | 0.321617 | 2.15E-05 | 0.003794 | 0.372402 | H200000741 | ARHGAP4 | Rho GTPase activating protein 4 | 393 |
| 12190 | -1.8013 | 0.855878 | 2.70E-02 | 0.061389 | 0.372422 | H200014603 | ROPN1 | rhophilin associated tail protein 1 | 54763 |
| 6742 | -2.2231 | 0.836422 | 8.12E-03 | 0.031291 | 0.372701 | H200016362 | NA | NA | - |
| 19903 | -4.0088 | 0.459305 | 1.13E-04 | 0.005439 | 0.372795 | H200017356 | PIK3R5 | phosphoinositide-3-kinase, regulatory subunit 5 | 23533 |
| 3290 | -3.2578 | 0.440292 | 5.39E-04 | 0.008913 | 0.373192 | H200004488 | RER1 | RER1 retention in endoplasmic reticulum 1 homolog (S. cerevisiae) | 11079 |
| 9811 | -2.0078 | 0.982178 | 1.50E-02 | 0.043861 | 0.373445 | H200010240 | NA | NA | - |
| 14634 | -2.0984 | 1.796692 | 1.16E-02 | 0.038161 | 0.373594 | H200000482 | PRKCH | protein kinase C, eta | 5583 |
| 13754 | -3.0436 | 3.718031 | 8.96E-04 | 0.01067 | 0.373696 | H200001968 | MS4A7 | membrane-spanning 4-domains, subfamily A, member 7 | 58475 |
| 4130 | -3.6101 | 0.58143 | 2.43E-04 | 0.006714 | 0.373842 | H200000960 | CNPPD1 | cyclin Pas1/PHO80 domain containing 1 | 27013 |
| 8736 | -2.088 | 0.562299 | 1.19E-02 | 0.038694 | 0.373946 | H200002563 | XAGE2 | X antigen family, member 2 | 9502 |
| 19489 | -2.0155 | 0.708295 | 1.47E-02 | 0.043485 | 0.374294 | H200019185 | ARSD | arylsulfatase D | 414 |
| 20728 | -2.4447 | 0.777547 | 4.33E-03 | 0.022341 | 0.374295 | H200018169 | BCL7C | B-cell CLL/lymphoma 7C | 9274 |
| 3343 | -2.5491 | 1.379552 | 3.25E-03 | 0.019458 | 0.374325 | H200006834 | LGALS9 | lectin, galactoside-binding, soluble, 9 | 3965 |
| 19336 | -2.5581 | 1.189824 | 3.17E-03 | 0.019199 | 0.374456 | H200011959 | NA | NA | - |
| 13098 | -2.5956 | 15.85403 | 2.86E-03 | 0.018254 | 0.37448 | H200014448 | TMEM176B | transmembrane protein 176B | 28959 |
| 17134 | -2.2604 | 0.930656 | 7.29E-03 | 0.029671 | 0.374505 | H200010926 | ADGB | androglobin | 79747 |
| 4903 | -3.2148 | 1.116146 | 5.94E-04 | 0.00931 | 0.374581 | H200015763 | CALHM2 | calcium homeostasis modulator 2 | 51063 |
| 19954 | -4.7591 | 0.373641 | 3.33E-05 | 0.004051 | 0.374624 | H200019962 | UCN3 | urocortin 3 | 114131 |
| 2603 | -2.9541 | 0.497757 | 1.11E-03 | 0.011718 | 0.375034 | H200015182 | NA | NA | 286126 |
| 10163 | -3.0121 | 1.578413 | 9.68E-04 | 0.011003 | 0.375137 | H200005027 | ITGAX | integrin, alpha X (complement component 3 receptor 4 subunit) | 3687 |
| 19453 | -2.7361 | 0.78265 | 1.97E-03 | 0.01537 | 0.375203 | H200017617 | NA | NA | - |
| 17666 | -2.6563 | 0.723868 | 2.43E-03 | 0.016909 | 0.375325 | H200014121 | NA | NA | - |
| 13532 | -2.2474 | 1.016438 | 7.57E-03 | 0.030316 | 0.375409 | H200013285 | COLQ | collagen-like tail subunit (single strand of homotrimer) of asymmetric acetylcholinesterase | 8292 |
| 12941 | -2.8693 | 0.798536 | 1.39E-03 | 0.012916 | 0.375454 | H200006890 | MYD88 | myeloid differentiation primary response 88 | 4615 |
| 12069 | -1.9158 | 0.33296 | 1.95E-02 | 0.0508 | 0.375811 | H200008897 | NA | NA | - |
| 16265 | -4.9621 | 0.663084 | 2.40E-05 | 0.003926 | 0.375937 | H200013123 | MAP1LC3A | microtubule-associated protein 1 light chain 3 alpha | 84557 |
| 12710 | -2.9738 | 0.735708 | 1.06E-03 | 0.011445 | 0.375945 | H200017738 | NA | NA | - |
| 14262 | -3.9113 | 1.050588 | 1.34E-04 | 0.005801 | 0.375969 | H200004533 | BATF | basic leucine zipper transcription factor, ATF-like | 10538 |
| 9069 | -2.1786 | 1.445075 | 9.19E-03 | 0.033669 | 0.375995 | H200018493 | NA | NA | - |
| 3526 | -4.7733 | 0.295202 | 3.30E-05 | 0.004051 | 0.37642 | H200015556 | PPP1R12C | protein phosphatase 1, regulatory subunit 12C | 54776 |
| 12077 | -3.6075 | 0.591831 | 2.44E-04 | 0.00673 | 0.376488 | H200009277 | NA | NA | - |
| 3081 | -1.9496 | 1.286719 | 1.77E-02 | 0.048266 | 0.376525 | H200016025 | GRIK4 | glutamate receptor, ionotropic, kainate 4 | 2900 |
| 14438 | -2.448 | 0.720108 | 4.29E-03 | 0.022273 | 0.376778 | H200012893 | PRR7 | proline rich 7 (synaptic) | 80758 |
| 8144 | -1.9064 | 1.091465 | 2.00E-02 | 0.051558 | 0.377056 | H200017751 | TNFRSF19 | tumor necrosis factor receptor superfamily, member 19 | 55504 |
| 14364 | -3.6952 | 0.477859 | 2.04E-04 | 0.006332 | 0.377283 | H200009449 | NA | NA | - |
| 18887 | -3.231 | 0.549592 | 5.75E-04 | 0.009191 | 0.377322 | H200011240 | FEZ2 | fasciculation and elongation protein zeta 2 (zygin II) | 9637 |
| 4824 | -3.1098 | 0.365419 | 7.62E-04 | 0.00998 | 0.377435 | H200011969 | ZNF655 | zinc finger protein 655 | 79027 |
| 8891 | -2.6919 | 0.718471 | 2.22E-03 | 0.016186 | 0.377489 | H200010109 | NA | NA | - |
| 8748 | -2.2368 | 0.773517 | 7.81E-03 | 0.030699 | 0.377521 | H200003275 | KCTD4 | potassium channel tetramerisation domain containing 4 | 386618 |
| 12221 | -1.8195 | 1.663145 | 2.57E-02 | 0.059515 | 0.377579 | H200016117 | FGF16 | fibroblast growth factor 16 | 8823 |
| 6621 | -3.6946 | 0.552491 | 2.04E-04 | 0.006332 | 0.37766 | H200010656 | BLOC1S1 | biogenesis of lysosomal organelles complex-1, subunit 1 | 2647 |
| 14105 | -3.5827 | 0.43589 | 2.57E-04 | 0.00686 | 0.377767 | H200018682 | NA | NA | - |
| 8347 | -3.0741 | 0.772875 | 8.28E-04 | 0.010354 | 0.378042 | H200005834 | NFKB2 | nuclear factor of kappa light polypeptide gene enhancer in B-cells 2 (p49/p100) | 4791 |
| 16933 | -4.5333 | 0.359221 | 4.62E-05 | 0.004512 | 0.37826 | H200001124 | SUN2 | Sad1 and UNC84 domain containing 2 | 25777 |
| 2089 | -2.3416 | 0.753583 | 5.78E-03 | 0.02598 | 0.378463 | H200012545 | LRTOMT | leucine rich transmembrane and 0-methyltransferase domain containing | 220074 |
| 19021 | -1.8872 | 1.088837 | 2.11E-02 | 0.053091 | 0.378482 | H200018478 | NA | NA | - |
| 962 | -3.1834 | 2.31055 | 6.39E-04 | 0.009407 | 0.378507 | H200002398 | HCLS1 | hematopoietic cell-specific Lyn substrate 1 | 3059 |
| 13232 | -2.3126 | 0.424248 | 6.29E-03 | 0.027011 | 0.378525 | H200020600 | PGPEP1L | pyroglutamyl-peptidase I-like | 145814 |
| 20590 | -5.8819 | 0.300682 | 5.48E-06 | 0.003715 | 0.378535 | H200010593 | USF2 | upstream transcription factor 2, c-fos interacting | 7392 |
| 4172 | -3.3329 | 0.494694 | 4.53E-04 | 0.008413 | 0.378719 | H200002884 | COQ4 | coenzyme Q4 homolog (S. cerevisiae) | 51117 |
| 16760 | -2.0114 | 1.050533 | 1.48E-02 | 0.043679 | 0.379807 | H200014519 | NA | NA | - |
| 16127 | -3.2544 | 1.072611 | 5.44E-04 | 0.008928 | 0.38009 | H200006355 | LILRB5 | leukocyte immunoglobulin-like receptor, subfamily B (with TM and ITIM domains), member 5 | 10990 |
| 18342 | -3.2988 | 0.504452 | 4.90E-04 | 0.008622 | 0.380094 | H200003189 | CA11 | carbonic anhydrase XI | 770 |
| 18730 | -2.2458 | 0.895274 | 7.60E-03 | 0.030406 | 0.380554 | H200002548 | USE1 | unconventional SNARE in the ER 1 homolog (S. cerevisiae) | 55850 |
| 21463 | -2.3884 | 0.799371 | 5.06E-03 | 0.024149 | 0.380626 | H200009851 | NA | NA | - |
| 1511 | -2.6769 | 0.825767 | 2.30E-03 | 0.01646 | 0.380922 | H200006739 | NA | NA | - |
| 7669 | -2.5117 | 0.980589 | 3.60E-03 | 0.020489 | 0.381197 | H200016866 | NA | NA | - |
| 10876 | -2.149 | 1.04065 | 1.00E-02 | 0.035246 | 0.381229 | H200017430 | GDF2 | growth differentiation factor 2 | 2658 |
| 7819 | -5.0339 | 0.357796 | 2.14E-05 | 0.003794 | 0.381231 | H200002497 | ZFAT | zinc finger and AT hook domain containing | 57623 |
| 18434 | -2.9116 | 0.821935 | 1.24E-03 | 0.01225 | 0.381563 | H200008147 | TINAGL1 | tubulointerstitial nephritis antigen-like 1 | 64129 |
| 11687 | -2.2328 | 0.848608 | 7.90E-03 | 0.030913 | 0.381962 | H200012424 | SLC9A3 | solute carrier family 9, subfamily A (NHE3, cation proton antiporter 3), member 3 | 6550 |
| 17731 | -1.9361 | 1.082306 | 1.84E-02 | 0.049255 | 0.382345 | H200017167 | NA | NA | - |
| 19206 | -2.3607 | 0.739767 | 5.48E-03 | 0.025194 | 0.382708 | H200005855 | ANKRD2 | ankyrin repeat domain 2 (stretch responsive muscle) | 26287 |
| 11781 | -3.6897 | 0.530971 | 2.05E-04 | 0.006332 | 0.382819 | H200016960 | HRASLS2 | HRAS-like suppressor 2 | 54979 |
| 2521 | -3.0543 | 0.353666 | 8.71E-04 | 0.010542 | 0.383085 | H200011358 | RNF166 | ring finger protein 166 | 115992 |
| 7007 | -2.819 | 0.573559 | 1.59E-03 | 0.013766 | 0.383646 | H200007213 | NA | NA | - |
| 5164 | -2.9176 | 0.206571 | 1.22E-03 | 0.012195 | 0.383658 | H200006696 | RPA2 | replication protein A2, 32kDa | 6118 |
| 4258 | -3.6681 | 0.256739 | 2.14E-04 | 0.006468 | 0.383668 | H200007040 | NA | NA | - |
| 7631 | -2.1117 | 0.440362 | 1.11E-02 | 0.037429 | 0.383711 | H200014990 | NA | NA | - |
| 7092 | -1.7893 | 6.670089 | 2.79E-02 | 0.062561 | 0.383777 | H200011351 | AIM2 | absent in melanoma 2 | 9447 |
| 15351 | -3.5746 | 0.449171 | 2.61E-04 | 0.00691 | 0.38378 | H200012803 | CCDC102A | coiled-coil domain containing 102A | 92922 |
| 15880 | -3.6248 | 0.764659 | 2.35E-04 | 0.006659 | 0.383918 | H200016146 | TLX3 | T-cell leukemia homeobox 3 | 30012 |
| 5640 | -3.4216 | 0.479533 | 3.70E-04 | 0.007696 | 0.384029 | H200007302 | PKNOX1 | PBX/knotted 1 homeobox 1 | 5316 |
| 18586 | -4.3524 | 0.312386 | 6.33E-05 | 0.00478 | 0.384508 | H200016483 | PRM3 | protamine 3 | 58531 |
| 6631 | -2.6885 | 0.943647 | 2.23E-03 | 0.016186 | 0.384589 | H200011060 | FMNL1 | formin-like 1 | 752 |
| 5300 | -4.0763 | 0.363405 | 1.01E-04 | 0.005431 | 0.385537 | H200013156 | UNC93B1 | unc-93 homolog B1 (C. elegans) | 81622 |
| 11501 | -2.8904 | 0.748934 | 1.31E-03 | 0.012515 | 0.385605 | H200003660 | LOC377711 | HEAT repeat-containing protein 7A-like | 377711 |
| 10850 | -1.8906 | 1.336572 | 2.09E-02 | 0.052771 | 0.385688 | H200016266 | NA | NA | 79972 |
| 12794 | -1.855 | 0.920929 | 2.32E-02 | 0.056001 | 0.385917 | H200000008 | MS4A2 | membrane-spanning 4-domains, subfamily A, member 2 | 2206 |
| 3241 | -1.824 | 1.020205 | 2.53E-02 | 0.058967 | 0.385953 | H200002202 | C8orf34 | chromosome 8 open reading frame 34 | 116328 |
| 15063 | -1.8993 | 1.642389 | 2.04E-02 | 0.052196 | 0.386423 | H200020688 | LOC151171 | uncharacterized LOC151171 | 151171 |
| 21380 | -1.9358 | 0.665147 | 1.84E-02 | 0.04926 | 0.386434 | H200006021 | CYP2E1 | cytochrome P450, family 2, subfamily E, polypeptide 1 | 1571 |
| 2314 | -6.107 | 0.237625 | 3.59E-06 | 0.003715 | 0.386436 | H200001484 | SMAP2 | small ArfGAP2 | 64744 |
| 6179 | -3.9884 | 0.605244 | 1.18E-04 | 0.005523 | 0.386473 | H200011381 | RPS6KA4 | ribosomal protein S6 kinase, 90kDa, polypeptide 4 | 8986 |
| 13315 | -2.0818 | 0.811448 | 1.21E-02 | 0.039016 | 0.386597 | H200003019 | SLC12A5 | solute carrier family 12 (potassium/chloride transporter), member 5 | 57468 |
| 16749 | -1.7842 | 1.581198 | 2.84E-02 | 0.063093 | 0.386936 | H200014109 | SLC46A2 | solute carrier family 46, member 2 | 57864 |
| 1386 | -3.745 | 0.485798 | 1.86E-04 | 0.006183 | 0.387157 | H200000973 | TRAPPC12 | trafficking protein particle complex 12 | 51112 |
| 16545 | -2.4376 | 1.01053 | 4.41E-03 | 0.022443 | 0.387689 | H200004561 | WDR60 | WD repeat domain 60 | 55112 |
| 14406 | -2.1627 | 0.920042 | 9.63E-03 | 0.034624 | 0.387742 | H200011373 | SIRT6 | sirtuin 6 | 51548 |
| 18935 | -2.6927 | 0.543437 | 2.21E-03 | 0.016167 | 0.387908 | H200013894 | PLK3 | polo-like kinase 3 | 1263 |
| 13101 | -2.214 | 0.809015 | 8.33E-03 | 0.031719 | 0.387922 | H200014490 | NA | NA | - |
| 14814 | -2.1495 | 0.704687 | 1.00E-02 | 0.035224 | 0.38802 | H200008890 | SPA17 | sperm autoantigenic protein 17 | 53340 |
| 9941 | -2.1617 | 0.996845 | 9.66E-03 | 0.034632 | 0.388147 | H200016628 | NA | NA | - |
| 8778 | -4.039 | 0.285633 | 1.09E-04 | 0.005439 | 0.388625 | H200004771 | LYL1 | lymphoblastic leukemia derived sequence 1 | 4066 |
| 2350 | -4.0296 | 0.395752 | 1.10E-04 | 0.005439 | 0.388676 | H200003336 | VPS18 | vacuolar protein sorting 18 homolog (S. cerevisiae) | 57617 |
| 14261 | -2.3531 | 0.959809 | 5.59E-03 | 0.025481 | 0.388706 | H200004527 | CELF4 | CUGBP, Elav-like family member 4 | 56853 |
| 3477 | -2.1884 | 1.069206 | 8.94E-03 | 0.03309 | 0.388745 | H200013270 | NA | NA | - |
| 14962 | -2.0915 | 0.855556 | 1.18E-02 | 0.038497 | 0.388775 | H200016062 | GHSR | growth hormone secretagogue receptor | 2693 |
| 21624 | -2.0129 | 1.283632 | 1.48E-02 | 0.043598 | 0.389169 | H200017469 | RHCG | Rh family, C glycoprotein | 51458 |
| 6440 | -3.5073 | 0.381599 | 3.07E-04 | 0.007429 | 0.389194 | H200001946 | SAT2 | spermidine/spermine N1-acetyltransferase family member 2 | 112483 |
| 10756 | -2.8528 | 0.192124 | 1.45E-03 | 0.013131 | 0.389309 | H200011730 | SLC25A1 | solute carrier family 25 (mitochondrial carrier; citrate transporter), member 1 | 6576 |
| 7584 | -3.0885 | 0.710974 | 8.02E-04 | 0.010245 | 0.389418 | H200012716 | KCNQ1DN | KCNQ1 downstream neighbor (non-protein coding) | 55539 |
| 11303 | -2.1418 | 0.621767 | 1.02E-02 | 0.03572 | 0.389671 | H200015749 | EVL | Enah/Vasp-like | 51466 |
| 19492 | -2.3792 | 0.81394 | 5.19E-03 | 0.024471 | 0.389794 | H200019511 | FCHO2 | FCH domain only 2 | 115548 |
| 19688 | -3.5278 | 0.587002 | 2.92E-04 | 0.007376 | 0.389897 | H200007114 | ANKRD55 | ankyrin repeat domain 55 | 79722 |
| 14607 | -2.4705 | 0.522758 | 4.03E-03 | 0.021768 | 0.38995 | H200020891 | NA | NA | - |
| 5342 | -2.2175 | 1.295974 | 8.25E-03 | 0.031574 | 0.389976 | H200015080 | IL21R | interleukin 21 receptor | 50615 |
| 2407 | -3.4599 | 0.298047 | 3.41E-04 | 0.007537 | 0.390543 | H200006014 | ARFIP2 | ADP-ribosylation factor interacting protein 2 | 23647 |
| 5620 | -2.9762 | 0.250221 | 1.06E-03 | 0.011443 | 0.390661 | H200006494 | APEH | N-acylaminoacyl-peptide hydrolase | 327 |
| 19403 | -1.8008 | 1.392694 | 2.70E-02 | 0.061444 | 0.390781 | H200015313 | C6orf123 | chromosome 6 open reading frame 123 | 26238 |
| 20895 | -2.4551 | 0.806898 | 4.21E-03 | 0.022146 | 0.39142 | H200004578 | MSC | musculin | 9242 |
| 1894 | -2.4608 | 0.688361 | 4.14E-03 | 0.022057 | 0.391526 | H200003099 | TMEM86A | transmembrane protein 86A | 144110 |
| 14476 | -3.8404 | 0.22268 | 1.53E-04 | 0.005935 | 0.392264 | H200014769 | C20orf195 | chromosome 20 open reading frame 195 | 79025 |
| 21200 | -3.3722 | 0.545091 | 4.13E-04 | 0.007987 | 0.392346 | H200019036 | ARPC4 | actin related protein 2/3 complex, subunit 4, 20kDa | 10093 |
| 34 | -2.4946 | 0.637363 | 3.77E-03 | 0.020941 | 0.39245 | H200001626 | CISH | cytokine inducible SH2-containing protein | 1154 |
| 14184 | -3.9077 | 0.387348 | 1.35E-04 | 0.005801 | 0.392477 | H200000757 | SURF1 | surfeit 1 | 6834 |
| 4716 | -2.8521 | 0.554753 | 1.46E-03 | 0.013131 | 0.392611 | H200006981 | PPP1R11 | protein phosphatase 1, regulatory (inhibitor) subunit 11 | 6992 |
| 2755 | -1.9574 | 1.161805 | 1.73E-02 | 0.047622 | 0.392684 | H200000469 | MPO | myeloperoxidase | 4353 |
| 10671 | -2.7609 | 0.630706 | 1.84E-03 | 0.014844 | 0.392689 | H200007592 | ZNF101 | zinc finger protein 101 | 94039 |
| 5360 | -4.6083 | 0.414185 | 4.17E-05 | 0.004304 | 0.393027 | H200015864 | C6orf47 | chromosome 6 open reading frame 47 | 57827 |
| 6852 | -1.898 | 1.330398 | 2.05E-02 | 0.052205 | 0.393402 | H2NC000011 | NA | NA | - |
| 8925 | -3.823 | 1.598832 | 1.58E-04 | 0.005936 | 0.393465 | H200011653 | CORO1A | coronin, actin binding protein, 1A | 11151 |
| 6597 | -3.6844 | 0.364604 | 2.07E-04 | 0.006342 | 0.393753 | H200009516 | ISCA2 | iron-sulfur cluster assembly 2 homolog (S. cerevisiae) | 122961 |
| 9555 | -3.1298 | 0.662747 | 7.23E-04 | 0.009887 | 0.393841 | H200019787 | NA | NA | - |
| 5241 | -2.0262 | 0.49489 | 1.42E-02 | 0.042694 | 0.393971 | H200010466 | 6-Sep | septin 6 | 23157 |
| 20978 | -3.1097 | 0.618564 | 7.62E-04 | 0.00998 | 0.394277 | H200008420 | TNFRSF25 | tumor necrosis factor receptor superfamily, member 25 | 8718 |
| 14657 | -3.4885 | 0.444563 | 3.20E-04 | 0.007429 | 0.394363 | H200001616 | TUSC2 | tumor suppressor candidate 2 | 11334 |
| 17400 | -2.6837 | 0.660365 | 2.26E-03 | 0.016277 | 0.394366 | H200001557 | KIAA1045 | KIAA1045 | 23349 |
| 10716 | -2.3498 | 0.649506 | 5.65E-03 | 0.02565 | 0.39454 | H200009830 | NA | NA | - |
| 14885 | -1.824 | 0.820157 | 2.53E-02 | 0.058967 | 0.394543 | H200012304 | TEF | thyrotrophic embryonic factor | 7008 |
| 4953 | -3.4462 | 0.36574 | 3.52E-04 | 0.007621 | 0.394551 | H200018351 | NA | NA | - |
| 12247 | -1.8398 | 1.043716 | 2.42E-02 | 0.057425 | 0.394553 | H200017281 | NA | NA | - |
| 11384 | -2.2727 | 0.866295 | 7.04E-03 | 0.028896 | 0.394704 | H200019555 | LOC84931 | uncharacterized LOC84931 | 84931 |
| 3732 | -2.8895 | 1.144554 | 1.32E-03 | 0.012529 | 0.394758 | H200003549 | CD40 | CD40 molecule, TNF receptor superfamily member 5 | 958 |
| 2512 | -2.7162 | 0.537112 | 2.07E-03 | 0.015727 | 0.395089 | H200010960 | DLX5 | distal-less homeobox 5 | 1749 |
| 2883 | -3.6228 | 0.434453 | 2.36E-04 | 0.006667 | 0.395138 | H200006549 | ZDHHC24 | zinc finger, DHHC-type containing 24 | 254359 |
| 3095 | -2.5822 | 0.894272 | 2.97E-03 | 0.018531 | 0.395168 | H200016761 | MUC17 | mucin 17, cell surface associated | 140453 |
| 13369 | -3.9568 | 0.473813 | 1.24E-04 | 0.005658 | 0.395292 | H200005655 | CYBA | cytochrome b-245, alpha polypeptide | 1535 |
| 7137 | -2.0771 | 0.881924 | 1.23E-02 | 0.039373 | 0.395295 | H200013601 | AGBL2 | ATP/GTP binding protein-like 2 | 79841 |
| 8088 | -2.9314 | 0.606332 | 1.18E-03 | 0.012028 | 0.395393 | H200015091 | TBR1 | T-box, brain, 1 | 10716 |
| 6188 | -2.4887 | 0.687197 | 3.83E-03 | 0.021104 | 0.395822 | H200011767 | C22orf23 | chromosome 22 open reading frame 23 | 84645 |
| 1792 | -3.4754 | 0.647172 | 3.31E-04 | 0.007506 | 0.396029 | H200020045 | NA | NA | - |
| 16601 | -2.0509 | 0.879692 | 1.32E-02 | 0.040846 | 0.3961 | H200007221 | TM4SF20 | transmembrane 4 L six family member 20 | 79853 |
| 3420 | -4.1188 | 0.353018 | 9.35E-05 | 0.0054 | 0.39624 | H200010592 | LZTS1 | leucine zipper, putative tumor suppressor 1 | 11178 |
| 5365 | -3.2969 | 0.348555 | 4.92E-04 | 0.008622 | 0.396279 | H200016214 | RARA | retinoic acid receptor, alpha | 5914 |
| 6290 | -1.8982 | 1.102642 | 2.05E-02 | 0.052205 | 0.396341 | H200016683 | NA | NA | - |
| 19061 | -1.8307 | 0.967828 | 2.48E-02 | 0.058249 | 0.396545 | H200020734 | NA | NA | - |
| 14666 | -2.4915 | 1.287887 | 3.80E-03 | 0.021022 | 0.39682 | H200002002 | RABAC1 | Rab acceptor 1 (prenylated) | 10567 |
| 20834 | -5.647 | 0.263572 | 8.47E-06 | 0.003715 | 0.396911 | H200001580 | PBXIP1 | pre-B-cell leukemia homeobox interacting protein 1 | 57326 |
| 4727 | -3.3073 | 0.741983 | 4.80E-04 | 0.008593 | 0.397027 | H200007403 | FOXE1 | forkhead box E1 (thyroid transcription factor 2) | 2304 |
| 10767 | -2.895 | 0.866294 | 1.30E-03 | 0.012515 | 0.397078 | H200012152 | ANXA6 | annexin A6 | 309 |
| 16838 | -2.7176 | 0.496059 | 2.06E-03 | 0.015694 | 0.397166 | H200018295 | NA | NA | - |
| 7083 | -2.022 | 0.982022 | 1.44E-02 | 0.042966 | 0.397455 | H200010965 | ZBTB32 | zinc finger and BTB domain containing 32 | 27033 |
| 13432 | -2.4834 | 0.512663 | 3.89E-03 | 0.021321 | 0.397824 | H200008677 | SERPINB1 | serpin peptidase inhibitor, clade B (ovalbumin), member 1 | 1992 |
| 8564 | -2.7233 | 0.86857 | 2.04E-03 | 0.015612 | 0.397939 | H200016100 | SSTR1 | somatostatin receptor 1 | 6751 |
| 9805 | -2.846 | 0.530812 | 1.48E-03 | 0.013269 | 0.39808 | H200010168 | KIR3DL2 | killer cell immunoglobulin-like receptor, three domains, long cytoplasmic tail, 2 | 3812 |
| 21091 | -2.5364 | 0.743019 | 3.37E-03 | 0.019674 | 0.398296 | H200013746 | ELF4 | E74-like factor 4 (ets domain transcription factor) | 2000 |
| 338 | -2.4882 | 0.747129 | 3.83E-03 | 0.021117 | 0.398449 | H200016066 | PRLHR | prolactin releasing hormone receptor | 2834 |
| 6202 | -2.3704 | 0.521087 | 5.33E-03 | 0.024783 | 0.398788 | H200012503 | CYSLTR1 | cysteinyl leukotriene receptor 1 | 10800 |
| 3718 | -2.9223 | 0.795739 | 1.21E-03 | 0.012146 | 0.398926 | H200002813 | GFRA2 | GDNF family receptor alpha 2 | 2675 |
| 3753 | -4.145 | 0.376545 | 8.91E-05 | 0.00538 | 0.399172 | H200004659 | SIRT2 | sirtuin 2 | 22933 |
| 18604 | -3.2153 | 0.255847 | 5.94E-04 | 0.00931 | 0.399407 | H200017581 | DALRD3 | DALR anticodon binding domain containing 3 | 55152 |
| 17150 | -2.6906 | 0.315079 | 2.22E-03 | 0.016186 | 0.39959 | H200011686 | ACBD4 | acyl-CoA binding domain containing 4 | 79777 |
| 17180 | -2.1746 | 0.838855 | 9.30E-03 | 0.033887 | 0.399754 | H200012898 | NA | NA | - |
| 12770 | -2.4808 | 1.111319 | 3.92E-03 | 0.021385 | 0.40024 | H200020730 | NA | NA | - |
| 3375 | -2.4725 | 0.495267 | 4.01E-03 | 0.021714 | 0.400297 | H200008354 | TXNIP | thioredoxin interacting protein | 10628 |
| 14560 | -1.9358 | 0.995222 | 1.84E-02 | 0.04926 | 0.40049 | H200018617 | NA | NA | - |
| 8287 | -3.142 | 2.429181 | 7.04E-04 | 0.009862 | 0.400666 | H200002842 | CMIP | c-Maf inducing protein | 80790 |
| 4967 | -2.4453 | 0.73839 | 4.32E-03 | 0.022336 | 0.40072 | H200018803 | SLC5A10 | solute carrier family 5 (sodium/glucose cotransporter), member 10 | 125206 |
| 20259 | -2.0384 | 0.842015 | 1.37E-02 | 0.041802 | 0.400751 | H200014138 | NA | NA | - |
| 9914 | -1.9601 | 1.266433 | 1.72E-02 | 0.047387 | 0.400956 | H200015162 | TNFRSF18 | tumor necrosis factor receptor superfamily, member 18 | 8784 |
| 2446 | -1.9851 | 1.087256 | 1.60E-02 | 0.045453 | 0.401013 | H200007896 | NA | NA | - |
| 11396 | -2.3226 | 0.758891 | 6.11E-03 | 0.02668 | 0.40116 | H200020267 | MAX | MYC associated factor X | 4149 |
| 4867 | -3.4886 | 0.453751 | 3.20E-04 | 0.007429 | 0.401224 | H200014195 | NA | NA | - |
| 19323 | -2.7414 | 0.540525 | 1.94E-03 | 0.015239 | 0.401288 | H200011513 | TMEM191A | transmembrane protein 191A (pseudogene) | 84222 |
| 18572 | -1.891 | 1.238802 | 2.09E-02 | 0.052709 | 0.401364 | H200015723 | MED26 | mediator complex subunit 26 | 9441 |
| 4973 | -3.5127 | 0.197122 | 3.03E-04 | 0.007429 | 0.401446 | H200019159 | SLX1B | SLX1 structure-specific endonuclease subunit homolog B (S. cerevisiae) | 79008 |
| 9973 | -4.8106 | 0.567872 | 3.02E-05 | 0.004028 | 0.401465 | H200018148 | GLIPR2 | GLI pathogenesis-related 2 | 152007 |
| 9138 | -2.9529 | 0.542261 | 1.12E-03 | 0.011719 | 0.401587 | H200000009 | PTPN7 | protein tyrosine phosphatase, non-receptor type 7 | 5778 |
| 7138 | -1.8501 | 0.919127 | 2.35E-02 | 0.056562 | 0.4016 | H200013607 | ZBTB3 | zinc finger and BTB domain containing 3 | 79842 |
| 10344 | -1.8288 | 0.678217 | 2.50E-02 | 0.058407 | 0.40177 | H200013725 | NA | NA | - |
| 8734 | -2.3347 | 0.95949 | 5.90E-03 | 0.026209 | 0.401901 | H200002539 | CLDN2 | claudin 2 | 9075 |
| 2766 | -2.7663 | 0.533516 | 1.81E-03 | 0.014755 | 0.402013 | H200001163 | NA | NA | 145225 |
| 13394 | -2.1399 | 1.186307 | 1.03E-02 | 0.035819 | 0.402184 | H200006801 | LYN | v-yes-1 Yamaguchi sarcoma viral related oncogene homolog | 4067 |
| 19681 | -3.2868 | 0.343511 | 5.03E-04 | 0.008682 | 0.402279 | H200006740 | PIM2 | pim-2 oncogene | 11040 |
| 7856 | -4.5548 | 0.34559 | 4.50E-05 | 0.004512 | 0.402361 | H200004071 | MKL1 | megakaryoblastic leukemia (translocation) 1 | 57591 |
| 2523 | -2.541 | 0.697342 | 3.32E-03 | 0.019619 | 0.402406 | H200011382 | TMEM160 | transmembrane protein 160 | 54958 |
| 18296 | -2.9698 | 0.565629 | 1.07E-03 | 0.011481 | 0.402409 | H200000861 | PLXNB2 | plexin B2 | 23654 |
| 13586 | -2.802 | 0.637968 | 1.66E-03 | 0.014054 | 0.402409 | H200015921 | NA | NA | - |
| 5905 | -1.8867 | 1.081205 | 2.12E-02 | 0.053112 | 0.40248 | H200020144 | CSH1 | chorionic somatomammotropin hormone 1 (placental lactogen) | 1442 |
| 143 | -2.9254 | 0.60025 | 1.20E-03 | 0.012124 | 0.402611 | H200006632 | CRHR1 | corticotropin releasing hormone receptor 1 | 1394 |
| 9435 | -3.0148 | 0.705512 | 9.61E-04 | 0.01096 | 0.40326 | H200014087 | FAM46C | family with sequence similarity 46, member C | 54855 |
| 12482 | -2.141 | 1.18671 | 1.02E-02 | 0.035729 | 0.403317 | H200007050 | ARHGDIB | Rho GDP dissociation inhibitor (GDI) beta | 397 |
| 19340 | -2.526 | 0.655075 | 3.46E-03 | 0.020025 | 0.40349 | H200012291 | DNAJC28 | DnaJ (Hsp40) homolog, subfamily C, member 28 | 54943 |
| 16301 | -1.7866 | 0.942317 | 2.82E-02 | 0.062875 | 0.403552 | H200014691 | NA | NA | - |
| 879 | -1.9757 | 1.40277 | 1.64E-02 | 0.046187 | 0.403788 | H200020027 | NA | NA | - |
| 45 | -2.4456 | 0.66616 | 4.32E-03 | 0.022332 | 0.404125 | H200002048 | LYPD3 | LY6/PLAUR domain containing 3 | 27076 |
| 15445 | -2.9402 | 1.915257 | 1.16E-03 | 0.011848 | 0.404405 | H200017339 | ADAM19 | ADAM metallopeptidase domain 19 | 8728 |
| 8461 | -3.0637 | 1.353484 | 8.52E-04 | 0.010429 | 0.404649 | H200011178 | TTC5 | tetratricopeptide repeat domain 5 | 91875 |
| 12414 | -3.6108 | 0.560108 | 2.42E-04 | 0.006714 | 0.404686 | H200003678 | TMEM216 | transmembrane protein 216 | 51259 |
| 13811 | -3.1582 | 0.379249 | 6.78E-04 | 0.009673 | 0.404785 | H200004646 | GPX7 | glutathione peroxidase 7 | 2882 |
| 12048 | -3.407 | 0.636905 | 3.81E-04 | 0.007752 | 0.40511 | H200007787 | SERTAD3 | SERTA domain containing 3 | 29946 |
| 2053 | -3.3771 | 0.480803 | 4.09E-04 | 0.007963 | 0.405148 | H200010693 | NFRKB | nuclear factor related to kappaB binding protein | 4798 |
| 15841 | -7.3044 | 0.129314 | 8.97E-07 | 0.003715 | 0.405323 | H200014548 | NA | NA | - |
| 14892 | -2.6661 | 1.013502 | 2.37E-03 | 0.016702 | 0.40534 | H200012666 | LIMS2 | LIM and senescent cell antigen-like domains 2 | 55679 |
| 14275 | -2.0334 | 2.848643 | 1.39E-02 | 0.042207 | 0.405834 | H200005263 | CD226 | CD226 molecule | 10666 |
| 13404 | -3.3049 | 0.464635 | 4.82E-04 | 0.008593 | 0.405909 | H200007205 | NA | NA | - |
| 11013 | -2.4245 | 0.582757 | 4.57E-03 | 0.022886 | 0.405923 | H200002045 | RBKS | ribokinase | 64080 |
| 7821 | -3.6607 | 0.381056 | 2.18E-04 | 0.006496 | 0.406031 | H200002521 | DCTN3 | dynactin 3 (p22) | 11258 |
| 15937 | -4.8519 | 0.203917 | 2.84E-05 | 0.004028 | 0.406133 | H200019108 | SSBP4 | single stranded DNA binding protein 4 | 170463 |
| 13965 | -2.6593 | 0.671323 | 2.42E-03 | 0.016876 | 0.406198 | H200012174 | NA | NA | - |
| 19710 | -2.3233 | 1.031125 | 6.09E-03 | 0.02668 | 0.406316 | H200008230 | ZNF490 | zinc finger protein 490 | 57474 |
| 1941 | -3.422 | 0.413326 | 3.70E-04 | 0.007696 | 0.406405 | H200005373 | ERCC1 | excision repair cross-complementing rodent repair deficiency, complementation group 1 (includes overlapping antisense sequence) | 2067 |
| 5496 | -2.705 | 0.532764 | 2.14E-03 | 0.015941 | 0.406502 | H200000462 | MAG | myelin associated glycoprotein | 4099 |
| 12022 | -2.9538 | 0.720523 | 1.12E-03 | 0.011718 | 0.406527 | H200006623 | SEMA4D | sema domain, immunoglobulin domain (Ig), transmembrane domain (TM) and short cytoplasmic domain, (semaphorin) 4D | 10507 |
| 7611 | -2.0609 | 0.667443 | 1.29E-02 | 0.040197 | 0.406585 | H200014182 | PDZD2 | PDZ domain containing 2 | 23037 |
| 9742 | -2.5264 | 0.944686 | 3.46E-03 | 0.020025 | 0.406603 | H200007134 | LTBP4 | latent transforming growth factor beta binding protein 4 | 8425 |
| 4988 | -2.0085 | 0.754056 | 1.49E-02 | 0.043805 | 0.407004 | H200019901 | SMIM14 | small integral membrane protein 14 | 201895 |
| 8017 | -2.9242 | 0.607519 | 1.20E-03 | 0.012132 | 0.407092 | H200011973 | REXO1 | REX1, RNA exonuclease 1 homolog (S. cerevisiae) | 57455 |
| 4856 | -2.8877 | 0.414675 | 1.32E-03 | 0.012529 | 0.407251 | H200013489 | ADAT3 | adenosine deaminase, tRNA-specific 3 | 113179 |
| 20520 | -2.6891 | 0.870254 | 2.23E-03 | 0.016186 | 0.407363 | H200006793 | SPN | sialophorin | 6693 |
| 11860 | -2.116 | 0.557845 | 1.10E-02 | 0.037204 | 0.407416 | H200020742 | NA | NA | - |
| 245 | -2.3725 | 0.991526 | 5.29E-03 | 0.024696 | 0.407497 | H200011548 | FLI1 | Friend leukemia virus integration 1 | 2313 |
| 5891 | -2.3919 | 0.865526 | 5.01E-03 | 0.024039 | 0.407507 | H200019408 | NA | NA | - |
| 2900 | -3.8084 | 0.470255 | 1.63E-04 | 0.005936 | 0.407802 | H200007315 | PDCD1 | programmed cell death 1 | 5133 |
| 2873 | -3.3304 | 0.312887 | 4.55E-04 | 0.00844 | 0.407879 | H200006145 | MARCKS | myristoylated alanine-rich protein kinase C substrate | 4082 |
| 2868 | -2.0925 | 0.767248 | 1.18E-02 | 0.038432 | 0.407984 | H200005795 | HMMR | hyaluronan-mediated motility receptor (RHAMM) | 3161 |
| 9611 | -3.8262 | 0.41818 | 1.57E-04 | 0.005935 | 0.408027 | H200000740 | CD97 | CD97 molecule | 976 |
| 19529 | -2.7551 | 0.856335 | 1.87E-03 | 0.014971 | 0.408087 | H200021085 | TMEM143 | transmembrane protein 143 | 55260 |
| 5835 | -2.6832 | 0.398654 | 2.26E-03 | 0.016277 | 0.40809 | H200016748 | YPEL3 | yippee-like 3 (Drosophila) | 83719 |
| 896 | -2.1091 | 0.704241 | 1.12E-02 | 0.037572 | 0.408091 | H200020793 | NA | NA | - |
| 3166 | -1.9985 | 0.60683 | 1.54E-02 | 0.044701 | 0.408163 | H200020163 | P4HTM | prolyl 4-hydroxylase, transmembrane (endoplasmic reticulum) | 54681 |
| 16822 | -2.4518 | 1.087758 | 4.24E-03 | 0.022198 | 0.408231 | H200017535 | TNFRSF14 | tumor necrosis factor receptor superfamily, member 14 | 8764 |
| 15219 | -2.6603 | 2.335087 | 2.41E-03 | 0.016858 | 0.408429 | H200006675 | LAPTM5 | lysosomal protein transmembrane 5 | 7805 |
| 16498 | -2.0716 | 0.850312 | 1.25E-02 | 0.039553 | 0.408547 | H200002287 | NA | NA | - |
| 9348 | -2.6431 | 0.824547 | 2.52E-03 | 0.017124 | 0.408591 | H200009913 | PALD1 | phosphatase domain containing, paladin 1 | 27143 |
| 12440 | -2.5113 | 0.622206 | 3.60E-03 | 0.020489 | 0.408931 | H200004842 | SPTB | spectrin, beta, erythrocytic | 6710 |
| 17991 | -2.0403 | 0.714045 | 1.36E-02 | 0.041668 | 0.40938 | H200007952 | LOC148709 | actin pseudogene | 148709 |
| 2057 | -3.2563 | 0.442662 | 5.41E-04 | 0.008913 | 0.409445 | H200011025 | DGKQ | diacylglycerol kinase, theta 110kDa | 1609 |
| 5332 | -4.1227 | 0.337009 | 9.27E-05 | 0.0054 | 0.409741 | H200014676 | NA | NA | - |
| 15002 | -1.9333 | 0.893852 | 1.85E-02 | 0.049473 | 0.409858 | H200017962 | CYFIP2 | cytoplasmic FMR1 interacting protein 2 | 26999 |
| 1997 | -4.2279 | 0.464513 | 7.78E-05 | 0.00516 | 0.410012 | H200008033 | ADCY7 | adenylate cyclase 7 | 113 |
| 10422 | -3.9508 | 0.514221 | 1.25E-04 | 0.005665 | 0.410165 | H200017501 | MAGEH1 | melanoma antigen family H, 1 | 28986 |
| 19395 | -2.9138 | 0.642263 | 1.23E-03 | 0.012235 | 0.410411 | H200014933 | UBXN10 | UBX domain protein 10 | 127733 |
| 5705 | -4.3746 | 0.188707 | 6.09E-05 | 0.00476 | 0.411079 | H200010644 | ZNF580 | zinc finger protein 580 | 51157 |
| 12531 | -3.1378 | 0.357669 | 7.09E-04 | 0.009862 | 0.411385 | H200009348 | TPCN2 | two pore segment channel 2 | 219931 |
| 2499 | -3.148 | 0.441918 | 6.95E-04 | 0.009818 | 0.411505 | H200010242 | CTSW | cathepsin W | 1521 |
| 13313 | -5.4786 | 0.279238 | 1.10E-05 | 0.003715 | 0.411588 | H200002995 | ARAP1 | ArfGAP with RhoGAP domain, ankyrin repeat and PH domain 1 | 116985 |
| 8203 | -3.6498 | 0.439365 | 2.23E-04 | 0.006575 | 0.411725 | H200020737 | ABRA | actin-binding Rho activating protein | 137735 |
| 13005 | -2.7601 | 0.653898 | 1.84E-03 | 0.014844 | 0.411766 | H200009930 | COBLL1 | cordon-bleu WH2 repeat protein-like 1 | 22837 |
| 6646 | -1.78 | 0.89518 | 2.87E-02 | 0.063514 | 0.411938 | H200011802 | XAGE1D | X antigen family, member 1D | 9503 |
| 9427 | -1.9329 | 0.683426 | 1.85E-02 | 0.049504 | 0.412024 | H200013707 | NA | NA | - |
| 16910 | -3.4443 | 0.449718 | 3.54E-04 | 0.007621 | 0.41216 | H200000286 | POU2F2 | POU class 2 homeobox 2 | 5452 |
| 826 | -3.2584 | 0.536826 | 5.38E-04 | 0.008913 | 0.41222 | H200017681 | NA | NA | - |
| 2775 | -3.0202 | 0.454723 | 9.48E-04 | 0.010899 | 0.41245 | H200001561 | KALRN | kalirin, RhoGEF kinase | 8997 |
| 3331 | -2.3561 | 0.558605 | 5.55E-03 | 0.025413 | 0.413 | H200006406 | USP4 | ubiquitin specific peptidase 4 (proto-oncogene) | 7375 |
| 16097 | -3.2293 | 0.433172 | 5.76E-04 | 0.0092 | 0.413014 | H200005143 | MAPK12 | mitogen-activated protein kinase 12 | 6300 |
| 17169 | -2.3756 | 0.601867 | 5.24E-03 | 0.024593 | 0.413258 | H200012476 | PRDM15 | PR domain containing 15 | 63977 |
| 21199 | -2.9425 | 0.46287 | 1.15E-03 | 0.011825 | 0.413501 | H200019018 | PXK | PX domain containing serine/threonine kinase | 54899 |
| 4872 | -4.6746 | 0.369922 | 3.77E-05 | 0.004216 | 0.41351 | H200014249 | TMBIM1 | transmembrane BAX inhibitor motif containing 1 | 64114 |
| 7258 | -4.4469 | 0.355414 | 5.36E-05 | 0.00473 | 0.413661 | H200019307 | NA | NA | - |
| 14206 | -2.9962 | 0.486417 | 1.00E-03 | 0.011181 | 0.413904 | H200001873 | CARD8 | caspase recruitment domain family, member 8 | 22900 |
| 15235 | -1.7957 | 1.1652 | 2.74E-02 | 0.061815 | 0.413972 | H200007435 | NA | NA | - |
| 16670 | -2.9255 | 1.576707 | 1.20E-03 | 0.012124 | 0.414003 | H200010315 | ATP5G2 | ATP synthase, H+ transporting, mitochondrial Fo complex, subunit C2 (subunit 9) | 517 |
| 21488 | -1.8951 | 0.717644 | 2.07E-02 | 0.052386 | 0.414135 | H200011009 | ADPRH | ADP-ribosylarginine hydrolase | 141 |
| 10467 | -2.7074 | 0.607774 | 2.12E-03 | 0.015934 | 0.414414 | H200019467 | NA | NA | - |
| 4893 | -4.304 | 0.262175 | 6.85E-05 | 0.004869 | 0.41446 | H200015359 | IKBKB | inhibitor of kappa light polypeptide gene enhancer in B-cells, kinase beta | 3551 |
| 19786 | -2.4199 | 0.206034 | 4.63E-03 | 0.023037 | 0.414465 | H200011982 | DGKD | diacylglycerol kinase, delta 130kDa | 8527 |
| 6874 | -2.161 | 1.942705 | 9.68E-03 | 0.034648 | 0.414504 | H200001067 | EVI2B | ecotropic viral integration site 2B | 2124 |
| 371 | -2.5547 | 0.513595 | 3.20E-03 | 0.019313 | 0.414634 | H200017604 | NA | NA | - |
| 19415 | -3.014 | 0.452322 | 9.63E-04 | 0.010963 | 0.415055 | H200015741 | TBC1D29 | TBC1 domain family, member 29 | 26083 |
| 10677 | -2.3349 | 0.67214 | 5.89E-03 | 0.026209 | 0.415256 | H200007948 | FRMD7 | FERM domain containing 7 | 90167 |
| 13944 | -3.1983 | 0.578457 | 6.18E-04 | 0.009361 | 0.415256 | H200011064 | OGT | O-linked N-acetylglucosamine (GlcNAc) transferase | 8473 |
| 7883 | -1.9983 | 0.768306 | 1.54E-02 | 0.044705 | 0.415299 | H200005537 | NA | NA | - |
| 13095 | -2.5112 | 0.996513 | 3.60E-03 | 0.020489 | 0.415306 | H200014134 | FAM53B | family with sequence similarity 53, member B | 9679 |
| 14055 | -2.2976 | 0.925384 | 6.55E-03 | 0.027654 | 0.415335 | H200016378 | NA | NA | 400809 |
| 8004 | -2.714 | 0.510464 | 2.08E-03 | 0.015763 | 0.415364 | H200011243 | L3MBTL3 | l(3)mbt-like 3 (Drosophila) | 84456 |
| 7265 | -3.0281 | 0.869966 | 9.30E-04 | 0.01078 | 0.415447 | H200019681 | MFSD7 | major facilitator superfamily domain containing 7 | 84179 |
| 14898 | -2.2087 | 0.75527 | 8.45E-03 | 0.032024 | 0.415504 | H200013022 | NAV2-AS4 | NAV2 antisense RNA 4 | 399876 |
| 14419 | -3.1156 | 0.538954 | 7.52E-04 | 0.009958 | 0.415648 | H200012103 | INO80B | INO80 complex subunit B | 83444 |
| 8547 | -2.0182 | 0.749733 | 1.45E-02 | 0.043297 | 0.415723 | H200015334 | ZNF391 | zinc finger protein 391 | 346157 |
| 5096 | -1.9493 | 1.161966 | 1.77E-02 | 0.048279 | 0.415854 | H200003324 | MCPH1 | microcephalin 1 | 79648 |
| 5976 | -2.151 | 0.698485 | 9.95E-03 | 0.035121 | 0.415872 | H200001839 | NA | NA | - |
| 4023 | -2.5327 | 0.963038 | 3.40E-03 | 0.019825 | 0.415972 | H200017271 | NA | NA | - |
| 20368 | -2.4063 | 0.580955 | 4.81E-03 | 0.02351 | 0.416025 | H200020188 | NA | NA | - |
| 20601 | -2.3751 | 1.124179 | 5.25E-03 | 0.024597 | 0.416151 | H200011311 | NPFF | neuropeptide FF-amide peptide precursor | 8620 |
| 17293 | -2.4355 | 0.94318 | 4.44E-03 | 0.022525 | 0.416167 | H200018224 | UNC45A | unc-45 homolog A (C. elegans) | 55898 |
| 6544 | -2.8969 | 0.722957 | 1.29E-03 | 0.012513 | 0.416243 | H200006886 | SDC1 | syndecan 1 | 6382 |
| 15379 | -2.0948 | 1.064506 | 1.17E-02 | 0.038327 | 0.416272 | H200014275 | JMJD4 | jumonji domain containing 4 | 65094 |
| 5865 | -3.2207 | 0.408372 | 5.86E-04 | 0.00929 | 0.416358 | H200018244 | PDIA2 | protein disulfide isomerase family A, member 2 | 64714 |
| 8702 | -3.6003 | 0.526066 | 2.48E-04 | 0.00677 | 0.41645 | H200001019 | VPS51 | vacuolar protein sorting 51 homolog (S. cerevisiae) | 738 |
| 7730 | -2.9486 | 0.76957 | 1.13E-03 | 0.011762 | 0.416526 | H200019864 | VPS28 | vacuolar protein sorting 28 homolog (S. cerevisiae) | 51160 |
| 9088 | -3.0725 | 0.417766 | 8.32E-04 | 0.010376 | 0.416554 | H200019283 | HPR | haptoglobin-related protein | 3250 |
| 19023 | -2.1752 | 0.653367 | 9.28E-03 | 0.033858 | 0.416753 | H200018502 | NA | NA | - |
| 11307 | -1.9106 | 0.953121 | 1.98E-02 | 0.051224 | 0.416922 | H200016081 | HTR5A | 5-hydroxytryptamine (serotonin) receptor 5A, G protein-coupled | 3361 |
| 2669 | -1.8146 | 1.104564 | 2.60E-02 | 0.059992 | 0.416934 | H200018530 | NA | NA | - |
| 10971 | -2.3825 | 0.627898 | 5.14E-03 | 0.024326 | 0.417171 | H200000121 | LTC4S | leukotriene C4 synthase | 4056 |
| 1576 | -2.1566 | 0.423407 | 9.80E-03 | 0.034805 | 0.417193 | H200009785 | NA | NA | - |
| 8689 | -2.1079 | 0.629347 | 1.13E-02 | 0.037636 | 0.417492 | H200000585 | CD8B | CD8b molecule | 926 |
| 13763 | -4.0788 | 0.379193 | 1.00E-04 | 0.005431 | 0.417518 | H200002366 | DENND6B | DENN/MADD domain containing 6B | 414918 |
| 7788 | -3.4951 | 0.370179 | 3.15E-04 | 0.007429 | 0.417738 | H200000983 | PNPLA6 | patatin-like phospholipase domain containing 6 | 10908 |
| 19768 | -2.6139 | 0.48009 | 2.73E-03 | 0.017762 | 0.417929 | H200010914 | DDX25 | DEAD (Asp-Glu-Ala-Asp) box helicase 25 | 29118 |
| 3277 | -2.2994 | 0.738501 | 6.52E-03 | 0.027554 | 0.41793 | H200003770 | RAB40A | RAB40A, member RAS oncogene family | 142684 |
| 4388 | -2.3039 | 1.360643 | 6.43E-03 | 0.027338 | 0.418019 | H200013144 | CST3 | cystatin C | 1471 |
| 4210 | -2.352 | 0.793874 | 5.61E-03 | 0.025526 | 0.418325 | H200004760 | SHBG | sex hormone-binding globulin | 6462 |
| 17798 | -3.2189 | 0.402047 | 5.88E-04 | 0.009296 | 0.418336 | H200020533 | TRIM78P | tripartite motif containing 78, pseudogene | 117852 |
| 12489 | -2.3935 | 0.584712 | 4.98E-03 | 0.024008 | 0.41835 | H200007424 | LINC00588 | long intergenic non-protein coding RNA 588 | 26138 |
| 17 | -3.2206 | 0.41347 | 5.86E-04 | 0.00929 | 0.418408 | H200000860 | NA | NA | - |
| 8770 | -3.4511 | 0.394733 | 3.48E-04 | 0.007612 | 0.418487 | H200004391 | PLA2G16 | phospholipase A2, group XVI | 11145 |
| 3997 | -2.7528 | 0.436339 | 1.88E-03 | 0.015028 | 0.419103 | H200016107 | NA | NA | - |
| 530 | -2.6209 | 0.510835 | 2.67E-03 | 0.017651 | 0.419306 | H200003621 | NA | NA | - |
| 16892 | -2.6759 | 0.564936 | 2.31E-03 | 0.016483 | 0.419335 | H200020931 | NA | NA | - |
| 10339 | -4.0668 | 0.349851 | 1.03E-04 | 0.005431 | 0.419483 | H200013387 | SPATA6 | spermatogenesis associated 6 | 54558 |
| 10012 | -2.5523 | 0.679202 | 3.22E-03 | 0.019402 | 0.419667 | H200019746 | NA | NA | - |
| 11380 | -2.3346 | 0.309902 | 5.90E-03 | 0.026209 | 0.419724 | H200019507 | IFT46 | intraflagellar transport 46 homolog (Chlamydomonas) | 56912 |
| 4763 | -1.8401 | 2.091348 | 2.42E-02 | 0.057424 | 0.41984 | H200009255 | ANO9 | anoctamin 9 | 338440 |
| 9371 | -2.0872 | 0.91597 | 1.19E-02 | 0.038739 | 0.420068 | H200011047 | NA | NA | - |
| 3262 | -4.0757 | 0.362627 | 1.01E-04 | 0.005431 | 0.420107 | H200003016 | STK40 | serine/threonine kinase 40 | 83931 |
| 7168 | -5.7445 | 0.178546 | 7.18E-06 | 0.003715 | 0.42017 | H200014819 | GNAO1 | guanine nucleotide binding protein (G protein), alpha activating activity polypeptide O | 2775 |
| 326 | -2.2825 | 0.556479 | 6.83E-03 | 0.028348 | 0.420231 | H200015354 | NA | NA | - |
| 18916 | -2.7928 | 0.555651 | 1.69E-03 | 0.014246 | 0.420294 | H200012778 | CRHR1-IT1 | CRHR1 intronic transcript 1 (non-protein coding) | 147081 |
| 18428 | -3.1986 | 0.42174 | 6.18E-04 | 0.009361 | 0.420352 | H200007773 | NA | NA | - |
| 3019 | -2.1907 | 0.788446 | 8.88E-03 | 0.032963 | 0.420377 | H200013009 | NA | NA | - |
| 10783 | -2.2014 | 0.735152 | 8.62E-03 | 0.032386 | 0.420592 | H200012912 | LTB4R2 | leukotriene B4 receptor 2 | 56413 |
| 9726 | -4.0802 | 0.432336 | 1.00E-04 | 0.005431 | 0.420785 | H200006374 | GNAI2 | guanine nucleotide binding protein (G protein), alpha inhibiting activity polypeptide 2 | 2771 |
| 10545 | -2.0771 | 0.469611 | 1.23E-02 | 0.039373 | 0.420805 | H200001820 | GADD45G | growth arrest and DNA-damage-inducible, gamma | 10912 |
| 506 | -1.824 | 0.785425 | 2.53E-02 | 0.058967 | 0.420986 | H200002481 | FNDC5 | fibronectin type III domain containing 5 | 252995 |
| 10082 | -2.3336 | 0.680797 | 5.91E-03 | 0.026248 | 0.421233 | H200001209 | CPSF4 | cleavage and polyadenylation specific factor 4, 30kDa | 10898 |
| 20455 | -2.3455 | 0.629428 | 5.71E-03 | 0.025809 | 0.421805 | H200003349 | SLC22A6 | solute carrier family 22 (organic anion transporter), member 6 | 9356 |
| 13228 | -2.909 | 0.402087 | 1.25E-03 | 0.012277 | 0.421839 | H200020552 | NA | NA | - |
| 7627 | -3.632 | 0.300027 | 2.33E-04 | 0.006651 | 0.421888 | H200014942 | LILRP2 | leukocyte immunoglobulin-like receptor pseudogene 2 | 79166 |
| 2194 | -1.9929 | 0.713339 | 1.56E-02 | 0.044948 | 0.42192 | H200017491 | TEX264 | testis expressed 264 | 51368 |
| 8273 | -2.0891 | 1.416072 | 1.19E-02 | 0.038613 | 0.42202 | H200002390 | NA | NA | - |
| 3668 | -1.8105 | 0.756461 | 2.63E-02 | 0.060294 | 0.422102 | H200000509 | SRD5A2 | steroid-5-alpha-reductase, alpha polypeptide 2 (3-oxo-5 alpha-steroid delta 4-dehydrogenase alpha 2) | 6716 |
| 20459 | -2.8462 | 0.546568 | 1.48E-03 | 0.013269 | 0.422107 | H200003397 | TRAPPC1 | trafficking protein particle complex 1 | 58485 |
| 4045 | -2.1372 | 0.847511 | 1.04E-02 | 0.035922 | 0.422134 | H200018387 | NA | NA | - |
| 884 | -3.5237 | 0.450276 | 2.94E-04 | 0.007412 | 0.422465 | H200020365 | NA | NA | - |
| 14155 | -1.9546 | 1.563258 | 1.74E-02 | 0.047858 | 0.422531 | H200020986 | PKP4 | plakophilin 4 | 8502 |
| 3072 | -2.7273 | 1.782474 | 2.02E-03 | 0.015588 | 0.422593 | H200015627 | DENND2D | DENN/MADD domain containing 2D | 79961 |
| 12945 | -3.801 | 0.292995 | 1.66E-04 | 0.005974 | 0.422676 | H200007222 | CALCOCO1 | calcium binding and coiled-coil domain 1 | 57658 |
| 15965 | -3.2998 | 0.345479 | 4.89E-04 | 0.008622 | 0.423333 | H200020296 | C19orf33 | chromosome 19 open reading frame 33 | 64073 |
| 3523 | -2.4219 | 0.693957 | 4.61E-03 | 0.022996 | 0.423735 | H200015526 | CNTN5 | contactin 5 | 53942 |
| 2419 | -3.5253 | 0.404687 | 2.93E-04 | 0.007394 | 0.423853 | H200006442 | FBRS | fibrosin | 64319 |
| 20962 | -4.1085 | 0.385291 | 9.52E-05 | 0.005429 | 0.423958 | H200007660 | IRF7 | interferon regulatory factor 7 | 3665 |
| 16788 | -2.1942 | 0.696437 | 8.80E-03 | 0.032691 | 0.423963 | H200015991 | KRTAP1-3 | keratin associated protein 1-3 | 81850 |
| 18107 | -2.9176 | 0.640914 | 1.22E-03 | 0.012195 | 0.42407 | H200013604 | INSL6 | insulin-like 6 | 11172 |
| 1695 | -2.01 | 0.546146 | 1.49E-02 | 0.043726 | 0.424184 | H200015479 | ZNF418 | zinc finger protein 418 | 147686 |
| 7561 | -2.5675 | 0.604997 | 3.09E-03 | 0.018905 | 0.424221 | H200011878 | LILRA3 | leukocyte immunoglobulin-like receptor, subfamily A (without TM domain), member 3 | 11026 |
| 15179 | -3.8061 | 0.344806 | 1.64E-04 | 0.005937 | 0.424297 | H200004775 | TCIRG1 | T-cell, immune regulator 1, ATPase, H+ transporting, lysosomal V0 subunit A3 | 10312 |
| 19298 | -3.734 | 0.39007 | 1.90E-04 | 0.00621 | 0.424312 | H200010367 | RYR1 | ryanodine receptor 1 (skeletal) | 6261 |
| 6804 | -4.1337 | 0.515683 | 9.04E-05 | 0.00538 | 0.424387 | H200019094 | MAF1 | MAF1 homolog (S. cerevisiae) | 84232 |
| 8656 | -1.884 | 0.931423 | 2.13E-02 | 0.05331 | 0.424439 | H200020328 | NA | NA | - |
| 2988 | -1.8828 | 1.323104 | 2.14E-02 | 0.053377 | 0.425013 | H200011495 | PLAC8 | placenta-specific 8 | 51316 |
| 20408 | -2.2554 | 0.544767 | 7.39E-03 | 0.029872 | 0.425019 | H200000713 | S100A3 | S100 calcium binding protein A3 | 6274 |
| 6624 | -2.7451 | 0.613716 | 1.92E-03 | 0.015142 | 0.425105 | H200010686 | ZNF628 | zinc finger protein 628 | 89887 |
| 17806 | -3.5196 | 0.388606 | 2.98E-04 | 0.00742 | 0.425143 | H200020913 | NA | NA | - |
| 6610 | -2.2892 | 0.735685 | 6.71E-03 | 0.02803 | 0.425202 | H200009950 | NA | NA | - |
| 8579 | -2.0977 | 0.68629 | 1.16E-02 | 0.038192 | 0.425456 | H200016854 | NA | NA | - |
| 16094 | -2.3866 | 0.54598 | 5.08E-03 | 0.024217 | 0.425541 | H200004817 | POLM | polymerase (DNA directed), mu | 27434 |
| 6525 | -1.9615 | 0.640045 | 1.71E-02 | 0.047353 | 0.425772 | H200006096 | DAZAP2 | DAZ associated protein 2 | 9802 |
| 18091 | -3.2603 | 0.513196 | 5.35E-04 | 0.008903 | 0.425772 | H200012844 | NA | NA | - |
| 5521 | -3.8229 | 0.380049 | 1.59E-04 | 0.005936 | 0.426333 | H200001904 | COPE | coatomer protein complex, subunit epsilon | 11316 |
| 5392 | -3.5059 | 0.254386 | 3.08E-04 | 0.007429 | 0.426396 | H200017384 | NA | NA | - |
| 5126 | -2.5223 | 1.118921 | 3.50E-03 | 0.02018 | 0.426426 | H200004820 | MAP3K14 | mitogen-activated protein kinase kinase kinase 14 | 9020 |
| 6057 | -2.5497 | 0.807042 | 3.25E-03 | 0.019456 | 0.426491 | H200005657 | B3GNT3 | UDP-GlcNAc:betaGal beta-1,3-N-acetylglucosaminyltransferase 3 | 10331 |
| 6224 | -2.3015 | 0.846561 | 6.48E-03 | 0.027442 | 0.426551 | H200013619 | ZNF474 | zinc finger protein 474 | 133923 |
| 1146 | -1.7752 | 0.678561 | 2.91E-02 | 0.063986 | 0.42677 | H200011138 | SLITRK3 | SLIT and NTRK-like family, member 3 | 22865 |
| 16015 | -3.28 | 0.506576 | 5.12E-04 | 0.00873 | 0.426839 | H200001035 | GNG5 | guanine nucleotide binding protein (G protein), gamma 5 | 2787 |
| 15513 | -2.3787 | 0.608767 | 5.20E-03 | 0.024471 | 0.426919 | H200020711 | NA | NA | - |
| 7603 | -3.1334 | 0.390171 | 7.17E-04 | 0.009869 | 0.427204 | H200013802 | LHX4 | LIM homeobox 4 | 89884 |
| 1847 | -2.3098 | 0.842834 | 6.33E-03 | 0.027123 | 0.42747 | H200000837 | FXYD6 | FXYD domain containing ion transport regulator 6 | 53826 |
| 2232 | -3.1359 | 1.511205 | 7.12E-04 | 0.009862 | 0.427623 | H200019083 | CD82 | CD82 molecule | 3732 |
| 8677 | -1.8431 | 0.57731 | 2.40E-02 | 0.057157 | 0.427666 | H2NC000004 | NA | NA | - |
| 5306 | -2.8263 | 0.806026 | 1.56E-03 | 0.013642 | 0.427685 | H200013512 | NA | NA | - |
| 8873 | -2.7989 | 0.481259 | 1.67E-03 | 0.014138 | 0.427818 | H200009325 | RAPGEF1 | Rap guanine nucleotide exchange factor (GEF) 1 | 2889 |
| 17001 | -4.004 | 0.256142 | 1.15E-04 | 0.005439 | 0.427874 | H200004496 | UBIAD1 | UbiA prenyltransferase domain containing 1 | 29914 |
| 17086 | -2.8052 | 0.296727 | 1.64E-03 | 0.014047 | 0.427897 | H200008646 | CHMP1A | charged multivesicular body protein 1A | 5119 |
| 13283 | -4.4122 | 0.243081 | 5.71E-05 | 0.00473 | 0.428102 | H200001499 | TINF2 | TERF1 (TRF1)-interacting nuclear factor 2 | 26277 |
| 20977 | -3.7694 | 1.134371 | 1.77E-04 | 0.00608 | 0.42823 | H200008402 | PSMB8 | proteasome (prosome, macropain) subunit, beta type, 8 (large multifunctional peptidase 7) | 5696 |
| 10175 | -2.894 | 0.778019 | 1.30E-03 | 0.012515 | 0.428689 | H200005739 | NEFM | neurofilament, medium polypeptide | 4741 |
| 21241 | -2.969 | 0.499296 | 1.08E-03 | 0.011503 | 0.428832 | H200020942 | NA | NA | - |
| 7068 | -2.4131 | 0.793967 | 4.72E-03 | 0.023284 | 0.429113 | H200010211 | EHD3 | EH-domain containing 3 | 30845 |
| 9516 | -1.8788 | 1.099632 | 2.16E-02 | 0.053784 | 0.42913 | H200017893 | SMAD3 | SMAD family member 3 | 4088 |
| 12883 | -3.2136 | 0.538327 | 5.96E-04 | 0.00931 | 0.429134 | H200004206 | NA | NA | 196266 |
| 21082 | -1.8756 | 0.92767 | 2.18E-02 | 0.054115 | 0.429254 | H200013360 | NA | NA | - |
| 6151 | -3.8101 | 0.322546 | 1.63E-04 | 0.005936 | 0.430067 | H200010193 | NA | NA | - |
| 1214 | -2.141 | 0.37552 | 1.02E-02 | 0.035729 | 0.430339 | H200014226 | ANK1 | ankyrin 1, erythrocytic | 286 |
| 1533 | -4.0479 | 0.180267 | 1.07E-04 | 0.005439 | 0.430433 | H200007855 | TOR4A | torsin family 4, member A | 54863 |
| 4657 | -2.217 | 0.427389 | 8.26E-03 | 0.031577 | 0.430637 | H200004291 | ZBTB4 | zinc finger and BTB domain containing 4 | 57659 |
| 13220 | -2.0543 | 0.850095 | 1.31E-02 | 0.040663 | 0.430679 | H200020172 | PGLYRP3 | peptidoglycan recognition protein 3 | 114771 |
| 9772 | -2.1129 | 0.654101 | 1.11E-02 | 0.037397 | 0.430683 | H200008346 | NA | NA | - |
| 21172 | -1.9215 | 1.203282 | 1.92E-02 | 0.05028 | 0.430911 | H200017564 | NA | NA | - |
| 10569 | -3.2862 | 0.53809 | 5.04E-04 | 0.008682 | 0.430957 | H200002960 | AP3B2 | adaptor-related protein complex 3, beta 2 subunit | 8120 |
| 989 | -1.8342 | 1.225771 | 2.46E-02 | 0.057829 | 0.430985 | H200003580 | SOSTDC1 | sclerostin domain containing 1 | 25928 |
| 7539 | -2.2196 | 0.686676 | 8.19E-03 | 0.031466 | 0.431055 | H200010762 | NA | NA | - |
| 16210 | -1.8341 | 0.863582 | 2.46E-02 | 0.057831 | 0.431187 | H200010469 | TRIM31 | tripartite motif containing 31 | 11074 |
| 19475 | -2.4153 | 0.448074 | 4.69E-03 | 0.023239 | 0.431345 | H200018733 | LOC339524 | uncharacterized LOC339524 | 339524 |
| 26 | -2.077 | 0.609132 | 1.23E-02 | 0.039373 | 0.431773 | H200001246 | EEFSEC | eukaryotic elongation factor, selenocysteine-tRNA-specific | 60678 |
| 1569 | -2.1971 | 0.679855 | 8.73E-03 | 0.032561 | 0.432271 | H200009707 | NA | NA | - |
| 9163 | -4.8254 | 0.21655 | 2.94E-05 | 0.004028 | 0.432311 | H200001167 | ARHGEF18 | Rho/Rac guanine nucleotide exchange factor (GEF) 18 | 23370 |
| 18749 | -2.6385 | 0.34525 | 2.55E-03 | 0.01723 | 0.432559 | H200003664 | TOX2 | TOX high mobility group box family member 2 | 84969 |
| 16124 | -1.7985 | 0.423298 | 2.72E-02 | 0.061643 | 0.432922 | H200006313 | CES1 | carboxylesterase 1 | 1066 |
| 4013 | -2.561 | 0.598356 | 3.15E-03 | 0.019104 | 0.433333 | H200016867 | FOXD4 | forkhead box D4 | 2298 |
| 14670 | -2.457 | 0.530111 | 4.18E-03 | 0.022124 | 0.433528 | H200002050 | B9D1 | B9 protein domain 1 | 27077 |
| 9469 | -2.2264 | 0.636406 | 8.04E-03 | 0.031179 | 0.433603 | H200015631 | MAML3 | mastermind-like 3 (Drosophila) | 55534 |
| 17608 | -1.8838 | 1.042689 | 2.13E-02 | 0.053322 | 0.433809 | H200011437 | NA | NA | - |
| 17260 | -2.6159 | 0.480884 | 2.71E-03 | 0.017735 | 0.433917 | H200016698 | NA | NA | - |
| 11374 | -2.6876 | 0.459914 | 2.24E-03 | 0.016209 | 0.434134 | H200019151 | TBC1D17 | TBC1 domain family, member 17 | 79735 |
| 19552 | -2.272 | 0.543736 | 7.05E-03 | 0.028939 | 0.434226 | H200000654 | NA | NA | - |
| 12601 | -1.8387 | 0.747734 | 2.43E-02 | 0.057511 | 0.434246 | H200012744 | AATK | apoptosis-associated tyrosine kinase | 9625 |
| 6890 | -2.9206 | 0.448881 | 1.21E-03 | 0.012146 | 0.434309 | H200001827 | C17orf62 | chromosome 17 open reading frame 62 | 79415 |
| 7532 | -3.0519 | 0.480335 | 8.75E-04 | 0.010542 | 0.434373 | H200010388 | ATP5D | ATP synthase, H+ transporting, mitochondrial F1 complex, delta subunit | 513 |
| 1253 | -2.392 | 0.567686 | 5.00E-03 | 0.024039 | 0.43439 | H200016120 | NOG | noggin | 9241 |
| 15302 | -3.2086 | 1.187042 | 6.03E-04 | 0.009326 | 0.434461 | H200010505 | NFKBIE | nuclear factor of kappa light polypeptide gene enhancer in B-cells inhibitor, epsilon | 4794 |
| 3837 | -2.9089 | 0.362351 | 1.25E-03 | 0.012277 | 0.434754 | H200008507 | NA | NA | - |
| 19357 | -2.2007 | 0.461393 | 8.64E-03 | 0.03241 | 0.434951 | H200013057 | PARK2 | parkinson protein 2, E3 ubiquitin protein ligase (parkin) | 5071 |
| 5720 | -3.2135 | 0.60895 | 5.96E-04 | 0.00931 | 0.435004 | H200011102 | BTF3 | basic transcription factor 3 | 689 |
| 19875 | -2.5755 | 0.280597 | 3.03E-03 | 0.018652 | 0.435104 | H200016168 | POLR3A | polymerase (RNA) III (DNA directed) polypeptide A, 155kDa | 11128 |
| 1294 | -3.4936 | 0.311748 | 3.17E-04 | 0.007429 | 0.435292 | H200018026 | ONECUT3 | one cut homeobox 3 | 390874 |
| 18588 | -2.6131 | 0.41061 | 2.73E-03 | 0.017762 | 0.435302 | H200016507 | PDPR | pyruvate dehydrogenase phosphatase regulatory subunit | 55066 |
| 18048 | -2.3579 | 0.724059 | 5.52E-03 | 0.025314 | 0.435342 | H200010630 | MBD5 | methyl-CpG binding domain protein 5 | 55777 |
| 15931 | -2.6323 | 0.613516 | 2.59E-03 | 0.017402 | 0.435806 | H200018752 | NA | NA | - |
| 681 | -2.5777 | 0.463733 | 3.01E-03 | 0.018597 | 0.435859 | H200010835 | SH3BP1 | SH3-domain binding protein 1 | 23616 |
| 21356 | -4.9082 | 0.281457 | 2.57E-05 | 0.004028 | 0.435949 | H200004881 | NA | NA | - |
| 6841 | -1.9609 | 0.603119 | 1.71E-02 | 0.047374 | 0.435966 | H200020964 | NA | NA | - |
| 9724 | -2.58 | 0.54284 | 2.99E-03 | 0.018549 | 0.4366 | H200006066 | AIP | aryl hydrocarbon receptor interacting protein | 9049 |
| 15550 | -2.5264 | 1.229616 | 3.46E-03 | 0.020025 | 0.43714 | H200000542 | TRAF1 | TNF receptor-associated factor 1 | 7185 |
| 19626 | -1.8358 | 0.727538 | 2.45E-02 | 0.057738 | 0.437208 | H200004382 | SCNN1B | sodium channel, non-voltage-gated 1, beta subunit | 6338 |
| 18071 | -3.8977 | 0.299824 | 1.37E-04 | 0.005806 | 0.437277 | H200011752 | NA | NA | - |
| 17394 | -4.1616 | 0.307006 | 8.61E-05 | 0.005263 | 0.437388 | H200001201 | ASMTL | acetylserotonin O-methyltransferase-like | 8623 |
| 9743 | -2.9964 | 0.541034 | 1.00E-03 | 0.011181 | 0.437462 | H200007152 | TXLNB | taxilin beta | 167838 |
| 1131 | -3.1289 | 0.691225 | 7.26E-04 | 0.009887 | 0.438104 | H200010396 | TRADD | TNFRSF1A-associated via death domain | 8717 |
| 3156 | -2.0806 | 0.587291 | 1.22E-02 | 0.039085 | 0.438158 | H200019475 | ELMO1 | engulfment and cell motility 1 | 9844 |
| 19491 | -2.5366 | 10.99815 | 3.36E-03 | 0.019674 | 0.438191 | H200019493 | KRT6A | keratin 6A | 3853 |
| 11274 | -2.0011 | 0.447961 | 1.53E-02 | 0.044434 | 0.438573 | H200014543 | FCAR | Fc fragment of IgA, receptor for | 2204 |
| 1179 | -2.2614 | 0.638901 | 7.28E-03 | 0.029625 | 0.438659 | H200012676 | NA | NA | - |
| 15035 | -2.083 | 0.677976 | 1.21E-02 | 0.038971 | 0.438839 | H200019500 | PLCD4 | phospholipase C, delta 4 | 84812 |
| 6805 | -2.1447 | 0.525389 | 1.01E-02 | 0.035498 | 0.438844 | H200019396 | NAPRT1 | nicotinate phosphoribosyltransferase domain containing 1 | 93100 |
| 13603 | -2.7363 | 0.492222 | 1.97E-03 | 0.01537 | 0.438963 | H200016699 | NA | NA | - |
| 20773 | -2.8664 | 0.384695 | 1.40E-03 | 0.012954 | 0.439126 | H200020479 | HDDC3 | HD domain containing 3 | 374659 |
| 11245 | -3.5224 | 0.494055 | 2.96E-04 | 0.007417 | 0.439531 | H200013065 | LAYN | layilin | 143903 |
| 13076 | -4.2831 | 0.218777 | 7.14E-05 | 0.004929 | 0.439604 | H200013332 | KIAA0226 | KIAA0226 | 9711 |
| 8799 | -3.226 | 0.346373 | 5.80E-04 | 0.009236 | 0.439706 | H200005597 | UNK | unkempt homolog (Drosophila) | 85451 |
| 5920 | -1.9481 | 0.387882 | 1.78E-02 | 0.048329 | 0.439871 | H200020602 | RANBP3L | RAN binding protein 3-like | 202151 |
| 15530 | -4.3221 | 0.341854 | 6.63E-05 | 0.004858 | 0.439883 | H200008489 | RPL5 | ribosomal protein L5 | 6125 |
| 5875 | -1.8367 | 0.58461 | 2.44E-02 | 0.057671 | 0.43989 | H200018648 | NWD1 | NACHT and WD repeat domain containing 1 | 284434 |
| 18644 | -2.1299 | 0.755325 | 1.06E-02 | 0.036428 | 0.440271 | H200019547 | AGBL4 | ATP/GTP binding protein-like 4 | 84871 |
| 4784 | -2.3189 | 0.693264 | 6.17E-03 | 0.026748 | 0.440611 | H200010069 | EOMES | eomesodermin | 8320 |
| 21495 | -2.3394 | 6.049973 | 5.81E-03 | 0.02609 | 0.440686 | H200011371 | PARM1 | prostate androgen-regulated mucin-like protein 1 | 25849 |
| 2794 | -3.8555 | 0.328125 | 1.49E-04 | 0.005879 | 0.440726 | H200002351 | DPP7 | dipeptidyl-peptidase 7 | 29952 |
| 10684 | -2.6959 | 0.597673 | 2.19E-03 | 0.016153 | 0.440781 | H200008310 | DUSP13 | dual specificity phosphatase 13 | 51207 |
| 3742 | -3.0234 | 0.368571 | 9.40E-04 | 0.010845 | 0.440859 | H200003953 | WNT6 | wingless-type MMTV integration site family, member 6 | 7475 |
| 12262 | -2.2315 | 0.98032 | 7.92E-03 | 0.030978 | 0.440882 | H200018023 | P2RY12 | purinergic receptor P2Y, G-protein coupled, 12 | 64805 |
| 13344 | -2.1461 | 0.531155 | 1.01E-02 | 0.035412 | 0.44091 | H200004497 | KCNQ3 | potassium voltage-gated channel, KQT-like subfamily, member 3 | 3786 |
| 7033 | -2.3454 | 0.591789 | 5.72E-03 | 0.025809 | 0.440949 | H200008661 | NA | NA | - |
| 15052 | -2.4121 | 0.484998 | 4.73E-03 | 0.02331 | 0.441078 | H200020266 | MYH7B | myosin, heavy chain 7B, cardiac muscle, beta | 57644 |
| 959 | -3.3049 | 0.38465 | 4.82E-04 | 0.008593 | 0.44117 | H200002084 | SLC35C1 | solute carrier family 35, member C1 | 55343 |
| 9036 | -1.9818 | 0.666555 | 1.61E-02 | 0.045759 | 0.441337 | H200016955 | EXD3 | exonuclease 3'-5' domain containing 3 | 54932 |
| 9882 | -2.4374 | 0.47725 | 4.42E-03 | 0.022443 | 0.441526 | H200013642 | SEMA6B | sema domain, transmembrane domain (TM), and cytoplasmic domain, (semaphorin) 6B | 10501 |
| 10691 | -4.6487 | 0.225509 | 3.90E-05 | 0.004216 | 0.441576 | H200008684 | ZNF835 | zinc finger protein 835 | 90485 |
| 19012 | -2.7286 | 0.594453 | 2.01E-03 | 0.01556 | 0.441618 | H200018074 | NA | NA | - |
| 19067 | -1.8227 | 0.836215 | 2.54E-02 | 0.059131 | 0.441636 | H200021096 | TSNARE1 | t-SNARE domain containing 1 | 203062 |
| 38 | -2.2228 | 0.732516 | 8.12E-03 | 0.031291 | 0.44165 | H200001674 | DUSP26 | dual specificity phosphatase 26 (putative) | 78986 |
| 11251 | -2.5867 | 0.594121 | 2.93E-03 | 0.018436 | 0.442027 | H200013421 | NA | NA | - |
| 12731 | -2.0566 | 0.578395 | 1.30E-02 | 0.040545 | 0.442261 | H200018848 | TMEM120A | transmembrane protein 120A | 83862 |
| 9702 | -2.3058 | 0.956295 | 6.40E-03 | 0.027269 | 0.442324 | H200005234 | PKI55 | DKFZp434H1419 | 150967 |
| 7171 | -3.0639 | 0.451846 | 8.51E-04 | 0.010429 | 0.442457 | H200015145 | TXN2 | thioredoxin 2 | 25828 |
| 9689 | -2.2975 | 1.05519 | 6.55E-03 | 0.027655 | 0.442548 | H200004516 | NA | NA | - |
| 8318 | -3.3427 | 0.348862 | 4.43E-04 | 0.008338 | 0.443382 | H200004344 | CCNDBP1 | cyclin D-type binding-protein 1 | 23582 |
| 5551 | -3.1331 | 0.41315 | 7.18E-04 | 0.009869 | 0.443401 | H200003116 | NARFL | nuclear prelamin A recognition factor-like | 64428 |
| 21676 | -2.7475 | 0.694599 | 1.91E-03 | 0.015128 | 0.443407 | H200020081 | CERS5 | ceramide synthase 5 | 91012 |
| 12718 | -1.8072 | 0.719352 | 2.66E-02 | 0.060687 | 0.443477 | H200018118 | NA | NA | - |
| 13471 | -2.8342 | 0.590704 | 1.53E-03 | 0.01351 | 0.443569 | H200010571 | BAK1 | BCL2-antagonist/killer 1 | 578 |
| 12371 | -3.1305 | 0.415013 | 7.22E-04 | 0.009887 | 0.443786 | H200001748 | AMDHD2 | amidohydrolase domain containing 2 | 51005 |
| 2457 | -3.2235 | 0.54585 | 5.83E-04 | 0.009269 | 0.443918 | H200008318 | INPP5K | inositol polyphosphate-5-phosphatase K | 51763 |
| 13904 | -2.9785 | 0.340082 | 1.05E-03 | 0.011443 | 0.44395 | H200009164 | CCDC170 | coiled-coil domain containing 170 | 80129 |
| 15680 | -2.8639 | 1.081604 | 1.41E-03 | 0.012998 | 0.44399 | H200006646 | CD83 | CD83 molecule | 9308 |
| 12425 | -3.3206 | 0.390526 | 4.65E-04 | 0.00858 | 0.444084 | H200004384 | EFNA5 | ephrin-A5 | 1946 |
| 2368 | -2.5751 | 0.539613 | 3.03E-03 | 0.018669 | 0.44411 | H200004120 | NA | NA | - |
| 18615 | -1.8488 | 0.701918 | 2.36E-02 | 0.05661 | 0.444116 | H200018009 | NYX | nyctalopin | 60506 |
| 5639 | -2.2505 | 0.542325 | 7.50E-03 | 0.030119 | 0.444434 | H200007296 | TADA3 | transcriptional adaptor 3 | 10474 |
| 607 | -3.1979 | 0.393473 | 6.19E-04 | 0.009361 | 0.444534 | H200007107 | CTDNEP1 | CTD nuclear envelope phosphatase 1 | 23399 |
| 16282 | -3.0005 | 0.360382 | 9.96E-04 | 0.011169 | 0.44463 | H200013889 | RPGR | retinitis pigmentosa GTPase regulator | 6103 |
| 14945 | -2.0285 | 0.58525 | 1.41E-02 | 0.042496 | 0.444651 | H200015296 | NA | NA | - |
| 9464 | -2.2223 | 0.761167 | 8.13E-03 | 0.031308 | 0.444729 | H200015281 | NA | NA | - |
| 14620 | -2.4773 | 0.47341 | 3.95E-03 | 0.02151 | 0.444799 | H2NC000009 | NA | NA | - |
| 1477 | -2.1623 | 5.270227 | 9.64E-03 | 0.034624 | 0.444864 | H200005195 | SELM | selenoprotein M | 140606 |
| 13818 | -3.5329 | 0.565429 | 2.88E-04 | 0.007363 | 0.444871 | H200005008 | IFI35 | interferon-induced protein 35 | 3430 |
| 14140 | -1.9144 | 0.792273 | 1.95E-02 | 0.05093 | 0.444991 | H200020232 | ZFP91 | ZFP91 zinc finger protein | 80829 |
| 9263 | -2.3043 | 2.659179 | 6.43E-03 | 0.027337 | 0.445038 | H200005775 | KCTD9 | potassium channel tetramerisation domain containing 9 | 54793 |
| 9115 | -1.9383 | 0.608494 | 1.83E-02 | 0.049104 | 0.44512 | H200020749 | MAGI1-IT1 | MAGI1 intronic transcript 1 (non-protein coding) | 151877 |
| 17530 | -2.9121 | 0.487626 | 1.24E-03 | 0.01225 | 0.445172 | H200007661 | NA | NA | - |
| 17231 | -3.9295 | 0.274651 | 1.31E-04 | 0.005728 | 0.445232 | H200015492 | CCDC109B | coiled-coil domain containing 109B | 55013 |
| 18141 | -3.0822 | 0.423708 | 8.14E-04 | 0.010284 | 0.445245 | H200015148 | SOX1 | SRY (sex determining region Y)-box 1 | 6656 |
| 13324 | -1.8965 | 0.591818 | 2.06E-02 | 0.052311 | 0.445297 | H200003405 | OXLD1 | oxidoreductase-like domain containing 1 | 339229 |
| 5825 | -3.4624 | 0.366216 | 3.39E-04 | 0.007534 | 0.44548 | H200016344 | NA | NA | - |
| 17553 | -1.8853 | 0.467085 | 2.12E-02 | 0.05322 | 0.445674 | H200008783 | NA | NA | 284158 |
| 8330 | -3.3167 | 0.37112 | 4.70E-04 | 0.008585 | 0.445742 | H200005056 | HSPBP1 | HSPA (heat shock 70kDa) binding protein, cytoplasmic cochaperone 1 | 23640 |
| 1706 | -2.8398 | 0.385086 | 1.50E-03 | 0.013418 | 0.445877 | H200016173 | ATP6V1G2 | ATPase, H+ transporting, lysosomal 13kDa, V1 subunit G2 | 534 |
| 1001 | -2.5261 | 0.724665 | 3.46E-03 | 0.020025 | 0.446027 | H200004292 | PRRG2 | proline rich Gla (G-carboxyglutamic acid) 2 | 5639 |
| 3765 | -2.4811 | 0.567283 | 3.91E-03 | 0.021385 | 0.446072 | H200005087 | TNR | tenascin R | 7143 |
| 18856 | -3.4286 | 0.30944 | 3.66E-04 | 0.007659 | 0.446205 | H200009388 | NA | NA | - |
| 9112 | -2.0389 | 0.70303 | 1.37E-02 | 0.041783 | 0.446291 | H200020423 | NA | NA | - |
| 18764 | -3.4896 | 0.424275 | 3.20E-04 | 0.007429 | 0.446369 | H200004442 | ZMAT5 | zinc finger, matrin-type 5 | 55954 |
| 6689 | -2.6481 | 0.224685 | 2.48E-03 | 0.017043 | 0.446422 | H200013744 | OXA1L | oxidase (cytochrome c) assembly 1-like | 5018 |
| 5305 | -2.6551 | 0.512471 | 2.44E-03 | 0.016925 | 0.446764 | H200013506 | DLGAP3 | discs, large (Drosophila) homolog-associated protein 3 | 58512 |
| 17879 | -2.5577 | 1.300608 | 3.18E-03 | 0.019203 | 0.446867 | H200002632 | DOCK2 | dedicator of cytokinesis 2 | 1794 |
| 17148 | -3.065 | 0.663841 | 8.49E-04 | 0.010429 | 0.4469 | H200011378 | SLC44A2 | solute carrier family 44, member 2 | 57153 |
| 3233 | -2.0606 | 0.484772 | 1.29E-02 | 0.040197 | 0.447015 | H200001822 | ZDHHC7 | zinc finger, DHHC-type containing 7 | 55625 |
| 7101 | -4.0873 | 0.095429 | 9.87E-05 | 0.005431 | 0.447053 | H200011749 | FTL | ferritin, light polypeptide | 2512 |
| 18193 | -1.991 | 0.683181 | 1.57E-02 | 0.045068 | 0.447067 | H200017476 | NA | NA | - |
| 1635 | -2.3747 | 0.531994 | 5.26E-03 | 0.024598 | 0.447114 | H200012771 | SARM1 | sterile alpha and TIR motif containing 1 | 23098 |
| 15576 | -2.3151 | 0.617092 | 6.24E-03 | 0.026907 | 0.447171 | H200001706 | MAN1C1 | mannosidase, alpha, class 1C, member 1 | 57134 |
| 7070 | -2.1032 | 0.540498 | 1.14E-02 | 0.037903 | 0.447204 | H200010235 | LINC00652 | long intergenic non-protein coding RNA 652 | 29075 |
| 10291 | -2.9707 | 0.485388 | 1.07E-03 | 0.011473 | 0.447264 | H200011107 | PPP1R18 | protein phosphatase 1, regulatory subunit 18 | 170954 |
| 1743 | -3.7933 | 0.351765 | 1.68E-04 | 0.006017 | 0.447304 | H200017759 | CHCHD5 | coiled-coil-helix-coiled-coil-helix domain containing 5 | 84269 |
| 12168 | -2.0139 | 1.285599 | 1.47E-02 | 0.043576 | 0.447322 | H200013487 | TSSK6 | testis-specific serine kinase 6 | 83983 |
| 21258 | -2.0749 | 0.623749 | 1.24E-02 | 0.039427 | 0.447327 | H200000297 | OVGP1 | oviductal glycoprotein 1, 120kDa | 5016 |
| 11841 | -3.3818 | 0.386055 | 4.05E-04 | 0.007933 | 0.447564 | H200019952 | NA | NA | - |
| 241 | -3.4782 | 0.209556 | 3.28E-04 | 0.007457 | 0.447631 | H200011500 | CUTA | cutA divalent cation tolerance homolog (E. coli) | 51596 |
| 19850 | -1.8629 | 1.903032 | 2.26E-02 | 0.05522 | 0.447983 | H200015022 | ITIH5 | inter-alpha-trypsin inhibitor heavy chain family, member 5 | 80760 |
| 17964 | -2.6731 | 1.930764 | 2.33E-03 | 0.016549 | 0.447991 | H200006782 | IRF1 | interferon regulatory factor 1 | 3659 |
| 13040 | -3.4931 | 0.349137 | 3.17E-04 | 0.007429 | 0.448025 | H200011480 | ATP6V0D1 | ATPase, H+ transporting, lysosomal 38kDa, V0 subunit d1 | 9114 |
| 11056 | -5.1634 | 0.184324 | 1.78E-05 | 0.003715 | 0.448357 | H200003975 | SSU72 | SSU72 RNA polymerase II CTD phosphatase homolog (S. cerevisiae) | 29101 |
| 13334 | -1.8423 | 1.754722 | 2.40E-02 | 0.057224 | 0.448473 | H200004093 | ELMO1 | engulfment and cell motility 1 | 9844 |
| 19914 | -3.0662 | 0.41972 | 8.46E-04 | 0.010421 | 0.448639 | H200018062 | RPLP2P1 | ribosomal protein, large P2, pseudogene 1 | 442175 |
| 14047 | -2.0329 | 0.536919 | 1.39E-02 | 0.042248 | 0.449115 | H200015998 | NA | NA | - |
| 6601 | -4.3795 | 0.24823 | 6.02E-05 | 0.004743 | 0.449231 | H200009564 | NA | NA | - |
| 12073 | -1.8253 | 2.581475 | 2.52E-02 | 0.05884 | 0.449397 | H200009229 | CTSH | cathepsin H | 1512 |
| 17582 | -2.884 | 0.509 | 1.34E-03 | 0.012577 | 0.44941 | H200010273 | ARSA | arylsulfatase A | 410 |
| 18333 | -2.3454 | 0.624806 | 5.72E-03 | 0.025809 | 0.449416 | H200002785 | FAM110A | family with sequence similarity 110, member A | 83541 |
| 16580 | -3.4595 | 0.302376 | 3.41E-04 | 0.007537 | 0.449508 | H200006111 | BTG2 | BTG family, member 2 | 7832 |
| 11971 | -4.1595 | 0.429161 | 8.64E-05 | 0.005263 | 0.449614 | H200004313 | IGFN1 | immunoglobulin-like and fibronectin type III domain containing 1 | 91156 |
| 18737 | -2.3617 | 0.668719 | 5.46E-03 | 0.025144 | 0.44994 | H200002928 | ZNF581 | zinc finger protein 581 | 51545 |
| 21615 | -1.9601 | 0.520061 | 1.72E-02 | 0.047387 | 0.450153 | H200017071 | NGB | neuroglobin | 58157 |
| 15558 | -2.4585 | 0.524852 | 4.17E-03 | 0.022089 | 0.450395 | H200000922 | REC8 | REC8 homolog (yeast) | 9985 |
| 3027 | -2.2292 | 0.202057 | 7.98E-03 | 0.031081 | 0.450903 | H200013389 | NA | NA | - |
| 18698 | -1.833 | 0.764103 | 2.47E-02 | 0.05798 | 0.451045 | H200000980 | NA | NA | - |
| 9963 | -2.5918 | 0.554456 | 2.89E-03 | 0.018371 | 0.451071 | H200017460 | NA | NA | - |
| 10851 | -3.5132 | 0.392079 | 3.02E-04 | 0.007429 | 0.451325 | H200016284 | NMUR1 | neuromedin U receptor 1 | 10316 |
| 5743 | -3.1889 | 0.434526 | 6.31E-04 | 0.009397 | 0.451356 | H200012236 | NA | NA | - |
| 5671 | -3.3159 | 0.243683 | 4.71E-04 | 0.008585 | 0.451358 | H200008816 | FAM195B | family with sequence similarity 195, member B | 348262 |
| 19955 | -3.537 | 0.339574 | 2.86E-04 | 0.007326 | 0.451658 | H200019968 | CMTM1 | CKLF-like MARVEL transmembrane domain containing 1 | 113540 |
| 3464 | -2.2712 | 0.868179 | 7.07E-03 | 0.028996 | 0.451803 | H200012540 | HPS6 | Hermansky-Pudlak syndrome 6 | 79803 |
| 14489 | -5.2341 | 0.144818 | 1.62E-05 | 0.003715 | 0.451854 | H200015499 | VPS11 | vacuolar protein sorting 11 homolog (S. cerevisiae) | 55823 |
| 5022 | -2.0302 | 0.61261 | 1.41E-02 | 0.042377 | 0.451992 | H2NC000011 | NA | NA | - |
| 16083 | -4.4115 | 0.238581 | 5.72E-05 | 0.00473 | 0.452106 | H200004407 | EXOC3L4 | exocyst complex component 3-like 4 | 91828 |
| 5038 | -4.906 | 0.221454 | 2.58E-05 | 0.004028 | 0.452217 | H200000640 | ST6GAL1 | ST6 beta-galactosamide alpha-2,6-sialyltranferase 1 | 6480 |
| 16991 | -2.2057 | 0.623611 | 8.52E-03 | 0.032145 | 0.452349 | H200004092 | RECQL4 | RecQ protein-like 4 | 9401 |
| 12429 | -4.8036 | 0.274583 | 3.07E-05 | 0.004028 | 0.45247 | H200004432 | CINP | cyclin-dependent kinase 2 interacting protein | 51550 |
| 6036 | -1.7831 | 0.677665 | 2.85E-02 | 0.063203 | 0.452691 | H200004547 | PRKG2 | protein kinase, cGMP-dependent, type II | 5593 |
| 17387 | -2.6949 | 0.403068 | 2.20E-03 | 0.016161 | 0.452745 | H200000827 | APBB1 | amyloid beta (A4) precursor protein-binding, family B, member 1 (Fe65) | 322 |
| 14685 | -4.2812 | 0.321413 | 7.16E-05 | 0.004929 | 0.452814 | H200002804 | MFSD5 | major facilitator superfamily domain containing 5 | 84975 |
| 5892 | -2.6517 | 0.62917 | 2.46E-03 | 0.017004 | 0.452934 | H200019414 | NA | NA | - |
| 13919 | -2.1733 | 8.521555 | 9.33E-03 | 0.033945 | 0.453527 | H200009918 | NA | NA | - |
| 6344 | -2.1712 | 0.379033 | 9.39E-03 | 0.03408 | 0.453617 | H200019319 | IGSF8 | immunoglobulin superfamily, member 8 | 93185 |
| 521 | -2.3462 | 0.501304 | 5.70E-03 | 0.025809 | 0.453668 | H200003235 | ANAPC2 | anaphase promoting complex subunit 2 | 29882 |
| 6489 | -2.242 | 0.533227 | 7.68E-03 | 0.030569 | 0.453677 | H200004244 | SIDT2 | SID1 transmembrane family, member 2 | 51092 |
| 464 | -2.426 | 1.089138 | 4.55E-03 | 0.022845 | 0.453724 | H200000273 | TRIM21 | tripartite motif containing 21 | 6737 |
| 4485 | -1.8987 | 0.67663 | 2.04E-02 | 0.052205 | 0.4539 | H200017722 | CPA6 | carboxypeptidase A6 | 57094 |
| 15671 | -2.2656 | 1.105896 | 7.19E-03 | 0.029355 | 0.454159 | H200006260 | NA | NA | - |
| 17624 | -2.724 | 0.417573 | 2.03E-03 | 0.015612 | 0.454258 | H200012197 | ZNF646 | zinc finger protein 646 | 9726 |
| 20587 | -4.0438 | 0.166564 | 1.08E-04 | 0.005439 | 0.454454 | H200010551 | ABHD14A | abhydrolase domain containing 14A | 25864 |
| 19028 | -2.0968 | 0.595705 | 1.16E-02 | 0.038226 | 0.454586 | H200018858 | CABYR | calcium binding tyrosine-(Y)-phosphorylation regulated | 26256 |
| 9962 | -2.793 | 0.432816 | 1.69E-03 | 0.014246 | 0.454701 | H200017442 | SFRP5 | secreted frizzled-related protein 5 | 6425 |
| 16030 | -2.2024 | 0.477538 | 8.60E-03 | 0.032332 | 0.454899 | H200001777 | C10orf76 | chromosome 10 open reading frame 76 | 79591 |
| 11257 | -1.9684 | 0.39754 | 1.68E-02 | 0.046826 | 0.455146 | H200013777 | SH2D1A | SH2 domain containing 1A | 4068 |
| 18084 | -2.9553 | 0.461586 | 1.11E-03 | 0.0117 | 0.45531 | H200012482 | DTX1 | deltex homolog 1 (Drosophila) | 1840 |
| 13626 | -2.589 | 0.560118 | 2.91E-03 | 0.018403 | 0.455576 | H200017821 | PCDHGB3 | protocadherin gamma subfamily B, 3 | 56102 |
| 457 | -1.9847 | 0.636001 | 1.60E-02 | 0.045471 | 0.455696 | H200000195 | GHRHR | growth hormone releasing hormone receptor | 2692 |
| 15697 | -3.3839 | 0.32937 | 4.02E-04 | 0.007913 | 0.455944 | H200007708 | DNM2 | dynamin 2 | 1785 |
| 21226 | -2.5203 | 0.719626 | 3.52E-03 | 0.020254 | 0.456378 | H200020200 | NA | NA | - |
| 21472 | -3.4944 | 0.128946 | 3.16E-04 | 0.007429 | 0.45667 | H200010249 | GGA3 | golgi-associated, gamma adaptin ear containing, ARF binding protein 3 | 23163 |
| 7439 | -2.2369 | 3.128613 | 7.80E-03 | 0.030699 | 0.45696 | H200005870 | CCL3 | chemokine (C-C motif) ligand 3 | 6348 |
| 7019 | -4.4251 | 0.325272 | 5.57E-05 | 0.00473 | 0.457249 | H200007925 | RABEP2 | rabaptin, RAB GTPase binding effector protein 2 | 79874 |
| 11582 | -3.5901 | 0.47123 | 2.53E-04 | 0.006809 | 0.457251 | H200007466 | ABCD1 | ATP-binding cassette, sub-family D (ALD), member 1 | 215 |
| 4732 | -2.6699 | 0.491485 | 2.35E-03 | 0.016619 | 0.45739 | H200007741 | MED6 | mediator complex subunit 6 | 10001 |
| 11728 | -2.5608 | 0.653421 | 3.15E-03 | 0.019109 | 0.457506 | H200014330 | NA | NA | - |
| 8603 | -3.2534 | 0.612255 | 5.45E-04 | 0.008938 | 0.457567 | H200017994 | STAB1 | stabilin 1 | 23166 |
| 12128 | -2.4292 | 0.555199 | 4.52E-03 | 0.022749 | 0.457726 | H200011587 | STRN4 | striatin, calmodulin binding protein 4 | 29888 |
| 13792 | -4.2913 | 0.297811 | 7.00E-05 | 0.004908 | 0.45787 | H200003844 | ITPRIP | inositol 1,4,5-trisphosphate receptor interacting protein | 85450 |
| 18240 | -4.4293 | 0.174544 | 5.52E-05 | 0.00473 | 0.458211 | H200019750 | ORAI2 | ORAI calcium release-activated calcium modulator 2 | 80228 |
| 2731 | -1.8887 | 0.691064 | 2.10E-02 | 0.052958 | 0.458218 | H200021262 | CLEC4C | C-type lectin domain family 4, member C | 170482 |
| 18792 | -3.0691 | 0.392879 | 8.40E-04 | 0.010389 | 0.458354 | H200005962 | PLEKHM2 | pleckstrin homology domain containing, family M (with RUN domain) member 2 | 23207 |
| 664 | -1.9198 | 0.671121 | 1.92E-02 | 0.050442 | 0.458654 | H200009773 | NA | NA | - |
| 13123 | -2.34 | 3.270032 | 5.81E-03 | 0.026063 | 0.458813 | H200015606 | IL7R | interleukin 7 receptor | 3575 |
| 17700 | -1.8107 | 0.723829 | 2.63E-02 | 0.06028 | 0.458841 | H200015665 | MAB21L1 | mab-21-like 1 (C. elegans) | 4081 |
| 9197 | -3.3512 | 0.360863 | 4.35E-04 | 0.00825 | 0.458906 | H200002711 | DENND3 | DENN/MADD domain containing 3 | 22898 |
| 14015 | -3.141 | 0.31736 | 7.05E-04 | 0.009862 | 0.459269 | H200014478 | NICN1 | nicolin 1 | 84276 |
| 6247 | -2.7387 | 0.404934 | 1.96E-03 | 0.015304 | 0.459395 | H200014753 | SLC7A4 | solute carrier family 7 (orphan transporter), member 4 | 6545 |
| 10330 | -2.5157 | 0.762314 | 3.56E-03 | 0.020385 | 0.459418 | H200012989 | GPR37L1 | G protein-coupled receptor 37 like 1 | 9283 |
| 20575 | -2.5955 | 0.544457 | 2.86E-03 | 0.018254 | 0.459769 | H200009815 | NA | NA | - |
| 16138 | -4.3603 | 0.260895 | 6.20E-05 | 0.00476 | 0.459791 | H200007049 | ADRBK1 | adrenergic, beta, receptor kinase 1 | 156 |
| 561 | -2.1097 | 0.698605 | 1.12E-02 | 0.03755 | 0.459913 | H200005135 | NA | NA | - |
| 4911 | -1.9244 | 0.62944 | 1.90E-02 | 0.050084 | 0.460109 | H200016143 | CAPS2 | calcyphosine 2 | 84698 |
| 17110 | -3.6763 | 0.340291 | 2.11E-04 | 0.006401 | 0.460148 | H200009786 | NA | NA | - |
| 16773 | -2.3214 | 0.640984 | 6.13E-03 | 0.02668 | 0.460159 | H200015249 | LRRC3DN | LRRC3 downstream neighbor (non-protein coding) | 54083 |
| 5440 | -3.2012 | 0.317606 | 6.14E-04 | 0.009361 | 0.460181 | H200019664 | UBL7 | ubiquitin-like 7 (bone marrow stromal cell-derived) | 84993 |
| 20763 | -2.6478 | 0.506553 | 2.49E-03 | 0.017043 | 0.460289 | H200020069 | CHADL | chondroadherin-like | 150356 |
| 16044 | -3.253 | 0.456165 | 5.46E-04 | 0.008938 | 0.460303 | H200002513 | SNX11 | sorting nexin 11 | 29916 |
| 12602 | -2.0117 | 0.602235 | 1.48E-02 | 0.043665 | 0.460471 | H200012750 | TLE6 | transducin-like enhancer of split 6 (E(sp1) homolog, Drosophila) | 79816 |
| 7588 | -2.8273 | 0.436911 | 1.55E-03 | 0.01361 | 0.460491 | H200013048 | NOXA1 | NADPH oxidase activator 1 | 10811 |
| 20840 | -2.3702 | 0.501248 | 5.33E-03 | 0.024783 | 0.460686 | H200001936 | CHRNA4 | cholinergic receptor, nicotinic, alpha 4 (neuronal) | 1137 |
| 4539 | -2.5915 | 0.46436 | 2.89E-03 | 0.018371 | 0.460762 | H200020358 | NA | NA | 65068 |
| 20920 | -2.7812 | 0.667712 | 1.75E-03 | 0.014412 | 0.460936 | H200005736 | DOK2 | docking protein 2, 56kDa | 9046 |
| 7889 | -1.9329 | 5.635037 | 1.85E-02 | 0.049504 | 0.461054 | H200005893 | TYMP | thymidine phosphorylase | 1890 |
| 12990 | -2.4613 | 0.578543 | 4.13E-03 | 0.022044 | 0.46113 | H200009176 | KCNK16 | potassium channel, subfamily K, member 16 | 83795 |
| 2659 | -2.1299 | 0.921287 | 1.06E-02 | 0.036428 | 0.461233 | H200017842 | RBM11 | RNA binding motif protein 11 | 54033 |
| 14550 | -2.7054 | 0.528956 | 2.13E-03 | 0.015941 | 0.461258 | H200018213 | NUDT14 | nudix (nucleoside diphosphate linked moiety X)-type motif 14 | 256281 |
| 19519 | -1.9726 | 0.745851 | 1.66E-02 | 0.046434 | 0.461575 | H200020681 | NA | NA | - |
| 112 | -2.4051 | 0.608602 | 4.83E-03 | 0.023541 | 0.461826 | H200005118 | JAM2 | junctional adhesion molecule 2 | 58494 |
| 14219 | -2.6155 | 0.457769 | 2.71E-03 | 0.017735 | 0.461834 | H200002603 | RNF31 | ring finger protein 31 | 55072 |
| 19674 | -2.5169 | 0.774891 | 3.55E-03 | 0.020357 | 0.46185 | H200006662 | ARHGEF6 | Rac/Cdc42 guanine nucleotide exchange factor (GEF) 6 | 9459 |
| 8288 | -4.0845 | 0.192632 | 9.95E-05 | 0.005431 | 0.462304 | H200002848 | NA | NA | - |
| 10987 | -2.6291 | 0.440191 | 2.62E-03 | 0.017437 | 0.462331 | H200000881 | NA | NA | - |
| 17841 | -3.5466 | 0.35568 | 2.78E-04 | 0.007165 | 0.462336 | H200000756 | XCL1 | chemokine (C motif) ligand 1 | 6375 |
| 73 | -3.1095 | 0.417218 | 7.63E-04 | 0.00998 | 0.462359 | H200003520 | TOLLIP | toll interacting protein | 54472 |
| 20608 | -2.615 | 0.444894 | 2.72E-03 | 0.017736 | 0.462458 | H200011691 | CCDC101 | coiled-coil domain containing 101 | 112869 |
| 151 | -2.1582 | 0.450961 | 9.76E-03 | 0.034741 | 0.462928 | H200007012 | CCND3 | cyclin D3 | 896 |
| 11953 | -4.1129 | 0.292313 | 9.42E-05 | 0.0054 | 0.463056 | H200003529 | RHBDF2 | rhomboid 5 homolog 2 (Drosophila) | 79651 |
| 6819 | -2.6808 | 0.22806 | 2.28E-03 | 0.016327 | 0.463935 | H200019848 | CHTF8 | CTF8, chromosome transmission fidelity factor 8 homolog (S. cerevisiae) | 54921 |
| 10733 | -2.811 | 0.502962 | 1.62E-03 | 0.013892 | 0.464194 | H200010608 | TRIM67 | tripartite motif containing 67 | 440730 |
| 10449 | -3.248 | 0.256568 | 5.53E-04 | 0.008974 | 0.464294 | H200018683 | NA | NA | - |
| 4998 | -4.3099 | 0.387823 | 6.79E-05 | 0.004858 | 0.46438 | H200020305 | PARP10 | poly (ADP-ribose) polymerase family, member 10 | 84875 |
| 15921 | -3.5682 | 0.35582 | 2.65E-04 | 0.006955 | 0.464403 | H200018348 | NA | NA | - |
| 16841 | -1.8609 | 0.632636 | 2.28E-02 | 0.055409 | 0.464626 | H200018621 | NA | NA | - |
| 6717 | -2.0549 | 0.458505 | 1.31E-02 | 0.040613 | 0.464677 | H200015216 | NADK | NAD kinase | 65220 |
| 10501 | -2.5485 | 0.444739 | 3.26E-03 | 0.019458 | 0.464852 | H200021295 | NA | NA | - |
| 4710 | -3.2326 | 0.454092 | 5.73E-04 | 0.009191 | 0.464857 | H200006625 | PTPN5 | protein tyrosine phosphatase, non-receptor type 5 (striatum-enriched) | 84867 |
| 16747 | -2.2369 | 0.440489 | 7.80E-03 | 0.030699 | 0.464979 | H200014085 | GGA2 | golgi-associated, gamma adaptin ear containing, ARF binding protein 2 | 23062 |
| 9031 | -2.6599 | 1.023072 | 2.41E-03 | 0.016864 | 0.465008 | H200016617 | TAPBPL | TAP binding protein-like | 55080 |
| 7104 | -2.6079 | 1.036724 | 2.77E-03 | 0.017955 | 0.465024 | H200011779 | FCGRT | Fc fragment of IgG, receptor, transporter, alpha | 2217 |
| 14128 | -2.1195 | 0.668676 | 1.09E-02 | 0.037024 | 0.465402 | H200019804 | NA | NA | - |
| 11644 | -4.8018 | 0.202329 | 3.09E-05 | 0.004028 | 0.465421 | H200010482 | GNB2 | guanine nucleotide binding protein (G protein), beta polypeptide 2 | 2783 |
| 16678 | -2.5037 | 0.433827 | 3.68E-03 | 0.020694 | 0.46546 | H200010695 | CD3D | CD3d molecule, delta (CD3-TCR complex) | 915 |
| 19572 | -3.4529 | 0.389535 | 3.47E-04 | 0.007612 | 0.465498 | H200001746 | RAPGEF1 | Rap guanine nucleotide exchange factor (GEF) 1 | 2889 |
| 5644 | -3.2093 | 0.387379 | 6.02E-04 | 0.009326 | 0.465699 | H200007634 | TSSC4 | tumor suppressing subtransferable candidate 4 | 10078 |
| 11473 | -3.5345 | 0.322632 | 2.87E-04 | 0.00735 | 0.465792 | H200002472 | SYNRG | synergin, gamma | 11276 |
| 3313 | -2.7662 | 0.304244 | 1.81E-03 | 0.014755 | 0.465844 | H200005622 | KCNJ10 | potassium inwardly-rectifying channel, subfamily J, member 10 | 3766 |
| 12315 | -2.2039 | 0.61427 | 8.56E-03 | 0.032249 | 0.466152 | H200020653 | NA | NA | - |
| 19380 | -2.7259 | 0.344885 | 2.02E-03 | 0.0156 | 0.466224 | H200014191 | SNX29 | sorting nexin 29 | 92017 |
| 7899 | -2.0434 | 1.561133 | 1.35E-02 | 0.041499 | 0.466295 | H200006297 | LCP1 | lymphocyte cytosolic protein 1 (L-plastin) | 3936 |
| 17267 | -2.0169 | 0.379724 | 1.46E-02 | 0.043396 | 0.466302 | H200017060 | ST6GALNAC5 | ST6 (alpha-N-acetyl-neuraminyl-2,3-beta-galactosyl-1,3)-N-acetylgalactosaminide alpha-2,6-sialyltransferase 5 | 81849 |
| 5622 | -3.4713 | 0.371953 | 3.33E-04 | 0.007506 | 0.4664 | H200006518 | NDST2 | N-deacetylase/N-sulfotransferase (heparan glucosaminyl) 2 | 8509 |
| 21351 | -2.9939 | 0.423555 | 1.01E-03 | 0.011196 | 0.466521 | H200004531 | DUSP8 | dual specificity phosphatase 8 | 1850 |
| 1035 | -2.0424 | 0.526818 | 1.36E-02 | 0.041563 | 0.466647 | H200005836 | GNGT1 | guanine nucleotide binding protein (G protein), gamma transducing activity polypeptide 1 | 2792 |
| 13257 | -2.2026 | 0.468293 | 8.59E-03 | 0.032321 | 0.466788 | H200000335 | NA | NA | - |
| 3491 | -3.8169 | 0.206477 | 1.61E-04 | 0.005936 | 0.46688 | H200014006 | CHKB | choline kinase beta | 1120 |
| 11853 | -3.0801 | 0.253125 | 8.18E-04 | 0.010318 | 0.467045 | H200020380 | ACADS | acyl-CoA dehydrogenase, C-2 to C-3 short chain | 35 |
| 20890 | -4.0908 | 0.238703 | 9.83E-05 | 0.005431 | 0.467124 | H200004240 | NA | NA | 153277 |
| 17851 | -3.2672 | 0.459852 | 5.27E-04 | 0.008857 | 0.467203 | H200001444 | ETHE1 | ethylmalonic encephalopathy 1 | 23474 |
| 11577 | -2.0313 | 0.733171 | 1.40E-02 | 0.04233 | 0.467465 | H200007412 | ZBTB7B | zinc finger and BTB domain containing 7B | 51043 |
| 8713 | -3.3749 | 0.232066 | 4.11E-04 | 0.007963 | 0.467556 | H200001725 | HDAC5 | histone deacetylase 5 | 10014 |
| 11936 | -2.2268 | 0.336497 | 8.03E-03 | 0.031179 | 0.467966 | H200002467 | ANKRD13A | ankyrin repeat domain 13A | 88455 |
| 14824 | -2.8334 | 0.491132 | 1.53E-03 | 0.013518 | 0.468092 | H200009294 | NA | NA | - |
| 13037 | -2.7902 | 0.391136 | 1.71E-03 | 0.014256 | 0.468096 | H200011450 | ABHD15 | abhydrolase domain containing 15 | 116236 |
| 11323 | -2.9458 | 0.415742 | 1.14E-03 | 0.011769 | 0.468181 | H200016841 | TRPM5 | transient receptor potential cation channel, subfamily M, member 5 | 29850 |
| 1603 | -3.6368 | 0.564997 | 2.30E-04 | 0.00665 | 0.468312 | H200011251 | LOC157503 | uncharacterized LOC157503 | 157503 |
| 6961 | -2.9129 | 0.135354 | 1.24E-03 | 0.01225 | 0.468432 | H200005241 | NRM | nurim (nuclear envelope membrane protein) | 11270 |
| 5516 | -1.9323 | 0.858985 | 1.86E-02 | 0.049532 | 0.468624 | H200001554 | NA | NA | - |
| 8500 | -3.368 | 0.359887 | 4.19E-04 | 0.008048 | 0.468628 | H200013060 | NA | NA | - |
| 1212 | -1.9502 | 1.184128 | 1.77E-02 | 0.048233 | 0.468654 | H200014202 | RGS16 | regulator of G-protein signaling 16 | 6004 |
| 3738 | -2.8325 | 0.464298 | 1.53E-03 | 0.013535 | 0.468713 | H200003905 | EYA2 | eyes absent homolog 2 (Drosophila) | 2139 |
| 8154 | -1.795 | 0.694222 | 2.75E-02 | 0.061895 | 0.46874 | H200018439 | UBR4 | ubiquitin protein ligase E3 component n-recognin 4 | 23352 |
| 19427 | -2.6136 | 0.326055 | 2.73E-03 | 0.017762 | 0.469252 | H200016453 | CYFIP2 | cytoplasmic FMR1 interacting protein 2 | 26999 |
| 12145 | -1.8984 | 0.614062 | 2.05E-02 | 0.052205 | 0.469422 | H200012649 | RLN2 | relaxin 2 | 6019 |
| 1668 | -2.0567 | 0.735157 | 1.30E-02 | 0.040543 | 0.469496 | H200014297 | LMO2 | LIM domain only 2 (rhombotin-like 1) | 4005 |
| 18183 | -1.9327 | 0.741574 | 1.85E-02 | 0.049518 | 0.469752 | H200017072 | RGS5 | regulator of G-protein signaling 5 | 8490 |
| 2018 | -1.9676 | 0.800359 | 1.68E-02 | 0.046882 | 0.469857 | H200009131 | MIA2 | melanoma inhibitory activity 2 | 117153 |
| 17078 | -2.0948 | 1.060805 | 1.17E-02 | 0.038327 | 0.47001 | H200008266 | DUSP10 | dual specificity phosphatase 10 | 11221 |
| 9553 | -2.3715 | 0.511842 | 5.31E-03 | 0.024727 | 0.470175 | H200019763 | KCNG4 | potassium voltage-gated channel, subfamily G, member 4 | 93107 |
| 6045 | -1.999 | 0.647311 | 1.54E-02 | 0.044643 | 0.470187 | H200005229 | CNPY3 | canopy 3 homolog (zebrafish) | 10695 |
| 1734 | -2.9145 | 0.373364 | 1.23E-03 | 0.012235 | 0.470548 | H200017361 | IL36B | interleukin 36, beta | 27177 |
| 7548 | -2.5518 | 0.541919 | 3.23E-03 | 0.019402 | 0.470901 | H200011148 | RBP1 | retinol binding protein 1, cellular | 5947 |
| 20632 | -2.6575 | 0.385883 | 2.43E-03 | 0.016906 | 0.470918 | H200012873 | IQSEC2 | IQ motif and Sec7 domain 2 | 23096 |
| 4612 | -4.0121 | 0.381503 | 1.13E-04 | 0.005439 | 0.470931 | H200002041 | CERS4 | ceramide synthase 4 | 79603 |
| 4348 | -2.8253 | 0.597005 | 1.56E-03 | 0.013655 | 0.47103 | H200011244 | GRK1 | G protein-coupled receptor kinase 1 | 6011 |
| 8620 | -1.9222 | 0.160447 | 1.91E-02 | 0.050241 | 0.47128 | H200018760 | ENKD1 | enkurin domain containing 1 | 84080 |
| 15566 | -1.9948 | 0.70805 | 1.56E-02 | 0.044842 | 0.471412 | H200001302 | SPAG16 | sperm associated antigen 16 | 79582 |
| 13608 | -2.1458 | 0.424481 | 1.01E-02 | 0.035429 | 0.47147 | H200017037 | CXorf57 | chromosome X open reading frame 57 | 55086 |
| 19025 | -2.5432 | 0.542817 | 3.30E-03 | 0.019584 | 0.471516 | H200018816 | NA | NA | - |
| 15315 | -2.5324 | 0.467401 | 3.40E-03 | 0.019825 | 0.471689 | H200011235 | NA | NA | - |
| 20987 | -2.3381 | 0.344598 | 5.84E-03 | 0.026111 | 0.471702 | H200008806 | C3 | complement component 3 | 718 |
| 3695 | -2.6076 | 0.223153 | 2.77E-03 | 0.017955 | 0.47184 | H200001691 | PARVB | parvin, beta | 29780 |
| 2372 | -2.7733 | 0.364916 | 1.78E-03 | 0.014589 | 0.471884 | H200004168 | KCNJ4 | potassium inwardly-rectifying channel, subfamily J, member 4 | 3761 |
| 562 | -4.1157 | 0.178703 | 9.39E-05 | 0.0054 | 0.472035 | H200005141 | EPS8L2 | EPS8-like 2 | 64787 |
| 7782 | -1.8944 | 1.547844 | 2.07E-02 | 0.052467 | 0.472113 | H200000627 | LCP2 | lymphocyte cytosolic protein 2 (SH2 domain containing leukocyte protein of 76kDa) | 3937 |
| 18171 | -3.4745 | 0.38581 | 3.32E-04 | 0.007506 | 0.4723 | H200016644 | GZMM | granzyme M (lymphocyte met-ase 1) | 3004 |
| 13870 | -2.9167 | 0.45361 | 1.23E-03 | 0.012195 | 0.472344 | H200007620 | ASNA1 | arsA arsenite transporter, ATP-binding, homolog 1 (bacterial) | 439 |
| 1841 | -3.3027 | 0.47814 | 4.85E-04 | 0.008593 | 0.47261 | H200000765 | MRPL23 | mitochondrial ribosomal protein L23 | 6150 |
| 1520 | -3.7874 | 0.273485 | 1.71E-04 | 0.006053 | 0.472615 | H200007125 | KRT20 | keratin 20 | 54474 |
| 691 | -2.5807 | 0.31947 | 2.98E-03 | 0.018545 | 0.473275 | H200011239 | C1orf115 | chromosome 1 open reading frame 115 | 79762 |
| 3501 | -3.348 | 0.324146 | 4.39E-04 | 0.008301 | 0.473344 | H200014410 | TNFAIP8L1 | tumor necrosis factor, alpha-induced protein 8-like 1 | 126282 |
| 19677 | -3.4024 | 0.292683 | 3.85E-04 | 0.00776 | 0.473469 | H200006692 | MPG | N-methylpurine-DNA glycosylase | 4350 |
| 3110 | -3.8378 | 0.292981 | 1.54E-04 | 0.005935 | 0.47371 | H200017503 | COMMD9 | COMM domain containing 9 | 29099 |
| 18505 | -3.4602 | 0.363901 | 3.41E-04 | 0.007537 | 0.473752 | H200011953 | TEX28 | testis expressed 28 | 1527 |
| 4455 | -1.9153 | 0.664008 | 1.95E-02 | 0.050843 | 0.474039 | H200016226 | LINC00626 | long intergenic non-protein coding RNA 626 | 79100 |
| 3471 | -3.5097 | 0.413686 | 3.05E-04 | 0.007429 | 0.474224 | H200012914 | ADCK4 | aarF domain containing kinase 4 | 79934 |
| 1157 | -3.3914 | 0.257348 | 3.96E-04 | 0.007814 | 0.474333 | H200011560 | ING4 | inhibitor of growth family, member 4 | 51147 |
| 10656 | -2.4953 | 0.430337 | 3.76E-03 | 0.020933 | 0.474394 | H200006838 | NA | NA | - |
| 14278 | -2.0595 | 0.914171 | 1.29E-02 | 0.040304 | 0.474409 | H200005293 | CASQ2 | calsequestrin 2 (cardiac muscle) | 845 |
| 6759 | -2.457 | 0.412099 | 4.18E-03 | 0.022124 | 0.47442 | H200017140 | NA | NA | - |
| 6246 | -2.8274 | 0.926175 | 1.55E-03 | 0.01361 | 0.474528 | H200014735 | NCF4 | neutrophil cytosolic factor 4, 40kDa | 4689 |
| 2031 | -2.0588 | 0.351336 | 1.29E-02 | 0.040374 | 0.47454 | H200009577 | LIN7B | lin-7 homolog B (C. elegans) | 64130 |
| 17311 | -3.4304 | 0.231968 | 3.64E-04 | 0.007657 | 0.474965 | H200019292 | LPAR5 | lysophosphatidic acid receptor 5 | 57121 |
| 11128 | -2.6409 | 0.386739 | 2.53E-03 | 0.017145 | 0.475136 | H200007395 | NA | NA | 23056 |
| 15611 | -3.9656 | 0.27101 | 1.22E-04 | 0.005642 | 0.475313 | H200003552 | MRPL38 | mitochondrial ribosomal protein L38 | 64978 |
| 724 | -2.4644 | 0.385218 | 4.10E-03 | 0.02198 | 0.475558 | H200012765 | FBXO46 | F-box protein 46 | 23403 |
| 14639 | -2.4931 | 0.485127 | 3.78E-03 | 0.020998 | 0.475671 | H200000548 | C5AR1 | complement component 5a receptor 1 | 728 |
| 9419 | -3.2966 | 0.314121 | 4.92E-04 | 0.008622 | 0.475947 | H200013327 | NA | NA | - |
| 10348 | -3.8067 | 0.627861 | 1.64E-04 | 0.005937 | 0.475962 | H200013773 | MGAT1 | mannosyl (alpha-1,3-)-glycoprotein beta-1,2-N-acetylglucosaminyltransferase | 4245 |
| 19562 | -2.0426 | 0.695893 | 1.36E-02 | 0.041563 | 0.475963 | H200001342 | NA | NA | 147343 |
| 8912 | -3.3339 | 0.319219 | 4.51E-04 | 0.008402 | 0.475997 | H200010923 | NA | NA | - |
| 12013 | -4.9161 | 0.168914 | 2.56E-05 | 0.004028 | 0.476005 | H200006237 | NAPA | N-ethylmaleimide-sensitive factor attachment protein, alpha | 8775 |
| 6008 | -2.2435 | 0.561943 | 7.65E-03 | 0.030476 | 0.476124 | H200003359 | FKBP11 | FK506 binding protein 11, 19 kDa | 51303 |
| 15081 | -3.4497 | 0.254663 | 3.49E-04 | 0.007612 | 0.476133 | H200000191 | FPGS | folylpolyglutamate synthase | 2356 |
| 6240 | -1.9252 | 0.629484 | 1.89E-02 | 0.050002 | 0.476279 | H200014379 | MORN1 | MORN repeat containing 1 | 79906 |
| 17567 | -3.6445 | 0.383556 | 2.27E-04 | 0.00661 | 0.476677 | H200009519 | TMEM219 | transmembrane protein 219 | 124446 |
| 6364 | -2.1624 | 0.526586 | 9.64E-03 | 0.034624 | 0.477005 | H200020127 | NA | NA | - |
| 5602 | -3.4295 | 0.283374 | 3.65E-04 | 0.007659 | 0.477038 | H200005710 | EVI2A | ecotropic viral integration site 2A | 2123 |
| 6637 | -3.5969 | 0.359514 | 2.50E-04 | 0.00677 | 0.477089 | H200011416 | A4GALT | alpha 1,4-galactosyltransferase | 53947 |
| 9940 | -2.3927 | 0.495487 | 4.99E-03 | 0.024024 | 0.477118 | H200016326 | NA | NA | - |
| 19922 | -1.9055 | 0.637876 | 2.01E-02 | 0.051617 | 0.477381 | H200018442 | NA | NA | - |
| 16661 | -4.3875 | 0.217572 | 5.92E-05 | 0.00473 | 0.477432 | H200009929 | CELF5 | CUGBP, Elav-like family member 5 | 60680 |
| 9999 | -3.5762 | 0.35973 | 2.60E-04 | 0.006895 | 0.477459 | H200019312 | AP1B1 | adaptor-related protein complex 1, beta 1 subunit | 162 |
| 7067 | -1.9796 | 2.442866 | 1.62E-02 | 0.045851 | 0.477576 | H200010205 | ADAM8 | ADAM metallopeptidase domain 8 | 101 |
| 5694 | -1.8106 | 0.630255 | 2.63E-02 | 0.060286 | 0.477736 | H200009938 | NA | NA | - |
| 16313 | -1.8892 | 0.474253 | 2.10E-02 | 0.052923 | 0.477751 | H200015403 | MAPKAPK3 | mitogen-activated protein kinase-activated protein kinase 3 | 7867 |
| 1185 | -1.8888 | 0.948812 | 2.10E-02 | 0.052958 | 0.477869 | H200013032 | IL27RA | interleukin 27 receptor, alpha | 9466 |
| 15056 | -3.7448 | 0.239794 | 1.86E-04 | 0.006183 | 0.477977 | H200020314 | NA | NA | - |
| 13485 | -2.5154 | 0.38969 | 3.56E-03 | 0.020385 | 0.47814 | H200011307 | SOCS7 | suppressor of cytokine signaling 7 | 30837 |
| 812 | -1.8948 | 0.572554 | 2.07E-02 | 0.052415 | 0.478314 | H200016945 | PITPNM2 | phosphatidylinositol transfer protein, membrane-associated 2 | 57605 |
| 8849 | -2.9606 | 0.357543 | 1.10E-03 | 0.01164 | 0.478661 | H200008185 | CAMSAP1 | calmodulin regulated spectrin-associated protein 1 | 157922 |
| 19952 | -3.8545 | 0.275044 | 1.49E-04 | 0.005885 | 0.478804 | H200019654 | SPSB2 | splA/ryanodine receptor domain and SOCS box containing 2 | 84727 |
| 5361 | -2.7293 | 0.657192 | 2.00E-03 | 0.015533 | 0.479017 | H200016166 | FGF19 | fibroblast growth factor 19 | 9965 |
| 20038 | -3.599 | 0.185603 | 2.49E-04 | 0.00677 | 0.479267 | H200002310 | RANGRF | RAN guanine nucleotide release factor | 29098 |
| 12820 | -1.863 | 0.557612 | 2.26E-02 | 0.055218 | 0.479279 | H200001172 | CADM3 | cell adhesion molecule 3 | 57863 |
| 336 | -2.8779 | 0.824335 | 1.36E-03 | 0.012747 | 0.479449 | H200015758 | HNF4G | hepatocyte nuclear factor 4, gamma | 3174 |
| 12220 | -2.5199 | 0.444811 | 3.52E-03 | 0.020261 | 0.479733 | H200016099 | NA | NA | - |
| 12363 | -3.5201 | 0.195642 | 2.97E-04 | 0.007417 | 0.479897 | H200001368 | C9orf142 | chromosome 9 open reading frame 142 | 286257 |
| 20849 | -2.2243 | 0.385806 | 8.09E-03 | 0.031232 | 0.479971 | H200002322 | KLHDC8B | kelch domain containing 8B | 200942 |
| 10843 | -2.3928 | 0.439622 | 4.99E-03 | 0.024024 | 0.480026 | H200015904 | TP73 | tumor protein p73 | 7161 |
| 8302 | -4.0015 | 0.218243 | 1.15E-04 | 0.005439 | 0.480128 | H200003584 | LDB1 | LIM domain binding 1 | 8861 |
| 6411 | -4.573 | 0.222117 | 4.38E-05 | 0.004437 | 0.480352 | H200000468 | MYL3 | myosin, light chain 3, alkali; ventricular, skeletal, slow | 4634 |
| 11922 | -2.4202 | 0.307531 | 4.63E-03 | 0.023035 | 0.480468 | H200002015 | CTSF | cathepsin F | 8722 |
| 6827 | -3.4458 | 0.388032 | 3.53E-04 | 0.007621 | 0.480645 | H200020228 | OSR2 | odd-skipped related 2 (Drosophila) | 116039 |
| 9630 | -3.7799 | 0.208983 | 1.73E-04 | 0.006053 | 0.480687 | H200001814 | ECHDC2 | enoyl CoA hydratase domain containing 2 | 55268 |
| 18234 | -3.1879 | 0.570429 | 6.33E-04 | 0.0094 | 0.480704 | H200019678 | CCDC97 | coiled-coil domain containing 97 | 90324 |
| 6360 | -3.4057 | 0.248786 | 3.82E-04 | 0.007754 | 0.480803 | H200020079 | NA | NA | - |
| 16653 | -3.3082 | 0.466537 | 4.78E-04 | 0.008593 | 0.480986 | H200009549 | DEDD2 | death effector domain containing 2 | 162989 |
| 3460 | -3.1921 | 0.305536 | 6.27E-04 | 0.009384 | 0.481239 | H200012492 | HCN2 | hyperpolarization activated cyclic nucleotide-gated potassium channel 2 | 610 |
| 13120 | -2.3455 | 0.528504 | 5.72E-03 | 0.025809 | 0.481288 | H200015280 | DNAH5 | dynein, axonemal, heavy chain 5 | 1767 |
| 21428 | -3.905 | 0.155485 | 1.35E-04 | 0.005801 | 0.481405 | H200008301 | MEPCE | methylphosphate capping enzyme | 56257 |
| 17472 | -2.6475 | 0.342632 | 2.49E-03 | 0.017043 | 0.481528 | H200004977 | C7orf55 | chromosome 7 open reading frame 55 | 154791 |
| 4026 | -3.1176 | 0.192411 | 7.48E-04 | 0.009958 | 0.481551 | H200017585 | LMTK3 | lemur tyrosine kinase 3 | 114783 |
| 12547 | -2.4557 | 0.424975 | 4.20E-03 | 0.022146 | 0.481661 | H200010108 | NA | NA | - |
| 5753 | -2.0153 | 0.723559 | 1.47E-02 | 0.043498 | 0.48167 | H200012924 | GBGT1 | globoside alpha-1,3-N-acetylgalactosaminyltransferase 1 | 26301 |
| 145 | -2.3062 | 1.602235 | 6.39E-03 | 0.027263 | 0.481798 | H200006940 | CAPG | capping protein (actin filament), gelsolin-like | 822 |
| 14554 | -2.6988 | 0.593602 | 2.17E-03 | 0.016063 | 0.48184 | H200018545 | NA | NA | - |
| 18480 | -2.3152 | 0.535747 | 6.24E-03 | 0.026907 | 0.482227 | H200010765 | C11orf52 | chromosome 11 open reading frame 52 | 91894 |
| 16505 | -2.206 | 0.514082 | 8.51E-03 | 0.032145 | 0.482244 | H200002661 | NA | NA | - |
| 8361 | -1.9926 | 0.303471 | 1.56E-02 | 0.044962 | 0.482261 | H200006570 | ILVBL | ilvB (bacterial acetolactate synthase)-like | 10994 |
| 13002 | -2.6209 | 0.374167 | 2.67E-03 | 0.017651 | 0.482343 | H200009888 | DPYSL5 | dihydropyrimidinase-like 5 | 56896 |
| 790 | -2.6165 | 0.135568 | 2.71E-03 | 0.017724 | 0.48238 | H200015829 | NA | NA | - |
| 9121 | -2.7879 | 0.474285 | 1.72E-03 | 0.014287 | 0.482408 | H200021105 | NA | NA | - |
| 3386 | -2.3024 | 0.488495 | 6.46E-03 | 0.027406 | 0.482779 | H200009048 | NA | NA | - |
| 16570 | -2.7129 | 0.499141 | 2.09E-03 | 0.015809 | 0.483073 | H200005707 | CADM1 | cell adhesion molecule 1 | 23705 |
| 17713 | -2.6176 | 0.489722 | 2.70E-03 | 0.017713 | 0.483287 | H200016383 | CIB3 | calcium and integrin binding family member 3 | 117286 |
| 14718 | -2.0194 | 0.433037 | 1.45E-02 | 0.043158 | 0.483381 | H200004330 | LIMK1 | LIM domain kinase 1 | 3984 |
| 14062 | -3.3198 | 0.254577 | 4.66E-04 | 0.00858 | 0.483444 | H200016740 | C1orf170 | chromosome 1 open reading frame 170 | 84808 |
| 3778 | -2.7464 | 0.407735 | 1.91E-03 | 0.015139 | 0.48354 | H200005805 | CRYGC | crystallin, gamma C | 1420 |
| 19123 | -2.2944 | 0.61477 | 6.61E-03 | 0.027832 | 0.48358 | H200002013 | NA | NA | - |
| 11712 | -3.7524 | 0.221986 | 1.82E-04 | 0.006164 | 0.483805 | H200013570 | CRYBA2 | crystallin, beta A2 | 1412 |
| 6316 | -1.8489 | 0.7639 | 2.36E-02 | 0.05661 | 0.484365 | H200017847 | CBLN4 | cerebellin 4 precursor | 140689 |
| 8983 | -2.4719 | 0.870442 | 4.02E-03 | 0.021726 | 0.484501 | H200014337 | MALL | mal, T-cell differentiation protein-like | 7851 |
| 448 | -3.441 | 0.248554 | 3.57E-04 | 0.007621 | 0.484569 | H200021078 | GTPBP6 | GTP binding protein 6 (putative) | 8225 |
| 7358 | -3.3762 | 0.378449 | 4.10E-04 | 0.007963 | 0.484572 | H200002052 | BCL2L14 | BCL2-like 14 (apoptosis facilitator) | 79370 |
| 1303 | -2.5818 | 0.323317 | 2.97E-03 | 0.018531 | 0.484634 | H200018424 | ADGB | androglobin | 79747 |
| 14443 | -1.811 | 0.853889 | 2.63E-02 | 0.06028 | 0.484666 | H200013243 | ZNF526 | zinc finger protein 526 | 116115 |
| 17380 | -2.2396 | 0.394597 | 7.74E-03 | 0.030653 | 0.484744 | H200000465 | CD1D | CD1d molecule | 912 |
| 17045 | -4.0975 | 0.270044 | 9.68E-05 | 0.005431 | 0.484795 | H200006444 | DNAJB2 | DnaJ (Hsp40) homolog, subfamily B, member 2 | 3300 |
| 14761 | -2.4449 | 0.184729 | 4.33E-03 | 0.022336 | 0.484802 | H200006556 | PRKCZ | protein kinase C, zeta | 5590 |
| 3873 | -1.8971 | 0.673897 | 2.05E-02 | 0.052283 | 0.484819 | H200010359 | TLX1 | T-cell leukemia homeobox 1 | 3195 |
| 20291 | -1.8832 | 0.489842 | 2.14E-02 | 0.053355 | 0.484858 | H200016008 | VPREB1 | pre-B lymphocyte 1 | 7441 |
| 21216 | -3.022 | 0.354021 | 9.43E-04 | 0.010874 | 0.485022 | H200019796 | WNT3A | wingless-type MMTV integration site family, member 3A | 89780 |
| 12256 | -2.0198 | 0.646825 | 1.45E-02 | 0.043116 | 0.485224 | H200017667 | NA | NA | - |
| 12517 | -2.1033 | 0.496266 | 1.14E-02 | 0.037903 | 0.485378 | H200008612 | WWC2-AS2 | WWC2 antisense RNA 2 | 152641 |
| 18243 | -2.2737 | 0.397057 | 7.01E-03 | 0.028833 | 0.485521 | H200020064 | NA | NA | - |
| 8529 | -2.3305 | 0.342618 | 5.97E-03 | 0.026365 | 0.485534 | H200014550 | C19orf26 | chromosome 19 open reading frame 26 | 255057 |
| 835 | -3.7749 | 0.139453 | 1.75E-04 | 0.006055 | 0.485584 | H200018079 | NA | NA | - |
| 497 | -2.6787 | 0.477696 | 2.29E-03 | 0.016399 | 0.485597 | H200002095 | AMIGO1 | adhesion molecule with Ig-like domain 1 | 57463 |
| 9220 | -1.9929 | 0.609479 | 1.56E-02 | 0.044948 | 0.485628 | H200003833 | ASPRV1 | aspartic peptidase, retroviral-like 1 | 151516 |
| 4412 | -3.89 | 0.24977 | 1.40E-04 | 0.005831 | 0.485735 | H200014284 | SIRT7 | sirtuin 7 | 51547 |
| 21316 | -2.3705 | 0.462904 | 5.33E-03 | 0.024783 | 0.485785 | H200002981 | RTBDN | retbindin | 83546 |
| 15633 | -2.3787 | 0.437456 | 5.20E-03 | 0.024471 | 0.486085 | H200004668 | CNNM3 | cyclin M3 | 26505 |
| 19898 | -3.1619 | 0.364176 | 6.73E-04 | 0.009642 | 0.486162 | H200017302 | TYMP | thymidine phosphorylase | 1890 |
| 16399 | -2.354 | 0.43697 | 5.58E-03 | 0.025463 | 0.486255 | H200019275 | C16orf46 | chromosome 16 open reading frame 46 | 123775 |
| 16778 | -2.3254 | 0.417455 | 6.06E-03 | 0.026619 | 0.486357 | H200015587 | IBA57 | IBA57, iron-sulfur cluster assembly homolog (S. cerevisiae) | 200205 |
| 14538 | -2.2858 | 0.464925 | 6.77E-03 | 0.028182 | 0.486397 | H200017785 | PLXNB3 | plexin B3 | 5365 |
| 15084 | -2.1775 | 0.525999 | 9.22E-03 | 0.033723 | 0.486829 | H200000221 | CD80 | CD80 molecule | 941 |
| 16158 | -3.6738 | 0.175292 | 2.12E-04 | 0.006416 | 0.487275 | H200007857 | NPRL2 | nitrogen permease regulator-like 2 (S. cerevisiae) | 10641 |
| 1622 | -2.4374 | 0.576584 | 4.42E-03 | 0.022443 | 0.487306 | H200012041 | CUL9 | cullin 9 | 23113 |
| 1065 | -2.0988 | 0.522712 | 1.15E-02 | 0.038151 | 0.487544 | H200007332 | BMP10 | bone morphogenetic protein 10 | 27302 |
| 3672 | -2.4761 | 0.466078 | 3.97E-03 | 0.02157 | 0.487577 | H200000557 | PRF1 | perforin 1 (pore forming protein) | 5551 |
| 2224 | -3.3054 | 0.287272 | 4.81E-04 | 0.008593 | 0.487616 | H200018703 | SLC48A1 | solute carrier family 48 (heme transporter), member 1 | 55652 |
| 1281 | -2.5838 | 0.259072 | 2.96E-03 | 0.018506 | 0.487661 | H200017592 | ROM1 | retinal outer segment membrane protein 1 | 6094 |
| 19507 | -1.8135 | 0.86504 | 2.61E-02 | 0.060084 | 0.48775 | H200020253 | NA | NA | - |
| 21079 | -3.4322 | 0.339586 | 3.63E-04 | 0.007653 | 0.487754 | H200013318 | FRAT2 | frequently rearranged in advanced T-cell lymphomas 2 | 23401 |
| 13443 | -2.3947 | 0.560907 | 4.97E-03 | 0.023948 | 0.487904 | H200009099 | NA | NA | 80098 |
| 19779 | -2.3796 | 0.310729 | 5.18E-03 | 0.024471 | 0.488107 | H200011608 | CRYL1 | crystallin, lambda 1 | 51084 |
| 4313 | -3.3371 | 0.330161 | 4.48E-04 | 0.008358 | 0.488191 | H200009694 | NA | NA | - |
| 987 | -2.215 | 0.416339 | 8.30E-03 | 0.031627 | 0.488307 | H200003556 | SSSCA1 | Sjogren syndrome/scleroderma autoantigen 1 | 10534 |
| 8695 | -2.8175 | 0.311437 | 1.59E-03 | 0.013777 | 0.488339 | H200000657 | GAST | gastrin | 2520 |
| 16992 | -2.497 | 0.416156 | 3.74E-03 | 0.020881 | 0.488698 | H200004110 | MED16 | mediator complex subunit 16 | 10025 |
| 6962 | -1.8328 | 0.420772 | 2.47E-02 | 0.057996 | 0.48876 | H200005247 | AHNAK2 | AHNAK nucleoprotein 2 | 113146 |
| 12851 | -3.042 | 0.313614 | 8.98E-04 | 0.01067 | 0.488777 | H200002686 | RILP | Rab interacting lysosomal protein | 83547 |
| 21354 | -3.4027 | 0.244588 | 3.84E-04 | 0.00776 | 0.488802 | H200004857 | ARHGEF11 | Rho guanine nucleotide exchange factor (GEF) 11 | 9826 |
| 9187 | -3.1477 | 0.39696 | 6.96E-04 | 0.009819 | 0.488914 | H200002307 | FAM58A | family with sequence similarity 58, member A | 92002 |
| 4434 | -2.7785 | 0.236647 | 1.76E-03 | 0.014473 | 0.488985 | H200015400 | ZNF41 | zinc finger protein 41 | 7592 |
| 1805 | -1.9671 | 0.580727 | 1.68E-02 | 0.046913 | 0.489029 | H200020775 | NA | NA | - |
| 21614 | -3.3784 | 0.226656 | 4.08E-04 | 0.007963 | 0.489177 | H200017065 | FOXP1 | forkhead box P1 | 27086 |
| 882 | -1.9768 | 0.461406 | 1.64E-02 | 0.046097 | 0.489199 | H200020341 | NA | NA | - |
| 15733 | -2.7111 | 0.077164 | 2.10E-03 | 0.015839 | 0.489227 | H200009276 | ATHL1 | ATH1, acid trehalase-like 1 (yeast) | 80162 |
| 6245 | -3.3981 | 0.182032 | 3.89E-04 | 0.00776 | 0.489367 | H200014729 | ECH1 | enoyl CoA hydratase 1, peroxisomal | 1891 |
| 10183 | -2.0892 | 0.640274 | 1.19E-02 | 0.038613 | 0.489665 | H200006119 | ANXA11 | annexin A11 | 311 |
| 439 | -2.8218 | 0.314733 | 1.58E-03 | 0.01371 | 0.489854 | H200020692 | NA | NA | - |
| 6381 | -2.2139 | 0.190426 | 8.33E-03 | 0.03172 | 0.490013 | H200021189 | ZNF276 | zinc finger protein 276 | 92822 |
| 3193 | -1.7955 | 0.827481 | 2.75E-02 | 0.061827 | 0.490111 | H2NC000006 | NA | NA | - |
| 10689 | -1.9296 | 0.550729 | 1.87E-02 | 0.049667 | 0.490223 | H200008660 | TTC12 | tetratricopeptide repeat domain 12 | 54970 |
| 20654 | -2.3179 | 0.412075 | 6.19E-03 | 0.026798 | 0.490417 | H200014019 | BCL6 | B-cell CLL/lymphoma 6 | 604 |
| 8710 | -2.4227 | 0.369318 | 4.59E-03 | 0.02296 | 0.490526 | H200001399 | CORO7 | coronin 7 | 79585 |
| 19115 | -1.993 | 0.450113 | 1.56E-02 | 0.044948 | 0.490614 | H200001633 | RNASET2 | ribonuclease T2 | 8635 |
| 12302 | -1.8715 | 0.541816 | 2.21E-02 | 0.054414 | 0.490622 | H200019923 | GGT7 | gamma-glutamyltransferase 7 | 2686 |
| 12804 | -2.4334 | 0.338386 | 4.46E-03 | 0.022579 | 0.490632 | H200000412 | BIRC5 | baculoviral IAP repeat containing 5 | 332 |
| 7832 | -2.3659 | 0.527062 | 5.40E-03 | 0.024946 | 0.4908 | H200002931 | NA | NA | - |
| 21236 | -2.3771 | 0.541332 | 5.22E-03 | 0.024535 | 0.490836 | H200020604 | NA | NA | - |
| 7339 | -2.6952 | 0.368519 | 2.20E-03 | 0.01616 | 0.490855 | H200001262 | CSRNP1 | cysteine-serine-rich nuclear protein 1 | 64651 |
| 4802 | -3.4406 | 0.329588 | 3.58E-04 | 0.007621 | 0.491179 | H200011137 | RPUSD1 | RNA pseudouridylate synthase domain containing 1 | 113000 |
| 637 | -3.2098 | 0.229327 | 6.01E-04 | 0.009326 | 0.49118 | H200008603 | SLC15A2 | solute carrier family 15 (H+/peptide transporter), member 2 | 6565 |
| 13505 | -3.5531 | 0.105803 | 2.74E-04 | 0.007084 | 0.491241 | H200012115 | PNPLA2 | patatin-like phospholipase domain containing 2 | 57104 |
| 7128 | -2.341 | 0.234759 | 5.79E-03 | 0.026006 | 0.49158 | H200012919 | ALKBH5 | alkB, alkylation repair homolog 5 (E. coli) | 54890 |
| 10840 | -3.3076 | 0.290846 | 4.79E-04 | 0.008593 | 0.491642 | H200015578 | AMN | amnion associated transmembrane protein | 81693 |
| 8338 | -2.2387 | 0.464714 | 7.76E-03 | 0.030653 | 0.491659 | H200005436 | SASH3 | SAM and SH3 domain containing 3 | 54440 |
| 17562 | -2.063 | 0.730834 | 1.28E-02 | 0.040063 | 0.491769 | H200009181 | NA | NA | 343629 |
| 5079 | -3.3751 | 0.174869 | 4.11E-04 | 0.007963 | 0.492159 | H200002558 | BCL7B | B-cell CLL/lymphoma 7B | 9275 |
| 19918 | -3.1796 | 0.307021 | 6.46E-04 | 0.009443 | 0.492504 | H200018110 | NA | NA | - |
| 12747 | -2.1125 | 0.587542 | 1.11E-02 | 0.037401 | 0.492702 | H200019608 | COL27A1 | collagen, type XXVII, alpha 1 | 85301 |
| 16584 | -2.3469 | 0.31235 | 5.69E-03 | 0.025802 | 0.492748 | H200006159 | GALT | galactose-1-phosphate uridylyltransferase | 2592 |
| 19769 | -2.5677 | 0.430211 | 3.09E-03 | 0.018905 | 0.492838 | H200010920 | NA | NA | - |
| 4282 | -2.5671 | 0.370976 | 3.10E-03 | 0.018905 | 0.492841 | H200008180 | PPP2R1A | protein phosphatase 2, regulatory subunit A, alpha | 5518 |
| 7872 | -2.6425 | 0.421985 | 2.52E-03 | 0.017125 | 0.492983 | H200004831 | TEKT3 | tektin 3 | 64518 |
| 159 | -4.0483 | 0.204131 | 1.07E-04 | 0.005439 | 0.492987 | H200007392 | ARHGDIA | Rho GDP dissociation inhibitor (GDI) alpha | 396 |
| 4197 | -2.3849 | 0.585368 | 5.11E-03 | 0.024293 | 0.493117 | H200004042 | ARRDC2 | arrestin domain containing 2 | 27106 |
| 10939 | -3.9666 | 0.16582 | 1.22E-04 | 0.005642 | 0.493292 | H200020464 | NA | NA | - |
| 14254 | -3.7257 | 0.263919 | 1.92E-04 | 0.006226 | 0.493302 | H200004153 | PLEKHM1 | pleckstrin homology domain containing, family M (with RUN domain) member 1 | 9842 |
| 4834 | -2.2468 | 0.637474 | 7.58E-03 | 0.03034 | 0.493604 | H200012657 | MMRN2 | multimerin 2 | 79812 |
| 13195 | -2.0553 | 0.51583 | 1.31E-02 | 0.040613 | 0.493654 | H200019026 | DERL1 | derlin 1 | 79139 |
| 4083 | -2.5099 | 0.379307 | 3.61E-03 | 0.020512 | 0.493703 | H200020263 | ITSN2 | intersectin 2 | 50618 |
| 20635 | -1.7868 | 0.471025 | 2.82E-02 | 0.062862 | 0.493738 | H200012903 | SOHLH2 | spermatogenesis and oogenesis specific basic helix-loop-helix 2 | 54937 |
| 1182 | -2.9409 | 0.340547 | 1.15E-03 | 0.011848 | 0.493879 | H200012706 | FOXD2-AS1 | FOXD2 antisense RNA 1 (head to head) | 84793 |
| 19716 | -4.2749 | 0.205122 | 7.23E-05 | 0.004946 | 0.493943 | H200008586 | TNNC2 | troponin C type 2 (fast) | 7125 |
| 9879 | -3.6924 | 0.253745 | 2.04E-04 | 0.006332 | 0.494089 | H200013612 | BPIFA2 | BPI fold containing family A, member 2 | 140683 |
| 8399 | -3.183 | 0.319456 | 6.40E-04 | 0.009407 | 0.494477 | H200008162 | ADAM33 | ADAM metallopeptidase domain 33 | 80332 |
| 12098 | -3.1992 | 0.334487 | 6.17E-04 | 0.009361 | 0.494556 | H200010375 | TAF10 | TAF10 RNA polymerase II, TATA box binding protein (TBP)-associated factor, 30kDa | 6881 |
| 411 | -2.3179 | 0.419685 | 6.19E-03 | 0.026798 | 0.494579 | H200019504 | LOC400752 | uncharacterized LOC400752 | 400752 |
| 15476 | -1.9307 | 0.736452 | 1.86E-02 | 0.049597 | 0.494661 | H200018841 | NA | NA | - |
| 15306 | -4.0629 | 0.216515 | 1.04E-04 | 0.005439 | 0.49484 | H200010837 | TP53I13 | tumor protein p53 inducible protein 13 | 90313 |
| 10710 | -3.5834 | 0.225677 | 2.57E-04 | 0.00686 | 0.494862 | H200009474 | C17orf70 | chromosome 17 open reading frame 70 | 80233 |
| 9385 | -2.0843 | 0.543758 | 1.20E-02 | 0.038927 | 0.494965 | H200011783 | SHCBP1L | SHC SH2-domain binding protein 1-like | 81626 |
| 15168 | -2.5959 | 0.421533 | 2.86E-03 | 0.018254 | 0.495026 | H200004069 | PTPRE | protein tyrosine phosphatase, receptor type, E | 5791 |
| 14853 | -1.9006 | 0.823653 | 2.03E-02 | 0.052104 | 0.495182 | H200010784 | CHAD | chondroadherin | 1101 |
| 3657 | -2.4585 | 0.356876 | 4.17E-03 | 0.022089 | 0.495188 | H200000099 | NA | NA | - |
| 10079 | -4.205 | 0.183486 | 8.08E-05 | 0.005233 | 0.495287 | H200001179 | ILK | integrin-linked kinase | 3611 |
| 5896 | -2.2377 | 0.496784 | 7.78E-03 | 0.030671 | 0.495333 | H200019462 | NA | NA | - |
| 10196 | -2.9 | 0.234895 | 1.28E-03 | 0.012441 | 0.495489 | H200006553 | VEGFB | vascular endothelial growth factor B | 7423 |
| 4692 | -3.1074 | 0.131591 | 7.66E-04 | 0.009987 | 0.495744 | H200005841 | ONECUT1 | one cut homeobox 1 | 3175 |
| 18894 | -1.932 | 0.541841 | 1.86E-02 | 0.049545 | 0.496076 | H200011620 | MXD1 | MAX dimerization protein 1 | 4084 |
| 1 | -1.9424 | 0.518843 | 1.80E-02 | 0.048772 | 0.496088 | H200000100 | OPRD1 | opioid receptor, delta 1 | 4985 |
| 16554 | -2.766 | 0.457207 | 1.82E-03 | 0.014755 | 0.496193 | H200004947 | ANKRD44 | ankyrin repeat domain 44 | 91526 |
| 11604 | -2.2206 | 1.399446 | 8.17E-03 | 0.031419 | 0.496283 | H200008582 | CSF2RA | colony stimulating factor 2 receptor, alpha, low-affinity (granulocyte-macrophage) | 1438 |
| 16761 | -2.2444 | 0.494732 | 7.64E-03 | 0.030457 | 0.496448 | H200014821 | RPH3AL | rabphilin 3A-like (without C2 domains) | 9501 |
| 1317 | -3.6151 | 0.187841 | 2.40E-04 | 0.00669 | 0.496635 | H200019160 | SLC25A28 | solute carrier family 25 (mitochondrial iron transporter), member 28 | 81894 |
| 19624 | -4.01 | 0.373573 | 1.13E-04 | 0.005439 | 0.496675 | H200004074 | BCL3 | B-cell CLL/lymphoma 3 | 602 |
| 11044 | -2.1692 | 0.743099 | 9.45E-03 | 0.034192 | 0.496741 | H200003547 | CLDN3 | claudin 3 | 1365 |
| 14860 | -3.1402 | 0.364863 | 7.06E-04 | 0.009862 | 0.496748 | H200011146 | MR1 | major histocompatibility complex, class I-related | 3140 |
| 20767 | -3.093 | 0.287461 | 7.93E-04 | 0.010187 | 0.496789 | H200020117 | MTMR1 | myotubularin related protein 1 | 8776 |
| 9776 | -2.7421 | 0.344567 | 1.94E-03 | 0.015222 | 0.496874 | H200008678 | NA | NA | - |
| 7659 | -1.8391 | 0.603892 | 2.42E-02 | 0.057474 | 0.497064 | H200016462 | NA | NA | - |
| 15090 | -2.0671 | 0.504428 | 1.26E-02 | 0.039775 | 0.497389 | H200000577 | CD3G | CD3g molecule, gamma (CD3-TCR complex) | 917 |
| 5857 | -2.2078 | 0.450606 | 8.47E-03 | 0.032072 | 0.497407 | H200017864 | PCDHGB4 | protocadherin gamma subfamily B, 4 | 8641 |
| 7177 | -2.3369 | 0.439953 | 5.86E-03 | 0.026174 | 0.497473 | H200015501 | PAFAH2 | platelet-activating factor acetylhydrolase 2, 40kDa | 5051 |
| 3099 | -3.0389 | 0.328074 | 9.05E-04 | 0.010685 | 0.497475 | H200016809 | NA | NA | - |
| 19231 | -1.9345 | 1.288718 | 1.84E-02 | 0.049352 | 0.497501 | H200007001 | IL18 | interleukin 18 (interferon-gamma-inducing factor) | 3606 |
| 4188 | -2.1594 | 0.49318 | 9.73E-03 | 0.034686 | 0.497526 | H200003644 | MSRA | methionine sulfoxide reductase A | 4482 |
| 10265 | -2.5282 | 0.429027 | 3.44E-03 | 0.019974 | 0.497693 | H200009943 | MFAP5 | microfibrillar associated protein 5 | 8076 |
| 2819 | -2.4388 | 0.425259 | 4.40E-03 | 0.022431 | 0.497781 | H200003509 | MCRS1 | microspherule protein 1 | 10445 |
| 20748 | -2.6169 | 0.358355 | 2.71E-03 | 0.017721 | 0.497814 | H200019291 | GPRC5C | G protein-coupled receptor, family C, group 5, member C | 55890 |
| 11077 | -2.2416 | 0.474595 | 7.69E-03 | 0.030595 | 0.497862 | H200005085 | PPYR1 | pancreatic polypeptide receptor 1 | 5540 |
| 9655 | -1.9268 | 0.534973 | 1.89E-02 | 0.049907 | 0.497871 | H200002972 | NA | NA | - |
| 3225 | -2.595 | 0.431562 | 2.87E-03 | 0.01827 | 0.497948 | H200001442 | ATP6V0B | ATPase, H+ transporting, lysosomal 21kDa, V0 subunit b | 533 |
| 16519 | -2.1551 | 0.621575 | 9.85E-03 | 0.034916 | 0.498002 | H200003113 | CYB5R2 | cytochrome b5 reductase 2 | 51700 |
| 12348 | -3.5011 | 0.236892 | 3.11E-04 | 0.007429 | 0.498113 | H200000614 | SOD3 | superoxide dismutase 3, extracellular | 6649 |
| 19092 | -1.9325 | 1.663312 | 1.86E-02 | 0.049523 | 0.498174 | H200000511 | TBXAS1 | thromboxane A synthase 1 (platelet) | 6916 |
| 19654 | -4.3575 | 0.179291 | 6.26E-05 | 0.00476 | 0.498191 | H200005570 | 9-Mar | membrane-associated ring finger (C3HC4) 9 | 92979 |
| 9678 | -3.6193 | 0.239695 | 2.38E-04 | 0.006669 | 0.498464 | H200004094 | NA | NA | - |
| 13129 | -2.4729 | 0.426876 | 4.00E-03 | 0.021704 | 0.498591 | H200015962 | C6orf25 | chromosome 6 open reading frame 25 | 80739 |
| 7034 | -2.3739 | 0.227137 | 5.27E-03 | 0.02461 | 0.49892 | H200008667 | NDUFB1 | NADH dehydrogenase (ubiquinone) 1 beta subcomplex, 1, 7kDa | 4707 |
| 12769 | -2.3093 | 0.499383 | 6.34E-03 | 0.027123 | 0.498937 | H200020724 | NA | NA | - |
| 13282 | -2.8572 | 0.281998 | 1.44E-03 | 0.013089 | 0.499065 | H200001481 | HDHD3 | haloacid dehalogenase-like hydrolase domain containing 3 | 81932 |
| 11915 | -2.1219 | 0.372596 | 1.08E-02 | 0.036901 | 0.499116 | H200001653 | B3GNT1 | UDP-GlcNAc:betaGal beta-1,3-N-acetylglucosaminyltransferase 1 | 11041 |
| 17583 | -3.0068 | 0.375053 | 9.82E-04 | 0.011076 | 0.499184 | H200010279 | SUSD3 | sushi domain containing 3 | 203328 |
| 7016 | -2.4258 | 0.396602 | 4.56E-03 | 0.022845 | 0.499266 | H200007599 | NA | NA | - |
| 4098 | -2.0441 | 0.650458 | 1.35E-02 | 0.041453 | 0.499404 | H200021005 | NA | NA | - |
| 6992 | -2.5004 | 0.363178 | 3.71E-03 | 0.020795 | 0.49977 | H200006459 | ADORA1 | adenosine A1 receptor | 134 |
| 5658 | -2.4746 | 0.388011 | 3.98E-03 | 0.021629 | 0.499802 | H200008370 | MEP1A | meprin A, alpha (PABA peptide hydrolase) | 4224 |
| 15979 | -1.8254 | 0.394082 | 2.52E-02 | 0.05883 | 0.499831 | H200021032 | DENND1B | DENN/MADD domain containing 1B | 163486 |
| 12798 | -2.6903 | 0.534012 | 2.22E-03 | 0.016186 | 0.499858 | H200000056 | TSPO | translocator protein (18kDa) | 706 |
| 266 | -2.3496 | 0.674435 | 5.65E-03 | 0.02565 | 0.499901 | H200012646 | GPR155 | G protein-coupled receptor 155 | 151556 |
| 17640 | -3.5785 | 0.171164 | 2.59E-04 | 0.006883 | 0.50027 | H200012957 | TTC7A | tetratricopeptide repeat domain 7A | 57217 |
| 17965 | -2.4557 | 0.467612 | 4.20E-03 | 0.022146 | 0.50031 | H200006788 | CKMT2 | creatine kinase, mitochondrial 2 (sarcomeric) | 1160 |
| 7327 | -2.2538 | 0.424453 | 7.43E-03 | 0.029951 | 0.500492 | H200000550 | GDF10 | growth differentiation factor 10 | 2662 |
| 11639 | -3.7099 | 0.294203 | 1.98E-04 | 0.006274 | 0.500622 | H200010144 | C19orf60 | chromosome 19 open reading frame 60 | 55049 |
| 7430 | -3.4679 | 0.295832 | 3.35E-04 | 0.007529 | 0.500825 | H200005472 | SPRYD3 | SPRY domain containing 3 | 84926 |
| 9259 | -2.7145 | 0.260089 | 2.08E-03 | 0.015763 | 0.50116 | H200005727 | NA | NA | - |
| 20210 | -2.4402 | 0.386951 | 4.38E-03 | 0.022409 | 0.50125 | H200011478 | NA | NA | 202347 |
| 1289 | -1.8094 | 0.988742 | 2.64E-02 | 0.060425 | 0.501254 | H200017972 | SLC37A1 | solute carrier family 37 (glycerol-3-phosphate transporter), member 1 | 54020 |
| 18727 | -2.2199 | 0.440572 | 8.19E-03 | 0.031449 | 0.501505 | H200002518 | TRIM62 | tripartite motif containing 62 | 55223 |
| 1033 | -3.154 | 0.258071 | 6.85E-04 | 0.00973 | 0.501609 | H200005812 | RASSF7 | Ras association (RalGDS/AF-6) domain family (N-terminal) member 7 | 8045 |
| 17000 | -2.9667 | 0.313692 | 1.08E-03 | 0.011546 | 0.501629 | H200004490 | NA | NA | - |
| 12271 | -2.3775 | 0.478702 | 5.22E-03 | 0.02453 | 0.501672 | H200018421 | NA | NA | - |
| 6891 | -2.8175 | 0.212709 | 1.59E-03 | 0.013777 | 0.50212 | H200001845 | DCXR | dicarbonyl/L-xylulose reductase | 51181 |
| 710 | -2.2475 | 0.497056 | 7.57E-03 | 0.030316 | 0.502185 | H200012029 | PIN1P1 | peptidylprolyl cis/trans isomerase, NIMA-interacting 1 pseudogene 1 | 5301 |
| 11674 | -2.6352 | 0.826525 | 2.57E-03 | 0.01733 | 0.502279 | H200011978 | LAIR1 | leukocyte-associated immunoglobulin-like receptor 1 | 3903 |
| 11826 | -2.075 | 0.683944 | 1.24E-02 | 0.039424 | 0.502777 | H200019198 | NA | NA | - |
| 1912 | -2.8149 | 0.283199 | 1.60E-03 | 0.013842 | 0.502969 | H200003883 | TMEM80 | transmembrane protein 80 | 283232 |
| 11387 | -2.83 | 0.30785 | 1.54E-03 | 0.013569 | 0.502969 | H200019881 | VWA5B2 | von Willebrand factor A domain containing 5B2 | 90113 |
| 5901 | -2.6863 | 0.373473 | 2.25E-03 | 0.016217 | 0.503262 | H200019812 | NA | NA | - |
| 11538 | -1.8956 | 0.887762 | 2.06E-02 | 0.052372 | 0.503544 | H200005518 | TMPRSS4 | transmembrane protease, serine 4 | 56649 |
| 11086 | -3.6291 | 0.282734 | 2.34E-04 | 0.006651 | 0.50367 | H200005471 | RHOBTB2 | Rho-related BTB domain containing 2 | 23221 |
| 1129 | -2.2192 | 0.225553 | 8.20E-03 | 0.031476 | 0.50378 | H200010372 | EPHX1 | epoxide hydrolase 1, microsomal (xenobiotic) | 2052 |
| 2678 | -2.6151 | 0.455846 | 2.72E-03 | 0.017736 | 0.503889 | H200018916 | TMEM55B | transmembrane protein 55B | 90809 |
| 14189 | -2.9455 | 0.435479 | 1.14E-03 | 0.011769 | 0.50389 | H200001107 | C21orf59 | chromosome 21 open reading frame 59 | 56683 |
| 684 | -2.6281 | 0.416952 | 2.62E-03 | 0.017447 | 0.503917 | H200010865 | VRK3 | vaccinia related kinase 3 | 51231 |
| 19709 | -2.0085 | 0.595729 | 1.49E-02 | 0.043805 | 0.503944 | H200008212 | NA | NA | - |
| 5002 | -2.9334 | 0.222055 | 1.18E-03 | 0.012 | 0.503978 | H200020637 | NA | NA | - |
| 1424 | -2.3813 | 0.494984 | 5.16E-03 | 0.024397 | 0.503979 | H200002565 | SUOX | sulfite oxidase | 6821 |
| 7259 | -2.1154 | 0.411149 | 1.10E-02 | 0.037227 | 0.503986 | H200019325 | WDR25 | WD repeat domain 25 | 79446 |
| 10620 | -4.4051 | 0.189139 | 5.79E-05 | 0.00473 | 0.50411 | H200005270 | GIT2 | G protein-coupled receptor kinase interacting ArfGAP 2 | 9815 |
| 13290 | -3.3534 | 0.24792 | 4.33E-04 | 0.00823 | 0.504145 | H200001861 | CCM2 | cerebral cavernous malformation 2 | 83605 |
| 10326 | -3.6565 | 0.27079 | 2.20E-04 | 0.006519 | 0.504154 | H200012941 | TMEM174 | transmembrane protein 174 | 134288 |
| 5584 | -3.4329 | 0.564429 | 3.62E-04 | 0.007653 | 0.504203 | H200004642 | OLFML2B | olfactomedin-like 2B | 25903 |
| 13759 | -2.8227 | 0.309531 | 1.57E-03 | 0.01371 | 0.504263 | H200002318 | IFFO2 | intermediate filament family orphan 2 | 126917 |
| 17313 | -3.0215 | 0.284142 | 9.45E-04 | 0.010877 | 0.504328 | H200019316 | ANKRD34A | ankyrin repeat domain 34A | 284615 |
| 19032 | -4.168 | 0.203058 | 8.52E-05 | 0.005263 | 0.504599 | H200019196 | APOL5 | apolipoprotein L, 5 | 80831 |
| 1106 | -2.8413 | 0.410335 | 1.50E-03 | 0.013389 | 0.504658 | H200009238 | PQLC1 | PQ loop repeat containing 1 | 80148 |
| 543 | -3.0692 | 0.141702 | 8.40E-04 | 0.010389 | 0.504787 | H200004067 | SNTA1 | syntrophin, alpha 1 | 6640 |
| 2298 | -1.9288 | 0.414484 | 1.88E-02 | 0.049734 | 0.504868 | H200000724 | TNP1 | transition protein 1 (during histone to protamine replacement) | 7141 |
| 16101 | -3.6975 | 0.391207 | 2.03E-04 | 0.006332 | 0.504876 | H200005191 | DHX58 | DEXH (Asp-Glu-X-His) box polypeptide 58 | 79132 |
| 6348 | -2.6745 | 0.38302 | 2.32E-03 | 0.01651 | 0.504931 | H200019367 | NA | NA | - |
| 6561 | -2.8707 | 0.328943 | 1.39E-03 | 0.012895 | 0.504966 | H200007664 | LILRA1 | leukocyte immunoglobulin-like receptor, subfamily A (with TM domain), member 1 | 11024 |
| 4627 | -3.6888 | 0.242374 | 2.06E-04 | 0.006332 | 0.504972 | H200002795 | AP2A2 | adaptor-related protein complex 2, alpha 2 subunit | 161 |
| 18009 | -2.056 | 0.325665 | 1.31E-02 | 0.040584 | 0.504972 | H200008736 | FANCA | Fanconi anemia, complementation group A | 2175 |
| 11566 | -3.0773 | 0.400524 | 8.23E-04 | 0.010333 | 0.505197 | H200006706 | ZNF787 | zinc finger protein 787 | 126208 |
| 494 | -3.6963 | 0.23483 | 2.03E-04 | 0.006332 | 0.505266 | H200001769 | RGS14 | regulator of G-protein signaling 14 | 10636 |
| 14998 | -2.2833 | 0.726709 | 6.82E-03 | 0.028333 | 0.505279 | H200017630 | PLTP | phospholipid transfer protein | 5360 |
| 18058 | -2.0452 | 0.470787 | 1.35E-02 | 0.041342 | 0.50537 | H200011318 | AGRP | agouti related protein homolog (mouse) | 181 |
| 19014 | -2.2662 | 0.426423 | 7.17E-03 | 0.029318 | 0.505465 | H200018098 | NA | NA | - |
| 17962 | -1.9906 | 0.14496 | 1.57E-02 | 0.045068 | 0.50558 | H200006758 | MAL | mal, T-cell differentiation protein | 4118 |
| 4747 | -2.562 | 0.404803 | 3.14E-03 | 0.019068 | 0.505672 | H200008495 | STAT6 | signal transducer and activator of transcription 6, interleukin-4 induced | 6778 |
| 15626 | -3.0455 | 0.309439 | 8.91E-04 | 0.010642 | 0.505696 | H200004294 | STK4 | serine/threonine kinase 4 | 6789 |
| 9795 | -3.1334 | 0.477838 | 7.17E-04 | 0.009869 | 0.50582 | H200009480 | NECAP2 | NECAP endocytosis associated 2 | 55707 |
| 12063 | -2.7213 | 1.159245 | 2.05E-03 | 0.015632 | 0.505931 | H200008541 | HLA-E | major histocompatibility complex, class I, E | 3133 |
| 3496 | -1.7736 | 1.044785 | 2.92E-02 | 0.064194 | 0.506342 | H200014060 | PRKCD | protein kinase C, delta | 5580 |
| 1728 | -3.3409 | 0.134641 | 4.45E-04 | 0.008352 | 0.506423 | H200017005 | LRWD1 | leucine-rich repeats and WD repeat domain containing 1 | 222229 |
| 5975 | -2.5853 | 0.202702 | 2.95E-03 | 0.01846 | 0.506431 | H200001833 | SS18L2 | synovial sarcoma translocation gene on chromosome 18-like 2 | 51188 |
| 17501 | -2.7892 | 0.218141 | 1.71E-03 | 0.014261 | 0.506612 | H200006171 | EDC4 | enhancer of mRNA decapping 4 | 23644 |
| 21139 | -2.3262 | 0.422152 | 6.04E-03 | 0.026566 | 0.506629 | H200016026 | NA | NA | - |
| 7898 | -2.273 | 0.389213 | 7.03E-03 | 0.028877 | 0.507062 | H200006279 | GRK6 | G protein-coupled receptor kinase 6 | 2870 |
| 7059 | -1.9536 | 0.535898 | 1.75E-02 | 0.047926 | 0.507128 | H200009825 | DUSP7 | dual specificity phosphatase 7 | 1849 |
| 12269 | -2.3295 | 0.525357 | 5.99E-03 | 0.026392 | 0.507316 | H200018397 | NA | NA | - |
| 861 | -2.5933 | 0.397116 | 2.88E-03 | 0.018341 | 0.5074 | H200019243 | NA | NA | 85370 |
| 5692 | -2.7842 | 0.563639 | 1.73E-03 | 0.014343 | 0.507563 | H200009914 | ARHGAP31 | Rho GTPase activating protein 31 | 57514 |
| 18645 | -3.0431 | 0.242227 | 8.97E-04 | 0.01067 | 0.507576 | H200019553 | NA | NA | - |
| 21035 | -3.1381 | 0.321272 | 7.08E-04 | 0.009862 | 0.507591 | H200011086 | CEP72 | centrosomal protein 72kDa | 55722 |
| 15572 | -2.1378 | 0.426929 | 1.03E-02 | 0.0359 | 0.50768 | H200001658 | RAPGEF3 | Rap guanine nucleotide exchange factor (GEF) 3 | 10411 |
| 19585 | -3.0408 | 0.264074 | 9.01E-04 | 0.010685 | 0.507772 | H200002180 | ACOX3 | acyl-CoA oxidase 3, pristanoyl | 8310 |
| 10015 | -2.0557 | 0.564225 | 1.31E-02 | 0.040594 | 0.508152 | H200020072 | ZSCAN18 | zinc finger and SCAN domain containing 18 | 65982 |
| 20335 | -1.8502 | 0.562638 | 2.35E-02 | 0.056554 | 0.508273 | H200018312 | NA | NA | - |
| 5591 | -2.0017 | 0.42941 | 1.52E-02 | 0.044412 | 0.508317 | H200005016 | CDKL5 | cyclin-dependent kinase-like 5 | 6792 |
| 8136 | -3.2065 | 0.292359 | 6.06E-04 | 0.009348 | 0.508417 | H200017371 | NA | NA | - |
| 8439 | -1.7816 | 0.520634 | 2.86E-02 | 0.063323 | 0.508428 | H200010062 | SNRPN | small nuclear ribonucleoprotein polypeptide N | 6638 |
| 4722 | -2.416 | 0.426823 | 4.68E-03 | 0.023211 | 0.508564 | H200007337 | CEBPE | CCAAT/enhancer binding protein (C/EBP), epsilon | 1053 |
| 18226 | -2.9028 | 0.305705 | 1.27E-03 | 0.012373 | 0.508655 | H200019298 | PCGF1 | polycomb group ring finger 1 | 84759 |
| 14733 | -1.8848 | 0.603372 | 2.13E-02 | 0.053257 | 0.508831 | H200005084 | HNF4A | hepatocyte nuclear factor 4, alpha | 3172 |
| 10619 | -2.3626 | 0.398667 | 5.45E-03 | 0.0251 | 0.508987 | H200005264 | FGF8 | fibroblast growth factor 8 (androgen-induced) | 2253 |
| 19919 | -4.3423 | 0.178414 | 6.44E-05 | 0.004829 | 0.509017 | H200018116 | STX5 | syntaxin 5 | 6811 |
| 9679 | -2.4109 | 0.410478 | 4.75E-03 | 0.023357 | 0.509031 | H200004112 | CELF5 | CUGBP, Elav-like family member 5 | 60680 |
| 15429 | -3.4994 | 0.219034 | 3.12E-04 | 0.007429 | 0.509086 | H200016579 | NA | NA | - |
| 15116 | -2.642 | 0.386891 | 2.53E-03 | 0.017125 | 0.509115 | H200001741 | SLC13A4 | solute carrier family 13 (sodium/sulfate symporters), member 4 | 26266 |
| 10943 | -3.0968 | 0.340535 | 7.86E-04 | 0.010131 | 0.509182 | H200020512 | EAF1 | ELL associated factor 1 | 85403 |
| 15581 | -2.1182 | 0.411371 | 1.09E-02 | 0.037112 | 0.509309 | H200002056 | TAOK3 | TAO kinase 3 | 51347 |
| 16973 | -2.3533 | 0.512463 | 5.59E-03 | 0.025481 | 0.509384 | H200003024 | PLXNA3 | plexin A3 | 55558 |
| 17487 | -2.1456 | 0.515399 | 1.01E-02 | 0.03544 | 0.509565 | H200005719 | GABRP | gamma-aminobutyric acid (GABA) A receptor, pi | 2568 |
| 11673 | -2.8933 | 0.208431 | 1.30E-03 | 0.012515 | 0.509667 | H200011972 | HTRA2 | HtrA serine peptidase 2 | 27429 |
| 15469 | -3.9457 | 0.180771 | 1.27E-04 | 0.005667 | 0.510024 | H200018479 | NA | NA | - |
| 5503 | -4.4994 | 0.172673 | 4.93E-05 | 0.004597 | 0.510135 | H200000836 | LRP10 | low density lipoprotein receptor-related protein 10 | 26020 |
| 12976 | -2.5698 | 0.394637 | 3.07E-03 | 0.018851 | 0.510223 | H200008440 | MAP2K3 | mitogen-activated protein kinase kinase 3 | 5606 |
| 5202 | -2.9862 | 0.333017 | 1.03E-03 | 0.011308 | 0.510592 | H200008572 | NHP2L1 | NHP2 non-histone chromosome protein 2-like 1 (S. cerevisiae) | 4809 |
| 977 | -2.7713 | 0.326114 | 1.79E-03 | 0.01462 | 0.510708 | H200003152 | NA | NA | - |
| 17602 | -3.1949 | 0.220318 | 6.23E-04 | 0.009361 | 0.510725 | H200011081 | RAD51B | RAD51 homolog B (S. cerevisiae) | 5890 |
| 3456 | -2.2526 | 0.525014 | 7.46E-03 | 0.030017 | 0.510762 | H200012160 | MARK4 | MAP/microtubule affinity-regulating kinase 4 | 57787 |
| 1474 | -1.7779 | 0.202081 | 2.89E-02 | 0.063644 | 0.510799 | H200005153 | CCDC50 | coiled-coil domain containing 50 | 152137 |
| 15037 | -2.7806 | 0.383523 | 1.75E-03 | 0.014426 | 0.510885 | H200019524 | DNAJC17 | DnaJ (Hsp40) homolog, subfamily C, member 17 | 55192 |
| 2863 | -2.1664 | 0.420729 | 9.53E-03 | 0.034399 | 0.511056 | H200005741 | DUOX2 | dual oxidase 2 | 50506 |
| 4082 | -1.8591 | 0.905012 | 2.29E-02 | 0.055655 | 0.511088 | H200020245 | NA | NA | - |
| 91 | -2.5886 | 0.329435 | 2.92E-03 | 0.018409 | 0.511134 | H200004304 | FAM167A | family with sequence similarity 167, member A | 83648 |
| 6018 | -3.122 | 0.289616 | 7.39E-04 | 0.009945 | 0.511159 | H200003763 | FAM174B | family with sequence similarity 174, member B | 400451 |
| 15339 | -3.0767 | 0.257596 | 8.25E-04 | 0.010344 | 0.511177 | H200012375 | MZB1 | marginal zone B and B1 cell-specific protein | 51237 |
| 5626 | -2.9579 | 0.361458 | 1.10E-03 | 0.011668 | 0.511295 | H200006850 | UNC119 | unc-119 homolog (C. elegans) | 9094 |
| 1197 | -1.853 | 0.558141 | 2.33E-02 | 0.056237 | 0.511313 | H200013460 | NA | NA | - |
| 7739 | -2.8909 | 0.293236 | 1.31E-03 | 0.012515 | 0.511395 | H200020262 | NA | NA | - |
| 19354 | -3.1989 | 0.428732 | 6.17E-04 | 0.009361 | 0.511826 | H200013027 | 2-Mar | membrane-associated ring finger (C3HC4) 2, E3 ubiquitin protein ligase | 51257 |
| 2774 | -1.7835 | 0.619846 | 2.84E-02 | 0.063182 | 0.511853 | H200001543 | NA | NA | - |
| 16503 | -1.8753 | 0.525532 | 2.19E-02 | 0.054162 | 0.511858 | H200002353 | NUDT2 | nudix (nucleoside diphosphate linked moiety X)-type motif 2 | 318 |
| 5534 | -3.0305 | 0.296739 | 9.24E-04 | 0.01078 | 0.511879 | H200002338 | MPV17L2 | MPV17 mitochondrial membrane protein-like 2 | 84769 |
| 6520 | -2.8037 | 0.13674 | 1.65E-03 | 0.014047 | 0.511879 | H200005746 | IKBIP | IKBKB interacting protein | 121457 |
| 21410 | -3.0606 | 0.13206 | 8.58E-04 | 0.010432 | 0.511946 | H200007517 | DOLK | dolichol kinase | 22845 |
| 11774 | -1.9346 | 1.064951 | 1.84E-02 | 0.049352 | 0.511956 | H200016586 | POF1B | premature ovarian failure, 1B | 79983 |
| 20701 | -1.8923 | 0.579555 | 2.08E-02 | 0.052624 | 0.511969 | H200016655 | NA | NA | - |
| 12356 | -3.6665 | 0.238653 | 2.16E-04 | 0.006489 | 0.512053 | H200000994 | RBM42 | RNA binding motif protein 42 | 79171 |
| 21032 | -3.2388 | 0.354452 | 5.64E-04 | 0.009085 | 0.512234 | H200011056 | TSPAN3 | tetraspanin 3 | 10099 |
| 6124 | -2.8122 | 0.331197 | 1.61E-03 | 0.013872 | 0.51225 | H200008727 | DCAF11 | DDB1 and CUL4 associated factor 11 | 80344 |
| 6559 | -3.1151 | 0.216532 | 7.52E-04 | 0.009958 | 0.512253 | H200007640 | ZNF335 | zinc finger protein 335 | 63925 |
| 10 | -1.9413 | 0.509541 | 1.81E-02 | 0.048858 | 0.512271 | H200000486 | KLKB1 | kallikrein B, plasma (Fletcher factor) 1 | 3818 |
| 15458 | -2.9086 | 0.362317 | 1.25E-03 | 0.012277 | 0.512459 | H200018057 | DMBX1 | diencephalon/mesencephalon homeobox 1 | 127343 |
| 19063 | -2.6899 | 0.300544 | 2.23E-03 | 0.016186 | 0.512869 | H200020758 | TPRA1 | transmembrane protein, adipocyte asscociated 1 | 131601 |
| 15362 | -1.8926 | 0.577019 | 2.08E-02 | 0.052624 | 0.513046 | H200013497 | NA | NA | - |
| 9463 | -2.3239 | 0.422013 | 6.08E-03 | 0.02664 | 0.513342 | H200015275 | NA | NA | - |
| 20933 | -2.5893 | 0.233624 | 2.91E-03 | 0.0184 | 0.513393 | H200006170 | FOSB | FBJ murine osteosarcoma viral oncogene homolog B | 2354 |
| 8524 | -2.75 | 0.3838 | 1.90E-03 | 0.015096 | 0.513548 | H200014200 | NA | NA | - |
| 4776 | -2.8036 | 0.08172 | 1.65E-03 | 0.014047 | 0.513675 | H200009689 | PNMA3 | paraneoplastic Ma antigen 3 | 29944 |
| 13699 | -1.8396 | 0.719951 | 2.42E-02 | 0.057425 | 0.513823 | H200021259 | NA | NA | - |
| 15739 | -3.6925 | 0.208363 | 2.04E-04 | 0.006332 | 0.513871 | H200009632 | ZFAND2B | zinc finger, AN1-type domain 2B | 130617 |
| 6498 | -2.814 | 0.237347 | 1.61E-03 | 0.01385 | 0.513912 | H200004630 | DTNBP1 | dystrobrevin binding protein 1 | 84062 |
| 1187 | -2.5074 | 0.389344 | 3.64E-03 | 0.020563 | 0.51398 | H200013056 | USH1C | Usher syndrome 1C (autosomal recessive, severe) | 10083 |
| 9528 | -2.9446 | 0.196831 | 1.14E-03 | 0.011779 | 0.514028 | H200018321 | NA | NA | - |
| 9064 | -2.1031 | 0.183798 | 1.14E-02 | 0.037906 | 0.514121 | H200018143 | NA | NA | - |
| 17692 | -2.8733 | 0.388053 | 1.38E-03 | 0.012841 | 0.514253 | H200015285 | EIF1B-AS1 | EIF1B antisense RNA 1 | 440952 |
| 8237 | -2.2701 | 0.351605 | 7.09E-03 | 0.029051 | 0.514275 | H200000538 | DUSP5 | dual specificity phosphatase 5 | 1847 |
| 12709 | -3.8811 | 0.248554 | 1.42E-04 | 0.005857 | 0.514296 | H200017732 | KDM5C | lysine (K)-specific demethylase 5C | 8242 |
| 12134 | -2.3931 | 0.366863 | 4.99E-03 | 0.024024 | 0.514329 | H200011943 | SF3A2 | splicing factor 3a, subunit 2, 66kDa | 8175 |
| 1694 | -2.3384 | 0.395362 | 5.83E-03 | 0.026096 | 0.514337 | H200015461 | PCDHB10 | protocadherin beta 10 | 56126 |
| 2464 | -3.4821 | 0.294948 | 3.26E-04 | 0.007449 | 0.514363 | H200008680 | NA | NA | - |
| 14952 | -2.5517 | 0.388808 | 3.23E-03 | 0.019402 | 0.514455 | H200015374 | NA | NA | - |
| 1928 | -1.9442 | 0.427618 | 1.80E-02 | 0.048627 | 0.514594 | H200004643 | PTP4A3 | protein tyrosine phosphatase type IVA, member 3 | 11156 |
| 10972 | -2.7955 | 0.281386 | 1.68E-03 | 0.014216 | 0.514752 | H200000127 | HSD17B3 | hydroxysteroid (17-beta) dehydrogenase 3 | 3293 |
| 1665 | -1.9945 | 0.362132 | 1.56E-02 | 0.044842 | 0.514809 | H200014267 | SLC9A3R1 | solute carrier family 9, subfamily A (NHE3, cation proton antiporter 3), member 3 regulator 1 | 9368 |
| 1812 | -2.1754 | 0.44035 | 9.28E-03 | 0.033852 | 0.515008 | H200021137 | THOC1 | THO complex 1 | 9984 |
| 7435 | -2.7999 | 0.240645 | 1.66E-03 | 0.014104 | 0.515187 | H200005822 | STAT2 | signal transducer and activator of transcription 2, 113kDa | 6773 |
| 14587 | -3.5117 | 0.127573 | 3.04E-04 | 0.007429 | 0.51535 | H200020083 | KCNC2 | potassium voltage-gated channel, Shaw-related subfamily, member 2 | 3747 |
| 16718 | -2.886 | 0.250722 | 1.33E-03 | 0.012548 | 0.515562 | H200012595 | FRAT1 | frequently rearranged in advanced T-cell lymphomas | 10023 |
| 14850 | -2.785 | 0.210462 | 1.73E-03 | 0.014333 | 0.51558 | H200010742 | FCHO1 | FCH domain only 1 | 23149 |
| 9227 | -2.053 | 0.536673 | 1.32E-02 | 0.040744 | 0.515723 | H200004207 | SEMA3F | sema domain, immunoglobulin domain (Ig), short basic domain, secreted, (semaphorin) 3F | 6405 |
| 12072 | -1.7975 | 0.652402 | 2.73E-02 | 0.06174 | 0.515757 | H200008927 | NA | NA | - |
| 13767 | -2.1076 | 0.424785 | 1.13E-02 | 0.037653 | 0.516042 | H200002698 | ADPRHL2 | ADP-ribosylhydrolase like 2 | 54936 |
| 1981 | -2.7728 | 0.482038 | 1.78E-03 | 0.014597 | 0.516086 | H200007273 | PWWP2B | PWWP domain containing 2B | 170394 |
| 5963 | -2.8384 | 0.304248 | 1.51E-03 | 0.013422 | 0.516247 | H200001121 | FOSL2 | FOS-like antigen 2 | 2355 |
| 20106 | -1.9525 | 0.437847 | 1.75E-02 | 0.048019 | 0.516253 | H200006086 | THOC5 | THO complex 5 | 8563 |
| 4461 | -2.868 | 0.219809 | 1.40E-03 | 0.012925 | 0.516258 | H200016582 | TRNAU1AP | tRNA selenocysteine 1 associated protein 1 | 54952 |
| 16452 | -2.1346 | 0.408907 | 1.04E-02 | 0.036093 | 0.516524 | H200000031 | GRIN1 | glutamate receptor, ionotropic, N-methyl D-aspartate 1 | 2902 |
| 3669 | -2.9571 | 0.236063 | 1.11E-03 | 0.011678 | 0.516688 | H200000527 | UBA1 | ubiquitin-like modifier activating enzyme 1 | 7317 |
| 17276 | -2.5764 | 0.43152 | 3.02E-03 | 0.018624 | 0.51674 | H200017458 | MSRB1 | methionine sulfoxide reductase B1 | 51734 |
| 13078 | -1.8893 | 0.513772 | 2.10E-02 | 0.052923 | 0.516797 | H200013356 | NA | NA | - |
| 1313 | -1.8473 | 0.440643 | 2.37E-02 | 0.056758 | 0.516798 | H200019112 | ZNF747 | zinc finger protein 747 | 65988 |
| 9839 | -3.0525 | 0.26236 | 8.74E-04 | 0.010542 | 0.517086 | H200011712 | RRP1 | ribosomal RNA processing 1 homolog (S. cerevisiae) | 8568 |
| 10369 | -4.0241 | 0.171457 | 1.11E-04 | 0.005439 | 0.517132 | H200014883 | NA | NA | - |
| 7365 | -2.5809 | 0.240855 | 2.98E-03 | 0.01854 | 0.517384 | H200002426 | THAP7 | THAP domain containing 7 | 80764 |
| 19176 | -2.0871 | 0.412041 | 1.19E-02 | 0.038739 | 0.517435 | H200004359 | CA7 | carbonic anhydrase VII | 766 |
| 19233 | -2.7857 | 0.2275 | 1.73E-03 | 0.01433 | 0.517492 | H200007025 | F8A1 | coagulation factor VIII-associated 1 | 8263 |
| 16187 | -2.1226 | 0.393227 | 1.08E-02 | 0.036881 | 0.517503 | H200009347 | NA | NA | - |
| 6608 | -3.8898 | 0.190859 | 1.40E-04 | 0.005831 | 0.517579 | H200009926 | RAB1B | RAB1B, member RAS oncogene family | 81876 |
| 19840 | -2.3645 | 0.472011 | 5.42E-03 | 0.025012 | 0.517746 | H200014334 | NA | NA | 116829 |
| 6600 | -2.3893 | 0.337397 | 5.04E-03 | 0.024137 | 0.517785 | H200009546 | NA | NA | - |
| 14080 | -2.7549 | 0.225809 | 1.87E-03 | 0.014972 | 0.517908 | H200017524 | MAN1B1 | mannosidase, alpha, class 1B, member 1 | 11253 |
| 10874 | -2.2163 | 0.441445 | 8.27E-03 | 0.031611 | 0.518065 | H200017406 | CLDN18 | claudin 18 | 51208 |
| 4891 | -2.3697 | 0.392503 | 5.34E-03 | 0.024799 | 0.518098 | H200015335 | SSTR4 | somatostatin receptor 4 | 6754 |
| 946 | -2.8553 | 0.288508 | 1.45E-03 | 0.013122 | 0.518117 | H200001638 | SYBU | syntabulin (syntaxin-interacting) | 55638 |
| 10976 | -2.2757 | 0.450961 | 6.97E-03 | 0.028716 | 0.518214 | H200000175 | KRT2 | keratin 2 | 3849 |
| 7334 | -2.7348 | 0.374852 | 1.98E-03 | 0.015394 | 0.518626 | H200000912 | GPAA1 | glycosylphosphatidylinositol anchor attachment 1 | 8733 |
| 5819 | -2.2707 | 0.518526 | 7.08E-03 | 0.029019 | 0.51863 | H200015988 | NA | NA | - |
| 12988 | -1.8536 | 0.525966 | 2.33E-02 | 0.056163 | 0.518656 | H200009152 | NA | NA | 440863 |
| 17250 | -2.4369 | 0.365581 | 4.42E-03 | 0.022459 | 0.51869 | H200016294 | GIPR | gastric inhibitory polypeptide receptor | 2696 |
| 11599 | -3.1956 | 0.152172 | 6.21E-04 | 0.009361 | 0.518866 | H200008244 | DCAF7 | DDB1 and CUL4 associated factor 7 | 10238 |
| 16466 | -2.6133 | 0.464625 | 2.73E-03 | 0.017762 | 0.518873 | H200000767 | HSPA6 | heat shock 70kDa protein 6 (HSP70B') | 3310 |
| 18703 | -2.9224 | 0.454438 | 1.21E-03 | 0.012146 | 0.518908 | H200001336 | NUDT16L1 | nudix (nucleoside diphosphate linked moiety X)-type motif 16-like 1 | 84309 |
| 4622 | -2.976 | 0.313098 | 1.06E-03 | 0.011443 | 0.51891 | H200002445 | RHOBTB1 | Rho-related BTB domain containing 1 | 9886 |
| 6593 | -1.8994 | 0.792614 | 2.04E-02 | 0.052196 | 0.519023 | H200009184 | NA | NA | - |
| 5824 | -1.9649 | 0.400946 | 1.69E-02 | 0.047086 | 0.51914 | H200016042 | NA | NA | - |
| 13260 | -2.4887 | 0.388832 | 3.83E-03 | 0.021104 | 0.51917 | H200000365 | EDN2 | endothelin 2 | 1907 |
| 8735 | -3.6909 | 0.182764 | 2.05E-04 | 0.006332 | 0.519244 | H200002557 | RAB24 | RAB24, member RAS oncogene family | 53917 |
| 9542 | -1.9805 | 0.220567 | 1.62E-02 | 0.045841 | 0.519455 | H200019057 | WDR54 | WD repeat domain 54 | 84058 |
| 10644 | -2.1706 | 0.461185 | 9.41E-03 | 0.034109 | 0.519684 | H200006410 | IVD | isovaleryl-CoA dehydrogenase | 3712 |
| 11819 | -1.9268 | 0.508936 | 1.89E-02 | 0.049907 | 0.519689 | H200018836 | NA | NA | - |
| 12323 | -2.694 | 0.291888 | 2.20E-03 | 0.016167 | 0.519724 | H200021033 | KLHDC4 | kelch domain containing 4 | 54758 |
| 12486 | -3.203 | 0.277476 | 6.11E-04 | 0.009361 | 0.519802 | H200007098 | DPH1 | DPH1 homolog (S. cerevisiae) | 1801 |
| 11184 | -2.6351 | 0.479833 | 2.57E-03 | 0.01733 | 0.520071 | H200010055 | ZMYM6 | zinc finger, MYM-type 6 | 9204 |
| 11318 | -2.4254 | 0.411813 | 4.56E-03 | 0.02286 | 0.520107 | H200016491 | ACTL6B | actin-like 6B | 51412 |
| 21036 | -5.8241 | 0.159039 | 6.18E-06 | 0.003715 | 0.520347 | H200011104 | NA | NA | - |
| 12292 | -2.1897 | 0.38743 | 8.91E-03 | 0.033006 | 0.520397 | H200019519 | DCAF4 | DDB1 and CUL4 associated factor 4 | 26094 |
| 20191 | -2.0895 | 0.454399 | 1.19E-02 | 0.038603 | 0.5208 | H200010664 | NA | NA | - |
| 7861 | -2.0971 | 0.27259 | 1.16E-02 | 0.038206 | 0.520893 | H200004421 | ANAPC15 | anaphase promoting complex subunit 15 | 25906 |
| 8005 | -1.977 | 0.426243 | 1.64E-02 | 0.046087 | 0.521304 | H200011261 | SHISA3 | shisa homolog 3 (Xenopus laevis) | 152573 |
| 3275 | -3.6469 | 0.201081 | 2.25E-04 | 0.006587 | 0.521371 | H200003746 | NA | NA | 147341 |
| 491 | -2.5713 | 0.29592 | 3.06E-03 | 0.018808 | 0.52156 | H200001739 | POLR3GL | polymerase (RNA) III (DNA directed) polypeptide G (32kD)-like | 84265 |
| 20281 | -2.4775 | 0.469143 | 3.95E-03 | 0.021506 | 0.521581 | H200015586 | PPP2R4 | protein phosphatase 2A activator, regulatory subunit 4 | 5524 |
| 10871 | -2.2194 | 0.364536 | 8.20E-03 | 0.031468 | 0.521766 | H200017092 | RALGPS2 | Ral GEF with PH domain and SH3 binding motif 2 | 55103 |
| 10897 | -2.3835 | 0.383895 | 5.13E-03 | 0.024315 | 0.521767 | H200018540 | NA | NA | - |
| 8087 | -2.5871 | 0.28239 | 2.93E-03 | 0.018436 | 0.521806 | H200015085 | HIPK4 | homeodomain interacting protein kinase 4 | 147746 |
| 14035 | -2.9492 | 0.249049 | 1.13E-03 | 0.011758 | 0.521841 | H200015286 | DHDDS | dehydrodolichyl diphosphate synthase | 79947 |
| 20337 | -3.3925 | 0.275963 | 3.95E-04 | 0.007814 | 0.522003 | H200018626 | NA | NA | - |
| 9221 | -2.5548 | 0.296243 | 3.20E-03 | 0.019313 | 0.522101 | H200003851 | SUSD1 | sushi domain containing 1 | 64420 |
| 10632 | -1.9782 | 0.564982 | 1.63E-02 | 0.045979 | 0.522106 | H200005698 | CACNA1B | calcium channel, voltage-dependent, N type, alpha 1B subunit | 774 |
| 19504 | -2.0551 | 0.65773 | 1.31E-02 | 0.040613 | 0.522207 | H200019939 | KIF1A | kinesin family member 1A | 547 |
| 4298 | -2.7357 | 0.453456 | 1.97E-03 | 0.015378 | 0.522311 | H200008940 | NA | NA | - |
| 8743 | -2.7257 | 0.291387 | 2.03E-03 | 0.0156 | 0.522536 | H200002937 | ZNF691 | zinc finger protein 691 | 51058 |
| 1879 | -2.4924 | 0.278317 | 3.79E-03 | 0.021017 | 0.522549 | H200002357 | ZNF541 | zinc finger protein 541 | 84215 |
| 19082 | -1.9864 | 0.629877 | 1.59E-02 | 0.045393 | 0.522644 | H200000107 | NA | NA | 1231 |
| 11161 | -3.4866 | 0.268373 | 3.22E-04 | 0.007429 | 0.522768 | H200009217 | NA | NA | - |
| 566 | -1.8058 | 0.449747 | 2.67E-02 | 0.060851 | 0.522784 | H200005189 | ATF7 | activating transcription factor 7 | 11016 |
| 209 | -1.7852 | 0.507937 | 2.83E-02 | 0.063001 | 0.522792 | H200009980 | NA | NA | - |
| 8631 | -2.791 | 0.239946 | 1.70E-03 | 0.014256 | 0.522801 | H200019182 | WDR55 | WD repeat domain 55 | 54853 |
| 4157 | -3.2035 | 0.336858 | 6.10E-04 | 0.009361 | 0.522936 | H200002142 | LGI2 | leucine-rich repeat LGI family, member 2 | 55203 |
| 4391 | -3.1866 | 0.266103 | 6.34E-04 | 0.009405 | 0.523023 | H200013186 | ASCL2 | achaete-scute complex homolog 2 (Drosophila) | 430 |
| 10726 | -2.1493 | 0.461917 | 1.00E-02 | 0.035239 | 0.523043 | H200010234 | MAN2C1 | mannosidase, alpha, class 2C, member 1 | 4123 |
| 1885 | -2.519 | 0.124923 | 3.53E-03 | 0.020279 | 0.523168 | H200002713 | LAGE3 | L antigen family, member 3 | 8270 |
| 14409 | -2.9946 | 0.255647 | 1.01E-03 | 0.011191 | 0.523708 | H200011699 | GADD45B | growth arrest and DNA-damage-inducible, beta | 4616 |
| 1714 | -3.5589 | 0.2333 | 2.71E-04 | 0.007042 | 0.523853 | H200016553 | L3MBTL2 | l(3)mbt-like 2 (Drosophila) | 83746 |
| 21025 | -1.9466 | 0.499992 | 1.78E-02 | 0.04839 | 0.523974 | H200010682 | PLCD3 | phospholipase C, delta 3 | 113026 |
| 11849 | -2.7326 | 0.306913 | 1.99E-03 | 0.015417 | 0.524063 | H200020332 | SGCZ | sarcoglycan, zeta | 137868 |
| 4496 | -3.136 | 0.264232 | 7.12E-04 | 0.009862 | 0.524134 | H200018132 | LOC158376 | uncharacterized LOC158376 | 158376 |
| 12392 | -3.4447 | 0.21373 | 3.54E-04 | 0.007621 | 0.524276 | H200002562 | GLIS2 | GLIS family zinc finger 2 | 84662 |
| 3769 | -2.353 | 0.303106 | 5.59E-03 | 0.025481 | 0.524278 | H200005419 | ASL | argininosuccinate lyase | 435 |
| 10238 | -3.4787 | 0.27643 | 3.28E-04 | 0.007457 | 0.524381 | H200008761 | FBXO6 | F-box protein 6 | 26270 |
| 10121 | -2.2949 | 0.555963 | 6.60E-03 | 0.027804 | 0.524508 | H200003103 | PINLYP | phospholipase A2 inhibitor and LY6/PLAUR domain containing | 390940 |
| 9114 | -2.3554 | 0.441574 | 5.56E-03 | 0.025425 | 0.524606 | H200020731 | NXF3 | nuclear RNA export factor 3 | 56000 |
| 15583 | -1.8903 | 0.804218 | 2.10E-02 | 0.052806 | 0.524727 | H200002080 | SNX8 | sorting nexin 8 | 29886 |
| 3432 | -2.5047 | 0.225766 | 3.67E-03 | 0.020675 | 0.524753 | H200011020 | DRD4 | dopamine receptor D4 | 1815 |
| 11959 | -1.9216 | 0.459803 | 1.91E-02 | 0.050277 | 0.524906 | H200003601 | LRRC16B | leucine rich repeat containing 16B | 90668 |
| 1492 | -2.6278 | 0.381174 | 2.63E-03 | 0.017453 | 0.525102 | H200005937 | CLPP | ClpP caseinolytic peptidase, ATP-dependent, proteolytic subunit homolog (E. coli) | 8192 |
| 11089 | -2.3549 | 0.348169 | 5.57E-03 | 0.025432 | 0.52522 | H200005797 | JMJD6 | jumonji domain containing 6 | 23210 |
| 10640 | -1.9923 | 0.132811 | 1.57E-02 | 0.044979 | 0.52524 | H200006078 | INPPL1 | inositol polyphosphate phosphatase-like 1 | 3636 |
| 14800 | -2.3166 | 0.503161 | 6.22E-03 | 0.026846 | 0.525294 | H200008154 | SERPINF1 | serpin peptidase inhibitor, clade F (alpha-2 antiplasmin, pigment epithelium derived factor), member 1 | 5176 |
| 21215 | -2.9124 | 0.165423 | 1.24E-03 | 0.01225 | 0.525311 | H200019778 | TUBGCP6 | tubulin, gamma complex associated protein 6 | 85378 |
| 2558 | -3.2667 | 0.227552 | 5.28E-04 | 0.008857 | 0.525378 | H200013216 | SLC23A1 | solute carrier family 23 (nucleobase transporters), member 1 | 9963 |
| 7720 | -3.1927 | 0.397415 | 6.26E-04 | 0.009378 | 0.525428 | H200019176 | ELOF1 | elongation factor 1 homolog (S. cerevisiae) | 84337 |
| 2129 | -1.8846 | 0.402708 | 2.13E-02 | 0.053257 | 0.525441 | H200014445 | ZNF608 | zinc finger protein 608 | 57507 |
| 16511 | -2.8668 | 0.287788 | 1.40E-03 | 0.012949 | 0.525471 | H200002733 | DNAJC30 | DnaJ (Hsp40) homolog, subfamily C, member 30 | 84277 |
| 12959 | -3.83 | 0.167773 | 1.56E-04 | 0.005935 | 0.525604 | H200007674 | PHF1 | PHD finger protein 1 | 5252 |
| 13967 | -3.4001 | 0.283805 | 3.88E-04 | 0.00776 | 0.525608 | H200012198 | RASA3 | RAS p21 protein activator 3 | 22821 |
| 6023 | -1.9456 | 0.540183 | 1.79E-02 | 0.048469 | 0.525691 | H200004113 | MDP1 | magnesium-dependent phosphatase 1 | 145553 |
| 4809 | -2.2522 | 0.189751 | 7.46E-03 | 0.030037 | 0.525715 | H200011511 | CAMTA2 | calmodulin binding transcription activator 2 | 23125 |
| 16242 | -2.4813 | 0.325722 | 3.91E-03 | 0.021385 | 0.525764 | H200011989 | NA | NA | - |
| 8198 | -2.1709 | 0.479717 | 9.40E-03 | 0.034092 | 0.526177 | H200020387 | NA | NA | - |
| 11171 | -2.617 | 0.202567 | 2.70E-03 | 0.017721 | 0.526202 | H200009621 | G6PC3 | glucose 6 phosphatase, catalytic, 3 | 92579 |
| 976 | -1.9236 | 0.572926 | 1.90E-02 | 0.050119 | 0.526298 | H200002850 | ZSWIM3 | zinc finger, SWIM-type containing 3 | 140831 |
| 16944 | -2.3387 | 0.367214 | 5.83E-03 | 0.026096 | 0.526344 | H200001830 | DNAH1 | dynein, axonemal, heavy chain 1 | 25981 |
| 17223 | -3.0876 | 0.273169 | 8.04E-04 | 0.010245 | 0.526359 | H200015112 | HSPG2 | heparan sulfate proteoglycan 2 | 3339 |
| 3096 | -1.8972 | 0.401155 | 2.05E-02 | 0.052283 | 0.526375 | H200016767 | NA | NA | - |
| 3483 | -2.2971 | 0.414728 | 6.56E-03 | 0.027677 | 0.526458 | H200013626 | CDC42EP1 | CDC42 effector protein (Rho GTPase binding) 1 | 11135 |
| 19550 | -2.9898 | 0.260679 | 1.02E-03 | 0.011259 | 0.52649 | H200000630 | PKN1 | protein kinase N1 | 5585 |
| 2349 | -3.4866 | 0.193646 | 3.22E-04 | 0.007429 | 0.526517 | H200003330 | CACNA1D | calcium channel, voltage-dependent, L type, alpha 1D subunit | 776 |
| 21613 | -1.9026 | 0.41105 | 2.02E-02 | 0.05191 | 0.526571 | H200017047 | MCM3AP-AS1 | MCM3AP antisense RNA 1 | 114044 |
| 11292 | -2.7788 | 0.251059 | 1.76E-03 | 0.014473 | 0.526651 | H200015327 | NEUROG3 | neurogenin 3 | 50674 |
| 10300 | -3.0317 | 0.404262 | 9.22E-04 | 0.010768 | 0.526699 | H200011493 | PLVAP | plasmalemma vesicle associated protein | 83483 |
| 7736 | -2.1588 | 0.361599 | 9.74E-03 | 0.03471 | 0.526705 | H200019936 | SIK3 | SIK family kinase 3 | 23387 |
| 12183 | -2.9734 | 0.303166 | 1.06E-03 | 0.011445 | 0.526753 | H200014241 | PPP1CA | protein phosphatase 1, catalytic subunit, alpha isozyme | 5499 |
| 5880 | -2.1314 | 0.35903 | 1.05E-02 | 0.036318 | 0.526812 | H200018702 | NA | NA | - |
| 17794 | -2.7025 | 0.325383 | 2.15E-03 | 0.015982 | 0.52687 | H200020201 | PNMA5 | paraneoplastic Ma antigen family member 5 | 114824 |
| 6710 | -1.9084 | 0.663928 | 1.99E-02 | 0.051375 | 0.527053 | H200014842 | NA | NA | - |
| 1242 | -2.682 | 0.421056 | 2.27E-03 | 0.016293 | 0.527156 | H200015698 | PCDHA7 | protocadherin alpha 7 | 56141 |
| 6686 | -3.2734 | 0.177381 | 5.19E-04 | 0.008769 | 0.527292 | H200013702 | CDK9 | cyclin-dependent kinase 9 | 1025 |
| 7535 | -4.0685 | 0.106332 | 1.03E-04 | 0.005431 | 0.527367 | H200010430 | RNF220 | ring finger protein 220 | 55182 |
| 13456 | -2.6032 | 0.372267 | 2.81E-03 | 0.01805 | 0.527387 | H200009817 | RFXANK | regulatory factor X-associated ankyrin-containing protein | 8625 |
| 14886 | -2.3126 | 0.426705 | 6.29E-03 | 0.027011 | 0.527498 | H200012310 | CST5 | cystatin D | 1473 |
| 1687 | -2.4743 | 0.250517 | 3.99E-03 | 0.021637 | 0.527548 | H200015099 | RNF183 | ring finger protein 183 | 138065 |
| 3661 | -1.9104 | 0.922665 | 1.98E-02 | 0.051225 | 0.52767 | H200000147 | FUCA1 | fucosidase, alpha-L- 1, tissue | 2517 |
| 6492 | -1.9092 | 0.432449 | 1.98E-02 | 0.051332 | 0.527688 | H200004274 | DCX | doublecortin | 1641 |
| 13845 | -3.1508 | 0.278613 | 6.90E-04 | 0.009761 | 0.527691 | H200006474 | PNKP | polynucleotide kinase 3'-phosphatase | 11284 |
| 20427 | -2.9803 | 0.299475 | 1.05E-03 | 0.011443 | 0.527743 | H200001829 | R3HDM4 | R3H domain containing 4 | 91300 |
| 5532 | -3.3296 | 0.193521 | 4.56E-04 | 0.008442 | 0.528033 | H200002314 | TMEM109 | transmembrane protein 109 | 79073 |
| 19828 | -2.3076 | 0.191245 | 6.37E-03 | 0.027203 | 0.5282 | H200013906 | RRP9 | ribosomal RNA processing 9, small subunit (SSU) processome component, homolog (yeast) | 9136 |
| 19636 | -2.4742 | 0.14384 | 3.99E-03 | 0.021637 | 0.529142 | H200004786 | IFFO1 | intermediate filament family orphan 1 | 25900 |
| 19604 | -3.9348 | 0.201812 | 1.30E-04 | 0.005699 | 0.529252 | H200003266 | TMEM87B | transmembrane protein 87B | 84910 |
| 4807 | -2.9944 | 0.29609 | 1.01E-03 | 0.011191 | 0.529293 | H200011203 | PNLIP | pancreatic lipase | 5406 |
| 16411 | -3.81 | 0.169972 | 1.63E-04 | 0.005936 | 0.529668 | H200019987 | NA | NA | - |
| 13784 | -2.2619 | 0.387682 | 7.27E-03 | 0.029621 | 0.52981 | H200003464 | RAB39B | RAB39B, member RAS oncogene family | 116442 |
| 14827 | -2.5009 | 0.216832 | 3.70E-03 | 0.020795 | 0.529845 | H200009620 | MTHFR | methylenetetrahydrofolate reductase (NAD(P)H) | 4524 |
| 11026 | -2.9456 | 0.299904 | 1.14E-03 | 0.011769 | 0.530065 | H200002763 | TESC | tescalcin | 54997 |
| 16719 | -3.1095 | 0.264492 | 7.63E-04 | 0.00998 | 0.530091 | H200012613 | ZNRF4 | zinc and ring finger 4 | 148066 |
| 20046 | -2.0248 | 0.596088 | 1.43E-02 | 0.042773 | 0.530118 | H200002708 | LAD1 | ladinin 1 | 3898 |
| 12010 | -2.9156 | 0.655112 | 1.23E-03 | 0.012216 | 0.530143 | H200006195 | TAGLN | transgelin | 6876 |
| 9339 | -2.9731 | 0.155029 | 1.06E-03 | 0.011445 | 0.530239 | H200009527 | SLC50A1 | solute carrier family 50 (sugar transporter), member 1 | 55974 |
| 13179 | -2.4429 | 0.436809 | 4.35E-03 | 0.022348 | 0.530252 | H200018266 | OPLAH | 5-oxoprolinase (ATP-hydrolysing) | 26873 |
| 7002 | -3.4359 | 0.170288 | 3.60E-04 | 0.007649 | 0.530316 | H200007147 | HSD17B1 | hydroxysteroid (17-beta) dehydrogenase 1 | 3292 |
| 205 | -3.3631 | 0.380787 | 4.24E-04 | 0.008117 | 0.530564 | H200009648 | LYRM9 | LYR motif containing 9 | 201229 |
| 14599 | -1.7986 | 0.542435 | 2.72E-02 | 0.061643 | 0.530648 | H200020511 | NA | NA | - |
| 11069 | -2.5096 | 0.305754 | 3.62E-03 | 0.020512 | 0.530796 | H200004705 | CCDC28A | coiled-coil domain containing 28A | 25901 |
| 14226 | -2.5504 | 0.173486 | 3.24E-03 | 0.019442 | 0.531137 | H200002965 | ATG16L2 | autophagy related 16-like 2 (S. cerevisiae) | 89849 |
| 16780 | -3.0445 | 0.259627 | 8.93E-04 | 0.010656 | 0.531246 | H200015611 | SCAMP2 | secretory carrier membrane protein 2 | 10066 |
| 13581 | -2.7448 | 0.191141 | 1.92E-03 | 0.015142 | 0.531357 | H200015867 | ZFYVE28 | zinc finger, FYVE domain containing 28 | 57732 |
| 12186 | -1.9801 | 0.301836 | 1.62E-02 | 0.045841 | 0.531363 | H200014555 | CPLX2 | complexin 2 | 10814 |
| 20132 | -2.7605 | 0.323923 | 1.84E-03 | 0.014844 | 0.531426 | H200007292 | FAT2 | FAT tumor suppressor homolog 2 (Drosophila) | 2196 |
| 7040 | -2.1738 | 0.427976 | 9.32E-03 | 0.033932 | 0.531471 | H200008739 | RSL24D1 | ribosomal L24 domain containing 1 | 51187 |
| 10325 | -3.862 | 0.407099 | 1.47E-04 | 0.005878 | 0.531512 | H200012935 | DYRK1B | dual-specificity tyrosine-(Y)-phosphorylation regulated kinase 1B | 9149 |
| 15764 | -3.0722 | 0.322961 | 8.33E-04 | 0.010376 | 0.531639 | H200010778 | RNF25 | ring finger protein 25 | 64320 |
| 17240 | -2.9137 | 0.308051 | 1.23E-03 | 0.012235 | 0.531641 | H200015890 | NA | NA | - |
| 17132 | -1.998 | 0.572095 | 1.54E-02 | 0.04472 | 0.531902 | H200010618 | MPZ | myelin protein zero | 4359 |
| 18593 | -3.1229 | 0.282899 | 7.37E-04 | 0.009945 | 0.532001 | H200016863 | NA | NA | - |
| 21022 | -2.1744 | 0.370029 | 9.31E-03 | 0.033887 | 0.532204 | H200010652 | SULT2B1 | sulfotransferase family, cytosolic, 2B, member 1 | 6820 |
| 4529 | -2.1333 | 0.452816 | 1.05E-02 | 0.036173 | 0.532447 | H200019954 | ST3GAL2 | ST3 beta-galactoside alpha-2,3-sialyltransferase 2 | 6483 |
| 15496 | -4.4633 | 0.166799 | 5.20E-05 | 0.004709 | 0.532854 | H200019649 | SF3A1 | splicing factor 3a, subunit 1, 120kDa | 10291 |
| 6352 | -2.07 | 0.443571 | 1.25E-02 | 0.039613 | 0.532903 | H200019699 | CDAN1 | codanin 1 | 146059 |
| 16378 | -1.8912 | 0.490365 | 2.09E-02 | 0.052699 | 0.533021 | H200018449 | NA | NA | 339692 |
| 518 | -3.126 | 0.251056 | 7.31E-04 | 0.009914 | 0.533085 | H200002909 | TPRG1L | tumor protein p63 regulated 1-like | 127262 |
| 20456 | -1.8885 | 0.448178 | 2.11E-02 | 0.052958 | 0.533371 | H200003355 | NA | NA | - |
| 11662 | -2.375 | 0.461688 | 5.25E-03 | 0.024597 | 0.533437 | H200011266 | EPB41L3 | erythrocyte membrane protein band 4.1-like 3 | 23136 |
| 11914 | -2.7713 | 0.139358 | 1.79E-03 | 0.01462 | 0.533446 | H200001635 | ESYT1 | extended synaptotagmin-like protein 1 | 23344 |
| 11981 | -2.7904 | 0.249161 | 1.71E-03 | 0.014256 | 0.533483 | H200004717 | ALG1 | ALG1, chitobiosyldiphosphodolichol beta-mannosyltransferase | 56052 |
| 7047 | -3.2554 | 0.235906 | 5.42E-04 | 0.008915 | 0.533564 | H200009113 | NA | NA | - |
| 9395 | -2.0382 | 0.510527 | 1.37E-02 | 0.041818 | 0.533942 | H200012187 | ZNF777 | zinc finger protein 777 | 27153 |
| 1346 | -2.2939 | 0.351243 | 6.62E-03 | 0.027832 | 0.53401 | H200020638 | NA | NA | - |
| 8151 | -1.9087 | 0.432442 | 1.99E-02 | 0.051359 | 0.534049 | H200018125 | CDKL1 | cyclin-dependent kinase-like 1 (CDC2-related kinase) | 8814 |
| 15297 | -2.0392 | 0.100479 | 1.37E-02 | 0.041763 | 0.534222 | H200010451 | ABCC3 | ATP-binding cassette, sub-family C (CFTR/MRP), member 3 | 8714 |
| 16240 | -3.7809 | 0.249489 | 1.73E-04 | 0.006053 | 0.534236 | H200011681 | CBFA2T3 | core-binding factor, runt domain, alpha subunit 2; translocated to, 3 | 863 |
| 641 | -2.1083 | 0.396238 | 1.12E-02 | 0.037598 | 0.534293 | H200008935 | NA | NA | - |
| 13484 | -2.0757 | 0.587434 | 1.23E-02 | 0.039392 | 0.534357 | H200011005 | JAK3 | Janus kinase 3 | 3718 |
| 18628 | -3.4632 | 0.143562 | 3.38E-04 | 0.007534 | 0.534771 | H200018763 | FBXO31 | F-box protein 31 | 79791 |
| 20478 | -2.8147 | 0.370862 | 1.60E-03 | 0.013842 | 0.534783 | H200004513 | LOC256021 | uncharacterized LOC256021 | 256021 |
| 6071 | -1.8376 | 0.453432 | 2.43E-02 | 0.057629 | 0.534966 | H200006393 | PLP2 | proteolipid protein 2 (colonic epithelium-enriched) | 5355 |
| 2286 | -1.842 | 0.389159 | 2.40E-02 | 0.057224 | 0.535062 | H200000296 | LMO1 | LIM domain only 1 (rhombotin 1) | 4004 |
| 10856 | -1.7981 | 0.489469 | 2.72E-02 | 0.061667 | 0.5351 | H200016338 | NA | NA | - |
| 4997 | -2.3248 | 0.356528 | 6.07E-03 | 0.026632 | 0.535165 | H200020299 | LINC00167 | long intergenic non-protein coding RNA 167 | 440072 |
| 8458 | -2.5118 | 0.30575 | 3.59E-03 | 0.020489 | 0.535341 | H200011136 | FAM149A | family with sequence similarity 149, member A | 25854 |
| 19671 | -2.0525 | 0.38767 | 1.32E-02 | 0.040762 | 0.535347 | H200006336 | BDH1 | 3-hydroxybutyrate dehydrogenase, type 1 | 622 |
| 14938 | -2.3321 | 0.455409 | 5.94E-03 | 0.026275 | 0.535511 | H200014922 | FAM110D | family with sequence similarity 110, member D | 79927 |
| 2583 | -1.9391 | 0.492172 | 1.82E-02 | 0.049042 | 0.53568 | H200014374 | NA | NA | - |
| 573 | -3.4503 | 0.222984 | 3.49E-04 | 0.007612 | 0.535708 | H200005563 | ZSWIM8 | zinc finger, SWIM-type containing 8 | 23053 |
| 4521 | -1.9948 | 0.564398 | 1.56E-02 | 0.044842 | 0.535915 | H200019574 | C14orf144 | chromosome 14 open reading frame 144 | 145195 |
| 7008 | -3.2773 | 0.226212 | 5.15E-04 | 0.008735 | 0.535954 | H200007219 | NA | NA | - |
| 17365 | -2.3876 | 0.312949 | 5.07E-03 | 0.024172 | 0.536088 | H200008489 | RPL5 | ribosomal protein L5 | 6125 |
| 19382 | -2.5819 | 0.246891 | 2.97E-03 | 0.018531 | 0.53631 | H200014215 | ADD1 | adducin 1 (alpha) | 118 |
| 17689 | -1.9362 | 0.408989 | 1.84E-02 | 0.049255 | 0.536433 | H200015243 | SCUBE2 | signal peptide, CUB domain, EGF-like 2 | 57758 |
| 5111 | -3.2916 | 0.269037 | 4.99E-04 | 0.008656 | 0.536441 | H200004078 | TLE3 | transducin-like enhancer of split 3 (E(sp1) homolog, Drosophila) | 7090 |
| 5308 | -2.1747 | 0.337176 | 9.30E-03 | 0.033887 | 0.536497 | H200013536 | AP5S1 | adaptor-related protein complex 5, sigma 1 subunit | 55317 |
| 19546 | -2.6427 | 0.281444 | 2.52E-03 | 0.017125 | 0.536511 | H200000582 | CHGB | chromogranin B (secretogranin 1) | 1114 |
| 499 | -3.5065 | 0.14412 | 3.08E-04 | 0.007429 | 0.536535 | H200002119 | ADCY6 | adenylate cyclase 6 | 112 |
| 644 | -2.3348 | 0.416382 | 5.90E-03 | 0.026209 | 0.536555 | H200008965 | NA | NA | - |
| 18416 | -2.4544 | 0.326923 | 4.21E-03 | 0.022146 | 0.536592 | H200007339 | PTGDR | prostaglandin D2 receptor (DP) | 5729 |
| 14775 | -2.8678 | 0.167998 | 1.40E-03 | 0.012925 | 0.536614 | H200007008 | NA | NA | - |
| 20544 | -3.7815 | 0.218359 | 1.72E-04 | 0.006053 | 0.536712 | H200007963 | DOCK8 | dedicator of cytokinesis 8 | 81704 |
| 3561 | -2.2395 | 0.122419 | 7.74E-03 | 0.030653 | 0.537095 | H200017402 | A4GNT | alpha-1,4-N-acetylglucosaminyltransferase | 51146 |
| 2559 | -2.8199 | 0.2244 | 1.58E-03 | 0.013754 | 0.537282 | H200013234 | NA | NA | - |
| 7115 | -2.2533 | 0.358328 | 7.44E-03 | 0.029988 | 0.537354 | H200012485 | NRXN2 | neurexin 2 | 9379 |
| 21471 | -2.4636 | 0.360884 | 4.11E-03 | 0.02199 | 0.537396 | H200010231 | MED18 | mediator complex subunit 18 | 54797 |
| 66 | -2.7624 | 0.247984 | 1.83E-03 | 0.014822 | 0.537409 | H200003146 | STK11IP | serine/threonine kinase 11 interacting protein | 114790 |
| 19858 | -2.4312 | 0.323446 | 4.49E-03 | 0.022685 | 0.537553 | H200015402 | CCDC9 | coiled-coil domain containing 9 | 26093 |
| 13053 | -1.8722 | 0.174657 | 2.21E-02 | 0.054399 | 0.537734 | H200012210 | EIF3L | eukaryotic translation initiation factor 3, subunit L | 51386 |
| 8824 | -2.5477 | 0.360626 | 3.27E-03 | 0.019477 | 0.538106 | H200006743 | NA | NA | - |
| 3919 | -2.9252 | 0.271376 | 1.20E-03 | 0.012124 | 0.538503 | H200012331 | IL12RB1 | interleukin 12 receptor, beta 1 | 3594 |
| 1448 | -2.5453 | 0.338288 | 3.28E-03 | 0.01954 | 0.538809 | H200003705 | RASSF1 | Ras association (RalGDS/AF-6) domain family member 1 | 11186 |
| 17708 | -3.4854 | 0.250079 | 3.23E-04 | 0.007429 | 0.539107 | H200016045 | HOXA3 | homeobox A3 | 3200 |
| 8114 | -2.6577 | 0.490287 | 2.43E-03 | 0.016906 | 0.539151 | H200016539 | NAAA | N-acylethanolamine acid amidase | 27163 |
| 19721 | -2.662 | 0.247858 | 2.40E-03 | 0.016816 | 0.539261 | H200008640 | ACR | acrosin | 49 |
| 2225 | -2.3092 | 0.568259 | 6.34E-03 | 0.027123 | 0.539295 | H200019005 | NA | NA | - |
| 12138 | -2.8043 | 0.365134 | 1.65E-03 | 0.014047 | 0.539299 | H200012275 | ZNHIT2 | zinc finger, HIT-type containing 2 | 741 |
| 3659 | -3.015 | 0.218418 | 9.61E-04 | 0.01096 | 0.539406 | H200000123 | SLC18A3 | solute carrier family 18 (vesicular acetylcholine), member 3 | 6572 |
| 18753 | -2.5567 | 0.253714 | 3.18E-03 | 0.019239 | 0.539482 | H200004014 | NA | NA | - |
| 540 | -2.3884 | 0.5017 | 5.06E-03 | 0.024149 | 0.539514 | H200004025 | KCNE3 | potassium voltage-gated channel, Isk-related family, member 3 | 10008 |
| 12449 | -2.6888 | 0.269197 | 2.23E-03 | 0.016186 | 0.539517 | H200005524 | TRMT2A | tRNA methyltransferase 2 homolog A (S. cerevisiae) | 27037 |
| 16185 | -2.0162 | 0.693004 | 1.46E-02 | 0.043443 | 0.539723 | H200009323 | MYCT1 | myc target 1 | 80177 |
| 13630 | -2.4289 | 0.28209 | 4.52E-03 | 0.022749 | 0.539772 | H200018153 | NA | NA | - |
| 12394 | -3.0009 | 0.162748 | 9.95E-04 | 0.011169 | 0.539917 | H200002870 | ZFYVE1 | zinc finger, FYVE domain containing 1 | 53349 |
| 18995 | -2.4583 | 0.428134 | 4.17E-03 | 0.022089 | 0.54071 | H200016982 | AGRN | agrin | 375790 |
| 12594 | -3.5627 | 0.200186 | 2.68E-04 | 0.007016 | 0.540749 | H200012370 | KIAA1661 | KIAA1661 protein | 85375 |
| 9734 | -1.9268 | 3.277172 | 1.89E-02 | 0.049907 | 0.540758 | H200006754 | KRT15 | keratin 15 | 3866 |
| 6317 | -2.0583 | 0.718249 | 1.30E-02 | 0.040416 | 0.540792 | H200018149 | DBNDD2 | dysbindin (dystrobrevin binding protein 1) domain containing 2 | 55861 |
| 8553 | -4.2899 | 0.154256 | 7.04E-05 | 0.004908 | 0.540931 | H200015690 | C19orf43 | chromosome 19 open reading frame 43 | 79002 |
| 16600 | -3.1371 | 0.282424 | 7.10E-04 | 0.009862 | 0.541088 | H200006919 | RGS10 | regulator of G-protein signaling 10 | 6001 |
| 3280 | -2.4823 | 0.256455 | 3.90E-03 | 0.021359 | 0.541159 | H200003800 | STK32C | serine/threonine kinase 32C | 282974 |
| 3699 | -2.9791 | 0.267743 | 1.05E-03 | 0.011443 | 0.541181 | H200002023 | NOTCH4 | notch 4 | 4855 |
| 9330 | -3.1817 | 0.269299 | 6.43E-04 | 0.009436 | 0.54121 | H200009129 | SIGLEC15 | sialic acid binding Ig-like lectin 15 | 284266 |
| 636 | -3.0109 | 0.160545 | 9.71E-04 | 0.01101 | 0.541297 | H200008585 | ENDOG | endonuclease G | 2021 |
| 4224 | -2.6187 | 0.320273 | 2.69E-03 | 0.017693 | 0.541297 | H200005212 | C1orf105 | chromosome 1 open reading frame 105 | 92346 |
| 2131 | -3.1787 | 0.283994 | 6.48E-04 | 0.009443 | 0.541319 | H200014469 | NA | NA | - |
| 19190 | -1.925 | 0.57416 | 1.90E-02 | 0.050015 | 0.541369 | H200005095 | PDE6H | phosphodiesterase 6H, cGMP-specific, cone, gamma | 5149 |
| 16727 | -3.126 | 0.243686 | 7.32E-04 | 0.009914 | 0.541395 | H200012993 | NA | NA | - |
| 6635 | -3.7419 | 0.201843 | 1.87E-04 | 0.006183 | 0.541461 | H200011108 | MAPT | microtubule-associated protein tau | 4137 |
| 5244 | -2.8515 | 0.248967 | 1.46E-03 | 0.013131 | 0.542011 | H200010496 | TMEM115 | transmembrane protein 115 | 11070 |
| 8185 | -2.3842 | 0.382294 | 5.12E-03 | 0.024315 | 0.542152 | H200019953 | MTHFSD | methenyltetrahydrofolate synthetase domain containing | 64779 |
| 1865 | -3.5293 | 0.221601 | 2.91E-04 | 0.007373 | 0.542523 | H200001905 | WBP1L | WW domain binding protein 1-like | 54838 |
| 1428 | -2.5364 | 0.319815 | 3.36E-03 | 0.019674 | 0.542699 | H200002897 | PTK2B | PTK2B protein tyrosine kinase 2 beta | 2185 |
| 10616 | -2.4547 | 0.336934 | 4.21E-03 | 0.022146 | 0.542768 | H200004938 | SEL1L3 | sel-1 suppressor of lin-12-like 3 (C. elegans) | 23231 |
| 3462 | -1.9775 | 0.425205 | 1.63E-02 | 0.04605 | 0.542768 | H200012516 | RNF186 | ring finger protein 186 | 54546 |
| 2942 | -2.9983 | 0.258425 | 1.00E-03 | 0.011181 | 0.54313 | H200009523 | NA | NA | - |
| 961 | -4.0315 | 0.097631 | 1.10E-04 | 0.005439 | 0.543146 | H200002392 | SDHAF2 | succinate dehydrogenase complex assembly factor 2 | 54949 |
| 2510 | -2.6855 | 0.353844 | 2.25E-03 | 0.016244 | 0.543177 | H200010936 | CEBPB | CCAAT/enhancer binding protein (C/EBP), beta | 1051 |
| 6150 | -2.656 | 0.270687 | 2.44E-03 | 0.016909 | 0.543196 | H200010175 | TWIST1 | twist basic helix-loop-helix transcription factor 1 | 7291 |
| 7322 | -1.8485 | 0.460169 | 2.36E-02 | 0.056628 | 0.543358 | H200000484 | POMC | proopiomelanocortin | 5443 |
| 4853 | -2.7254 | 0.23763 | 2.03E-03 | 0.0156 | 0.543389 | H200013459 | KCNH8 | potassium voltage-gated channel, subfamily H (eag-related), member 8 | 131096 |
| 18235 | -3.2483 | 0.323725 | 5.53E-04 | 0.008974 | 0.543529 | H200019684 | NA | NA | - |
| 15984 | -3.5088 | 0.159463 | 3.06E-04 | 0.007429 | 0.543545 | H200021086 | MEF2D | myocyte enhancer factor 2D | 4209 |
| 16770 | -3.2172 | 0.176153 | 5.92E-04 | 0.00931 | 0.543589 | H200015207 | RUNDC1 | RUN domain containing 1 | 146923 |
| 15876 | -2.324 | 0.389332 | 6.08E-03 | 0.02664 | 0.543734 | H200016098 | POU3F3 | POU class 3 homeobox 3 | 5455 |
| 1935 | -2.6521 | 0.367748 | 2.46E-03 | 0.017004 | 0.543805 | H200005017 | KLK5 | kallikrein-related peptidase 5 | 25818 |
| 16652 | -2.0564 | 0.343066 | 1.30E-02 | 0.040554 | 0.543892 | H200009531 | ZDHHC14 | zinc finger, DHHC-type containing 14 | 79683 |
| 6423 | -1.9266 | 0.251322 | 1.89E-02 | 0.049908 | 0.544048 | H200001180 | MBD1 | methyl-CpG binding domain protein 1 | 4152 |
| 10549 | -5.1897 | 0.078876 | 1.71E-05 | 0.003715 | 0.544436 | H200001868 | EMP3 | epithelial membrane protein 3 | 2014 |
| 7089 | -2.8639 | 0.349445 | 1.41E-03 | 0.012998 | 0.544523 | H200011321 | ZBTB7A | zinc finger and BTB domain containing 7A | 51341 |
| 9399 | -3.3652 | 0.126513 | 4.22E-04 | 0.008093 | 0.544584 | H200012235 | DNAAF3 | dynein, axonemal, assembly factor 3 | 352909 |
| 13221 | -3.0422 | 0.242342 | 8.98E-04 | 0.01067 | 0.544639 | H200020190 | ESYT2 | extended synaptotagmin-like protein 2 | 57488 |
| 4449 | -2.4294 | 0.34661 | 4.51E-03 | 0.022745 | 0.545083 | H200016154 | PTCH2 | patched 2 | 8643 |
| 19787 | -2.7429 | 0.252634 | 1.93E-03 | 0.015202 | 0.545123 | H200011988 | KLHL36 | kelch-like family member 36 | 79786 |
| 16470 | -2.2053 | 0.337711 | 8.53E-03 | 0.032173 | 0.545131 | H200000815 | NA | NA | 57245 |
| 1326 | -2.1777 | 0.142008 | 9.22E-03 | 0.033716 | 0.545281 | H200019546 | NA | NA | - |
| 21534 | -2.4389 | 0.321856 | 4.40E-03 | 0.022431 | 0.545325 | H200013265 | ERN1 | endoplasmic reticulum to nucleus signaling 1 | 2081 |
| 8111 | -2.0351 | 1.187553 | 1.39E-02 | 0.042061 | 0.545328 | H200016225 | APOBEC3G | apolipoprotein B mRNA editing enzyme, catalytic polypeptide-like 3G | 60489 |
| 21267 | -2.0286 | 0.4162 | 1.41E-02 | 0.042496 | 0.545419 | H200000683 | MLN | motilin | 4295 |
| 14738 | -2.3247 | 0.325905 | 6.07E-03 | 0.026632 | 0.545612 | H200005422 | PRADC1 | protease-associated domain containing 1 | 84279 |
| 19342 | -2.785 | 0.289037 | 1.73E-03 | 0.014333 | 0.545676 | H200012315 | KCNB2 | potassium voltage-gated channel, Shab-related subfamily, member 2 | 9312 |
| 7685 | -1.7744 | 0.533754 | 2.92E-02 | 0.064118 | 0.545773 | H200017626 | SLC25A18 | solute carrier family 25 (glutamate carrier), member 18 | 83733 |
| 2019 | -2.8383 | 0.243739 | 1.51E-03 | 0.013422 | 0.545832 | H200009149 | LOC143188 | uncharacterized LOC143188 | 143188 |
| 1003 | -3.1124 | 0.235017 | 7.57E-04 | 0.00998 | 0.545911 | H200004316 | HAND2 | heart and neural crest derivatives expressed 2 | 9464 |
| 10437 | -3.2495 | 0.203435 | 5.51E-04 | 0.008974 | 0.546066 | H200018255 | NA | NA | - |
| 11064 | -1.8275 | 0.461009 | 2.51E-02 | 0.058576 | 0.546092 | H200004355 | NA | NA | - |
| 2701 | -2.5397 | 0.311572 | 3.33E-03 | 0.019619 | 0.546103 | H200020050 | NA | NA | - |
| 3395 | -1.9466 | 0.101291 | 1.78E-02 | 0.04839 | 0.546179 | H200009446 | GDPD3 | glycerophosphodiester phosphodiesterase domain containing 3 | 79153 |
| 4323 | -2.0882 | 0.373386 | 1.19E-02 | 0.03868 | 0.546346 | H200010098 | CMPK1 | cytidine monophosphate (UMP-CMP) kinase 1, cytosolic | 51727 |
| 17421 | -2.2814 | 0.345175 | 6.86E-03 | 0.028404 | 0.546351 | H200002371 | ANKRD13D | ankyrin repeat domain 13 family, member D | 338692 |
| 20042 | -3.7289 | 0.175458 | 1.91E-04 | 0.006226 | 0.546537 | H200002358 | ARHGAP17 | Rho GTPase activating protein 17 | 55114 |
| 5158 | -2.1693 | 0.408649 | 9.45E-03 | 0.034192 | 0.546588 | H200006340 | ELP5 | elongator acetyltransferase complex subunit 5 | 23587 |
| 15104 | -2.1107 | 0.442169 | 1.12E-02 | 0.037516 | 0.546629 | H200001029 | LGALS4 | lectin, galactoside-binding, soluble, 4 | 3960 |
| 16312 | -1.9156 | 0.430536 | 1.95E-02 | 0.050821 | 0.546896 | H200015101 | DCSTAMP | dendrocyte expressed seven transmembrane protein | 81501 |
| 11018 | -2.6369 | 0.190652 | 2.56E-03 | 0.017274 | 0.547025 | H200002383 | NA | NA | 256306 |
| 1984 | -2.3661 | 0.325766 | 5.39E-03 | 0.024946 | 0.547059 | H200007303 | ITGA10 | integrin, alpha 10 | 8515 |
| 9282 | -2.2792 | 0.429131 | 6.90E-03 | 0.028531 | 0.547088 | H200006849 | NME3 | NME/NM23 nucleoside diphosphate kinase 3 | 4832 |
| 15187 | -3.4115 | 0.208999 | 3.78E-04 | 0.007743 | 0.547333 | H200005155 | ORAI1 | ORAI calcium release-activated calcium modulator 1 | 84876 |
| 19643 | -2.2481 | 0.609322 | 7.55E-03 | 0.030292 | 0.547361 | H200005148 | EHD4 | EH-domain containing 4 | 30844 |
| 1328 | -2.2412 | 0.333871 | 7.70E-03 | 0.030606 | 0.547607 | H200019570 | NA | NA | - |
| 15338 | -2.7894 | 0.268003 | 1.71E-03 | 0.014261 | 0.547645 | H200012357 | ZNF653 | zinc finger protein 653 | 115950 |
| 8501 | -3.0926 | 0.108621 | 7.94E-04 | 0.010187 | 0.547832 | H200013078 | SLC39A3 | solute carrier family 39 (zinc transporter), member 3 | 29985 |
| 10389 | -2.1469 | 0.626775 | 1.01E-02 | 0.035362 | 0.547883 | H200015975 | NA | NA | - |
| 2531 | -2.8047 | 0.247091 | 1.64E-03 | 0.014047 | 0.547914 | H200011762 | ITM2C | integral membrane protein 2C | 81618 |
| 16049 | -3.1736 | 0.294968 | 6.56E-04 | 0.009538 | 0.547923 | H200002863 | NA | NA | - |
| 4712 | -2.3215 | 0.142903 | 6.13E-03 | 0.02668 | 0.54801 | H200006649 | GLB1 | galactosidase, beta 1 | 2720 |
| 6305 | -3.4857 | 0.17965 | 3.23E-04 | 0.007429 | 0.548039 | H200017437 | PSMD13 | proteasome (prosome, macropain) 26S subunit, non-ATPase, 13 | 5719 |
| 13528 | -2.9471 | 0.274135 | 1.13E-03 | 0.011769 | 0.548109 | H200013237 | ZNF423 | zinc finger protein 423 | 23090 |
| 12453 | -2.9218 | 0.281772 | 1.21E-03 | 0.012146 | 0.548318 | H200005572 | CLEC3B | C-type lectin domain family 3, member B | 7123 |
| 13916 | -2.671 | 0.272326 | 2.34E-03 | 0.016608 | 0.548467 | H200009592 | TMEM71 | transmembrane protein 71 | 137835 |
| 6121 | -2.1202 | 0.460071 | 1.09E-02 | 0.03697 | 0.548527 | H200008697 | NA | NA | - |
| 2291 | -2.8768 | 0.499981 | 1.36E-03 | 0.012772 | 0.548547 | H200000362 | MGMT | O-6-methylguanine-DNA methyltransferase | 4255 |
| 19884 | -2.0621 | 0.458051 | 1.28E-02 | 0.04011 | 0.548745 | H200016566 | TMPRSS13 | transmembrane protease, serine 13 | 84000 |
| 17659 | -1.887 | 0.461801 | 2.11E-02 | 0.053091 | 0.548921 | H200013747 | NA | NA | - |
| 19481 | -3.1246 | 0.192736 | 7.34E-04 | 0.009931 | 0.548986 | H200018805 | FHAD1 | forkhead-associated (FHA) phosphopeptide binding domain 1 | 114827 |
| 2680 | -2.0492 | 0.579463 | 1.33E-02 | 0.040947 | 0.549078 | H200018940 | NA | NA | - |
| 16365 | -3.0066 | 0.254116 | 9.82E-04 | 0.011076 | 0.549164 | H200017731 | SLC22A17 | solute carrier family 22, member 17 | 51310 |
| 2429 | -2.2507 | 0.378328 | 7.50E-03 | 0.030119 | 0.549221 | H200007130 | CENPB | centromere protein B, 80kDa | 1059 |
| 12641 | -2.3649 | 0.476027 | 5.41E-03 | 0.024999 | 0.549292 | H200014644 | PEA15 | phosphoprotein enriched in astrocytes 15 | 8682 |
| 21284 | -2.3209 | 0.357011 | 6.14E-03 | 0.02668 | 0.54937 | H200001461 | C19orf25 | chromosome 19 open reading frame 25 | 148223 |
| 2995 | -2.712 | 0.292015 | 2.10E-03 | 0.015819 | 0.549493 | H200011869 | LINC00302 | long intergenic non-protein coding RNA 302 | 388699 |
| 5680 | -3.271 | 0.212407 | 5.23E-04 | 0.008802 | 0.549671 | H200009202 | CREB3 | cAMP responsive element binding protein 3 | 10488 |
| 5830 | -2.3692 | 0.34414 | 5.35E-03 | 0.024821 | 0.549899 | H200016398 | BIRC7 | baculoviral IAP repeat containing 7 | 79444 |
| 13862 | -2.8826 | 0.258487 | 1.34E-03 | 0.012598 | 0.54997 | H200007240 | IVL | involucrin | 3713 |
| 16675 | -2.7521 | 0.401947 | 1.88E-03 | 0.015031 | 0.550304 | H200010665 | ABCA2 | ATP-binding cassette, sub-family A (ABC1), member 2 | 20 |
| 19137 | -2.2322 | 0.314697 | 7.91E-03 | 0.030947 | 0.550517 | H200002465 | SNRNP25 | small nuclear ribonucleoprotein 25kDa (U11/U12) | 79622 |
| 13075 | -2.4072 | 0.288051 | 4.80E-03 | 0.023472 | 0.550523 | H200013326 | NA | NA | - |
| 1979 | -2.4303 | 0.403946 | 4.50E-03 | 0.02272 | 0.550731 | H200007249 | PYY2 | peptide YY, 2 (pseudogene) | 23615 |
| 9370 | -1.8955 | 0.503278 | 2.06E-02 | 0.052372 | 0.550755 | H200011029 | RTN1 | reticulon 1 | 6252 |
| 6095 | -2.0523 | 0.308157 | 1.32E-02 | 0.040777 | 0.550984 | H200007533 | OR52K3P | olfactory receptor, family 52, subfamily K, member 3 pseudogene | 390035 |
| 14174 | -2.387 | 0.298445 | 5.08E-03 | 0.024205 | 0.551065 | H200000353 | CSF2 | colony stimulating factor 2 (granulocyte-macrophage) | 1437 |
| 4407 | -2.9141 | 0.122232 | 1.23E-03 | 0.012235 | 0.551157 | H200013946 | E4F1 | E4F transcription factor 1 | 1877 |
| 21253 | -1.8029 | 0.307721 | 2.69E-02 | 0.061217 | 0.55118 | H2NC000007 | NA | NA | - |
| 1778 | -1.7906 | 0.428708 | 2.78E-02 | 0.062447 | 0.551184 | H200019593 | ZNF626 | zinc finger protein 626 | 199777 |
| 2632 | -1.8686 | 0.408773 | 2.23E-02 | 0.054677 | 0.5513 | H200016660 | NA | NA | - |
| 16481 | -2.1391 | 0.29652 | 1.03E-02 | 0.035839 | 0.551344 | H200001521 | FUK | fucokinase | 197258 |
| 12474 | -2.5506 | 0.315755 | 3.24E-03 | 0.019442 | 0.551477 | H200006670 | ZNF592 | zinc finger protein 592 | 9640 |
| 19794 | -2.7457 | 0.28582 | 1.92E-03 | 0.015139 | 0.551523 | H200012362 | TTC23L | tetratricopeptide repeat domain 23-like | 153657 |
| 4522 | -2.2286 | 0.370496 | 7.99E-03 | 0.031096 | 0.551754 | H200019580 | NA | NA | - |
| 15914 | -1.8821 | 1.234398 | 2.14E-02 | 0.053454 | 0.55178 | H200017974 | NA | NA | - |
| 9247 | -2.1073 | 0.568553 | 1.13E-02 | 0.037664 | 0.551782 | H200005015 | HOXC4 | homeobox C4 | 3221 |
| 9890 | -2.0879 | 0.23447 | 1.19E-02 | 0.038694 | 0.551815 | H200014022 | DDX28 | DEAD (Asp-Glu-Ala-Asp) box polypeptide 28 | 55794 |
| 20068 | -1.8616 | 0.511489 | 2.27E-02 | 0.05531 | 0.551828 | H200003854 | ZCCHC3 | zinc finger, CCHC domain containing 3 | 85364 |
| 16478 | -3.2653 | 0.224708 | 5.30E-04 | 0.008857 | 0.551835 | H200001195 | NA | NA | - |
| 4422 | -1.9912 | 0.440674 | 1.57E-02 | 0.045068 | 0.551894 | H200014688 | CNTFR | ciliary neurotrophic factor receptor | 1271 |
| 14958 | -1.8209 | 0.293278 | 2.55E-02 | 0.059307 | 0.551905 | H200015730 | NA | NA | - |
| 868 | -2.3213 | 0.297114 | 6.13E-03 | 0.02668 | 0.552101 | H200019605 | PSEN1 | presenilin 1 | 5663 |
| 19950 | -2.4418 | 0.324603 | 4.36E-03 | 0.022353 | 0.552274 | H200019630 | HDAC5 | histone deacetylase 5 | 10014 |
| 3568 | -2.1054 | 0.296022 | 1.13E-02 | 0.037803 | 0.55234 | H200017480 | CHMP4A | charged multivesicular body protein 4A | 29082 |
| 5064 | -2.133 | 0.430248 | 1.05E-02 | 0.036188 | 0.552405 | H200001804 | NA | NA | - |
| 11466 | -3.0839 | 0.160262 | 8.11E-04 | 0.010284 | 0.552475 | H200002098 | F11R | F11 receptor | 50848 |
| 17139 | -2.0976 | 0.201881 | 1.16E-02 | 0.038192 | 0.552508 | H200010980 | ASB13 | ankyrin repeat and SOCS box containing 13 | 79754 |
| 6641 | -2.3349 | 0.184536 | 5.89E-03 | 0.026209 | 0.552538 | H200011464 | BAP1 | BRCA1 associated protein-1 (ubiquitin carboxy-terminal hydrolase) | 8314 |
| 8653 | -2.9371 | 0.132474 | 1.17E-03 | 0.01193 | 0.552651 | H200020298 | LENG8 | leukocyte receptor cluster (LRC) member 8 | 114823 |
| 5279 | -2.2186 | 0.306327 | 8.22E-03 | 0.03151 | 0.552655 | H200012058 | NNAT | neuronatin | 4826 |
| 3045 | -2.4138 | 0.374073 | 4.71E-03 | 0.023284 | 0.552673 | H200014457 | FANCD2OS | FANCD2 opposite strand | 115795 |
| 1798 | -2.0289 | 0.360059 | 1.41E-02 | 0.042473 | 0.552706 | H200020401 | SLC1A2 | solute carrier family 1 (glial high affinity glutamate transporter), member 2 | 6506 |
| 10315 | -2.5077 | 0.286777 | 3.64E-03 | 0.020559 | 0.553243 | H200012247 | NT5C1B | 5'-nucleotidase, cytosolic IB | 93034 |
| 206 | -2.0005 | 0.514313 | 1.53E-02 | 0.044497 | 0.553355 | H200009654 | ELL3 | elongation factor RNA polymerase II-like 3 | 80237 |
| 10936 | -2.374 | 0.199291 | 5.27E-03 | 0.02461 | 0.553517 | H200020138 | GRAMD1B | GRAM domain containing 1B | 57476 |
| 4411 | -2.0962 | 0.264013 | 1.16E-02 | 0.038245 | 0.553886 | H200014278 | RASA4 | RAS p21 protein activator 4 | 10156 |
| 615 | -1.898 | 0.75125 | 2.05E-02 | 0.052205 | 0.554003 | H200007487 | KLK12 | kallikrein-related peptidase 12 | 43849 |
| 17331 | -3.051 | 0.246269 | 8.78E-04 | 0.010543 | 0.554015 | H200020100 | WDR65 | WD repeat domain 65 | 149465 |
| 20561 | -3.0737 | 0.218682 | 8.29E-04 | 0.010354 | 0.554038 | H200009055 | LOC90246 | uncharacterized LOC90246 | 90246 |
| 17765 | -2.6404 | 0.28148 | 2.54E-03 | 0.01716 | 0.554124 | H200018711 | NA | NA | - |
| 16579 | -1.8125 | 20.46287 | 2.62E-02 | 0.060187 | 0.554166 | H200006105 | SPARCL1 | SPARC-like 1 (hevin) | 8404 |
| 12999 | -2.1954 | 0.454918 | 8.77E-03 | 0.032653 | 0.554428 | H200009574 | TRIM47 | tripartite motif containing 47 | 91107 |
| 4260 | -2.9223 | 0.282573 | 1.21E-03 | 0.012146 | 0.554453 | H200007064 | IRF2 | interferon regulatory factor 2 | 3660 |
| 3264 | -2.1022 | 0.390703 | 1.14E-02 | 0.037941 | 0.554529 | H200003040 | ARMC7 | armadillo repeat containing 7 | 79637 |
| 18659 | -2.3219 | 0.302532 | 6.12E-03 | 0.02668 | 0.554605 | H200020313 | NA | NA | - |
| 1795 | -2.414 | 0.325026 | 4.71E-03 | 0.023282 | 0.554628 | H200020371 | KIR3DL3 | killer cell immunoglobulin-like receptor, three domains, long cytoplasmic tail, 3 | 115653 |
| 17177 | -2.9024 | 0.23884 | 1.28E-03 | 0.01238 | 0.55479 | H200012856 | ECE2 | endothelin converting enzyme 2 | 9718 |
| 8378 | -1.79 | 0.573595 | 2.79E-02 | 0.062532 | 0.554832 | H200007336 | BFSP2 | beaded filament structural protein 2, phakinin | 8419 |
| 475 | -3.1456 | 0.217578 | 6.99E-04 | 0.009835 | 0.554913 | H200000979 | CHMP7 | charged multivesicular body protein 7 | 91782 |
| 14842 | -1.8843 | 0.384606 | 2.13E-02 | 0.053277 | 0.55515 | H200010362 | NA | NA | - |
| 8622 | -2.9213 | 0.294679 | 1.21E-03 | 0.012146 | 0.555311 | H200018784 | SAMD10 | sterile alpha motif domain containing 10 | 140700 |
| 10889 | -2.6895 | 0.199907 | 2.23E-03 | 0.016186 | 0.555322 | H200018160 | SDF2L1 | stromal cell-derived factor 2-like 1 | 23753 |
| 15058 | -1.7762 | 0.396723 | 2.90E-02 | 0.063831 | 0.555574 | H200020622 | NA | NA | - |
| 19917 | -1.7881 | 0.509801 | 2.81E-02 | 0.062704 | 0.555977 | H200018092 | NA | NA | - |
| 11373 | -2.1632 | 0.451999 | 9.62E-03 | 0.0346 | 0.556073 | H200019145 | NA | NA | - |
| 12206 | -3.2192 | 0.300201 | 5.88E-04 | 0.009296 | 0.556245 | H200015363 | SZRD1 | SUZ RNA binding domain containing 1 | 26099 |
| 8531 | -1.8267 | 0.430267 | 2.51E-02 | 0.058663 | 0.55635 | H200014574 | PPEF2 | protein phosphatase, EF-hand calcium binding domain 2 | 5470 |
| 4286 | -2.3351 | 0.353274 | 5.89E-03 | 0.026209 | 0.556376 | H200008228 | MYO16 | myosin XVI | 23026 |
| 16791 | -2.413 | 0.303084 | 4.72E-03 | 0.023284 | 0.556607 | H200016033 | NA | NA | - |
| 9800 | -2.2403 | 0.285144 | 7.72E-03 | 0.030646 | 0.556754 | H200009818 | DUSP15 | dual specificity phosphatase 15 | 128853 |
| 15813 | -2.6508 | 0.255621 | 2.47E-03 | 0.017017 | 0.556815 | H200013076 | NA | NA | - |
| 8642 | -2.8591 | 0.282711 | 1.43E-03 | 0.013052 | 0.55684 | H200019876 | TRMT61A | tRNA methyltransferase 61 homolog A (S. cerevisiae) | 115708 |
| 12767 | -3.0712 | 0.195796 | 8.36E-04 | 0.010387 | 0.556924 | H200020416 | MLLT6 | myeloid/lymphoid or mixed-lineage leukemia (trithorax homolog, Drosophila); translocated to, 6 | 4302 |
| 7021 | -2.1956 | 0.315189 | 8.76E-03 | 0.03265 | 0.557032 | H200007949 | GAD2 | glutamate decarboxylase 2 (pancreatic islets and brain, 65kDa) | 2572 |
| 6080 | -2.2429 | 0.302447 | 7.67E-03 | 0.030511 | 0.557076 | H200006779 | RAPGEF5 | Rap guanine nucleotide exchange factor (GEF) 5 | 9771 |
| 14258 | -3.2435 | 0.170454 | 5.59E-04 | 0.00902 | 0.557101 | H200004485 | HOXC9 | homeobox C9 | 3225 |
| 7745 | -2.3572 | 0.251619 | 5.53E-03 | 0.025347 | 0.557254 | H200020618 | CDH24 | cadherin 24, type 2 | 64403 |
| 6971 | -3.2599 | 0.083299 | 5.36E-04 | 0.008903 | 0.55727 | H200005645 | BIN3 | bridging integrator 3 | 55909 |
| 16696 | -2.1831 | 0.329278 | 9.08E-03 | 0.033378 | 0.5573 | H200011479 | NA | NA | - |
| 12748 | -2.3128 | 0.29447 | 6.28E-03 | 0.027011 | 0.557394 | H200019614 | WDR83 | WD repeat domain 83 | 84292 |
| 11595 | -4.6536 | 0.160531 | 3.88E-05 | 0.004216 | 0.557433 | H200008196 | NA | NA | - |
| 19502 | -1.8566 | 0.636491 | 2.31E-02 | 0.055887 | 0.557501 | H200019915 | KLHDC7B | kelch domain containing 7B | 113730 |
| 19938 | -2.3435 | 0.195817 | 5.75E-03 | 0.025895 | 0.557708 | H200019202 | TRPT1 | tRNA phosphotransferase 1 | 83707 |
| 15206 | -2.9776 | 0.239745 | 1.05E-03 | 0.011443 | 0.557788 | H200005945 | CAPNS1 | calpain, small subunit 1 | 826 |
| 21166 | -2.071 | 0.488694 | 1.25E-02 | 0.039577 | 0.557799 | H200017492 | CAMK1D | calcium/calmodulin-dependent protein kinase ID | 57118 |
| 19283 | -2.9746 | 0.272804 | 1.06E-03 | 0.011443 | 0.557918 | H200009613 | NA | NA | - |
| 6938 | -1.8008 | 0.474081 | 2.70E-02 | 0.061444 | 0.557963 | H200004107 | TRPM4 | transient receptor potential cation channel, subfamily M, member 4 | 54795 |
| 6208 | -2.6283 | 0.25628 | 2.62E-03 | 0.017447 | 0.558016 | H200012859 | TSPAN9 | tetraspanin 9 | 10867 |
| 14974 | -2.502 | 0.254275 | 3.69E-03 | 0.020751 | 0.558059 | H200016490 | TRO | trophinin | 7216 |
| 7199 | -2.1952 | 0.397596 | 8.77E-03 | 0.032653 | 0.558122 | H200016333 | OTUD7B | OTU domain containing 7B | 56957 |
| 13798 | -1.842 | 0.473121 | 2.40E-02 | 0.057224 | 0.558204 | H200004200 | GUCA2B | guanylate cyclase activator 2B (uroguanylin) | 2981 |
| 20879 | -2.4037 | 0.266123 | 4.85E-03 | 0.023571 | 0.558249 | H200003818 | KIF12 | kinesin family member 12 | 113220 |
| 10492 | -2.0093 | 0.390758 | 1.49E-02 | 0.043741 | 0.558319 | H200020613 | NA | NA | - |
| 17707 | -2.2169 | 0.475557 | 8.26E-03 | 0.031577 | 0.55835 | H200016027 | NFATC2 | nuclear factor of activated T-cells, cytoplasmic, calcineurin-dependent 2 | 4773 |
| 16262 | -3.278 | 0.260222 | 5.14E-04 | 0.008735 | 0.558453 | H200012797 | NOTCH1 | notch 1 | 4851 |
| 12725 | -2.0961 | 0.264522 | 1.16E-02 | 0.038245 | 0.558657 | H200018492 | NA | NA | - |
| 9740 | -2.8629 | 0.237288 | 1.41E-03 | 0.012998 | 0.558789 | H200006826 | NA | NA | - |
| 11192 | -2.9762 | 0.222446 | 1.06E-03 | 0.011443 | 0.558962 | H200010435 | SPAG7 | sperm associated antigen 7 | 9552 |
| 10173 | -1.964 | 0.448519 | 1.70E-02 | 0.047134 | 0.559088 | H200005715 | ATP9A | ATPase, class II, type 9A | 10079 |
| 21219 | -2.449 | 0.253683 | 4.28E-03 | 0.02224 | 0.559149 | H200019826 | DLK2 | delta-like 2 homolog (Drosophila) | 65989 |
| 7770 | -2.9992 | 0.198308 | 9.98E-04 | 0.011176 | 0.559283 | H200000199 | GUCA2A | guanylate cyclase activator 2A (guanylin) | 2980 |
| 15089 | -2.5824 | 0.224778 | 2.97E-03 | 0.018531 | 0.559314 | H200000571 | LIF | leukemia inhibitory factor | 3976 |
| 4342 | -4.7328 | 0.087218 | 3.46E-05 | 0.004076 | 0.559447 | H200010888 | ZC3H3 | zinc finger CCCH-type containing 3 | 23144 |
| 7108 | -2.1217 | 0.343958 | 1.08E-02 | 0.036909 | 0.559569 | H200012111 | FSCN1 | fascin homolog 1, actin-bundling protein (Strongylocentrotus purpuratus) | 6624 |
| 8196 | -5.1607 | 0.109607 | 1.79E-05 | 0.003715 | 0.559629 | H200020363 | SPTBN1 | spectrin, beta, non-erythrocytic 1 | 6711 |
| 21503 | -1.8804 | 0.375219 | 2.15E-02 | 0.053671 | 0.559631 | H200011751 | SLC27A5 | solute carrier family 27 (fatty acid transporter), member 5 | 10998 |
| 16376 | -2.245 | 0.457263 | 7.62E-03 | 0.030454 | 0.559706 | H200018141 | TRPV6 | transient receptor potential cation channel, subfamily V, member 6 | 55503 |
| 2861 | -3.1154 | 0.17804 | 7.52E-04 | 0.009958 | 0.559803 | H200005717 | HMP19 | HMP19 protein | 51617 |
| 15129 | -6.0379 | 0.062945 | 4.09E-06 | 0.003715 | 0.559926 | H200002471 | KIAA1407 | KIAA1407 | 57577 |
| 533 | -2.9524 | 0.229187 | 1.12E-03 | 0.011719 | 0.560071 | H200003663 | HDAC10 | histone deacetylase 10 | 83933 |
| 15173 | -2.6693 | 0.274174 | 2.35E-03 | 0.01662 | 0.560085 | H200004419 | GHDC | GH3 domain containing | 84514 |
| 10727 | -2.4528 | 0.355219 | 4.23E-03 | 0.022178 | 0.560246 | H200010252 | PRKACB | protein kinase, cAMP-dependent, catalytic, beta | 5567 |
| 10844 | -1.7998 | 0.347749 | 2.71E-02 | 0.061542 | 0.56026 | H200015910 | KRT3 | keratin 3 | 3850 |
| 15363 | -3.218 | 0.199345 | 5.90E-04 | 0.00931 | 0.560437 | H200013515 | NA | NA | - |
| 17336 | -2.4106 | 0.297239 | 4.76E-03 | 0.023357 | 0.560502 | H200020450 | NA | NA | - |
| 5618 | -3.6817 | 0.151975 | 2.09E-04 | 0.006367 | 0.560658 | H200006470 | MEF2D | myocyte enhancer factor 2D | 4209 |
| 3704 | -3.372 | 0.182149 | 4.14E-04 | 0.007987 | 0.560869 | H200002077 | C19orf66 | chromosome 19 open reading frame 66 | 55337 |
| 8956 | -1.9352 | 0.513435 | 1.84E-02 | 0.049314 | 0.561172 | H200013155 | ADAP1 | ArfGAP with dual PH domains 1 | 11033 |
| 6165 | -2.9418 | 0.231767 | 1.15E-03 | 0.011846 | 0.561289 | H200010929 | FAM154A | family with sequence similarity 154, member A | 158297 |
| 12236 | -1.8349 | 0.407075 | 2.45E-02 | 0.057812 | 0.561416 | H200016859 | PLXNA4 | plexin A4 | 91584 |
| 10077 | -2.6191 | 0.295399 | 2.69E-03 | 0.017693 | 0.561424 | H200001155 | IMP3 | IMP3, U3 small nucleolar ribonucleoprotein, homolog (yeast) | 55272 |
| 21349 | -2.4542 | 0.564058 | 4.22E-03 | 0.022146 | 0.561568 | H200004507 | EMCN | endomucin | 51705 |
| 2058 | -2.1579 | 0.388231 | 9.77E-03 | 0.034743 | 0.561748 | H200011031 | PIP | prolactin-induced protein | 5304 |
| 5636 | -3.1955 | 0.321764 | 6.22E-04 | 0.009361 | 0.561773 | H200007254 | C1QTNF5 | C1q and tumor necrosis factor related protein 5 | 114902 |
| 18073 | -2.3091 | 0.319242 | 6.34E-03 | 0.027123 | 0.562189 | H200011776 | IFT140 | intraflagellar transport 140 homolog (Chlamydomonas) | 9742 |
| 10141 | -1.7963 | 0.566323 | 2.74E-02 | 0.061772 | 0.562284 | H200004195 | FUT6 | fucosyltransferase 6 (alpha (1,3) fucosyltransferase) | 2528 |
| 21545 | -2.6751 | 0.205677 | 2.31E-03 | 0.016491 | 0.562492 | H200013675 | LRGUK | leucine-rich repeats and guanylate kinase domain containing | 136332 |
| 19959 | -3.5538 | 0.207103 | 2.73E-04 | 0.007084 | 0.562732 | H200020016 | NA | NA | - |
| 11444 | -2.0094 | 0.416961 | 1.49E-02 | 0.043741 | 0.562756 | H200000982 | TENM4 | teneurin transmembrane protein 4 | 26011 |
| 2540 | -2.1515 | 0.284003 | 9.94E-03 | 0.035107 | 0.562917 | H200012148 | NA | NA | - |
| 19002 | -2.1836 | 0.296625 | 9.06E-03 | 0.03337 | 0.562923 | H200017362 | IL36A | interleukin 36, alpha | 27179 |
| 2431 | -3.0985 | 0.152195 | 7.83E-04 | 0.01011 | 0.562977 | H200007154 | TRIM55 | tripartite motif containing 55 | 84675 |
| 12212 | -2.129 | 0.366968 | 1.06E-02 | 0.036487 | 0.563035 | H200015719 | SSTR5 | somatostatin receptor 5 | 6755 |
| 8373 | -4.1997 | 0.141525 | 8.12E-05 | 0.005233 | 0.5633 | H200006998 | PEX11B | peroxisomal biogenesis factor 11 beta | 8799 |
| 1558 | -1.8962 | 0.411533 | 2.06E-02 | 0.052337 | 0.563329 | H200009001 | NA | NA | - |
| 13878 | -2.3553 | 0.257867 | 5.56E-03 | 0.025427 | 0.563359 | H200008000 | DHX8 | DEAH (Asp-Glu-Ala-His) box polypeptide 8 | 1659 |
| 3934 | -3.8218 | 0.122024 | 1.59E-04 | 0.005936 | 0.563417 | H200013073 | ZDHHC12 | zinc finger, DHHC-type containing 12 | 84885 |
| 5416 | -3.0366 | 0.191314 | 9.08E-04 | 0.010685 | 0.563455 | H200018524 | NA | NA | - |
| 11231 | -2.3351 | 0.320853 | 5.89E-03 | 0.026209 | 0.563575 | H200012329 | SHH | sonic hedgehog | 6469 |
| 7044 | -2.4006 | 0.284034 | 4.89E-03 | 0.023701 | 0.563682 | H200009071 | NA | NA | - |
| 2322 | -2.5189 | 0.315428 | 3.53E-03 | 0.020279 | 0.563691 | H200001864 | MRPS12 | mitochondrial ribosomal protein S12 | 6183 |
| 10258 | -3.9012 | 0.104572 | 1.36E-04 | 0.005801 | 0.563867 | H200009569 | PRR14 | proline rich 14 | 78994 |
| 16979 | -3.1444 | 0.174995 | 7.01E-04 | 0.009846 | 0.564073 | H200003380 | SHISA5 | shisa homolog 5 (Xenopus laevis) | 51246 |
| 14281 | -3.0278 | 0.198137 | 9.30E-04 | 0.01078 | 0.564177 | H200005619 | CCDC85B | coiled-coil domain containing 85B | 11007 |
| 12175 | -3.6572 | 0.13257 | 2.20E-04 | 0.006519 | 0.564493 | H200013861 | GAK | cyclin G associated kinase | 2580 |
| 6324 | -1.9512 | 0.440212 | 1.76E-02 | 0.048156 | 0.564505 | H200018227 | NXF2 | nuclear RNA export factor 2 | 56001 |
| 8544 | -2.0012 | 0.250556 | 1.53E-02 | 0.044434 | 0.564517 | H200015008 | SMAGP | small cell adhesion glycoprotein | 57228 |
| 8455 | -1.8867 | 0.38079 | 2.12E-02 | 0.053112 | 0.56461 | H200010822 | CCDC62 | coiled-coil domain containing 62 | 84660 |
| 21409 | -1.9863 | 0.322364 | 1.59E-02 | 0.045393 | 0.564808 | H200007215 | MUSK | muscle, skeletal, receptor tyrosine kinase | 4593 |
| 16117 | -2.7969 | 0.262491 | 1.68E-03 | 0.014184 | 0.564834 | H200005951 | MLC1 | megalencephalic leukoencephalopathy with subcortical cysts 1 | 23209 |
| 9207 | -2.1377 | 0.200526 | 1.03E-02 | 0.0359 | 0.564847 | H200003115 | TMEM53 | transmembrane protein 53 | 79639 |
| 2083 | -2.4361 | 0.284202 | 4.43E-03 | 0.022505 | 0.564937 | H200012189 | SLC12A9 | solute carrier family 12 (potassium/chloride transporters), member 9 | 56996 |
| 6161 | -2.5201 | 0.188925 | 3.52E-03 | 0.02026 | 0.564964 | H200010597 | C11orf68 | chromosome 11 open reading frame 68 | 83638 |
| 10112 | -2.1495 | 0.20045 | 1.00E-02 | 0.035224 | 0.565098 | H200002705 | NA | NA | - |
| 6719 | -1.8885 | 0.489166 | 2.11E-02 | 0.052958 | 0.565244 | H200015240 | ZNF837 | zinc finger protein 837 | 116412 |
| 6003 | -2.3641 | 0.345165 | 5.42E-03 | 0.02503 | 0.565279 | H200003021 | PAK6 | p21 protein (Cdc42/Rac)-activated kinase 6 | 56924 |
| 9897 | -2.3012 | 0.361271 | 6.48E-03 | 0.027454 | 0.565307 | H200014396 | TIGD4 | tigger transposable element derived 4 | 201798 |
| 6480 | -2.6297 | 0.30192 | 2.61E-03 | 0.017425 | 0.565329 | H200003846 | MS4A14 | membrane-spanning 4-domains, subfamily A, member 14 | 84689 |
| 7482 | -2.226 | 0.218154 | 8.05E-03 | 0.031187 | 0.56538 | H200008084 | NA | NA | - |
| 12119 | -2.3225 | 0.279914 | 6.11E-03 | 0.02668 | 0.565584 | H200011201 | SLC13A3 | solute carrier family 13 (sodium-dependent dicarboxylate transporter), member 3 | 64849 |
| 16214 | -2.0136 | 0.271198 | 1.47E-02 | 0.043589 | 0.565698 | H200010517 | C11orf71 | chromosome 11 open reading frame 71 | 54494 |
| 86 | -3.1715 | 0.214162 | 6.59E-04 | 0.009552 | 0.565784 | H200003954 | NA | NA | 92454 |
| 14851 | -2.033 | 0.435184 | 1.39E-02 | 0.042244 | 0.565818 | H200010760 | TP53I11 | tumor protein p53 inducible protein 11 | 9537 |
| 10293 | -2.4942 | 0.283807 | 3.77E-03 | 0.020956 | 0.565863 | H200011415 | NKX3-2 | NK3 homeobox 2 | 579 |
| 5033 | -2.069 | 0.292964 | 1.26E-02 | 0.039652 | 0.565929 | H200000586 | DBH | dopamine beta-hydroxylase (dopamine beta-monooxygenase) | 1621 |
| 14301 | -2.6304 | 0.267448 | 2.61E-03 | 0.017425 | 0.565931 | H200006427 | STARD3 | StAR-related lipid transfer (START) domain containing 3 | 10948 |
| 10392 | -2.4553 | 0.354816 | 4.20E-03 | 0.022146 | 0.565965 | H200016005 | NA | NA | - |
| 8012 | -2.9818 | 0.322518 | 1.04E-03 | 0.011428 | 0.565967 | H200011623 | SH3BGRL3 | SH3 domain binding glutamic acid-rich protein like 3 | 83442 |
| 3767 | -3.4719 | 0.248806 | 3.33E-04 | 0.007506 | 0.566017 | H200005111 | TRIM8 | tripartite motif containing 8 | 81603 |
| 15721 | -2.6078 | 0.277191 | 2.77E-03 | 0.017955 | 0.566048 | H200008848 | UBTD1 | ubiquitin domain containing 1 | 80019 |
| 13166 | -2.442 | 0.274162 | 4.36E-03 | 0.022351 | 0.566367 | H200017536 | DCPS | decapping enzyme, scavenger | 28960 |
| 11747 | -2.1065 | 0.474308 | 1.13E-02 | 0.037724 | 0.566383 | H200015416 | MILR1 | mast cell immunoglobulin-like receptor 1 | 284021 |
| 12209 | -1.9712 | 0.416937 | 1.66E-02 | 0.046542 | 0.566419 | H200015689 | GPR35 | G protein-coupled receptor 35 | 2859 |
| 1869 | -2.5336 | 0.229866 | 3.39E-03 | 0.019794 | 0.566462 | H200001953 | GPATCH3 | G patch domain containing 3 | 63906 |
| 12778 | -3.8187 | 0.101533 | 1.60E-04 | 0.005936 | 0.566464 | H200021110 | DENND3 | DENN/MADD domain containing 3 | 22898 |
| 19039 | -3.1723 | 0.192485 | 6.58E-04 | 0.009552 | 0.56652 | H200019576 | NA | NA | - |
| 9277 | -3.4422 | 0.210526 | 3.56E-04 | 0.007621 | 0.566601 | H200006511 | NA | NA | - |
| 15055 | -2.6419 | 0.28852 | 2.53E-03 | 0.017125 | 0.566728 | H200020308 | NA | NA | - |
| 13012 | -3.0093 | 0.218407 | 9.76E-04 | 0.011047 | 0.566855 | H200010292 | ANAPC10 | anaphase promoting complex subunit 10 | 10393 |
| 18388 | -3.1153 | 0.149216 | 7.52E-04 | 0.009958 | 0.566913 | H200005819 | SLC10A3 | solute carrier family 10 (sodium/bile acid cotransporter family), member 3 | 8273 |
| 20971 | -2.9758 | 0.229893 | 1.06E-03 | 0.011443 | 0.56692 | H200008046 | LENG1 | leukocyte receptor cluster (LRC) member 1 | 79165 |
| 18510 | -2.7332 | 0.304703 | 1.98E-03 | 0.015408 | 0.567104 | H200012309 | E2F2 | E2F transcription factor 2 | 1870 |
| 18522 | -2.3151 | 0.402322 | 6.24E-03 | 0.026907 | 0.567183 | H200013045 | HIST1H3I | histone cluster 1, H3i | 8354 |
| 8047 | -3.4156 | 0.196991 | 3.74E-04 | 0.007706 | 0.567186 | H200013185 | NA | NA | - |
| 8582 | -3.1695 | 0.19429 | 6.62E-04 | 0.009571 | 0.567352 | H200016884 | PLA2G2E | phospholipase A2, group IIE | 30814 |
| 293 | -3.3376 | 0.122112 | 4.47E-04 | 0.008358 | 0.567505 | H200013828 | FAM65A | family with sequence similarity 65, member A | 79567 |
| 21010 | -2.3721 | 0.283427 | 5.30E-03 | 0.02471 | 0.567542 | H200009940 | NA | NA | - |
| 16679 | -2.5909 | 0.328829 | 2.90E-03 | 0.018379 | 0.56757 | H200010713 | OSTF1 | osteoclast stimulating factor 1 | 26578 |
| 19056 | -2.2887 | 0.481513 | 6.72E-03 | 0.028043 | 0.567756 | H200020378 | EBF3 | early B-cell factor 3 | 253738 |
| 14997 | -1.9954 | 0.141998 | 1.55E-02 | 0.044836 | 0.56778 | H200017624 | CES2 | carboxylesterase 2 | 8824 |
| 20261 | -2.1624 | 0.360881 | 9.64E-03 | 0.034624 | 0.567802 | H200014464 | TIMP4 | TIMP metallopeptidase inhibitor 4 | 7079 |
| 4728 | -2.2049 | 0.33573 | 8.54E-03 | 0.032185 | 0.568258 | H200007409 | FOXH1 | forkhead box H1 | 8928 |
| 10308 | -2.5796 | 0.146454 | 3.00E-03 | 0.018549 | 0.56826 | H200011873 | SNAPC4 | small nuclear RNA activating complex, polypeptide 4, 190kDa | 6621 |
| 19517 | -3.7048 | 0.146783 | 2.00E-04 | 0.006328 | 0.568265 | H200020657 | NA | NA | - |
| 18131 | -2.4566 | 0.34592 | 4.19E-03 | 0.022136 | 0.568454 | H200014744 | NA | NA | - |
| 2009 | -2.8032 | 0.233868 | 1.65E-03 | 0.014047 | 0.56854 | H200008745 | TMEM63B | transmembrane protein 63B | 55362 |
| 19385 | -1.7906 | 0.32394 | 2.78E-02 | 0.062447 | 0.568732 | H200014245 | ABHD5 | abhydrolase domain containing 5 | 51099 |
| 11594 | -1.8216 | 0.380014 | 2.55E-02 | 0.059244 | 0.568761 | H200008178 | CYHR1 | cysteine/histidine-rich 1 | 50626 |
| 7098 | -1.8734 | 0.188885 | 2.20E-02 | 0.054284 | 0.568782 | H200011707 | DCAF8 | DDB1 and CUL4 associated factor 8 | 50717 |
| 16947 | -2.2529 | 0.200956 | 7.45E-03 | 0.030011 | 0.568831 | H200001860 | QRICH1 | glutamine-rich 1 | 54870 |
| 15692 | -2.8523 | 0.215242 | 1.46E-03 | 0.013131 | 0.56895 | H200007358 | GALR2 | galanin receptor 2 | 8811 |
| 17520 | -2.4617 | 0.292781 | 4.13E-03 | 0.022044 | 0.56931 | H200007257 | TRAPPC9 | trafficking protein particle complex 9 | 83696 |
| 2708 | -2.1862 | 0.306082 | 9.00E-03 | 0.033183 | 0.569591 | H200020128 | WASF2 | WAS protein family, member 2 | 10163 |
| 6037 | -2.6557 | 0.239103 | 2.44E-03 | 0.016909 | 0.56977 | H200004849 | ELAC1 | elaC homolog 1 (E. coli) | 55520 |
| 13066 | -2.0456 | 0.580807 | 1.34E-02 | 0.041307 | 0.569826 | H200012928 | BCL11A | B-cell CLL/lymphoma 11A (zinc finger protein) | 53335 |
| 18267 | -2.3044 | 0.359432 | 6.43E-03 | 0.027337 | 0.569976 | H200021204 | NLRP4 | NLR family, pyrin domain containing 4 | 147945 |
| 16271 | -2.7057 | 0.4009 | 2.13E-03 | 0.015941 | 0.570183 | H200013195 | NA | NA | - |
| 14785 | -2.0516 | 0.367542 | 1.32E-02 | 0.040821 | 0.570333 | H200007696 | NA | NA | 199725 |
| 18088 | -3.2926 | 0.224865 | 4.97E-04 | 0.008649 | 0.570348 | H200012530 | KIF26B | kinesin family member 26B | 55083 |
| 17103 | -1.8043 | 0.493913 | 2.68E-02 | 0.061076 | 0.570552 | H200009412 | NA | NA | - |
| 5434 | -1.9919 | 0.317178 | 1.57E-02 | 0.045014 | 0.570555 | H200019592 | NA | NA | - |
| 2396 | -3.1982 | 0.195257 | 6.18E-04 | 0.009361 | 0.57057 | H200005308 | PRODH | proline dehydrogenase (oxidase) 1 | 5625 |
| 8192 | -2.9267 | 0.243547 | 1.20E-03 | 0.012106 | 0.570582 | H200020031 | NA | NA | - |
| 14704 | -3.4398 | 0.108321 | 3.58E-04 | 0.007621 | 0.570628 | H200003594 | HECTD3 | HECT domain containing E3 ubiquitin protein ligase 3 | 79654 |
| 985 | -2.8316 | 0.210694 | 1.54E-03 | 0.013549 | 0.57065 | H200003532 | MCF2L | MCF.2 cell line derived transforming sequence-like | 23263 |
| 10484 | -2.0528 | 0.208389 | 1.32E-02 | 0.040744 | 0.570677 | H200020233 | IFT172 | intraflagellar transport 172 homolog (Chlamydomonas) | 26160 |
| 19691 | -2.6542 | 0.122916 | 2.45E-03 | 0.01695 | 0.570779 | H200007428 | SAFB2 | scaffold attachment factor B2 | 9667 |
| 18705 | -2.0033 | 0.58244 | 1.52E-02 | 0.044273 | 0.570811 | H200001360 | KLF13 | Kruppel-like factor 13 | 51621 |
| 13380 | -2.431 | 0.280601 | 4.49E-03 | 0.022685 | 0.57085 | H200006065 | MTM1 | myotubularin 1 | 4534 |
| 18719 | -2.1948 | 0.300957 | 8.78E-03 | 0.03267 | 0.57136 | H200002120 | PCLO | piccolo presynaptic cytomatrix protein | 27445 |
| 15385 | -2.612 | 0.277122 | 2.74E-03 | 0.0178 | 0.571562 | H200014631 | TERF1 | telomeric repeat binding factor (NIMA-interacting) 1 | 7013 |
| 15118 | -1.9028 | 0.395579 | 2.02E-02 | 0.0519 | 0.571628 | H200001765 | APLNR | apelin receptor | 187 |
| 12922 | -3.122 | 0.172118 | 7.39E-04 | 0.009945 | 0.571668 | H200006088 | NAGA | N-acetylgalactosaminidase, alpha- | 4668 |
| 16188 | -2.5409 | 0.289089 | 3.32E-03 | 0.019619 | 0.572226 | H200009353 | TMEM134 | transmembrane protein 134 | 80194 |
| 5034 | -1.9343 | 0.323719 | 1.85E-02 | 0.049352 | 0.57226 | H200000592 | PRM2 | protamine 2 | 5620 |
| 11934 | -1.7819 | 0.246937 | 2.86E-02 | 0.063323 | 0.572277 | H200002443 | CXXC5 | CXXC finger protein 5 | 51523 |
| 3083 | -2.398 | 0.467668 | 4.92E-03 | 0.023817 | 0.572352 | H200016049 | FGF22 | fibroblast growth factor 22 | 27006 |
| 3711 | -2.4092 | 0.241885 | 4.78E-03 | 0.023403 | 0.572575 | H200002451 | TTC38 | tetratricopeptide repeat domain 38 | 55020 |
| 16559 | -2.2845 | 0.242257 | 6.80E-03 | 0.02825 | 0.57268 | H200005013 | MYLPF | myosin light chain, phosphorylatable, fast skeletal muscle | 29895 |
| 6333 | -1.9466 | 0.387067 | 1.78E-02 | 0.04839 | 0.572756 | H200018909 | EPS15L1 | epidermal growth factor receptor pathway substrate 15-like 1 | 58513 |
| 17436 | -2.0938 | 0.720354 | 1.17E-02 | 0.038372 | 0.572947 | H200003125 | SMOC2 | SPARC related modular calcium binding 2 | 64094 |
| 11702 | -2.5856 | 0.296325 | 2.94E-03 | 0.01846 | 0.572974 | H200013166 | FAM161B | family with sequence similarity 161, member B | 145483 |
| 5767 | -2.6073 | 0.292164 | 2.77E-03 | 0.017957 | 0.573039 | H200013376 | SLC8A2 | solute carrier family 8 (sodium/calcium exchanger), member 2 | 6543 |
| 11601 | -2.1262 | 0.359344 | 1.07E-02 | 0.036682 | 0.573064 | H200008552 | CC2D1A | coiled-coil and C2 domain containing 1A | 54862 |
| 1027 | -2.4435 | 0.257447 | 4.34E-03 | 0.022348 | 0.5732 | H200005456 | ZC3H10 | zinc finger CCCH-type containing 10 | 84872 |
| 7275 | -2.2564 | 0.331598 | 7.37E-03 | 0.029847 | 0.573216 | H200020085 | WFIKKN1 | WAP, follistatin/kazal, immunoglobulin, kunitz and netrin domain containing 1 | 117166 |
| 19096 | -2.5382 | 0.242648 | 3.35E-03 | 0.019638 | 0.573476 | H200000559 | SLC15A1 | solute carrier family 15 (oligopeptide transporter), member 1 | 6564 |
| 20656 | -2.0812 | 0.385358 | 1.21E-02 | 0.039035 | 0.573642 | H200014043 | EZR | ezrin | 7430 |
| 19767 | -2.5647 | 0.268282 | 3.12E-03 | 0.018959 | 0.573722 | H200010896 | C1RL | complement component 1, r subcomponent-like | 51279 |
| 17890 | -2.173 | 0.915596 | 9.34E-03 | 0.033963 | 0.57378 | H200003338 | SAMHD1 | SAM domain and HD domain 1 | 25939 |
| 10720 | -2.0381 | 0.238432 | 1.37E-02 | 0.041818 | 0.57397 | H200009878 | MAP3K1 | mitogen-activated protein kinase kinase kinase 1, E3 ubiquitin protein ligase | 4214 |
| 15477 | -2.2072 | 0.338035 | 8.48E-03 | 0.032081 | 0.57406 | H200018859 | MTHFS | 5,10-methenyltetrahydrofolate synthetase (5-formyltetrahydrofolate cyclo-ligase) | 10588 |
| 21324 | -2.7794 | 0.175353 | 1.75E-03 | 0.014464 | 0.574277 | H200003361 | STAT5B | signal transducer and activator of transcription 5B | 6777 |
| 6824 | -1.9902 | 0.38291 | 1.58E-02 | 0.045068 | 0.57433 | H200020186 | NA | NA | - |
| 12593 | -2.1062 | 0.373713 | 1.13E-02 | 0.037746 | 0.5744 | H200012364 | NA | NA | - |
| 10224 | -2.0495 | 0.409207 | 1.33E-02 | 0.040923 | 0.574453 | H200008025 | PYGO2 | pygopus homolog 2 (Drosophila) | 90780 |
| 14198 | -2.1821 | 0.354874 | 9.10E-03 | 0.033441 | 0.574507 | H200001493 | UBE2J2 | ubiquitin-conjugating enzyme E2, J2 | 118424 |
| 1609 | -2.055 | 0.353223 | 1.31E-02 | 0.040613 | 0.574702 | H200011607 | OSGEP | O-sialoglycoprotein endopeptidase | 55644 |
| 2400 | -3.8774 | 0.148481 | 1.43E-04 | 0.005857 | 0.574718 | H200005640 | OGFR | opioid growth factor receptor | 11054 |
| 20100 | -2.6448 | 0.212135 | 2.51E-03 | 0.017102 | 0.574789 | H200005724 | TMEM186 | transmembrane protein 186 | 25880 |
| 17402 | -2.5963 | 0.231072 | 2.86E-03 | 0.018254 | 0.574793 | H200001581 | SV2B | synaptic vesicle glycoprotein 2B | 9899 |
| 9460 | -3.3678 | 0.166506 | 4.19E-04 | 0.008048 | 0.575296 | H200015233 | C19orf73 | chromosome 19 open reading frame 73 | 55150 |
| 9774 | -2.9513 | 0.058852 | 1.12E-03 | 0.011725 | 0.575314 | H200008654 | NOL6 | nucleolar protein 6 (RNA-associated) | 65083 |
| 16416 | -3.5059 | 0.216813 | 3.08E-04 | 0.007429 | 0.575356 | H200020041 | MB21D1 | Mab-21 domain containing 1 | 115004 |
| 2804 | -3.4081 | 0.286748 | 3.80E-04 | 0.007752 | 0.575379 | H200002755 | FAM89B | family with sequence similarity 89, member B | 23625 |
| 831 | -1.7893 | 0.313225 | 2.80E-02 | 0.062561 | 0.575424 | H200017747 | NA | NA | - |
| 12859 | -3.8949 | 0.146227 | 1.38E-04 | 0.005806 | 0.575449 | H200003066 | WDR45 | WD repeat domain 45 | 11152 |
| 13559 | -2.0057 | 0.454087 | 1.51E-02 | 0.044017 | 0.575549 | H200014751 | NA | NA | - |
| 7796 | -3.5118 | 0.1502 | 3.04E-04 | 0.007429 | 0.575763 | H200001363 | GRIA1 | glutamate receptor, ionotropic, AMPA 1 | 2890 |
| 18253 | -2.1003 | 0.231256 | 1.15E-02 | 0.038062 | 0.576075 | H200020468 | TPTE2 | transmembrane phosphoinositide 3-phosphatase and tensin homolog 2 | 93492 |
| 8487 | -2.2029 | 0.324855 | 8.58E-03 | 0.032301 | 0.576279 | H200012342 | NA | NA | - |
| 3413 | -2.6096 | 0.272423 | 2.76E-03 | 0.0179 | 0.576521 | H200010230 | NA | NA | - |
| 3703 | -2.2805 | 0.313988 | 6.88E-03 | 0.02845 | 0.576599 | H200002071 | VNN1 | vanin 1 | 8876 |
| 15428 | -1.9131 | 0.613158 | 1.96E-02 | 0.051069 | 0.576627 | H200016561 | PAG1 | phosphoprotein associated with glycosphingolipid microdomains 1 | 55824 |
| 7756 | -2.9406 | 0.209292 | 1.16E-03 | 0.011848 | 0.576642 | H200021028 | NA | NA | - |
| 3691 | -2.1173 | 0.328613 | 1.10E-02 | 0.037142 | 0.577023 | H200001643 | TRAF4 | TNF receptor-associated factor 4 | 9618 |
| 8586 | -3.0393 | 0.081925 | 9.03E-04 | 0.010685 | 0.577403 | H200017216 | NR1D1 | nuclear receptor subfamily 1, group D, member 1 | 9572 |
| 10328 | -4.3141 | 0.113307 | 6.75E-05 | 0.004858 | 0.577487 | H200012965 | SUSD2 | sushi domain containing 2 | 56241 |
| 11635 | -1.9264 | 0.384361 | 1.89E-02 | 0.049925 | 0.577634 | H200010096 | REPS1 | RALBP1 associated Eps domain containing 1 | 85021 |
| 20128 | -2.6642 | 0.1412 | 2.38E-03 | 0.016739 | 0.577661 | H200007244 | MFSD10 | major facilitator superfamily domain containing 10 | 10227 |
| 21133 | -2.2386 | 0.40086 | 7.76E-03 | 0.030653 | 0.577665 | H200015670 | NA | NA | - |
| 2753 | -2.539 | 0.221832 | 3.34E-03 | 0.019619 | 0.577731 | H200000445 | NA | NA | - |
| 13694 | -2.3905 | 0.205595 | 5.03E-03 | 0.024081 | 0.578206 | H200021193 | ATP6V0E2 | ATPase, H+ transporting V0 subunit e2 | 155066 |
| 13196 | -2.65 | 0.213804 | 2.47E-03 | 0.017018 | 0.578288 | H200019032 | NA | NA | - |
| 1856 | -2.5976 | 0.2673 | 2.85E-03 | 0.018251 | 0.578341 | H200001223 | NA | NA | 57183 |
| 21660 | -2.2709 | 0.304883 | 7.08E-03 | 0.029009 | 0.578465 | H200019321 | C10orf71 | chromosome 10 open reading frame 71 | 118461 |
| 5030 | -1.8763 | 0.32553 | 2.18E-02 | 0.054055 | 0.578759 | H200000260 | PLA2G1B | phospholipase A2, group IB (pancreas) | 5319 |
| 17332 | -1.8745 | 0.286643 | 2.19E-02 | 0.054174 | 0.578884 | H200020118 | SF3A1 | splicing factor 3a, subunit 1, 120kDa | 10291 |
| 127 | -2.3127 | 0.308585 | 6.28E-03 | 0.027011 | 0.578993 | H200005872 | PTPN4 | protein tyrosine phosphatase, non-receptor type 4 (megakaryocyte) | 5775 |
| 4889 | -2.02 | 0.323399 | 1.45E-02 | 0.043116 | 0.579032 | H200015311 | NA | NA | - |
| 20841 | -2.4177 | 0.358544 | 4.66E-03 | 0.023141 | 0.57908 | H200001942 | CIB1 | calcium and integrin binding 1 (calmyrin) | 10519 |
| 14961 | -2.4635 | 0.267919 | 4.11E-03 | 0.02199 | 0.579191 | H200016056 | CHRNG | cholinergic receptor, nicotinic, gamma (muscle) | 1146 |
| 3278 | -2.8932 | 0.223763 | 1.30E-03 | 0.012515 | 0.579274 | H200003776 | GFOD2 | glucose-fructose oxidoreductase domain containing 2 | 81577 |
| 19685 | -2.0482 | 0.355025 | 1.34E-02 | 0.041051 | 0.579646 | H200007072 | AMPD3 | adenosine monophosphate deaminase 3 | 272 |
| 11024 | -2.0298 | 0.328543 | 1.41E-02 | 0.042389 | 0.579672 | H200002455 | C1D | C1D nuclear receptor corepressor | 10438 |
| 20196 | -2.319 | 0.270535 | 6.17E-03 | 0.026748 | 0.579798 | H200010718 | G0S2 | G0/G1switch 2 | 50486 |
| 6111 | -2.0916 | 0.425254 | 1.18E-02 | 0.038497 | 0.57988 | H200008293 | NA | NA | - |
| 6755 | -2.9391 | 0.209042 | 1.16E-03 | 0.011872 | 0.580005 | H200016808 | NA | NA | - |
| 352 | -2.9085 | 0.129106 | 1.25E-03 | 0.012277 | 0.580258 | H200016518 | NA | NA | - |
| 3727 | -3.3987 | 0.200625 | 3.89E-04 | 0.00776 | 0.580267 | H200003211 | NAT6 | N-acetyltransferase 6 (GCN5-related) | 24142 |
| 15290 | -2.8591 | 0.225754 | 1.43E-03 | 0.013052 | 0.580914 | H200010077 | NF2 | neurofibromin 2 (merlin) | 4771 |
| 3463 | -2.545 | 0.240941 | 3.29E-03 | 0.01954 | 0.580951 | H200012534 | ASB6 | ankyrin repeat and SOCS box containing 6 | 140459 |
| 4356 | -1.8743 | 0.131316 | 2.19E-02 | 0.054174 | 0.581049 | H200011624 | C14orf2 | chromosome 14 open reading frame 2 | 9556 |
| 13800 | -1.9614 | 0.544552 | 1.71E-02 | 0.047358 | 0.581091 | H200004224 | TESPA1 | thymocyte expressed, positive selection associated 1 | 9840 |
| 4932 | -2.5561 | 0.231969 | 3.19E-03 | 0.019256 | 0.581104 | H200017241 | ATG9B | autophagy related 9B | 285973 |
| 3636 | -3.0154 | 0.114896 | 9.59E-04 | 0.010959 | 0.581471 | H200020852 | LOC219688 | uncharacterized LOC219688 | 219688 |
| 16475 | -2.5179 | 0.221537 | 3.54E-03 | 0.020321 | 0.58169 | H200001165 | IGSF21 | immunoglobin superfamily, member 21 | 84966 |
| 20401 | -4.0224 | 0.13854 | 1.11E-04 | 0.005439 | 0.581821 | H200000333 | TRIM26 | tripartite motif containing 26 | 7726 |
| 9931 | -3.2076 | 0.229449 | 6.04E-04 | 0.009326 | 0.581899 | H200015940 | USF1 | upstream transcription factor 1 | 7391 |
| 4222 | -2.8347 | 0.215603 | 1.52E-03 | 0.01351 | 0.58193 | H200005188 | ABCC10 | ATP-binding cassette, sub-family C (CFTR/MRP), member 10 | 89845 |
| 5166 | -2.5139 | 0.198979 | 3.58E-03 | 0.020435 | 0.581974 | H200006720 | PCDH1 | protocadherin 1 | 5097 |
| 11490 | -1.8176 | 0.362041 | 2.58E-02 | 0.059686 | 0.581981 | H200003238 | MED24 | mediator complex subunit 24 | 9862 |
| 19679 | -2.2396 | 0.273707 | 7.74E-03 | 0.030653 | 0.582043 | H200006716 | FBLN1 | fibulin 1 | 2192 |
| 12614 | -3.0198 | 0.201076 | 9.49E-04 | 0.0109 | 0.58227 | H200013178 | ATOH8 | atonal homolog 8 (Drosophila) | 84913 |
| 19071 | -2.6444 | 0.194684 | 2.51E-03 | 0.017102 | 0.582283 | H200021144 | CCDC111 | coiled-coil domain containing 111 | 201973 |
| 3929 | -3.4093 | 0.165084 | 3.80E-04 | 0.007752 | 0.582296 | H200013019 | NA | NA | - |
| 13408 | -2.7928 | 0.236886 | 1.70E-03 | 0.014246 | 0.582424 | H200007537 | CASZ1 | castor zinc finger 1 | 54897 |
| 18783 | -2.0623 | 0.223531 | 1.28E-02 | 0.040103 | 0.582442 | H200005558 | CTNS | cystinosin, lysosomal cystine transporter | 1497 |
| 13325 | -2.0134 | 0.368942 | 1.47E-02 | 0.043598 | 0.582443 | H200003707 | THAP3 | THAP domain containing, apoptosis associated protein 3 | 90326 |
| 17656 | -1.8953 | 0.361518 | 2.06E-02 | 0.052375 | 0.582461 | H200013717 | MLYCD | malonyl-CoA decarboxylase | 23417 |
| 19146 | -2.993 | 0.181147 | 1.01E-03 | 0.011207 | 0.582529 | H200003147 | FAM134A | family with sequence similarity 134, member A | 79137 |
| 10174 | -2.013 | 0.366669 | 1.48E-02 | 0.043598 | 0.582537 | H200005721 | TIPIN | TIMELESS interacting protein | 54962 |
| 8434 | -3.0615 | 0.184524 | 8.56E-04 | 0.010432 | 0.582585 | H200009996 | PITPNM3 | PITPNM family member 3 | 83394 |
| 2253 | -2.1685 | 0.289442 | 9.47E-03 | 0.034232 | 0.583001 | H200020193 | SLC1A7 | solute carrier family 1 (glutamate transporter), member 7 | 6512 |
| 10827 | -1.9639 | 0.465127 | 1.70E-02 | 0.047134 | 0.583043 | H200015144 | NDUFS7 | NADH dehydrogenase (ubiquinone) Fe-S protein 7, 20kDa (NADH-coenzyme Q reductase) | 374291 |
| 11145 | -2.1666 | 0.228731 | 9.53E-03 | 0.03439 | 0.583115 | H200008457 | SF1 | splicing factor 1 | 7536 |
| 14932 | -2.0867 | 0.356337 | 1.20E-02 | 0.038753 | 0.583221 | H200014566 | NA | NA | - |
| 21653 | -2.885 | 0.175211 | 1.33E-03 | 0.012562 | 0.583404 | H200018947 | ABHD8 | abhydrolase domain containing 8 | 79575 |
| 15217 | -1.8539 | 0.456335 | 2.32E-02 | 0.056137 | 0.58341 | H200006651 | MYOM2 | myomesin 2 | 9172 |
| 7425 | -1.9857 | 0.410413 | 1.60E-02 | 0.045424 | 0.583425 | H200005418 | FBP2 | fructose-1,6-bisphosphatase 2 | 8789 |
| 6628 | -3.4348 | 0.186703 | 3.61E-04 | 0.007651 | 0.583427 | H200010734 | CACNA1A | calcium channel, voltage-dependent, P/Q type, alpha 1A subunit | 773 |
| 4502 | -2.1442 | 0.30631 | 1.01E-02 | 0.035529 | 0.583697 | H200018488 | NA | NA | - |
| 6990 | -2.075 | 0.324096 | 1.24E-02 | 0.039424 | 0.584098 | H200006435 | MTSS1 | metastasis suppressor 1 | 9788 |
| 11975 | -2.27 | 0.304761 | 7.10E-03 | 0.029051 | 0.584157 | H200004361 | AQP2 | aquaporin 2 (collecting duct) | 359 |
| 3401 | -2.0207 | 0.375514 | 1.44E-02 | 0.043072 | 0.584517 | H200009802 | NA | NA | - |
| 3737 | -1.9364 | 0.333575 | 1.84E-02 | 0.049255 | 0.584749 | H200003899 | MLST8 | MTOR associated protein, LST8 homolog (S. cerevisiae) | 64223 |
| 8739 | -2.0843 | 0.223314 | 1.20E-02 | 0.038927 | 0.584779 | H200002889 | ABHD6 | abhydrolase domain containing 6 | 57406 |
| 5681 | -2.6109 | 0.136652 | 2.75E-03 | 0.017862 | 0.584783 | H200009504 | NA | NA | 4328 |
| 953 | -2.4841 | 0.241254 | 3.88E-03 | 0.021301 | 0.584971 | H200002012 | ARPC1B | actin related protein 2/3 complex, subunit 1B, 41kDa | 10095 |
| 17853 | -2.322 | 0.222715 | 6.12E-03 | 0.02668 | 0.585344 | H200001468 | MAZ | MYC-associated zinc finger protein (purine-binding transcription factor) | 4150 |
| 18067 | -2.6444 | 0.248625 | 2.51E-03 | 0.017102 | 0.585411 | H200011704 | APOC4 | apolipoprotein C-IV | 346 |
| 16463 | -1.9713 | 0.286926 | 1.66E-02 | 0.046542 | 0.585747 | H200000453 | INHA | inhibin, alpha | 3623 |
| 1375 | -1.8068 | 0.359184 | 2.66E-02 | 0.060742 | 0.585751 | H200000279 | SFTPC | surfactant protein C | 6440 |
| 7731 | -2.2913 | 0.167872 | 6.67E-03 | 0.027932 | 0.585768 | H200019882 | CLK3 | CDC-like kinase 3 | 1198 |
| 6291 | -2.4573 | 0.230926 | 4.18E-03 | 0.022124 | 0.585849 | H200016701 | SLC16A8 | solute carrier family 16, member 8 (monocarboxylic acid transporter 3) | 23539 |
| 20673 | -2.1646 | 0.283256 | 9.58E-03 | 0.034504 | 0.586194 | H200015135 | MED12 | mediator complex subunit 12 | 9968 |
| 6120 | -1.9599 | 0.270305 | 1.72E-02 | 0.047395 | 0.586251 | H200008679 | SLC52A3 | solute carrier family 52, riboflavin transporter, member 3 | 113278 |
| 21073 | -2.4402 | 0.250302 | 4.38E-03 | 0.022409 | 0.586436 | H200012962 | NUP210L | nucleoporin 210kDa-like | 91181 |
| 8846 | -2.705 | 0.197651 | 2.14E-03 | 0.015941 | 0.586451 | H200007859 | BOLL | bol, boule-like (Drosophila) | 66037 |
| 14706 | -2.7232 | 0.202829 | 2.04E-03 | 0.015612 | 0.586678 | H200003902 | ZNF76 | zinc finger protein 76 | 7629 |
| 4668 | -2.3249 | 0.187258 | 6.07E-03 | 0.026632 | 0.58669 | H200004701 | GEMIN8 | gem (nuclear organelle) associated protein 8 | 54960 |
| 3061 | -2.4201 | 0.153536 | 4.63E-03 | 0.023035 | 0.586921 | H200015217 | CEACAM5 | carcinoembryonic antigen-related cell adhesion molecule 5 | 1048 |
| 2433 | -3.1575 | 0.182031 | 6.78E-04 | 0.009673 | 0.586926 | H200007178 | MYLK2 | myosin light chain kinase 2 | 85366 |
| 19711 | -2.4013 | 0.241157 | 4.88E-03 | 0.023671 | 0.587076 | H200008236 | NA | NA | 200213 |
| 3153 | -1.9814 | 0.387332 | 1.61E-02 | 0.045797 | 0.587281 | H200019445 | NA | NA | - |
| 306 | -2.015 | 0.355223 | 1.47E-02 | 0.043498 | 0.58732 | H200014546 | PLCH1 | phospholipase C, eta 1 | 23007 |
| 12025 | -3.3967 | 0.115219 | 3.91E-04 | 0.007781 | 0.58741 | H200006949 | THAP11 | THAP domain containing 11 | 57215 |
| 5609 | -3.2798 | 0.132374 | 5.12E-04 | 0.00873 | 0.587611 | H200006084 | UBE2N | ubiquitin-conjugating enzyme E2N | 7334 |
| 17043 | -2.5815 | 0.251979 | 2.98E-03 | 0.018531 | 0.587651 | H200006420 | ALG12 | ALG12, alpha-1,6-mannosyltransferase | 79087 |
| 897 | -2.492 | 0.176099 | 3.79E-03 | 0.021017 | 0.5888 | H200021095 | FAM151A | family with sequence similarity 151, member A | 338094 |
| 17781 | -3.1243 | 0.176939 | 7.35E-04 | 0.009931 | 0.588838 | H200019471 | DIAPH1 | diaphanous homolog 1 (Drosophila) | 1729 |
| 813 | -1.803 | 0.209026 | 2.69E-02 | 0.061217 | 0.588841 | H200016963 | PCIF1 | PDX1 C-terminal inhibiting factor 1 | 63935 |
| 1319 | -2.026 | 0.145128 | 1.42E-02 | 0.042699 | 0.588851 | H200019184 | DGUOK | deoxyguanosine kinase | 1716 |
| 8938 | -2.3302 | 0.184386 | 5.98E-03 | 0.026365 | 0.588897 | H200012371 | CACNA1H | calcium channel, voltage-dependent, T type, alpha 1H subunit | 8912 |
| 479 | -2.7212 | 0.180359 | 2.05E-03 | 0.015632 | 0.589048 | H200001027 | BLCAP | bladder cancer associated protein | 10904 |
| 21298 | -3.2898 | 0.181716 | 5.01E-04 | 0.008671 | 0.589317 | H200002197 | AGTRAP | angiotensin II receptor-associated protein | 57085 |
| 17419 | -1.7988 | 0.375178 | 2.72E-02 | 0.061627 | 0.589416 | H200002347 | NA | NA | - |
| 15705 | -2.0305 | 0.274971 | 1.40E-02 | 0.042377 | 0.589485 | H200008088 | DNAJC4 | DnaJ (Hsp40) homolog, subfamily C, member 4 | 3338 |
| 12869 | -2.2763 | 0.227438 | 6.96E-03 | 0.028687 | 0.589594 | H200003470 | CCDC130 | coiled-coil domain containing 130 | 81576 |
| 3522 | -2.4431 | 0.235561 | 4.35E-03 | 0.022348 | 0.589749 | H200015508 | NA | NA | - |
| 5973 | -2.6576 | 0.304296 | 2.43E-03 | 0.016906 | 0.589776 | H200001809 | RRAS | related RAS viral (r-ras) oncogene homolog | 6237 |
| 7038 | -1.8029 | 0.357226 | 2.69E-02 | 0.061217 | 0.590136 | H200008715 | NA | NA | - |
| 21559 | -2.0982 | 0.410566 | 1.16E-02 | 0.038171 | 0.590425 | H200014411 | NA | NA | - |
| 9837 | -2.4792 | 1.64198 | 3.93E-03 | 0.021443 | 0.590459 | H200011688 | HLA-F | major histocompatibility complex, class I, F | 3134 |
| 330 | -2.9464 | 0.217549 | 1.14E-03 | 0.011769 | 0.590665 | H200015686 | CALML4 | calmodulin-like 4 | 91860 |
| 10319 | -2.1921 | 0.358906 | 8.85E-03 | 0.03288 | 0.590679 | H200012579 | TTC39A | tetratricopeptide repeat domain 39A | 22996 |
| 11916 | -3.2307 | 0.237035 | 5.75E-04 | 0.009191 | 0.590714 | H200001659 | APOBEC3C | apolipoprotein B mRNA editing enzyme, catalytic polypeptide-like 3C | 27350 |
| 15999 | -3.2118 | 0.311885 | 5.99E-04 | 0.009326 | 0.590723 | H200000275 | CYTH1 | cytohesin 1 | 9267 |
| 8511 | -2.0249 | 0.437519 | 1.43E-02 | 0.042773 | 0.590854 | H200013482 | DUSP9 | dual specificity phosphatase 9 | 1852 |
| 20753 | -2.4421 | 0.224387 | 4.36E-03 | 0.022351 | 0.590938 | H200019357 | TSSK1B | testis-specific serine kinase 1B | 83942 |
| 17093 | -2.6783 | 0.226661 | 2.29E-03 | 0.0164 | 0.591062 | H200008724 | GNG8 | guanine nucleotide binding protein (G protein), gamma 8 | 94235 |
| 4888 | -1.824 | 0.254247 | 2.53E-02 | 0.058967 | 0.591079 | H200015009 | YME1L1 | YME1-like 1 (S. cerevisiae) | 10730 |
| 18167 | -1.9236 | 0.365783 | 1.90E-02 | 0.050119 | 0.591254 | H200016312 | UTRN | utrophin | 7402 |
| 18566 | -2.2581 | 0.125316 | 7.34E-03 | 0.02977 | 0.591277 | H200015349 | PCYT2 | phosphate cytidylyltransferase 2, ethanolamine | 5833 |
| 5041 | -2.8876 | 0.178128 | 1.32E-03 | 0.012529 | 0.591346 | H200000966 | TSSC1 | tumor suppressing subtransferable candidate 1 | 7260 |
| 5696 | -2.1014 | 0.314158 | 1.15E-02 | 0.037986 | 0.59149 | H200009962 | NA | NA | - |
| 919 | -2.3224 | 0.212385 | 6.11E-03 | 0.02668 | 0.591648 | H200000184 | PFKFB1 | 6-phosphofructo-2-kinase/fructose-2,6-biphosphatase 1 | 5207 |
| 7218 | -2.4554 | 0.260656 | 4.20E-03 | 0.022146 | 0.591867 | H200017407 | ANGPT4 | angiopoietin 4 | 51378 |
| 20190 | -2.0729 | 0.216926 | 1.24E-02 | 0.0395 | 0.592211 | H200010646 | PMF1 | polyamine-modulated factor 1 | 11243 |
| 10370 | -2.3274 | 0.256996 | 6.02E-03 | 0.026514 | 0.59249 | H200014889 | ADAMTSL2 | ADAMTS-like 2 | 9719 |
| 12908 | -2.644 | 0.191213 | 2.51E-03 | 0.017104 | 0.592606 | H200005352 | TOR2A | torsin family 2, member A | 27433 |
| 1700 | -2.4888 | 0.259748 | 3.83E-03 | 0.021104 | 0.592649 | H200015817 | STAT5B | signal transducer and activator of transcription 5B | 6777 |
| 14351 | -2.8748 | 0.24454 | 1.37E-03 | 0.012807 | 0.592656 | H200008731 | LRRC61 | leucine rich repeat containing 61 | 65999 |
| 2862 | -2.0718 | 0.388509 | 1.25E-02 | 0.039551 | 0.592689 | H200005723 | LSM7 | LSM7 homolog, U6 small nuclear RNA associated (S. cerevisiae) | 51690 |
| 3754 | -1.9562 | 0.194451 | 1.74E-02 | 0.047762 | 0.592757 | H200004665 | SFXN2 | sideroflexin 2 | 118980 |
| 13623 | -3.0268 | 0.198033 | 9.32E-04 | 0.010788 | 0.592935 | H200017791 | CTDSP1 | CTD (carboxy-terminal domain, RNA polymerase II, polypeptide A) small phosphatase 1 | 58190 |
| 13911 | -2.7652 | 0.195446 | 1.82E-03 | 0.014755 | 0.593087 | H200009538 | NA | NA | - |
| 17092 | -2.8608 | 0.260536 | 1.42E-03 | 0.013034 | 0.593145 | H200008718 | INGX | inhibitor of growth family, X-linked, pseudogene | 27160 |
| 10933 | -3.0297 | 0.165261 | 9.26E-04 | 0.01078 | 0.593282 | H200020108 | CCPG1 | cell cycle progression 1 | 9236 |
| 8900 | -1.9546 | 0.367728 | 1.74E-02 | 0.047858 | 0.593532 | H200010495 | KNDC1 | kinase non-catalytic C-lobe domain (KIND) containing 1 | 85442 |
| 15838 | -1.893 | 0.281703 | 2.08E-02 | 0.05261 | 0.593575 | H200014222 | NA | NA | - |
| 19588 | -2.0257 | 0.380397 | 1.42E-02 | 0.042715 | 0.59359 | H200002506 | ABCA6 | ATP-binding cassette, sub-family A (ABC1), member 6 | 23460 |
| 21097 | -2.2292 | 0.272276 | 7.98E-03 | 0.031081 | 0.593599 | H200014102 | PAX2 | paired box 2 | 5076 |
| 19899 | -2.8624 | 0.23893 | 1.42E-03 | 0.012998 | 0.593693 | H200017308 | SNX17 | sorting nexin 17 | 9784 |
| 2616 | -2.9973 | 0.119887 | 1.00E-03 | 0.011181 | 0.593804 | H200015900 | CREB3L3 | cAMP responsive element binding protein 3-like 3 | 84699 |
| 14011 | -1.8461 | 0.225441 | 2.38E-02 | 0.056872 | 0.593847 | H200014146 | UTF1 | undifferentiated embryonic cell transcription factor 1 | 8433 |
| 13261 | -2.0897 | 0.273157 | 1.18E-02 | 0.038603 | 0.593896 | H200000667 | EDDM3A | epididymal protein 3A | 10876 |
| 11406 | -1.8293 | 0.586576 | 2.49E-02 | 0.058396 | 0.594261 | H200020671 | NA | NA | - |
| 21334 | -3.0909 | 0.150816 | 7.97E-04 | 0.010222 | 0.594321 | H200003765 | ZNF395 | zinc finger protein 395 | 55893 |
| 7969 | -2.0503 | 0.332219 | 1.33E-02 | 0.040882 | 0.594353 | H200009693 | LOC91450 | uncharacterized LOC91450 | 91450 |
| 20786 | -1.8127 | 0.307381 | 2.62E-02 | 0.060181 | 0.594444 | H200021233 | NA | NA | - |
| 5443 | -2.1865 | 0.311311 | 8.99E-03 | 0.033168 | 0.594839 | H200019990 | CAMK2N2 | calcium/calmodulin-dependent protein kinase II inhibitor 2 | 94032 |
| 20581 | -2.074 | 0.262655 | 1.24E-02 | 0.03943 | 0.594929 | H200010189 | USP10 | ubiquitin specific peptidase 10 | 9100 |
| 13622 | -2.51 | 0.198261 | 3.61E-03 | 0.020512 | 0.594959 | H200017773 | GTPBP1 | GTP binding protein 1 | 9567 |
| 11238 | -3.1675 | 0.176801 | 6.65E-04 | 0.009584 | 0.594984 | H200012691 | SYT7 | synaptotagmin VII | 9066 |
| 15498 | -2.2998 | 0.28625 | 6.51E-03 | 0.027537 | 0.595147 | H200019957 | ABCC12 | ATP-binding cassette, sub-family C (CFTR/MRP), member 12 | 94160 |
| 5424 | -3.1045 | 0.104923 | 7.71E-04 | 0.010006 | 0.595173 | H200018904 | GIT1 | G protein-coupled receptor kinase interacting ArfGAP 1 | 28964 |
| 17404 | -2.451 | 0.241266 | 4.25E-03 | 0.022206 | 0.595372 | H200001605 | EPAS1 | endothelial PAS domain protein 1 | 2034 |
| 13212 | -2.3915 | 0.223776 | 5.01E-03 | 0.024061 | 0.595415 | H200019792 | DAXX | death-domain associated protein | 1616 |
| 18712 | -2.3209 | 0.213428 | 6.14E-03 | 0.02668 | 0.595469 | H200001740 | SLC41A3 | solute carrier family 41, member 3 | 54946 |
| 18135 | -1.9891 | 0.338877 | 1.58E-02 | 0.045187 | 0.595511 | H200014792 | MMP25 | matrix metallopeptidase 25 | 64386 |
| 15155 | -2.514 | 0.230567 | 3.58E-03 | 0.020435 | 0.595594 | H200003635 | GSTZ1 | glutathione S-transferase zeta 1 | 2954 |
| 10993 | -2.862 | 0.193109 | 1.42E-03 | 0.013005 | 0.595725 | H200001237 | BABAM1 | BRISC and BRCA1 A complex member 1 | 29086 |
| 14770 | -2.784 | 0.454829 | 1.73E-03 | 0.014343 | 0.595741 | H200006942 | KIAA0247 | KIAA0247 | 9766 |
| 10836 | -3.0289 | 0.136494 | 9.28E-04 | 0.01078 | 0.595828 | H200015530 | VPS4A | vacuolar protein sorting 4 homolog A (S. cerevisiae) | 27183 |
| 10189 | -3.7608 | 0.077044 | 1.79E-04 | 0.006117 | 0.595937 | H200006475 | ACD | adrenocortical dysplasia homolog (mouse) | 65057 |
| 15809 | -1.9605 | 0.270349 | 1.71E-02 | 0.047387 | 0.596174 | H200013028 | RPL39L | ribosomal protein L39-like | 116832 |
| 6104 | -1.8625 | 0.399389 | 2.27E-02 | 0.055235 | 0.59621 | H200007919 | TNIK | TRAF2 and NCK interacting kinase | 23043 |
| 4029 | -2.283 | 0.237501 | 6.83E-03 | 0.028347 | 0.596218 | H200017627 | TP53RK | TP53 regulating kinase | 112858 |
| 2697 | -3.1711 | 0.202767 | 6.60E-04 | 0.009552 | 0.596354 | H200019718 | GTPBP3 | GTP binding protein 3 (mitochondrial) | 84705 |
| 5067 | -2.632 | 0.217773 | 2.59E-03 | 0.017406 | 0.596394 | H200002130 | FBXL12 | F-box and leucine-rich repeat protein 12 | 54850 |
| 7178 | -2.2412 | 0.113137 | 7.70E-03 | 0.030606 | 0.596449 | H200015507 | GMPR2 | guanosine monophosphate reductase 2 | 51292 |
| 17838 | -2.4464 | 0.227467 | 4.31E-03 | 0.022318 | 0.596506 | H200000726 | CCNO | cyclin O | 10309 |
| 3681 | -2.1267 | 0.266568 | 1.07E-02 | 0.036674 | 0.596535 | H200001239 | MON1A | MON1 homolog A (yeast) | 84315 |
| 5097 | -2.1215 | 0.349234 | 1.08E-02 | 0.036916 | 0.59665 | H200003626 | CCDC22 | coiled-coil domain containing 22 | 28952 |
| 3365 | -2.6258 | 0.116878 | 2.64E-03 | 0.017531 | 0.596924 | H200007950 | BRSK2 | BR serine/threonine kinase 2 | 9024 |
| 19091 | -1.8457 | 0.500443 | 2.38E-02 | 0.056903 | 0.596979 | H200000493 | DSG3 | desmoglein 3 | 1830 |
| 16873 | -2.7256 | 0.145149 | 2.03E-03 | 0.0156 | 0.597119 | H200020141 | NA | NA | - |
| 5653 | -2.8975 | 0.160782 | 1.29E-03 | 0.012501 | 0.597165 | H200008032 | MGAT2 | mannosyl (alpha-1,6-)-glycoprotein beta-1,2-N-acetylglucosaminyltransferase | 4247 |
| 7998 | -2.6683 | 0.212713 | 2.36E-03 | 0.016639 | 0.597352 | H200010887 | SSPO | SCO-spondin homolog (Bos taurus) | 23145 |
| 3518 | -2.1657 | 0.249184 | 9.55E-03 | 0.034443 | 0.597408 | H200015176 | ZNF575 | zinc finger protein 575 | 284346 |
| 13448 | -1.812 | 0.577113 | 2.62E-02 | 0.060197 | 0.597528 | H200009437 | TNIP2 | TNFAIP3 interacting protein 2 | 79155 |
| 11581 | -2.1594 | 0.20729 | 9.72E-03 | 0.034686 | 0.597616 | H200007460 | PTCH1 | patched 1 | 5727 |
| 6629 | -2.177 | 0.21481 | 9.24E-03 | 0.033755 | 0.59792 | H200011036 | ZNF460 | zinc finger protein 460 | 10794 |
| 19213 | -3.903 | 0.106986 | 1.36E-04 | 0.005801 | 0.597927 | H200006217 | MRPL49 | mitochondrial ribosomal protein L49 | 740 |
| 13329 | -1.9492 | 0.306685 | 1.77E-02 | 0.048285 | 0.598034 | H200003755 | MED31 | mediator complex subunit 31 | 51003 |
| 2393 | -2.3452 | 0.263227 | 5.72E-03 | 0.025809 | 0.598123 | H200005278 | CLEC2L | C-type lectin domain family 2, member L | 154790 |
| 4435 | -2.5223 | 0.166595 | 3.50E-03 | 0.02018 | 0.59813 | H200015418 | PLEKHS1 | pleckstrin homology domain containing, family S member 1 | 79949 |
| 4703 | -1.8119 | 0.407614 | 2.62E-02 | 0.060197 | 0.598391 | H200006263 | CYP11A1 | cytochrome P450, family 11, subfamily A, polypeptide 1 | 1583 |
| 12763 | -2.0229 | 0.310888 | 1.43E-02 | 0.042936 | 0.598453 | H200020368 | NA | NA | - |
| 5956 | -2.3856 | 0.252514 | 5.10E-03 | 0.024263 | 0.598518 | H200000747 | PPBPP2 | pro-platelet basic protein pseudogene 2 | 10895 |
| 16660 | -2.1307 | 0.222643 | 1.05E-02 | 0.03637 | 0.598545 | H200009911 | BET1L | blocked early in transport 1 homolog (S. cerevisiae)-like | 51272 |
| 7847 | -2.4463 | 0.243768 | 4.31E-03 | 0.022318 | 0.598828 | H200003685 | NTRK3 | neurotrophic tyrosine kinase, receptor, type 3 | 4916 |
| 20819 | -2.2358 | 0.249839 | 7.83E-03 | 0.030734 | 0.598913 | H200000826 | USP5 | ubiquitin specific peptidase 5 (isopeptidase T) | 8078 |
| 14366 | -2.6446 | 0.229666 | 2.51E-03 | 0.017102 | 0.598914 | H200009473 | NA | NA | - |
| 18627 | -2.972 | 0.190668 | 1.07E-03 | 0.011465 | 0.599073 | H200018745 | NA | NA | - |
| 8940 | -2.2588 | 0.245733 | 7.33E-03 | 0.029761 | 0.599104 | H200012395 | NA | NA | - |
| 12733 | -2.5394 | 0.120135 | 3.34E-03 | 0.019619 | 0.599131 | H200018872 | IFRD2 | interferon-related developmental regulator 2 | 7866 |
| 7434 | -2.2563 | 0.251845 | 7.38E-03 | 0.029847 | 0.599163 | H200005804 | CDKN2B | cyclin-dependent kinase inhibitor 2B (p15, inhibits CDK4) | 1030 |
| 13931 | -2.5155 | 0.258961 | 3.56E-03 | 0.020385 | 0.59918 | H200010346 | SELE | selectin E | 6401 |
| 10241 | -2.0617 | 0.210338 | 1.28E-02 | 0.04014 | 0.599216 | H200008803 | NA | NA | - |
| 11033 | -2.0235 | 0.276739 | 1.43E-02 | 0.042908 | 0.599237 | H200003137 | GAREML | GRB2 associated, regulator of MAPK1-like | 150946 |
| 11388 | -2.4639 | 0.253614 | 4.10E-03 | 0.02199 | 0.599343 | H200019887 | PTH2 | parathyroid hormone 2 | 113091 |
| 15 | -2.5505 | 0.27314 | 3.24E-03 | 0.019442 | 0.599347 | H200000552 | CSF3R | colony stimulating factor 3 receptor (granulocyte) | 1441 |
| 6663 | -2.4508 | 0.222059 | 4.26E-03 | 0.022206 | 0.599582 | H200012580 | LRRC2 | leucine rich repeat containing 2 | 79442 |
| 10447 | -2.571 | 0.222727 | 3.07E-03 | 0.018815 | 0.599611 | H200018659 | NA | NA | - |
| 10393 | -2.6927 | 0.150922 | 2.21E-03 | 0.016167 | 0.599614 | H200016023 | NA | NA | - |
| 19044 | -2.2441 | 0.22434 | 7.64E-03 | 0.030457 | 0.599628 | H200019642 | SPATC1L | spermatogenesis and centriole associated 1-like | 84221 |
| 19058 | -2.6241 | 0.157219 | 2.65E-03 | 0.017573 | 0.599725 | H200020402 | SLC35C2 | solute carrier family 35, member C2 | 51006 |
| 10590 | -2.5213 | 0.215959 | 3.51E-03 | 0.020214 | 0.599792 | H200003774 | LOC150622 | uncharacterized LOC150622 | 150622 |
| 20625 | -2.2472 | 0.265012 | 7.57E-03 | 0.030322 | 0.599796 | H200012493 | RNF2 | ring finger protein 2 | 6045 |
| 11559 | -2.2251 | 0.368742 | 8.07E-03 | 0.031187 | 0.599805 | H200006344 | NA | NA | - |
| 19838 | -2.6642 | 0.193848 | 2.38E-03 | 0.016739 | 0.600286 | H200014310 | RAB3C | RAB3C, member RAS oncogene family | 115827 |
| 349 | -2.1466 | 0.267521 | 1.01E-02 | 0.035383 | 0.600317 | H200016488 | ADCY1 | adenylate cyclase 1 (brain) | 107 |
| 13939 | -2.1168 | 0.252189 | 1.10E-02 | 0.037171 | 0.600384 | H200010726 | E2F1 | E2F transcription factor 1 | 1869 |
| 13433 | -2.6319 | 0.184082 | 2.59E-03 | 0.017406 | 0.600425 | H200008695 | RNF215 | ring finger protein 215 | 200312 |
| 12958 | -1.9747 | 0.343695 | 1.65E-02 | 0.046284 | 0.600515 | H200007656 | ELF3 | E74-like factor 3 (ets domain transcription factor, epithelial-specific ) | 1999 |
| 20815 | -2.2285 | 0.223686 | 7.99E-03 | 0.031096 | 0.600655 | H200000778 | TBC1D13 | TBC1 domain family, member 13 | 54662 |
| 15403 | -1.9974 | 0.341201 | 1.54E-02 | 0.044762 | 0.600821 | H200015415 | NA | NA | - |
| 14878 | -2.876 | 0.154437 | 1.37E-03 | 0.012789 | 0.60087 | H200011930 | GRAMD2 | GRAM domain containing 2 | 196996 |
| 3265 | -2.7233 | 0.175873 | 2.04E-03 | 0.015612 | 0.600889 | H200003342 | TRDN | triadin | 10345 |
| 5154 | -2.2812 | 0.256653 | 6.86E-03 | 0.028411 | 0.601025 | H200006292 | SLC38A3 | solute carrier family 38, member 3 | 10991 |
| 16406 | -1.8562 | 0.392445 | 2.31E-02 | 0.055914 | 0.601045 | H200019637 | PDZD7 | PDZ domain containing 7 | 79955 |
| 17865 | -2.411 | 0.266135 | 4.75E-03 | 0.023357 | 0.601233 | H200001896 | NA | NA | 388494 |
| 2445 | -1.9049 | 0.118351 | 2.01E-02 | 0.051679 | 0.601262 | H200007890 | EIF2B2 | eukaryotic translation initiation factor 2B, subunit 2 beta, 39kDa | 8892 |
| 4368 | -2.6475 | 0.192643 | 2.49E-03 | 0.017043 | 0.601365 | H200012052 | NA | NA | - |
| 20353 | -2.4131 | 0.211684 | 4.72E-03 | 0.023284 | 0.601416 | H200019410 | NA | NA | - |
| 6785 | -1.9944 | 0.23702 | 1.56E-02 | 0.044842 | 0.601534 | H200018304 | NA | NA | - |
| 20968 | -2.4623 | 0.140335 | 4.12E-03 | 0.022023 | 0.601708 | H200008016 | KLK3 | kallikrein-related peptidase 3 | 354 |
| 19945 | -2.4602 | 0.235127 | 4.14E-03 | 0.02207 | 0.601815 | H200019280 | NA | NA | - |
| 18452 | -1.8632 | 0.308677 | 2.26E-02 | 0.055218 | 0.601846 | H200009245 | HSD17B8 | hydroxysteroid (17-beta) dehydrogenase 8 | 7923 |
| 6825 | -2.3252 | 0.296067 | 6.06E-03 | 0.026619 | 0.601953 | H200020204 | NA | NA | - |
| 4986 | -3.1801 | 0.171413 | 6.45E-04 | 0.009443 | 0.602221 | H200019877 | CLDN15 | claudin 15 | 24146 |
| 10178 | -2.1398 | 0.334173 | 1.03E-02 | 0.035819 | 0.602325 | H200005769 | IL19 | interleukin 19 | 29949 |
| 1174 | -2.7212 | 0.217245 | 2.05E-03 | 0.015632 | 0.602339 | H200012326 | GSR | glutathione reductase | 2936 |
| 2696 | -2.0844 | 0.222219 | 1.20E-02 | 0.038927 | 0.602458 | H200019700 | POLDIP3 | polymerase (DNA-directed), delta interacting protein 3 | 84271 |
| 18981 | -1.9635 | 0.256113 | 1.70E-02 | 0.047158 | 0.602687 | H200016222 | CYP2A7 | cytochrome P450, family 2, subfamily A, polypeptide 7 | 1549 |
| 13546 | -2.4615 | 0.203959 | 4.13E-03 | 0.022044 | 0.602752 | H200014021 | BCAM | basal cell adhesion molecule (Lutheran blood group) | 4059 |
| 19874 | -2.9764 | 0.190347 | 1.06E-03 | 0.011443 | 0.602964 | H200016162 | TNFRSF10A | tumor necrosis factor receptor superfamily, member 10a | 8797 |
| 13712 | -1.9114 | 0.295997 | 1.97E-02 | 0.051174 | 0.603214 | H200000044 | KEL | Kell blood group, metallo-endopeptidase | 3792 |
| 1049 | -2.368 | 0.180914 | 5.36E-03 | 0.024864 | 0.603333 | H200006572 | BTD | biotinidase | 686 |
| 12051 | -2.2126 | 0.203569 | 8.36E-03 | 0.031783 | 0.603649 | H200008113 | SLC35A4 | solute carrier family 35, member A4 | 113829 |
| 8704 | -3.4478 | 0.245076 | 3.51E-04 | 0.007621 | 0.603804 | H200001043 | RNPEPL1 | arginyl aminopeptidase (aminopeptidase B)-like 1 | 57140 |
| 20453 | -1.8497 | 0.492008 | 2.35E-02 | 0.056597 | 0.603885 | H200003023 | SUFU | suppressor of fused homolog (Drosophila) | 51684 |
| 19297 | -2.2441 | 0.250642 | 7.64E-03 | 0.030457 | 0.603919 | H200010065 | MTG1 | mitochondrial GTPase 1 homolog (S. cerevisiae) | 92170 |
| 18532 | -2.6186 | 0.207621 | 2.69E-03 | 0.017693 | 0.603931 | H200013467 | SH3RF2 | SH3 domain containing ring finger 2 | 153769 |
| 12309 | -2.1801 | 0.25919 | 9.16E-03 | 0.033586 | 0.604018 | H200020297 | NA | NA | - |
| 11439 | -1.9489 | 0.290822 | 1.77E-02 | 0.048288 | 0.60405 | H200000644 | TAC1 | tachykinin, precursor 1 | 6863 |
| 16286 | -3.2048 | 0.186814 | 6.08E-04 | 0.009361 | 0.604142 | H200013937 | NA | NA | - |
| 2258 | -1.8342 | 0.367237 | 2.46E-02 | 0.057829 | 0.604175 | H200020531 | DKFZP547L112 | uncharacterized protein DKFZp547L112 | 81787 |
| 287 | -2.0761 | 0.223209 | 1.23E-02 | 0.039392 | 0.604311 | H200013472 | CPA5 | carboxypeptidase A5 | 93979 |
| 8890 | -2.0831 | 0.385316 | 1.21E-02 | 0.038971 | 0.604479 | H200010091 | SOX18 | SRY (sex determining region Y)-box 18 | 54345 |
| 12595 | -1.9206 | 0.564343 | 1.92E-02 | 0.050366 | 0.60448 | H200012388 | PILRA | paired immunoglobin-like type 2 receptor alpha | 29992 |
| 15527 | -2.7621 | 0.158494 | 1.83E-03 | 0.014822 | 0.604897 | H200021163 | NA | NA | - |
| 10504 | -2.6298 | 0.178325 | 2.61E-03 | 0.017425 | 0.604899 | H200021325 | NA | NA | - |
| 18391 | -1.8489 | 0.37174 | 2.36E-02 | 0.05661 | 0.604937 | H200005849 | TNNT3 | troponin T type 3 (skeletal, fast) | 7140 |
| 2078 | -1.9955 | 0.186276 | 1.55E-02 | 0.044836 | 0.605143 | H200011839 | MAU2 | MAU2 chromatid cohesion factor homolog (C. elegans) | 23383 |
| 16520 | -2.201 | 0.261094 | 8.63E-03 | 0.03241 | 0.605153 | H200003119 | HTR6 | 5-hydroxytryptamine (serotonin) receptor 6, G protein-coupled | 3362 |
| 931 | -2.0965 | 0.272878 | 1.16E-02 | 0.038245 | 0.605157 | H200000896 | VAV2 | vav 2 guanine nucleotide exchange factor | 7410 |
| 11078 | -3.2078 | 0.210581 | 6.04E-04 | 0.009326 | 0.605163 | H200005091 | CD81 | CD81 molecule | 975 |
| 21465 | -2.4963 | 0.239139 | 3.75E-03 | 0.02091 | 0.605179 | H200009875 | SH3BP5L | SH3-binding domain protein 5-like | 80851 |
| 10430 | -1.7887 | 0.31673 | 2.80E-02 | 0.062622 | 0.605527 | H200017881 | TLL2 | tolloid-like 2 | 7093 |
| 15463 | -2.0943 | 0.361376 | 1.17E-02 | 0.038353 | 0.605574 | H200018123 | GLTPD1 | glycolipid transfer protein domain containing 1 | 80772 |
| 11871 | -3.1741 | 0.145379 | 6.55E-04 | 0.009537 | 0.605763 | H200021164 | NA | NA | - |
| 5862 | -2.4145 | 0.188731 | 4.70E-03 | 0.023272 | 0.60591 | H200017918 | NA | NA | - |
| 15280 | -2.2311 | 0.189127 | 7.93E-03 | 0.030995 | 0.605992 | H200009389 | NA | NA | - |
| 2594 | -2.295 | 0.144114 | 6.60E-03 | 0.027804 | 0.606013 | H200014784 | RALGAPA1 | Ral GTPase activating protein, alpha subunit 1 (catalytic) | 253959 |
| 3897 | -1.8801 | 0.297614 | 2.16E-02 | 0.05369 | 0.606078 | H200011499 | IGFBP5 | insulin-like growth factor binding protein 5 | 3488 |
| 4005 | -2.3321 | 0.27145 | 5.94E-03 | 0.026275 | 0.606227 | H200016487 | C11orf75 | chromosome 11 open reading frame 75 | 56935 |
| 670 | -1.9453 | 0.231975 | 1.79E-02 | 0.048493 | 0.60626 | H200010129 | VPS13C | vacuolar protein sorting 13 homolog C (S. cerevisiae) | 54832 |
| 14117 | -2.5895 | 0.169894 | 2.91E-03 | 0.0184 | 0.606322 | H200019394 | WWP2 | WW domain containing E3 ubiquitin protein ligase 2 | 11060 |
| 2112 | -2.4895 | 0.171414 | 3.82E-03 | 0.021096 | 0.606587 | H200013383 | NANP | N-acetylneuraminic acid phosphatase | 140838 |
| 6623 | -2.4894 | 0.223927 | 3.82E-03 | 0.021096 | 0.606603 | H200010680 | KISS1 | KiSS-1 metastasis-suppressor | 3814 |
| 20238 | -2.2295 | 0.273357 | 7.97E-03 | 0.031081 | 0.606853 | H200012998 | NA | NA | - |
| 5590 | -2.4867 | 0.143404 | 3.85E-03 | 0.021189 | 0.606881 | H200004998 | NAGLU | N-acetylglucosaminidase, alpha | 4669 |
| 20001 | -1.7794 | 0.394202 | 2.88E-02 | 0.063552 | 0.60698 | H200000386 | HDC | histidine decarboxylase | 3067 |
| 10222 | -2.3321 | 0.179966 | 5.94E-03 | 0.026275 | 0.606985 | H200008001 | POLR2A | polymerase (RNA) II (DNA directed) polypeptide A, 220kDa | 5430 |
| 10405 | -2.007 | 0.193861 | 1.50E-02 | 0.043917 | 0.607019 | H200016735 | MUTYH | mutY homolog (E. coli) | 4595 |
| 12940 | -2.0837 | 0.343851 | 1.21E-02 | 0.038948 | 0.607047 | H200006872 | FBXW5 | F-box and WD repeat domain containing 5 | 54461 |
| 6410 | -1.999 | 0.298497 | 1.54E-02 | 0.044643 | 0.607072 | H200000450 | IL11 | interleukin 11 | 3589 |
| 382 | -1.989 | 0.126722 | 1.58E-02 | 0.045187 | 0.60716 | H200018014 | NEU4 | sialidase 4 | 129807 |
| 9628 | -2.4698 | 0.220297 | 4.04E-03 | 0.021781 | 0.60718 | H200001506 | MGC2752 | CENPB DNA-binding domains containing 1 pseudogene | 65996 |
| 17835 | -2.073 | 0.272884 | 1.24E-02 | 0.039497 | 0.607221 | H200000684 | POU6F1 | POU class 6 homeobox 1 | 5463 |
| 704 | -2.3522 | 0.194711 | 5.61E-03 | 0.025526 | 0.607393 | H200011673 | GRIPAP1 | GRIP1 associated protein 1 | 56850 |
| 14083 | -3.4018 | 0.164112 | 3.86E-04 | 0.00776 | 0.607729 | H200017566 | C19orf53 | chromosome 19 open reading frame 53 | 28974 |
| 16974 | -2.7022 | 0.232379 | 2.15E-03 | 0.015982 | 0.607734 | H200003326 | CHFR | checkpoint with forkhead and ring finger domains, E3 ubiquitin protein ligase | 55743 |
| 7560 | -2.9713 | 0.228351 | 1.07E-03 | 0.011471 | 0.607985 | H200011576 | OTUB1 | OTU domain, ubiquitin aldehyde binding 1 | 55611 |
| 17846 | -1.8398 | 0.293178 | 2.42E-02 | 0.057425 | 0.608051 | H200001106 | YIF1A | Yip1 interacting factor homolog A (S. cerevisiae) | 10897 |
| 15391 | -3.4425 | 0.185829 | 3.56E-04 | 0.007621 | 0.608063 | H200014703 | ZBTB45 | zinc finger and BTB domain containing 45 | 84878 |
| 14317 | -2.4481 | 0.166047 | 4.29E-03 | 0.022273 | 0.608352 | H200007187 | RNF216 | ring finger protein 216 | 54476 |
| 14904 | -2.3121 | 0.503785 | 6.29E-03 | 0.027033 | 0.608466 | H200013094 | ALDH16A1 | aldehyde dehydrogenase 16 family, member A1 | 126133 |
| 11997 | -3.4001 | 0.1279 | 3.88E-04 | 0.00776 | 0.608726 | H200005477 | SFI1 | Sfi1 homolog, spindle assembly associated (yeast) | 9814 |
| 15087 | -2.1259 | 0.215542 | 1.07E-02 | 0.03669 | 0.60875 | H200000263 | BRPF1 | bromodomain and PHD finger containing, 1 | 7862 |
| 7387 | -2.8849 | 0.12613 | 1.33E-03 | 0.012562 | 0.608837 | H200003542 | ELOVL1 | ELOVL fatty acid elongase 1 | 64834 |
| 20403 | -1.8673 | 0.274909 | 2.24E-02 | 0.054817 | 0.608909 | H200000357 | CYP17A1 | cytochrome P450, family 17, subfamily A, polypeptide 1 | 1586 |
| 8400 | -2.4839 | 0.165381 | 3.88E-03 | 0.021306 | 0.609145 | H200008168 | NA | NA | - |
| 10415 | -3.2797 | 0.159545 | 5.12E-04 | 0.00873 | 0.609231 | H200017139 | NA | NA | - |
| 18134 | -2.6478 | 0.153035 | 2.49E-03 | 0.017043 | 0.609239 | H200014786 | NA | NA | - |
| 13841 | -3.5155 | 0.127814 | 3.01E-04 | 0.007429 | 0.609294 | H200006142 | UROS | uroporphyrinogen III synthase | 7390 |
| 10858 | -2.1173 | 0.35051 | 1.10E-02 | 0.037142 | 0.609304 | H200016646 | SH2D3A | SH2 domain containing 3A | 10045 |
| 21169 | -2.1514 | 0.260487 | 9.94E-03 | 0.035107 | 0.609365 | H200017522 | ADAMTS6 | ADAM metallopeptidase with thrombospondin type 1 motif, 6 | 11174 |
| 8964 | -2.0014 | 0.241482 | 1.52E-02 | 0.044427 | 0.609456 | H200013535 | NA | NA | - |
| 10009 | -2.5672 | 0.173722 | 3.10E-03 | 0.018905 | 0.609462 | H200019716 | FAM104A | family with sequence similarity 104, member A | 84923 |
| 12115 | -1.9898 | 0.308891 | 1.58E-02 | 0.045106 | 0.609635 | H200011153 | SIX2 | SIX homeobox 2 | 10736 |
| 2690 | -1.8924 | 0.332458 | 2.08E-02 | 0.052624 | 0.609742 | H200019344 | NAA10 | N(alpha)-acetyltransferase 10, NatA catalytic subunit | 8260 |
| 2208 | -2.1617 | 0.348695 | 9.66E-03 | 0.034632 | 0.609752 | H200017943 | NA | NA | - |
| 21173 | -2.44 | 0.180199 | 4.39E-03 | 0.022409 | 0.609763 | H200017570 | EPN1 | epsin 1 | 29924 |
| 14636 | -1.9797 | 0.379578 | 1.62E-02 | 0.045851 | 0.609815 | H200000506 | PDGFB | platelet-derived growth factor beta polypeptide | 5155 |
| 9709 | -2.4811 | 0.187882 | 3.91E-03 | 0.021385 | 0.610665 | H200005608 | ENTHD2 | ENTH domain containing 2 | 146705 |
| 7419 | -1.809 | 0.338469 | 2.64E-02 | 0.06046 | 0.610775 | H200005062 | KLC4 | kinesin light chain 4 | 89953 |
| 14590 | -3.1844 | 0.139557 | 6.38E-04 | 0.009407 | 0.610942 | H200020113 | OCEL1 | occludin/ELL domain containing 1 | 79629 |
| 18658 | -2.0525 | 0.269615 | 1.32E-02 | 0.040762 | 0.611018 | H200020307 | NA | NA | - |
| 8612 | -2.3827 | 0.241361 | 5.14E-03 | 0.024326 | 0.611124 | H200018380 | NA | NA | - |
| 17625 | -2.8223 | 0.288795 | 1.57E-03 | 0.01371 | 0.611146 | H200012203 | C1QTNF4 | C1q and tumor necrosis factor related protein 4 | 114900 |
| 10303 | -1.9088 | 2.079147 | 1.99E-02 | 0.051357 | 0.611234 | H200011819 | S100A9 | S100 calcium binding protein A9 | 6280 |
| 7357 | -2.1608 | 0.238114 | 9.69E-03 | 0.034648 | 0.61148 | H200002046 | YIPF1 | Yip1 domain family, member 1 | 54432 |
| 15169 | -2.2585 | 0.185675 | 7.33E-03 | 0.02977 | 0.611665 | H200004371 | CALCA | calcitonin-related polypeptide alpha | 796 |
| 15743 | -3.0827 | 0.1597 | 8.13E-04 | 0.010284 | 0.611729 | H200009680 | GRB2 | growth factor receptor-bound protein 2 | 2885 |
| 1887 | -2.5961 | 0.203206 | 2.86E-03 | 0.018254 | 0.611733 | H200002737 | SSNA1 | Sjogren syndrome nuclear autoantigen 1 | 8636 |
| 12354 | -2.5485 | 0.22267 | 3.26E-03 | 0.019458 | 0.611738 | H200000970 | RBM23 | RNA binding motif protein 23 | 55147 |
| 6286 | -2.7521 | 0.182982 | 1.88E-03 | 0.015031 | 0.612022 | H200016635 | FZR1 | fizzy/cell division cycle 20 related 1 (Drosophila) | 51343 |
| 20803 | -1.9315 | 0.265812 | 1.86E-02 | 0.049553 | 0.612058 | H200000066 | G6PC | glucose-6-phosphatase, catalytic subunit | 2538 |
| 4630 | -2.715 | 0.196475 | 2.08E-03 | 0.015763 | 0.612265 | H200002825 | IFI27L1 | interferon, alpha-inducible protein 27-like 1 | 122509 |
| 366 | -2.4694 | 0.236737 | 4.04E-03 | 0.021798 | 0.612426 | H200017254 | HEXDC | hexosaminidase (glycosyl hydrolase family 20, catalytic domain) containing | 284004 |
| 14132 | -2.7488 | 0.192746 | 1.90E-03 | 0.015105 | 0.612441 | H200019852 | CTU1 | cytosolic thiouridylase subunit 1 homolog (S. pombe) | 90353 |
| 8474 | -3.8161 | 0.085546 | 1.61E-04 | 0.005936 | 0.612733 | H200011896 | GABRD | gamma-aminobutyric acid (GABA) A receptor, delta | 2563 |
| 14139 | -1.902 | 0.374593 | 2.03E-02 | 0.051952 | 0.612765 | H200020226 | EPB41L1 | erythrocyte membrane protein band 4.1-like 1 | 2036 |
| 51 | -2.3612 | 0.166223 | 5.47E-03 | 0.02517 | 0.612822 | H200002404 | C16orf3 | chromosome 16 open reading frame 3 | 750 |
| 3205 | -2.2159 | 0.24586 | 8.28E-03 | 0.031626 | 0.612862 | H200000350 | CLPS | colipase, pancreatic | 1208 |
| 7091 | -2.1262 | 0.292115 | 1.07E-02 | 0.036682 | 0.612981 | H200011345 | PPM1J | protein phosphatase, Mg2+/Mn2+ dependent, 1J | 333926 |
| 8279 | -1.9293 | 0.275214 | 1.87E-02 | 0.049699 | 0.613117 | H200002462 | ADCK1 | aarF domain containing kinase 1 | 57143 |
| 314 | -1.8171 | 0.173887 | 2.58E-02 | 0.059713 | 0.613304 | H200014926 | EMX2 | empty spiracles homeobox 2 | 2018 |
| 3794 | -3.1062 | 0.173337 | 7.69E-04 | 0.010006 | 0.613384 | H200006565 | TAF6 | TAF6 RNA polymerase II, TATA box binding protein (TBP)-associated factor, 80kDa | 6878 |
| 15487 | -2.139 | 0.243587 | 1.03E-02 | 0.035839 | 0.61361 | H200019263 | GPR61 | G protein-coupled receptor 61 | 83873 |
| 13690 | -2.6347 | 0.126544 | 2.58E-03 | 0.01733 | 0.613625 | H200020861 | NA | NA | - |
| 11340 | -2.1029 | 0.238882 | 1.14E-02 | 0.037908 | 0.613716 | H200017607 | PGLYRP2 | peptidoglycan recognition protein 2 | 114770 |
| 10634 | -3.4182 | 0.113605 | 3.72E-04 | 0.007706 | 0.613874 | H200006006 | RNH1 | ribonuclease/angiogenin inhibitor 1 | 6050 |
| 13371 | -2.3002 | 0.222685 | 6.50E-03 | 0.027522 | 0.613896 | H200005679 | LOC153577 | uncharacterized LOC153577 | 153577 |
| 5650 | -1.832 | 0.210326 | 2.48E-02 | 0.058105 | 0.61417 | H200007990 | TMA7 | translation machinery associated 7 homolog (S. cerevisiae) | 51372 |
| 9868 | -2.0172 | 0.256685 | 1.46E-02 | 0.043372 | 0.614698 | H200012906 | NA | NA | - |
| 2319 | -1.9113 | 0.277816 | 1.97E-02 | 0.051176 | 0.614881 | H200001834 | ZNF275 | zinc finger protein 275 | 10838 |
| 386 | -1.9476 | 0.238889 | 1.78E-02 | 0.048329 | 0.615038 | H200018346 | ZNF71 | zinc finger protein 71 | 58491 |
| 14539 | -2.6834 | 0.211621 | 2.26E-03 | 0.016277 | 0.615223 | H200017803 | GJA3 | gap junction protein, alpha 3, 46kDa | 2700 |
| 20811 | -2.8871 | 0.371463 | 1.32E-03 | 0.012532 | 0.615368 | H200000446 | IRF9 | interferon regulatory factor 9 | 10379 |
| 16845 | -1.8801 | 0.33267 | 2.16E-02 | 0.05369 | 0.615409 | H200018669 | NA | NA | - |
| 9358 | -2.1162 | 0.337452 | 1.10E-02 | 0.037198 | 0.615561 | H200010317 | CXCR4 | chemokine (C-X-C motif) receptor 4 | 7852 |
| 10662 | -2.2576 | 0.298229 | 7.35E-03 | 0.029789 | 0.615578 | H200007194 | CHPF2 | chondroitin polymerizing factor 2 | 54480 |
| 4333 | -2.3005 | 0.215845 | 6.50E-03 | 0.027502 | 0.615629 | H200010502 | NPTXR | neuronal pentraxin receptor | 23467 |
| 9498 | -1.9636 | 0.277771 | 1.70E-02 | 0.047158 | 0.615778 | H200017109 | SIGLEC7 | sialic acid binding Ig-like lectin 7 | 27036 |
| 2960 | -2.3514 | 0.2514 | 5.62E-03 | 0.025556 | 0.615834 | H200010307 | KCNN3 | potassium intermediate/small conductance calcium-activated channel, subfamily N, member 3 | 3782 |
| 7907 | -3.064 | 0.138809 | 8.51E-04 | 0.010429 | 0.615964 | H200006677 | TESK1 | testis-specific kinase 1 | 7016 |
| 11276 | -2.4467 | 0.144336 | 4.30E-03 | 0.022315 | 0.616048 | H200014567 | IL6R | interleukin 6 receptor | 3570 |
| 10564 | -2.1713 | 0.218263 | 9.39E-03 | 0.03408 | 0.616394 | H200002610 | SLC25A19 | solute carrier family 25 (mitochondrial thiamine pyrophosphate carrier), member 19 | 60386 |
| 2613 | -2.904 | 0.151926 | 1.27E-03 | 0.012344 | 0.616396 | H200015870 | SCD5 | stearoyl-CoA desaturase 5 | 79966 |
| 16995 | -2.3703 | 0.231377 | 5.33E-03 | 0.024783 | 0.61647 | H200004140 | SNAPIN | SNAP-associated protein | 23557 |
| 21411 | -2.5893 | 0.254312 | 2.91E-03 | 0.0184 | 0.616516 | H200007523 | PIN1 | peptidylprolyl cis/trans isomerase, NIMA-interacting 1 | 5300 |
| 282 | -2.207 | 0.207141 | 8.49E-03 | 0.032081 | 0.616519 | H200013406 | PURB | purine-rich element binding protein B | 5814 |
| 8411 | -2.2606 | 0.186517 | 7.29E-03 | 0.029671 | 0.616613 | H200008874 | NRSN2 | neurensin 2 | 80023 |
| 17900 | -2.4379 | 0.244705 | 4.41E-03 | 0.022443 | 0.616709 | H200003742 | GFER | growth factor, augmenter of liver regeneration | 2671 |
| 4307 | -2.4302 | 0.24464 | 4.50E-03 | 0.02272 | 0.616941 | H200009338 | NA | NA | - |
| 21000 | -2.2404 | 0.226059 | 7.72E-03 | 0.030646 | 0.617106 | H200009536 | S100A1 | S100 calcium binding protein A1 | 6271 |
| 18286 | -2.3842 | 0.255053 | 5.12E-03 | 0.024315 | 0.6173 | H200000149 | AADAC | arylacetamide deacetylase | 13 |
| 19400 | -2.0506 | 0.22897 | 1.33E-02 | 0.040863 | 0.6174 | H200014999 | FAM65C | family with sequence similarity 65, member C | 140876 |
| 5904 | -2.6961 | 0.181006 | 2.19E-03 | 0.016152 | 0.617425 | H200019842 | AFG3L1P | AFG3 ATPase family member 3-like 1 (S. cerevisiae), pseudogene | 172 |
| 13633 | -2.1638 | 0.238397 | 9.60E-03 | 0.034569 | 0.61743 | H200018195 | TG | thyroglobulin | 7038 |
| 11016 | -1.8719 | 0.229126 | 2.21E-02 | 0.054399 | 0.617483 | H200002075 | KIAA1033 | KIAA1033 | 23325 |
| 20824 | -2.2532 | 0.364668 | 7.44E-03 | 0.029988 | 0.617484 | H200001176 | CORO1B | coronin, actin binding protein, 1B | 57175 |
| 15249 | -3.7511 | 0.130745 | 1.83E-04 | 0.006172 | 0.61754 | H200008171 | ESAM | endothelial cell adhesion molecule | 90952 |
| 1263 | -2.4722 | 0.180919 | 4.01E-03 | 0.021722 | 0.617591 | H200016524 | TRPC4 | transient receptor potential cation channel, subfamily C, member 4 | 7223 |
| 11436 | -3.162 | 0.133748 | 6.73E-04 | 0.009642 | 0.617638 | H200000602 | ZBTB48 | zinc finger and BTB domain containing 48 | 3104 |
| 20745 | -1.7739 | 0.26053 | 2.92E-02 | 0.064168 | 0.617674 | H200018959 | TMEM222 | transmembrane protein 222 | 84065 |
| 8872 | -2.0367 | 0.236841 | 1.38E-02 | 0.041906 | 0.617712 | H200009023 | NA | NA | - |
| 13648 | -1.9302 | 0.230738 | 1.87E-02 | 0.049621 | 0.618149 | H200018937 | MTSS1L | metastasis suppressor 1-like | 92154 |
| 10274 | -2.8121 | 0.20895 | 1.61E-03 | 0.013872 | 0.618304 | H200010329 | ECI1 | enoyl-CoA delta isomerase 1 | 1632 |
| 8654 | -1.948 | 0.418799 | 1.78E-02 | 0.048329 | 0.618343 | H200020304 | NA | NA | - |
| 16259 | -2.4335 | 0.297137 | 4.46E-03 | 0.022579 | 0.618452 | H200012767 | SLC45A1 | solute carrier family 45, member 1 | 50651 |
| 14357 | -2.5822 | 0.13982 | 2.97E-03 | 0.018531 | 0.618533 | H200009087 | NA | NA | - |
| 11435 | -2.2082 | 0.219607 | 8.46E-03 | 0.032052 | 0.618585 | H200000596 | PROC | protein C (inactivator of coagulation factors Va and VIIIa) | 5624 |
| 994 | -1.8571 | 0.294799 | 2.30E-02 | 0.055839 | 0.618592 | H200003918 | CCDC74B | coiled-coil domain containing 74B | 91409 |
| 5147 | -1.7857 | 0.315025 | 2.82E-02 | 0.062936 | 0.618714 | H200005930 | HDAC11 | histone deacetylase 11 | 79885 |
| 14588 | -2.4963 | 0.216555 | 3.75E-03 | 0.02091 | 0.618716 | H200020089 | NA | NA | - |
| 18244 | -2.2179 | 0.267789 | 8.24E-03 | 0.031553 | 0.618838 | H200020082 | SOCS3 | suppressor of cytokine signaling 3 | 9021 |
| 14338 | -2.1461 | 0.248979 | 1.01E-02 | 0.035412 | 0.618994 | H200008285 | ZSCAN5A | zinc finger and SCAN domain containing 5A | 79149 |
| 11598 | -2.7401 | 0.192736 | 1.95E-03 | 0.015285 | 0.619032 | H200008226 | KCNK7 | potassium channel, subfamily K, member 7 | 10089 |
| 21704 | -2.405 | 0.200733 | 4.83E-03 | 0.023541 | 0.619197 | H200021269 | C21orf67 | chromosome 21 open reading frame 67 | 84536 |
| 19807 | -1.8782 | 0.341994 | 2.17E-02 | 0.053817 | 0.619467 | H200012796 | NA | NA | - |
| 4700 | -2.692 | 0.179922 | 2.22E-03 | 0.016186 | 0.619514 | H200006221 | LMAN2 | lectin, mannose-binding 2 | 10960 |
| 8988 | -2.1448 | 0.202522 | 1.01E-02 | 0.035498 | 0.619699 | H200014675 | CACNA1G | calcium channel, voltage-dependent, T type, alpha 1G subunit | 8913 |
| 4453 | -2.7059 | 0.130515 | 2.13E-03 | 0.015941 | 0.619842 | H200016202 | NA | NA | 26221 |
| 5048 | -2.3604 | 0.227795 | 5.48E-03 | 0.02521 | 0.61987 | H200001044 | CASP10 | caspase 10, apoptosis-related cysteine peptidase | 843 |
| 19428 | -2.4576 | 0.220951 | 4.18E-03 | 0.02211 | 0.620335 | H200016471 | NA | NA | - |
| 9321 | -2.3658 | 0.22945 | 5.40E-03 | 0.024946 | 0.620347 | H200008743 | HCN3 | hyperpolarization activated cyclic nucleotide-gated potassium channel 3 | 57657 |
| 1378 | -1.8725 | 0.257517 | 2.20E-02 | 0.054378 | 0.620402 | H200000593 | PRKAA2 | protein kinase, AMP-activated, alpha 2 catalytic subunit | 5563 |
| 11011 | -1.8038 | 0.366248 | 2.68E-02 | 0.061154 | 0.620572 | H200002021 | ACSL5 | acyl-CoA synthetase long-chain family member 5 | 51703 |
| 14203 | -2.1591 | 0.225357 | 9.73E-03 | 0.034686 | 0.620773 | H200001843 | GUCD1 | guanylyl cyclase domain containing 1 | 83606 |
| 13536 | -1.943 | 0.297822 | 1.80E-02 | 0.048741 | 0.620789 | H200013617 | ZNF778 | zinc finger protein 778 | 197320 |
| 13280 | -2.0534 | 0.216486 | 1.31E-02 | 0.040716 | 0.620943 | H200001457 | BLOC1S4 | biogenesis of lysosomal organelles complex-1, subunit 4, cappuccino | 55330 |
| 9961 | -2.4391 | 0.222035 | 4.40E-03 | 0.022429 | 0.621038 | H200017436 | RNF185 | ring finger protein 185 | 91445 |
| 9003 | -2.3417 | 0.075204 | 5.78E-03 | 0.02598 | 0.621166 | H200015429 | TMEM203 | transmembrane protein 203 | 94107 |
| 13576 | -2.3255 | 0.209868 | 6.06E-03 | 0.026616 | 0.621182 | H200015517 | FEV | FEV (ETS oncogene family) | 54738 |
| 21429 | -2.3334 | 0.248457 | 5.92E-03 | 0.026252 | 0.621192 | H200008307 | NA | NA | - |
| 18451 | -2.3435 | 0.226978 | 5.75E-03 | 0.025895 | 0.621257 | H200009239 | ZC3H12A | zinc finger CCCH-type containing 12A | 80149 |
| 8559 | -2.3878 | 0.236525 | 5.07E-03 | 0.024167 | 0.621374 | H200015762 | NA | NA | - |
| 6634 | -2.7223 | 0.167871 | 2.04E-03 | 0.015627 | 0.62145 | H200011090 | ATAD1 | ATPase family, AAA domain containing 1 | 84896 |
| 17015 | -2.7873 | 0.1404 | 1.72E-03 | 0.014296 | 0.621704 | H200005232 | URM1 | ubiquitin related modifier 1 | 81605 |
| 10839 | -2.4219 | 0.282696 | 4.60E-03 | 0.022996 | 0.621857 | H200015572 | BRAT1 | BRCA1-associated ATM activator 1 | 221927 |
| 15317 | -2.359 | 0.290661 | 5.51E-03 | 0.025278 | 0.621918 | H200011259 | GID8 | GID complex subunit 8 homolog (S. cerevisiae) | 54994 |
| 2589 | -2.8904 | 0.069665 | 1.31E-03 | 0.012515 | 0.621925 | H200014730 | PHKG2 | phosphorylase kinase, gamma 2 (testis) | 5261 |
| 11135 | -2.0954 | 0.294056 | 1.17E-02 | 0.038279 | 0.621969 | H200007769 | OGDH | oxoglutarate (alpha-ketoglutarate) dehydrogenase (lipoamide) | 4967 |
| 15486 | -2.2554 | 0.249564 | 7.39E-03 | 0.029872 | 0.621991 | H200019245 | CACNG8 | calcium channel, voltage-dependent, gamma subunit 8 | 59283 |
| 5086 | -1.9474 | 0.115894 | 1.78E-02 | 0.048348 | 0.622066 | H200002920 | BCKDK | branched chain ketoacid dehydrogenase kinase | 10295 |
| 15752 | -2.3905 | 0.213516 | 5.03E-03 | 0.024081 | 0.622137 | H200010066 | FAM214B | family with sequence similarity 214, member B | 80256 |
| 15917 | -1.9697 | 0.329461 | 1.67E-02 | 0.046698 | 0.622209 | H200018016 | NPFFR1 | neuropeptide FF receptor 1 | 64106 |
| 19637 | -2.7083 | 0.159515 | 2.12E-03 | 0.01593 | 0.622266 | H200004792 | TMPRSS5 | transmembrane protease, serine 5 | 80975 |
| 17644 | -2.0313 | 0.170708 | 1.40E-02 | 0.04233 | 0.622429 | H200013005 | LOC115110 | uncharacterized LOC115110 | 115110 |
| 21515 | -2.024 | 0.218306 | 1.43E-02 | 0.042857 | 0.622446 | H200012463 | MAGEC2 | melanoma antigen family C, 2 | 51438 |
| 10248 | -2.6996 | 0.134608 | 2.17E-03 | 0.016035 | 0.622635 | H200009165 | LRRC8E | leucine rich repeat containing 8 family, member E | 80131 |
| 4133 | -1.9548 | 0.450017 | 1.74E-02 | 0.047858 | 0.622939 | H200001002 | TRAFD1 | TRAF-type zinc finger domain containing 1 | 10906 |
| 5771 | -2.3388 | 0.11901 | 5.82E-03 | 0.026096 | 0.622965 | H200013708 | LMF2 | lipase maturation factor 2 | 91289 |
| 6175 | -1.892 | 0.255398 | 2.08E-02 | 0.052648 | 0.623008 | H200011333 | ZNF821 | zinc finger protein 821 | 55565 |
| 13943 | -2.0762 | 0.371084 | 1.23E-02 | 0.039392 | 0.62302 | H200011058 | UTP15 | UTP15, U3 small nucleolar ribonucleoprotein, homolog (S. cerevisiae) | 84135 |
| 2116 | -2.0569 | 0.221419 | 1.30E-02 | 0.040527 | 0.623149 | H200013715 | PSKH1 | protein serine kinase H1 | 5681 |
| 19600 | -1.9345 | 0.175622 | 1.84E-02 | 0.049352 | 0.623345 | H200002934 | NA | NA | - |
| 7994 | -2.0072 | 0.281113 | 1.50E-02 | 0.043914 | 0.623557 | H200010839 | ALLC | allantoicase | 55821 |
| 4752 | -2.4596 | 0.235399 | 4.15E-03 | 0.022084 | 0.623595 | H200008549 | GRIK1 | glutamate receptor, ionotropic, kainate 1 | 2897 |
| 8431 | -2.5342 | 0.135066 | 3.38E-03 | 0.019764 | 0.623604 | H200009682 | PPP6R2 | protein phosphatase 6, regulatory subunit 2 | 9701 |
| 9616 | -3.2151 | 0.119789 | 5.94E-04 | 0.00931 | 0.623615 | H200001078 | MKNK1 | MAP kinase interacting serine/threonine kinase 1 | 8569 |
| 11963 | -2.8594 | 0.160094 | 1.43E-03 | 0.013052 | 0.623828 | H200003933 | PRAF2 | PRA1 domain family, member 2 | 11230 |
| 7451 | -1.939 | 0.311269 | 1.82E-02 | 0.049047 | 0.623928 | H200006582 | RABGGTA | Rab geranylgeranyltransferase, alpha subunit | 5875 |
| 17414 | -2.6729 | 0.182729 | 2.33E-03 | 0.016549 | 0.623976 | H200002293 | NA | NA | - |
| 916 | -2.4416 | 0.182242 | 4.37E-03 | 0.022354 | 0.624205 | H200000142 | ADRA1D | adrenoceptor alpha 1D | 146 |
| 12043 | -1.9906 | 0.05192 | 1.57E-02 | 0.045068 | 0.624286 | H200007733 | STAT5A | signal transducer and activator of transcription 5A | 6776 |
| 5870 | -2.4514 | 0.210059 | 4.25E-03 | 0.022206 | 0.624291 | H200018298 | IQCK | IQ motif containing K | 124152 |
| 7080 | -3.3837 | 0.13458 | 4.03E-04 | 0.007913 | 0.624305 | H200010639 | RAB35 | RAB35, member RAS oncogene family | 11021 |
| 21181 | -1.8728 | 0.439952 | 2.20E-02 | 0.05433 | 0.624347 | H200017950 | TEX264 | testis expressed 264 | 51368 |
| 875 | -2.1157 | 0.198443 | 1.10E-02 | 0.037215 | 0.624534 | H200019979 | TTBK1 | tau tubulin kinase 1 | 84630 |
| 15840 | -3.018 | 0.147034 | 9.53E-04 | 0.010914 | 0.624661 | H200014246 | OTUD5 | OTU domain containing 5 | 55593 |
| 7062 | -2.1701 | 0.14834 | 9.42E-03 | 0.034137 | 0.624832 | H200009855 | NA | NA | - |
| 15220 | -1.9646 | 0.318248 | 1.69E-02 | 0.047086 | 0.624887 | H200006681 | RXRB | retinoid X receptor, beta | 6257 |
| 11968 | -2.4521 | 0.235019 | 4.24E-03 | 0.022198 | 0.625381 | H200003987 | ZHX2 | zinc fingers and homeoboxes 2 | 22882 |
| 11919 | -2.0814 | 0.206579 | 1.21E-02 | 0.039029 | 0.625392 | H200001701 | HAUS4 | HAUS augmin-like complex, subunit 4 | 54930 |
| 5196 | -3.353 | 0.12365 | 4.33E-04 | 0.00823 | 0.625633 | H200008216 | SLC22A12 | solute carrier family 22 (organic anion/urate transporter), member 12 | 116085 |
| 15622 | -2.8902 | 0.193685 | 1.31E-03 | 0.012515 | 0.625683 | H200003962 | RNF135 | ring finger protein 135 | 84282 |
| 9208 | -2.9055 | 0.153237 | 1.26E-03 | 0.012324 | 0.625773 | H200003121 | LRRC29 | leucine rich repeat containing 29 | 26231 |
| 9842 | -1.9978 | 0.425232 | 1.54E-02 | 0.044721 | 0.625977 | H200011742 | MEF2D | myocyte enhancer factor 2D | 4209 |
| 96 | -2.5067 | 0.202002 | 3.65E-03 | 0.020598 | 0.625978 | H200004358 | ALPI | alkaline phosphatase, intestinal | 248 |
| 2238 | -2.2384 | 0.202347 | 7.77E-03 | 0.030657 | 0.626144 | H200019439 | NA | NA | - |
| 13019 | -2.2044 | 0.263932 | 8.55E-03 | 0.032222 | 0.626442 | H200010666 | KCTD17 | potassium channel tetramerisation domain containing 17 | 79734 |
| 16477 | -2.6622 | 0.163767 | 2.40E-03 | 0.016816 | 0.626633 | H200001189 | INTS5 | integrator complex subunit 5 | 80789 |
| 6091 | -2.1733 | 0.348063 | 9.33E-03 | 0.033945 | 0.626783 | H200007201 | GYLTL1B | glycosyltransferase-like 1B | 120071 |
| 20897 | -2.58 | 0.208829 | 2.99E-03 | 0.018549 | 0.626804 | H200004602 | ATF6B | activating transcription factor 6 beta | 1388 |
| 2954 | -2.0564 | 0.207631 | 1.30E-02 | 0.040554 | 0.626973 | H200009951 | KDM4B | lysine (K)-specific demethylase 4B | 23030 |
| 7990 | -2.101 | 0.326951 | 1.15E-02 | 0.038017 | 0.627032 | H200010507 | PGAP3 | post-GPI attachment to proteins 3 | 93210 |
| 3847 | -2.1424 | 0.3101 | 1.02E-02 | 0.035671 | 0.62711 | H200008911 | NA | NA | - |
| 6083 | -2.771 | 0.189593 | 1.79E-03 | 0.01462 | 0.627156 | H200006821 | IL1RN | interleukin 1 receptor antagonist | 3557 |
| 20859 | -3.1511 | 0.139044 | 6.89E-04 | 0.009761 | 0.627176 | H200002726 | TLN1 | talin 1 | 7094 |
| 15500 | -3.9779 | 0.078149 | 1.20E-04 | 0.005583 | 0.627414 | H200019981 | NME6 | NME/NM23 nucleoside diphosphate kinase 6 | 10201 |
| 18109 | -1.9254 | 0.358457 | 1.89E-02 | 0.049989 | 0.627585 | H200013628 | STK33 | serine/threonine kinase 33 | 65975 |
| 11657 | -2.1172 | 0.281654 | 1.10E-02 | 0.037142 | 0.628323 | H200011212 | ZNF880 | zinc finger protein 880 | 400713 |
| 14292 | -1.9538 | 0.252599 | 1.75E-02 | 0.047909 | 0.628392 | H200006029 | PPP3CC | protein phosphatase 3, catalytic subunit, gamma isozyme | 5533 |
| 655 | -2.0671 | 0.211817 | 1.26E-02 | 0.039775 | 0.628448 | H200009387 | NA | NA | - |
| 16309 | -1.8309 | 0.384168 | 2.48E-02 | 0.058249 | 0.628521 | H200015071 | NA | NA | - |
| 14086 | -2.6888 | 0.146057 | 2.23E-03 | 0.016186 | 0.628559 | H200017880 | AP1S3 | adaptor-related protein complex 1, sigma 3 subunit | 130340 |
| 7253 | -2.1795 | 0.217454 | 9.17E-03 | 0.033618 | 0.628613 | H200018969 | NA | NA | - |
| 14037 | -2.0108 | 0.172573 | 1.48E-02 | 0.043697 | 0.628637 | H200015594 | DNAJB5 | DnaJ (Hsp40) homolog, subfamily B, member 5 | 25822 |
| 20352 | -1.8716 | 0.229848 | 2.21E-02 | 0.054414 | 0.628955 | H200019404 | CAPZB | capping protein (actin filament) muscle Z-line, beta | 832 |
| 9230 | -2.9412 | 0.13605 | 1.15E-03 | 0.011848 | 0.629339 | H200004237 | ZBTB17 | zinc finger and BTB domain containing 17 | 7709 |
| 1758 | -2.3035 | 0.191768 | 6.44E-03 | 0.027359 | 0.629482 | H200018501 | RAB4B | RAB4B, member RAS oncogene family | 53916 |
| 20779 | -2.3096 | 0.249023 | 6.33E-03 | 0.027123 | 0.629483 | H200020853 | ANTXR2 | anthrax toxin receptor 2 | 118429 |
| 9465 | -2.2278 | 0.19653 | 8.01E-03 | 0.031128 | 0.629594 | H200015583 | NA | NA | - |
| 16814 | -2.199 | 0.21635 | 8.68E-03 | 0.032474 | 0.629763 | H200017155 | NA | NA | - |
| 16932 | -2.3332 | 0.259326 | 5.92E-03 | 0.026253 | 0.629773 | H200001118 | ELL | elongation factor RNA polymerase II | 8178 |
| 2772 | -3.6288 | 0.113677 | 2.34E-04 | 0.006651 | 0.630056 | H200001235 | PACSIN1 | protein kinase C and casein kinase substrate in neurons 1 | 29993 |
| 12428 | -2.015 | 0.307763 | 1.47E-02 | 0.043498 | 0.630189 | H200004414 | SUV39H1 | suppressor of variegation 3-9 homolog 1 (Drosophila) | 6839 |
| 15482 | -2.336 | 0.260713 | 5.87E-03 | 0.026206 | 0.63025 | H200019197 | LRRC3 | leucine rich repeat containing 3 | 81543 |
| 10021 | -1.9477 | 0.303233 | 1.78E-02 | 0.048329 | 0.630273 | H200020428 | HOXA-AS2 | HOXA cluster antisense RNA 2 | 285943 |
| 12828 | -2.4691 | 0.198125 | 4.05E-03 | 0.021805 | 0.630284 | H200001552 | WBSCR17 | Williams-Beuren syndrome chromosome region 17 | 64409 |
| 20808 | -2.2872 | 0.218031 | 6.75E-03 | 0.028122 | 0.630318 | H200000416 | COMP | cartilage oligomeric matrix protein | 1311 |
| 11313 | -2.0844 | 0.23552 | 1.20E-02 | 0.038927 | 0.630346 | H200016437 | MICAL3 | microtubule associated monoxygenase, calponin and LIM domain containing 3 | 57553 |
| 17446 | -2.1593 | 0.35828 | 9.73E-03 | 0.034686 | 0.63044 | H200003813 | EIF3G | eukaryotic translation initiation factor 3, subunit G | 8666 |
| 17950 | -2.6385 | 0.209917 | 2.55E-03 | 0.01723 | 0.630615 | H200006046 | IDH3G | isocitrate dehydrogenase 3 (NAD+) gamma | 3421 |
| 1450 | -2.057 | 0.309515 | 1.30E-02 | 0.040527 | 0.630862 | H200004013 | KIRREL3 | kin of IRRE like 3 (Drosophila) | 84623 |
| 8561 | -2.184 | 0.270565 | 9.05E-03 | 0.033342 | 0.630983 | H200016070 | GPR25 | G protein-coupled receptor 25 | 2848 |
| 4555 | -2.6346 | 0.127873 | 2.58E-03 | 0.01733 | 0.631047 | H200021118 | NA | NA | - |
| 6976 | -1.9229 | 0.215875 | 1.91E-02 | 0.050171 | 0.631057 | H200005699 | LAMC3 | laminin, gamma 3 | 10319 |
| 12660 | -2.749 | 0.161436 | 1.90E-03 | 0.015105 | 0.631095 | H200015434 | WBP2 | WW domain binding protein 2 | 23558 |
| 11958 | -2.3194 | 0.213675 | 6.16E-03 | 0.026737 | 0.631183 | H200003583 | C6orf106 | chromosome 6 open reading frame 106 | 64771 |
| 17710 | -2.9103 | 0.190911 | 1.25E-03 | 0.012276 | 0.631502 | H200016353 | VWA5B2 | von Willebrand factor A domain containing 5B2 | 90113 |
| 1676 | -2.4863 | 0.15986 | 3.85E-03 | 0.021209 | 0.631638 | H200014677 | DIEXF | digestive organ expansion factor homolog (zebrafish) | 27042 |
| 15373 | -2.1497 | 0.310979 | 9.99E-03 | 0.035224 | 0.631749 | H200013919 | TNK2 | tyrosine kinase, non-receptor, 2 | 10188 |
| 6902 | -2.4549 | 0.203793 | 4.21E-03 | 0.022146 | 0.631786 | H200002255 | ZNF574 | zinc finger protein 574 | 64763 |
| 4337 | -1.7964 | 0.235494 | 2.74E-02 | 0.061772 | 0.631876 | H200010834 | LINC00176 | long intergenic non-protein coding RNA 176 | 284739 |
| 15824 | -1.8461 | 0.302667 | 2.38E-02 | 0.056872 | 0.632002 | H200013486 | GNMT | glycine N-methyltransferase | 27232 |
| 16024 | -2.8772 | 0.172684 | 1.36E-03 | 0.012768 | 0.632153 | H200001421 | BTBD6 | BTB (POZ) domain containing 6 | 90135 |
| 15190 | -2.4775 | 0.183109 | 3.95E-03 | 0.021506 | 0.632166 | H200005185 | SOLH | small optic lobes homolog (Drosophila) | 6650 |
| 11045 | -1.9964 | 0.19425 | 1.55E-02 | 0.044828 | 0.632463 | H200003565 | BTBD2 | BTB (POZ) domain containing 2 | 55643 |
| 19547 | -2.222 | 0.218129 | 8.14E-03 | 0.031324 | 0.632507 | H200000588 | CHRNB2 | cholinergic receptor, nicotinic, beta 2 (neuronal) | 1141 |
| 15431 | -2.6893 | 0.143978 | 2.23E-03 | 0.016186 | 0.632654 | H200016603 | KBTBD4 | kelch repeat and BTB (POZ) domain containing 4 | 55709 |
| 6026 | -1.875 | 0.308396 | 2.19E-02 | 0.054162 | 0.632858 | H200004143 | XYLT2 | xylosyltransferase II | 64132 |
| 5702 | -3.0108 | 0.130497 | 9.71E-04 | 0.01101 | 0.633033 | H200010318 | PTGFR | prostaglandin F receptor (FP) | 5737 |
| 13742 | -2.6251 | 0.255795 | 2.64E-03 | 0.017539 | 0.633255 | H200001540 | DNAJB12 | DnaJ (Hsp40) homolog, subfamily B, member 12 | 54788 |
| 21395 | -2.0603 | 0.1292 | 1.29E-02 | 0.040229 | 0.633257 | H200006763 | BRE | brain and reproductive organ-expressed (TNFRSF1A modulator) | 9577 |
| 226 | -1.7957 | 0.298256 | 2.74E-02 | 0.061815 | 0.63399 | H200010746 | RHOF | ras homolog family member F (in filopodia) | 54509 |
| 12892 | -2.6707 | 0.147312 | 2.34E-03 | 0.016608 | 0.634 | H200004592 | NA | NA | - |
| 15730 | -2.8707 | 0.164596 | 1.39E-03 | 0.012895 | 0.634068 | H200009234 | PHF23 | PHD finger protein 23 | 79142 |
| 1744 | -2.2008 | 0.222564 | 8.64E-03 | 0.03241 | 0.634077 | H200017765 | LYPLA2 | lysophospholipase II | 11313 |
| 21367 | -1.8085 | 0.304307 | 2.65E-02 | 0.060535 | 0.634128 | H200005291 | FARS2 | phenylalanyl-tRNA synthetase 2, mitochondrial | 10667 |
| 10546 | -2.6303 | 0.18782 | 2.61E-03 | 0.017425 | 0.63413 | H200001826 | NFKBIB | nuclear factor of kappa light polypeptide gene enhancer in B-cells inhibitor, beta | 4793 |
| 15312 | -2.6298 | 0.164679 | 2.61E-03 | 0.017425 | 0.634143 | H200010909 | FDXACB1 | ferredoxin-fold anticodon binding domain containing 1 | 91893 |
| 14602 | -2.2936 | 0.213499 | 6.63E-03 | 0.027832 | 0.634303 | H200020825 | NA | NA | - |
| 3436 | -2.2015 | 0.212505 | 8.62E-03 | 0.032386 | 0.634331 | H200011352 | AP5Z1 | adaptor-related protein complex 5, zeta 1 subunit | 9907 |
| 5965 | -2.9075 | 0.124453 | 1.26E-03 | 0.012286 | 0.634374 | H200001429 | RIC8A | resistance to inhibitors of cholinesterase 8 homolog A (C. elegans) | 60626 |
| 10147 | -2.7341 | 0.127765 | 1.98E-03 | 0.015394 | 0.634602 | H200004267 | CERK | ceramide kinase | 64781 |
| 15797 | -2.966 | 0.164516 | 1.08E-03 | 0.011546 | 0.63468 | H200012316 | SLC6A9 | solute carrier family 6 (neurotransmitter transporter, glycine), member 9 | 6536 |
| 20474 | -2.4843 | 0.210848 | 3.88E-03 | 0.021299 | 0.63522 | H200004163 | EBF1 | early B-cell factor 1 | 1879 |
| 5566 | -2.0836 | 0.267976 | 1.21E-02 | 0.038948 | 0.63523 | H200003858 | HYAL4 | hyaluronoglucosaminidase 4 | 23553 |
| 19512 | -2.0549 | 0.210952 | 1.31E-02 | 0.040613 | 0.635917 | H200020319 | CCDC148 | coiled-coil domain containing 148 | 130940 |
| 11356 | -2.3204 | 0.268673 | 6.15E-03 | 0.026703 | 0.636263 | H200018367 | PQLC3 | PQ loop repeat containing 3 | 130814 |
| 18857 | -2.1189 | 0.227751 | 1.09E-02 | 0.037061 | 0.6364 | H200009696 | NA | NA | - |
| 12030 | -2.7086 | 0.179698 | 2.12E-03 | 0.01593 | 0.636465 | H200007003 | FAM134C | family with sequence similarity 134, member C | 162427 |
| 8350 | -2.0315 | 0.325809 | 1.40E-02 | 0.04233 | 0.636473 | H200005864 | GNA15 | guanine nucleotide binding protein (G protein), alpha 15 (Gq class) | 2769 |
| 15392 | -2.143 | 0.309833 | 1.02E-02 | 0.035612 | 0.636475 | H200014709 | RPS27 | ribosomal protein S27 | 6232 |
| 10766 | -1.9967 | 0.255875 | 1.55E-02 | 0.044807 | 0.636757 | H200012134 | ABCB8 | ATP-binding cassette, sub-family B (MDR/TAP), member 8 | 11194 |
| 21589 | -1.977 | 0.48903 | 1.64E-02 | 0.046087 | 0.636762 | H200015907 | NA | NA | - |
| 5946 | -2.2329 | 0.264965 | 7.89E-03 | 0.030913 | 0.636887 | H200000343 | TNFSF8 | tumor necrosis factor (ligand) superfamily, member 8 | 944 |
| 17319 | -2.2342 | 0.199626 | 7.86E-03 | 0.030843 | 0.636924 | H200019672 | YIF1B | Yip1 interacting factor homolog B (S. cerevisiae) | 90522 |
| 20194 | -2.3547 | 0.147756 | 5.57E-03 | 0.025432 | 0.636947 | H200010694 | DDX59 | DEAD (Asp-Glu-Ala-Asp) box polypeptide 59 | 83479 |
| 6860 | -2.0703 | 0.237091 | 1.25E-02 | 0.039613 | 0.636972 | H200000331 | C6 | complement component 6 | 729 |
| 1429 | -2.0826 | 0.332701 | 1.21E-02 | 0.038981 | 0.63731 | H200002915 | NA | NA | - |
| 18808 | -1.9172 | 0.271531 | 1.94E-02 | 0.050697 | 0.637313 | H200007036 | HOXD1 | homeobox D1 | 3231 |
| 10277 | -2.4278 | 0.107044 | 4.53E-03 | 0.022795 | 0.637426 | H200010655 | NCDN | neurochondrin | 23154 |
| 14633 | -2.5865 | 0.173638 | 2.94E-03 | 0.018445 | 0.63744 | H200000476 | PDE6G | phosphodiesterase 6G, cGMP-specific, rod, gamma | 5148 |
| 21031 | -2.11 | 0.254161 | 1.12E-02 | 0.037549 | 0.637534 | H200011038 | CBL | Cbl proto-oncogene, E3 ubiquitin protein ligase | 867 |
| 3297 | -2.0823 | 0.191798 | 1.21E-02 | 0.038981 | 0.637552 | H200004862 | GOLGA2P5 | golgin A2 pseudogene 5 | 55592 |
| 13837 | -1.8415 | 0.234681 | 2.41E-02 | 0.057239 | 0.637733 | H200006094 | MANF | mesencephalic astrocyte-derived neurotrophic factor | 7873 |
| 16529 | -1.991 | 0.071635 | 1.57E-02 | 0.045068 | 0.637874 | H200003801 | CEP250 | centrosomal protein 250kDa | 11190 |
| 17715 | -1.8331 | 0.291489 | 2.47E-02 | 0.05798 | 0.637933 | H200016407 | ARFGAP2 | ADP-ribosylation factor GTPase activating protein 2 | 84364 |
| 11460 | -2.6543 | 0.146254 | 2.45E-03 | 0.01695 | 0.638006 | H200001742 | NA | NA | - |
| 9778 | -1.9053 | 0.268736 | 2.01E-02 | 0.051626 | 0.638138 | H200008702 | NA | NA | - |
| 9258 | -1.8409 | 0.26677 | 2.41E-02 | 0.057322 | 0.638596 | H200005709 | MTRF1L | mitochondrial translational release factor 1-like | 54516 |
| 11513 | -3.0626 | 0.15702 | 8.53E-04 | 0.010432 | 0.638637 | H200004372 | INSL3 | insulin-like 3 (Leydig cell) | 3640 |
| 21176 | -2.1543 | 0.195017 | 9.87E-03 | 0.034967 | 0.638758 | H200017896 | NA | NA | - |
| 18776 | -2.3887 | 0.170279 | 5.05E-03 | 0.024149 | 0.638928 | H200005178 | C1orf50 | chromosome 1 open reading frame 50 | 79078 |
| 11865 | -1.9034 | 0.340688 | 2.02E-02 | 0.051854 | 0.639679 | H200021092 | TIGD1 | tigger transposable element derived 1 | 200765 |
| 13499 | -2.405 | 0.211386 | 4.83E-03 | 0.023541 | 0.639705 | H200011759 | ROBO4 | roundabout, axon guidance receptor, homolog 4 (Drosophila) | 54538 |
| 1469 | -2.3571 | 0.18054 | 5.53E-03 | 0.025347 | 0.639805 | H200004815 | PRKCQ-AS1 | PRKCQ antisense RNA 1 | 439949 |
| 21476 | -1.7806 | 0.285577 | 2.87E-02 | 0.063452 | 0.63986 | H200010581 | SNRNP35 | small nuclear ribonucleoprotein 35kDa (U11/U12) | 11066 |
| 21373 | -2.2332 | 0.179575 | 7.89E-03 | 0.030913 | 0.639903 | H200005647 | GTF2F1 | general transcription factor IIF, polypeptide 1, 74kDa | 2962 |
| 6510 | -2.0775 | 0.232471 | 1.23E-02 | 0.039356 | 0.639932 | H200005342 | YBEY | ybeY metallopeptidase (putative) | 54059 |
| 10483 | -2.6499 | 0.221003 | 2.47E-03 | 0.017018 | 0.640214 | H200020227 | NA | NA | - |
| 15132 | -3.5185 | 0.09663 | 2.99E-04 | 0.007425 | 0.640322 | H200002501 | IER5 | immediate early response 5 | 51278 |
| 8467 | -2.2129 | 0.219977 | 8.35E-03 | 0.031778 | 0.640493 | H200011534 | YIPF3 | Yip1 domain family, member 3 | 25844 |
| 8861 | -2.1944 | 0.122094 | 8.79E-03 | 0.032682 | 0.64056 | H200008613 | BAIAP2-AS1 | BAIAP2 antisense RNA 1 (head to head) | 440465 |
| 13300 | -1.9794 | 0.263523 | 1.62E-02 | 0.045857 | 0.640586 | H200002265 | TUBGCP2 | tubulin, gamma complex associated protein 2 | 10844 |
| 11176 | -2.0322 | 0.142766 | 1.40E-02 | 0.042307 | 0.640821 | H200009675 | DLST | dihydrolipoamide S-succinyltransferase (E2 component of 2-oxo-glutarate complex) | 1743 |
| 10490 | -2.6139 | 0.158115 | 2.73E-03 | 0.017762 | 0.640825 | H200020589 | PCED1B | PC-esterase domain containing 1B | 91523 |
| 17943 | -1.8732 | 0.266536 | 2.20E-02 | 0.054303 | 0.640857 | H200005672 | IHH | indian hedgehog | 3549 |
| 11467 | -2.7177 | 0.157624 | 2.06E-03 | 0.015694 | 0.640946 | H200002116 | ZNF48 | zinc finger protein 48 | 197407 |
| 1176 | -1.8306 | 0.220409 | 2.48E-02 | 0.058249 | 0.641392 | H200012350 | GTF2A1 | general transcription factor IIA, 1, 19/37kDa | 2957 |
| 425 | -2.1388 | 0.175558 | 1.03E-02 | 0.035849 | 0.641442 | H200020240 | GSTM4 | glutathione S-transferase mu 4 | 2948 |
| 4870 | -1.9855 | 0.22806 | 1.60E-02 | 0.045424 | 0.64154 | H200014225 | RANGAP1 | Ran GTPase activating protein 1 | 5905 |
| 14744 | -2.1543 | 0.214328 | 9.87E-03 | 0.034967 | 0.641644 | H200005494 | KREMEN1 | kringle containing transmembrane protein 1 | 83999 |
| 17859 | -2.2253 | 0.291831 | 8.06E-03 | 0.031187 | 0.641718 | H200001824 | PHF15 | PHD finger protein 15 | 23338 |
| 11773 | -2.5662 | 0.153296 | 3.10E-03 | 0.018915 | 0.641764 | H200016580 | SSBP3 | single stranded DNA binding protein 3 | 23648 |
| 12617 | -2.112 | 0.21212 | 1.11E-02 | 0.037425 | 0.641841 | H200013504 | PODXL2 | podocalyxin-like 2 | 50512 |
| 15790 | -2.2653 | 0.239532 | 7.19E-03 | 0.029367 | 0.641946 | H200011942 | NA | NA | - |
| 232 | -2.6975 | 0.08155 | 2.18E-03 | 0.016125 | 0.642258 | H200010818 | SH3GL1 | SH3-domain GRB2-like 1 | 6455 |
| 15354 | -2.7619 | 0.05972 | 1.83E-03 | 0.014822 | 0.642344 | H200013117 | ABCA7 | ATP-binding cassette, sub-family A (ABC1), member 7 | 10347 |
| 428 | -2.1956 | 0.187606 | 8.76E-03 | 0.03265 | 0.642697 | H200020270 | PLCD4 | phospholipase C, delta 4 | 84812 |
| 19255 | -2.1747 | 0.077787 | 9.30E-03 | 0.033887 | 0.642727 | H200008141 | GBA2 | glucosidase, beta (bile acid) 2 | 57704 |
| 6427 | -1.9489 | 0.270056 | 1.77E-02 | 0.048288 | 0.642744 | H200001228 | CPSF3L | cleavage and polyadenylation specific factor 3-like | 54973 |
| 5157 | -1.8149 | 0.1184 | 2.60E-02 | 0.059961 | 0.642748 | H200006334 | ID3 | inhibitor of DNA binding 3, dominant negative helix-loop-helix protein | 3399 |
| 15700 | -2.9775 | 0.164538 | 1.05E-03 | 0.011443 | 0.642884 | H200007738 | GPR108 | G protein-coupled receptor 108 | 56927 |
| 11954 | -1.942 | 0.180369 | 1.81E-02 | 0.048809 | 0.642991 | H200003535 | PTPN23 | protein tyrosine phosphatase, non-receptor type 23 | 25930 |
| 16673 | -2.2153 | 0.209416 | 8.29E-03 | 0.031626 | 0.643056 | H200010641 | NA | NA | - |
| 8056 | -2.9303 | 0.160729 | 1.19E-03 | 0.012039 | 0.643389 | H200013571 | MYH9 | myosin, heavy chain 9, non-muscle | 4627 |
| 6234 | -1.8285 | 0.197436 | 2.50E-02 | 0.058444 | 0.64355 | H200014023 | TMEM74B | transmembrane protein 74B | 55321 |
| 10573 | -2.2448 | 0.151401 | 7.63E-03 | 0.030457 | 0.643747 | H200003008 | NAGPA | N-acetylglucosamine-1-phosphodiester alpha-N-acetylglucosaminidase | 51172 |
| 11147 | -1.9292 | 0.277649 | 1.87E-02 | 0.049699 | 0.643788 | H200008481 | NCAPH2 | non-SMC condensin II complex, subunit H2 | 29781 |
| 2580 | -1.7941 | 0.180571 | 2.76E-02 | 0.061983 | 0.644061 | H200014048 | HNRNPUL1 | heterogeneous nuclear ribonucleoprotein U-like 1 | 11100 |
| 21603 | -1.9241 | 0.264255 | 1.90E-02 | 0.050108 | 0.644136 | H200016643 | GPS1 | G protein pathway suppressor 1 | 2873 |
| 16508 | -2.7558 | 0.131701 | 1.86E-03 | 0.01495 | 0.64433 | H200002691 | WDR46 | WD repeat domain 46 | 9277 |
| 15111 | -1.8418 | 0.281992 | 2.40E-02 | 0.057239 | 0.64434 | H200001403 | NA | NA | - |
| 5987 | -1.8974 | 0.112228 | 2.05E-02 | 0.052269 | 0.644556 | H200002261 | LANCL1 | LanC lantibiotic synthetase component C-like 1 (bacterial) | 10314 |
| 19140 | -1.9031 | 0.344563 | 2.02E-02 | 0.051874 | 0.644653 | H200002791 | ZDHHC18 | zinc finger, DHHC-type containing 18 | 84243 |
| 21101 | -2.076 | 0.270588 | 1.23E-02 | 0.039392 | 0.644697 | H200014150 | CDK5R2 | cyclin-dependent kinase 5, regulatory subunit 2 (p39) | 8941 |
| 17661 | -2.4193 | 0.131934 | 4.64E-03 | 0.023059 | 0.644757 | H200013771 | NA | NA | - |
| 6458 | -2.17 | 0.249682 | 9.42E-03 | 0.034137 | 0.644859 | H200002730 | FAM167B | family with sequence similarity 167, member B | 84734 |
| 9644 | -1.9007 | 0.311837 | 2.03E-02 | 0.0521 | 0.644982 | H200002266 | XKR4 | XK, Kell blood group complex subunit-related family, member 4 | 114786 |
| 4971 | -1.9855 | 0.205138 | 1.60E-02 | 0.045424 | 0.645022 | H200019135 | LOC284600 | uncharacterized LOC284600 | 284600 |
| 28 | -2.7596 | 0.124603 | 1.85E-03 | 0.014855 | 0.645035 | H200001270 | KIF5C | kinesin family member 5C | 3800 |
| 21657 | -2.3961 | 0.067441 | 4.95E-03 | 0.023913 | 0.64505 | H200018995 | RPUSD4 | RNA pseudouridylate synthase domain containing 4 | 84881 |
| 17614 | -2.205 | 0.167144 | 8.53E-03 | 0.032185 | 0.645109 | H200011793 | TGIF2LY | TGFB-induced factor homeobox 2-like, Y-linked | 90655 |
| 438 | -2.276 | 0.12572 | 6.97E-03 | 0.028705 | 0.645146 | H200020674 | NA | NA | - |
| 15820 | -2.3041 | 0.216896 | 6.43E-03 | 0.027337 | 0.645174 | H200013438 | NA | NA | 400688 |
| 12279 | -2.3759 | 0.16596 | 5.24E-03 | 0.024577 | 0.645211 | H200018801 | NA | NA | - |
| 19426 | -2.0064 | 0.228187 | 1.50E-02 | 0.043974 | 0.645226 | H200016447 | NA | NA | - |
| 18108 | -2.0836 | 0.262417 | 1.21E-02 | 0.038948 | 0.645285 | H200013622 | CACNG1 | calcium channel, voltage-dependent, gamma subunit 1 | 786 |
| 21002 | -1.9342 | 0.248807 | 1.85E-02 | 0.049361 | 0.645287 | H200009560 | ST7L | suppression of tumorigenicity 7 like | 54879 |
| 15083 | -2.1769 | 0.217271 | 9.24E-03 | 0.033755 | 0.645296 | H200000215 | HOXC6 | homeobox C6 | 3223 |
| 4454 | -2.8161 | 0.197018 | 1.60E-03 | 0.013811 | 0.645382 | H200016208 | EMC10 | ER membrane protein complex subunit 10 | 284361 |
| 8450 | -2.1122 | 0.138519 | 1.11E-02 | 0.037421 | 0.645397 | H200010756 | NA | NA | - |
| 19142 | -2.1232 | 0.222627 | 1.08E-02 | 0.036874 | 0.645514 | H200002815 | GPN2 | GPN-loop GTPase 2 | 54707 |
| 16725 | -1.9708 | 0.228919 | 1.66E-02 | 0.046574 | 0.645619 | H200012969 | TAF6L | TAF6-like RNA polymerase II, p300/CBP-associated factor (PCAF)-associated factor, 65kDa | 10629 |
| 11253 | -1.9001 | 0.270282 | 2.04E-02 | 0.052126 | 0.645639 | H200013445 | LONRF3 | LON peptidase N-terminal domain and ring finger 3 | 79836 |
| 16820 | -3.1049 | 0.123325 | 7.70E-04 | 0.010006 | 0.646078 | H200017511 | GPR132 | G protein-coupled receptor 132 | 29933 |
| 15481 | -2.588 | 0.147602 | 2.92E-03 | 0.018418 | 0.64609 | H200019191 | ULBP3 | UL16 binding protein 3 | 79465 |
| 10611 | -1.9678 | 0.261606 | 1.68E-02 | 0.046879 | 0.646453 | H200004884 | MRPL53 | mitochondrial ribosomal protein L53 | 116540 |
| 18423 | -1.8011 | 0.1464 | 2.70E-02 | 0.06141 | 0.646546 | H200007719 | POR | P450 (cytochrome) oxidoreductase | 5447 |
| 3989 | -2.37 | 0.17455 | 5.33E-03 | 0.024789 | 0.646646 | H200015727 | IL1RAPL1 | interleukin 1 receptor accessory protein-like 1 | 11141 |
| 265 | -2.7065 | 0.165986 | 2.13E-03 | 0.015941 | 0.646822 | H200012640 | NA | NA | - |
| 14251 | -2.7345 | 0.147993 | 1.98E-03 | 0.015394 | 0.646861 | H200004123 | PPP1R37 | protein phosphatase 1, regulatory subunit 37 | 284352 |
| 11839 | -1.9021 | 0.212616 | 2.02E-02 | 0.051949 | 0.64713 | H200019644 | VPS26B | vacuolar protein sorting 26 homolog B (S. pombe) | 112936 |
| 193 | -1.815 | 0.239624 | 2.60E-02 | 0.059961 | 0.647131 | H200009220 | PTGES2 | prostaglandin E synthase 2 | 80142 |
| 19246 | -2.5486 | 0.168462 | 3.26E-03 | 0.019458 | 0.647162 | H200007755 | IL15 | interleukin 15 | 3600 |
| 18020 | -1.9221 | 0.272428 | 1.91E-02 | 0.050251 | 0.647184 | H200009442 | NA | NA | - |
| 13906 | -2.2443 | 0.241734 | 7.64E-03 | 0.030457 | 0.647363 | H200009188 | NA | NA | 343574 |
| 18021 | -2.2057 | 0.135107 | 8.52E-03 | 0.032145 | 0.647432 | H200009448 | LTBP3 | latent transforming growth factor beta binding protein 3 | 4054 |
| 6451 | -1.7962 | 0.247723 | 2.74E-02 | 0.061772 | 0.647468 | H200002368 | NA | NA | - |
| 4663 | -2.102 | 0.20636 | 1.14E-02 | 0.03795 | 0.647576 | H200004363 | HOXA5 | homeobox A5 | 3202 |
| 15034 | -2.0421 | 0.274425 | 1.36E-02 | 0.041577 | 0.647704 | H200019482 | WIBG | within bgcn homolog (Drosophila) | 84305 |
| 3729 | -2.025 | 0.19047 | 1.43E-02 | 0.042773 | 0.647833 | H200003519 | PSEN2 | presenilin 2 (Alzheimer disease 4) | 5664 |
| 6704 | -1.7922 | 0.158305 | 2.77E-02 | 0.062235 | 0.647839 | H200014486 | ZNF34 | zinc finger protein 34 | 80778 |
| 16625 | -2.2579 | 0.218926 | 7.34E-03 | 0.029785 | 0.648182 | H200008361 | SPATA6L | spermatogenesis associated 6-like | 55064 |
| 15151 | -2.9215 | 0.131289 | 1.21E-03 | 0.012146 | 0.648215 | H200003303 | DCUN1D2 | DCN1, defective in cullin neddylation 1, domain containing 2 (S. cerevisiae) | 55208 |
| 13577 | -1.959 | 0.248928 | 1.72E-02 | 0.047476 | 0.648249 | H200015535 | FICD | FIC domain containing | 11153 |
| 18710 | -2.0756 | 0.210322 | 1.23E-02 | 0.039392 | 0.648311 | H200001716 | TESK2 | testis-specific kinase 2 | 10420 |
| 9830 | -2.2152 | 0.259893 | 8.30E-03 | 0.031626 | 0.64844 | H200011314 | CHST1 | carbohydrate (keratan sulfate Gal-6) sulfotransferase 1 | 8534 |
| 16346 | -1.9352 | 0.359577 | 1.84E-02 | 0.049314 | 0.648555 | H200016929 | NA | NA | - |
| 1014 | -2.4352 | 0.174903 | 4.44E-03 | 0.022526 | 0.648582 | H200004726 | ZKSCAN4 | zinc finger with KRAB and SCAN domains 4 | 387032 |
| 17678 | -1.9009 | 0.345056 | 2.03E-02 | 0.052088 | 0.649128 | H200014833 | JUNB | jun B proto-oncogene | 3726 |
| 6883 | -2.2391 | 0.189053 | 7.75E-03 | 0.030653 | 0.649198 | H200001465 | FES | feline sarcoma oncogene | 2242 |
| 10305 | -2.5404 | 0.132446 | 3.33E-03 | 0.019619 | 0.649328 | H200011843 | SCNN1D | sodium channel, non-voltage-gated 1, delta subunit | 6339 |
| 15435 | -1.7767 | 0.225004 | 2.90E-02 | 0.063749 | 0.649579 | H200016935 | PADI1 | peptidyl arginine deiminase, type I | 29943 |
| 16226 | -3.0667 | 0.129522 | 8.45E-04 | 0.010421 | 0.649623 | H200011229 | ELMSAN1 | ELM2 and Myb/SANT-like domain containing 1 | 91748 |
| 2928 | -1.8715 | 0.255933 | 2.21E-02 | 0.054414 | 0.649654 | H200008787 | BTN3A1 | butyrophilin, subfamily 3, member A1 | 11119 |
| 17090 | -2.0698 | 0.22338 | 1.25E-02 | 0.039615 | 0.649692 | H200008694 | NA | NA | - |
| 10188 | -2.0355 | 0.247194 | 1.38E-02 | 0.042034 | 0.649786 | H200006173 | PRCP | prolylcarboxypeptidase (angiotensinase C) | 5547 |
| 19613 | -2.787 | 0.149189 | 1.72E-03 | 0.0143 | 0.649793 | H200003652 | SHKBP1 | SH3KBP1 binding protein 1 | 92799 |
| 19541 | -2.4945 | 0.159151 | 3.77E-03 | 0.020941 | 0.649847 | H200000232 | MAPK3 | mitogen-activated protein kinase 3 | 5595 |
| 5621 | -2.1876 | 0.162135 | 8.96E-03 | 0.033126 | 0.650389 | H200006512 | SCAP | SREBF chaperone | 22937 |
| 5643 | -1.8083 | 0.274852 | 2.65E-02 | 0.060545 | 0.650566 | H200007628 | DIRAS2 | DIRAS family, GTP-binding RAS-like 2 | 54769 |
| 9352 | -2.2289 | 0.212788 | 7.98E-03 | 0.031081 | 0.650751 | H200009961 | FBXW4P1 | F-box and WD repeat domain containing 4 pseudogene 1 | 26226 |
| 19330 | -2.336 | 0.167007 | 5.87E-03 | 0.026206 | 0.650829 | H200011887 | LOC100499484-C9ORF174 | LOC100499484-C9orf174 readthrough | 57653 |
| 14829 | -2.2937 | 0.222904 | 6.63E-03 | 0.027832 | 0.651066 | H200009644 | SHARPIN | SHANK-associated RH domain interactor | 81858 |
| 11125 | -1.8691 | 0.258679 | 2.23E-02 | 0.05464 | 0.651083 | H200007365 | IPO13 | importin 13 | 9670 |
| 20185 | -1.8942 | 0.133787 | 2.07E-02 | 0.052485 | 0.651582 | H200010290 | MSL3 | male-specific lethal 3 homolog (Drosophila) | 10943 |
| 13744 | -2.3773 | 0.192718 | 5.22E-03 | 0.024533 | 0.652108 | H200001564 | DOCK9 | dedicator of cytokinesis 9 | 23348 |
| 21417 | -1.8491 | 0.208855 | 2.36E-02 | 0.05661 | 0.652162 | H200007595 | VPS9D1 | VPS9 domain containing 1 | 9605 |
| 18552 | -2.4624 | 0.140587 | 4.12E-03 | 0.022023 | 0.652262 | H200014589 | C2orf54 | chromosome 2 open reading frame 54 | 79919 |
| 19509 | -2.9503 | 0.113281 | 1.12E-03 | 0.011725 | 0.652433 | H200020277 | SLC48A1 | solute carrier family 48 (heme transporter), member 1 | 55652 |
| 893 | -2.0812 | 0.168787 | 1.21E-02 | 0.039035 | 0.652657 | H200020763 | RHBDF1 | rhomboid 5 homolog 1 (Drosophila) | 64285 |
| 17902 | -2.1028 | 0.201567 | 1.14E-02 | 0.037908 | 0.653474 | H200003766 | NA | NA | - |
| 495 | -1.9787 | 0.207268 | 1.63E-02 | 0.045919 | 0.65349 | H200001787 | NA | NA | - |
| 14505 | -1.9119 | 0.195112 | 1.97E-02 | 0.051137 | 0.653731 | H200016259 | C12orf52 | chromosome 12 open reading frame 52 | 84934 |
| 10654 | -2.7025 | 0.127341 | 2.15E-03 | 0.015982 | 0.653835 | H200006814 | C7orf26 | chromosome 7 open reading frame 26 | 79034 |
| 939 | -1.9574 | 0.22735 | 1.73E-02 | 0.047622 | 0.653881 | H200001276 | NA | NA | - |
| 4582 | -2.8518 | 0.131376 | 1.46E-03 | 0.013131 | 0.654025 | H200000545 | RORA | RAR-related orphan receptor A | 6095 |
| 4664 | -2.0252 | 0.179786 | 1.42E-02 | 0.042764 | 0.654089 | H200004369 | EFNA3 | ephrin-A3 | 1944 |
| 8335 | -2.5516 | 0.157687 | 3.23E-03 | 0.019403 | 0.654138 | H200005122 | B4GALT7 | xylosylprotein beta 1,4-galactosyltransferase, polypeptide 7 | 11285 |
| 20308 | -1.8534 | 0.295907 | 2.33E-02 | 0.056194 | 0.654168 | H200016798 | CDH7 | cadherin 7, type 2 | 1005 |
| 3682 | -1.863 | 0.145912 | 2.26E-02 | 0.055218 | 0.654347 | H200001245 | GABARAPL2 | GABA(A) receptor-associated protein-like 2 | 11345 |
| 6013 | -2.1533 | 0.196883 | 9.89E-03 | 0.034998 | 0.654525 | H200003709 | RUSC2 | RUN and SH3 domain containing 2 | 9853 |
| 14609 | -2.3793 | 0.093779 | 5.19E-03 | 0.024471 | 0.654741 | H200021199 | C5orf45 | chromosome 5 open reading frame 45 | 51149 |
| 4588 | -2.1551 | 0.212374 | 9.85E-03 | 0.034916 | 0.654969 | H200000901 | KIAA1239 | KIAA1239 | 57495 |
| 20833 | -2.6223 | 0.118478 | 2.67E-03 | 0.017625 | 0.654974 | H200001562 | DMAP1 | DNA methyltransferase 1 associated protein 1 | 55929 |
| 19200 | -2.1239 | 0.155258 | 1.08E-02 | 0.03684 | 0.655077 | H200005499 | DEF8 | differentially expressed in FDCP 8 homolog (mouse) | 54849 |
| 14207 | -2.0105 | 0.197365 | 1.49E-02 | 0.043702 | 0.655189 | H200001891 | ABCG1 | ATP-binding cassette, sub-family G (WHITE), member 1 | 9619 |
| 16938 | -2.3911 | 0.322014 | 5.02E-03 | 0.02407 | 0.65535 | H200001474 | GABARAP | GABA(A) receptor-associated protein | 11337 |
| 18597 | -2.4276 | 0.115004 | 4.54E-03 | 0.022799 | 0.655386 | H200017201 | HDAC7 | histone deacetylase 7 | 51564 |
| 11015 | -3.8504 | 0.092055 | 1.50E-04 | 0.005903 | 0.655615 | H200002069 | CHMP2A | charged multivesicular body protein 2A | 27243 |
| 12676 | -1.8098 | 0.160888 | 2.64E-02 | 0.060372 | 0.655945 | H200016194 | CBX8 | chromobox homolog 8 | 57332 |
| 14102 | -2.3799 | 0.240165 | 5.18E-03 | 0.024463 | 0.65611 | H200018640 | NA | NA | - |
| 13618 | -2.3416 | 0.229754 | 5.78E-03 | 0.02598 | 0.656115 | H200017441 | MYT1 | myelin transcription factor 1 | 4661 |
| 16891 | -2.3464 | 0.135756 | 5.70E-03 | 0.025809 | 0.656142 | H200020925 | HAGHL | hydroxyacylglutathione hydrolase-like | 84264 |
| 2307 | -2.4209 | 0.163468 | 4.62E-03 | 0.023023 | 0.656356 | H200001122 | VAC14 | Vac14 homolog (S. cerevisiae) | 55697 |
| 14367 | -2.001 | 0.15464 | 1.53E-02 | 0.044437 | 0.656582 | H200009491 | FAM3A | family with sequence similarity 3, member A | 60343 |
| 17023 | -2.1882 | 0.151641 | 8.95E-03 | 0.03309 | 0.656762 | H200005612 | RNF4 | ring finger protein 4 | 6047 |
| 6577 | -2.239 | 0.201189 | 7.75E-03 | 0.030653 | 0.656832 | H200008424 | RBBP8NL | RBBP8 N-terminal like | 140893 |
| 5486 | -2.1297 | 0.199746 | 1.06E-02 | 0.036434 | 0.656835 | H200000058 | GCGR | glucagon receptor | 2642 |
| 8173 | -2.0123 | 0.267597 | 1.48E-02 | 0.043631 | 0.656884 | H200019241 | NA | NA | 85374 |
| 3849 | -1.8245 | 0.159187 | 2.53E-02 | 0.058952 | 0.657113 | H200009219 | NA | NA | - |
| 2320 | -1.7898 | 0.262368 | 2.79E-02 | 0.062535 | 0.657125 | H200001840 | XAB2 | XPA binding protein 2 | 56949 |
| 18348 | -2.6051 | 0.139197 | 2.79E-03 | 0.018002 | 0.657322 | H200003563 | SF3B4 | splicing factor 3b, subunit 4, 49kDa | 10262 |
| 9636 | -2.83 | 0.110118 | 1.54E-03 | 0.013569 | 0.657322 | H200001886 | SMG9 | smg-9 homolog, nonsense mediated mRNA decay factor (C. elegans) | 56006 |
| 11905 | -2.5856 | 0.165907 | 2.94E-03 | 0.01846 | 0.657362 | H200001249 | SLC17A7 | solute carrier family 17 (sodium-dependent inorganic phosphate cotransporter), member 7 | 57030 |
| 21021 | -1.9184 | 0.231545 | 1.93E-02 | 0.050564 | 0.657364 | H200010350 | IDUA | iduronidase, alpha-L- | 3425 |
| 6774 | -1.9466 | 0.215409 | 1.78E-02 | 0.04839 | 0.65758 | H200017882 | NA | NA | - |
| 17867 | -2.7339 | 0.16449 | 1.98E-03 | 0.015394 | 0.657681 | H200002204 | MCOLN1 | mucolipin 1 | 57192 |
| 20035 | -1.8939 | 0.22366 | 2.07E-02 | 0.052515 | 0.657912 | H200001978 | RBM14 | RNA binding motif protein 14 | 10432 |
| 21268 | -2.4425 | 0.177371 | 4.36E-03 | 0.022348 | 0.658181 | H200000701 | PPP4C | protein phosphatase 4, catalytic subunit | 5531 |
| 743 | -2.1187 | 0.235351 | 1.09E-02 | 0.037076 | 0.65825 | H200013567 | NA | NA | - |
| 10212 | -2.1219 | 0.14655 | 1.08E-02 | 0.036901 | 0.658515 | H200007313 | CHD5 | chromodomain helicase DNA binding protein 5 | 26038 |
| 661 | -2.1117 | 0.157535 | 1.11E-02 | 0.037429 | 0.658553 | H200009743 | NA | NA | - |
| 15156 | -2.5896 | 0.09583 | 2.91E-03 | 0.0184 | 0.658567 | H200003641 | TPCN1 | two pore segment channel 1 | 53373 |
| 15875 | -1.9185 | 0.187428 | 1.93E-02 | 0.050564 | 0.658712 | H200016092 | NEUROG1 | neurogenin 1 | 4762 |
| 4905 | -2.1041 | 0.200142 | 1.14E-02 | 0.037866 | 0.658833 | H200016071 | GPR31 | G protein-coupled receptor 31 | 2853 |
| 14804 | -2.4069 | 0.160029 | 4.81E-03 | 0.023484 | 0.658888 | H200008486 | CXXC1 | CXXC finger protein 1 | 30827 |
| 11023 | -1.9427 | 0.196942 | 1.80E-02 | 0.048765 | 0.659 | H200002449 | RHOD | ras homolog family member D | 29984 |
| 2809 | -1.8368 | 0.300696 | 2.44E-02 | 0.057671 | 0.659094 | H200003105 | CDC42SE1 | CDC42 small effector 1 | 56882 |
| 17307 | -2.3323 | 0.139516 | 5.94E-03 | 0.026275 | 0.65916 | H200018960 | B4GALT3 | UDP-Gal:betaGlcNAc beta 1,4- galactosyltransferase, polypeptide 3 | 8703 |
| 3378 | -1.8789 | 0.261356 | 2.16E-02 | 0.053784 | 0.659237 | H200008668 | PRPF31 | PRP31 pre-mRNA processing factor 31 homolog (S. cerevisiae) | 26121 |
| 2294 | -1.9026 | 0.180428 | 2.02E-02 | 0.05191 | 0.659415 | H200000676 | JUND | jun D proto-oncogene | 3727 |
| 6653 | -2.0722 | 0.133284 | 1.25E-02 | 0.039539 | 0.659825 | H200012176 | RAB11FIP3 | RAB11 family interacting protein 3 (class II) | 9727 |
| 8083 | -1.9413 | 0.216237 | 1.81E-02 | 0.048858 | 0.660186 | H200015037 | NA | NA | - |
| 17059 | -2.4337 | 0.157163 | 4.46E-03 | 0.022579 | 0.660362 | H200007180 | FADD | Fas (TNFRSF6)-associated via death domain | 8772 |
| 17599 | -2.225 | 0.165105 | 8.07E-03 | 0.031187 | 0.66049 | H200011039 | ERCC2 | excision repair cross-complementing rodent repair deficiency, complementation group 2 | 2068 |
| 5062 | -1.9648 | 0.1952 | 1.69E-02 | 0.047086 | 0.660614 | H200001780 | VPS39 | vacuolar protein sorting 39 homolog (S. cerevisiae) | 23339 |
| 15723 | -2.2604 | 0.116794 | 7.30E-03 | 0.029671 | 0.660974 | H200008872 | NA | NA | - |
| 18061 | -2.1608 | 0.169043 | 9.68E-03 | 0.034648 | 0.661022 | H200011348 | SH2B2 | SH2B adaptor protein 2 | 10603 |
| 13880 | -1.8453 | 0.145066 | 2.38E-02 | 0.056967 | 0.661335 | H200008024 | PDE4D | phosphodiesterase 4D, cAMP-specific | 5144 |
| 9980 | -2.434 | 0.201488 | 4.46E-03 | 0.022579 | 0.661637 | H200018226 | NA | NA | - |
| 16541 | -1.7964 | 0.315824 | 2.74E-02 | 0.061772 | 0.661642 | H200004229 | ZNF707 | zinc finger protein 707 | 286075 |
| 19087 | -1.9846 | 0.253343 | 1.60E-02 | 0.045471 | 0.661811 | H200000161 | CD40LG | CD40 ligand | 959 |
| 9468 | -2.2382 | 0.235419 | 7.77E-03 | 0.03066 | 0.661901 | H200015613 | HSPB9 | heat shock protein, alpha-crystallin-related, B9 | 94086 |
| 610 | -2.0927 | 0.156283 | 1.18E-02 | 0.038431 | 0.661973 | H200007421 | NA | NA | - |
| 689 | -2.071 | 0.181137 | 1.25E-02 | 0.039577 | 0.662417 | H200011215 | GEMIN7 | gem (nuclear organelle) associated protein 7 | 79760 |
| 15864 | -1.924 | 0.231933 | 1.90E-02 | 0.050108 | 0.662515 | H200015386 | MAST1 | microtubule associated serine/threonine kinase 1 | 22983 |
| 11686 | -1.9903 | 0.190114 | 1.58E-02 | 0.045068 | 0.662665 | H200012406 | TAOK2 | TAO kinase 2 | 9344 |
| 3163 | -2.544 | 0.111146 | 3.30E-03 | 0.01957 | 0.662842 | H200019849 | NA | NA | - |
| 13372 | -2.0318 | 0.141853 | 1.40E-02 | 0.04233 | 0.663051 | H200005685 | RNF126 | ring finger protein 126 | 55658 |
| 20000 | -1.9765 | 0.186825 | 1.64E-02 | 0.046125 | 0.663357 | H200000078 | PLA2G5 | phospholipase A2, group V | 5322 |
| 6069 | -2.0308 | 0.245565 | 1.40E-02 | 0.042356 | 0.663393 | H200006369 | FHIT | fragile histidine triad | 2272 |
| 19925 | -2.1666 | 0.114687 | 9.52E-03 | 0.03439 | 0.663433 | H200018472 | LOC286059 | tumor necrosis factor receptor superfamily, member 10d, decoy with truncated death domain pseudogene | 286059 |
| 13655 | -2.1691 | 0.196952 | 9.45E-03 | 0.034197 | 0.663507 | H200019311 | CYHR1 | cysteine/histidine-rich 1 | 50626 |
| 17153 | -1.8628 | 0.34599 | 2.27E-02 | 0.05522 | 0.663531 | H200011716 | VWF | von Willebrand factor | 7450 |
| 16940 | -2.099 | 0.179074 | 1.15E-02 | 0.038151 | 0.663833 | H200001498 | RHBDD2 | rhomboid domain containing 2 | 57414 |
| 17994 | -2.0721 | 0.191948 | 1.25E-02 | 0.039539 | 0.66385 | H200008278 | MAP7D1 | MAP7 domain containing 1 | 55700 |
| 12821 | -1.7886 | 0.198843 | 2.80E-02 | 0.062622 | 0.663856 | H200001190 | CAMKV | CaM kinase-like vesicle-associated | 79012 |
| 15483 | -2.5091 | 0.157372 | 3.62E-03 | 0.020512 | 0.663954 | H200019215 | NA | NA | - |
| 10714 | -2.0415 | 0.183046 | 1.36E-02 | 0.041615 | 0.664033 | H200009806 | NA | NA | - |
| 8278 | -2.8236 | 0.14104 | 1.57E-03 | 0.013697 | 0.664078 | H200002444 | THTPA | thiamine triphosphatase | 79178 |
| 14276 | -2.4329 | 0.161612 | 4.47E-03 | 0.022604 | 0.664204 | H200005269 | MAPK11 | mitogen-activated protein kinase 11 | 5600 |
| 4952 | -1.9511 | 0.217651 | 1.76E-02 | 0.048156 | 0.664409 | H200018049 | YY2 | YY2 transcription factor | 404281 |
| 16432 | -2.4279 | 0.124526 | 4.53E-03 | 0.022795 | 0.664479 | H200020801 | NA | NA | - |
| 8212 | -1.7919 | 0.255528 | 2.77E-02 | 0.062285 | 0.664928 | H200021123 | TNS1 | tensin 1 | 7145 |
| 2166 | -1.9846 | 0.159275 | 1.60E-02 | 0.045471 | 0.664931 | H200016019 | NA | NA | - |
| 1439 | -2.4412 | 0.169432 | 4.37E-03 | 0.022368 | 0.665004 | H200003319 | NA | NA | - |
| 15795 | -1.9543 | 0.182283 | 1.75E-02 | 0.047876 | 0.665045 | H200012292 | PCBP3 | poly(rC) binding protein 3 | 54039 |
| 1830 | -2.1572 | 0.196462 | 9.79E-03 | 0.034781 | 0.665124 | H200000059 | LTK | leukocyte receptor tyrosine kinase | 4058 |
| 17984 | -1.8769 | 0.25557 | 2.18E-02 | 0.053976 | 0.665153 | H200007590 | EXD3 | exonuclease 3'-5' domain containing 3 | 54932 |
| 12386 | -2.4 | 0.158348 | 4.89E-03 | 0.023723 | 0.665276 | H200002490 | INTS9 | integrator complex subunit 9 | 55756 |
| 14423 | -2.1326 | 0.180423 | 1.05E-02 | 0.036218 | 0.665441 | H200012151 | NRDE2 | NRDE-2, necessary for RNA interference, domain containing | 55051 |
| 13150 | -2.2154 | 0.142384 | 8.29E-03 | 0.031626 | 0.665644 | H200016776 | ALKBH4 | alkB, alkylation repair homolog 4 (E. coli) | 54784 |
| 14004 | -1.9121 | 0.162849 | 1.97E-02 | 0.051136 | 0.665667 | H200013772 | HIST1H2BO | histone cluster 1, H2bo | 8348 |
| 20192 | -2.2305 | 0.116449 | 7.95E-03 | 0.031034 | 0.665683 | H200010670 | FLYWCH2 | FLYWCH family member 2 | 114984 |
| 15755 | -2.4999 | 0.144111 | 3.71E-03 | 0.020795 | 0.665828 | H200010392 | UBTF | upstream binding transcription factor, RNA polymerase I | 7343 |
| 14058 | -2.0699 | 0.118579 | 1.25E-02 | 0.039613 | 0.666218 | H200016408 | NA | NA | - |
| 16645 | -1.9226 | 0.223847 | 1.91E-02 | 0.050194 | 0.666326 | H200009169 | NA | NA | - |
| 19472 | -1.8873 | 0.221688 | 2.11E-02 | 0.053085 | 0.66649 | H200018419 | PHLDB1 | pleckstrin homology-like domain, family B, member 1 | 23187 |
| 860 | -1.9105 | 0.185672 | 1.98E-02 | 0.051224 | 0.666538 | H200019225 | TRIM51 | tripartite motif-containing 51 | 84767 |
| 20148 | -1.9128 | 0.355495 | 1.96E-02 | 0.051098 | 0.666566 | H200008366 | DBNDD2 | dysbindin (dystrobrevin binding protein 1) domain containing 2 | 55861 |
| 8038 | -1.961 | 0.161689 | 1.71E-02 | 0.047374 | 0.666663 | H200012787 | ATP13A2 | ATPase type 13A2 | 23400 |
| 11844 | -1.8691 | 0.217066 | 2.23E-02 | 0.05464 | 0.66675 | H200019982 | ZNF598 | zinc finger protein 598 | 90850 |
| 5537 | -2.1231 | 0.240265 | 1.08E-02 | 0.036874 | 0.66676 | H200002664 | RAB11FIP1 | RAB11 family interacting protein 1 (class I) | 80223 |
| 7449 | -2.2076 | 0.193499 | 8.48E-03 | 0.032079 | 0.666784 | H200006558 | TIE1 | tyrosine kinase with immunoglobulin-like and EGF-like domains 1 | 7075 |
| 5736 | -2.2249 | 0.182553 | 8.07E-03 | 0.031188 | 0.66681 | H200011862 | OXT | oxytocin/neurophysin I prepropeptide | 5020 |
| 20056 | -2.4521 | 0.150919 | 4.24E-03 | 0.022198 | 0.666821 | H200003118 | N4BP2L1 | NEDD4 binding protein 2-like 1 | 90634 |
| 18707 | -2.5101 | 0.133976 | 3.61E-03 | 0.020512 | 0.666895 | H200001384 | PNMAL2 | paraneoplastic Ma antigen family-like 2 | 57469 |
| 6070 | -2.908 | 0.115334 | 1.26E-03 | 0.012277 | 0.666971 | H200006375 | PRKACA | protein kinase, cAMP-dependent, catalytic, alpha | 5566 |
| 17439 | -2.562 | 0.117479 | 3.14E-03 | 0.019068 | 0.667148 | H200003439 | HGS | hepatocyte growth factor-regulated tyrosine kinase substrate | 9146 |
| 5652 | -2.3831 | 0.158217 | 5.13E-03 | 0.024326 | 0.667291 | H200008014 | HESX1 | HESX homeobox 1 | 8820 |
| 3490 | -1.9216 | 0.128712 | 1.92E-02 | 0.050277 | 0.667375 | H200013988 | IRF2BP1 | interferon regulatory factor 2 binding protein 1 | 26145 |
| 12085 | -2.0517 | 0.174498 | 1.32E-02 | 0.040821 | 0.66789 | H200009657 | MOSPD3 | motile sperm domain containing 3 | 64598 |
| 17401 | -3.0078 | 0.115347 | 9.79E-04 | 0.011076 | 0.667903 | H200001563 | USP21 | ubiquitin specific peptidase 21 | 27005 |
| 5174 | -2.2016 | 0.133324 | 8.62E-03 | 0.032386 | 0.667941 | H200007100 | SFSWAP | splicing factor, suppressor of white-apricot homolog (Drosophila) | 6433 |
[truncated: 56,199 more chars]
