# Supplementary material for: A regulatory mutant on TRIM26 conferring the risk of nasopharyngeal carcinoma by inducing low immune response
Source: Cancer Med. 2018 Jun 28;7(8):3848–61. doi: 10.1002/cam4.1537 (PMC6089173; doi:10.1002/cam4.1537)
Supplement: Supplementary file 4 [file CAM4-7-3848-s004.docx]

Supplemental Table 10. Differentially expressed genes between high-Trim26-NPC and NP samples.

| Row | d.value | stdev | rawp | q.value | R.fold | oligo_id | SYMBOL | GENENAME | LocusLink |
| --- | --- | --- | --- | --- | --- | --- | --- | --- | --- |
| 7130 | -3.42945 | 16.255345 | 3.73E-03 | 0.1058952 | 0.100262 | H200013227 | PLD4 | phospholipase D family, member 4 | 122618 |
| 11896 | -3.69691 | 8.7280913 | 2.34E-03 | 0.0979394 | 0.115659 | H200000567 | BLK | B lymphoid tyrosine kinase | 640 |
| 570 | -4.15252 | 13.518285 | 1.08E-03 | 0.0839146 | 0.130163 | H200005521 | PTPN6 | protein tyrosine phosphatase, non-receptor type 6 | 5777 |
| 19935 | -6.38728 | 4.1303839 | 4.51E-05 | 0.0482589 | 0.169431 | H200018876 | IL24 | interleukin 24 | 11009 |
| 9251 | -5.2475 | 2.2616194 | 2.01E-04 | 0.0633652 | 0.170661 | H200005347 | FAIM3 | Fas apoptotic inhibitory molecule 3 | 9214 |
| 9306 | -4.31729 | 3.9129923 | 8.29E-04 | 0.0784042 | 0.188834 | H200007989 | CD22 | CD22 molecule | 933 |
| 12123 | -4.99768 | 5.8288461 | 2.96E-04 | 0.0633652 | 0.195227 | H200011533 | KLF2 | Kruppel-like factor 2 (lung) | 10365 |
| 18443 | -3.52611 | 4.7613208 | 3.14E-03 | 0.1035944 | 0.198368 | H200008841 | CPNE5 | copine V | 57699 |
| 11163 | -3.60275 | 10.227631 | 2.75E-03 | 0.1014468 | 0.219038 | H200009241 | GPSM3 | G-protein signaling modulator 3 | 63940 |
| 20921 | -4.60364 | 3.7925026 | 5.28E-04 | 0.070732 | 0.222372 | H200005742 | DCP1A | DCP1 decapping enzyme homolog A (S. cerevisiae) | 55802 |
| 2088 | -3.64996 | 4.0898115 | 2.53E-03 | 0.0982771 | 0.222623 | H200012243 | PARVG | parvin, gamma | 64098 |
| 10353 | -4.55161 | 8.3090207 | 5.74E-04 | 0.0735416 | 0.236436 | H200014123 | PTPRCAP | protein tyrosine phosphatase, receptor type, C-associated protein | 5790 |
| 19205 | -4.19003 | 3.6602063 | 1.02E-03 | 0.0839146 | 0.243839 | H200005837 | NA | NA | - |
| 16803 | -4.60582 | 1.9926833 | 5.26E-04 | 0.070732 | 0.24821 | H200016745 | UPK1B | uroplakin 1B | 7348 |
| 18189 | -3.72162 | 1.0600208 | 2.24E-03 | 0.0979394 | 0.248457 | H200017428 | NA | NA | - |
| 14397 | -3.72541 | 1.3627877 | 2.22E-03 | 0.0979394 | 0.249899 | H200010987 | 1-Sep | septin 1 | 1731 |
| 556 | -3.72066 | 4.947434 | 2.24E-03 | 0.0979394 | 0.257687 | H200004785 | NA | NA | - |
| 9735 | -3.72788 | 4.932483 | 2.22E-03 | 0.0979394 | 0.264351 | H200006772 | DPT | dermatopontin | 1805 |
| 4360 | -3.55771 | 1.4782218 | 2.97E-03 | 0.1024962 | 0.266273 | H200011672 | RHOH | ras homolog family member H | 399 |
| 16060 | -4.00514 | 2.9077139 | 1.38E-03 | 0.0909941 | 0.269137 | H200003273 | TFEB | transcription factor EB | 7942 |
| 15085 | -3.86118 | 6.9708557 | 1.77E-03 | 0.0935068 | 0.27956 | H200000239 | CD48 | CD48 molecule | 962 |
| 13271 | -4.96683 | 1.4188189 | 3.10E-04 | 0.0633652 | 0.293979 | H200001071 | ATP2A3 | ATPase, Ca++ transporting, ubiquitous | 489 |
| 11319 | -4.13336 | 4.1341343 | 1.12E-03 | 0.0853193 | 0.294466 | H200016509 | TRAF3IP3 | TRAF3 interacting protein 3 | 80342 |
| 11521 | -4.1204 | 3.6470189 | 1.14E-03 | 0.085653 | 0.296484 | H200004752 | PPP1R16B | protein phosphatase 1, regulatory subunit 16B | 26051 |
| 16859 | -3.46968 | 1.1051996 | 3.47E-03 | 0.1045639 | 0.297527 | H200019405 | FXYD5 | FXYD domain containing ion transport regulator 5 | 53827 |
| 11138 | -3.59952 | 1.5985681 | 2.76E-03 | 0.1014468 | 0.300073 | H200008083 | IL16 | interleukin 16 | 3603 |
| 21361 | -3.44078 | 3.7298037 | 3.66E-03 | 0.1052221 | 0.30612 | H200004935 | GMIP | GEM interacting protein | 51291 |
| 5448 | -5.11823 | 0.7681661 | 2.46E-04 | 0.0633652 | 0.31002 | H200020044 | TNFRSF13C | tumor necrosis factor receptor superfamily, member 13C | 115650 |
| 15792 | -3.85725 | 1.5019263 | 1.78E-03 | 0.0936829 | 0.3182 | H200011966 | DPEP2 | dipeptidase 2 | 64174 |
| 4601 | -3.84634 | 10.996068 | 1.81E-03 | 0.0944911 | 0.323972 | H200001631 | PTGDS | prostaglandin D2 synthase 21kDa (brain) | 5730 |
| 6593 | -4.78672 | 0.4794924 | 4.02E-04 | 0.065389 | 0.330806 | H200009184 | NA | NA | - |
| 9445 | -3.76176 | 2.5512168 | 2.09E-03 | 0.0968513 | 0.33358 | H200014491 | FCRL5 | Fc receptor-like 5 | 83416 |
| 15416 | -4.03241 | 0.4726242 | 1.32E-03 | 0.0889091 | 0.33685 | H200015849 | LY6G5C | lymphocyte antigen 6 complex, locus G5C | 80741 |
| 18729 | -4.54413 | 1.1338842 | 5.81E-04 | 0.0737951 | 0.348194 | H200002542 | STK10 | serine/threonine kinase 10 | 6793 |
| 12153 | -6.72942 | 0.1744281 | 2.99E-05 | 0.0482589 | 0.348882 | H200013029 | FBXO2 | F-box protein 2 | 26232 |
| 884 | -4.7723 | 0.4387865 | 4.12E-04 | 0.065389 | 0.349035 | H200020365 | NA | NA | - |
| 14105 | -4.28137 | 0.4484149 | 8.80E-04 | 0.079111 | 0.34997 | H200018682 | NA | NA | - |
| 3928 | -3.76195 | 1.6770321 | 2.09E-03 | 0.0968513 | 0.350524 | H200012717 | GNG7 | guanine nucleotide binding protein (G protein), gamma 7 | 2788 |
| 819 | -4.34472 | 0.9066114 | 7.91E-04 | 0.077886 | 0.351745 | H200017319 | PTPN18 | protein tyrosine phosphatase, non-receptor type 18 (brain-derived) | 26469 |
| 7162 | -4.12255 | 1.2828241 | 1.14E-03 | 0.085653 | 0.351909 | H200014747 | HMHA1 | histocompatibility (minor) HA-1 | 23526 |
| 17014 | -4.16451 | 2.375466 | 1.06E-03 | 0.0839146 | 0.353946 | H200005226 | LAT2 | linker for activation of T cells family, member 2 | 7462 |
| 20626 | -3.31305 | 0.7173936 | 4.58E-03 | 0.1111579 | 0.355908 | H200012499 | DAGLA | diacylglycerol lipase, alpha | 747 |
| 3017 | -4.17034 | 0.2454376 | 1.05E-03 | 0.0839146 | 0.359402 | H200012985 | CLCF1 | cardiotrophin-like cytokine factor 1 | 23529 |
| 21638 | -5.22337 | 0.5884231 | 2.08E-04 | 0.0633652 | 0.361708 | H200018205 | C22orf32 | chromosome 22 open reading frame 32 | 91689 |
| 1215 | -3.40858 | 1.3112426 | 3.87E-03 | 0.1058952 | 0.366129 | H200014244 | LY86 | lymphocyte antigen 86 | 9450 |
| 160 | -5.7164 | 0.5525927 | 1.05E-04 | 0.0630916 | 0.3671 | H200007398 | TMSB4Y | thymosin beta 4, Y-linked | 9087 |
| 8151 | -3.7885 | 0.3475236 | 1.99E-03 | 0.0968513 | 0.368468 | H200018125 | CDKL1 | cyclin-dependent kinase-like 1 (CDC2-related kinase) | 8814 |
| 13858 | -4.81592 | 1.6925536 | 3.87E-04 | 0.065389 | 0.370258 | H200006908 | CD53 | CD53 molecule | 963 |
| 10871 | -3.70789 | 0.3472189 | 2.29E-03 | 0.0979394 | 0.371475 | H200017092 | RALGPS2 | Ral GEF with PH domain and SH3 binding motif 2 | 55103 |
| 615 | -3.84644 | 0.5755335 | 1.81E-03 | 0.0944911 | 0.371579 | H200007487 | KLK12 | kallikrein-related peptidase 12 | 43849 |
| 8936 | -3.51541 | 1.9509237 | 3.20E-03 | 0.1035944 | 0.371637 | H200012063 | KRT14 | keratin 14 | 3861 |
| 2340 | -3.89594 | 0.9190437 | 1.66E-03 | 0.0920659 | 0.372276 | H200002648 | ULK4 | unc-51-like kinase 4 (C. elegans) | 54986 |
| 1379 | -4.3988 | 1.3430438 | 7.27E-04 | 0.0766926 | 0.373338 | H200000611 | POU2AF1 | POU class 2 associating factor 1 | 5450 |
| 9602 | -5.68539 | 0.7726133 | 1.09E-04 | 0.0630916 | 0.377859 | H200000342 | CD1C | CD1c molecule | 911 |
| 11390 | -3.81365 | 0.7309837 | 1.91E-03 | 0.0963887 | 0.380183 | H200019911 | CMTM7 | CKLF-like MARVEL transmembrane domain containing 7 | 112616 |
| 19958 | -6.37086 | 0.4211588 | 4.59E-05 | 0.0482589 | 0.38289 | H200020010 | FLJ22447 | uncharacterized LOC400221 | 400221 |
| 3191 | -4.06628 | 0.5378722 | 1.25E-03 | 0.0874811 | 0.386364 | H200021321 | PLCB2 | phospholipase C, beta 2 | 5330 |
| 9320 | -3.65847 | 2.0679718 | 2.49E-03 | 0.0979394 | 0.386757 | H200008441 | FERMT3 | fermitin family member 3 | 83706 |
| 8994 | -3.70382 | 0.3195414 | 2.31E-03 | 0.0979394 | 0.391496 | H200015031 | VWA5B1 | von Willebrand factor A domain containing 5B1 | 127731 |
| 13059 | -3.48347 | 0.3850109 | 3.38E-03 | 0.1045639 | 0.39152 | H200012566 | NA | NA | 286354 |
| 8914 | -3.67818 | 1.0097257 | 2.41E-03 | 0.0979394 | 0.400001 | H200011231 | RAB3IP | RAB3A interacting protein | 117177 |
| 12901 | -3.35623 | 0.9645581 | 4.24E-03 | 0.1090499 | 0.401997 | H200004990 | LIMD2 | LIM domain containing 2 | 80774 |
| 20575 | -3.40872 | 0.5204559 | 3.87E-03 | 0.1058952 | 0.405785 | H200009815 | NA | NA | - |
| 3837 | -3.66325 | 0.3650977 | 2.47E-03 | 0.0979394 | 0.40843 | H200008507 | NA | NA | - |
| 2742 | -3.33871 | 0.6851129 | 4.38E-03 | 0.1095379 | 0.410816 | H200000023 | ABHD14B | abhydrolase domain containing 14B | 84836 |
| 12883 | -4.21577 | 0.4833068 | 9.79E-04 | 0.0831898 | 0.411621 | H200004206 | NA | NA | 196266 |
| 19188 | -6.43368 | 0.2408214 | 4.30E-05 | 0.0482589 | 0.419392 | H200005071 | GP2 | glycoprotein 2 (zymogen granule membrane) | 2813 |
| 3395 | -5.18568 | 0.0847011 | 2.21E-04 | 0.0633652 | 0.420514 | H200009446 | GDPD3 | glycerophosphodiester phosphodiesterase domain containing 3 | 79153 |
| 19298 | -4.49091 | 0.390882 | 6.27E-04 | 0.076403 | 0.420528 | H200010367 | RYR1 | ryanodine receptor 1 (skeletal) | 6261 |
| 1190 | -3.90664 | 0.2401731 | 1.63E-03 | 0.0914734 | 0.424351 | H200013086 | WDR31 | WD repeat domain 31 | 114987 |
| 14976 | -3.62376 | 0.2702298 | 2.65E-03 | 0.1005255 | 0.429254 | H200016514 | RPL32P3 | ribosomal protein L32 pseudogene 3 | 132241 |
| 5277 | -4.06152 | 0.4505127 | 1.26E-03 | 0.0874811 | 0.431343 | H200012034 | NA | NA | - |
| 15332 | -4.02947 | 0.5639746 | 1.33E-03 | 0.0889233 | 0.432452 | H200012001 | VAV1 | vav 1 guanine nucleotide exchange factor | 7409 |
| 7240 | -3.56094 | 1.0450683 | 2.96E-03 | 0.1024962 | 0.436766 | H200018239 | NA | NA | 79164 |
| 2314 | -6.21089 | 0.2816733 | 5.60E-05 | 0.0518194 | 0.441547 | H200001484 | SMAP2 | small ArfGAP2 | 64744 |
| 2053 | -3.40871 | 0.5162192 | 3.87E-03 | 0.1058952 | 0.444512 | H200010693 | NFRKB | nuclear factor related to kappaB binding protein | 4798 |
| 11492 | -3.69472 | 0.6404386 | 2.35E-03 | 0.0979394 | 0.445101 | H200003262 | RNASE6 | ribonuclease, RNase A family, k6 | 6039 |
| 3734 | -3.32923 | 0.5634881 | 4.45E-03 | 0.1100195 | 0.445948 | H200003573 | NA | NA | - |
| 10691 | -4.97847 | 0.2765065 | 3.04E-04 | 0.0633652 | 0.453638 | H200008684 | ZNF835 | zinc finger protein 835 | 90485 |
| 14824 | -3.4103 | 0.4839962 | 3.86E-03 | 0.1058952 | 0.454609 | H200009294 | NA | NA | - |
| 14601 | -5.79188 | 0.9404349 | 9.53E-05 | 0.0630916 | 0.45795 | H200020819 | NA | NA | - |
| 2862 | -3.59316 | 0.3568816 | 2.79E-03 | 0.101597 | 0.458174 | H200005723 | LSM7 | LSM7 homolog, U6 small nuclear RNA associated (S. cerevisiae) | 51690 |
| 13304 | -4.95701 | 0.2139258 | 3.15E-04 | 0.0633652 | 0.45819 | H200002597 | ZNF185 | zinc finger protein 185 (LIM domain) | 7739 |
| 19258 | -3.91308 | 1.1125115 | 1.61E-03 | 0.0914041 | 0.461342 | H200008467 | NA | NA | - |
| 18763 | -3.33144 | 0.3472798 | 4.43E-03 | 0.1098034 | 0.463388 | H200004424 | SULT1C2 | sulfotransferase family, cytosolic, 1C, member 2 | 6819 |
| 19025 | -3.33896 | 0.4769934 | 4.37E-03 | 0.1095379 | 0.465852 | H200018816 | NA | NA | - |
| 3730 | -4.04334 | 0.2780162 | 1.29E-03 | 0.0883163 | 0.467971 | H200003525 | SLC29A1 | solute carrier family 29 (nucleoside transporters), member 1 | 2030 |
| 19903 | -3.76412 | 0.4841148 | 2.08E-03 | 0.0968513 | 0.468517 | H200017356 | PIK3R5 | phosphoinositide-3-kinase, regulatory subunit 5 | 23533 |
| 14386 | -3.36019 | 0.8852943 | 4.22E-03 | 0.1090499 | 0.468657 | H200010565 | VASP | vasodilator-stimulated phosphoprotein | 7408 |
| 17001 | -4.10836 | 0.2994625 | 1.16E-03 | 0.086144 | 0.472491 | H200004496 | UBIAD1 | UbiA prenyltransferase domain containing 1 | 29914 |
| 1034 | -3.45567 | 0.3033829 | 3.56E-03 | 0.1052221 | 0.474011 | H200005818 | NA | NA | - |
| 11582 | -5.1506 | 0.3684026 | 2.34E-04 | 0.0633652 | 0.476239 | H200007466 | ABCD1 | ATP-binding cassette, sub-family D (ALD), member 1 | 215 |
| 17698 | -5.57229 | 0.1717589 | 1.28E-04 | 0.0630916 | 0.476539 | H200015641 | NA | NA | - |
| 11161 | -7.63765 | 0.1721727 | 1.11E-05 | 0.0477624 | 0.476681 | H200009217 | NA | NA | - |
| 7627 | -3.87765 | 0.3186475 | 1.72E-03 | 0.0929964 | 0.478348 | H200014942 | LILRP2 | leukocyte immunoglobulin-like receptor pseudogene 2 | 79166 |
| 400 | -3.96597 | 0.1848894 | 1.47E-03 | 0.0914041 | 0.480318 | H200018798 | NA | NA | - |
| 5955 | -4.5754 | 0.3627881 | 5.53E-04 | 0.0721858 | 0.480429 | H200000741 | ARHGAP4 | Rho GTPase activating protein 4 | 393 |
| 91 | -3.39099 | 0.3287549 | 3.99E-03 | 0.1069886 | 0.48187 | H200004304 | FAM167A | family with sequence similarity 167, member A | 83648 |
| 9939 | -4.83142 | 0.4929852 | 3.78E-04 | 0.065389 | 0.482802 | H200016320 | ARHGEF1 | Rho guanine nucleotide exchange factor (GEF) 1 | 9138 |
| 21324 | -4.46946 | 0.1894452 | 6.52E-04 | 0.0765008 | 0.485536 | H200003361 | STAT5B | signal transducer and activator of transcription 5B | 6777 |
| 11026 | -3.38632 | 0.3590164 | 4.03E-03 | 0.1070738 | 0.485701 | H200002763 | TESC | tescalcin | 54997 |
| 4622 | -3.51304 | 0.3525787 | 3.21E-03 | 0.1035944 | 0.486888 | H200002445 | RHOBTB1 | Rho-related BTB domain containing 1 | 9886 |
| 17913 | -5.51218 | 0.1427044 | 1.39E-04 | 0.0630916 | 0.488923 | H200004176 | FASTKD1 | FAST kinase domains 1 | 79675 |
| 11584 | -3.70181 | 0.3276353 | 2.32E-03 | 0.0979394 | 0.488931 | H200007490 | NA | NA | - |
| 19955 | -3.93788 | 0.3479529 | 1.54E-03 | 0.0914041 | 0.490035 | H200019968 | CMTM1 | CKLF-like MARVEL transmembrane domain containing 1 | 113540 |
| 15824 | -3.71506 | 0.2586959 | 2.26E-03 | 0.0979394 | 0.494743 | H200013486 | GNMT | glycine N-methyltransferase | 27232 |
| 15169 | -4.97228 | 0.1538005 | 3.07E-04 | 0.0633652 | 0.497656 | H200004371 | CALCA | calcitonin-related polypeptide alpha | 796 |
| 9101 | -4.06129 | 0.2221538 | 1.26E-03 | 0.0874811 | 0.498516 | H200020013 | NA | NA | - |
| 15995 | -4.64868 | 0.414452 | 4.93E-04 | 0.0693846 | 0.499085 | H200000227 | IMPDH1 | IMP (inosine 5'-monophosphate) dehydrogenase 1 | 3614 |
| 20890 | -4.31143 | 0.2814519 | 8.37E-04 | 0.0784907 | 0.499474 | H200004240 | NA | NA | 153277 |
| 19052 | -3.67809 | 0.452518 | 2.41E-03 | 0.0979394 | 0.501033 | H200020028 | FAM83C | family with sequence similarity 83, member C | 128876 |
| 5084 | -3.95544 | 0.4607329 | 1.50E-03 | 0.0914041 | 0.50248 | H200002896 | C20orf196 | chromosome 20 open reading frame 196 | 149840 |
| 19111 | -3.75375 | 0.5057569 | 2.12E-03 | 0.0968513 | 0.502503 | H200001301 | TWF2 | twinfilin, actin-binding protein, homolog 2 (Drosophila) | 11344 |
| 13967 | -4.50894 | 0.2848155 | 6.11E-04 | 0.0760375 | 0.503191 | H200012198 | RASA3 | RAS p21 protein activator 3 | 22821 |
| 14800 | -3.55567 | 0.3933338 | 2.98E-03 | 0.1024962 | 0.505584 | H200008154 | SERPINF1 | serpin peptidase inhibitor, clade F (alpha-2 antiplasmin, pigment epithelium derived factor), member 1 | 5176 |
| 9197 | -3.58353 | 0.3740909 | 2.84E-03 | 0.101854 | 0.505683 | H200002711 | DENND3 | DENN/MADD domain containing 3 | 22898 |
| 1597 | -3.36899 | 0.9564031 | 4.15E-03 | 0.1090499 | 0.510674 | H200010895 | CCDC88B | coiled-coil domain containing 88B | 283234 |
| 17692 | -3.66031 | 0.3675734 | 2.48E-03 | 0.0979394 | 0.510761 | H200015285 | EIF1B-AS1 | EIF1B antisense RNA 1 | 440952 |
| 20834 | -5.60173 | 0.2773024 | 1.22E-04 | 0.0630916 | 0.512764 | H200001580 | PBXIP1 | pre-B-cell leukemia homeobox interacting protein 1 | 57326 |
| 12945 | -3.99551 | 0.295022 | 1.40E-03 | 0.0909941 | 0.515932 | H200007222 | CALCOCO1 | calcium binding and coiled-coil domain 1 | 57658 |
| 10549 | -5.44239 | 0.1579943 | 1.54E-04 | 0.0630916 | 0.516795 | H200001868 | EMP3 | epithelial membrane protein 3 | 2014 |
| 18155 | -3.49905 | 0.1109391 | 3.29E-03 | 0.1045639 | 0.518167 | H200015884 | MAGEC3 | melanoma antigen family C, 3 | 139081 |
| 4057 | -3.68237 | 0.4698271 | 2.40E-03 | 0.0979394 | 0.523592 | H200019099 | EGLN2 | egl nine homolog 2 (C. elegans) | 112398 |
| 4435 | -3.743 | 0.1942661 | 2.16E-03 | 0.0968513 | 0.524149 | H200015418 | PLEKHS1 | pleckstrin homology domain containing, family S member 1 | 79949 |
| 1971 | -3.75593 | 0.2338387 | 2.11E-03 | 0.0968513 | 0.524917 | H200006869 | GYPC | glycophorin C (Gerbich blood group) | 2995 |
| 16066 | -4.04667 | 0.1221271 | 1.29E-03 | 0.0883163 | 0.52521 | H200003629 | CNRIP1 | cannabinoid receptor interacting protein 1 | 25927 |
| 607 | -3.82799 | 0.3346773 | 1.87E-03 | 0.0959212 | 0.52661 | H200007107 | CTDNEP1 | CTD nuclear envelope phosphatase 1 | 23399 |
| 8778 | -3.55774 | 0.3174479 | 2.97E-03 | 0.1024962 | 0.528663 | H200004771 | LYL1 | lymphoblastic leukemia derived sequence 1 | 4066 |
| 649 | -3.60419 | 0.102149 | 2.74E-03 | 0.1014468 | 0.528963 | H200009315 | NA | NA | - |
| 3360 | -3.37633 | 0.2967662 | 4.09E-03 | 0.1082123 | 0.529909 | H200007600 | FGF7 | fibroblast growth factor 7 | 2252 |
| 6360 | -3.40845 | 0.2939923 | 3.87E-03 | 0.1058952 | 0.531271 | H200020079 | NA | NA | - |
| 9247 | -3.44996 | 0.4114549 | 3.60E-03 | 0.1052221 | 0.531814 | H200005015 | HOXC4 | homeobox C4 | 3221 |
| 11599 | -4.43046 | 0.1600932 | 6.92E-04 | 0.0765008 | 0.533126 | H200008244 | DCAF7 | DDB1 and CUL4 associated factor 7 | 10238 |
| 15056 | -3.69301 | 0.2873066 | 2.36E-03 | 0.0979394 | 0.533317 | H200020314 | NA | NA | - |
| 17148 | -3.78567 | 0.5055063 | 2.00E-03 | 0.0968513 | 0.533538 | H200011378 | SLC44A2 | solute carrier family 44, member 2 | 57153 |
| 1448 | -3.35559 | 0.3181311 | 4.25E-03 | 0.1090499 | 0.53399 | H200003705 | RASSF1 | Ras association (RalGDS/AF-6) domain family member 1 | 11186 |
| 661 | -4.98047 | 0.1348652 | 3.03E-04 | 0.0633652 | 0.535002 | H200009743 | NA | NA | - |
| 9726 | -3.75668 | 0.4414534 | 2.11E-03 | 0.0968513 | 0.537832 | H200006374 | GNAI2 | guanine nucleotide binding protein (G protein), alpha inhibiting activity polypeptide 2 | 2771 |
| 16933 | -3.60366 | 0.4065304 | 2.74E-03 | 0.1014468 | 0.539598 | H200001124 | SUN2 | Sad1 and UNC84 domain containing 2 | 25777 |
| 12674 | -3.4026 | 0.1712418 | 3.91E-03 | 0.1061874 | 0.539892 | H200016170 | ZNF550 | zinc finger protein 550 | 162972 |
| 11802 | -4.09039 | 0.2258216 | 1.20E-03 | 0.0865608 | 0.544965 | H200018058 | NA | NA | - |
| 12207 | -3.31634 | 0.2070108 | 4.56E-03 | 0.110715 | 0.546409 | H200015381 | NA | NA | - |
| 11086 | -4.45189 | 0.2676578 | 6.69E-04 | 0.0765008 | 0.546619 | H200005471 | RHOBTB2 | Rho-related BTB domain containing 2 | 23221 |
| 19546 | -3.35693 | 0.2741184 | 4.24E-03 | 0.1090499 | 0.549476 | H200000582 | CHGB | chromogranin B (secretogranin 1) | 1114 |
| 4502 | -3.74345 | 0.2365475 | 2.16E-03 | 0.0968513 | 0.550589 | H200018488 | NA | NA | - |
| 28 | -5.07519 | 0.1384532 | 2.63E-04 | 0.0633652 | 0.550605 | H200001270 | KIF5C | kinesin family member 5C | 3800 |
| 9832 | -3.57025 | 0.1301144 | 2.91E-03 | 0.1024962 | 0.550701 | H200011338 | SERPINB9 | serpin peptidase inhibitor, clade B (ovalbumin), member 9 | 5272 |
| 7512 | -3.74064 | 0.2293303 | 2.17E-03 | 0.0968513 | 0.551475 | H200009296 | CTC1 | CTS telomere maintenance complex component 1 | 80169 |
| 7482 | -3.91475 | 0.1722728 | 1.61E-03 | 0.0914041 | 0.552472 | H200008084 | NA | NA | - |
| 2019 | -3.41004 | 0.2628969 | 3.86E-03 | 0.1058952 | 0.552656 | H200009149 | LOC143188 | uncharacterized LOC143188 | 143188 |
| 21657 | -4.48684 | 0.0956141 | 6.31E-04 | 0.076403 | 0.558276 | H200018995 | RPUSD4 | RNA pseudouridylate synthase domain containing 4 | 84881 |
| 11037 | -3.79673 | 0.5615518 | 1.97E-03 | 0.0968513 | 0.559854 | H200003185 | RGS19 | regulator of G-protein signaling 19 | 10287 |
| 10388 | -3.42222 | 0.2499596 | 3.78E-03 | 0.1058952 | 0.563679 | H200015673 | FOXO4 | forkhead box O4 | 4303 |
| 5062 | -3.55165 | 0.1932782 | 3.01E-03 | 0.1026076 | 0.563762 | H200001780 | VPS39 | vacuolar protein sorting 39 homolog (S. cerevisiae) | 23339 |
| 6733 | -3.49948 | 0.6248447 | 3.29E-03 | 0.1045639 | 0.563912 | H200015976 | NA | NA | - |
| 9624 | -4.97276 | 0.0670831 | 3.07E-04 | 0.0633652 | 0.564894 | H200001458 | NA | NA | - |
| 17883 | -3.66216 | 0.1500015 | 2.47E-03 | 0.0979394 | 0.567242 | H200002964 | C3orf18 | chromosome 3 open reading frame 18 | 51161 |
| 2753 | -3.4685 | 0.223844 | 3.48E-03 | 0.1045639 | 0.567515 | H200000445 | NA | NA | - |
| 12590 | -3.59737 | 0.2847295 | 2.77E-03 | 0.1014468 | 0.568222 | H200012038 | RGS9 | regulator of G-protein signaling 9 | 8787 |
| 17661 | -5.22997 | 0.1166174 | 2.07E-04 | 0.0633652 | 0.569192 | H200013771 | NA | NA | - |
| 18593 | -3.45884 | 0.2978992 | 3.54E-03 | 0.1051472 | 0.570891 | H200016863 | NA | NA | - |
| 13690 | -4.2927 | 0.1381828 | 8.62E-04 | 0.0784907 | 0.571568 | H200020861 | NA | NA | - |
| 17436 | -3.8801 | 0.4299332 | 1.71E-03 | 0.0929702 | 0.572645 | H200003125 | SMOC2 | SPARC related modular calcium binding 2 | 64094 |
| 13283 | -4.75236 | 0.2208041 | 4.24E-04 | 0.065389 | 0.574446 | H200001499 | TINF2 | TERF1 (TRF1)-interacting nuclear factor 2 | 26277 |
| 9973 | -4.22139 | 0.5158613 | 9.70E-04 | 0.0831898 | 0.57977 | H200018148 | GLIPR2 | GLI pathogenesis-related 2 | 152007 |
| 14489 | -5.81898 | 0.1505521 | 9.11E-05 | 0.0630916 | 0.585443 | H200015499 | VPS11 | vacuolar protein sorting 11 homolog (S. cerevisiae) | 55823 |
| 8993 | -5.54317 | 0.0763656 | 1.33E-04 | 0.0630916 | 0.589761 | H200015025 | DCLK3 | doublecortin-like kinase 3 | 85443 |
| 14067 | -4.69462 | 0.1414295 | 4.61E-04 | 0.0686835 | 0.59055 | H200016806 | TRPM6 | transient receptor potential cation channel, subfamily M, member 6 | 140803 |
| 10079 | -3.9813 | 0.2225415 | 1.43E-03 | 0.0909941 | 0.591771 | H200001179 | ILK | integrin-linked kinase | 3611 |
| 3952 | -4.32339 | 0.2703721 | 8.21E-04 | 0.0781359 | 0.593189 | H200013857 | RPS28 | ribosomal protein S28 | 6234 |
| 7697 | -3.55086 | 0.1765557 | 3.01E-03 | 0.1026076 | 0.595356 | H200018338 | NA | NA | - |
| 5332 | -3.47759 | 0.3346193 | 3.42E-03 | 0.1045639 | 0.59748 | H200014676 | NA | NA | - |
| 2660 | -4.20569 | 0.1468239 | 9.96E-04 | 0.0837309 | 0.598202 | H200017848 | NA | NA | - |
| 12709 | -3.81722 | 0.271727 | 1.90E-03 | 0.0960996 | 0.601231 | H200017732 | KDM5C | lysine (K)-specific demethylase 5C | 8242 |
| 8012 | -3.76344 | 0.290041 | 2.08E-03 | 0.0968513 | 0.601955 | H200011623 | SH3BGRL3 | SH3 domain binding glutamic acid-rich protein like 3 | 83442 |
| 11466 | -3.58698 | 0.1812046 | 2.82E-03 | 0.1018066 | 0.602076 | H200002098 | F11R | F11 receptor | 50848 |
| 17371 | -4.64829 | 0.1359187 | 4.93E-04 | 0.0693846 | 0.603136 | H200000067 | NA | NA | - |
| 7602 | -4.43372 | 0.1691577 | 6.89E-04 | 0.0765008 | 0.605171 | H200013784 | GALNT6 | UDP-N-acetyl-alpha-D-galactosamine:polypeptide N-acetylgalactosaminyltransferase 6 (GalNAc-T6) | 11226 |
| 8212 | -3.44054 | 0.2033887 | 3.66E-03 | 0.1052221 | 0.605557 | H200021123 | TNS1 | tensin 1 | 7145 |
| 20038 | -3.35209 | 0.2120433 | 4.28E-03 | 0.1090499 | 0.60822 | H200002310 | RANGRF | RAN guanine nucleotide release factor | 29098 |
| 1791 | -3.93394 | 0.4825878 | 1.56E-03 | 0.0914041 | 0.609783 | H200020039 | NA | NA | - |
| 5038 | -3.95589 | 0.2619469 | 1.50E-03 | 0.0914041 | 0.612426 | H200000640 | ST6GAL1 | ST6 beta-galactosamide alpha-2,6-sialyltranferase 1 | 6480 |
| 11971 | -4.09348 | 0.3582343 | 1.19E-03 | 0.0865608 | 0.616336 | H200004313 | IGFN1 | immunoglobulin-like and fibronectin type III domain containing 1 | 91156 |
| 15156 | -3.54667 | 0.1415689 | 3.03E-03 | 0.1027813 | 0.616802 | H200003641 | TPCN1 | two pore segment channel 1 | 53373 |
| 19032 | -4.76107 | 0.1875505 | 4.19E-04 | 0.065389 | 0.62029 | H200019196 | APOL5 | apolipoprotein L, 5 | 80831 |
| 14590 | -5.27352 | 0.1268374 | 1.93E-04 | 0.0633652 | 0.621039 | H200020113 | OCEL1 | occludin/ELL domain containing 1 | 79629 |
| 19716 | -3.36968 | 0.267733 | 4.14E-03 | 0.1090499 | 0.621736 | H200008586 | TNNC2 | troponin C type 2 (fast) | 7125 |
| 19069 | -4.29045 | 0.1216029 | 8.66E-04 | 0.0784907 | 0.621886 | H200021120 | ACOT8 | acyl-CoA thioesterase 8 | 10005 |
| 3929 | -3.88131 | 0.1915066 | 1.70E-03 | 0.0929702 | 0.623588 | H200013019 | NA | NA | - |
| 6365 | -3.41149 | 0.1124587 | 3.85E-03 | 0.1058952 | 0.627238 | H200020429 | DPH5 | DPH5 homolog (S. cerevisiae) | 51611 |
| 13519 | -3.38807 | 0.2425156 | 4.02E-03 | 0.1069886 | 0.627673 | H200012851 | SPINK4 | serine peptidase inhibitor, Kazal type 4 | 27290 |
| 11997 | -5.17664 | 0.1273603 | 2.25E-04 | 0.0633652 | 0.628381 | H200005477 | SFI1 | Sfi1 homolog, spindle assembly associated (yeast) | 9814 |
| 18240 | -3.56055 | 0.2142667 | 2.96E-03 | 0.1024962 | 0.629032 | H200019750 | ORAI2 | ORAI calcium release-activated calcium modulator 2 | 80228 |
| 4182 | -3.41409 | 0.1666031 | 3.83E-03 | 0.1058952 | 0.629832 | H200003288 | NA | NA | - |
| 16138 | -3.47308 | 0.2883882 | 3.45E-03 | 0.1045639 | 0.63277 | H200007049 | ADRBK1 | adrenergic, beta, receptor kinase 1 | 156 |
| 13734 | -3.76663 | 0.1363016 | 2.07E-03 | 0.0968513 | 0.633617 | H200001160 | CPNE6 | copine VI (neuronal) | 9362 |
| 12959 | -3.44446 | 0.2091649 | 3.64E-03 | 0.1052221 | 0.635449 | H200007674 | PHF1 | PHD finger protein 1 | 5252 |
| 19541 | -3.38947 | 0.1806446 | 4.00E-03 | 0.1069886 | 0.635715 | H200000232 | MAPK3 | mitogen-activated protein kinase 3 | 5595 |
| 14378 | -3.59033 | 0.1797899 | 2.81E-03 | 0.101597 | 0.637586 | H200010185 | KANK1 | KN motif and ankyrin repeat domains 1 | 23189 |
| 18576 | -4.85555 | 0.122175 | 3.64E-04 | 0.065389 | 0.639511 | H200016061 | GDNF | glial cell derived neurotrophic factor | 2668 |
| 8586 | -4.78237 | 0.0850809 | 4.05E-04 | 0.065389 | 0.641346 | H200017216 | NR1D1 | nuclear receptor subfamily 1, group D, member 1 | 9572 |
| 7796 | -4.33557 | 0.1552013 | 8.04E-04 | 0.077886 | 0.641445 | H200001363 | GRIA1 | glutamate receptor, ionotropic, AMPA 1 | 2890 |
| 20042 | -3.63915 | 0.2040324 | 2.58E-03 | 0.0993436 | 0.642199 | H200002358 | ARHGAP17 | Rho GTPase activating protein 17 | 55114 |
| 19262 | -3.91189 | 0.0875306 | 1.62E-03 | 0.0914041 | 0.64229 | H200008515 | FLJ10038 | uncharacterized protein FLJ10038 | 55056 |
| 8434 | -3.40644 | 0.2011171 | 3.89E-03 | 0.1058952 | 0.642545 | H200009996 | PITPNM3 | PITPNM family member 3 | 83394 |
| 11905 | -3.53294 | 0.1837629 | 3.10E-03 | 0.1035944 | 0.645452 | H200001249 | SLC17A7 | solute carrier family 17 (sodium-dependent inorganic phosphate cotransporter), member 7 | 57030 |
| 21036 | -4.42768 | 0.2293769 | 6.95E-04 | 0.0765008 | 0.646036 | H200011104 | NA | NA | - |
| 7869 | -3.80231 | 0.0785668 | 1.95E-03 | 0.0968513 | 0.647845 | H200004801 | KCNMB4 | potassium large conductance calcium-activated channel, subfamily M, beta member 4 | 27345 |
| 15723 | -3.82323 | 0.116607 | 1.88E-03 | 0.0959212 | 0.653482 | H200008872 | NA | NA | - |
| 18628 | -4.40944 | 0.1279582 | 7.15E-04 | 0.0766926 | 0.65489 | H200018763 | FBXO31 | F-box protein 31 | 79791 |
| 13618 | -3.86652 | 0.1854396 | 1.75E-03 | 0.0934477 | 0.655814 | H200017441 | MYT1 | myelin transcription factor 1 | 4661 |
| 8906 | -3.51336 | 0.1630416 | 3.21E-03 | 0.1035944 | 0.655957 | H200010851 | NA | NA | - |
| 15910 | -3.98914 | 0.1606631 | 1.41E-03 | 0.0909941 | 0.656143 | H200017642 | NA | NA | - |
| 13542 | -4.22854 | 0.2633707 | 9.59E-04 | 0.0831898 | 0.659261 | H200013973 | NA | NA | - |
| 11238 | -3.39843 | 0.1985204 | 3.94E-03 | 0.1064932 | 0.659876 | H200012691 | SYT7 | synaptotagmin VII | 9066 |
| 19654 | -3.70022 | 0.2043791 | 2.33E-03 | 0.0979394 | 0.66002 | H200005570 | 9-Mar | membrane-associated ring finger (C3HC4) 9 | 92979 |
| 15483 | -3.55772 | 0.1664911 | 2.97E-03 | 0.1024962 | 0.660942 | H200019215 | NA | NA | - |
| 15797 | -3.74034 | 0.1768805 | 2.17E-03 | 0.0968513 | 0.662147 | H200012316 | SLC6A9 | solute carrier family 6 (neurotransmitter transporter, glycine), member 9 | 6536 |
| 16080 | -3.61313 | 0.1218194 | 2.70E-03 | 0.1014468 | 0.662641 | H200004081 | NA | NA | - |
| 20194 | -3.46818 | 0.1412421 | 3.48E-03 | 0.1045639 | 0.662902 | H200010694 | DDX59 | DEAD (Asp-Glu-Ala-Asp) box polypeptide 59 | 83479 |
| 9636 | -3.63073 | 0.1422447 | 2.62E-03 | 0.1003799 | 0.665376 | H200001886 | SMG9 | smg-9 homolog, nonsense mediated mRNA decay factor (C. elegans) | 56006 |
| 5237 | -3.89642 | 0.1399585 | 1.66E-03 | 0.0920659 | 0.666858 | H200010134 | NISCH | nischarin | 11188 |
| 21181 | -3.45472 | 0.2465061 | 3.57E-03 | 0.1052221 | 0.668552 | H200017950 | TEX264 | testis expressed 264 | 51368 |
| 21378 | -3.61745 | 0.4298768 | 2.68E-03 | 0.1014055 | 0.669986 | H200005997 | RHOG | ras homolog family member G | 391 |
| 2675 | -3.33838 | 0.1277154 | 4.38E-03 | 0.1095379 | 0.670491 | H200018602 | NA | NA | - |
| 20847 | -3.47533 | 0.1035749 | 3.43E-03 | 0.1045639 | 0.670907 | H200002298 | TANGO6 | transport and golgi organization 6 homolog (Drosophila) | 79613 |
| 16702 | -3.91503 | 0.1077398 | 1.61E-03 | 0.0914041 | 0.671167 | H200011835 | FAM181A | family with sequence similarity 181, member A | 90050 |
| 10087 | -4.87099 | 0.0882229 | 3.56E-04 | 0.0648892 | 0.671214 | H200001559 | SLC25A38 | solute carrier family 25, member 38 | 54977 |
| 12279 | -3.31761 | 0.1596528 | 4.55E-03 | 0.1106659 | 0.673662 | H200018801 | NA | NA | - |
| 18707 | -3.36411 | 0.153934 | 4.18E-03 | 0.1090499 | 0.673758 | H200001384 | PNMAL2 | paraneoplastic Ma antigen family-like 2 | 57469 |
| 543 | -3.75869 | 0.1150431 | 2.10E-03 | 0.0968513 | 0.679161 | H200004067 | SNTA1 | syntrophin, alpha 1 | 6640 |
| 17531 | -3.89704 | 0.1343204 | 1.66E-03 | 0.0920659 | 0.680174 | H200007667 | ARNT | aryl hydrocarbon receptor nuclear translocator | 405 |
| 8474 | -5.09298 | 0.0995281 | 2.56E-04 | 0.0633652 | 0.682007 | H200011896 | GABRD | gamma-aminobutyric acid (GABA) A receptor, delta | 2563 |
| 8196 | -5.2089 | 0.1330945 | 2.12E-04 | 0.0633652 | 0.682326 | H200020363 | SPTBN1 | spectrin, beta, non-erythrocytic 1 | 6711 |
| 2697 | -4.57057 | 0.1514928 | 5.57E-04 | 0.0721858 | 0.683659 | H200019718 | GTPBP3 | GTP binding protein 3 (mitochondrial) | 84705 |
| 3261 | -3.87333 | 0.1114014 | 1.73E-03 | 0.0929964 | 0.685082 | H200003010 | DCLK1 | doublecortin-like kinase 1 | 9201 |
| 20056 | -3.58426 | 0.1460178 | 2.84E-03 | 0.101854 | 0.687095 | H200003118 | N4BP2L1 | NEDD4 binding protein 2-like 1 | 90634 |
| 144 | -3.82403 | 0.0653578 | 1.88E-03 | 0.0959212 | 0.688503 | H200006638 | VEGFC | vascular endothelial growth factor C | 7424 |
| 15496 | -3.59964 | 0.1980768 | 2.76E-03 | 0.1014468 | 0.693791 | H200019649 | SF3A1 | splicing factor 3a, subunit 1, 120kDa | 10291 |
| 1405 | -3.35232 | 0.0757201 | 4.28E-03 | 0.1090499 | 0.693967 | H200001775 | OXNAD1 | oxidoreductase NAD-binding domain containing 1 | 92106 |
| 18419 | -3.44512 | 0.1188713 | 3.63E-03 | 0.1052221 | 0.699044 | H200007369 | NA | NA | - |
| 8553 | -3.35729 | 0.1913041 | 4.24E-03 | 0.1090499 | 0.702734 | H200015690 | C19orf43 | chromosome 19 open reading frame 43 | 79002 |
| 11977 | -3.64619 | 0.0748748 | 2.54E-03 | 0.098667 | 0.702963 | H200004669 | PKN3 | protein kinase N3 | 29941 |
| 835 | -3.50929 | 0.1335449 | 3.24E-03 | 0.1036239 | 0.703908 | H200018079 | NA | NA | - |
| 10833 | -3.32102 | 0.0498069 | 4.52E-03 | 0.1105631 | 0.705889 | H200015500 | CAPRIN2 | caprin family member 2 | 65981 |
| 2107 | -4.33718 | 0.1285779 | 8.01E-04 | 0.077886 | 0.709368 | H200013329 | ARSB | arylsulfatase B | 411 |
| 15129 | -4.62025 | 0.1191287 | 5.15E-04 | 0.070732 | 0.713361 | H200002471 | KIAA1407 | KIAA1407 | 57577 |
| 1877 | -3.31994 | 0.1367478 | 4.53E-03 | 0.1105631 | 0.715872 | H200002333 | NA | NA | - |
| 7974 | -3.71481 | 0.0713732 | 2.27E-03 | 0.0979394 | 0.717564 | H200009747 | NA | NA | - |
| 637 | -3.36726 | 0.1606389 | 4.16E-03 | 0.1090499 | 0.718929 | H200008603 | SLC15A2 | solute carrier family 15 (H+/peptide transporter), member 2 | 6565 |
| 13454 | -3.35936 | 0.0803653 | 4.22E-03 | 0.1090499 | 0.726258 | H200009793 | NA | NA | - |
| 8077 | -3.90926 | 0.0814002 | 1.62E-03 | 0.0914557 | 0.783025 | H200014681 | NA | NA | - |
| 9895 | -3.46719 | 0.0647319 | 3.49E-03 | 0.1045639 | 0.797695 | H200014372 | CYP4F11 | cytochrome P450, family 4, subfamily F, polypeptide 11 | 57834 |
| 19874 | -3.73245 | 0.0745866 | 2.20E-03 | 0.0977916 | 0.858927 | H200016162 | TNFRSF10A | tumor necrosis factor receptor superfamily, member 10a | 8797 |
| 5519 | 3.84248 | 0.0468702 | 1.83E-03 | 0.0944911 | 1.227848 | H200001596 | TBCK | TBC1 domain containing kinase | 93627 |
| 13125 | 3.355437 | 0.0583529 | 4.25E-03 | 0.1090499 | 1.232795 | H200015630 | SCYL1 | SCY1-like 1 (S. cerevisiae) | 57410 |
| 21610 | 3.474985 | 0.1015325 | 3.44E-03 | 0.1045639 | 1.29288 | H200017017 | TFE3 | transcription factor binding to IGHM enhancer 3 | 7030 |
| 21094 | 3.96047 | 0.0589808 | 1.49E-03 | 0.0914041 | 1.304657 | H200014072 | PFKL | phosphofructokinase, liver | 5211 |
| 7141 | 3.246195 | 0.0405523 | 5.17E-03 | 0.1139264 | 1.305431 | H200013649 | RHPN1 | rhophilin, Rho GTPase binding protein 1 | 114822 |
| 13319 | 4.20041 | 0.061531 | 1.00E-03 | 0.0839069 | 1.317632 | H200003351 | SAFB | scaffold attachment factor B | 6294 |
| 16769 | 3.925535 | 0.0798115 | 1.58E-03 | 0.0914041 | 1.327995 | H200015201 | HMGXB3 | HMG box domain containing 3 | 22993 |
| 10129 | 3.208299 | 0.0516494 | 5.54E-03 | 0.1175692 | 1.349602 | H200003483 | CEP170 | centrosomal protein 170kDa | 9859 |
| 14678 | 3.391303 | 0.0602679 | 3.99E-03 | 0.1069886 | 1.355395 | H200002430 | UBAP2 | ubiquitin associated protein 2 | 55833 |
| 12358 | 6.442894 | 0.0643243 | 4.25E-05 | 0.0482589 | 1.384756 | H200001018 | PIH1D1 | PIH1 domain containing 1 | 55011 |
| 2323 | 3.435595 | 0.0863294 | 3.69E-03 | 0.1056609 | 1.389932 | H200001882 | NA | NA | 56930 |
| 8264 | 3.323949 | 0.1358689 | 4.49E-03 | 0.1102207 | 1.393153 | H200001708 | BCORL1 | BCL6 corepressor-like 1 | 63035 |
| 13637 | 4.49571 | 0.0391645 | 6.23E-04 | 0.076403 | 1.42469 | H200018527 | NA | NA | - |
| 14212 | 3.665503 | 0.1268877 | 2.46E-03 | 0.0979394 | 1.440862 | H200002229 | ZNF205 | zinc finger protein 205 | 7755 |
| 19576 | 3.225178 | 0.0781954 | 5.37E-03 | 0.1157521 | 1.441735 | H200001794 | ABCF1 | ATP-binding cassette, sub-family F (GCN20), member 1 | 23 |
| 7896 | 3.303939 | 0.0701051 | 4.66E-03 | 0.1118241 | 1.446436 | H200005971 | POLD2 | polymerase (DNA directed), delta 2, accessory subunit | 5425 |
| 19518 | 3.509114 | 0.0910432 | 3.24E-03 | 0.1036239 | 1.446754 | H200020675 | LINC00477 | long intergenic non-protein coding RNA 477 | 144360 |
| 16480 | 3.981006 | 0.114177 | 1.43E-03 | 0.0909941 | 1.448095 | H200001219 | JTB | jumping translocation breakpoint | 10899 |
| 1117 | 3.479115 | 0.0781042 | 3.41E-03 | 0.1045639 | 1.450112 | H200009660 | YKT6 | YKT6 v-SNARE homolog (S. cerevisiae) | 10652 |
| 16588 | 4.038166 | 0.0916231 | 1.31E-03 | 0.0885433 | 1.454926 | H200006491 | MLX | MLX, MAX dimerization protein | 6945 |
| 17476 | 3.803357 | 0.0787199 | 1.94E-03 | 0.0968513 | 1.461312 | H200005025 | CUL7 | cullin 7 | 9820 |
| 20127 | 3.242321 | 0.066248 | 5.20E-03 | 0.1140536 | 1.465578 | H200007226 | NA | NA | - |
| 1500 | 3.4632 | 0.0672435 | 3.51E-03 | 0.1046203 | 1.475789 | H200006317 | LSM4 | LSM4 homolog, U6 small nuclear RNA associated (S. cerevisiae) | 25804 |
| 13533 | 3.290931 | 0.109056 | 4.77E-03 | 0.1118241 | 1.477207 | H200013587 | PPRC1 | peroxisome proliferator-activated receptor gamma, coactivator-related 1 | 23082 |
| 21375 | 3.399528 | 0.1008196 | 3.93E-03 | 0.1064864 | 1.480313 | H200005671 | DDX31 | DEAD (Asp-Glu-Ala-Asp) box polypeptide 31 | 64794 |
| 1444 | 3.270556 | 0.0902847 | 4.94E-03 | 0.1129163 | 1.492098 | H200003657 | KIF21A | kinesin family member 21A | 55605 |
| 6178 | 6.450091 | 0.0284733 | 4.20E-05 | 0.0482589 | 1.495972 | H200011363 | NA | NA | - |
| 4562 | 3.655674 | 0.1194329 | 2.50E-03 | 0.0979394 | 1.506498 | H200011103 | TCF3 | transcription factor 3 | 6929 |
| 21113 | 3.432789 | 0.1801881 | 3.71E-03 | 0.1058632 | 1.507764 | H200014862 | NA | NA | - |
| 15432 | 3.708849 | 0.1070993 | 2.29E-03 | 0.0979394 | 1.511617 | H200016609 | SNX4 | sorting nexin 4 | 8723 |
| 15750 | 4.224084 | 0.0785095 | 9.65E-04 | 0.0831898 | 1.534221 | H200010042 | MRPL34 | mitochondrial ribosomal protein L34 | 64981 |
| 16741 | 4.432938 | 0.0837177 | 6.90E-04 | 0.0765008 | 1.547166 | H200013729 | BCS1L | BC1 (ubiquinol-cytochrome c reductase) synthesis-like | 617 |
| 18057 | 3.840475 | 0.0426557 | 1.83E-03 | 0.0944911 | 1.568034 | H200011016 | PFKP | phosphofructokinase, platelet | 5214 |
| 12342 | 5.277563 | 0.0586202 | 1.92E-04 | 0.0633652 | 1.570525 | H200000258 | COIL | coilin | 8161 |
| 9345 | 3.642727 | 0.1284607 | 2.56E-03 | 0.0990044 | 1.574291 | H200009883 | FAM160B2 | family with sequence similarity 160, member B2 | 64760 |
| 10046 | 3.518434 | 0.1271819 | 3.18E-03 | 0.1035944 | 1.574996 | H200009498 | CYC1 | cytochrome c-1 | 1537 |
| 9275 | 3.970318 | 0.1230736 | 1.46E-03 | 0.0914041 | 1.577558 | H200006487 | NAP1L4 | nucleosome assembly protein 1-like 4 | 4676 |
| 9314 | 4.213317 | 0.218007 | 9.83E-04 | 0.0831898 | 1.579412 | H200008369 | TRIM14 | tripartite motif containing 14 | 9830 |
| 10048 | 3.428589 | 0.1328063 | 3.74E-03 | 0.1058952 | 1.590595 | H200009498 | CYC1 | cytochrome c-1 | 1537 |
| 10050 | 3.405468 | 0.138565 | 3.89E-03 | 0.1058952 | 1.614234 | H200009498 | CYC1 | cytochrome c-1 | 1537 |
| 2769 | 3.248536 | 0.0340266 | 5.15E-03 | 0.1138284 | 1.615229 | H200001205 | NT5DC3 | 5'-nucleotidase domain containing 3 | 51559 |
| 17042 | 5.081 | 0.1674439 | 2.61E-04 | 0.0633652 | 1.626808 | H200006414 | ARF5 | ADP-ribosylation factor 5 | 381 |
| 4733 | 3.670528 | 0.254182 | 2.44E-03 | 0.0979394 | 1.626824 | H200007759 | UBE2Z | ubiquitin-conjugating enzyme E2Z | 65264 |
| 7555 | 3.258845 | 0.0544882 | 5.05E-03 | 0.1129929 | 1.629206 | H200011522 | FAM115A | family with sequence similarity 115, member A | 9747 |
| 15857 | 3.335829 | 0.0660457 | 4.40E-03 | 0.1095482 | 1.631058 | H200015308 | PAPD7 | PAP associated domain containing 7 | 11044 |
| 7764 | 3.788976 | 0.1262852 | 1.99E-03 | 0.0968513 | 1.639518 | H200009498 | CYC1 | cytochrome c-1 | 1537 |
| 13696 | 4.153433 | 0.0786789 | 1.08E-03 | 0.0839146 | 1.641191 | H200021217 | NA | NA | - |
| 2276 | 3.418215 | 0.1390559 | 3.81E-03 | 0.1058952 | 1.641605 | H200021315 | SNF8 | SNF8, ESCRT-II complex subunit, homolog (S. cerevisiae) | 11267 |
| 7768 | 3.545363 | 0.1456976 | 3.04E-03 | 0.1027813 | 1.642449 | H200009498 | CYC1 | cytochrome c-1 | 1537 |
| 12226 | 3.444011 | 0.2124591 | 3.64E-03 | 0.1052221 | 1.647987 | H200016455 | DNPEP | aspartyl aminopeptidase | 23549 |
| 19288 | 5.153437 | 0.0675968 | 2.33E-04 | 0.0633652 | 1.650625 | H200009679 | NA | NA | - |
| 18829 | 3.362541 | 0.1020817 | 4.20E-03 | 0.1090499 | 1.650802 | H200008176 | NIPSNAP1 | nipsnap homolog 1 (C. elegans) | 8508 |
| 2970 | 3.337728 | 0.3655622 | 4.38E-03 | 0.1095379 | 1.653399 | H200010711 | LSR | lipolysis stimulated lipoprotein receptor | 51599 |
| 1937 | 3.302345 | 0.1533079 | 4.67E-03 | 0.1118241 | 1.653806 | H200005325 | ZNF689 | zinc finger protein 689 | 115509 |
| 593 | 3.281723 | 0.1224697 | 4.85E-03 | 0.1122832 | 1.657836 | H200006655 | KIAA0232 | KIAA0232 | 9778 |
| 8766 | 4.015429 | 0.1472725 | 1.35E-03 | 0.0902548 | 1.659666 | H200004059 | ANKRD6 | ankyrin repeat domain 6 | 22881 |
| 427 | 3.513681 | 0.1453432 | 3.21E-03 | 0.1035944 | 1.659689 | H200020264 | MBD6 | methyl-CpG binding domain protein 6 | 114785 |
| 1613 | 4.973316 | 0.068617 | 3.06E-04 | 0.0633652 | 1.660159 | H200011655 | PAIP1 | poly(A) binding protein interacting protein 1 | 10605 |
| 8811 | 3.279277 | 0.2042674 | 4.87E-03 | 0.1122832 | 1.664726 | H200006309 | ZDHHC16 | zinc finger, DHHC-type containing 16 | 84287 |
| 14891 | 3.296162 | 0.1821219 | 4.72E-03 | 0.1118241 | 1.665849 | H200012660 | NA | NA | - |
| 10027 | 3.917848 | 0.0993486 | 1.60E-03 | 0.0914041 | 1.670135 | H200020500 | MSL1 | male-specific lethal 1 homolog (Drosophila) | 339287 |
| 5612 | 3.508253 | 0.1109286 | 3.24E-03 | 0.1036239 | 1.676035 | H200006114 | ATP11B | ATPase, class VI, type 11B | 23200 |
| 12951 | 3.668045 | 0.1014627 | 2.45E-03 | 0.0979394 | 1.68642 | H200007294 | ZNF184 | zinc finger protein 184 | 7738 |
| 3986 | 5.071962 | 0.0865342 | 2.64E-04 | 0.0633652 | 1.693961 | H200015685 | SCRIB | scribbled homolog (Drosophila) | 23513 |
| 12283 | 4.143549 | 0.1588344 | 1.10E-03 | 0.0842736 | 1.696165 | H200019133 | WDR18 | WD repeat domain 18 | 57418 |
| 12369 | 3.66346 | 0.0947404 | 2.47E-03 | 0.0979394 | 1.696382 | H200001724 | EXTL3 | exostoses (multiple)-like 3 | 2137 |
| 4109 | 5.48794 | 0.0567403 | 1.44E-04 | 0.0630916 | 1.701067 | H200007830 | GAPDH | glyceraldehyde-3-phosphate dehydrogenase | 2597 |
| 10052 | 3.324025 | 0.16068 | 4.49E-03 | 0.1102207 | 1.702773 | H200009498 | CYC1 | cytochrome c-1 | 1537 |
| 4107 | 4.920219 | 0.0629844 | 3.33E-04 | 0.0633652 | 1.705869 | H200007830 | GAPDH | glyceraldehyde-3-phosphate dehydrogenase | 2597 |
| 4111 | 5.668321 | 0.0550231 | 1.12E-04 | 0.0630916 | 1.706321 | H200007830 | GAPDH | glyceraldehyde-3-phosphate dehydrogenase | 2597 |
| 3237 | 3.445315 | 0.1005788 | 3.63E-03 | 0.1052221 | 1.709142 | H200001870 | MRPL17 | mitochondrial ribosomal protein L17 | 63875 |
| 15930 | 3.241985 | 0.1965096 | 5.21E-03 | 0.1140536 | 1.71161 | H200018734 | ZNF554 | zinc finger protein 554 | 115196 |
| 3218 | 3.285963 | 0.1046739 | 4.81E-03 | 0.1118241 | 1.716647 | H200001068 | YTHDF3 | YTH domain family, member 3 | 253943 |
| 9617 | 3.455716 | 0.0917246 | 3.56E-03 | 0.1052221 | 1.717549 | H200001096 | NA | NA | - |
| 4203 | 3.440199 | 0.091468 | 3.66E-03 | 0.1052221 | 1.721447 | H200004398 | SMAD5 | SMAD family member 5 | 4090 |
| 12672 | 3.552688 | 0.1545369 | 3.00E-03 | 0.1026076 | 1.722882 | H200015862 | TWSG1 | twisted gastrulation homolog 1 (Drosophila) | 57045 |
| 4105 | 4.911832 | 0.0661521 | 3.37E-04 | 0.0633652 | 1.726616 | H200007830 | GAPDH | glyceraldehyde-3-phosphate dehydrogenase | 2597 |
| 21103 | 3.608775 | 0.0474972 | 2.72E-03 | 0.1014468 | 1.728895 | H200014458 | SUGP2 | SURP and G patch domain containing 2 | 10147 |
| 68 | 3.530242 | 0.0509155 | 3.12E-03 | 0.1035944 | 1.73138 | H200003170 | ASUN | asunder, spermatogenesis regulator | 55726 |
| 20854 | 3.29021 | 0.0324945 | 4.77E-03 | 0.1118241 | 1.736303 | H200002672 | CCDC14 | coiled-coil domain containing 14 | 64770 |
| 10137 | 3.743119 | 0.0769781 | 2.16E-03 | 0.0968513 | 1.740534 | H200003863 | ZNF449 | zinc finger protein 449 | 203523 |
| 13388 | 4.463114 | 0.0954706 | 6.58E-04 | 0.0765008 | 1.746923 | H200006445 | AP3M2 | adaptor-related protein complex 3, mu 2 subunit | 10947 |
| 7766 | 4.610517 | 0.1067172 | 5.22E-04 | 0.070732 | 1.756174 | H200009498 | CYC1 | cytochrome c-1 | 1537 |
| 7762 | 5.013257 | 0.1030104 | 2.89E-04 | 0.0633652 | 1.76272 | H200009498 | CYC1 | cytochrome c-1 | 1537 |
| 18523 | 4.047535 | 0.0955947 | 1.29E-03 | 0.0883163 | 1.763245 | H200013063 | POMT2 | protein-O-mannosyltransferase 2 | 29954 |
| 16293 | 3.445379 | 0.1443215 | 3.63E-03 | 0.1052221 | 1.764456 | H200014311 | TCEB1 | transcription elongation factor B (SIII), polypeptide 1 (15kDa, elongin C) | 6921 |
| 6869 | 3.520917 | 0.1056288 | 3.17E-03 | 0.1035944 | 1.765007 | H200000729 | ZNF74 | zinc finger protein 74 | 7625 |
| 5937 | 4.775344 | 0.0698414 | 4.10E-04 | 0.065389 | 1.773221 | H200007830 | GAPDH | glyceraldehyde-3-phosphate dehydrogenase | 2597 |
| 2452 | 4.176826 | 0.1007495 | 1.04E-03 | 0.0839146 | 1.773332 | H200007968 | CTBP2 | C-terminal binding protein 2 | 1488 |
| 17383 | 3.779883 | 0.1069012 | 2.02E-03 | 0.0968513 | 1.774558 | H200000779 | PPP4R1 | protein phosphatase 4, regulatory subunit 1 | 9989 |
| 6924 | 3.919671 | 0.1633418 | 1.59E-03 | 0.0914041 | 1.786891 | H200003371 | VEZT | vezatin, adherens junctions transmembrane protein | 55591 |
| 16496 | 4.150724 | 0.1117819 | 1.09E-03 | 0.0839146 | 1.788257 | H200001979 | ATG5 | autophagy related 5 | 9474 |
| 12515 | 3.744408 | 0.1277602 | 2.15E-03 | 0.0968513 | 1.792073 | H200008588 | RPS2 | ribosomal protein S2 | 6187 |
| 740 | 3.323707 | 0.0985912 | 4.49E-03 | 0.1102207 | 1.792099 | H200013525 | RBM39 | RNA binding motif protein 39 | 9584 |
| 5933 | 4.327434 | 0.0800513 | 8.17E-04 | 0.0781359 | 1.798099 | H200007830 | GAPDH | glyceraldehyde-3-phosphate dehydrogenase | 2597 |
| 21269 | 3.466032 | 0.0690577 | 3.49E-03 | 0.1045639 | 1.799967 | H200000707 | RRM1 | ribonucleotide reductase M1 | 6240 |
| 7284 | 4.103739 | 0.1530168 | 1.17E-03 | 0.086144 | 1.800483 | H200020471 | RNF187 | ring finger protein 187 | 149603 |
| 5935 | 4.103765 | 0.0850254 | 1.17E-03 | 0.086144 | 1.801155 | H200007830 | GAPDH | glyceraldehyde-3-phosphate dehydrogenase | 2597 |
| 4862 | 4.193897 | 0.1052005 | 1.01E-03 | 0.0839146 | 1.802505 | H200013845 | SPATS2 | spermatogenesis associated, serine-rich 2 | 65244 |
| 591 | 3.328195 | 0.2103584 | 4.46E-03 | 0.1100195 | 1.80314 | H200006347 | CDC34 | cell division cycle 34 | 997 |
| 11471 | 3.435248 | 0.369001 | 3.70E-03 | 0.1056609 | 1.806589 | H200002164 | PARP12 | poly (ADP-ribose) polymerase family, member 12 | 64761 |
| 19260 | 4.433966 | 0.0785596 | 6.88E-04 | 0.0765008 | 1.81344 | H200008491 | DCTN4 | dynactin 4 (p62) | 51164 |
| 5550 | 3.864089 | 0.0654859 | 1.76E-03 | 0.0934933 | 1.815955 | H200003098 | C1QTNF6 | C1q and tumor necrosis factor related protein 6 | 114904 |
| 8423 | 6.339569 | 0.0717528 | 4.84E-05 | 0.0482589 | 1.817653 | H200009302 | NA | NA | - |
| 18184 | 3.450771 | 0.1209535 | 3.60E-03 | 0.1052221 | 1.820814 | H200017090 | NDUFA6 | NADH dehydrogenase (ubiquinone) 1 alpha subcomplex, 6, 14kDa | 4700 |
| 11784 | 5.09526 | 0.0301733 | 2.55E-04 | 0.0633652 | 1.821188 | H200016990 | ALDOA | aldolase A, fructose-bisphosphate | 226 |
| 10292 | 4.075731 | 0.0731624 | 1.23E-03 | 0.0874811 | 1.835226 | H200011113 | CUL5 | cullin 5 | 8065 |
| 15551 | 3.319111 | 0.1307368 | 4.53E-03 | 0.1105631 | 1.835624 | H200000560 | CEBPG | CCAAT/enhancer binding protein (C/EBP), gamma | 1054 |
| 5242 | 4.184757 | 0.1045141 | 1.03E-03 | 0.0839146 | 1.839527 | H200010472 | KHSRP | KH-type splicing regulatory protein | 8570 |
| 10572 | 5.191231 | 0.0950397 | 2.19E-04 | 0.0633652 | 1.852068 | H200002990 | FBXW11 | F-box and WD repeat domain containing 11 | 23291 |
| 14190 | 3.74979 | 0.0873898 | 2.13E-03 | 0.0968513 | 1.85537 | H200001113 | NA | NA | - |
| 7464 | 4.425806 | 0.0734543 | 6.97E-04 | 0.0765008 | 1.856493 | H200007016 | ANKIB1 | ankyrin repeat and IBR domain containing 1 | 54467 |
| 15226 | 3.482358 | 0.1805226 | 3.39E-03 | 0.1045639 | 1.857767 | H200007037 | NFE2L1 | nuclear factor (erythroid-derived 2)-like 1 | 4779 |
| 6614 | 3.341111 | 0.1340047 | 4.36E-03 | 0.1095379 | 1.865365 | H200010282 | MAPK8IP3 | mitogen-activated protein kinase 8 interacting protein 3 | 23162 |
| 12439 | 5.857146 | 0.0518612 | 8.58E-05 | 0.0630916 | 1.868323 | H200004836 | SREK1 | splicing regulatory glutamine/lysine-rich protein 1 | 140890 |
| 16617 | 4.954774 | 0.1247019 | 3.17E-04 | 0.0633652 | 1.873055 | H200007981 | SKP1 | S-phase kinase-associated protein 1 | 6500 |
| 16118 | 3.336887 | 0.1943624 | 4.39E-03 | 0.1095379 | 1.873543 | H200005957 | PUF60 | poly-U binding splicing factor 60KDa | 22827 |
| 5939 | 5.129795 | 0.0713863 | 2.42E-04 | 0.0633652 | 1.877275 | H200007830 | GAPDH | glyceraldehyde-3-phosphate dehydrogenase | 2597 |
| 2590 | 3.561535 | 0.0854704 | 2.95E-03 | 0.1024962 | 1.884156 | H200014736 | NA | NA | - |
| 15427 | 3.476045 | 0.2899191 | 3.43E-03 | 0.1045639 | 1.885613 | H200016555 | IFI6 | interferon, alpha-inducible protein 6 | 2537 |
| 12622 | 3.963703 | 0.1670078 | 1.48E-03 | 0.0914041 | 1.890798 | H200013558 | ZFP64 | ZFP64 zinc finger protein | 55734 |
| 15260 | 3.573479 | 0.0738547 | 2.89E-03 | 0.1024962 | 1.899057 | H200008581 | TRAP1 | TNF receptor-associated protein 1 | 10131 |
| 2803 | 3.348774 | 0.1441656 | 4.30E-03 | 0.1090499 | 1.900873 | H200002749 | LMAN2L | lectin, mannose-binding 2-like | 81562 |
| 21365 | 5.468438 | 0.2095275 | 1.49E-04 | 0.0630916 | 1.901619 | H200005267 | KEAP1 | kelch-like ECH-associated protein 1 | 9817 |
| 11931 | 3.653909 | 0.2402024 | 2.51E-03 | 0.0979394 | 1.902846 | H200002413 | SLCO3A1 | solute carrier organic anion transporter family, member 3A1 | 28232 |
| 12387 | 3.259388 | 0.1500642 | 5.05E-03 | 0.1129929 | 1.90507 | H200002508 | NA | NA | - |
| 5456 | 3.392474 | 0.1456218 | 3.98E-03 | 0.1069886 | 1.917087 | H200020424 | NA | NA | - |
| 19684 | 3.913668 | 0.0727212 | 1.61E-03 | 0.0914041 | 1.919594 | H200007066 | TPI1 | triosephosphate isomerase 1 | 7167 |
| 6897 | 3.536044 | 0.0778178 | 3.09E-03 | 0.1035911 | 1.920397 | H200002201 | ACTR3B | ARP3 actin-related protein 3 homolog B (yeast) | 57180 |
| 1838 | 3.96416 | 0.1680268 | 1.48E-03 | 0.0914041 | 1.939749 | H200000439 | GNA11 | guanine nucleotide binding protein (G protein), alpha 11 (Gq class) | 2767 |
| 20552 | 5.848779 | 0.076188 | 8.68E-05 | 0.0630916 | 1.943006 | H200008651 | ANAPC11 | anaphase promoting complex subunit 11 | 51529 |
| 20526 | 4.094232 | 0.1426701 | 1.19E-03 | 0.0865608 | 1.944316 | H200007155 | ATP5I | ATP synthase, H+ transporting, mitochondrial Fo complex, subunit E | 521 |
| 13564 | 3.843431 | 0.1979992 | 1.82E-03 | 0.0944911 | 1.946319 | H200014805 | SRC | v-src sarcoma (Schmidt-Ruppin A-2) viral oncogene homolog (avian) | 6714 |
| 11664 | 3.220128 | 0.0668875 | 5.41E-03 | 0.1160087 | 1.955679 | H200011290 | CAP1 | CAP, adenylate cyclase-associated protein 1 (yeast) | 10487 |
| 21045 | 4.415099 | 0.0822134 | 7.09E-04 | 0.0766926 | 1.959534 | H200011490 | LPHN1 | latrophilin 1 | 22859 |
| 7376 | 3.377018 | 0.1606285 | 4.09E-03 | 0.1082123 | 1.967449 | H200002836 | FAM189B | family with sequence similarity 189, member B | 10712 |
| 20905 | 3.425696 | 0.1286143 | 3.76E-03 | 0.1058952 | 1.972031 | H200004982 | RB1CC1 | RB1-inducible coiled-coil 1 | 9821 |
| 20705 | 3.952462 | 0.0986277 | 1.51E-03 | 0.0914041 | 1.973061 | H200016703 | NA | NA | - |
| 13883 | 4.923114 | 0.1270453 | 3.31E-04 | 0.0633652 | 1.975337 | H200008066 | AHCY | adenosylhomocysteinase | 191 |
| 13982 | 3.298317 | 0.3455447 | 4.71E-03 | 0.1118241 | 1.979394 | H200012940 | AKIP1 | A kinase (PRKA) interacting protein 1 | 56672 |
| 7192 | 3.416306 | 0.2172892 | 3.82E-03 | 0.1058952 | 1.982021 | H200015959 | TGM6 | transglutaminase 6 | 343641 |
| 6661 | 6.613543 | 0.2038296 | 3.50E-05 | 0.0482589 | 1.984647 | H200012556 | THY1 | Thy-1 cell surface antigen | 7070 |
| 14916 | 5.429487 | 0.1079825 | 1.56E-04 | 0.0630916 | 1.989317 | H200013806 | FBXL19 | F-box and leucine-rich repeat protein 19 | 54620 |
| 12932 | 4.919605 | 0.0940857 | 3.33E-04 | 0.0633652 | 1.992701 | H200006492 | SMARCA4 | SWI/SNF related, matrix associated, actin dependent regulator of chromatin, subfamily a, member 4 | 6597 |
| 21083 | 3.569851 | 0.0754302 | 2.91E-03 | 0.1024962 | 1.998912 | H200013366 | NREP | neuronal regeneration related protein | 9315 |
| 14867 | 3.485396 | 0.17628 | 3.37E-03 | 0.1045639 | 2.004831 | H200011520 | NAB1 | NGFI-A binding protein 1 (EGR1 binding protein 1) | 4664 |
| 17895 | 4.528729 | 0.128851 | 5.93E-04 | 0.0745449 | 2.009071 | H200003392 | NUFIP2 | nuclear fragile X mental retardation protein interacting protein 2 | 57532 |
| 15720 | 5.541311 | 0.0926034 | 1.34E-04 | 0.0630916 | 2.018374 | H200008546 | ARIH1 | ariadne homolog, ubiquitin-conjugating enzyme E2 binding protein, 1 (Drosophila) | 25820 |
| 3627 | 3.573564 | 0.0762875 | 2.89E-03 | 0.1024962 | 2.025811 | H200020466 | CCDC43 | coiled-coil domain containing 43 | 124808 |
| 12398 | 3.60906 | 0.0743285 | 2.72E-03 | 0.1014468 | 2.028853 | H200002918 | TUBGCP4 | tubulin, gamma complex associated protein 4 | 27229 |
| 3116 | 3.982663 | 0.1199749 | 1.43E-03 | 0.0909941 | 2.031564 | H200017575 | TMEM259 | transmembrane protein 259 | 91304 |
| 3863 | 3.406626 | 0.1573923 | 3.88E-03 | 0.1058952 | 2.050409 | H200009671 | SGK1 | serum/glucocorticoid regulated kinase 1 | 6446 |
| 2895 | 3.702384 | 0.0687916 | 2.32E-03 | 0.0979394 | 2.064755 | H200007261 | ERI2 | ERI1 exoribonuclease family member 2 | 112479 |
| 17386 | 4.909887 | 0.0487923 | 3.38E-04 | 0.0633652 | 2.0737 | H200000821 | STRAP | serine/threonine kinase receptor associated protein | 11171 |
| 16819 | 4.393075 | 0.0864623 | 7.34E-04 | 0.0766926 | 2.078582 | H200017505 | FANCL | Fanconi anemia, complementation group L | 55120 |
| 3603 | 3.52566 | 0.1612138 | 3.14E-03 | 0.1035944 | 2.081845 | H200019326 | BTBD10 | BTB (POZ) domain containing 10 | 84280 |
| 4694 | 3.278695 | 0.1993325 | 4.87E-03 | 0.1122832 | 2.113874 | H200005865 | MIF | macrophage migration inhibitory factor (glycosylation-inhibiting factor) | 4282 |
| 12811 | 3.608116 | 0.0802887 | 2.72E-03 | 0.1014468 | 2.122062 | H200000786 | CDK2AP1 | cyclin-dependent kinase 2 associated protein 1 | 8099 |
| 5102 | 3.305205 | 0.346122 | 4.65E-03 | 0.1118241 | 2.124391 | H200003680 | ARHGAP10 | Rho GTPase activating protein 10 | 79658 |
| 19556 | 3.409359 | 0.1659467 | 3.87E-03 | 0.1058952 | 2.129461 | H200000986 | CPD | carboxypeptidase D | 1362 |
| 10748 | 3.959722 | 0.1350035 | 1.49E-03 | 0.0914041 | 2.132016 | H200011350 | TK1 | thymidine kinase 1, soluble | 7083 |
| 2814 | 3.917862 | 0.1312662 | 1.60E-03 | 0.0914041 | 2.134584 | H200003443 | CACHD1 | cache domain containing 1 | 57685 |
| 10056 | 4.125395 | 0.1847306 | 1.14E-03 | 0.085653 | 2.14499 | H200000045 | TNFRSF1A | tumor necrosis factor receptor superfamily, member 1A | 7132 |
| 8795 | 3.256766 | 0.126876 | 5.07E-03 | 0.1132076 | 2.147619 | H200005549 | ATP5H | ATP synthase, H+ transporting, mitochondrial Fo complex, subunit d | 10476 |
| 1835 | 3.489356 | 0.0922421 | 3.35E-03 | 0.1045639 | 2.169708 | H200000409 | FGD1 | FYVE, RhoGEF and PH domain containing 1 | 2245 |
| 4025 | 3.986842 | 0.127117 | 1.42E-03 | 0.0909941 | 2.193973 | H200017579 | TMEM68 | transmembrane protein 68 | 137695 |
| 1586 | 3.253173 | 0.1728861 | 5.10E-03 | 0.1135172 | 2.196959 | H200010473 | JAG1 | jagged 1 | 182 |
| 20755 | 3.208084 | 0.2136033 | 5.54E-03 | 0.1175692 | 2.206895 | H200019671 | KIAA1109 | KIAA1109 | 84162 |
| 19704 | 5.755325 | 0.3261768 | 1.00E-04 | 0.0630916 | 2.207702 | H200007874 | UBE2L6 | ubiquitin-conjugating enzyme E2L 6 | 9246 |
| 7758 | 3.595799 | 0.1611638 | 2.78E-03 | 0.1014483 | 2.207877 | H200021052 | NA | NA | - |
| 20596 | 4.365624 | 0.2318569 | 7.62E-04 | 0.077886 | 2.208238 | H200010955 | NA | NA | - |
| 20912 | 6.791781 | 0.074979 | 2.78E-05 | 0.0482589 | 2.211664 | H200005356 | ZNF473 | zinc finger protein 473 | 25888 |
| 2040 | 3.920569 | 0.1310468 | 1.59E-03 | 0.0914041 | 2.211856 | H200009963 | ZNF629 | zinc finger protein 629 | 23361 |
| 11259 | 4.46364 | 0.1651593 | 6.57E-04 | 0.0765008 | 2.221906 | H200013801 | ZBED3 | zinc finger, BED-type containing 3 | 84327 |
| 4052 | 3.292055 | 0.2541808 | 4.76E-03 | 0.1118241 | 2.227974 | H200018749 | NA | NA | - |
| 10458 | 3.417775 | 0.1552298 | 3.81E-03 | 0.1058952 | 2.229321 | H200019069 | NA | NA | - |
| 9977 | 3.655348 | 0.151707 | 2.51E-03 | 0.0979394 | 2.231918 | H200018196 | NA | NA | 64163 |
| 16287 | 3.796016 | 0.1077303 | 1.97E-03 | 0.0968513 | 2.23288 | H200013955 | DLG1 | discs, large homolog 1 (Drosophila) | 1739 |
| 18684 | 5.570489 | 0.537827 | 1.28E-04 | 0.0630916 | 2.242975 | H200000220 | ISG15 | ISG15 ubiquitin-like modifier | 9636 |
| 7126 | 3.263935 | 0.6396041 | 5.01E-03 | 0.1129929 | 2.251619 | H200012895 | WDR78 | WD repeat domain 78 | 79819 |
| 15848 | 3.754415 | 0.2979386 | 2.11E-03 | 0.0968513 | 2.251952 | H200014626 | PALLD | palladin, cytoskeletal associated protein | 23022 |
| 4586 | 3.408515 | 0.0814549 | 3.87E-03 | 0.1058952 | 2.258859 | H200000877 | PLS3 | plastin 3 | 5358 |
| 15756 | 3.231713 | 0.1445877 | 5.30E-03 | 0.1152663 | 2.263301 | H200010398 | CPOX | coproporphyrinogen oxidase | 1371 |
| 8769 | 3.3882 | 0.070576 | 4.01E-03 | 0.1069886 | 2.264774 | H200004385 | MPP3 | membrane protein, palmitoylated 3 (MAGUK p55 subfamily member 3) | 4356 |
| 7110 | 3.342309 | 0.0941034 | 4.35E-03 | 0.1095379 | 2.267608 | H200012135 | NME1 | NME/NM23 nucleoside diphosphate kinase 1 | 4830 |
| 2468 | 3.751626 | 0.0708955 | 2.13E-03 | 0.0968513 | 2.275109 | H200008728 | FAM115A | family with sequence similarity 115, member A | 9747 |
| 542 | 3.682409 | 0.1751737 | 2.40E-03 | 0.0979394 | 2.278705 | H200004049 | TMEM51 | transmembrane protein 51 | 55092 |
| 6533 | 3.570202 | 0.1345323 | 2.91E-03 | 0.1024962 | 2.29007 | H200006476 | SLC6A2 | solute carrier family 6 (neurotransmitter transporter, noradrenalin), member 2 | 6530 |
| 12444 | 3.891767 | 0.1869447 | 1.68E-03 | 0.0924157 | 2.296369 | H200005174 | GGPS1 | geranylgeranyl diphosphate synthase 1 | 9453 |
| 16076 | 4.23796 | 0.2017299 | 9.45E-04 | 0.0831898 | 2.300033 | H200004033 | HOOK2 | hook homolog 2 (Drosophila) | 29911 |
| 6567 | 4.232209 | 0.10146 | 9.54E-04 | 0.0831898 | 2.302973 | H200008020 | HN1L | hematological and neurological expressed 1-like | 90861 |
| 7679 | 3.395531 | 0.0824258 | 3.96E-03 | 0.1068092 | 2.307088 | H200017270 | NCKAP1 | NCK-associated protein 1 | 10787 |
| 6334 | 3.871136 | 0.0857581 | 1.74E-03 | 0.0929964 | 2.31129 | H200018915 | ZNRF3 | zinc and ring finger 3 | 84133 |
| 10375 | 3.442945 | 0.0941432 | 3.65E-03 | 0.1052221 | 2.34636 | H200015239 | ATAD2 | ATPase family, AAA domain containing 2 | 29028 |
| 4843 | 3.555603 | 0.6137869 | 2.98E-03 | 0.1024962 | 2.346969 | H200013055 | ARHGAP26 | Rho GTPase activating protein 26 | 23092 |
| 16151 | 4.826556 | 0.1182888 | 3.81E-04 | 0.065389 | 2.374427 | H200007495 | LGR4 | leucine-rich repeat containing G protein-coupled receptor 4 | 55366 |
| 19329 | 3.68935 | 0.1270998 | 2.37E-03 | 0.0979394 | 2.382483 | H200011585 | NA | NA | - |
| 8625 | 3.658523 | 0.0985925 | 2.49E-03 | 0.0979394 | 2.384935 | H200019110 | USP37 | ubiquitin specific peptidase 37 | 57695 |
| 14610 | 3.21852 | 0.2541817 | 5.43E-03 | 0.1160087 | 2.390278 | H200021205 | NA | NA | - |
| 20873 | 3.272112 | 0.243399 | 4.93E-03 | 0.1129163 | 2.415309 | H200003462 | FAM199X | family with sequence similarity 199, X-linked | 139231 |
| 546 | 3.770903 | 0.1336832 | 2.05E-03 | 0.0968513 | 2.41743 | H200004381 | LBX1 | ladybird homeobox 1 | 10660 |
| 12463 | 3.261022 | 0.1854218 | 5.03E-03 | 0.1129929 | 2.421165 | H200005976 | PSMD2 | proteasome (prosome, macropain) 26S subunit, non-ATPase, 2 | 5708 |
| 13725 | 4.602155 | 0.1156421 | 5.30E-04 | 0.070732 | 2.427408 | H200000774 | HDAC2 | histone deacetylase 2 | 3066 |
| 19230 | 3.581298 | 0.1435433 | 2.85E-03 | 0.1019649 | 2.433228 | H200006995 | COL5A2 | collagen, type V, alpha 2 | 1290 |
| 15817 | 3.465611 | 0.1577423 | 3.50E-03 | 0.1045639 | 2.463484 | H200013408 | GOLIM4 | golgi integral membrane protein 4 | 27333 |
| 662 | 3.532252 | 0.156196 | 3.11E-03 | 0.1035944 | 2.482331 | H200009749 | NA | NA | - |
| 15201 | 3.480213 | 0.2076304 | 3.40E-03 | 0.1045639 | 2.49868 | H200005891 | PNLIPRP1 | pancreatic lipase-related protein 1 | 5407 |
| 8635 | 3.448228 | 0.2514056 | 3.61E-03 | 0.1052221 | 2.519026 | H200019514 | FAM213A | family with sequence similarity 213, member A | 84293 |
| 12738 | 3.33302 | 0.2095763 | 4.42E-03 | 0.1097343 | 2.525282 | H200019210 | NA | NA | - |
| 11164 | 9.706987 | 0.0668464 | 1.79E-06 | 0.0232358 | 2.525546 | H200009247 | FAR1 | fatty acyl CoA reductase 1 | 84188 |
| 2958 | 3.629272 | 0.1847581 | 2.62E-03 | 0.1003799 | 2.542285 | H200010283 | SKA3 | spindle and kinetochore associated complex subunit 3 | 221150 |
| 14287 | 3.261419 | 0.774064 | 5.03E-03 | 0.1129929 | 2.545899 | H200005691 | FUT1 | fucosyltransferase 1 (galactoside 2-alpha-L-fucosyltransferase, H blood group) | 2523 |
| 7428 | 4.177063 | 0.0548862 | 1.04E-03 | 0.0839146 | 2.558753 | H200005448 | MYO10 | myosin X | 4651 |
| 7071 | 3.475934 | 0.7244687 | 3.43E-03 | 0.1045639 | 2.576429 | H200010253 | SDR42E1 | short chain dehydrogenase/reductase family 42E, member 1 | 93517 |
| 15777 | 4.753756 | 0.2788156 | 4.23E-04 | 0.065389 | 2.577885 | H200011508 | FAM135A | family with sequence similarity 135, member A | 57579 |
| 1226 | 3.598697 | 0.3653057 | 2.77E-03 | 0.1014468 | 2.578565 | H200014938 | BCL2L11 | BCL2-like 11 (apoptosis facilitator) | 10018 |
| 20358 | 3.58986 | 0.0979722 | 2.81E-03 | 0.101597 | 2.580035 | H200019766 | NA | NA | 81023 |
| 16546 | 4.354056 | 0.1377153 | 7.78E-04 | 0.077886 | 2.602692 | H200004567 | CEP70 | centrosomal protein 70kDa | 80321 |
| 6129 | 3.489236 | 0.4725864 | 3.35E-03 | 0.1045639 | 2.604291 | H200009077 | NA | NA | 148490 |
| 18258 | 3.20869 | 0.1313639 | 5.53E-03 | 0.1175692 | 2.613385 | H200020818 | NA | NA | 200107 |
| 12823 | 3.985287 | 0.3361903 | 1.42E-03 | 0.0909941 | 2.63932 | H200001214 | HS6ST1 | heparan sulfate 6-O-sulfotransferase 1 | 9394 |
| 18584 | 3.486292 | 0.0939023 | 3.37E-03 | 0.1045639 | 2.648513 | H200016459 | CLDN12 | claudin 12 | 9069 |
| 18029 | 3.352014 | 0.103079 | 4.28E-03 | 0.1090499 | 2.666509 | H200009828 | NA | NA | 203411 |
| 9884 | 3.701691 | 0.1650896 | 2.32E-03 | 0.0979394 | 2.691314 | H200013666 | KIF5B | kinesin family member 5B | 3799 |
| 21507 | 3.517383 | 0.3268717 | 3.19E-03 | 0.1035944 | 2.698829 | H200012083 | BST2 | bone marrow stromal cell antigen 2 | 684 |
| 18233 | 3.942852 | 0.1998568 | 1.53E-03 | 0.0914041 | 2.703236 | H200019376 | MGC2889 | uncharacterized protein MGC2889 | 84789 |
| 5187 | 4.218483 | 0.0977382 | 9.75E-04 | 0.0831898 | 2.71673 | H200007830 | GAPDH | glyceraldehyde-3-phosphate dehydrogenase | 2597 |
| 2571 | 3.36705 | 0.3707041 | 4.16E-03 | 0.1090499 | 2.719391 | H200013662 | KIAA1217 | KIAA1217 | 56243 |
| 14273 | 3.754064 | 0.2428886 | 2.12E-03 | 0.0968513 | 2.732799 | H200005239 | MCM2 | minichromosome maintenance complex component 2 | 4171 |
| 1097 | 4.679932 | 0.0796736 | 4.72E-04 | 0.0693846 | 2.766984 | H200008852 | AEBP2 | AE binding protein 2 | 121536 |
| 11600 | 4.152116 | 4.7683655 | 1.08E-03 | 0.0839146 | 2.816668 | H200008250 | FCGR3A | Fc fragment of IgG, low affinity IIIa, receptor (CD16a) | 2214 |
| 8592 | 5.243205 | 0.2050334 | 2.02E-04 | 0.0633652 | 2.834259 | H200017288 | OR1F1 | olfactory receptor, family 1, subfamily F, member 1 | 4992 |
| 5894 | 3.783558 | 0.2379421 | 2.01E-03 | 0.0968513 | 2.850279 | H200019438 | NA | NA | - |
| 5613 | 4.660333 | 0.458258 | 4.85E-04 | 0.0693846 | 2.85802 | H200006132 | DDR1 | discoidin domain receptor tyrosine kinase 1 | 780 |
| 3055 | 3.272788 | 0.272452 | 4.93E-03 | 0.1129163 | 2.864017 | H200014861 | FLJ31485 | uncharacterized LOC440119 | 440119 |
| 13541 | 3.771753 | 0.1852026 | 2.05E-03 | 0.0968513 | 2.928614 | H200013967 | FAM57A | family with sequence similarity 57, member A | 79850 |
| 18207 | 4.579101 | 0.2402349 | 5.50E-04 | 0.0721858 | 2.939327 | H200018212 | NA | NA | - |
| 12497 | 4.084561 | 0.1924829 | 1.21E-03 | 0.086962 | 2.94068 | H200007804 | FUT10 | fucosyltransferase 10 (alpha (1,3) fucosyltransferase) | 84750 |
| 8591 | 4.042636 | 0.3689625 | 1.30E-03 | 0.0883163 | 3.008196 | H200017282 | TSHZ3 | teashirt zinc finger homeobox 3 | 57616 |
| 20198 | 4.733851 | 0.162818 | 4.37E-04 | 0.0665029 | 3.024164 | H200011044 | DYNLRB1 | dynein, light chain, roadblock-type 1 | 83658 |
| 18813 | 3.481769 | 0.247325 | 3.39E-03 | 0.1045639 | 3.036676 | H200007102 | KCNB1 | potassium voltage-gated channel, Shab-related subfamily, member 1 | 3745 |
| 16020 | 3.417386 | 0.91995 | 3.81E-03 | 0.1058952 | 3.048873 | H200001373 | CMPK2 | cytidine monophosphate (UMP-CMP) kinase 2, mitochondrial | 129607 |
| 14096 | 3.349569 | 0.3380116 | 4.30E-03 | 0.1090499 | 3.062494 | H200018284 | NA | NA | - |
| 16211 | 4.392234 | 0.1617522 | 7.34E-04 | 0.0766926 | 3.077932 | H200010487 | LIN7C | lin-7 homolog C (C. elegans) | 55327 |
| 10776 | 3.465864 | 0.2251886 | 3.49E-03 | 0.1045639 | 3.082613 | H200012538 | NA | NA | - |
| 2934 | 3.537581 | 0.3135422 | 3.08E-03 | 0.1035911 | 3.090398 | H200009143 | NA | NA | - |
| 3558 | 3.667966 | 0.3162115 | 2.45E-03 | 0.0979394 | 3.096911 | H200017076 | EIF2AK2 | eukaryotic translation initiation factor 2-alpha kinase 2 | 5610 |
| 17888 | 8.12272 | 0.4338732 | 6.28E-06 | 0.0406626 | 3.111783 | H200003030 | STAT1 | signal transducer and activator of transcription 1, 91kDa | 6772 |
| 2051 | 4.913955 | 0.1001687 | 3.36E-04 | 0.0633652 | 3.137654 | H200010669 | TEAD4 | TEA domain family member 4 | 7004 |
| 4249 | 5.178345 | 0.5096793 | 2.24E-04 | 0.0633652 | 3.139966 | H200006654 | ST5 | suppression of tumorigenicity 5 | 6764 |
| 17839 | 4.156286 | 0.1038947 | 1.08E-03 | 0.0839146 | 3.150437 | H200000732 | HLTF | helicase-like transcription factor | 6596 |
| 12775 | 4.307469 | 0.31375 | 8.43E-04 | 0.0784907 | 3.156173 | H200020796 | NA | NA | 399986 |
| 4535 | 3.513747 | 0.2368097 | 3.21E-03 | 0.1035944 | 3.165418 | H200020026 | NA | NA | - |
| 10649 | 3.921752 | 1.2966678 | 1.59E-03 | 0.0914041 | 3.18885 | H200006760 | CX3CL1 | chemokine (C-X3-C motif) ligand 1 | 6376 |
| 11983 | 3.47039 | 0.253655 | 3.47E-03 | 0.1045639 | 3.194207 | H200004741 | KLHL32 | kelch-like family member 32 | 114792 |
| 3049 | 3.685238 | 0.3439777 | 2.39E-03 | 0.0979394 | 3.210351 | H200014505 | UGT1A6 | UDP glucuronosyltransferase 1 family, polypeptide A6 | 54578 |
| 17974 | 4.659637 | 0.0736257 | 4.86E-04 | 0.0693846 | 3.215878 | H200007186 | FAM64A | family with sequence similarity 64, member A | 54478 |
| 19412 | 3.884806 | 7.3901857 | 1.69E-03 | 0.0929649 | 3.249087 | H200015711 | GBP4 | guanylate binding protein 4 | 115361 |
| 17766 | 3.68635 | 0.3111897 | 2.38E-03 | 0.0979394 | 3.259008 | H200019013 | PLXNA1 | plexin A1 | 5361 |
| 21093 | 4.710497 | 0.3140285 | 4.50E-04 | 0.067816 | 3.269729 | H200013770 | CASK | calcium/calmodulin-dependent serine protein kinase (MAGUK family) | 8573 |
| 16337 | 4.062539 | 0.1418932 | 1.25E-03 | 0.0874811 | 3.282911 | H200016543 | NA | NA | - |
| 1685 | 3.988716 | 0.4174256 | 1.41E-03 | 0.0909941 | 3.287024 | H200015075 | PCDHGA4 | protocadherin gamma subfamily A, 4 | 56111 |
| 4067 | 4.28969 | 0.1382538 | 8.67E-04 | 0.0784907 | 3.288018 | H200019503 | CKM | creatine kinase, muscle | 1158 |
| 11106 | 3.233581 | 0.2983935 | 5.28E-03 | 0.1151813 | 3.343152 | H200006563 | ATP1B2 | ATPase, Na+/K+ transporting, beta 2 polypeptide | 482 |
| 13068 | 4.878972 | 0.1512482 | 3.52E-04 | 0.0648892 | 3.343817 | H200012952 | TEX14 | testis expressed 14 | 56155 |
| 11961 | 5.04451 | 0.1259381 | 2.76E-04 | 0.0633652 | 3.421491 | H200003909 | RBBP8 | retinoblastoma binding protein 8 | 5932 |
| 15877 | 3.524832 | 0.2558382 | 3.15E-03 | 0.1035944 | 3.509767 | H200016116 | FGF17 | fibroblast growth factor 17 | 8822 |
| 18003 | 3.669796 | 0.1376416 | 2.44E-03 | 0.0979394 | 3.514913 | H200008664 | NA | NA | - |
| 15460 | 4.770753 | 0.1670613 | 4.13E-04 | 0.065389 | 3.534939 | H200018081 | LINC00852 | long intergenic non-protein coding RNA 852 | 84657 |
| 528 | 3.382255 | 0.4935127 | 4.06E-03 | 0.1076268 | 3.547487 | H200003313 | NA | NA | - |
| 11548 | 3.472538 | 0.7213776 | 3.45E-03 | 0.1045639 | 3.565777 | H200005922 | GPR143 | G protein-coupled receptor 143 | 4935 |
| 21519 | 4.295465 | 0.3833786 | 8.58E-04 | 0.0784907 | 3.566132 | H200012511 | MARVELD2 | MARVEL domain containing 2 | 153562 |
| 9386 | 3.871776 | 0.240948 | 1.74E-03 | 0.0929964 | 3.574429 | H200011789 | NA | NA | - |
| 10493 | 3.772293 | 0.3534819 | 2.05E-03 | 0.0968513 | 3.577152 | H200020915 | NA | NA | - |
| 550 | 3.987123 | 0.2537824 | 1.42E-03 | 0.0909941 | 3.588061 | H200004429 | ADAMTS6 | ADAM metallopeptidase with thrombospondin type 1 motif, 6 | 11174 |
| 18120 | 3.559067 | 0.4210402 | 2.97E-03 | 0.1024962 | 3.59571 | H200014050 | EPHB4 | EPH receptor B4 | 2050 |
| 2443 | 3.485236 | 0.8195922 | 3.37E-03 | 0.1045639 | 3.60838 | H200007582 | CXCL6 | chemokine (C-X-C motif) ligand 6 | 6372 |
| 12094 | 3.774648 | 0.2317479 | 2.04E-03 | 0.0968513 | 3.609009 | H200010043 | SPR | sepiapterin reductase (7,8-dihydrobiopterin:NADP+ oxidoreductase) | 6697 |
| 6675 | 3.707055 | 0.3720117 | 2.30E-03 | 0.0979394 | 3.640904 | H200013008 | NOX1 | NADPH oxidase 1 | 27035 |
| 11969 | 3.609693 | 0.4426798 | 2.71E-03 | 0.1014468 | 3.667674 | H200004289 | LRRC1 | leucine rich repeat containing 1 | 55227 |
| 4879 | 4.812264 | 1.1234633 | 3.89E-04 | 0.065389 | 3.731215 | H200014623 | STAP2 | signal transducing adaptor family member 2 | 55620 |
| 18728 | 4.171445 | 0.1854128 | 1.05E-03 | 0.0839146 | 3.750191 | H200002524 | WBP5 | WW domain binding protein 5 | 51186 |
| 9514 | 3.350498 | 0.294481 | 4.29E-03 | 0.1090499 | 3.75993 | H200017869 | PCDHGB6 | protocadherin gamma subfamily B, 6 | 56100 |
| 4791 | 3.762473 | 0.3918047 | 2.09E-03 | 0.0968513 | 3.784118 | H200010443 | PTK7 | protein tyrosine kinase 7 | 5754 |
| 11402 | 4.406715 | 0.360165 | 7.19E-04 | 0.0766926 | 3.7869 | H200020623 | LOC401445 | uncharacterized LOC401445 | 401445 |
| 17812 | 3.300035 | 0.1961029 | 4.69E-03 | 0.1118241 | 3.860115 | H200020985 | NA | NA | - |
| 6599 | 3.690333 | 0.345754 | 2.37E-03 | 0.0979394 | 3.903657 | H200009540 | USP31 | ubiquitin specific peptidase 31 | 57478 |
| 6642 | 4.357303 | 0.3550815 | 7.72E-04 | 0.077886 | 4.009031 | H200011470 | ATP2C1 | ATPase, Ca++ transporting, type 2C, member 1 | 27032 |
| 14544 | 3.545579 | 0.5735501 | 3.04E-03 | 0.1027813 | 4.020236 | H200017857 | GRIN3A | glutamate receptor, ionotropic, N-methyl-D-aspartate 3A | 116443 |
| 13476 | 4.334196 | 0.069618 | 8.06E-04 | 0.077886 | 4.191574 | H200010625 | NA | NA | - |
| 14151 | 4.064367 | 0.3732094 | 1.25E-03 | 0.0874811 | 4.413904 | H200020938 | NA | NA | - |
| 11220 | 3.512793 | 0.639223 | 3.22E-03 | 0.1035944 | 4.443077 | H200011907 | NA | NA | - |
| 14856 | 4.343429 | 0.2698878 | 7.93E-04 | 0.077886 | 4.444601 | H200010814 | NOXRED1 | NADP-dependent oxidoreductase domain containing 1 | 122945 |
| 19065 | 3.923807 | 0.3745357 | 1.58E-03 | 0.0914041 | 4.500533 | H200020782 | NA | NA | - |
| 17390 | 3.311922 | 0.7132189 | 4.59E-03 | 0.1111721 | 4.527097 | H200001153 | ARNT2 | aryl-hydrocarbon receptor nuclear translocator 2 | 9915 |
| 20320 | 3.333538 | 0.364154 | 4.42E-03 | 0.1097343 | 4.653405 | H200017534 | NA | NA | - |
| 12245 | 3.673422 | 0.2449061 | 2.43E-03 | 0.0979394 | 4.815553 | H200017257 | NA | NA | - |
| 21696 | 4.2538 | 0.3182634 | 9.20E-04 | 0.0821175 | 4.93923 | H200020889 | HDX | highly divergent homeobox | 139324 |
| 432 | 6.530722 | 0.2414303 | 3.84E-05 | 0.0482589 | 4.978954 | H200020318 | RNF32 | ring finger protein 32 | 140545 |
| 17688 | 3.912956 | 0.4280885 | 1.61E-03 | 0.0914041 | 4.995249 | H200015237 | NA | NA | - |
| 18844 | 3.309966 | 1.3280988 | 4.61E-03 | 0.1113453 | 5.02458 | H200008954 | CSRNP3 | cysteine-serine-rich nuclear protein 3 | 80034 |
| 12434 | 3.430108 | 0.4072911 | 3.73E-03 | 0.1058952 | 5.193843 | H200004770 | SLCO1A2 | solute carrier organic anion transporter family, member 1A2 | 6579 |
| 21207 | 3.820258 | 0.5007568 | 1.89E-03 | 0.0960209 | 5.348952 | H200019398 | NA | NA | - |
| 6147 | 3.363335 | 0.4594603 | 4.19E-03 | 0.1090499 | 5.550308 | H200009861 | NA | NA | - |
| 5717 | 3.537101 | 0.7908072 | 3.08E-03 | 0.1035911 | 6.061874 | H200011072 | MLLT4 | myeloid/lymphoid or mixed-lineage leukemia (trithorax homolog, Drosophila); translocated to, 4 | 4301 |
| 3431 | 4.346012 | 0.3663782 | 7.89E-04 | 0.077886 | 6.562232 | H200011014 | OPCML | opioid binding protein/cell adhesion molecule-like | 4978 |
| 15359 | 3.706929 | 0.5364418 | 2.30E-03 | 0.0979394 | 6.974943 | H200013183 | NA | NA | - |
| 951 | 3.491792 | 2.8341691 | 3.34E-03 | 0.1045639 | 7.041569 | H200001704 | VSIG4 | V-set and immunoglobulin domain containing 4 | 11326 |
| 16816 | 3.556816 | 0.3089413 | 2.98E-03 | 0.1024962 | 7.045774 | H200017179 | NA | NA | - |
| 10250 | 4.46178 | 0.2374721 | 6.59E-04 | 0.0765008 | 7.185146 | H200009189 | NA | NA | - |
| 8648 | 3.268068 | 0.5785536 | 4.97E-03 | 0.1129929 | 7.354741 | H200019948 | KCTD17 | potassium channel tetramerisation domain containing 17 | 79734 |
| 11851 | 3.463077 | 0.4536508 | 3.51E-03 | 0.1046203 | 8.065786 | H200020356 | NA | NA | - |
| 11806 | 3.628155 | 0.2421404 | 2.63E-03 | 0.1003799 | 8.281854 | H200018106 | WNT8A | wingless-type MMTV integration site family, member 8A | 7478 |
| 20154 | 3.353855 | 0.7852513 | 4.26E-03 | 0.1090499 | 8.55001 | H200008438 | FERMT1 | fermitin family member 1 | 55612 |
| 8862 | 4.3518 | 0.8666316 | 7.81E-04 | 0.077886 | 9.406844 | H200008619 | ATP8B4 | ATPase, class I, type 8B, member 4 | 79895 |
| 20481 | 3.513544 | 0.5960952 | 3.21E-03 | 0.1035944 | 10.23522 | H200004543 | IL17A | interleukin 17A | 3605 |
| 3093 | 3.344519 | 6.5072736 | 4.33E-03 | 0.1095379 | 14.38361 | H200016737 | CCL8 | chemokine (C-C motif) ligand 8 | 6355 |
| 3183 | 3.626329 | 0.4361048 | 2.64E-03 | 0.1004225 | 15.47182 | H200020941 | NA | NA | - |
| 18821 | 3.296636 | 1.1168917 | 4.72E-03 | 0.1118241 | 19.82145 | H200007488 | SPO11 | SPO11 meiotic protein covalently bound to DSB homolog (S. cerevisiae) | 23626 |
